# Odisha tribal family health survey: methods, tools, and protocols for a comprehensive health assessment survey

**DOI:** 10.3389/fpubh.2023.1157241

**Published:** 2023-07-10

**Authors:** Jaya Singh Kshatri, Asit Mansingh, A. K. Kavitha, Haimanti Bhattacharya, Dinesh Bhuyan, Debdutta Bhattacharya, Tanveer Rehman, Aparajita Swain, Debashis Mishra, Indramani Tripathy, Manas R. Mohapatra, Moushumi Nayak, Uttam Kumar Sahoo, Sanghamitra Pati

**Affiliations:** ^1^ICMR-Regional Medical Research Centre (Department of Health Research, Ministry of Health and Family Welfare, Government of India), Bhubaneswar, India; ^2^Scheduled Castes and Scheduled Tribes Research and Training Institute, Government of Odisha, Bhubaneswar, India

**Keywords:** tribal health, survey, indigenous population, aboriginal, India, NFHS

## Abstract

Tribal or indigenous communities have unique health behaviors, challenges, and inequities that nationally representative surveys cannot document. Odisha has one of India’s largest and most diverse tribal populations, constituting more than a fifth of the state. State and tribe-specific health data generation is recommended in India’s national roadmap of tribal health. The Odisha tribal family health survey (OTFHS) aims to describe and compare the health status of tribal communities in the state of Odisha and to estimate the prevalence of key maternal-child health indicators and chronic diseases. This paper summarizes the methodology, protocols, and tools used in this survey. This is a population-based cross-sectional survey with a multistage random sampling design in 13 (tribal sub-plan areas) districts of Odisha, India. We will include participants of all age groups and gender who belong to tribal communities. The sample size was calculated for each tribe and aggregated to 40,921, which will be collected from 10,230 households spread over 341 clusters. The survey data will be collected electronically in modules consisting of Village, Household, and Individual level questionnaires. The age-group-specific questionnaires were adapted from other national family health surveys with added constructs related to specific health issues of tribal communities, including-critical indicators related to infectious and non-communicable diseases, multimorbidity, nutrition, healthcare-seeking behavior, self-rated health, psycho-social status, maternal and child health and geriatric health. A battery of laboratory investigations will be conducted at the household level and the central laboratory. The tests include liver function tests, kidney function tests, lipid profile, iron profile, and seroprevalence of scrub typhus and hepatitis infections. The datasets from household questionnaires, field measurements and tests and laboratory reports will be connected using a common unique ID in the database management system (DBMS) built for this survey. Robust quality control measures have been built into each step of the survey. The study examines the data focused on different aspects of family health, including reproductive health, adolescent and child health, gender issues in the family, ageing, mental health, and other social problems in a family. Multistage random sampling has been used in the study to enable comparison between tribes. The anthropometric measurements and biochemical tests would help to identify the indicators of chronic diseases among various age groups of the population.

## Introduction

In India, indigenous or tribal groups constitute around 8.6% of the total population, with over 90% living in rural areas (1–3). Odisha is one of the most diverse states for indigenous populations, with 62 tribal groups and 22.8% of its population belonging to scheduled tribes amounting to over 10 million persons (4).

The public healthcare provisions for tribal populations remain subsumed in common rural healthcare programs. However, due to a unique geographical and socio-cultural milieu, there are substantial gaps in healthcare access and unmet needs in these groups compared to the rest of the population (3, 5, 6). Tribal people in India have worse health indicators than the general population (3). This is compounded by the lack of connectivity, health literacy, awareness and unique healthcare-seeking behavior among these groups (7, 8).

It is well recognized that high-quality and reliable data on demographics, socio-economic markers and health parameters are necessary for planning, targeting, and implementing interventions in specific vulnerable groups. There are limited data available on the health metrics of the tribal population of Odisha or India. Although regular surveys are being conducted across the country to estimate health indicators of the population, such as the national family health survey (NFHS), these national surveys are not targeted at any group. They, therefore, collect fewer samples and non-specific information from tribal populations. In statistical terms, these also tend to be underpowered for meaningful inferences to be made for these groups. A report from the expert committee on tribal health jointly issued by the Ministries of Health and Tribal affairs of India recognized this and suggested state and tribe-specific health data generation as a crucial step in policy planning (3).

The proposed Odisha tribal family health survey (OTFHS) is planned to fill these gaps in evidence. OTFHS aims to describe and compare the health status of tribal communities in the state of Odisha and to estimate the prevalence of key maternal-child health indicators and chronic diseases. It is the first state-level survey of tribals in India, where all the tribes of Odisha will be included and compared for the very first time. In this paper, we describe the methods and make public the tools used and protocols for OTFHS.

## Methods

### Design, setting and participants

**Figure 1:**
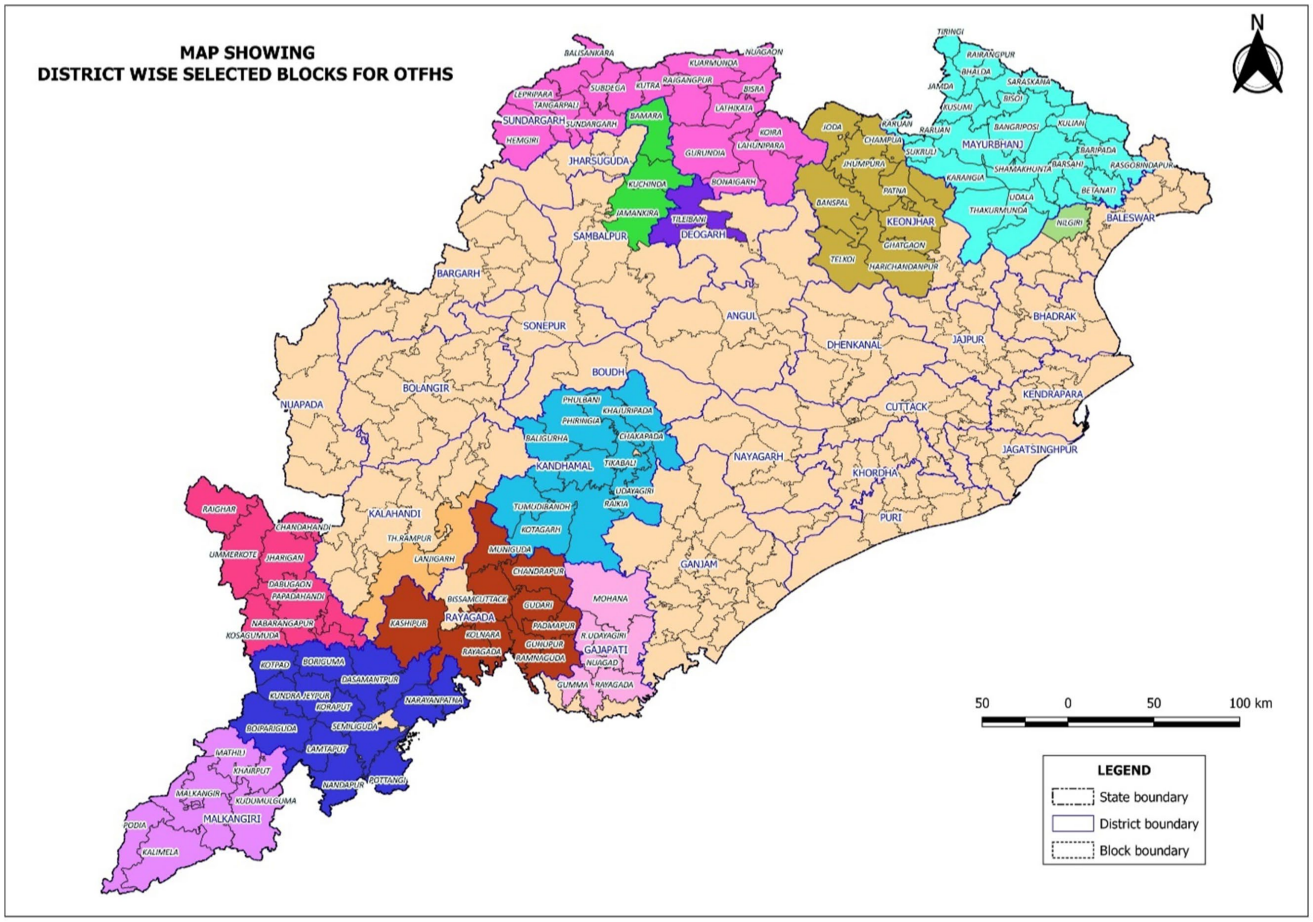
Map showing district wise selected blocks for Odisha tribal family health survey.

This study is a population-based cross-sectional survey with a multistage random sampling design carried out in the state of Odisha, India. There are 30 administrative districts in the state, among which 13 districts (119 sub-district level blocks) are tribal-dominated and included in the survey (Figure 1).

#### Study population

Participants of all age groups and gender who belong to one of the notified tribes, are permanent residents of the region and provide written consent will be included in the survey. Bedridden patients and study participants with recognizable cognitive impairments will be excluded. Data will be collected for 1 year from June 2022.

### Sample size calculations

Our sample size calculations were powered to get estimates of a proportion of people over 15 years with high or very high blood sugar (>140 mg/dL), which in rural Odisha, as per the NFHS-4 estimates, was 10.7% (9).The following standard formula for minimum sample size required (*n*) was used, where confidence levels of 95%, and *α* of 0.05 were preset:


$$\begin{array}{c} n=\left[\mathrm{DEFF}*Np\left(1-p\right)\right]/\left[\left(d^{2}/Z^{2}{}_{1-\alpha /2}\right)*\left(N-1\right)+p*\left(1-p\right)\right]\\ {}*\;\mathrm{Finite Population Adjustment} \end{array}$$

where *N* was the total tribe population, *p* was the expected proportion of high/very high blood sugar, and d was the relative precision set at 30%. A finite population correction (FPC) was applied where FPC was estimated with the formula $\sqrt{\left(N-n\right)/\left(N-1\right)}$ . The latest population sizes for each tribe were provided by the government tribal affairs department. For the estimation of design effect (DEFF), cluster size was fixed at 30 households, and a variable intra-class correlation of 0.019 that has been previously reported was assumed (10, 11). Assumed response rates were 70%, and household size was 4. The sample size was calculated for each tribe separately to enable comparisons. The aggregated sample size for the survey was 40,921, which will be collected from 10,230 households spread over 341 clusters across the state. The tribe-specific estimates are provided in Additional file 1.

### Sampling

The survey uses a multistage sampling design. The primary and secondary sampling units will be clusters and households, respectively. The survey population was stratified based on tribes in the first sampling stage. The sampling frame included all the 119 tribal-dominated blocks (from 13 districts) notified by the state government. As the tribal groups are not spread uniformly across the state, clusters required per district were proportionally allocated for each tribe. A village, notified hamlet or an urban local body ward was considered a “cluster”.The clusters from each district were selected by probability proportional to the size method. Clusters of smaller tribes who reside in limited geographical areas were purposively selected. In the next stage, households from the cluster are selected by systematic random sampling. The nearest village will be included if the cluster cannot meet the number of households required. In each selected household, all eligible study participants are invited to participate in the survey.

### Survey tools

The survey uses the following tools to collect data at the cluster, household, and individual levels:

1. Cluster/village level questionnaire—it collects data on the village demographics, availability and accessibility of health/educational facilities and other social welfare schemes, connectivity and critical environmental parameters.
2. Household level questionnaire—information collected includes members’ list, housing characteristics, water source, sanitation, ownership of assets, access to welfare schemes, health insurance, injury, death, and disability status of members.
3. Individual level questionnaires—following age-group specific questionnaires were prepared to capture major indicators from all domains of health:

- Age 0–4 years old
- Age 5–9 years old
- Age 10–19 years old female
- Age 10–19 years old male
- Age 20–59 years old women
- Age 20–59 years old men
- Age 60 & above old

The individual-level questionnaires aimed to provide estimates of key indicators related to infectious and non-communicable diseases, multimorbidity, nutrition, healthcare-seeking behavior, self-rated health, psycho-social status, maternal and child health and geriatric health. The indicators will be age and/or gender-specific, as shown in the Table 1 below.

**Table 1:** Categories of indicators and modules for data collection in Odisha tribal family health survey.

Categories of indicators	Age/gender classification of OTFHS modules							
	Age group (in years)	0–4	5–9	10–19		20–59		≥60
	Gender	Both	Both	Female	Male	Female	Male	Both
Immunization, vitamin-A supplementation & child feeding practices		✓						
Childhood diseases (diarrhea, ARI & anemia)		✓	✓					
Iron folic acid supplementation and deworming			✓	✓	✓			
Mid-day meal program & absenteeism			✓					
Tobacco use and alcohol consumption				✓	✓	✓	✓	✓
Chronic disease conditions				✓	✓	✓	✓	✓
Knowledge of HIV/AIDS				✓	✓	✓	✓	✓
Marriage and fertility				✓	✓	✓	✓	
Current use of family planning methods				✓		✓		
Maternal and child health				✓		✓		
Women’s empowerment				✓		✓		
Gender-based violence				✓		✓		
Functional limitations								✓
Quality of life								✓

Persons who identify as third-gender or transgenders will be administered a separate module.

The study tools were developed using the demographic and health survey, longitudinal aging study of India and NFHS questionnaires as a starting point and customized to meet the survey objectives (12–14). The questionnaires were translated into Odia. An expert committee provided feedback on the tools, which was incorporated in multiple iterative pre-testing rounds. The entire set of study tools is provided in Additional file 2, and an interviewers manual was developed to train the users (Additional file 3).

### Pre-testing of tools

The tools developed were pre-tested in two iterations of a pilot study involving 20 households in a similar population (Kandhamal district) but outside the sampling frame. Our aims were to:

- Document the feasibility and challenges in field-level preparations and sampling,
- Test the comprehension, duration and acceptance of each of the questionnaire modules, and
- Examine the procedures and bottlenecks in field laboratory practices (sample collection, processing, storage, transport and waste disposal).

We used observational checklists and qualitative debriefings of the participants and data collectors to gather data. We found that with the help of local village health workers, sampling methods and recruitment of participants were adequate. We identified questions with poor comprehension and most hesitancy (for sensitive questions) and reframed the language accordingly. The hesitation for providing blood samples was marked, especially in children, for which counselling measures were added to the training manual. A key challenge was timing the interviews to suit the availability of the household members who remain out of the house during the daytime for work. The visit schedule was also revised to early mornings or late evenings to cover these individuals.

### Survey procedures and human resources

The survey will be conducted by field teams placed at the district level. These teams would consist of two male and two female investigators, among whom two would be qualified laboratory technicians to carry out anthropometric and laboratory investigations and venipuncture, and one would be a public health professional who would lead the team. The teams would prepare a detailed monthly micro plan of field activities in consultation with the state government departments and local body representatives. A comprehensive manual of operational procedures (MOP) was developed to standardize the field-level activities of the investigators who will be trained using this document (Additional file 4). The field teams will carry out household visits according to the micro plan. The team lead will carry out the sampling, and the teams will collect data and samples from the participants recruited. Following the assessment of health conditions undertaken as part of the survey, any individual identified with abnormal findings or suggestive of potential healthcare needs will be provided with a detailed report (Additional file 4) in a standard format along with advice for referral to the nearest public health facility for further management. The help of village-level health workers and tribal leaders will be sought to bridge the communication gap with the community.

### Data and sample collection

Cluster-level data will be collected from the village health workers or tribal leaders, and household information by the self-declared head of the household. Data collection will be done through personal interviews in isolation of everyone using a custom-built android application. Data on children below 10 years of age will be collected from their mothers. Electronic data capture using tablets (offline) will be done to record information on each module separately, and a unique ID will be generated for each participant and household. Blood samples will be collected by venipuncture for the estimation of various health parameters at the household and laboratory-based assays. The same will then be transferred in gel vacutainers, and centrifuged at the district laboratory, and separated serum samples will be labelled with barcodes and transported to the laboratory facility at the host institute, Indian Council of Medical Research-Regional Medical Research Centre in Bhubaneswar, for further testing. The cold chain will be maintained during the transportation of samples.

### Anthropometric measurements and laboratory investigations

Anthropometric measurements will be done for all participants at the household level. These will include measurement of weight using an electronic weighing scale (separate for adults and children), measurement of height using a stadiometer (for those aged above 2 years) or infantometer (for those below 2 years of age) and measurement of waist-to-hip ratio (WHR) using an ergonomic circumference measuring tape. A dynamometer will be used to test the strength of grip in the dominant hand of all adults.

A digital haemoglobinometer will be used to test the haemoglobin of all participants. Among adult and adolescent participants, blood pressure will be measured (average of 3 readings) using automated digital blood pressure measuring machines and random blood glucose will be tested using a digital glucometer. Screening for hemoglobinopathies (sickle cell disease, trait and thalassemia)will be performed using a validated point-of-care device that uses a microchip-based cellulose acetate electrophoresis test for hemoglobin variant detection (15). The detailed procedures for all the household-level tests and sample handling are mentioned in the MOP, and the laboratory technicians would undergo comprehensive hands-on training before fieldwork.

The serum will be tested for different biochemical parameters using an automated biochemistry analyzer-EM 360 (Transasia Biomedical Pvt. Ltd.). The tests include liver function tests (direct and total bilirubin, serum albumin, alkaline phosphatase, SGOT and SGPT), kidney function tests (serum urea, creatinine), lipid profile (cholesterol, lipoproteins), iron profile (serum iron and total iron binding capacity). Tests to assess the seroprevalence of scrub typhus (IgG) and hepatitis B (HBsAg) infections will also be performed using enzyme-linked immunosorbent assay (16, 17).

### Data management

**Figure 2:**
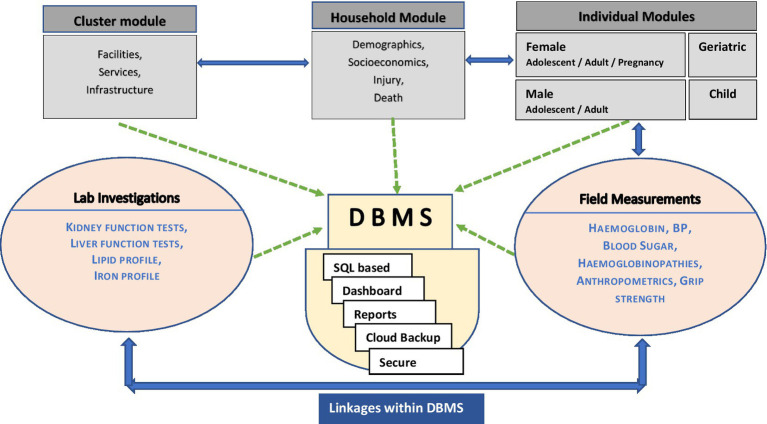
Data base management systems of the survey.

A custom-built database management system (DBMS) based on an open-source Structured Query Language (SQL) software platform has been developed for managing the data collected under the study. The datasets from household questionnaires, field measurements and tests and laboratory reports (linked with DBMS on a server) are connected using a common unique ID by the DBMS. Automated analytics, quality checks, performance reports and a real-time web-based dashboard are integrated into the functions of this DBMS, which is hosted in secure institutional servers (Figure 2).

### Quality control

#### Monitoring fieldwork

The survey quality will beensured at multiple levels, both internally and externally, by real-time quality checks, field monitoring visits for supportive supervision by investigators, and laboratory quality controls. Study progress for each team will be monitored centrally in real-time using the dashboard indicators integrated into DBMS (daily clusters completed, data collected, samples collected), and necessary feedback will be provided to the field teams. A separate android application to verify the data collected in the field (supervisor module) has been built to check the data collected for key ‘bell-weather’ questions against the answers recorded by the field investigators. A hierarchical model of randomly verifying 5% of data collected every month at each level is put in place (field investigators-field supervisors-internal investigators-external monitors). The external monitors will be recruited from local medical colleges and district health officials.

#### Laboratory quality controls

Internal and external steps have been included to maintain laboratory quality control and assurance at the field and laboratory levels. These include daily quality control runs for the biochemical parameters before testing the samples by following the instrument’s operating protocol, weekly maintenance of the instrument using acid/alkali auto wash, a quality audit by testing blindly selected previously processed samples, and the fresh samples and troubleshooting when necessary. For external audits, participation in external quality audit (EQA) will be undertaken with a nationally accredited laboratory.

#### Data quality checks

Measures to ensure data quality have been incorporated in the data collection and post-data collection phases. The field teams are instructed to upload all the data they collect daily to DBMS. A statistician will check for missing/faulty data entry before validating the data collected. Routine checks will be performed for duplication, completeness of crucial variables, estimates for indicators, missing values, and meta-data. Each field investigator is provided with a unique credential to log into the android application for data collection. Once the data is uploaded from the field, editing access for the DBMS remains exclusively with the statistician and the principal investigator. A log is maintained for all such edits made. We are following the guidance from National Guidelines for Data Quality in Surveys towards this end (18).

### Analysis plan

The necessary steps of cleaning and transforming raw data downloaded from the servers will be carried out before analysis. This involves reformatting, creating categories, correcting and combining the data sets and checking for data structures and outliers. Survey weights will be added to the data to account for variability due to design and sampling errors. To take into consideration the overall selection probability of each household participating in the survey, a design weight will be generated, which would be adjusted for household and individual non-response. Standard errors will be used to measure the sampling errors in the survey. For estimating standard errors for means, proportions, and other complex rates, we will use the Taylor linearization method (19). For variance estimation of more complex statistics such as total fertility rates and child mortality rates, the Jackknife repeated replication approach will be used (20). Descriptive analysis will be performed using R software packages to arrive at a list of tribe-specific indicators that have been predefined and provided in Additional file 5. Confidence levels, where applicable, will be set at 95%. As per our objectives, no hypothesis testing will be carried out in the primary data analysis.

## Discussion

This survey aims to provide data on a comprehensive list of health indicators in tribal groups from Odisha. To our knowledge, this is the first of its kind extensive survey in India targeting the tribal population exclusively and covering a wide array of health parameters from varied domains. Policymakers identified the need for the survey at the state level and recognized the lack of sufficient data on tribal groups in nationally representative surveys such as the NFHS, which has included a population of around 10,000 tribals from the state in its latest round, and which does not provide tribe wise segregated data (21).

The scope of the survey is broad as its objectives are to capture data on different aspects of family health, including reproductive health, adolescent and child health, gender issues in the family, ageing, mental health, and social problems a family faces (22). This survey would also provide a holistic picture of all additional determinants of tribal family health, such as living and working conditions, physical and psycho-social environment, education and economic factors, health practices, cultural factors and relationships (23).

Our sample size calculations were done to enable comparisons between tribes. The tribe population sizes ranged between a low of 13 individuals to over 1.6 million. We recognize the limitation of minimum sample size estimations in large surveys with varied objectives, including that the sample size would be underpowered for some and overpowered for other indicators (24). However, to account for logistical and resource constraints, we have aimed our calculations estimates of high blood sugar. A rarer outcome, such as a maternal mortality ratio, would necessitate the inclusion of entire tribes, leading to the redundancy of the sampling process. This limitation would be mitigated by the *post hoc* addition of design and survey weights prior to analyses.

We have translated (and back-translated) adapted tools used in other national surveys of India with modifications to suit our objectives. These were reviewed and rated by an expert committee. As these questionnaires were not using new “scales of measurement” specifically, we have not gone through an instrument’s systematic steps of validation. Instead, we carried out a pre-testing where these questions were tested for their comprehension, response, and feasibility. Our field measurements and laboratory investigations aimed to capture most of the commonly used chronic disease indicators in specific age groups. We included all of the measures provided in national surveys to enable comparisons with the general population while including additional measures unique to the tribal people, depending on feasibility. The complex nature of the collected data, needing interlinkages at the back end, requires a robust data management system built on an open-source SQL platform (25, 26).

Tribal communities are considered vulnerable groups, and additional care needs to be factored into the ethical considerations around research involving them (21). Recognizing this, we have involved all stakeholders right to the village level to protect the rights of tribals and are following the national ethical guideline before, during and after our survey.

This study would provide data on the reach of the health and social security programs and generate evidence for targeted interventions by multiple departments. The findings will also help to identify key behavioral and attitude-related research questions that can be answered in a future qualitative study among a targeted subset of the participants. The data architecture has been designed to allow repeated ‘rounds’ of surveys in future. While the survey can act as a baseline for a sample repository, this can form the foundation for further longitudinal studies and implementation research.

## Ethics statement

The studies involving human/animal participants were reviewed and approved by Institutional Human Ethics Committee (IHEC) of ICMR-Regional Medical Research Centre, Bhubaneswar. Before participating in the survey, written informed consent/assent form will be obtained from the study participants/participants’ legal guardians/next of kin.

## Author contributions

JK and SP were responsible for the conceptualization of the study. SP is the chief investigator and led the design of the study and the application for funding. JK led the preparation of this manuscript for submission. AM, AK, HB, and DiB have contributed to the design of the study and the grant applications and the writing of the manuscript. DeB and TR have advised on the sample size, and laboratory-based analysis, and developed the analytic plan. JK is the project co-PI. IT, MM, MN, and US reviewed the study proposal and are external evaluators of the study. AS and DM provided technical support for the project. All authors contributed to the article and approved the submitted version.

## Funding

This study is being funded by Scheduled Castes and Scheduled Tribes Research and Training Institute, Government of Odisha (Grant no: 1399/R-01/21). The funders have no influence on study methods, analysis, interpretation, or publication of results.

## Conflict of interest

The authors declare that the research was conducted in the absence of any commercial or financial relationships that could be construed as a potential conflict of interest.

## Publisher’s note

All claims expressed in this article are solely those of the authors and do not necessarily represent those of their affiliated organizations, or those of the publisher, the editors and the reviewers. Any product that may be evaluated in this article, or claim that may be made by its manufacturer, is not guaranteed or endorsed by the publisher.

## Abbreviations

AIDS: Acquired Immuno Deficiency Syndrome
ARI: Acute Respiratory Infection
DBMS: Database Management System
DEFF: Design Effect
dl: Deciliter
EQA: External Quality Audit
FPC: Finite Population Correction
HBsAg: Hepatitis B Surface Antigen
HIV: Human Immunodeficiency Virus
IgG: Immunoglobulin G
mg: Milligram
MOP: Manual of Operating Protocol
NFHS: National Family Health Survey
OTFHS: Odisha Tribal Family Health Survey
WHR: Waist to Hip Ratio
