# Supplementary material for: Odisha tribal family health survey: methods, tools, and protocols for a comprehensive health assessment survey
Source: Front Public Health. 2023 Jul 10;11:1157241. doi: 10.3389/fpubh.2023.1157241 (PMC10364047; doi:10.3389/fpubh.2023.1157241)
Supplement: Supplementary file 1 [file Table_1.DOCX]

**OTFHS Questionnaires**

**Cluster- Questionnaire**

| **IDENTIFICATION** |
| --- |
| DISTRICT NAME: DISTRICT CODE:  BLOCK NAME: BLOCK CODE:  CLUSTER NAME: CLUSTER CODE:  RESPONDENT NAME:   1. VILLAGE PRADHAN 2) ANY OTHER PANCHAYAT MEMBER 3) TEACHER 4) GRAM SEVAK 5) ANGANWADI WORKER (AWW) 6) ACCREDITED SOCIAL HEALTH ACTIVIST (ASHA) 7) OTHERS (SPECIFY__________________________)  INTERVIEW DATE: INTERVIEW START TIME: INTERVIEW END TIME:  NAME OF THE INVESTIGATOR:  CODE OF THE INVESTIGATOR:  SIGNATURE OF THE INVESTIGATOR: |

| **Sl.no.** | **Question** | **Response** | **Skip To** |
| --- | --- | --- | --- |
|  | Does the cluster belong to a Village/Hamlet/NAC/Municipality Corporation | Village……………………………………1  Hamlet…………………...........................2  Notified Area Council (NAC)……………3  Municipality Corporation………………..4 |  |
|  | Total households in the cluster |  |  |
|  | Total ST households in the cluster |  |  |
|  | Main source of drinking water in the village | **Piped water**  Piped into dwelling……………….….11  piped to yard/plot............…………….12  public tap/standpipe………………….13  Tube well or bore well…….…………21  **Dug well**  protected well………………………...31  un protected well…………….……….32  **Water from spring**  Protected spring………………………41  Unprotected spring………………… ..42 Rainwater……………………………..51  Tanker truck…………………………..61  Cart with small tank…………..……… 71  **Surface water (river/dam/ lake/pond/stream/canal/**  Irrigation channel)……………….........81  Bottled water……………………..……91  Hand pump……………………………92  Other _____________________96  (specify) |  |
|  | Is the drainage facility available in the village? | Yes……………………………………….1  No………………………………………..2 |  |
|  | The type of drainage facility in the village: | Yes No  A. Underground drainage…………..1 2  B. Open with outlet…………………1 2  C. Open without outlet.……………..1 2 |  |
|  | Is the electricity available in the village? | Yes……………………………………….1  No………………………………………..2 |  |
|  | Main source of irrigation in the village: | Tank/pond…………………………….. 01 Stream/river…………………………….02 Canal……………………………………03 Well……………………………………...04  Tube well………………………………..05  Other (specify)________________ 96 |  |
|  | Distance from the nearest town (kms) | Name of town_________  Distance |  |
|  | Distance from the district headquarters (kms) |  |  |
|  | Distance to the nearest railway station (kms) |  |  |
|  | Distance to the nearest bus station (kms) |  |  |
|  | Is the village connected by an all-weather road to the health facility? | YES NO  Sub Center……..………… 1 2  Primary Health Center…… 1 2  Block PHC…………… 1 2  Community Health Center/ 1 2  Rural Hospital…… 1 2  District Hospital……… 1 2 |  |
|  | \| Educational facilities available in the village: \| GOVT. \| \| PVT. \| \| Education facilities:  If not in the village, distance to nearest govt. facility available (in Km.) \| \| --- \| --- \| --- \| --- \| --- \| --- \| \|  \| Yes \| No \| Yes \| No \|  \| \| Primary School \| 1 \| 2 \| 1 \| 2 \|  \| \| Secondary School \| 1 \| 2 \| 1 \| 2 \|  \| \| Higher Secondary School \| 1 \| 2 \| 1 \| 2 \|  \| \| College \| 1 \| 2 \| 1 \| 2 \|  \| \| Non-formal Education \| 1 \| 2 \| 1 \| 2 \|  \| \| Religious School/ Madrasa/Mission School \| 1 \| 2 \| 1 \| 2 \|  \| \| Tribal Residence School/ Eklavya Model Residential School (EMRS) \| 1 \| 2 \| 1 \| 2 \|  \| | |  |
|  | \| Health facility available in the village \| \| \| Health facilities: If not in the village, distance to nearest facility available \| \| \| \| --- \| --- \| --- \| --- \| --- \| --- \| \|  \| \| \| Distance to the nearest health facility in km \| Whether accessible throughout the year \| \| \|  \| Yes \| No \|  \| Yes \| No \| \| Integrated Child Development Scheme (ICDS)- Anganwadi \| 1 \| 2 \|  \|  \|  \| \| Sub-Centre \| 1 \| 2 \|  \| 1 \| 2 \| \| Primary Health Centre (PHC) \| 1 \| 2 \|  \| 1 \| 2 \| \| Block PHC \| 1 \| 2 \|  \| 1 \| 2 \| \| Community Health Centre (CHC)/ Rural Health Centre \| 1 \| 2 \|  \| 1 \| 2 \| \| District/ Govt. Hospital \| 1 \| 2 \|  \| 1 \| 2 \| \| Govt. Dispensary \| 1 \| 2 \|  \| 1 \| 2 \| \| Private Clinic \| 1 \| 2 \|  \| 1 \| 2 \| \| Private Hospital \| 1 \| 2 \|  \| 1 \| 2 \| \| AYUSH Health Facility \| 1 \| 2 \|  \| 1 \| 2 \| \| Nutrition Rehabilitation Center \| 1 \| 2 \|  \| 1 \| 2 \| \| Health & Wellness Center \| 1 \| 2 \|  \| 1 \| 2 \| | |  |
|  | Availability of health provider in the Village (staying and/or visiting):   \| Anganwadi Worker (AWW) \| \| --- \| \| Accredited Social Health Activist (ASHA) \| \| Trained Birth Attendant (TBA) \| \| Auxiliary Nurse Midwife (ANM) \| \| Trained Lady Doctor \| \| Trained Private Doctor \| \| Unani Doctor \| \| Ayurvedic Doctor \| \| Homeopathic Doctor \| \| Sidha Doctor \| \| Registered Medical Practitioner (RMP) \| \| Traditional Healer \| \| Faith healer \| \| Untrained Dai \| \| Lady Health Visitor (LHV)  Bengali Doctor \| \| Others \|   (Specify _________________________)  M | \| Yes \| No \| IF YES, NUMBER \| \| --- \| --- \| --- \| \| 1 \| 2 \|  \| \| 1 \| 2 \|  \| \| 1 \| 2 \|  \| \| 1 \| 2 \|  \| \| 1 \| 2 \|  \| \| 1 \| 2 \|  \| \| 1 \| 2 \|  \| \| 1 \| 2 \|  \| \| 1 \| 2 \|  \| \| 1 \| 2 \|  \| \| 1 \| 2 \|  \| \| 1 \| 2 \|  \| \| 1 \| 2 \|  \| \| 1 \| 2 \|  \| \| 1 \| 2 \|  \| \| 1 \| 2 \|  \| |  |
|  | Whether the village was covered by Mobile Health Clinic | Yes ……………………….1  No…………………………2 | If ‘yes’ ask q. 18 |
|  | Number of visits of Mobile Health Clinic in the last three months: |  |  |
|  | Number of health or family welfare camps in the last six months in and around the village: |  |  |
|  | Does the cluster have the following facilities?   \|  \| YES \| NO \| \| --- \| --- \| --- \| \| Post / Telegraph office \| 1 \| 2 \| \| STD Booth \| 1 \| 2 \| \| Pharmacy/medical shop \| 1 \| 2 \| \| Bank \| 1 \| 2 \| \| PDS \| 1 \| 2 \| \| Adult Education Center \| 1 \| 2 \| \| Youth Club \| 1 \| 2 \| \| Mahila Mandal \| 1 \| 2 \| \| Self Help Groups \| 1 \| 2 \| \| Paan Shop \| 1 \| 2 \| \| Haat/Market \| 1 \| 2 \| \| Kirana/ general provision shop \| 1 \| 2 \| \| Credit Cooperative Society \| 1 \| 2 \| \| Agricultural Cooperative Society \| 1 \| 2 \| \| Milk Cooperative Society \| 1 \| 2 \| \| Fishermen’s Cooperative Society \| 1 \| 2 \| \| Mills / Small Scale Industries \| 1 \| 2 \| \| Others  Specify________________ \| 1 \| 2 \| | |  |
|  | During the last six months how many times cleaning, fogging drive was undertaken in the village? |  |  |
|  | Has Rogi Kalyan Samiti/Jana Arogya Samiti been constituted in the hospital of your area? | Yes ……………………….1  No…………………………2 |  |
|  | Have these programmes implemented the village?   \| Janani Shishu Suraksha Karyakaram (JSSK) \| \| --- \| \| Mid-day Meal Programme (MMP) \| \| Integrated Child Development Scheme (ICDS) \| \| National Rural Employment Guarantee Act (NREGA) \| \| Village Health, Sanitation and Nutrition Programme (VHNS) \| \| Basudha Scheme \| \| Pradhan Mantri Ujjwala Yojana (PMUY) \| \| Mission Shakti \| \| Pradhan Mantri Kaushal Vikas Yojana (PMKVY) \| \| Odisha Rural Development and Marketing Society (ORMAS) \| \| Odisha Live hood Mission (OLM) \| \| Other (Specify)____________ \| | \| PROGRAMMES IMPLEMENTED \| \| BENEFICIARIES IN LAST 1YEAR \| \| \| --- \| --- \| --- \| --- \| \| YES \| NO \| YES \| NO \| \| 1 \| 2 \| 1 \| 2 \| \| 1 \| 2 \| 1 \| 2 \| \| 1 \| 2 \| 1 \| 2 \| \| 1 \| 2 \| 1 \| 2 \| \| 1 \| 2 \| 1 \| 2 \| \| 1 \| 2 \| 1 \| 2 \| \| 1 \| 2 \| 1 \| 2 \| \| 1 \| 2 \| 1 \| 2 \| \| 1 \| 2 \| 1 \| 2 \| \| 1 \| 2 \| 1 \| 2 \| \| 1 \| 2 \| 1 \| 2 \| \| 1 \| 2 \| 1 \| 2 \| |  |
|  | Any outbreak/ communicable disease reported in the cluster during the last one year: | Malaria…………………………………..1  Diarrheal Disease…………………………2  Anthrax…………………………………. 3  Measles………………………………….4  Dengue…………………………………..5  Chicken pox……………………………… 6  Chikungunya …………………………….7  [Acute Encephalitis Syndrome (AES)](https://healthlibrary.askapollo.com/disease/acute-encephalitis-syndrome-aes/)……. 8  None …………………………………….9  Other communicable diseases……….10  (Specify______________________) |  |
|  | Was there any natural calamity reported affecting the cluster during the past one year? | Earthquake……………………………….1  Flood……………………………………..2  Cyclone…………………………………..3  Drought…………………………………..4  Landslides………………………………..5  None ……………………………….…….6  Other……………………………………..7 (Specify_________________________) |  |
|  | Does the cluster have a mobile network facility? | Yes ……………………………………1  no ……………………………………..2  Sometimes ……………………………3 |  |

****************************END*********************************************************

**Household - Questionnaire**

| **IDENTIFICATION** |
| --- |
| DISTRICT NAME: DISTRICT CODE:  BLOCK NAME: BLOCK CODE:  CLUSTER NAME: CLUSTER CODE:  TYPE OF PSU (URBAN=1, RURAL=2):  HOUSEHOLD NUMBER: HOUSEHOLD ID: INTERVIEW DATE: INTERVIEW START TIME: INTERVIEW END TIME:  NAME OF THE INVESTIGATOR:  CODE OF THE INVESTIGATOR:  SIGNATURE OF THE INVESTIGATOR:  INTERVIEW RESULT: 1) COMPLETED 2) NO HOUSEHOLD MEMBER AT HOME OR NO COMPETENT RESPONDENT AT HOME AT TIME OF VISIT 3) ENTIRE HOUSEHOLD ABSENT FOR EXTENDED PERIOD OF TIME 4) POSTPONED 5) REFUSED 6) DWELLING VACANT OR ADDRESS NOT A DWELLING 7) DWELLING DESTROYED 8) DWELLING NOT FOUND 9) OTHER (SPECIFY _________________________________) |

| No. | Question | Coding Categories | Skip |
| --- | --- | --- | --- |
|  | What is your name? |  |  |
|  | How old are you?  (Verify age with Biju Swasthya Kalyan Yojana (BSKY) card) | Age in Years:________  Date of birth:________ |  |
|  | Sex | Male……………………………..1  Female………………………….2  Transgender………………….3 |  |
|  | What is your religion? | Hindu…………………………….1  Muslim…………………………..2  Christian………………………..3  Other……………………………..9  (Specify) |  |
|  | Ethnic group | ST ………………………………………..1  PVTGs ………………………………….2 |  |
|  | Name of the Tribe /PVTG | **ST**  Bagata ……………………………..1  Baiga ………………………………..2  Banjara …………………………….3  Bathudi …………………………….4  Bhottada ………………………….5  Bhuiya ………………………………6  Bhumia ……………………………..7  Bhumij ……………………………...8  Bhunjia ………………………………9  Binjhal ……………………………….10  Binjhia ……………………………….11  Birhor ………………………………..12  BondoPoraja ……………………..13  Chenchu …………………………….14  Dal ……………………………………..15  Desia Bhumij ………………………16  Dharua ……………………………….17  Didayi ………………………………….18  Gadaba ………………………………..19  Gandia …………………………………20  Ghara …………………………………..21  Gond ……………………………………22  Ho ………………………………………..23  Holva ……………………………………24  Jatapu ………………………………….25  Juang ……………………………………26  Kandha Gauda ……………………...27  Kawar ……………………………………28  Kharia ……………………………………29  Kharwar ………………………………..30  Khond ……………………………………31  Kisan ………………………………………32  Kol ………………………………………….33  Kolah Loharas …………………….34  Kolha ………………………………….35  Koli ……………………………………..36  Kondadora ………………………….37  Kora …………………………………….38  Korua …………………………………..39  Kotia …………………………………….40  Koya ……………………………………..41  Kulis ………………………………………42  Lodha …………………………………….43  Madia ……………………………………44  Mahali …………………………………..45  Mankidi …………………………………46  Mankirdia ………………………………47  Matya …………………………………….48  Mirdhas ………………………………….49  Munda ……………………………………50  Mundari …………………………………51  Omanatya ………………………………52  Oraon …………………………………….53  Parenga …………………………………54  Paroja …………………………………….55  Pentia …………………………………….56  Rajuar …………………………………….57  Santal …………………………………….58  Saora ……………………………………..59  Shabar…………………………………….60  Sounti …………………………………….61  Tharua ……………………………………62  **PVTGs**  Birhor …………………………………….63  Bondo …………………………………….64  Chuktia Bhunjia……………………….65  Didayi …………………………………….66  Juang ……………………………………..67  Kharia …………………………………….68  Dongria Khond ……………………….69  Kutia Khond ……………………………70  Lanjia Saora ……………………………71  Lodha ……………………………………..72  Mankidia ………………………………..73  Paudi Bhuyan …………………………74  Saora ……………………………………..75 |  |
|  | How many members are in your household? |  |  |
|  | How many members are currently living in your household? |  |  |
|  | How many members of your family are currently engaged in any economic activity? |  |  |
|  | What is the montly expenditure of your household? | Rs. _______ |  |
|  | What is the gross annual income of your household from different sources? | RS:________ |  |
|  | Does your family possess any of the following cards?  Can you show me? | \|  \| Yes \| No \| \| --- \| --- \| --- \| \| Annapoorna Card \| 1 \| 2 \| \| Anna Antyodaya Yojana card \| 1 \| 2 \| \| Ration card (NFSA) \| 1 \| 2 \| |  |
|  | Do you or any of your family members own any land apart from the house you are living in? | Yes……………………..1  No………………………2 |  |
|  | Do you/your family have any of the health insurance schemes? | Yes……………………..1  No………………………2 | If ‘no’ skip q. 22 |
|  | What type of health scheme or health insurance? | \|  \| Yes \| No \| \| --- \| --- \| --- \| \| Employees State Insurance Scheme (ESIS) \| 1 \| 2 \| \| Central Govt. Health Schemes (CGHS) \| 1 \| 2 \| \| Biju Swasthya Kalyan Yojana (BSKY) \| 1 \| 2 \| \| Rashtriya Swasthya Bima Yojana (RSBY) \| 1 \| 2 \| \| Odisha State Treatment Fund (OSTF) \| 1 \| 2 \| \| Community Health Insurance Programme \| 1 \| 2 \| \| Other Health Insurance Through Employer \| 1 \| 2 \| \| Other Privately Purchased Commercial Health Insurance \| 1 \| 2 \| |  |
|  | Over the past 12 months, have any of the family members had any injury that warranted the need for medical treatment or has altered your daily activities for one day or more? | Yes …………………………………….1  No ……………………………………..2 | If ‘no’ skip q.24 |
|  | Type of injury | \|  \| Yes \| No \| \| --- \| --- \| --- \| \| Road traffic \| 1 \| 2 \| \| Fall \| 1 \| 2 \| \| Electric shock \| 1 \| 2 \| \| Struck/hit by person or object \| 1 \| 2 \| \| Stab \| 1 \| 2 \| \| Gunshot \| 1 \| 2 \| \| Burn \| 1 \| 2 \| \| Drowning/ near-drowning \| 1 \| 2 \| \| Bite \| 1 \| 2 \| \| Poisoning \| 1 \| 2 \| \| Others  (Specify____________) \| 1 \| 2 \| | If answer is Bite  ask q25  if answer is Poisoning  ask q26). |
|  | Type of bite | Dog ……………………………..1  Snake …………………………..2  Monkey ……………………….3  Rodent ………………………..4  Any other animal………….5  (Specify ______________) |  |
|  | Type of poisoning | Pesticide ………………………1  Others ………………………….2  (Specify _________________) |  |
|  | **If yes to any of the above**  How did the injury happen? Was it an accident, did someone else do this to the injured person or did the injured person do this to him/herself? | It was an accident (unintentional) …1  Someone else did it deliberately (intentional) ………………………………….2  The person did it to him/herself deliberately (self-inflicted)……………..3  Don’t know/ cannot remember ……..4 |  |
|  | Did the injured person need to be admitted in hospital for at least one night? | Yes ……………………………………………….1  No…………………………………………………2  Refused…………………………………………3  Don’t know / cannot remember……9 |  |
|  | Did the injured person die after their injury? | Yes……………………………………….1  No………………………………………..2 |  |
|  | Was there any birth of a child in your household last year? | Yes……………………………………….1  No………………………………………..2 |  |
|  | How many children took birth in the last one year? |  |  |
|  | How many births that occurred last year have been registered under civil authority? |  |  |
|  | Within last one years is there any child died |  |  |
|  | Does your household have an electricity connection? | Yes……………………………………….1  No………………………………………..2 |  |
|  | What is the source of water that your family members use for cleaning, washing purposes? | \|  \| Yes \| No \| \| --- \| --- \| --- \| \| River/pond \| 1 \| 2 \| \| Well without a lid \| 1 \| 2 \| \| Well with covered lid \| 1 \| 2 \| \| Tube well \| 1 \| 2 \| \| Pipe water \| 1 \| 2 \| \| Pipe water with bore well \| 1 \| 2 \| |  |
|  | What is the source of water that your family members use for drinking and cooking purposes? | \|  \| Yes \| No \| \| --- \| --- \| --- \| \| Surface water (River/pond/dam/ lake/stream/canal/irrigation channel) \| 1 \| 2 \| \| Piped into dwelling \| 1 \| 2 \| \| Piped into Yard/Plot \| 1 \| 2 \| \| Piped to neighbour \| 1 \| 2 \| \| Public tap/Stand pipe \| 1 \| 2 \| \| Tube well or borehole \| 1 \| 2 \| \| Protected dug well \| 1 \| 2 \| \| Unprotected dug well \| 1 \| 2 \| \| Protected spring \| 1 \| 2 \| \| Unprotected spring \| 1 \| 2 \| \| Rainwater \| 1 \| 2 \| \| Tanker truck \|  \|  \| \| Cart with small tank \| 1 \| 2 \| \| bottled water \| 1 \| 2 \| \| community RO plant \| 1 \| 2 \| \| Bottled water \| 1 \| 2 \| \| Others  (Specify ____________) \| 1 \| 2 \| |  |
|  | How do you make the water safe for drinking? | Directly drink source water…………1  Boil ………………………………………..2  filter ………………………………….3  Electronic water purifier…………………………4  Strain it through a cloth……………..5  Let it stand and settle …………………….6  Others ……………………………………………7  (Specify……………………………………….) |  |
|  | Does your household use a community toilet/ personal toilet? | No toilets ………………………………….1  Community /Shared toilet ………..2  Personal toilet …………………………..3 | skip |
|  | What kind of facility do members of your household usually use? | \|  \| Yes \| No \| \| --- \| --- \| --- \| \| Flush to piped sewer system \| 1 \| 2 \| \| Flush to septic tank \| 1 \| 2 \| \| Flush to pit latrine \| 1 \| 2 \| \| Flush to somewhere else \| 1 \| 2 \| \| Flush to don’t know where \| 1 \| 2 \| \| Ventilated improved pit (VIP)/biogas latrine \| 1 \| 2 \| \| Pit latrine with slab \| 1 \| 2 \| \| Pit latrine without slab/open pit \| 1 \| 2 \| \| Twin pit / composting toilet \| 1 \| 2 \| \| Dry toilet \| 1 \| 2 \| \| No facilities/uses open space or field \| 1 \| 2 \| \| Other  (Specify______________) \| 1 \| 2 \| | If response is  ‘No facilities/uses open space or field’ then ask q. 38 |
|  | Where is the toilet facility located? | In own dwelling …………………….1  In own yard/plot …………………..2  Elsewhere …………………………….3 |  |
|  | Do you share the toilet facility with other households? | Yes……………………………………….1  No………………………………………..2 | If ‘no’ then goto q.38 |
|  | Including your own household, how many households use this toilet facility? | Number:______  Don’t know ……………………98 |  |
|  | Do members of your household have access to a toilet facility? | Yes……………………………………….1  No………………………………………..2 |  |
|  | What kind of toilet facility do members of your household have access to? | Own toilet ……………………………1  Community toilet …………………2  Shared toilet with other household …….3 |  |
|  | How many rooms are there in your house? | Rooms: ____ |  |
|  | How many rooms in this household are used for sleeping? | Rooms: ____ |  |
|  | How many members of this household sleeping in one room? |  |  |
|  | What type of fuel does your household mainly use for cooking? | \|  \| Yes \| No \| \| --- \| --- \| --- \| \| Electricity \| 1 \| 2 \| \| LPG/Natural gas \| 1 \| 2 \| \| Biogas \| 1 \| 2 \| \| Kerosene \| 1 \| 2 \| \| Coal/ Lignite \| 1 \| 2 \| \| Charcoal \| 1 \| 2 \| \| Wood \| 1 \| 2 \| \| Straw/Shrubs/Grass \| 1 \| 2 \| \| Agricultural crop waste \| 1 \| 2 \| \| Dung cakes \| 1 \| 2 \| \| Solar \| 1 \| 2 \| \| No food cooked in the household \| 1 \| 2 \| \| Other  (Specify______________) \| 1 \| 2 \| |  |
|  | Do you and your family use iodised salt for cooking? | Yes……………………………………….1  No………………………………………..2 | Observation basis |
|  | Is the cooking usually done in the house, in a separate building or outdoors? | In the house………………………….1  In a separate building …………..2  Outdoors ……………………………..3  Others ………………………………….4  (Specify ___________________) |  |
|  | Does your household have a separate room that the members use for cooking? | Yes……………………………………….1  No………………………………………..2 |  |
|  | Does your household own any of the following animals? | \|  \| Yes \| No \| \| --- \| --- \| --- \| \| Cows/Bulls/Buffaloes/Yaks \| 1 \| 2 \| \| Horses/Donkeys/Mules \| 1 \| 2 \| \| Goas/Sheep \| 1 \| 2 \| \| Pigs \| 1 \| 2 \| \| Chickens/Ducks \| 1 \| 2 \| \| None \| 1 \| 2 \| \| Other  (Specify______________) \| 1 \| 2 \| |  |
|  | Where do you keep your livestock at your residence? | Separate animal shed …………….1  Inside the house …………………….2  Other ……………………………………..3  (Specify ___________________) |  |
|  | How do you dispose the solid & animal waste products? | Dispose in water bodies ……….1  Residing place ………………………2  Dig a pit ……………………………….3  Throw anywhere ………………….4  Common pit ………………………….5  Others(specify) …………………….6 |  |
|  | Have you ever vaccinated your livestock animals? | Yes……………………………………….1  No………………………………………..2 |  |
|  | Do you/any family members skin dead animals? | Yes……………………………………….1  No………………………………………..2 | If ‘yes’ ask q50 |
|  | Do you/any family members wear protective equipment’s for it? | Yes……………………………………….1  No………………………………………..2 |  |
|  | What do you/any family members do with the dead bodies of animal/ livestock? | Burial …………………………………..1  Throw them away ……………….2  Distribute among villagers …..3  Selling the carcass ……………….4  Report to closest veterinary office …..5  Incineration ……………………………………..6  Consume the meat …………………………..7  Others(specify) ………………………………..8 |  |
|  | Do you consume meat? | Yes……………………………………….1  No………………………………………..2 |  |
|  | What type of meat do you consume? | \|  \| Yes \| No \| \| --- \| --- \| --- \| \| Beef \| 1 \| 2 \| \| Pig \| 1 \| 2 \| \| Goat \| 1 \| 2 \| \| Sheep  Others (Specify) \| 1 \| 2 \| \| Other  (Specify______________) \| 1 \| 2 \| |  |
|  | How do you consume the meat? | \|  \| Yes \| No \| \| --- \| --- \| --- \| \| Cooked \| 1 \| 2 \| \| Roasted \| 1 \| 2 \| \| Raw \| 1 \| 2 \| \| Dried \| 1 \| 2 \| \| Other  (Specify______________) \| 1 \| 2 \| |  |
|  | How often do you consume the meat? | Once in a week ……………………1  Twice in week ……………………..2  Occasionally ………………………3  Daily ……………………………………4 |  |
|  | Do you preserve meat? | Yes……………………………………….1  No………………………………………..2 |  |
|  | If yes, how do you preserve meat? (Explain by respondent) | Refrigerate ………………………….1  Keep in water ……………………...2  Dried ……………………………………3  Any traditional methods ………4 |  |
|  | Do you consume animal blood? | Yes……………………………………….1  No………………………………………..2 |  |
|  | If yes, how do you consume? | \|  \| Yes \| No \| \| --- \| --- \| --- \| \| Cooked \| 1 \| 2 \| \| Raw \| 1 \| 2 \| \| Dried \| 1 \| 2 \| \| Other  (Specify______________) \| 1 \| 2 \| |  |
|  | What you do in case of animals suspected with anthrax/ other diseases in the them? | None ………………………………………1  Inform relevant authority ……….2  Disinfect the infected areas …….3  Treat with antibiotic ………………..4  Traditional methods ………………..5  Wait and see ……………………………6  Others (Specify) ………………………7 |  |
|  | How do you handle animals dead due to anthrax/disease? | Throw them away …………………..1  Consume the meat ………………….2  Burying ……………………………………3  Burning ……………………………………4  Both burning and burying ……….5  Inform health officials …………….6  Inform veterinary officials ………7  Others (Specify) ……………………..8 |  |
|  | If you throw the dead animals, then where do you do? | Forest ……………………………….1  Agricultural fields ………………2  Nearby house ……………………3  Outside village …………………..4  Drainage system ………………..5  Water bodies …………………….6  Others(Specify) …………………7 |  |
|  | If you bury the animals, then where do you do? | Forest ……………………………….1  Agricultural fields ………………2  Nearby house ……………………3  Outside village …………………..4  Others(Specify) ………………….5 |  |
|  | If you burn the animals, then where do you do? | Forest …………………………………1  Agricultural fields ………………..2  Nearby house ………………………3  Outside village ……………………..4  Others(Specify) …………………….5 |  |
|  | Does this household share a sleeping room with (this/these) animal (s)? | Yes……………………………………….1  No………………………………………..2 |  |
|  | Does your household have any mosquito nets that can be used while sleeping? | Yes……………………………………….1  No………………………………………..2 |  |
|  | How many mosquito nets does your household have? | Number of nets: |  |
|  | Did anyone sleep under this mosquito net last night? | Yes……………………………………….1  No………………………………………..2 |  |
|  | How many members slept under mosquito net last night? |  |  |
|  | Does your household have | YES NO  Electricity………………………………1 2  Mattress………………………………..1 2  Pressure cooker……………………..1 2  Chair………………………………………1 2  Cot or bed………………………………1 2  Table………………………………………1 2  Electric fan……………………………..1 2  Radio or transistor………………….1 2  Black and white television………1 2  Colour television…………………….1 2  Sewing machine……………………..1 2  mobile telephone……………………1 2  landline…………………………………..1 2  internet…………………………………..1 2  computer…………………………………1 2  refrigerator……………………………..1 2  air conditioner or cooler………….1 2  washing machine…………………….1 2  watch or clock…………………………1 2  bicycle…………………………………….1 2  motorcycle or scooter…………….1 2  animal drawn cart…………………..1 2  car…………………………………………..1 2  water pump…………………………….1 2  thresher…………………………………..1 2  tractor……………………………………..1 2 |  |
|  | Main material of the floor | **Natural Floor**  Mud/clay/earth…………………………..11  Sand……………………………………………12  Dung…………………………………………..13  **Rudimentary Floor**  Raw wood Planks…………………………21  Palm/Bamboo………………………………22  Brick…………………………………………….23  Stone…………………………………………..24  **Finished Floor**  Parquet or Polished wood…………..31  Vinyl or Asphalt……………………………32  Ceramic Tiles……………………………….33  Cement………………………………………..34  Carpet………………………………………….35  Polished Stone/Marble/Granite……36  Others………………………………………….96  (Specify) |  |
|  | Main Material of the Roof  (Record Observation) | Natural Roofing  No Roof……………………………...........11  Thatch/Palm leaf/ Reed/Grass…………………………………12  Mud……………………………………………13  Sod/Mud and Grass Mixture………14  Plastic/Polythene Sheeting…………………………………….15  **Rudimentary Roofing**  Rustic Mat……………………………………21  Palm/Bamboo………………………………22  Raw Wood Planks/Timber…………….23  Unburnt Brick……………………………….24  Loosely Packed Stone…………………..25  **Finished Roofing**  Metal/Gi……………………………………..31  Wood………………………………………….32  Calamine/Cement Fiber………………33  Asbestos Sheets………………………….34  RCC/RBC/Cement/Concrete………..35  Roofing Shingles………………………….36  Tiles……………………………………………..37  Slate…………………………………………….38  Burnt Brick……………………………………39  Others………………………………………….96  (Specify) |  |
|  | Main Material of the Exterior wall | **Natural Walls**  No Walls……………………………………..11  Cane /Palm/Trunks/Bamboo……….12  Mud…………………………………………….13  Grass /Reeds /Thatch…………………..14  **Rudimentary Walls**  Bamboo With Mud……………………..21  Stone With Mud………………………….22  Plywood………………………………………23  Cardboard……………………………………24  Unburnt Brick………………………………25  Raw Wood/Reused Wood……………26 Finished Walls Cement/Concrete………………………31  Stone With Lime/Cement…………..32  Burnt Bricks……………………………….33  Cement Blocks…………………………..34  Wood Planks/Shingles……………….35  Gi/Metal/Asbestos Sheets………….36  Others………………………………………….96  (Specify) |  |
|  | Does any usual resident of your household including you have any disability? | Yes………………………1  No……………………….2 | If ‘Yes’then askq78 |
|  | \| Sl. no \| Name \| Age (in years) \| Gender  (Male .1  Female .2) \| What type of disability does the individual have? \| \| \| \| \| \| \| --- \| --- \| --- \| --- \| --- \| --- \| --- \| --- \| --- \| --- \| \| 1 \|  \|  \|  \| Hearing (Yes…1  No….2) \| Speech  (Yes…1  No….2) \| Visual (Yes…1  No….2) \| Mental (Yes…1  No….2) \| Locomotor (Yes…1  No….2) \| Other \| \| \| 2 \|  \|  \|  \|  \|  \|  \|  \|  \|  \| \| \| 3 \|  \|  \|  \|  \|  \|  \|  \|  \|  \| \| \| 4 \|  \|  \|  \|  \|  \|  \|  \|  \|  \| \| \| 5 \|  \|  \|  \|  \|  \|  \|  \|  \|  \| \| | | |
|  | How often do you/ your family members eat the following food items?   \| Items \| Daily (1) \| Weekly (2) \| Occ. (3) \| Never (5) \| \| --- \| --- \| --- \| --- \| --- \| \| Milk or curd \|  \|  \|  \|  \| \| Pulses or beans \|  \|  \|  \|  \| \| Dark green leafy vegetables \|  \|  \|  \|  \| \| Fruits \|  \|  \|  \|  \| \| Eggs \|  \|  \|  \|  \| \| Fish \|  \|  \|  \|  \| \| Chicken or meat \|  \|  \|  \|  \| \| Fried foods \|  \|  \|  \|  \| \| Aerated drinks \|  \|  \|  \|  \| | | |
|  | Did any of your family members died last year? | Yes………………………1  No……………………….2 |  |
|  | How many persons died? | Total deaths:____ |  |

| Name of the person/people died | Was male/ female  (Male-1 &  Female-2) | Was death registered under Civil authority?  (Yes – 1 &  No – 2) | How old was when he/she died?  (Age in years) | Did die during pregnancy, during child birth or within two months after the end of pregnancy or child birth?  (Yes – 1 &  No – 2)  (Ask if female & died when 12 years or old) | Was the death due an accident, violence, poisoning, drowning, disaster, homicide or suicide?  (Yes – 1 &  No – 2) |
| --- | --- | --- | --- | --- | --- |
| 1. |  |  |  |  |  |
| 2. |  |  |  |  |  |
| 3. |  |  |  |  |  |
| 4. |  |  |  |  |  |
| 5. |  |  |  |  |  |
| 6. |  |  |  |  |  |
| 7. |  |  |  |  |  |
| 8. |  |  |  |  |  |

******************************END*********************************************************

**Age 0 – 4 Years Old Questionnaire**

| **IDENTIFICATION** | |
| --- | --- |
| DISTRICT NAME:  BLOCK NAME:  CLUSTER NAME:  TYPE OF PSU (URBAN=1, RURAL=2):  HOUSEHOLD NUMBER:  INDIVIDUAL ID:  INTERVIEWDATE:  INTERVIEW START TIME:  NAME OF THE INVESTIGATOR:  CODE OF THE INVESTIGATOR:  SIGNATURE OF THE INVESTIGATOR:  INTERVIEW RESULTS: 1) COMPLETED  2) NOT AT HOME  3) POSTPONED  4) REFUSED  5) PARTLY COMPLETED  6) INCAPACITATED  7) OTHERS  (SPECIFY __________________) | DISTRICT CODE:  BLOCK CODE:  CLUSTER CODE:  HOUSEHOLD ID:  INTERVIEW END TIME: |

| **Section 1:  Information about the child of age less than 5 years** | | | |
| --- | --- | --- | --- |
| **No.** | **Questions and Filters** | **Coding Categories** | **Skip** |
|  | How many children under five years do you have currently? | Number:________________ |  |
|  | Name of the child |  |  |
|  | What is the birth date of your child? | Date:__/__/____ (dd/mm/yyyy) |  |
|  | Age of the child | \| Year \| Month \| Day \| \| --- \| --- \| --- \| \|  \|  \|  \| |  |
|  | Birth order of the child | Birth order:_________________ |  |
|  | Sex of the child | Male…………………..1  Female………………...2 |  |
|  | Religion? | Hindu…………………………1  Muslim………………………2  Christian………………………3  No religion ……………………4  Other…………………………..9  (Specify __________________) |  |
|  | Ethnic group | Listed ST (Scheduled Tribe) …1  Primitive Vulnerable Tribal Groups (PVTGs)… …………2 |  |
|  | Mention tribe/PVTG name | **ST**  Bagata ……………………………..1  Baiga ………………………………..2  Banjara …………………………….3  Bathudi …………………………….4  Bhottada ………………………….5  Bhuiya ………………………………6  Bhumia ……………………………..7  Bhumij ……………………………...8  Bhunjia ………………………………9  Binjhal ……………………………….10  Binjhia ……………………………….11  Birhor ………………………………..12  BondoPoraja ……………………..13  Chenchu …………………………….14  Dal ……………………………………..15  Desia Bhumij ………………………16  Dharua ……………………………….17  Didayi ………………………………….18  Gadaba ………………………………..19  Gandia …………………………………20  Ghara …………………………………..21  Gond ……………………………………22  Ho ………………………………………..23  Holva ……………………………………24  Jatapu ………………………………….25  Juang ……………………………………26  Kandha Gauda ……………………...27  Kawar ……………………………………28  Kharia ……………………………………29  Kharwar ………………………………..30  Khond ……………………………………31  Kisan ………………………………………32  Kol …………………………………………33  Kolah Loharas …………………….34  Kolha ………………………………….35  Koli ……………………………………..36  Kondadora ………………………….37  Kora …………………………………….38  Korua …………………………………..39  Kotia …………………………………….40  Koya ……………………………………..41  Kulis ………………………………………42  Lodha …………………………………….43  Madia ……………………………………44  Mahali …………………………………..45  Mankidi …………………………………46  Mankirdia ………………………………47  Matya ……………………………………48  Mirdhas …………………………………49  Munda …………………………………..50  Mundari …………………………………51  Omanatya ………………………………52  Oraon …………………………………….53  Parenga …………………………………54  Paroja ……………………………………55  Pentia …………………………………….56  Rajuar ……………………………………57  Santal …………………………………….58  Saora ……………………………………..59  Shabar……………………………………60  Sounti ……………………………………61  Tharua …………………………………..62  **PVTGs**  Birhor …………………………………….63  Bondo ……………………………………64  Chuktia Bhunjia………………………65  Didayi …………………………………….66  Juang ……………………………………..67  Kharia …………………………………….68  Dongria Khond ………………………69  Kutia Khond …………………………..70  Lanjia Saora ……………………………71  Lodha …………………………………….72  Mankidia ……………………………….73  Paudi Bhuyan …………………………74  Saora ……………………………………..75 |  |
| **Section 2: Child Vaccinations and Vitamin A Supplementation** | | | |
| **No.** | **Questions** | **Coding Categories** | **Skip** |
|  | Check the age of the Child | 0-35 months ……………1  36-60 months …………..2 | If q201 is 2 then skip to 401 |
|  | Within the last six months, was (NAME) given a vitamin A dose like (this/any of these)?  SHOW COMMON AMPOULES/ CAPSULES/SYRUPS | Yes…………….1  No……………...2  Don’t know…….3 |  |
|  | Did (NAME) ever receive any vaccinations to prevent (him/her) from getting diseases, including vaccinations received in a Pulse Polio campaign? | Yes…………….1  No……………...2  Don’t know…….3 |  |
|  | Do you have a card or other document where (NAME)'s vaccinations are written down?  IF YES: May I see the card or other document where vaccinations are written down? | Yes, Seen…………1  Yes, not seen……...2  No card……………3 | If ‘yes, seen’ then got to 205 ow skip to 206 |

|  | Fill this from Child Vaccination Card | | |
| --- | --- | --- | --- |
|  |  | | |
| Vaccine Name | | Received  Yes ……..1  No ………2 | No. of doses |
| BCG | |  |  |
| POLIO | |  |  |
| DPT | |  |  |
| IPV | |  |  |
| HEPATITIS B | |  |  |
| PENTAVALENT | |  |  |
| ROTAVIRUS | |  |  |
| JE | |  |  |
| MCV | |  |  |
| DPT 1 BOOSTER | |  |  |
| VITAMIN A (LAST DOSE) | |  |  |
| VITAMIN A (NEXT-TO-LAST DOSE) | |  |  |

| **No.** | **Questions and Filters** | **Coding Categories** | **Skip** |
| --- | --- | --- | --- |
|  | A BCG vaccination against tuberculosis, that is, an injection in the arm or shoulder that usually causes a scar? | Yes…………….1  No……………...2  Don’t know…….3 |  |
|  | Polio vaccine, that is, drops in the mouth, including vaccine received in a Pulse Polio campaign? | Yes…………….1  No……………...2  Don’t know…….3 | If ‘no’ or don’t know then go to 210 |
|  | Was the first polio vaccine received in the first two weeks after birth or later? | First 2 weeks…….1  Later……………..2 |  |
|  | How many times was the oral polio vaccine given? | Number:__ |  |
|  | A DPT vaccination, that is, an injection given in the thigh or buttocks, sometimes at the same time as polio drops? | Yes…………….1  No……………...2  Don’t know…….3 | If ‘no’ or don’t know then go to 212 |
|  | How many times was a DPT vaccination given? | Number: |  |
|  | An IPV injection that is given in the upper arm to protect against polio, often at the same time as oral polio drops? | Yes…………….1  No……………...2  Don’t know…….3 | If ‘no’ or don’t know then go to 214 |
|  | How many times was an IPV vaccination given? | Number |  |
|  | A pentavalent vaccine/injection that is given in the thigh or buttocks, sometimes given at the same time as polio drops? | Yes…………….1  No……………...2  Don’t know…….3 | If ‘no’ or don’t know then go to 216 |
|  | How many times was a pentavalent vaccination given? | Number:__ |  |
|  | Was (NAME) given an injection at birth to prevent Hepatitis B? | Yes…………….1  No……………...2  Don’t know…….3 | If ‘no’ or don’t know then go to 219 |
|  | Was the first Hepatitis B vaccine received in the first two weeks after birth or later? | First two weeks…………1  Later…………………….2 |  |
|  | How many times was a Hepatitis B vaccination given? | Number: |  |
|  | Has (NAME) received a rotavirus vaccine, that is, liquid in the mouth to prevent diarrhea? | Yes…………….1  No……………...2  Don’t know…….3 | If ‘no’ or don’t know then go to 221 |
|  | IF YES: How many times was the rotavirus vaccine given? | Number:__ |  |
|  | Did (NAME) ever receive a JE vaccination against Japanese encephalitis? | Yes…………….1  No……………...2  Don’t know…….3 | If ‘no’ or don’t know then go to 223 |
|  | How many times was a JE vaccination given? | Number:__ |  |
|  | Was (NAME) ever given a measles or MMR injection - that is, a shot in the arm at the age of 9 months or older - to prevent (him/her) from getting measles? | Yes…………….1  No……………...2  Don’t know…….3 | If ‘no’ or don’t know then go to 225 |
|  | How many times was a measles or MMR vaccination given? | Number:__ |  |
|  | Was (NAME) ever given a DPT1 booster dose? | Yes…………….1  No……………...2  Don’t know…….3 |  |
|  | Where did (NAME) receive most of (his/her) vaccinations? | Public health sector……….1  Private health sector………2  AYUSH …………………..3  Others …………………….4  (Specify _________________) |  |
| **Section 3: Child Feeding Practices** | | | |
|  | Did you ever breastfeed (NAME)? | Yes…………………1  No………………….2 | If ‘no’ then go to 305 |
|  | How long after birth did you start breastfeeding (NAME)?  If less than one hour, record ‘00' hours. If less than 24 hours, record hours. Otherwise, record days. | Immediately…….000  Hours:__  Days:___ |  |
|  | Do you still breastfeed your child? | Yes…………………1  No………………….2 | If ‘yes’ skip to 305 |
|  | Why did you stop breastfeeding the child? | \|  \| Yes \| No \| \| --- \| --- \| --- \| \| Working lady \| 1 \| 2 \| \| Got pregnant \|  \|  \| \| Milk quantity reduced \|  \|  \| \| Family members asked to stop \|  \|  \| \| Cracked nipples / abscess \|  \|  \| \| Prolonged illness of mothers \|  \|  \| \| Others  (Specify_________) \|  \|  \| |  |
|  | Did (NAME) drink anything from a bottle with a nipple yesterday or last night? | Yes…………….1  No……………...2  Don’t know…….3 |  |
|  | Yesterday how many times did you breastfed your child (name)? | No of times: Day: ________  No of times: Night:_______ |  |
|  | At what age complementary feeding with the breastfeeding should be initiated to the child? | Month:___________  Don’t know…………………………98 |  |
|  | Now I would like to ask you about liquids or foods that (NAME) had yesterday during the day or at night.  I am interested in whether your child had the item I mention even if it was combined with other foods | \| Items \| Yes \| No \|  \| \| --- \| --- \| --- \| --- \| \| Plain water? \| 1 \| 2 \|  \| \| Juice or juice drinks? \| 1 \| 2 \|  \| \| Clear broth? \| 1 \| 2 \|  \| \| Milk such as tinned, powdered, or fresh animal milk? \| 1 \| 2 \| Number of times drank milk: \| \| Infant formula? \| 1 \| 2 \| Number of times drank milk: \| \| Any other liquids? \| 1 \| 2 \|  \| \| Yogurt? \| 1 \| 2 \| Number of times drank milk: \| \| Any commercially fortified baby food, e.g. Cerelac or ? \| 1 \| 2 \|  \| \| Any bread, roti, chapati, rice, noodles, biscuits, idli, or any other foods made from grains? \| 1 \| 2 \|  \| \| Any pumpkin, carrots, squash or sweet potatoes that are yellow or orange inside? \| 1 \| 2 \|  \| \| Any white potatoes, white yams, manioc, cassava, or any other foods made from roots? \| 1 \| 2 \|  \| \| Any dark green, leafy vegetables? \| 1 \| 2 \|  \| \| Any ripe mangoes, papayas, cantaloupe or jackfruit? \| 1 \| 2 \|  \| \| Any other fruits or vegetables? \| 1 \| 2 \|  \| \| Any liver, kidney, heart or other organ meat? \| 1 \| 2 \|  \| \| Any chickens, duck, or other birds? \| 1 \| 2 \|  \| \| Any other meat? \| 1 \| 2 \|  \| \| Any eggs? \| 1 \| 2 \|  \| \| Any fresh or dried fish or shellfish? \| 1 \| 2 \|  \| \| Any foods made from beans, peas, lentils, or nuts? \| 1 \| 2 \|  \| \| Any cheese or other food made from milk? \| 1 \| 2 \|  \| \| Any other solid, semi-solid, or soft food? \| 1 \| 2 \|  \| |  |
|  | How many times did (NAME) eat solid, semi-solid, or soft foods yesterday during the day or at night? | Number of times:__  Don’t know………….8 |  |
| **Section 4: Treatment of Childhood Diseases** | | | |
|  | Does the child have any of the following syndrome /disability | \|  \| Yes \| No \| \| --- \| --- \| --- \| \| Cleft lip (treated/ untreated) \| 1 \| 2 \| \| Cleft palate (treated/ untreated) \| 1 \| 2 \| \| Down Syndrome \| 1 \| 2 \| \| Congenital heart defects \| 1 \| 2 \| \| Oral health problems (Dental) \| 1 \| 2 \| \| Dwarfism \| 1 \| 2 \| \| Autism \| 1 \| 2 \| \| Epilepsy \| 1 \| 2 \| \| Abnormality is Vision/ speech/ hearing \| 1 \| 2 \| \| Mentally Challenged \| 1 \| 2 \| \| Sickle cell disease \| 1 \| 2 \| |  |
|  | Does the child have any of the following symptoms? (In last one month)?   \| Fever \| Yes \| No \| \| --- \| --- \| --- \| \| Vomiting \| 1 \| 2 \| \| Cough & Cold \| 1 \| 2 \| \| Diarrhea \| 1 \| 2 \| \| Pneumonia \| 1 \| 2 \| \| Nausea \| 1 \| 2 \| \| Pain   1. Joint 2. Hands 3. Feet 4. Muscle \| 1 \| 2 \| \| Body ache \| 1 \| 2 \| \| Headache \| 1 \| 2 \| \| Stomachache \| 1 \| 2 \| \| Anemia \| 1 \| 2 \| \| Swelling   1. Hands 2. Feet \| 1 \| 2 \| \| Frequent infections \| 1 \| 2 \| \| Vision problem \| 1 \| 2 \| \| Weakness \| 1 \| 2 \| \| Dark Urine \| 1 \| 2 \| \| Yellowing of the eyes and skin \| 1 \| 2 \| \| Jaundice \| 1 \| 2 \| \| Tiredness \| 1 \| 2 \| \| Loss of appetite \| 1 \| 2 \| \| Loss of sleep \| 1 \| 2 \| \| Excessive thirst \| 1 \| 2 \| \| Blood in stool \| 1 \| 2 \| \| Confusion \| 1 \| 2 \| \| Anxiety \| 1 \| 2 \| \| Disturbed sleep \| 1 \| 2 \| \| Swelling in abdomen \| 1 \| 2 \| \| Abdominal pain \| 1 \| 2 \| | |  |
|  | (Ask q403, 404 & 405 if the child suffered from diarrhea.)  What was given to the child during the last episode of diarrhea? | \|  \| Yes \| No \| \| --- \| --- \| --- \| \| ORS packet \|  \|  \| \| Home made ORS \|  \|  \| \| Zinc tablets \|  \|  \| \| Gruel made from rice (or other local grain) \|  \|  \| \| Others  (Specify_______) \|  \|  \| \| Can’t say \|  \|  \| |  |
|  | During diarrhea, how much liquid was given to drink? | Much less…………….1  Somewhat less……….2  About the same……....3  More…………………4  Nothing to drink……..5  Don't know…………..8 |  |
|  | \|  \| diarrhea \| \| Fever \| \| \| --- \| --- \| --- \| --- \| --- \| \| Yes \| No \| Yes \| No \| \| Home \| 1 \| 2 \| 1 \| 2 \| \| ASHA/AWW \| 1 \| 2 \| 1 \| 2 \| \| ANM/SC \| 1 \| 2 \| 1 \| 2 \| \| PHC/ HWC \| 1 \| 2 \| 1 \| 2 \| \| CHC/District hospital \| 1 \| 2 \| 1 \| 2 \| \| Private hospital \| 1 \| 2 \| 1 \| 2 \| \| AYUSH \| 1 \| 2 \| 1 \| 2 \| \| Did not take advice or treatment \| 1 \| 2 \| 1 \| 2 \| \| Others  (Specify________) \| 1 \| 2 \| 1 \| 2 \|   Ask this question if the child suffered from diarrhea/fever  Where did you seek advice or treatment when the child suffered from diarrhea/ fever? | |  |
|  | Ask q406 if the child detected anemia positive.  If the child found anemia “positive” what advice was given by doctor/nurse/ANM? | Medical advice …………1  Dietary advice …………2  Both ……………………3 |  |
|  | Has the child ever been tested for blood sugar? | Yes ……………………1  No ……………………..2 | If ‘no’ then skip to section5 |
|  | If “yes” when blood sugar was checked last time? | Month:  Year: |  |
|  | What was the result of blood sugar test? | Low ………………………1  Normal …………………2  High …………………...3 |  |
|  | If the answer is “high” what advice was given by doctor/nurse/ANM? | Medical advice …………1  Dietary advice …………2  Both ……………………3 |  |
|  | **Section 5: Biomarker** | | |
|  | Weight in Kilogram | \| Kg: \|  \|  \|  \| . \|  \|  \| \| --- \| --- \| --- \| --- \| --- \| --- \| --- \|   Not present………….994  Refused………………..995  Other……………………996 |  |
|  | Height in Centimeters | \| Cm: \|  \|  \|  \| . \|  \|  \| \| --- \| --- \| --- \| --- \| --- \| --- \| --- \|   Not present………….994  Refused………………..995  Other……………………996 |  |
|  | Waist circumference in centimeters | \| Cm: \|  \|  \|  \| . \|  \|  \| \| --- \| --- \| --- \| --- \| --- \| --- \| --- \|   Not present………….994  Refused………………..995  Other……………………996 |  |
|  | Hip circumference in centimeters | \| Cm: \|  \|  \|  \| . \|  \|  \| \| --- \| --- \| --- \| --- \| --- \| --- \| --- \|   Not present………….994  Refused………………..995  Other……………………996 |  |
|  | Isometric hand grip strength in Kilogram | \| Kg: \|  \|  \|  \| . \|  \|  \| \| --- \| --- \| --- \| --- \| --- \| --- \| --- \|   Not present………….994  Refused………………..995  Other……………………996 |  |
|  | When the child has taken food last? | 1 hour …………………1  1 to 2 hours ……………2  More than 2 hours …….3 |  |
|  | Record blood glucose in MG/DL | \| mg/dl: \|  \|  \|  \| \| --- \| --- \| --- \| --- \|   Refused …………………………….995  Other…………………………………996  Not tested…………………………997 |  |
|  | Record hemoglobin level | \| g/dl: \|  \|  \|  \| . \|  \| \| --- \| --- \| --- \| --- \| --- \| --- \|   Refused …………………………….995  Other…………………………………996  Not tested…………………………997 |  |
|  | Record the SCD/SCT percentage (%) here | \| Haemoglobin A2/C/E \| % \| \| --- \| --- \| \| Haemoglobin S \| % \| \| Haemoglobin F \| % \| \| Haemoglobin A \| % \|   Refused …………………………….995  Other…………………………………996  Not tested…………………………997 |  |
|  | **Section 6: Rating of health** | |  |
| 601. | We would like to know how good or bad your child health is TODAY.  This scale is numbered from 0 to 100  100 means the best health you can imagine. 0 means the worst health you can imagine.  Mark an X on the scale to indicate how your health is TODAY  Now, please write the number you marked on the scale in the box below. | Your Health Today = |  |

************************************END******************************************

**Age 5 – 9 Years Old Questionnaire**

| **IDENTIFICATION** | |
| --- | --- |
| DISTRICT NAME:  BLOCK NAME:  CLUSTER NAME:  TYPE OF PSU (URBAN=1, RURAL=2):  HOUSEHOLD NUMBER:  INDIVIDUAL ID:  INTERVIEW DATE:  INTERVIEW START TIME:  NAME OF THE INVESTIGATOR:  CODE OF THE INVESTIGATOR:  SIGNATURE OF THE INVESTIGATOR:  INTERVIEW RESULTS: 1) COMPLETED  2) NOT AT HOME  3) POSTPONED  4) REFUSED  5) PARTLY COMPLETED  6) INCAPACITATED  7) OTHERS  (SPECIFY ________) | DISTRICT CODE:  BLOCK CODE:  CLUSTER CODE:  HOUSEHOLD ID:  INTERVIEW END TIME: |

| **Section 1:  Information about the child** | | | |
| --- | --- | --- | --- |
| **No.** | **Questions and Filters** | **Coding Categories** | **Skip** |
|  | How many children age 5 to 9 years do you have currently? | **Number:________________** |  |
|  | Name of the child | **__________________** |  |
|  | What is the birth date of your child? | Date :__/__/____ (dd/mm/yyyy) |  |
|  | Age of the child in completed years | Age in years:_______________ |  |
|  | Birth order of the child | Birth order:_________________ |  |
|  | Sex of the child | Male…………………..1  Female………………...2 |  |
|  | Mention religion? | Hindu…………………………….1  Muslim…………………………..2  Christian…………………………3  No religion ………………………4  Other……………………………..9  (Specify ___________________________) |  |
|  | Ethnic group | Listed ST (Scheduled Tribe)………1  Primitive Vulnerable Tribal Groups (PVTGs) …2 |  |
|  | Mention tribe/PVTG name | **ST**  Bagata ……………………………..1  Baiga ………………………………..2  Banjara …………………………….3  Bathudi …………………………….4  Bhottada ………………………….5  Bhuiya ………………………………6  Bhumia ……………………………..7  Bhumij ……………………………...8  Bhunjia ………………………………9  Binjhal ……………………………….10  Binjhia ……………………………….11  Birhor ………………………………..12  BondoPoraja ……………………..13  Chenchu …………………………….14  Dal ……………………………………..15  Desia Bhumij ………………………16  Dharua ……………………………….17  Didayi ………………………………….18  Gadaba ………………………………..19  Gandia …………………………………20  Ghara …………………………………..21  Gond ……………………………………22  Ho ………………………………………..23  Holva ……………………………………24  Jatapu ………………………………….25  Juang ……………………………………26  Kandha Gauda ……………………...27  Kawar ……………………………………28  Kharia ……………………………………29  Kharwar ………………………………..30  Khond ……………………………………31  Kisan ………………………………………32  Kol …………………………………………33  Kolah Loharas …………………….34  Kolha ………………………………….35  Koli ……………………………………..36  Kondadora ………………………….37  Kora …………………………………….38  Korua …………………………………..39  Kotia …………………………………….40  Koya ……………………………………..41  Kulis ………………………………………42  Lodha …………………………………….43  Madia ……………………………………44  Mahali …………………………………..45  Mankidi …………………………………46  Mankirdia ………………………………47  Matya ……………………………………48  Mirdhas …………………………………49  Munda …………………………………..50  Mundari …………………………………51  Omanatya ………………………………52  Oraon …………………………………….53  Parenga …………………………………54  Paroja ……………………………………55  Pentia …………………………………….56  Rajuar ……………………………………57  Santal …………………………………….58  Saora ……………………………………..59  Shabar……………………………………60  Sounti ……………………………………61  Tharua …………………………………..62  **PVTGs**  Birhor …………………………………….63  Bondo ……………………………………64  Chuktia Bhunjia………………………65  Didayi …………………………………….66  Juang ……………………………………..67  Kharia …………………………………….68  Dongria Khond ……………………….69  Kutia Khond …………………………..70  Lanjia Saora ……………………………71  Lodha …………………………………….72  Mankidia ……………………………….73  Paudi Bhuyan …………………………74  Saora ……………………………………..75 |  |
|  | Is child currently going to school? | Yes…………………….1  No……………………..2 |  |
|  | In which class the child is studying? |  |  |
| **Section 2: Treatment of Childhood Diseases** | | | |
|  | Has the child ever diagnosed with any of these disease conditions? (Syndrome /disability)? | \|  \| Yes \| No \| \| --- \| --- \| --- \| \| Cleft lip (treated/ untreated) \| 1 \| 2 \| \| Cleft palate (treated/ untreated) \| 1 \| 2 \| \| Down Syndrome \| 1 \| 2 \| \| Congenital heart defects \| 1 \| 2 \| \| Oral health problems (Dental) \| 1 \| 2 \| \| Dwarfism \| 1 \| 2 \| \| Malnourished \| 1 \| 2 \| \| Autism \| 1 \| 2 \| \| Epilepsy \| 1 \| 2 \| \| Abnormality in Vision \| 1 \| 2 \| \| Abnormality in speech \| 1 \| 2 \| \| Abnormality in hearing \| 1 \| 2 \| \| Mentally Challenged \| 1 \| 2 \| |  |
|  | Does the child have any of the following symptoms? (In last one month)?   \| Fever \| Yes \| No \| \| --- \| --- \| --- \| \| Vomiting \| 1 \| 2 \| \| Cough & Cold \| 1 \| 2 \| \| Diarrhea \| 1 \| 2 \| \| Pneumonia \| 1 \| 2 \| \| Nausea \| 1 \| 2 \| \| Pain   1. Joint 2. Hands 3. Feet 4. Muscle \| 1 \| 2 \| \| Body ache \| 1 \| 2 \| \| Headache \| 1 \| 2 \| \| Stomachache \| 1 \| 2 \| \| Anemia \| 1 \| 2 \| \| Swelling   1. Hands 2. Feet \| 1 \| 2 \| \| Frequent infections \| 1 \| 2 \| \| Vision problem \| 1 \| 2 \| \| Weakness \| 1 \| 2 \| \| Dark Urine \| 1 \| 2 \| \| Yellowing of the eyes and skin \| 1 \| 2 \| \| Jaundice \| 1 \| 2 \| \| Tiredness \| 1 \| 2 \| \| Loss of appetite \| 1 \| 2 \| \| Loss of sleep \| 1 \| 2 \| \| Excessive thirst \| 1 \| 2 \| \| Blood in stool \| 1 \| 2 \| \| Confusion \| 1 \| 2 \| \| Anxiety \| 1 \| 2 \| \| Disturbed sleep \| 1 \| 2 \| \| Swelling in abdomen \| 1 \| 2 \| \| Abdominal pain \| 1 \| 2 \| | |  |
|  | (Ask q203, 204 & 205 if the child suffered from diarrhea.)  What was given to the child during the last episode of diarrhea? | \|  \| Yes \| No \| \| --- \| --- \| --- \| \| ORS packet \|  \|  \| \| Home made ORS \|  \|  \| \| Zinc tablets \|  \|  \| \| Gruel made from rice (or other local grain) \|  \|  \| \| Others  (Specify_______) \|  \|  \| \| Can’t say \|  \|  \| |  |
|  | During diarrhea, how much liquid was given to drink? | Much less…………….1  Somewhat less……….2  About the same……....3  More…………………4  Nothing to drink……..5  Don't know…………..8 |  |
|  | Ask this question if the child suffered from diarrhea/fever  Where did you seek advice or treatment when the child suffered from diarrhea/ fever? | |  |
|  | \|  \| diarrhea \| \| Fever \| \| \| --- \| --- \| --- \| --- \| --- \| \| Yes \| No \| Yes \| No \| \| Home \| 1 \| 2 \| 1 \| 2 \| \| ASHA/AWW \| 1 \| 2 \| 1 \| 2 \| \| ANM/SC \| 1 \| 2 \| 1 \| 2 \| \| PHC/ HWC \| 1 \| 2 \| 1 \| 2 \| \| CHC/District hospital \| 1 \| 2 \| 1 \| 2 \| \| Private hospital \| 1 \| 2 \| 1 \| 2 \| \| AYUSH \| 1 \| 2 \| 1 \| 2 \| \| Did not take advice or treatment \| 1 \| 2 \| 1 \| 2 \| \| Others  (Specify________) \| 1 \| 2 \| 1 \| 2 \|   Ask this | |  |
|  | Ask q206 if the child detected anemia positive.  If the child found anemia “positive” what advice was given by doctor/nurse/ANM? | Medical advice …………1  Dietary advice …………2  Both ……………………3 |  |
|  | Has the child ever been tested for blood sugar? | Yes ……………………1  No ……………………..2 | If ‘no’ then skip to section5 |
|  | If “yes” when blood sugar was checked last time? | Month:  Year: |  |
|  | What was the result of blood sugar test? | Low ………………………1  Normal …………………2  High …………………...3 |  |
|  | If the answer is “high” what advice was given by doctor/nurse/ANM? | Medical advice …………1  Dietary advice …………2  Both ……………………3 |  |
| **Section 3: IFA Supplementation and deworming medications** | | | |
|  | Do you receive Iron and Folic Acid (IFA) supplement / tablets for your child? | Yes……………………………..1  No………………………………2  Don’t know…………………9 | If ‘yes  go to 206 |
|  | \| Source of getting  Anganawadi ..1  School ……...2  ASHA………3  Others …….4  (Specify____) \| Frequency of receiving IFA?  Daily……………1  More than once in a week………………2  Weekly……………3  Monthly…………..4  Quarterly…………5  Half Yearly……….6  Yearly………….....7  Don’t know……….9 \| Do you consume IFA supplements?  Yes…………1  No………….2 \| Do you consume IFA supplement as per recommendation?  Yes …………...1  No ……….........2  Don’t know …..3 \| \| --- \| --- \| --- \| --- \| \|  \|  \|  \|  \| | |  |
|  | Do you receive deworming tablets / syrup? | Yes………………………………1  No……………………………….2 | If ‘no’ then go to 401 |
|  | \| Source of getting  Anganawadi 1  School ……2  ASHA……3  Others ……4  (Specify____) \| Frequency of receiving?  Daily…………………1  More than once in a week2  Weekly…………………3  Monthly……………….4  Quarterly………………..5  Half Yearly…………...6  Yearly…………………7  Don’t know……………9 \| Do you consume?  Yes……1  No…….2 \| Do you consume supplement as per recommendation?  Yes ………………....1  No …………..........2  Don’t know …….3 \| \| --- \| --- \| --- \| --- \| \|  \|  \|  \|  \| | |  |
| **Section 4: Mid-day Meal Programme (MDM) & Absenteeism** | | | |
|  | In school do you receive mid-day meal (MDM)? | Yes…………………………..1  No……………………………2 | If ‘no’ then go to 405 |
|  | How often do you eat the mid-day meal (MDM) in school in a week? | ___Days |  |
|  | Do you like the mid-day meal that is given in school? | Yes…………………………..1  No……………………………2 |  |
|  | Why do you not like the mid-day meal daily that is given in school? | Not tasty………………….......1  Do not like the menu…………2  Less quantity…………………3  Dirty (the way it is served)…..4  Others…………………………..5  (Specify______________) |  |
|  | Did you discontinue school in the last 15 days? | Yes…………………………..1  No……………………………2 | If ‘no’ then end |
|  | Did you discontinue school while you were sick in the last 15 days? | Yes…………………………..1  No……………………………2 |  |
|  | How many days were you absent due to sickness in the last 15 working school days? | ____days |  |
|  | **Section 5: Biomarker** | |  |
|  | Weight in Kilogram | \| Kg: \|  \|  \|  \| . \|  \|  \| \| --- \| --- \| --- \| --- \| --- \| --- \| --- \|   Not present………….994  Refused………………..995  Other……………………996 |  |
|  | Height in Centimeters | \| Cm: \|  \|  \|  \| . \|  \|  \| \| --- \| --- \| --- \| --- \| --- \| --- \| --- \|   Not present………….994  Refused………………..995  Other……………………996 |  |
|  | Waist circumference in centimeters | \| Cm: \|  \|  \|  \| . \|  \|  \| \| --- \| --- \| --- \| --- \| --- \| --- \| --- \|   Not present………….994  Refused………………..995  Other……………………996 |  |
|  | Hip circumference in centimeters | \| Cm: \|  \|  \|  \| . \|  \|  \| \| --- \| --- \| --- \| --- \| --- \| --- \| --- \|   Not present………….994  Refused………………..995  Other……………………996 |  |
|  | When the child has taken food last? | 1 hour …………………1  1 to 2 hours ……………2  More than 2 hours …….3 |  |
|  | Record the time of the blood glucose test | \|  \| Hours \| \| \|  \| Minutes \| \| \| --- \| --- \| --- \| --- \| --- \| --- \| --- \| \|  \| \|  \|  \| : \|  \|  \|   Not tested …………………….996 |  |
|  | Record blood glucose in MG/DL | \| mg/dl: \|  \|  \|  \| \| --- \| --- \| --- \| --- \|   Refused …………………………….995  Other…………………………………996  Not tested…………………………997 |  |
|  | Record haemoglobin level | \| g/dl: \|  \|  \|  \| . \|  \| \| --- \| --- \| --- \| --- \| --- \| --- \|   Refused …………………………….995  Other…………………………………996  Not tested…………………………997 |  |
|  | Record the SCD/SCT percentage (%) here | \| Haemoglobin A2/C/E \| % \| \| --- \| --- \| \| Haemoglobin S \| % \| \| Haemoglobin F \| % \| \| Haemoglobin A \| % \|   Refused …………………………….995  Other…………………………………996  Not tested…………………………997 |  |
|  | **Section 6: Rating of health** | |  |
| 601. | We would like to know how good or bad your health is TODAY.  This scale is numbered from 0 to 100  100 means the best health you can imagine. 0 means the worst health you can imagine.  Mark an X on the scale to indicate how your health is TODAY  Now, please write the number you marked on the scale in the box below. | Your Health Today = |  |

*******************************End************************************************

**Age 10 – 19 Years Old Female Questionnaire**

| **IDENTIFICATION** | |
| --- | --- |
| DISTRICT NAME:  BLOCK NAME:  CLUSTER NAME:  TYPE OF PSU (URBAN=1, RURAL=2):  HOUSEHOLD NUMBER:  INDIVIDUAL ID:  INTERVIEW DATE:  INTERVIEW START TIME:  NAME OF THE INVESTIGATOR:  CODE OF THE INVESTIGATOR:  SIGNATURE OF THE INVESTIGATOR:  INTERVIEW RESULTS: 1) COMPLETED  2) NOT AT HOME  3) POSTPONED  4) REFUSED  5) PARTLY COMPLETED  6) INCAPACITATED  7) OTHERS  (SPECIFY __________________) | DISTRICT CODE:  BLOCK CODE:  CLUSTER CODE:  HOUSEHOLD ID:  INTERVIEW END TIME: |

| **Section 1:  Respondent's Background Characteristics** | | | | |
| --- | --- | --- | --- | --- |
| **No.** | **Questions and Filters** | | **Coding Categories** | **Skip** |
|  | What is your name? | |  |  |
|  | Age | | Date of birth:  Age___________ in years |  |
|  | Sex | | Male………………1  Female…………….2  Transgender……….3 |  |
|  | Are you currently going to school/collage? | | Yes ……………1  No …………...2 | If ‘no. then skip to q107 |
|  | In which grade are you studying? | |  |  |
|  | Are you attending government or private school? | | Govt. School………..1  Private School………2 |  |
|  | Have you ever attended school? | | Yes ……………1  No …………...2 | If no, skip que.108 |
|  | What is the highest grade you completed? | | Grade completed: ________ Years |  |
|  | What is your religion? | | Hindu…………………………….1  Muslim…………………………..2  Christian…………………………3  No religion ………………………4  Other……………………………..9  (Specify ___________________________) |  |
|  | Ethnic group | | Listed ST (Scheduled Tribe)……………………1  Primitive Vulnerable Tribal Groups (PVTGs) …2 |  |
|  | What is your tribe/ PVTG? | | **ST**  Bagata ……………………………..1  Baiga ………………………………..2  Banjara …………………………….3  Bathudi …………………………….4  Bhottada ………………………….5  Bhuiya ………………………………6  Bhumia ……………………………..7  Bhumij ……………………………...8  Bhunjia ………………………………9  Binjhal ……………………………….10  Binjhia ……………………………….11  Birhor ………………………………..12  BondoPoraja ……………………..13  Chenchu …………………………….14  Dal ……………………………………..15  Desia Bhumij ………………………16  Dharua ……………………………….17  Didayi ………………………………….18  Gadaba ………………………………..19  Gandia …………………………………20  Ghara …………………………………..21  Gond ……………………………………22  Ho ………………………………………..23  Holva ……………………………………24  Jatapu ………………………………….25  Juang ……………………………………26  Kandha Gauda ……………………...27  Kawar ……………………………………28  Kharia ……………………………………29  Kharwar ………………………………..30  Khond ……………………………………31  Kisan ………………………………………32  Kol …………………………………………33  Kolah Loharas …………………….34  Kolha ………………………………….35  Koli ……………………………………..36  Kondadora ………………………….37  Kora …………………………………….38  Korua …………………………………..39  Kotia …………………………………….40  Koya ……………………………………..41  Kulis ………………………………………42  Lodha …………………………………….43  Madia ……………………………………44  Mahali …………………………………..45  Mankidi …………………………………46  Mankirdia ………………………………47  Matya ……………………………………48  Mirdhas …………………………………49  Munda …………………………………..50  Mundari …………………………………51  Omanatya ………………………………52  Oraon …………………………………….53  Parenga …………………………………54  Paroja ……………………………………55  Pentia …………………………………….56  Rajuar ……………………………………57  Santal …………………………………….58  Saora ……………………………………..59  Shabar……………………………………60  Sounti ……………………………………61  Tharua …………………………………..62  **PVTGs**  Birhor …………………………………….63  Bondo ……………………………………64  Chuktia Bhunjia………………………65  Didayi …………………………………….66  Juang ……………………………………..67  Kharia …………………………………….68  Dongria Khond ………………………69  Kutia Khond …………………………..70  Lanjia Saora ……………………………71  Lodha …………………………………….72  Mankidia ……………………………….73  Paudi Bhuyan …………………………74  Saora ……………………………………..75 |  |
|  | What is your occupation, that is, what kind of work do you mainly do? | | Professional (technical/ administrative/  Managerial, etc.) ……………………1  Clerical ………………………………2  Sales worker …………………………3  Service worker ……………………….4  Production worker (skilled& unskilled) 5  Agricultural …………………………6  Horticulture …………………………7  Wage Earning ………………………8  Shifting cultivation …………………9  Forest collection …………………….10  Food gathering ………………………11  Small business ………………………12  Fishing ………………………………13  Going to school/studying ……………14  Looking for work ……………………..15  Retired ………………………………...16  Unable to work/ill/handicapped ………17  Housework/childcare …………………18  Others …………………………………19  (Specify_______________________) |  |
|  | Do you usually work throughout the year, or do you work seasonally, or only once in a while? | | Throughout the Year ………….1  Seasonally/Part of The Year…..2  Once in A While ………………3 |  |
|  | Are you paid in cash or kind for this, or you are not paid at all? | | Cash only………………1  Cash and kind………….2  In kind only……………3  Not paid……………….4 |  |
|  | What kind of toilet do you use? | | Own toilet ……………………………1  Community toilet …………………….2  Shared toilet with other household……3  No facility/uses open space or field ….4 |  |
|  | Do you use any of these to wash your hands? (Hand hygiene) | | \|  \| After toilet \| Before food \| \| --- \| --- \| --- \| \| Soap/detergent \|  \|  \| \| Ash/mud \|  \|  \| \| Nothing \|  \|  \| \| Others \|  \|  \| |  |
|  | Are you covered by any health scheme or health insurance? | | Yes ……………1  No …………...2 | If no, skip que.118 |
|  | What type of health scheme or health insurance? | | \|  \| Yes \| No \| \| --- \| --- \| --- \| \| Employees State Insurance Scheme (ESIS) \| 1 \| 2 \| \| Central Govt. Health Schemes (CGHS) \| 1 \| 2 \| \| State Health Insurance Scheme \| 1 \| 2 \| \| Rashtriya Swasthya Bima Yojana (RSBY) \| 1 \| 2 \| \| Community Health Insurance Programme \| 1 \| 2 \| \| Other Health Insurance Through Employer \| 1 \| 2 \| \| Other Privately Purchased Commercial Health Insurance \| 1 \| 2 \| \| Others  (Specify__________) \| 1 \| 2 \| |  |
| **Section 2: IFA Supplementation and deworming medications** | | | | |
|  | Do you receive Iron and Folic Acid (IFA) supplement / tablets? | | Yes……………………………..1  No………………………………2  Don’t know…………………9 | If ‘yes  go to 202 |
|  | \| Source of getting  Anganawadi ..1  School …………2  ASHA……………3  Others ………..4  (Specify____) \| Frequency of receiving IFA?  Daily……………………………1  More than once in a week2  Weekly……………………………3  Monthly………………………….4  Quarterly……………………..…5  Half Yearly………………………6  Yearly…………………………….7  Don’t know…………………..9 \| Do you consume IFA supplements?  Yes…………1  No…………….2 \| Do you consume IFA supplement as per recommendation?  Yes ………………...1  No …………........2  Don’t know …..3 \| \| --- \| --- \| --- \| --- \| \|  \|  \|  \|  \| | | |  |
|  | Do you receive deworming tablets / syrup? | | Yes………………………………1  No……………………………….2 | If ‘no’ then go to 301 |
|  | \| Source of getting  Anganawadi ….1  School ……………2  ASHA………………3  Others …………..4  (Specify______) \| Frequency of receiving?  Daily………………………………….1  More than once in a week…2  Weekly……………………………….3  Monthly……………………………..4  Quarterly……………………………5  Half Yearly………………………...6  Yearly……………………………..…7  Don’t know………………………..9 \| Do you consume?  Yes…………1  No………….2 \| Do you consume nt as per recommendation?  Yes ………………....1  No …………..........2  Don’t know …….3 \| \| --- \| --- \| --- \| --- \| \|  \|  \|  \|  \| | | |  |
| **Section 3: Mid-day Meal Programme & Absenteeism**  (check q.104, if answer is ‘yes’ ask this section 4, otherwise skip) | | | | |
|  | In school do you receive mid-day meal (MDM)? | | Yes…………………………..1  No……………………………2 | If ‘no’ then go to 305 |
|  | How often do you eat the mid-day meal (MDM) in school in a week? | | ___Days |  |
|  | Do you like the mid-day meal that is given in school? | | Yes…………………………..1  No……………………………2 | If ‘yes’, then skip 304 |
|  | Why do you not like the mid-day meal daily that is given in school? | | Not tasty…………………...1  Do not like the menu….2  Less quantity………………3  Dirty (the way it is served)…..4  Others…………………………..5  (Specify______________) |  |
|  | Did you discontinue school in the last 15 days? | | Yes…………………………..1  No……………………………2 |  |
|  | Did you discontinue school while you were sick in the last 15 days? | | Yes…………………………..1  No……………………………2 |  |
|  | How many days were you absent due to sickness in the last 15 working school days? | | ____days |  |
| **Section 4: Smoking & Alcohol Consumption** | | | | |
|  | Do you currently smoke or use tobacco in any form? | | Yes ………………..1  No …………………2  Don’t want to say ….8 | If ‘no’, skip to 408 |
|  | In what other form do you currently smoke or use tobacco? Any other form?   \| Tobacco form \| Frequency \| How often do you use tobacco?  Almost every day……1  Once a week………...2  less than once a week.3  Don’t want to say …..8  (Fill this when the frequency of smoking is more than 0) \| How long have you been smoking   \| W \| W \| M \| M \| Y \| Y \| \| --- \| --- \| --- \| --- \| --- \| --- \|   (Fill this when the frequency of smoking is more than 0) \| \| --- \| --- \| --- \| --- \| --- \| --- \| --- \| --- \| --- \| --- \| \| Cigar \|  \|  \|  \| \| A pipe \|  \|  \|  \| \| Hookah \|  \|  \|  \| \| Gutka / paan masala \|  \|  \|  \| \| Tobacco \|  \|  \|  \| \| Khaini \|  \|  \|  \| \| Paan with tobacco \|  \|  \|  \| \| Other chewing tobacco \|  \|  \|  \| \| Snuff \|  \|  \|  \| \| Other  (Specify___) \|  \|  \|  \| | | | |
|  | During the last 12 months, have you ever tried to stop smoking or using tobacco in any other form? | | Yes ……………1  No …………...2 |  |
|  | In last 12 months, have you visited a doctor/ other health care provider? | | Yes ……………1  No …………...2 |  |
|  | During these visits, were you advised to quit smoking or using tobacco in any other form? | | Yes ……………1  No …………...2 |  |
|  | Do you drink alcohol? | | Yes ………………..1  No …………………2  Don’t want to say ….8 | If ‘yes’, then ask 409-412 |
|  | \|  \| Frequency \| How often do you use tobacco?  Almost every day……1  Once a week………...2  less than once a week.3  Don’t want to say …..8  (Fill this when the frequency of alcohol is more than 0) \| How long have you been smoking   \| W \| W \| M \| M \| Y \| Y \| \| --- \| --- \| --- \| --- \| --- \| --- \|   (Fill this when the frequency of alcohol is more than 0) \| \| --- \| --- \| --- \| --- \| --- \| --- \| --- \| --- \| --- \| --- \| \| Tadi madi \|  \|  \|  \| \| Country Liquor \|  \|  \|  \| \| Beer \|  \|  \|  \| \| Wine \|  \|  \|  \| \| Hard Liquor \|  \|  \|  \| \| Other  (Specify___) \|  \|  \|  \| | | |  |
|  | During the last 12 months, have you ever tried to stop alcohol? | | Yes ……………1  No …………...2 |  |
|  | Have you ever done any of the following?   \|  \| Yes \| No \| Where did you get?  Home ……1  Shop …….2  Other …… 4  (Specify ________) \| \| --- \| --- \| --- \| --- \| \| Tattooing \|  \|  \|  \| \| Piercing \|  \|  \|  \| | | |  |
| **Section 5: Marriage, Reproduction & Family Planning**  (Check the age of participant, if age is 12 years or more then ask Section 5,6) | | | | |
|  | What is your current marital status? | | Never married ……………………1  Currently married ………………...2  Widower …………………………3  Divorced …………………………4  Separated …………………………5  Live-In ……………………………6  Don’t want to say…………………8 | If response is “never married” skip the section |
|  | Have you been married once or more than once? | | Only Once……………….1  More than once………….2 |  |
|  | Whether you are biologically related to your husband before marriage? | | Yes ……………1  No …………...2 |  |
|  | If yes, specify the marriage with | | First cousin (paternal and maternal) ……1  Second cousin ………………………….2  Uncle–niece ……………………………3  Others …………………………………4 |  |
|  | How old are you at the time of marriage? | | __________________completed in years |  |
|  | In what month and year did you get married? | |  |  |
|  | How many times have you conceived till now? | | No. of Pregnancies: |  |
|  | How many months after marriage did you conceive for the first time? | | Months:__ |  |
|  | Number of live births | | Number: |  |
|  | Number of still births | | Number: |  |
|  | Number of abortions | | Number: |  |
|  | Number of living children | | Son:  Daughter:  Total: |  |
|  | Number of children died | | Son:  Daughter:  Total: |  |
|  | Number of births in the last five years | | Number:__ |  |
|  | When was the last child born? | | \| DD \| MM \| YY \| \| --- \| --- \| --- \| \|  \|  \|  \| |  |
|  | Are you pregnant now? | | Yes…………….1  No……………...2  Unsure…………8 | If ‘yes’ ask 517 |
|  | How many months pregnant are you? | | Months:_________ |  |
|  | Where do you planned to deliver your baby? | | Husband/In-laws place ……………1  Maternal place …………………….2  Other . ……………………………3  (Specify _____________________) |  |
|  | Have you ever heard of anything to delay or avoid getting pregnant? | | Yes……………………1  No…………………….2 | If ‘Yes’ ask 521 |
|  | Knowledge & Practice of methods of family planning that couple can use to avoid pregnancy?  (Check list – multiple options), if knowledge is checked then ask about practice.  (Show the prepared chart for better understanding)   \|  \| Knowledge \| Practice \| \| --- \| --- \| --- \| \| Female sterilization \|  \|  \| \| Male sterilization \|  \|  \| \| IUD/PPIUD \|  \|  \| \| Injectables \|  \|  \| \| Pill \|  \|  \| \| Condom/nirodh \|  \|  \| \| Female condom \|  \|  \| \| Emergency contraception \|  \|  \| \| Diaphragm \|  \|  \| \| Foam/jelly \|  \|  \| \| Standard days method \|  \|  \| \| Lactational Amenorrhoea Method \|  \|  \| \| Rhythm method \|  \|  \| \| Withdrawal \|  \|  \| \| Other modern method \|  \|  \| \| Other traditional method \|  \|  \| | | |  |
|  | Were you ever told by a health worker about any methods of family planning that you can use to avoid pregnancy? | | Yes…………………..1  No……………………2 |  |
|  | You started using (CURRENT METHOD) in (MONTH/YEAR). At that time, were you told about side effects or problems you might have with the method? | | Yes…………………..1  No……………………2 |  |
|  | When you got pregnant, did you want to get pregnant at that time? | | Yes…………………..1  No……………………2 | If ‘yes’, then ask 524 |
|  | Did you want to have the baby later on or did you not want any more children? | | Later……………………1  No more/none…………...2 |  |
| **Section 6: Knowledge of HIV/AIDS among Adults** | | | | |
|  | Now I would like to talk about something else. Have you ever heard of an illness called AIDS/HIV? | | Yes…………………..1  No……………………2 |  |
|  | Knowledge regarding HIV/AIDS   \| Transmission knowledge \| Prevention knowledge \| Treatment knowledge \| \| --- \| --- \| --- \| \|  \|  \|  \|   Adequate knowledge ………………..1  Inadequate knowledge ………………2 | | |  |
|  | Can people reduce their chances of getting HIV by using a condom every time they have sex? | | Yes……………………1  No…………………….2  Don’t know…………...8 |  |
| **Section 7: Women Empowerment, Menstrual Hygiene & Gender based Violence** | | | | |
|  | Who usually makes decisions about health care for yourself: mainly you, mainly your husband, you and your husband jointly, or someone else? | | Respondent………………….1  Husband…………………….2  Respondent and husband jointly………………………..3  Someone else………………..4  Other………………………...5 |  |
|  | Who usually makes decisions about the following?   \| Health care of yourself \| Major household purchase \| Visit to your family or relatives \| \| --- \| --- \| --- \| \|  \|  \|  \|   Respondent……………………………………………..1  Husband………………………………………………...2  Respondent and husband jointly………………………..3  Someone else……………………………………………4  Other……………………….............................................5 | | |  |
|  | Do you own this or any other house/ any agricultural or non-agricultural land either alone or jointly with someone else? | | Alone only……………1  Jointly only……………2  Both alone and jointly…3  Does not own………….4 |  |
|  | Do you have a bank or savings account that you yourself use? | | Yes……………………….1  No………………………..2 | If ‘no’ then skip 705 |
|  | Do you have any mobile phone that you yourself use? | | Yes……………………….1  No………………………..2 | If ‘yes’ ask 706 |
|  | Do you use mobile phones for any financial transactions? | | Yes ……………1  No …………...2 |  |
|  | Have you ever used the internet? | | Yes ……………1  No …………...2 |  |
|  | Are you covered by any health scheme or health insurance? | | Yes ……………1  No …………...2 | If no, skip que. 709 |
|  | What type of health scheme or health insurance? | | \|  \| Yes \| No \| \| --- \| --- \| --- \| \| Employees State Insurance Scheme (ESIS) \| 1 \| 2 \| \| Central Govt. Health Schemes (CGHS) \| 1 \| 2 \| \| Biju Swasthya Kalyan Yojana (BSKY) \| 1 \| 2 \| \| Biju Krushak Kalyan Yojana (BKKY) \| 1 \| 2 \| \| Odisha State Treatment Fund (OSTF) \| 1 \| 2 \| \| Rashtriya Swasthya Bima Yojana (RSBY) \| 1 \| 2 \| \| Community Health Insurance Programme \| 1 \| 2 \| \| Other Health Insurance Through Employer \| 1 \| 2 \| \| Other Privately Purchased Commercial \| 1 \| 2 \| \| Health Insurance \| 1 \| 2 \| |  |
| **Menstrual Hygiene** | | | | |
|  | Have you ever had your monthly period? | | Yes…………….1  No……………...2 |  |
|  | How old were you when you had your first monthly period? | |  |  |
|  | What was the most common absorbent material used during last menstrual period? | | \|  \| Yes \| No \| \| --- \| --- \| --- \| \| Cloth \| 1 \| 2 \| \| Locally prepared napkins \| 1 \| 2 \| \| Sanitary napkins \| 1 \| 2 \| \| Tampons \| 1 \| 2 \| \| Menstrual cup \| 1 \| 2 \| \| Nothing \| 1 \| 2 \| \| Other  (Specify________) \| 1 \| 2 \| |  |
|  | How do you wash your sanity cloths? | | With water …………………………….1  With water & soap or detergent ………2  With water & mud/ash ………………..3  Other ………………………………….4 |  |
|  | What do you do with the absorbent material | | Dispose it …………….1  Reuse it ……………….2 |  |
|  | During menstruation is there any restriction for following places? | | \|  \| Yes \| No \| \| --- \| --- \| --- \| \| Religious place \| 1 \| 2 \| \| Shrine \| 1 \| 2 \| \| School \| 1 \| 2 \| \| Kitchen \| 1 \| 2 \| \| Bathroom \| 1 \| 2 \| \| River \| 1 \| 2 \| \| Pond \| 1 \| 2 \| \| Living room \| 1 \| 2 \| |  |
|  | During menstruation following activities are restricted | | \|  \| Yes \| No \| \| --- \| --- \| --- \| \| Cooking \| 1 \| 2 \| \| Bathing \| 1 \| 2 \| \| Studying \| 1 \| 2 \| \| Routine household activities \| 1 \| 2 \| \| Playing \| 1 \| 2 \| |  |
|  | During menstrual period, where do you stay | | Inside the home ………….1  Outside the house but in the village ……….2  Outside the house but in the village in a room or hut……………………………………3  Outskirt of the village in open …………4  Outskirt of the village in a room or hut …….5 |  |
| **Gender Based Violence** | | | | |
|  | Have you ever encountered violence in any form? | | Yes……………………….1  No………………………..2 |  |
|  | By whom | | Husband ………………………1  Ex-husband ……………………2  Partner …………………………3  Brother …………………………4  Father …………………………..5  Father in law ……………………6  Brother in law …………………..7  Other relatives …………………..8  Neighbours ………………………9  Friends/colleges/teachers ………10  Others……………………………11  (Specify_____________________) |  |
|  | What type of violence did you experienced? | | Physical …………..1  Sexual …………….2  Both ………………3 |  |
|  | Check 207, If conceived then ask  During pregnancy time have experienced any type of violence? | | Yes……………………….1  No………………………..2 |  |
| **Section 8: Maternal and Child Health (Mothers - for last birth in the 5 years before the survey, if the child is more than 5 years then skip section 8)** | | | | |
| Check 211 if number of births is one or more & ask section 7 | | | | |
|  | Was your last pregnancy got registered? | | Yes……………………….1  No………………………..2 |  |
|  | How many months pregnant were you when you registered? | | Months:___  Don’t know…………98 |  |
|  | With whom did you register? | | ANM…………………..1  ASHA…………………..2  AWW………………….3  Other………………….4 |  |
|  | Did you receive a Mother and Child Protection Card after registration? | | Yes……………………….1  No………………………..2 |  |
|  | How many months pregnant were you when you first received antenatal care for this pregnancy? | | Months:___  Don’t know……………….98 |  |
|  | How many times did you receive antenatal care during this pregnancy? | | Num of times:__  Don’t know……………….98 |  |
|  | During this pregnancy, were you given an injection in the arm to prevent the baby from getting tetanus, that is, convulsions after birth? | | Yes…………………………..1  No……………………………2  Don’t know……………..8 |  |
|  | During this pregnancy, how many times did you get a tetanus injection? | | Times:__  Don’t know……………….8 |  |
|  | At any time before this pregnancy, did you receive any tetanus injections? | | Yes……………………………1  No……………………………2  Don’t know………………8 |  |
|  | Before this pregnancy, how many times did you receive a tetanus injection? | | Times:__  Don’t know…………….8 |  |
|  | How many years ago did you receive the last tetanus injection before this pregnancy? | | Years ago:__ |  |
|  | During this pregnancy, were you given or did you buy any iron folic acid tablets or syrup? | | Yes……………………………1  No…………………………….2  Don’t know………………8 |  |
|  | During the whole pregnancy, for how many days did you take the tablets or syrup? | | Num of days:__  Don’t know……………………….998 |  |
|  | Where did you give birth to (NAME)? | | Pub. Health sector………….1  Pvt. Health sector…………..2  Home……………………………..3  Other……………………………..4  (Specify._____________) |  |
|  | In which place the child was born? | | Husband/In-laws place ……………1  Maternal place …………………….2  Other . ……………………………3  (Specify _____________________) |  |
|  | Was (NAME) delivered by caesarean section, that is, did they cut your belly open to take the baby out? | | Yes……………………….1  No………………………..2 | If ‘yes’ ask 816 |
|  | When was the decision made for you to have a C-section? Was it before the onset of labour or after the onset of labour? | | Before onset of labour……………1  After onset of labour………………2  Don't know…………………………….3 |  |
|  | Who conducted/ assisted with the delivery of (NAME)? | | \|  \| YES \| NO \| \| --- \| --- \| --- \| \| **Health personnel** \|  \|  \| \| Doctor \| 1 \| 2 \| \| Anm/nurse/ midwife/lhv \| 1 \| 2 \| \| Other health professional \| 1 \| 2 \| \| **Other Person** \|  \|  \| \| Dai (tba) \| 1 \| 2 \| \| Friend/relative \| 1 \| 2 \| \| No one \| 1 \| 2 \| \| Others  (specify___________) \| 1 \| 2 \| |  |
|  | Did anyone check on your health while you were still in the facility? | | Yes……………………….1  No………………………..2 | If ‘no’ then skip to 821 |
|  | How long after delivery did the first check take place? | | Hours:__  Days:__  Weeks:__  Don’t know ………………..98 |  |
|  | Who checked on your health at that time? | | \|  \| YES \| NO \| \| --- \| --- \| --- \| \| **Health personnel** \|  \|  \| \| Doctor \| 1 \| 2 \| \| Anm/nurse/ midwife/lhv \| 1 \| 2 \| \| Other health professional \| 1 \| 2 \| \| **Other Person** \|  \|  \| \| Dai (tba) \| 1 \| 2 \| \| Friend/relative \| 1 \| 2 \| \| No one \| 1 \| 2 \| \| Others  (specify___________) \| 1 \| 2 \| |  |
|  | How much in total did it cost you out of your pocket for this delivery? | | Cost Rs._____  Don’t know…………99998 |  |
|  | Check 814, if ‘home’ then ask 822 & 823 | | |  |
|  | After your baby born, have you taken the baby to any health facility for check-up with in 24 hours after birth? | | Yes ……………1  No …………….2 |  |
|  | Who checked your child’s health at that time? | | \|  \| YES \| NO \| \| --- \| --- \| --- \| \| **Health personnel** \|  \|  \| \| Doctor \| 1 \| 2 \| \| Anm/nurse/ midwife/lhv \| 1 \| 2 \| \| Other health professional \| 1 \| 2 \| \| **Other Person** \|  \|  \| \| Dai (tba) \| 1 \| 2 \| \| Friend/relative \| 1 \| 2 \| \| No one \| 1 \| 2 \| \| Others  (specify___________) \| 1 \| 2 \| |  |
| **Section 9- Chronic Disease Conditions** | | | | |
|  | \| Have you ever diagnosed with any of the following disease condition by any health care provider? \| \| \| \| If YES, Have you sought treatment for this \| \| \| --- \| --- \| --- \| --- \| --- \| --- \| \|  \| Yes \| No \| Don’t Know \| Yes \| No \| \| 1.Diabetes \| 1 \| 2 \| 8 \| 1 \| 2 \| \| 2. Hypertension \| 1 \| 2 \| 8 \| 1 \| 2 \| \| 3. Arthritis \| 1 \| 2 \| 8 \| 1 \| 2 \| \| 4. Acid Peptic Disease \| 1 \| 2 \| 8 \| 1 \| 2 \| \| 5. Asthma \| 1 \| 2 \| 8 \| 1 \| 2 \| \| 6. heart diseases \| 1 \| 2 \| 8 \| 1 \| 2 \| \| 7. Stroke \| 1 \| 2 \| 8 \| 1 \| 2 \| \| 8. chronic kidney diseases \| 1 \| 2 \| 8 \| 1 \| 2 \| \| 9. Chronic Liver Disease(alcoholic) \| 1 \| 2 \| 8 \| 1 \| 2 \| \| 10.Chronic Back Ache \| 1 \| 2 \| 8 \| 1 \| 2 \| \| 11. Tuberculosis \| 1 \| 2 \| 8 \| 1 \| 2 \| \| 12. Filariasis \| 1 \| 2 \| 8 \| 1 \| 2 \| \| 13. Visual difficulty \| 1 \| 2 \| 8 \| 1 \| 2 \| \| 14. Deafness \| 1 \| 2 \| 8 \| 1 \| 2 \| \| 15. Cancer \| 1 \| 2 \| 8 \| 1 \| 2 \| \| 16. Dementia \| 1 \| 2 \| 8 \| 1 \| 2 \| \| 17. Epilepsy \| 1 \| 2 \| 8 \| 1 \| 2 \| \| 18. Thyroid disease \| 1 \| 2 \| 8 \| 1 \| 2 \| \| 19. Cancer \| 1 \| 2 \| 8 \| 1 \| 2 \| \| 20. Sickle cell disorder \| 1 \| 2 \| 8 \| 1 \| 2 \| \| 21.Confusion \| 1 \| 2 \| 8 \| 1 \| 2 \| \| 22. Anxiety \| 1 \| 2 \| 8 \| 1 \| 2 \| \| 23. Disturbed sleep \| 1 \| 2 \| 8 \| 1 \| 2 \| \| 24. Frequent infection \| 1 \| 2 \| 8 \| 1 \| 2 \| \| 25. Weakness \| 1 \| 2 \| 8 \| 1 \| 2 \| \| 26. Joint pain \| 1 \| 2 \| 8 \| 1 \| 2 \| \| 27. Hands pain \| 1 \| 2 \| 8 \| 1 \| 2 \| \| 28. Feet pain \| 1 \| 2 \| 8 \| 1 \| 2 \| \| 29. Muscle pain \| 1 \| 2 \| 8 \| 1 \| 2 \| \| Any other chronic disease  (Specify_____________________) \| 1 \| 2 \| 8 \| 1 \| 2 \| | | |  |
|  | If 801 is ‘Cancer’, Specify name of the cancer | | Oral Cancer ………………..1  Breast Cancer ………………2  Cervical Cancer ……………3  Others ………………………4  (Specify _________________) |  |
|  | If 801 is ‘Sickle cell disease’, then ask the following   \| Specify the condition  Sickle cell disease ………1  Sickle cell trait ………….2  Sickle cell disorder (but don’t know which one) …3 \| when the test was done?  Month:  Year: \| If found “positive” what advice was given by doctor/nurse/ANM  Medical advice …………1  Dietary advice …………2  Both ……………………3 \| \| --- \| --- \| --- \| \|  \|  \|  \| | | |  |
|  | Have you ever taken HBV vaccination? | Yes ………………1  No ………………2 | |  |
|  | Have you ever under gone through blood transfusion process? | Yes ……………1  No …………….2 | |  |
|  | Have you ever under gone through IV process? | Yes ……………1  No …………….2 | |  |
| **Section- 10: Health care seeking behavior** | | | | |
|  | Did you visit any health facility for treatment of any disease or injury during past 12 months? | | Yes……………………….1  No………………………..2 | If response is “no” skip que no. 1002-1005 |
|  | Where did you visit for your treatment? | | Government health facility………..1  Private health facility………………2 |  |
|  | For which condition did you visit the health facility? | | Acute………………………………….1  Chronic……………………………….2  Injury………………………………….3 |  |
|  | Specify the condition | |  |  |
|  | Are you cured of the condition now? | | Cured………………………………..1  Not cured……………………………2 |  |
| **Section 11: Symptomatic Profile** | | | | |
|  | Has your blood pressure ever been checked prior to this survey? | | Yes ……………………………….1  No ………………………………..2 | If ‘yes’ ask 1102 - 1104 |
|  | When did you check your blood pressure last? | | Month:  Year: |  |
|  | Have you been informed by any Doctor/Nurse/ANM that you have high/low BP? | | Yes ……………………………….1  No ………………………………..2 |  |
|  | Are you taking any medicine for BP? | | Yes ……………………………….1  No ………………………………..2 |  |
|  | Have you ever been tested for anaemia? | | Yes ……………………..1  No ………………………2 | If ‘yes’ ask 1106 |
|  | If found “positive” what advice was given by doctor/nurse/ANM? | | Medical advice …………1  Dietary advice …………2  Both ……………………3 |  |
|  | Have you ever been tested for blood sugar | | Yes ……………………………….1  No ………………………………..2 | If ‘yes’ ask 1108- 1110 |
|  | If “yes” when blood sugar was checked last time? | | Month:  Year: |  |
|  | What was the result of blood sugar test? | | Low ……………………………..1  Normal …………………………2  High ……………………………...3 |  |
|  | If the answer is “high” what advice was given by doctor/nurse/ANM? | | Medical advice …………1  Dietary advice …………2  Both ……………………3 |  |
|  | Do you have any of the following symptoms? | |  |  |
|  | Do you have any of the following acute symptoms?  (In the last one month)   \| Fever \| Yes \| No \| \| --- \| --- \| --- \| \| Vomiting \| 1 \| 2 \| \| Nausea \| 1 \| 2 \| \| Pain \|  \|  \| \| 1. Joint \| 1 \| 2 \| \| 1. Hands \| 1 \| 2 \| \| 1. Feet \| 1 \| 2 \| \| 1. Muscle \| 1 \| 2 \| \| Body ache \| 1 \| 2 \| \| Headache \| 1 \| 2 \| \| Stomachache \| 1 \| 2 \| \| Anemia \| 1 \| 2 \| \| Swelling Hands \| 1 \| 2 \| \| Swelling Feet \| 1 \| 2 \| \| Dark Urine \| 1 \| 2 \| \| Yellowing of the eyes and skin \| 1 \| 2 \| \| Jaundice \| 1 \| 2 \| \| Tiredness \| 1 \| 2 \| \| Loss of appetite \| 1 \| 2 \| \| Loss of sleep \| 1 \| 2 \| \| Excessive thirst \| 1 \| 2 \| \| Blood in stool \| 1 \| 2 \| \| Swelling in abdomen \| 1 \| 2 \| \| Abdominal pain \| 1 \| 2 \| \| Diarrhea \| 1 \| 2 \| | | |  |
|  | **Section 12: Biomarker** | | | |
|  | Weight in Kilogram | | \| Kg: \|  \|  \|  \| . \|  \|  \| \| --- \| --- \| --- \| --- \| --- \| --- \| --- \|   Not present………….994  Refused………………..995  Other……………………996 |  |
|  | Height in Centimeters | | \| Cm: \|  \|  \|  \| . \|  \|  \| \| --- \| --- \| --- \| --- \| --- \| --- \| --- \|   Not present………….994  Refused………………..995  Other……………………996 |  |
|  | Waist circumference in centimeters | | \| Cm: \|  \|  \|  \| . \|  \|  \| \| --- \| --- \| --- \| --- \| --- \| --- \| --- \|   Not present………….994  Refused………………..995  Other……………………996 |  |
|  | Hip circumference in centimeters | | \| Cm: \|  \|  \|  \| . \|  \|  \| \| --- \| --- \| --- \| --- \| --- \| --- \| --- \|   Not present………….994  Refused………………..995  Other……………………996 |  |
|  | Isometric hand grip strength in Kilogram | | \| Kg: \|  \|  \|  \| . \|  \|  \| \| --- \| --- \| --- \| --- \| --- \| --- \| --- \|   Not present………….994  Refused………………..995  Other……………………996 |  |
|  | The systolic and diastolic pressure for the BP reading   \|  \| Measurement1 \| Measurement2 \| Average \| \| --- \| --- \| --- \| --- \| \| Systolic \|  \|  \|  \| \| Diastolic \|  \|  \|  \|   Refused ………………………………….994  Technical problems ………………..995  Other………………………………………996 | | |  |
|  | When you have taken food last? | | 1 hour …………………1  1 to 2 hours ……………2  More than 2 hours …….3 |  |
|  | Record blood glucose in MG/DL | | \| mg/dl: \|  \|  \|  \| \| --- \| --- \| --- \| --- \|   Refused …………………………….995  Other…………………………………996  Not tested…………………………997 |  |
|  | Record haemoglobin level | | \| g/dl: \|  \|  \|  \| . \|  \| \| --- \| --- \| --- \| --- \| --- \| --- \|   Refused …………………………….995  Other…………………………………996  Not tested…………………………997 |  |
|  | Record the SCD/SCT percentage (%) here | | \| Haemoglobin A2/C/E \| % \| \| --- \| --- \| \| Haemoglobin S \| % \| \| Haemoglobin F \| % \| \| Haemoglobin A \| % \|   Refused …………………………….995  Other…………………………………996  Not tested…………………………997 |  |
| **Section 14: Rating of health** | | | | |
| 1401. | We would like to know how good or bad your health is TODAY.  This scale is numbered from 0 to 100  100 means the best health you can imagine. 0 means the worst health you can imagine.  Mark an X on the scale to indicate how your health is TODAY  Now, please write the number you marked on the scale in the box below. | | Your Health Today = |  |

**************************************END************************************************

**Age 10 – 19 Years Old Male Questionnaire**

| **IDENTIFICATION** | |
| --- | --- |
| DISTRICT NAME:  BLOCK NAME:  CLUSTER NAME:  TYPE OF PSU (URBAN=1, RURAL=2):  HOUSEHOLD NUMBER:  INDIVIDUAL ID:  INTERVIEW DATE:  INTERVIEW START TIME:  NAME OF THE INVESTIGATOR:  CODE OF THE INVESTIGATOR:  SIGNATURE OF THE INVESTIGATOR:  INTERVIEW RESULTS: 1) COMPLETED  2) NOT AT HOME  3) POSTPONED  4) REFUSED  5) PARTLY COMPLETED  6) INCAPACITATED  7) OTHERS  (SPECIFY __________________) | DISTRICT CODE:  BLOCK CODE:  CLUSTER CODE:  HOUSEHOLD ID:  INTERVIEW END TIME: |

| **Section 1:  Respondent's Background Characteristics** | | | |
| --- | --- | --- | --- |
| **No.** | **Questions and Filters** | **Coding Categories** | **Skip** |
|  | What is your name? |  |  |
|  | Age  (Verify from valid document) | Date of birth:  Age___________ in years |  |
|  | Are you currently going to school/collage? | Yes ……………1  No …………...2 | If ‘no. then skip to q106 |
|  | In which grade are you studying? |  |  |
|  | Are you attending government or private school/collage? | Govt. School………..1  Private School………2 |  |
|  | Have you ever attended school? | Yes ……………1  No …………...2 | If no, skip que.107 |
|  | What is the highest grade you completed? | Grade completed: ________ Years |  |
|  | What is your religion? | Hindu…………………………….1  Muslim…………………………..2  Christian…………………………3  No religion ………………………4  Other……………………………..9  (Specify ___________________________) |  |
|  | Ethnic group | Listed ST (Scheduled Tribe)……………1  Primitive Vulnarable Tribal Groups (PVTGs) ……………………………….2 |  |
|  | What is your tribe/ PVTG? | **ST**  Bagata ……………………………..1  Baiga ………………………………..2  Banjara …………………………….3  Bathudi …………………………….4  Bhottada ………………………….5  Bhuiya ………………………………6  Bhumia ……………………………..7  Bhumij ……………………………...8  Bhunjia ………………………………9  Binjhal ……………………………….10  Binjhia ……………………………….11  Birhor ………………………………..12  BondoPoraja ……………………..13  Chenchu …………………………….14  Dal ……………………………………..15  Desia Bhumij ………………………16  Dharua ……………………………….17  Didayi ………………………………….18  Gadaba ………………………………..19  Gandia …………………………………20  Ghara …………………………………..21  Gond ……………………………………22  Ho ………………………………………..23  Holva ……………………………………24  Jatapu ………………………………….25  Juang ……………………………………26  Kandha Gauda ……………………...27  Kawar ……………………………………28  Kharia ……………………………………29  Kharwar ………………………………..30  Khond ……………………………………31  Kisan ………………………………………32  Kol …………………………………………33  Kolah Loharas …………………….34  Kolha ………………………………….35  Koli ……………………………………..36  Kondadora ………………………….37  Kora …………………………………….38  Korua …………………………………..39  Kotia …………………………………….40  Koya ……………………………………..41  Kulis ………………………………………42  Lodha …………………………………….43  Madia ……………………………………44  Mahali …………………………………..45  Mankidi …………………………………46  Mankirdia ………………………………47  Matya ……………………………………48  Mirdhas …………………………………49  Munda …………………………………..50  Mundari …………………………………51  Omanatya ………………………………52  Oraon …………………………………….53  Parenga …………………………………54  Paroja ……………………………………55  Pentia …………………………………….56  Rajuar ……………………………………57  Santal …………………………………….58  Saora ……………………………………..59  Shabar……………………………………60  Sounti ……………………………………61  Tharua …………………………………..62  **PVTGs**  Birhor …………………………………….63  Bondo ……………………………………64  Chuktia Bhunjia………………………65  Didayi …………………………………….66  Juang ……………………………………..67  Kharia …………………………………….68  Dongria Khond ………………………69  Kutia Khond …………………………..70  Lanjia Saora ……………………………71  Lodha …………………………………….72  Mankidia ……………………………….73  Paudi Bhuyan …………………………74  Saora ……………………………………..75 |  |
|  | What is your occupation, that is, what kind of work do you mainly do? | Professional (technical/ administrative/  Managerial, etc.) …………………1  Clerical ……………………………2  Sales worker ……………………3  Service worker …………………….4  Production worker (skilled& unskilled) ..5  Agricultural ………………………..6  Horticulture ………………………..7  Wage Earning ……………………..8  Shifting cultivation ………………..9  Forest collection …………………10  Food gathering …………………...11  Small business …………………...12  Fishing …………………………...13  Going to school/studying ………..14  Looking for work ………………..15  Retired …………………………..16  Unable to work/ill/handicapped …17  Housework/childcare ……………18  Others ……………………………19  (Specify_______________________) |  |
|  | Do you usually work throughout the year, or do you work seasonally, or only once in a while? | Throughout the Year ………….1  Seasonally/Part of The Year…..2  Once in A While ………………3 |  |
|  | Are you paid in cash or kind for this, or you are not paid at all? | Cash only………………1  Cash and kind………….2  In kind only……………3  Not paid……………….4 |  |
|  | Do you have any mobile phone that you yourself use? | Yes……………………….1  No………………………..2 | If ‘no’ then skip q.115 |
|  | Do you use mobile phones for any financial transactions? | Yes ……………1  No …………...2 |  |
|  | Do you have an account in a bank or other financial institutions that you yourself use? | Yes ……………1  No …………...2 |  |
|  | Have you ever used the internet? | Yes ……………1  No …………...2 |  |
|  | Are you covered by any health scheme or health insurance? | Yes ……………1  No …………...2 | If no, skip que.119 |
|  | What type of health scheme or health insurance? | \|  \| Yes \| No \| \| --- \| --- \| --- \| \| Employees State Insurance Scheme (ESIS) \| 1 \| 2 \| \| Central Govt. Health Schemes (CGHS) \| 1 \| 2 \| \| Biju Swasthya Kalyan Yojana (BSKY) \| 1 \| 2 \| \| Biju Krushak Kalyan Yojana (BKKY) \| 1 \| 2 \| \| Odisha State Treatment Fund (OSTF) \| 1 \| 2 \| \| Rashtriya Swasthya Bima Yojana (RSBY)/ PMSBY \| 1 \| 2 \| \| Community Health Insurance Programme \| 1 \| 2 \| \| Other Health Insurance Through Employer \| 1 \| 2 \| \| Other Privately Purchased Commercial \| 1 \| 2 \| \| Health Insurance \| 1 \| 2 \| |  |
|  | What kind of toilet do you use? | Own toilet ………………………1  Community toilet ……………….2  Shared toilet with other household……3  No facility/uses open space or field ….4 |  |
|  | Do you use any of these to wash your hands? (Hand hygiene) | \|  \| Yes \| No \| \| --- \| --- \| --- \| \| Soap/detergent \| 1 \| 2 \| \| Ash/mud \| 1 \| 2 \| \| Nothing \| 1 \| 2 \| \| Others  (Specify__) \| 1 \| 2 \| |  |
| **Section 2: IFA Supplementation and deworming medications** | | | |
|  | Do you receive Iron and Folic Acid (IFA) supplement / tablets? | Yes……………………………..1  No………………………………2  Don’t know…………………9 | If ‘yes’ then ask 202 |
|  | \| Source of getting  Anganawadi ….1  School ……………2  ASHA………………3  Others …………..4  (Specify______) \| Frequency of receiving IFA?  Daily………………………………….1  More than once in a week…2  Weekly……………………………….3  Monthly……………………………..4  Quarterly……………………………5  Half Yearly………………………...6  Yearly……………………………..…7  Don’t know………………………..9 \| Do you consume IFA supplements?  Yes………………1  No………………..2 \| Do you consume supplement as per recommendation?  Yes ………………...1  No …………........2  Don’t know …..3 \| \| --- \| --- \| --- \| --- \| \|  \|  \|  \|  \| | | |
|  | Do you receive deworming tablets / syrup? | Yes………………………………1  No……………………………….2 | If ‘yes’ then ask 204 |
|  | \| Source of getting  Anganawadi ….1  School ……………2  ASHA………………3  Others …………..4  (Specify______) \| Frequency of receiving?  Daily………………………………….1  More than once in a week…2  Weekly……………………………….3  Monthly……………………………..4  Quarterly……………………………5  Half Yearly………………………...6  Yearly……………………………..…7  Don’t know………………………..9 \| Do you consume?  Yes…………1  No………….2 \| Do you consume supplement as per recommendation?  Yes ………………....1  No …………..........2  Don’t know …….3 \| \| --- \| --- \| --- \| --- \| \|  \|  \|  \|  \| | | |
| **Section 3: Mid-day Meal Programme & Absenteeism**  (check q.103, if answer is ‘yes’ ask this section 4, otherwise skip) | | | |
|  | In school do you receive mid-day meal (MDM)? | Yes…………………………..1  No……………………………2 | If ‘no’ then go to 305 |
|  | How often do you eat the mid-day meal (MDM) in school in a week? | ___Days |  |
|  | Do you like the mid-day meal that is given in school? | Yes…………………………..1  No……………………………2 |  |
|  | Why do you not like the mid-day meal daily that is given in school? | Not tasty…………………...1  Do not like the menu….2  Less quantity………………3  Dirty (the way it is served)…..4  Others…………………………..5  (Specify______________) |  |
|  | Did you discontinue school in the last 15 days? | Yes…………………………..1  No……………………………2 |  |
|  | Did you discontinue school while you were sick in the last 15 days? | Yes…………………………..1  No……………………………2 |  |
|  | How many days were you absent due to sickness in the last 15 working school days? | ____days |  |
| **Section 4: Tobacco & Alcohol Consumption** | | | |
|  | Do you currently smoke or use tobacco in any other form? | Yes ………………..1  No …………………2  Don’t want to say ….8 | If ‘no’ or ‘ Don’t want to say’,then skip que. 402 – 405 |
|  | In what other form do you currently smoke or use tobacco? Any other form?   \| Tobacco form \| Frequency \| How often do you use tobacco?  Almost every day……1  Once a week………...2  less than once a week.3  Don’t want to say …..8  (Fill this when the frequency of smoking is more than 0) \| How long have you been smoking   \| W \| W \| M \| M \| Y \| Y \| \| --- \| --- \| --- \| --- \| --- \| --- \|   (Fill this when the frequency of smoking is more than 0) \| \| --- \| --- \| --- \| --- \| --- \| --- \| --- \| --- \| --- \| --- \| \| Cigar \|  \|  \|  \| \| A pipe \|  \|  \|  \| \| Hookah \|  \|  \|  \| \| Gutka / paan masala \|  \|  \|  \| \| Tobacco \|  \|  \|  \| \| Khaini \|  \|  \|  \| \| Paan with tobacco \|  \|  \|  \| \| Other chewing tobacco \|  \|  \|  \| \| Snuff \|  \|  \|  \| \| Other  (Specify___) \|  \|  \|  \| | |  |
|  | During the last 12 months, have you ever tried to stop smoking or using tobacco in any other form? | Yes ……………1  No …………...2 |  |
|  | In last 12 months, have you visited a doctor/ other health care provider? | Yes ……………1  No …………...2 |  |
|  | During these visits, were you advised to quit smoking or using tobacco in any other form? | Yes ……………1  No …………...2 |  |
|  | Do you drink alcohol? | Yes ………………..1  No …………………2  Don’t want to say ….8 | If ‘no’ or ‘Don’t want to say’ then skip 407 & 408 |
|  | \|  \| Frequency \| How often do you use tobacco?  Almost every day……1  Once a week………...2  less than once a week.3  Don’t want to say …..8  (Fill this when the frequency of alcohol is more than 0) \| How long have you been smoking   \| W \| W \| M \| M \| Y \| Y \| \| --- \| --- \| --- \| --- \| --- \| --- \|   (Fill this when the frequency of alcohol is more than 0) \| \| --- \| --- \| --- \| --- \| --- \| --- \| --- \| --- \| --- \| --- \| \| Tadi madi \|  \|  \|  \| \| Country Liquor \|  \|  \|  \| \| Beer \|  \|  \|  \| \| Wine \|  \|  \|  \| \| Hard Liquor \|  \|  \|  \| \| Other  (Specify___) \|  \|  \|  \| | |  |
|  | During the last 12 months, have you ever tried to stop smoking or using tobacco in any other form? | Yes ……………1  No …………...2 |  |
|  | Have you ever done any of the following?   \|  \| Yes \| No \| Where did you get?  Home ……1  Shop …….2  Other …… 3  (Specify ________) \| \| --- \| --- \| --- \| --- \| \| Tattooing \|  \|  \|  \| \| Piercing \|  \|  \|  \| | |  |
|  | Where do you generally go for shaving/hair cut? | At home ………………….1  Saloon ……………………..2  Village barber …………..3 |  |
|  | Does the razor used for shaving is shared? | Yes …………….1  No………………2 |  |
| **Section 5: Marriage & Family Planning**  (Check the age of participant, if age is 12 years or more then ask Section 5,6) | | | |
|  | What is your current marital status? | Never married ……………………1  Currently married ………………...2  Widower …………………………3  Divorced …………………………4  Separated …………………………5  Live-In ……………………………6  Don’t want to say…………………8 | If response is “never married” skip the section |
|  | Whether you are biologically related to your wife before marriage? | Yes ……………1  No …………...2 |  |
|  | If yes, specify the marriage with | First cousin (paternal and maternal) 1  Second cousin …………………….2  Uncle–niece ………………………3  Others ……………………………4 |  |
|  | How old are you at the time of marriage? | _____________completed years  Date of marriage: mm/yyyy |  |
|  | Have you or your partner/partners ever used any method to delay or avoid pregnancy? | Yes ……………1  No …………...2 | If ‘no’ then skip 206 |
|  | Knowledge & Practice of methods of family planning that couple can use to avoid pregnancy?  (Check list – multiple options), if knowledge is checked then ask about practice.  (Show the prepared chart for better understanding)   \|  \| Knowledge \| Practice \| \| --- \| --- \| --- \| \| Female sterilization \|  \|  \| \| Male sterilization \|  \|  \| \| IUD/PPIUD \|  \|  \| \| Injectables \|  \|  \| \| Pill \|  \|  \| \| Condom/nirodh \|  \|  \| \| Female condom \|  \|  \| \| Emergency contraception \|  \|  \| \| Diaphragm \|  \|  \| \| Foam/jelly \|  \|  \| \| Standard days method \|  \|  \| \| Lactational Amenorrhoea Method \|  \|  \| \| Rhythm method \|  \|  \| \| Withdrawal \|  \|  \| \| Other modern method \|  \|  \| \| Other traditional method \|  \|  \| | |  |
|  | Do you know of a place where you can obtain a method of family planning? | Yes ……………1  No …………...2 |  |
| **Section 6: Knowledge of HIV/AIDS among Adults** | | | |
|  | Now I would like to talk about something else. Have you ever heard of an illness called AIDS/HIV? | Yes…………………..1  No……………………2 | If 601 is ‘no’, skip to section 7 |
|  | Knowledge regarding HIV/AIDS   \| Transmission knowledge \| Prevention knowledge \| Treatment knowledge \| \| --- \| --- \| --- \| \|  \|  \|  \|   Adequate knowledge ………………..1  Inadequate knowledge ………………2 | |  |
|  | Can people reduce their chances of getting HIV by using a condom every time they have sex? | Yes……………………1  No…………………….2  Don’t know…………...8 |  |
| **Section 7: Chronic Disease Conditions** | | | |
|  | \| Have you ever diagnosed with any of the following disease condition by any health care provider? \| \| \| \| If YES, Have you sought treatment for this \| \| \| --- \| --- \| --- \| --- \| --- \| --- \| \|  \| Yes \| No \| Don’t Know \| Yes \| No \| \| 1.Diabetes \| 1 \| 2 \| 8 \| 1 \| 2 \| \| 2. Hypertension \| 1 \| 2 \| 8 \| 1 \| 2 \| \| 3. Arthritis \| 1 \| 2 \| 8 \| 1 \| 2 \| \| 4. Acid Peptic Disease \| 1 \| 2 \| 8 \| 1 \| 2 \| \| 5. Asthma \| 1 \| 2 \| 8 \| 1 \| 2 \| \| 6. heart diseases \| 1 \| 2 \| 8 \| 1 \| 2 \| \| 7. Stroke \| 1 \| 2 \| 8 \| 1 \| 2 \| \| 8. chronic kidney diseases \| 1 \| 2 \| 8 \| 1 \| 2 \| \| 9. Chronic Liver Disease(alcoholic) \| 1 \| 2 \| 8 \| 1 \| 2 \| \| 10.Chronic Back Ache \| 1 \| 2 \| 8 \| 1 \| 2 \| \| 11. Tuberculosis \| 1 \| 2 \| 8 \| 1 \| 2 \| \| 12. Filariasis \| 1 \| 2 \| 8 \| 1 \| 2 \| \| 13. Visual difficulty \| 1 \| 2 \| 8 \| 1 \| 2 \| \| 14. Deafness \| 1 \| 2 \| 8 \| 1 \| 2 \| \| 15. Cancer \| 1 \| 2 \| 8 \| 1 \| 2 \| \| 16. Dementia \| 1 \| 2 \| 8 \| 1 \| 2 \| \| 17. Epilepsy \| 1 \| 2 \| 8 \| 1 \| 2 \| \| 18. Thyroid disease \| 1 \| 2 \| 8 \| 1 \| 2 \| \| 19. Cancer \| 1 \| 2 \| 8 \| 1 \| 2 \| \| 20. Sickle cell disease \| 1 \| 2 \| 8 \| 1 \| 2 \| \| 21.Confusion \| 1 \| 2 \| 8 \| 1 \| 2 \| \| 22. Anxiety \| 1 \| 2 \| 8 \| 1 \| 2 \| \| 23. Disturbed sleep \| 1 \| 2 \| 8 \| 1 \| 2 \| \| 24. Frequent infection \| 1 \| 2 \| 8 \| 1 \| 2 \| \| 25. Weakness \| 1 \| 2 \| 8 \| 1 \| 2 \| \| 26. Joint pain \| 1 \| 2 \| 8 \| 1 \| 2 \| \| 27. Hands pain \| 1 \| 2 \| 8 \| 1 \| 2 \| \| 28. Feet pain \| 1 \| 2 \| 8 \| 1 \| 2 \| \| 29. Muscle pain \| 1 \| 2 \| 8 \| 1 \| 2 \| \| Any other chronic disease  (Specify_____________________) \| 1 \| 2 \| 8 \| 1 \| 2 \| | |  |
|  | If 501 is ‘Cancer’, Specify name of the cancer | Oral Cancer ………………..1  Pre cancers lesion ………………2  prostate cancer ……………3  Others ………………………4  (Specify _________________) |  |
|  | If 501 is ‘Sickle cell disease’, then ask the following   \| Specify the condition  Sickle cell disease ………1  Sickle cell trait ………….2  Sickle cell disorder (but don’t know which one) …3 \| when the test was done?  Month:  Year: \| If found “positive” what advice was given by doctor/nurse/ANM  Medical advice …………1  Dietary advice ………….2  Both …………………….3 \| \| --- \| --- \| --- \| \|  \|  \|  \| | |  |
|  | Have you ever taken HBV vaccination? | Yes ………………1  No ………………2 |  |
|  | Have you ever under gone through blood transfusion process? | Yes ……………1  No …………….2 |  |
|  | Have you ever under gone through IV process? | Yes ……………1  No …………….2 |  |
| **Section 8: Health care seeking behavior** | | | |
|  | Did you visit any health facility for treatment of any disease or injury during past 12 months? | Yes……………………….1  No………………………..2 | If response is “no” skip que no. 802-805 |
|  | Where did you visit for your treatment? | ASHA/AWW ………………1  ANM/SC ……………………2  PHC/ HWC …………………3  CHC/District hospital ………4  Private hospital ……………..5  AYUSH ……………………..6  Other ………………………..7  (Specify ____________________) |  |
|  | For which condition did you visit the health facility? | Acute…………………………….1  Chronic…………………………..2  Injury…………………………….3 |  |
|  | Specify the condition |  |  |
|  | Are you cured of the condition now? | Cured………………………..1  Not cured……………………2 |  |
| **Section 9: Symptomatic Profile** | | | |
|  | Has your blood pressure ever been checked prior to this survey? | Yes ……………………………….1  No ………………………………..2 | If ‘yes’ ask 902-904 |
|  | When did you check your blood pressure last? | Month:  Year: |  |
|  | Have you been informed by any Doctor/Nurse/ANM that you have high/low BP? | Yes ……………………………….1  No ………………………………..2 |  |
|  | Are you taking any medicine for BP? | Yes ……………………………….1  No ………………………………..2 |  |
|  | Have you ever been tested for anemia? | Yes ……………………………….1  No ………………………2 | If ‘yes’ ask 906 |
|  | If found “positive” what advice was given by doctor/nurse/ANM? | Medical advice …………1  Dietary advice …………2  Both ……………………3 |  |
|  | Have you ever been tested for blood sugar | Yes ……………………………….1  No ………………………………..2 | If ‘yes’ ask 908-910 |
|  | If “yes” when blood sugar was checked last time? | Month:  Year: |  |
|  | What was the result of blood sugar test? | Low ……………………………..1  Normal …………………………2  High ……………………………...3 |  |
|  | If the answer is “high” what advice was given by doctor/nurse/ANM? | Medical advice …………1  Dietary advice …………2  Both ……………………3 |  |
|  | Do you have any of the following symptoms? (In last one month)   \| Fever \| Yes \| No \| \| --- \| --- \| --- \| \| Vomiting \| 1 \| 2 \| \| Nausea \| 1 \| 2 \| \| Pain \|  \|  \| \| 1. Joint \| 1 \| 2 \| \| 1. Hands \| 1 \| 2 \| \| 1. Feet \| 1 \| 2 \| \| 1. Muscle \| 1 \| 2 \| \| Body ache \| 1 \| 2 \| \| Headache \| 1 \| 2 \| \| Stomachache \| 1 \| 2 \| \| Anemia \| 1 \| 2 \| \| Swelling Hands \| 1 \| 2 \| \| Swelling Feet \| 1 \| 2 \| \| Dark Urine \| 1 \| 2 \| \| Yellowing of the eyes and skin \| 1 \| 2 \| \| Jaundice \| 1 \| 2 \| \| Tiredness \| 1 \| 2 \| \| Loss of appetite \| 1 \| 2 \| \| Loss of sleep \| 1 \| 2 \| \| Excessive thirst \| 1 \| 2 \| \| Blood in stool \| 1 \| 2 \| \| Swelling in abdomen \| 1 \| 2 \| \| Abdominal pain \| 1 \| 2 \| \| Diarrhea \| 1 \| 2 \| | |  |
| **Section 10: Biomarker** | | | |
|  | Weight in Kilogram | \| Kg: \|  \|  \|  \| . \|  \|  \| \| --- \| --- \| --- \| --- \| --- \| --- \| --- \|   Not present………….994  Refused………………..995  Other……………………996 |  |
|  | Height in Centimeters | \| Cm: \|  \|  \|  \| . \|  \|  \| \| --- \| --- \| --- \| --- \| --- \| --- \| --- \|   Not present………….994  Refused………………..995  Other……………………996 |  |
|  | Waist circumference in centimeters | \| Cm: \|  \|  \|  \| . \|  \|  \| \| --- \| --- \| --- \| --- \| --- \| --- \| --- \|   Not present………….994  Refused………………..995  Other……………………996 |  |
|  | Hip circumference in centimeters | \| Cm: \|  \|  \|  \| . \|  \|  \| \| --- \| --- \| --- \| --- \| --- \| --- \| --- \|   Not present………….994  Refused………………..995  Other…………………996 |  |
|  | Isometric hand grip strength in Kilogram | \| Kg: \|  \|  \|  \| . \|  \|  \| \| --- \| --- \| --- \| --- \| --- \| --- \| --- \|   Not present………….994  Refused………………995  Other…………………996 |  |
|  | The systolic and diastolic pressure for the BP reading   \|  \| Measurement1 \| Measurement2 \| Average \| \| --- \| --- \| --- \| --- \| \| Systolic \|  \|  \|  \| \| Diastolic \|  \|  \|  \|   Refused ……………………………994  Technical problems ………………..995  Other……………………………….996 | |  |
|  | When you have taken food last? | 1 hour …………………1  1 to 2 hours ……………2  More than 2 hours …….3 |  |
|  | Record blood glucose in MG/DL | \| mg/dl: \|  \|  \|  \| \| --- \| --- \| --- \| --- \|   Refused ………………………995  Other………………………….996  Not tested……………………..997 |  |
|  | Record haemoglobin level | \| g/dl: \|  \|  \|  \| . \|  \| \| --- \| --- \| --- \| --- \| --- \| --- \|   Refused ………………………995  Other………………………….996  Not tested……………………..997 |  |
|  | Record the SCD/SCT percentage (%) here | \| Haemoglobin A2/C/E \| % \| \| --- \| --- \| \| Haemoglobin S \| % \| \| Haemoglobin F \| % \| \| Haemoglobin A \| % \|   Refused ………………………995  Other………………………… 996  Not tested…………………… 997 |  |
| **Section 11: Rating of health** | | | |
| 1101. | We would like to know how good or bad your health is TODAY.  This scale is numbered from 0 to 100  100 means the best health you can imagine. 0 means the worst health you can imagine.  Mark an X on the scale to indicate how your health is TODAY  Now, please write the number you marked on the scale in the box below. | Your Health Today = |  |

**************************************END************************************************

**Age 20-59 Years Old Men Questionnaire**

| **IDENTIFICATION** | |
| --- | --- |
| DISTRICT NAME:  BLOCK NAME:  CLUSTER NAME:  TYPE OF PSU (URBAN=1, RURAL=2):  HOUSEHOLD NUMBER:  INDIVIDUAL ID:  INTERVIEW DATE:  INTERVIEW START TIME:  NAME OF THE INVESTIGATOR:  CODE OF THE INVESTIGATOR:  SIGNATURE OF THE INVESTIGATOR:  INTERVIEW RESULTS: 1) COMPLETED  2) NOT AT HOME  3) POSTPONED  4) REFUSED  5) PARTLY COMPLETED  6) INCAPACITATED  7) OTHERS  (SPECIFY __________________) | DISTRICT CODE:  BLOCK CODE:  CLUSTER CODE:  HOUSEHOLD ID:  INTERVIEW END TIME: |

|  | | | |  |
| --- | --- | --- | --- | --- |
| **Section 1:  Respondent's Background Characteristics** | | | | |
| **No.** | **Questions and Filters** | **Coding Categories** | | **Skip** |
|  | What is your name? |  | |  |
|  | Age  (Verify from valid document) | Date of birth:  Age___________ in years | |  |
|  | Have you ever attended school? | Yes ……………1  No …………...2 | | If no, skip que.104 |
|  | What is the highest grade you completed? | Grade completed: ________ Years | |  |
|  | What is your religion? | Hindu…………………………….1  Muslim…………………………..2  Christian…………………………3  No religion ………………………4  Other……………………………..9  (Specify ___________________________) | |  |
|  | Ethnic group | Listed ST (Scheduled Tribe)………………1  Primitive Vulnarable Tribal Groups (PVTGs) ………………………………….2 | |  |
|  | Mention tribe/PVTG name | **ST**  Bagata ………………………………1  Baiga ………………………………..2  Banjara ……………………………...3  Bathudi ……………………………...4  Bhottada …………………………….5  Bhuiya ………………………………6  Bhumia ……………………………...7  Bhumij ……………………………....8  Bhunjia ………………………………9  Binjhal ………………………………10  Binjhia …………………………….11  Birhor ……………………………….12  BondoPoraja ………………………13  Chenchu ……………………………14  Dal …………………………………15  Desia Bhumij ………………………16  Dharua ………………………………17  Didayi ………………………………18  Gadaba …………………………….19  Gandia ………………………………20  Ghara ………………………………21  Gond ……………………………….22  Ho …………………………………23  Holva ………………………………24  Jatapu ………………………………25  Juang ………………………………26  Kandha Gauda ……………………..27  Kawar ………………………………28  Kharia ………………………………29  Kharwar ………………………….30  Khond ……………………………31  Kisan ……………………………….32  Kol …………………………………33  Kolah Loharas ………………………34  Kolha ……………………………….35  Koli …………………………………36  Kondadora ………………………….37  Kora ……………………………….38  Korua ………………………………39  Kotia ……………………………….40  Koya ………………………………..41  Kulis ………………………………42  Lodha ………………………………43  Madia ………………………………44  Mahali ……………………………..45  Mankidi ……………………………46  Mankirdia …………………………47  Matya ………………………………48  Mirdhas ……………………………49  Munda ……………………………..50  Mundari ……………………………51  Omanatya …………………………52  Oraon ……………………………….53  Parenga ……………………………54  Paroja ………………………………55  Pentia ……………………………….56  Rajuar ………………………………57  Santal ……………………………….58  Saora ……………………………….59  Shabar………………………………60  Sounti ………………………………61  Tha…………………………………..62  **PVTGs**  Birhor ……………………………63  Bondo ……………………………64  Chuktia Bhunjia………………….65  Didayi ……………………………66  Juang …………………………….67  Kharia …………………………....68  Dongria Khond …………………..69  Kutia Khond ……………………..70  Lanjia Saora ………...……………71  Lodha …………………………….72  Mankidia ………………………….73  Paudi Bhuyan ……………………74  Saora ……………………………..75 | |  |
|  | What is your occupation, that is, what kind of work do you mainly do? | Professional (technical/ administrative/  Managerial, etc.) ……………………..1  Clerical ……………………………….2  Sales worker ………………………….3  Service worker ……………………..4  Production worker (skilled& unskilled)5  Agricultural …………………………6  Horticulture …………………………7  Wage Earning ………………………8  Shifting cultivation …………………9  Forest collection ……………………10  Food gathering ……………………11  Small business …………………….12  Fishing …………………………….13  Going to school/studying ………….14  Looking for work ………………….15  Retired ……………………………..16  Unable to work/ill/handicapped ……17  Housework/childcare ………………18  Others ……………………………...19  (Specify_______________________) | |  |
|  | Do you usually work throughout the year, or do you work seasonally, or only once in a while? | Throughout the Year ………….1  Seasonally/Part of The Year…..2  Once in A While ………………3 | |  |
|  | Are you paid in cash or kind for this, or you are not paid at all? | Cash only………………1  Cash and kind………….2  In kind only……………3  Not paid……………….4 | |  |
|  | Do you have mobile phone that you use yourself? | Yes ……………1  No …………...2 | | If ‘no’ then skip q.112 |
|  | Do you use mobile phones for any financial transactions? | Yes ……………1  No …………...2 | |  |
|  | Do you have an account in a bank or other financial institutions that you yourself use? | Yes ……………1  No …………...2 | |  |
|  | Have you ever used the internet? | Yes ……………1  No …………...2 | |  |
|  | Are you covered by any health scheme or health insurance? | Yes ……………1  No …………...2 | | If no, skip que.116 |
|  | What type of health scheme or health insurance? | \|  \| Yes \| No \| \| --- \| --- \| --- \| \| Employees State Insurance Scheme (ESIS) \| 1 \| 2 \| \| Central Govt. Health Schemes (CGHS) \| 1 \| 2 \| \| Biju Swasthya Kalyan Yojana (BSKY) \| 1 \| 2 \| \| Biju Krushak Kalyan Yojana (BKKY) \| 1 \| 2 \| \| Odisha State Treatment Fund (OSTF) \| 1 \| 2 \| \| Rashtriya Swasthya Bima Yojana (RSBY)/ PMSBY \| 1 \| 2 \| \| Community Health Insurance Programme \| 1 \| 2 \| \| Other Health Insurance Through Employer \| 1 \| 2 \| \| Other Privately Purchased Commercial Health Insurance \| 1 \| 2 \| | |  |
|  | What kind of toilet do you use? | Own toilet ……………………………1  Community toilet …………………….2  Shared toilet with other household……3  No facility/uses open space or field ….4 | |  |
|  | Hand hygiene practices? | \|  \| After toilet \| Before food \| \| --- \| --- \| --- \| \| Soap/detergent \|  \|  \| \| Ash/mud \|  \|  \| \| Nothing \|  \|  \| \| Others \|  \|  \| | |  |
| **Section 2: Marriage, Family Planning & Reproduction** | | | |  |
|  | What is your current marital status? | Never married ……………………1  Currently married ………………..2  Widower …………………………3  Divorced …………………………4  Separated …………………………5  Live-In ……………………………6  Don’t want to say…………………8 | | If response is “never married” or “Don’t want to say” skip the section |
|  | Whether you are biologically related to your wife? | Yes ……………1  No …………...2 | | If ‘no’ then skip to q.204 |
|  | If yes, specify the marriage with | First cousin (paternal and maternal) …1  Second cousin ………………………2  Uncle–niece …………………………3  Others ………………………………4 | |  |
|  | How old were you when you (first) got married? | _____________completed years  Date of marriage: mm/yyyy | |  |
|  | Have you or your partner/partners ever used any method to delay or avoid pregnancy? | Yes ……………1  No …………...2 | | If ‘no’ then skip 206 |
|  | Knowledge & Practice of methods of family planning that couple can use to avoid pregnancy?  (Check list – multiple options), if knowledge is checked then ask about practice.  (Show the prepared chart for better understanding)   \|  \| Knowledge \| Practice \| \| --- \| --- \| --- \| \| Female sterilization \|  \|  \| \| Male sterilization \|  \|  \| \| IUD/PPIUD \|  \|  \| \| Injectables \|  \|  \| \| Pill \|  \|  \| \| Condom/nirodh \|  \|  \| \| Female condom \|  \|  \| \| Emergency contraception \|  \|  \| \| Diaphragm \|  \|  \| \| Foam/jelly \|  \|  \| \| Standard days method \|  \|  \| \| Lactational Amenorrhoea Method \|  \|  \| \| Rhythm method \|  \|  \| \| Withdrawal \|  \|  \| \| Other modern method \|  \|  \| \| Other traditional method \|  \|  \| | | |  |
|  | How old were you when your first child was born? |  | |  |
| **Section 3: Tobacco & Alcohol Consumption** | | | |  |
|  | Do you currently smoke or use tobacco in any other form? | Yes ………………..1  No …………………2  Don’t want to say ….8 | | If ‘no’ or ‘ Don’t want to say’,then skip que. 302 – 305 |
|  | In what other form do you currently smoke or use tobacco? Any other form?   \| Tobacco form \| Frequency \| How often do you use tobacco?  Almost every day……1  Once a week………...2  less than once a week.3  Don’t want to say …..8  (Fill this when the frequency of smoking is more than 0) \| How long have you been smoking   \| W \| W \| M \| M \| Y \| Y \| \| --- \| --- \| --- \| --- \| --- \| --- \|   (Fill this when the frequency of smoking is more than 0) \| \| --- \| --- \| --- \| --- \| --- \| --- \| --- \| --- \| --- \| --- \| \| Cigar \|  \|  \|  \| \| A pipe \|  \|  \|  \| \| Hookah \|  \|  \|  \| \| Gutka / paan masala \|  \|  \|  \| \| Tobacco \|  \|  \|  \| \| Khaini \|  \|  \|  \| \| Paan with tobacco \|  \|  \|  \| \| Other chewing tobacco \|  \|  \|  \| \| Snuff \|  \|  \|  \| \| Other  (Specify___) \|  \|  \|  \| | | |  |
|  | During the last 12 months, have you ever tried to stop smoking or using tobacco in any other form? | Yes ……………1  No …………...2 | |  |
|  | In last 12 months, have you visited a doctor/ other health care provider? | Yes ……………1  No …………...2 | |  |
|  | During these visits, were you advised to quit smoking or using tobacco in any other form? | Yes ……………1  No …………...2 | |  |
|  | Do you drink alcohol? | Yes ………………..1  No …………………2  Don’t want to say ….8 | | If ‘no’ or ‘Don’t want to say’ goto 313 |
|  | \|  \| Frequency \| How often do you use tobacco?  Almost every day……1  Once a week………...2  less than once a week.3  Don’t want to say …..8  (Fill this when the frequency of alcohol is more than 0) \| How long have you been smoking   \| W \| W \| M \| M \| Y \| Y \| \| --- \| --- \| --- \| --- \| --- \| --- \|   (Fill this when the frequency of alcohol is more than 0) \| \| --- \| --- \| --- \| --- \| --- \| --- \| --- \| --- \| --- \| --- \| \| Tadi madi \|  \|  \|  \| \| Country Liquor \|  \|  \|  \| \| Beer \|  \|  \|  \| \| Wine \|  \|  \|  \| \| Hard Liquor \|  \|  \|  \| \| Other  (Specify___) \|  \|  \|  \| | | |  |
|  | During the last 12 months, have you ever tried to stop alcohol? | Yes ……………1  No …………...2 | |  |
|  | Have you ever done any of the following?   \|  \| Yes \| No \| Where did you get?  Home ……1  Shop …….2  Other …… 3  (Specify ________) \| \| --- \| --- \| --- \| --- \| \| Tattooing \|  \|  \|  \| \| Piercing \|  \|  \|  \| | | |  |
|  | Where do you generally go for shaving/hair cut? | At home ……………………1  Saloon ……………………...2  Village barber ………………3 | |  |
|  | Does the razor used for shaving is shared? | Yes …………….1  No………………2 | |  |
| **Section 4: Knowledge of HIV/AIDS among Adults** | | | |  |
|  | Now I would like to talk about something else. Have you ever heard of an illness called AIDS/HIV? | Yes…………………..1  No……………………2 | | If 401 is ‘no’, skip to section 5 |
|  | Knowledge regarding HIV/AIDS   \| Transmission knowledge \| Prevention knowledge \| Treatment knowledge \| \| --- \| --- \| --- \| \|  \|  \|  \|   Adequate knowledge ………………..1  Inadequate knowledge ………………2 | | |  |
|  | Can people reduce their chances of getting HIV by using a condom every time they have sex? | Yes……………………1  No…………………….2  Don’t know…………...8 | |  |
| **Section 5: Chronic Disease Conditions** | | | |  |
|  | \| Have you ever diagnosed with any of the following disease condition by any health care provider? \| \| \| \| If YES, Have you sought treatment for this \| \| \| --- \| --- \| --- \| --- \| --- \| --- \| \|  \| Yes \| No \| Don’t Know \| Yes \| No \| \| 1.Diabetes \| 1 \| 2 \| 8 \| 1 \| 2 \| \| 2. Hypertension \| 1 \| 2 \| 8 \| 1 \| 2 \| \| 3. Arthritis \| 1 \| 2 \| 8 \| 1 \| 2 \| \| 4. Acid Peptic Disease \| 1 \| 2 \| 8 \| 1 \| 2 \| \| 5. Asthma \| 1 \| 2 \| 8 \| 1 \| 2 \| \| 6. heart diseases \| 1 \| 2 \| 8 \| 1 \| 2 \| \| 7. Stroke \| 1 \| 2 \| 8 \| 1 \| 2 \| \| 8. chronic kidney diseases \| 1 \| 2 \| 8 \| 1 \| 2 \| \| 9. Chronic Liver Disease(alcoholic) \| 1 \| 2 \| 8 \| 1 \| 2 \| \| 10.Chronic Back Ache \| 1 \| 2 \| 8 \| 1 \| 2 \| \| 11. Tuberculosis \| 1 \| 2 \| 8 \| 1 \| 2 \| \| 12. Filariasis \| 1 \| 2 \| 8 \| 1 \| 2 \| \| 13. Visual difficulty \| 1 \| 2 \| 8 \| 1 \| 2 \| \| 14. Deafness \| 1 \| 2 \| 8 \| 1 \| 2 \| \| 15. Cancer \| 1 \| 2 \| 8 \| 1 \| 2 \| \| 16. Dementia \| 1 \| 2 \| 8 \| 1 \| 2 \| \| 17. Epilepsy \| 1 \| 2 \| 8 \| 1 \| 2 \| \| 18. Thyroid disease \| 1 \| 2 \| 8 \| 1 \| 2 \| \| 19. Cancer \| 1 \| 2 \| 8 \| 1 \| 2 \| \| 20. Sickle cell disease \| 1 \| 2 \| 8 \| 1 \| 2 \| \| 21.Confusion \| 1 \| 2 \| 8 \| 1 \| 2 \| \| 22. Anxiety \| 1 \| 2 \| 8 \| 1 \| 2 \| \| 23. Disturbed sleep \| 1 \| 2 \| 8 \| 1 \| 2 \| \| 24. Frequent infection \| 1 \| 2 \| 8 \| 1 \| 2 \| \| 25. Weakness \| 1 \| 2 \| 8 \| 1 \| 2 \| \| 26. Joint pain \| 1 \| 2 \| 8 \| 1 \| 2 \| \| 27. Hands pain \| 1 \| 2 \| 8 \| 1 \| 2 \| \| 28. Feet pain \| 1 \| 2 \| 8 \| 1 \| 2 \| \| 29. Muscle pain \| 1 \| 2 \| 8 \| 1 \| 2 \| \| Any other chronic disease  (Specify_____________________) \| 1 \| 2 \| 8 \| 1 \| 2 \| | | |  |
|  | If 501 is ‘Cancer’, Specify name of the cancer | Oral Cancer ………………..1  Pre cancers lesion ………………2  prostate cancer ……………3  Others ………………………4  (Specify _________________) | |  |
|  | If 501 is ‘Sickle cell disease’, then ask the following   \| Specify the condition  Sickle cell disease ………1  Sickle cell trait ………….2  Sickle cell disorder (but don’t know which one) …3 \| when the test was done?  Month:  Year: \| If found “positive” what advice was given by doctor/nurse/ANM  Medical advice …………1  Dietary advice …………2  Both ……………………3 \| \| --- \| --- \| --- \| \|  \|  \|  \| | | |  |
|  | Have you ever taken HBV vaccination? | | Yes ………………1  No ………………2 |  |
|  | Have you ever under gone through blood transfusion process? | | Yes ……………1  No …………….2 |  |
|  | Have you ever under gone through IV process? | | Yes ……………1  No …………….2 |  |
| **Section 6: Health care seeking behavior** | | | |  |
|  | Did you visit any health facility for treatment of any disease or injury during past 12 months? | Yes……………………….1  No………………………..2 | | If response is “no” skip que no. 602-605 |
|  | Where did you visit for your treatment? | ASHA/AWW ………………1  ANM/SC ……………………2  PHC/ HWC …………………3  CHC/District hospital ………4  Private hospital ……………..5  AYUSH ……………………..6  Other ………………………..7  (Specify ____________________) | |  |
|  | For which condition did you visit the health facility? | Acute………………………………….1  Chronic………………………………..2  Injury………………………………….3 | |  |
|  | Specify the condition |  | |  |
|  | Are you cured of the condition now? | Cured………………………………..1  Not cured……………………………2 | |  |
|  | **Section 8: Symptomatic Profile** | | | |
|  | Has your blood pressure ever been checked prior to this survey? | Yes ……………………………….1  No ………………………………..2 | | If ‘yes’ ask 802 - 804 |
|  | When did you check your blood pressure last? | Month:  Year: | |  |
|  | Have you been informed by any Doctor/Nurse/ANM that you have high/low BP? | Yes ……………………………….1  No ………………………………..2 | |  |
|  | Are you taking any medicine for BP? | Yes ……………………………….1  No ………………………………..2 | |  |
|  | Have you ever been tested for anaemia | Yes ……………………………….1  No ………………………2 | | If ‘yes’ ask 806 |
|  | If found “positive” what advice was given by doctor/nurse/ANM? | Medical advice …………1  Dietary advice …………2  Both ……………………3 | |  |
|  | Have you ever been tested for blood sugar | Yes ……………………………….1  No ………………………………..2 | | If ‘yes’ ask 808 - 810 |
|  | If “yes” when blood sugar was checked last time? | Month:  Year: | |  |
|  | What was the result of blood sugar test? | Low ……………………………..1  Normal …………………………2  High ……………………………...3 | |  |
|  | If the answer is “high” what advice was given by doctor/nurse/ANM? | Medical advice …………1  Dietary advice …………2  Both ……………………3 | |  |
|  | Do you have any of the following symptoms? (In last one month)   \| Fever \| Yes \| No \| \| --- \| --- \| --- \| \| Vomiting \| 1 \| 2 \| \| Nausea \| 1 \| 2 \| \| Pain \|  \|  \| \| 1. Joint \| 1 \| 2 \| \| 1. Hands \| 1 \| 2 \| \| 1. Feet \| 1 \| 2 \| \| 1. Muscle \| 1 \| 2 \| \| Body ache \| 1 \| 2 \| \| Headache \| 1 \| 2 \| \| Stomachache \| 1 \| 2 \| \| Anemia \| 1 \| 2 \| \| Swelling Hands \| 1 \| 2 \| \| Swelling Feet \| 1 \| 2 \| \| Dark Urine \| 1 \| 2 \| \| Yellowing of the eyes and skin \| 1 \| 2 \| \| Jaundice \| 1 \| 2 \| \| Tiredness \| 1 \| 2 \| \| Loss of appetite \| 1 \| 2 \| \| Loss of sleep \| 1 \| 2 \| \| Excessive thirst \| 1 \| 2 \| \| Blood in stool \| 1 \| 2 \| \| Swelling in abdomen \| 1 \| 2 \| \| Abdominal pain \| 1 \| 2 \| \| Diarrhea \| 1 \| 2 \| | | |  |
|  | **Section 9: Biomarker** | | | |
|  | Weight in Kilogram | \| Kg: \|  \|  \|  \| . \|  \|  \| \| --- \| --- \| --- \| --- \| --- \| --- \| --- \|   Not present………….994  Refused………………..995  Other……………………996 | |  |
|  | Height in Centimeters | \| Cm: \|  \|  \|  \| . \|  \|  \| \| --- \| --- \| --- \| --- \| --- \| --- \| --- \|   Not present………….994  Refused………………..995  Other……………………996 | |  |
|  | Waist circumference in centimeters | \| Cm: \|  \|  \|  \| . \|  \|  \| \| --- \| --- \| --- \| --- \| --- \| --- \| --- \|   Not present………….994  Refused………………..995  Other……………………996 | |  |
|  | Hip circumference in centimeters | \| Cm: \|  \|  \|  \| . \|  \|  \| \| --- \| --- \| --- \| --- \| --- \| --- \| --- \|   Not present………….994  Refused………………..995  Other……………………996 | |  |
|  | Isometric hand grip strength in Kilogram | \| Kg: \|  \|  \|  \| . \|  \|  \| \| --- \| --- \| --- \| --- \| --- \| --- \| --- \|   Not present………….994  Refused………………..995  Other……………………996 | |  |
|  | The systolic and diastolic pressure for the BP reading   \|  \| Measurement1 \| Measurement2 \| Average \| \| --- \| --- \| --- \| --- \| \| Systolic \|  \|  \|  \| \| Diastolic \|  \|  \|  \|   Refused ………………………………….994  Technical problems ………………..995  Other………………………………………996 | | |  |
|  | When you have taken food last? | 1 hour …………………1  1 to 2 hours ……………2  More than 2 hours …….3 | |  |
|  | Record blood glucose in MG/DL | \| mg/dl: \|  \|  \|  \| \| --- \| --- \| --- \| --- \|   Refused …………………… 995  Other…………………………996  Not tested……………………997 | |  |
|  | Record haemoglobin level | \| g/dl: \|  \|  \|  \| . \|  \| \| --- \| --- \| --- \| --- \| --- \| --- \|   Refused …………………… 995  Other……………………………996  Not tested……………………997 | |  |
|  | Record the SCD/SCT percentage (%) here | \| Haemoglobin A2/C/E \| % \| \| --- \| --- \| \| Haemoglobin S \| % \| \| Haemoglobin F \| % \| \| Haemoglobin A \| % \|   Refused ……………………… 995  Other……………………………996  Not tested………………………997 | |  |
| **Section 10: Rating of health** | | | | |
| 1001 | We would like to know how good or bad your health is TODAY.  This scale is numbered from 0 to 100  100 means the best health you can imagine. 0 means the worst health you can imagine.  Mark an X on the scale to indicate how your health is TODAY  Now, please write the number you marked on the scale in the box below. | Your Health Today = | |  |

**************************************END************************************************

**Age 20-59 Years Old Women Questionnaire**

| **IDENTIFICATION** | |
| --- | --- |
| DISTRICT NAME:  BLOCK NAME:  CLUSTER NAME:  TYPE OF PSU (URBAN=1, RURAL=2):  HOUSEHOLD NUMBER:  INDIVIDUAL ID:  INTERVIEW DATE:  INTERVIEW START TIME:  NAME OF THE INVESTIGATOR:  CODE OF THE INVESTIGATOR:  SIGNATURE OF THE INVESTIGATOR:  INTERVIEW RESULTS: 1) COMPLETED  2) NOT AT HOME  3) POSTPONED  4) REFUSED  5) PARTLY COMPLETED  6) INCAPACITATED  7) OTHERS  (SPECIFY __________________) | DISTRICT CODE:  BLOCK CODE:  CLUSTER CODE:  HOUSEHOLD ID:  INTERVIEW END TIME: |

|  | | | | |
| --- | --- | --- | --- | --- |
| **Section 1:  Respondent's Background Characteristics** | | | | |
| **No.** | **Questions and Filters** | **Coding Categories** | | **Skip** |
|  | Name of the women? |  | |  |
|  | Age  (Verify from valid document) | Date of birth:  Age___________ in years | |  |
|  | Have you ever attended school? | Yes ……………1  No …………...2 | | If no, skip que.104 |
|  | What is the completed years of education? | Grade completed: ________ Years | |  |
|  | What is your religion? | Hindu…………………………….1  Muslim…………………………..2  Christian…………………………3  No religion ………………………4  Other……………………………..9  (Specify ___________________________) | |  |
|  | Ethnic group | Listed ST (Scheduled Tribe)………………1  Primitive Vulnerable Tribal Groups (PVTGs) …2 | |  |
|  | Name of the tribe/PVTG? | **ST**  Bagata ……………………………..1  Baiga ………………………………..2  Banjara …………………………….3  Bathudi …………………………….4  Bhottada ………………………….5  Bhuiya ………………………………6  Bhumia ……………………………..7  Bhumij ……………………………...8  Bhunjia ………………………………9  Binjhal ……………………………….10  Binjhia ……………………………….11  Birhor ………………………………..12  BondoPoraja ……………………..13  Chenchu …………………………….14  Dal ……………………………………..15  Desia Bhumij ………………………16  Dharua ……………………………….17  Didayi ………………………………….18  Gadaba ………………………………..19  Gandia …………………………………20  Ghara …………………………………..21  Gond ……………………………………22  Ho ………………………………………..23  Holva ……………………………………24  Jatapu ………………………………….25  Juang ……………………………………26  Kandha Gauda ……………………...27  Kawar ……………………………………28  Kharia ……………………………………29  Kharwar ………………………………..30  Khond ……………………………………31  Kisan ………………………………………32  Kol …………………………………………33  Kolah Loharas …………………….34  Kolha ………………………………….35  Koli ……………………………………..36  Kondadora ………………………….37  Kora …………………………………….38  Korua …………………………………..39  Kotia …………………………………….40  Koya ……………………………………..41  Kulis ………………………………………42  Lodha …………………………………….43  Madia ……………………………………44  Mahali …………………………………..45  Mankidi …………………………………46  Mankirdia ………………………………47  Matya ……………………………………48  Mirdhas …………………………………49  Munda …………………………………..50  Mundari …………………………………51  Omanatya ………………………………52  Oraon …………………………………….53  Parenga …………………………………54  Paroja ……………………………………55  Pentia …………………………………….56  Rajuar ……………………………………57  Santal …………………………………….58  Saora ……………………………………..59  Shabar……………………………………60  Sounti ……………………………………61  Tharua …………………………………..62  **PVTGs**  Birhor …………………………………….63  Bondo ……………………………………64  Chuktia Bhunjia………………………65  Didayi …………………………………….66  Juang ……………………………………..67  Kharia …………………………………….68  Dongria Khond ………………………69  Kutia Khond …………………………..70  Lanjia Saora ……………………………71  Lodha …………………………………….72  Mankidia ……………………………….73  Paudi Bhuyan …………………………74  Saora ……………………………………..75 | |  |
|  | What is your occupation, that is, what kind of work do you mainly do? | Professional (technical/ administrative/  Managerial, etc.) ……………………1  Clerical ………………………………2  Sales worker …………………………3  Service worker ……………………….4  Production worker (skilled& unskilled) 5  Agricultural …………………………6  Horticulture …………………………7  Wage Earning ………………………8  Shifting cultivation …………………9  Forest collection …………………….10  Food gathering ………………………11  Small business ………………………12  Fishing ………………………………13  Going to school/studying ……………14  Looking for work ……………………..15  Retired ………………………………...16  Unable to work/ill/handicapped ………17  Housework/childcare …………………18  Others …………………………………19  (Specify_______________________) | |  |
|  | Do you usually work throughout the year, or do you work seasonally, or only once in a while? | Throughout the Year ………….1  Seasonally/Part of The Year…..2  Once in A While ………………3 | |  |
|  | Are you paid in cash or kind for this, or you are not paid at all? | Cash only………………1  Cash and kind………….2  In kind only……………3  Not paid……………….4 | |  |
|  | What kind of toilet do you use? | Own toilet ……………………………1  Community toilet …………………….2  Shared toilet with other household……3  No facility/uses open space or field ….4 | |  |
|  | Do you use any of these to wash your hands? (Hand hygiene) | \|  \| After toilet \| Before food \| \| --- \| --- \| --- \| \| Soap/detergent \|  \|  \| \| Ash/mud \|  \|  \| \| Nothing \|  \|  \| \| Others \|  \|  \| | |  |
| **Section 2: Marriage & Reproduction** | | | | |
| **No.** | **Questions** | **Coding Categories** | | **Skip** |
|  | Marital status | Never married ……………………1  Currently married ………………..2  Widower …………………………3  Divorced …………………………4  Separated …………………………5  Live-In ……………………………6  Don’t want to say…………………8 | | If response is “never married” or “Don’t want to say” skip to 208 |
|  | Have you been married once or more than once? | Only Once……………….1  More than once………….2 | |  |
|  | Whether you are biologically related to your husband before marriage? | Yes ……………1  No …………...2 | | If ‘no’ then skip to q.205 |
|  | If yes, specify the marriage with | First cousin (paternal and maternal) ……1  Second cousin …………………………..2  Uncle–niece …………………………….3  Others …………………………………..4  Not related ……………………………...5 | |  |
|  | How old were you when you (first) got married? | _____________completed years | |  |
|  | In what month and year did you get married? |  | |  |
|  | How many times have you conceived till now? | No. of Pregnancies: | |  |
|  | How many months after marriage did you conceive for the first time? | Months: __ | |  |
|  | Number of live births | Number: | |  |
|  | Number of still births | Number: | |  |
|  | Number of abortions | Number: | |  |
|  | Number of living children | Son:  Daughter:  Total: | |  |
|  | Number of children died | Son:  Daughter:  Total: | |  |
|  | Number of births in the last five years | Number:__ | |  |
|  | When was the last child born? | \| DD \| MM \| YY \| \| --- \| --- \| --- \| \|  \|  \|  \| | |  |
|  | Are you pregnant now? | Yes…………….1  No……………...2  Unsure…………8 | | If ‘yes’ ask 217 |
|  | How many months pregnant are you? | Months:_________ | |  |
|  | Where do you planned to deliver your baby? | Husband/In-laws place ……………1  Maternal place …………………….2  Other . ……………………………3  (Specify _____________________) | |  |
| **Section 3: Family Planning (Currently married women 15-49 years) & Unmet Need for Family Planning** | | | | |
|  | Have you ever heard of anything to delay or avoid getting pregnant? | Yes……………………1  No…………………….2 | |  |
|  | Knowledge & Practice of methods of family planning that couple can use to avoid pregnancy?  (Check list – multiple options), if knowledge is checked then ask about practice.  (Show the prepared chart for better understanding)   \|  \| Knowledge \| Practice \| \| --- \| --- \| --- \| \| Female sterilization \|  \|  \| \| Male sterilization \|  \|  \| \| IUD/PPIUD \|  \|  \| \| Injectables \|  \|  \| \| Pill \|  \|  \| \| Condom/nirodh \|  \|  \| \| Female condom \|  \|  \| \| Emergency contraception \|  \|  \| \| Diaphragm \|  \|  \| \| Foam/jelly \|  \|  \| \| Standard days method \|  \|  \| \| Lactational Amenorrhoea Method \|  \|  \| \| Rhythm method \|  \|  \| \| Withdrawal \|  \|  \| \| Other modern method \|  \|  \| \| Other traditional method \|  \|  \| | | |  |
|  | Were you ever told by a health worker about any methods of family planning that you can use to avoid pregnancy? | Yes…………………..1  No……………………2 | |  |
|  | You started using (CURRENT METHOD) in (MONTH/YEAR). At that time, were you told about side effects or problems you might have with the method? | Yes…………………..1  No……………………2 | |  |
|  | When you got pregnant, did you want to get pregnant at that time? | Yes…………………..1  No……………………2 | |  |
|  | Did you want to have the baby later on or did you not want any more children? | Later……………………1  No more/none…………...2 | |  |
| **Section 4: Knowledge of HIV/AIDS among Adults (age 15-49 years)** | | | | |
|  | Now I would like to talk about something else. Have you ever heard of an illness called AIDS/HIV? | Yes…………………..1  No……………………2 | | If 401 is ‘no’, skip to section5 |
|  | Knowledge regarding HIV/AIDS   \| Transmission knowledge \| Prevention knowledge \| Treatment knowledge \| \| --- \| --- \| --- \| \|  \|  \|  \|   Adequate knowledge ………………..1  Inadequate knowledge ………………2 | | |  |
|  | Can people reduce their chances of getting HIV by using a condom every time they have sex? | Yes……………………1  No…………………….2  Don’t know…………...8 | |  |
| **Section 5: Women's Empowerment, Menstrual Hygiene & Gender based Violence** | | | | |
|  | Who usually makes decisions about health care for yourself: mainly you, mainly your husband, you and your husband jointly, or someone else? | Respondent………………….1  Husband…………………….2  Respondent and husband jointly………………………..3  Someone else………………..4  Other………………………...5 | |  |
|  | Who usually makes decisions about the following?   \| Health care of yourself \| Major household purchase \| Visit to your family or relatives \| \| --- \| --- \| --- \| \|  \|  \|  \|   Respondent……………………………………………..1  Husband………………………………………………...2  Respondent and husband jointly………………………..3  Someone else……………………………………………4  Other……………………….............................................5 | | |  |
|  | Do you own this or any other house/ any agricultural or non-agricultural land either alone or jointly with someone else? | Alone only……………1  Jointly only……………2  Both alone and jointly…3  Does not own………….4 | |  |
|  | Do you have a bank or savings account that you yourself use? | Yes……………………….1  No………………………..2 | |  |
|  | Do you have any mobile phone that you yourself use? | Yes……………………….1  No………………………..2 | | If ‘yes’ ask 506 |
|  | Do you use mobile phones for any financial transactions? | Yes ……………1  No …………...2 | |  |
|  | Have you ever used the internet? | Yes ……………1  No …………...2 | |  |
|  | Are you covered by any health scheme or health insurance? | Yes ……………1  No …………...2 | | If no, skip que. 509 |
|  | What type of health scheme or health insurance? | \|  \| Yes \| No \| \| --- \| --- \| --- \| \| Employees State Insurance Scheme (ESIS) \| 1 \| 2 \| \| Central Govt. Health Schemes (CGHS) \| 1 \| 2 \| \| Biju Swasthya Kalyan Yojana (BSKY) \| 1 \| 2 \| \| Biju Krushak Kalyan Yojana (BKKY) \| 1 \| 2 \| \| Odisha State Treatment Fund (OSTF) \| 1 \| 2 \| \| Rashtriya Swasthya Bima Yojana (RSBY) \| 1 \| 2 \| \| Community Health Insurance Programme \| 1 \| 2 \| \| Other Health Insurance Through Employer \| 1 \| 2 \| \| Other Privately Purchased Commercial \| 1 \| 2 \| \| Health Insurance \| 1 \| 2 \| | |  |
| **Menstrual Hygiene** | | | | |
|  | Have you ever had your monthly period? | Yes…………….1  No……………...2 | | If ‘no’ then goto 524 |
|  | How old were you when you had your first monthly period? |  | |  |
|  | What was the most common absorbent material used during last menstrual period? | \|  \| Yes \| No \| \| --- \| --- \| --- \| \| Cloth \| 1 \| 2 \| \| Locally prepared napkins \| 1 \| 2 \| \| Sanitary napkins \| 1 \| 2 \| \| Tampons \| 1 \| 2 \| \| Menstrual cup \| 1 \| 2 \| \| Nothing \| 1 \| 2 \| \| Other  (Specify________) \| 1 \| 2 \| | |  |
|  | How do you wash your sanity cloths? | With water …………………………….1  With water & soap or detergent ………2  With water & mud/ash ………………..3  Other ………………………………….4 | |  |
|  | What do you do with the absorbent material | Dispose it …………….1  Reuse it ……………….2 | |  |
|  | During menstruation is there any restriction for following places? | \|  \| Yes \| No \| \| --- \| --- \| --- \| \| Religious place \| 1 \| 2 \| \| Shrine \| 1 \| 2 \| \| School \| 1 \| 2 \| \| Kitchen \| 1 \| 2 \| \| Bathroom \| 1 \| 2 \| \| River \| 1 \| 2 \| \| Pond \| 1 \| 2 \| \| Living room \| 1 \| 2 \| \|  \|  \|  \| \|  \|  \|  \| | |  |
|  | During menstruation following activities are restricted | \|  \| Yes \| No \| \| --- \| --- \| --- \| \| Cooking \| 1 \| 2 \| \| Bathing \| 1 \| 2 \| \| Studying \| 1 \| 2 \| \| Routine household activities \| 1 \| 2 \| \| Playing \| 1 \| 2 \| \|  \|  \|  \| | |  |
|  | During menstrual period, where do you stay | Inside the home ………….1  Outside the house but in the village ……….2  Outside the house but in the village in a room or hut……………………………………3  Outskirt of the village in open …………4  Outskirt of the village in a room or hut …….5 | |  |
|  | **Gender Based Violence** | | | |
|  | Have you ever encountered violence in any form? | Yes……………………….1  No………………………..2 | |  |
|  | By whom | Husband ………………………1  Ex-husband ……………………2  Partner …………………………3  Brother …………………………4  Father …………………………..5  Father in law ……………………6  Brother in law …………………..7  Other relatives …………………..8  Neighbours ………………………9  Friends/colleges/teachers ………10  Others……………………………11  (Specify_____________________) | |  |
|  | What type of violence did you experienced? | Physical …………..1  Sexual …………….2  Both ………………3 | |  |
|  | Check 207, If conceived then ask  During pregnancy time have experienced any type of violence? | Yes……………………….1  No………………………..2 | |  |
| **Section 6: Tobacco Use and Alcohol Consumption among Adults (age 15 years and above)** | | | | |
|  | Do you currently smoke or use tobacco in any other form? | Yes……………………….1  No………………………..2  Don’t want to say ……….8 | | If ‘no’ then goto 606 |
|  | In what other form do you currently smoke or use tobacco? Any other form?   \| Tobacco form \| Frequency \| How often do you use tobacco?  Almost every day……1  Once a week………...2  less than once a week.3  Don’t want to say …..8  (Fill this when the frequency of smoking is more than 0) \| How long have you been smoking?   \| W \| W \| M \| M \| Y \| Y \| \| --- \| --- \| --- \| --- \| --- \| --- \|   (Fill this when the frequency of smoking is more than 0) \| \| --- \| --- \| --- \| --- \| --- \| --- \| --- \| --- \| --- \| --- \| \| Cigar \|  \|  \|  \| \| A pipe \|  \|  \|  \| \| Hookah \|  \|  \|  \| \| Gutka / paan masala \|  \|  \|  \| \| Tobacco \|  \|  \|  \| \| Khaini \|  \|  \|  \| \| Paan with tobacco \|  \|  \|  \| \| Other chewing tobacco \|  \|  \|  \| \| Snuff \|  \|  \|  \| \| Other  (Specify___) \|  \|  \|  \| | | | |
|  | During the last 12 months, have you ever tried to stop smoking or using tobacco in any other form? | Yes ……………1  No …………...2 | |  |
|  | In last 12 months, have you visited a doctor/ other health care provider? | Yes ……………1  No …………...2 | |  |
|  | During these visits, were you advised to quit smoking or using tobacco in any other form? | Yes ……………1  No …………...2 | |  |
|  | Do you drink alcohol? | Yes……………………….1  No………………………..2  Don’t want to say ……….8 | | If ‘no then goto ask 610 |
|  | \|  \| Frequency \| How often do you use tobacco?  Almost every day……1  Once a week………...2  less than once a week.3  Don’t want to say …..8  (Fill this when the frequency of alcohol is more than 0) \| How long have you been smoking   \| W \| W \| M \| M \| Y \| Y \| \| --- \| --- \| --- \| --- \| --- \| --- \|   (Fill this when the frequency of alcohol is more than 0) \| \| --- \| --- \| --- \| --- \| --- \| --- \| --- \| --- \| --- \| --- \| \| Tadi madi \|  \|  \|  \| \| Country Liquor \|  \|  \|  \| \| Beer \|  \|  \|  \| \| Wine \|  \|  \|  \| \| Hard Liquor \|  \|  \|  \| \| Other  (Specify___) \|  \|  \|  \| | | |  |
|  | During the last 12 months, have you ever tried to stop alcohol? | Yes ……………1  No …………...2 | |  |
|  | Have you ever done any of the following?   \|  \| Yes \| No \| Where did you get?  Home ……1  Shop …….2  Other …… 3  (Specify ________) \| \| --- \| --- \| --- \| --- \| \| Tattooing \|  \|  \|  \| \| Piercing \|  \|  \|  \| | | |  |
| **Section 7: Maternal and Child Health (Mothers - for last birth in the 5 years before the survey, , if the child is more than 5 years then skip section 7)** | | | | |
| Check 211 if number of births is one or more ask section 7 | | | | |
|  | Was your last pregnancy got registered? | Yes……………………….1  No………………………..2 | |  |
|  | How many months pregnant were you when you registered? | Months:___  Don’t know…………98 | |  |
|  | With whom did you register? | ANM…………………..1  ASHA…………………..2  AWW………………….3  Other………………….4 | |  |
|  | Did you receive a Mother and Child Protection Card after registration? | Yes……………………….1  No………………………..2 | |  |
|  | How many months pregnant were you when you first received antenatal care for this pregnancy? | Months:___  Don’t know……………….98 | |  |
|  | How many times did you receive antenatal care during this pregnancy? | Num of times:__  Don’t know……………….98 | |  |
|  | During this pregnancy, were you given an injection in the arm to prevent the baby from getting tetanus, that is, convulsions after birth? | Yes…………………………..1  No……………………………2  Don’t know……………..8 | |  |
|  | During this pregnancy, how many times did you get a tetanus injection? | Times:__  Don’t know……………….8 | |  |
|  | At any time before this pregnancy, did you receive any tetanus injections? | Yes……………………………1  No……………………………2  Don’t know………………8 | |  |
|  | Before this pregnancy, how many times did you receive a tetanus injection? | Times:__  Don’t know…………….8 | |  |
|  | How many years ago did you receive the last tetanus injection before this pregnancy? | Years ago:__ | |  |
|  | During this pregnancy, were you given or did you buy any iron folic acid tablets or syrup? | Yes……………………………1  No…………………………….2  Don’t know…………………..8 | |  |
|  | During the whole pregnancy, for how many days did you take the tablets or syrup? | Num of days:__  Don’t know……………………….998 | |  |
|  | Where did you give birth to (NAME)? | Pub. Health sector………………..1  Pvt. Health sector………………...2  Home……………………………..3  Other……………………………..4  (Specify._____________) | |  |
|  | In which place the child was born? | Husband/In-laws place ……………1  Maternal place …………………….2  Other . ……………………………3  (Specify _____________________) | |  |
|  | Was (NAME) delivered by caesarean section, that is, did they cut your belly open to take the baby out? | Yes……………………….1  No………………………..2 | | If ‘yes’ ask 716 |
|  | When was the decision made for you to have a C-section? Was it before the onset of labour or after the onset of labour? | Before onset of labour……………1  After onset of labour………………2  Don't know…………………………….3 | |  |
|  | Who conducted/ assisted with the delivery of (NAME)? | \|  \| YES \| NO \| \| --- \| --- \| --- \| \| **Health personnel** \|  \|  \| \| Doctor \| 1 \| 2 \| \| Anm/nurse/ midwife/lhv \| 1 \| 2 \| \| Other health professional \| 1 \| 2 \| \| **Other Person** \|  \|  \| \| Dai (tba) \| 1 \| 2 \| \| Friend/relative \| 1 \| 2 \| \| No one \| 1 \| 2 \| \| Others  (specify___________) \| 1 \| 2 \| | |  |
|  | Did anyone check on your health while you were still in the facility? | Yes……………………….1  No………………………..2 | |  |
|  | How long after delivery did the first check take place? | Hours:__  Days:__  Weeks:__  Don’t know ………………..98 | |  |
|  | Who checked your health at that time? | \|  \| YES \| NO \| \| --- \| --- \| --- \| \| **Health personnel** \|  \|  \| \| Doctor \| 1 \| 2 \| \| Anm/nurse/ midwife/lhv \| 1 \| 2 \| \| Other health professional \| 1 \| 2 \| \| **Other Person** \|  \|  \| \| Dai (tba) \| 1 \| 2 \| \| Friend/relative \| 1 \| 2 \| \| No one \| 1 \| 2 \| \| Others  (specify___________) \| 1 \| 2 \| | |  |
|  | How much in total did it cost you out of your pocket for this delivery? | Cost Rs._____  Don’t know…………99998 | |  |
|  | Check 714, if ‘home’ then ask 722 & 723 | | |  |
|  | After your baby born, have you taken the baby to any health facility for check-up with in 24 hours after birth? | Yes ……………1  No …………….2 | |  |
|  | Who checked your child’s health at that time? | \|  \| YES \| NO \| \| --- \| --- \| --- \| \| **Health personnel** \|  \|  \| \| Doctor \| 1 \| 2 \| \| Anm/nurse/ midwife/lhv \| 1 \| 2 \| \| Other health professional \| 1 \| 2 \| \| **Other Person** \|  \|  \| \| Dai (tba) \| 1 \| 2 \| \| Friend/relative \| 1 \| 2 \| \| No one \| 1 \| 2 \| \| Others  (specify___________) \| 1 \| 2 \| | |  |
| **Section 8- Chronic Disease Conditions** | | | | |
| 801 | \| Have you ever diagnosed with any of the following disease condition by any health care provider? \| \| \| \| If YES, Have you sought treatment for this \| \| \| --- \| --- \| --- \| --- \| --- \| --- \| \|  \| Yes \| No \| Don’t Know \| Yes \| No \| \| 1.Diabetes \| 1 \| 2 \| 8 \| 1 \| 2 \| \| 2. Hypertension \| 1 \| 2 \| 8 \| 1 \| 2 \| \| 3. Arthritis \| 1 \| 2 \| 8 \| 1 \| 2 \| \| 4. Acid Peptic Disease \| 1 \| 2 \| 8 \| 1 \| 2 \| \| 5. Asthma \| 1 \| 2 \| 8 \| 1 \| 2 \| \| 6. heart diseases \| 1 \| 2 \| 8 \| 1 \| 2 \| \| 7. Stroke \| 1 \| 2 \| 8 \| 1 \| 2 \| \| 8. chronic kidney diseases \| 1 \| 2 \| 8 \| 1 \| 2 \| \| 9. Chronic Liver Disease(alcoholic) \| 1 \| 2 \| 8 \| 1 \| 2 \| \| 10.Chronic Back Ache \| 1 \| 2 \| 8 \| 1 \| 2 \| \| 11. Tuberculosis \| 1 \| 2 \| 8 \| 1 \| 2 \| \| 12. Filariasis \| 1 \| 2 \| 8 \| 1 \| 2 \| \| 13. Visual difficulty \| 1 \| 2 \| 8 \| 1 \| 2 \| \| 14. Deafness \| 1 \| 2 \| 8 \| 1 \| 2 \| \| 15. Cancer \| 1 \| 2 \| 8 \| 1 \| 2 \| \| 16. Dementia \| 1 \| 2 \| 8 \| 1 \| 2 \| \| 17. Epilepsy \| 1 \| 2 \| 8 \| 1 \| 2 \| \| 18. Thyroid disease \| 1 \| 2 \| 8 \| 1 \| 2 \| \| 19. Cancer \| 1 \| 2 \| 8 \| 1 \| 2 \| \| 20. Sickle cell disorder \| 1 \| 2 \| 8 \| 1 \| 2 \| \| 21.Confusion \| 1 \| 2 \| 8 \| 1 \| 2 \| \| 22. Anxiety \| 1 \| 2 \| 8 \| 1 \| 2 \| \| 23. Disturbed sleep \| 1 \| 2 \| 8 \| 1 \| 2 \| \| 24. Frequent infection \| 1 \| 2 \| 8 \| 1 \| 2 \| \| 25. Weakness \| 1 \| 2 \| 8 \| 1 \| 2 \| \| 26. Joint pain \| 1 \| 2 \| 8 \| 1 \| 2 \| \| 27. Hands pain \| 1 \| 2 \| 8 \| 1 \| 2 \| \| 28. Feet pain \| 1 \| 2 \| 8 \| 1 \| 2 \| \| 29. Muscle pain \| 1 \| 2 \| 8 \| 1 \| 2 \| \| Any other chronic disease  (Specify_____________________) \| 1 \| 2 \| 8 \| 1 \| 2 \| | | |  |
| 802 | If 801 is ‘Cancer’, Specify name of the cancer | Oral Cancer ………………..1  Breast Cancer ………………2  Cervical Cancer ……………3  Others ………………………4  (Specify _________________) | |  |
| 803. | If 801 is ‘Sickle cell disease’, then ask the following   \| Specify the condition  Sickle cell disease ………1  Sickle cell trait ………….2  Sickle cell disorder (but don’t know which one) …3 \| when the test was done?  Month:  Year: \| If found “positive” what advice was given by doctor/nurse/ANM  Medical advice …………1  Dietary advice …………2  Both ……………………3 \| \| --- \| --- \| --- \| \|  \|  \|  \| | | |  |
| 804. | Have you ever taken HBV vaccination? | | Yes ………………1  No ………………2 |  |
| 805. | Have you ever under gone through blood transfusion process? | | Yes ……………1  No …………….2 |  |
| 806. | Have you ever under gone through IV process? | | Yes ……………1  No …………….2 |  |
| **Section- 9: Health care seeking behavior** | | | | |
|  | Did you visit any health facility for treatment of any disease or injury during past 12 months? | Yes……………………….1  No………………………..2 | | If response is “no” skip que no. 902-905 |
|  | Where did you visit for your treatment? | Government health facility………..1  Private health facility…………………2 | |  |
|  | For which condition did you visit the health facility? | Acute/Emergency……………………1  Chronic……………………………….2  Injury………………………………….3  Pregnancy related conditions …………4 | |  |
|  | Specify the condition |  | |  |
|  | Are you cured of the condition now? | Cured………………………………….1  Not cured……………………………2 | |  |
|  | **Section 10: Symptomatic Profile** | | |  |
|  | Has your blood pressure ever been checked prior to this survey? | Yes ……………………………….1  No ………………………………..2 | | If ‘yes’ ask 1002 - 1004 |
|  | When did you check your blood pressure last? | Month:  Year: | |  |
|  | Have you been informed by any Doctor/Nurse/ANM that you have high/low BP? | Yes ……………………………….1  No ………………………………..2 | |  |
|  | Are you taking any medicine for BP? | Yes ……………………………….1  No ………………………………..2 | |  |
|  | Have you ever been tested for anaemia? | Yes ……………………………….1  No ………………………2 | | If ‘yes’ ask 1006 |
|  | If found “positive” what advice was given by doctor/nurse/ANM? | Medical advice …………1  Dietary advice …………2  Both ……………………3 | |  |
|  | Have you ever been tested for blood sugar | Yes ……………………………….1  No ………………………………..2 | | If ‘yes’ ask 1008 - 1010 |
|  | If “yes” when blood sugar was checked last time? | Month:  Year: | |  |
|  | What was the result of blood sugar test? | Low ……………………………..1  Normal …………………………2  High ……………………………...3 | |  |
|  | If the answer is “high” what advice was given by doctor/nurse/ANM? | Medical advice …………1  Dietary advice …………2  Both ……………………3 | |  |
|  | Do you have any of the following acute symptoms?  (In the last one month)   \| Fever \| Yes \| No \| \| --- \| --- \| --- \| \| Vomiting \| 1 \| 2 \| \| Nausea \| 1 \| 2 \| \| Pain \|  \|  \| \| 1. Joint \| 1 \| 2 \| \| 1. Hands \| 1 \| 2 \| \| 1. Feet \| 1 \| 2 \| \| 1. Muscle \| 1 \| 2 \| \| Body ache \| 1 \| 2 \| \| Headache \| 1 \| 2 \| \| Stomachache \| 1 \| 2 \| \| Anemia \| 1 \| 2 \| \| Swelling Hands \| 1 \| 2 \| \| Swelling Feet \| 1 \| 2 \| \| Dark Urine \| 1 \| 2 \| \| Yellowing of the eyes and skin \| 1 \| 2 \| \| Jaundice \| 1 \| 2 \| \| Tiredness \| 1 \| 2 \| \| Loss of appetite \| 1 \| 2 \| \| Loss of sleep \| 1 \| 2 \| \| Excessive thirst \| 1 \| 2 \| \| Blood in stool \| 1 \| 2 \| \| Swelling in abdomen \| 1 \| 2 \| \| Abdominal pain \| 1 \| 2 \| \| Diarrhea \| 1 \| 2 \| | | |  |
|  | **Section 11: Biomarker** | | |  |
|  | Weight in Kilogram | \| Kg: \|  \|  \|  \| . \|  \|  \| \| --- \| --- \| --- \| --- \| --- \| --- \| --- \|   Not present………….994  Refused………………..995  Other……………………996 | |  |
|  | Height in Centimeters | \| Cm: \|  \|  \|  \| . \|  \|  \| \| --- \| --- \| --- \| --- \| --- \| --- \| --- \|   Not present………….994  Refused………………..995  Other……………………996 | |  |
|  | Waist circumference in centimeters | \| Cm: \|  \|  \|  \| . \|  \|  \| \| --- \| --- \| --- \| --- \| --- \| --- \| --- \|   Not present………….994  Refused………………..995  Other……………………996 | |  |
|  | Hip circumference in centimeters | \| Cm: \|  \|  \|  \| . \|  \|  \| \| --- \| --- \| --- \| --- \| --- \| --- \| --- \|   Not present………….994  Refused………………..995  Other……………………996 | |  |
|  | Isometric hand grip strength in Kilogram | \| Kg: \|  \|  \|  \| . \|  \|  \| \| --- \| --- \| --- \| --- \| --- \| --- \| --- \|   Not present………….994  Refused………………..995  Other……………………996 | |  |
|  | The systolic and diastolic pressure for the BP reading   \|  \| Measurement1 \| Measurement2 \| Average \| \| --- \| --- \| --- \| --- \| \| Systolic \|  \|  \|  \| \| Diastolic \|  \|  \|  \|   Refused ………………………………….994  Technical problems ………………..995  Other………………………………………996 | | |  |
|  | When you have taken food last? | 1 hour …………………1  1 to 2 hours ……………2  More than 2 hours …….3 | |  |
|  | Record blood glucose in MG/DL | \| mg/dl: \|  \|  \|  \| \| --- \| --- \| --- \| --- \|   Refused …………………………….995  Other…………………………………996  Not tested…………………………997 | |  |
|  | Record haemoglobin level | \| g/dl: \|  \|  \|  \| . \|  \| \| --- \| --- \| --- \| --- \| --- \| --- \|   Refused …………………………….995  Other…………………………………996  Not tested…………………………997 | |  |
|  | Record the SCD/SCT percentage (%) here | \| Haemoglobin A2/C/E \| % \| \| --- \| --- \| \| Haemoglobin S \| % \| \| Haemoglobin F \| % \| \| Haemoglobin A \| % \|   Refused …………………………….995  Other…………………………………996  Not tested…………………………997 | |  |
| **Section 12: Rating of health** | | | | |
| 1201 | We would like to know how good or bad your health is TODAY.  This scale is numbered from 0 to 100  100 means the best health you can imagine. 0 means the worst health you can imagine.  Mark an X on the scale to indicate how your health is TODAY  Now, please write the number you marked on the scale in the box below. | Your Health Today = | |  |

**************************************END************************************************

**Age 60+ years old (Geriatric Population) Questionnaire**

| **IDENTIFICATION** | |
| --- | --- |
| DISTRICT NAME:  BLOCK NAME:  CLUSTER NAME:  TYPE OF PSU (URBAN=1, RURAL=2):  HOUSEHOLD NUMBER:  INDIVIDUAL ID:  INTERVIEW DATE:  INTERVIEW START TIME:  NAME OF THE INVESTIGATOR:  CODE OF THE INVESTIGATOR:  SIGNATURE OF THE INVESTIGATOR:  INTERVIEW RESULTS: 1) COMPLETED  2) NOT AT HOME  3) POSTPONED  4) REFUSED  5) PARTLY COMPLETED  6) INCAPACITATED  7) OTHERS  (SPECIFY _______________) | DISTRICT CODE:  BLOCK CODE:  CLUSTER CODE:  HOUSEHOLD ID:  INTERVIEW END TIME: |

| **Section 1:  Respondent's Background Characteristics** | | | | |
| --- | --- | --- | --- | --- |
| **No.** | **Questions and Filters** | | **Coding Categories** | **Skip** |
|  | What is your name? | |  |  |
|  | Age  [Verify from valid card] | | Date of birth:  Age___________ in years |  |
|  | Have you ever attended school? | | Yes ……………1  No …………...2 | If ‘no’, skip q.104 |
|  | What is the highest grade you completed? | | Grade completed: ________ Years |  |
|  | What is your religion? | | Hindu…………………………….1  Muslim…………………………..2  Christian…………………………3  No religion ………………………4  Other……………………………..9  (Specify ___________________________) |  |
|  | Ethnic group | | Listed ST (Scheduled Tribe)……………………1  Primitive Vulnerable Tribal Groups (PVTGs) …2 |  |
|  | Name of the tribe/PVTG? | | **ST**  Bagata ……………………………..1  Baiga ………………………………..2  Banjara …………………………….3  Bathudi …………………………….4  Bhottada ………………………….5  Bhuiya ………………………………6  Bhumia ……………………………..7  Bhumij ……………………………...8  Bhunjia ………………………………9  Binjhal ……………………………….10  Binjhia ……………………………….11  Birhor ………………………………..12  BondoPoraja ……………………..13  Chenchu …………………………….14  Dal ……………………………………..15  Desia Bhumij ………………………16  Dharua ……………………………….17  Didayi ………………………………….18  Gadaba ………………………………..19  Gandia …………………………………20  Ghara …………………………………..21  Gond ……………………………………22  Ho ………………………………………..23  Holva ……………………………………24  Jatapu ………………………………….25  Juang ……………………………………26  Kandha Gauda ……………………...27  Kawar ……………………………………28  Kharia ……………………………………29  Kharwar ………………………………..30  Khond ……………………………………31  Kisan ………………………………………32  Kol …………………………………………33  Kolah Loharas …………………….34  Kolha ………………………………….35  Koli ……………………………………..36  Kondadora ………………………….37  Kora …………………………………….38  Korua …………………………………..39  Kotia …………………………………….40  Koya ……………………………………..41  Kulis ………………………………………42  Lodha …………………………………….43  Madia ……………………………………44  Mahali …………………………………..45  Mankidi …………………………………46  Mankirdia ………………………………47  Matya ……………………………………48  Mirdhas …………………………………49  Munda …………………………………..50  Mundari …………………………………51  Omanatya ………………………………52  Oraon …………………………………….53  Parenga …………………………………54  Paroja ……………………………………55  Pentia …………………………………….56  Rajuar ……………………………………57  Santal …………………………………….58  Saora ……………………………………..59  Shabar……………………………………60  Sounti ……………………………………61  Tharua …………………………………..62  **PVTGs**  Birhor …………………………………….63  Bondo ……………………………………64  Chuktia Bhunjia………………………65  Didayi …………………………………….66  Juang ……………………………………..67  Kharia …………………………………….68  Dongria Khond ………………………69  Kutia Khond …………………………..70  Lanjia Saora ……………………………71  Lodha …………………………………….72  Mankidia ……………………………….73  Paudi Bhuyan …………………………74  Saora ……………………………………..75 |  |
|  | What is your occupation, that is, what kind of work do you mainly do? | | Professional (technical/ administrative/  Managerial, etc.) ………………………..1  Clerical ………………………………….2  Sales worker …………………………….3  Service worker …………………………..4  Production worker (skilled& unskilled) …5  Agricultural ………………………………6  Horticulture ………………………………7  Wage Earning ……………………………8  Shifting cultivation ………………………9  Forest collection …………………………10  Food gathering …………………………11  Small business ………………………….12  Fishing ………………………………….13  Going to school/studying ……………….14  Looking for work ……………………….15  Retired …………………………………..16  Unable to work/ill/handicapped …………17  Housework/childcare ……………………18  I don’t work …………………………… 19  Others …………………………………...20  (Specify_______________________) |  |
|  | Do you usually work throughout the year, or do you work seasonally, or only once in a while? | | Throughout the Year ………….1  Seasonally/Part of The Year…...2  Once in A While ………………3 |  |
|  | Are you paid in cash or kind for this, or you are not paid at all? | | Cash only………………1  Cash and kind………….2  In kind only……………3  Not paid……………….4 |  |
|  | Please tell me, whether you are currently receiving pension or expect to receive pension in future. | | Currently receiving…………………..1  Expected to receive in future………...2  Neither currently receiving nor  expected to receive in future…………3 |  |
|  | How much money did you receive in last month as pension? | | Rs. |  |
|  | Do you have mobile phone that you use yourself? | | Yes ……………1  No …………...2 | If ‘no’ then skip q.114 |
|  | Do you use mobile phones for any financial transactions? | | Yes ……………1  No …………...2 |  |
|  | Do you have an account in a bank or other financial institutions that you yourself use? | | Yes ……………1  No …………...2 |  |
|  | Have you ever used the internet? | | Yes ……………1  No …………...2 |  |
|  | What kind of toilet do you use? | | Own toilet ……………………………1  Community toilet …………………….2  Shared toilet with another household……3  No facility/uses open space or field ….4 |  |
|  | Hand hygiene practices? | | \|  \| After toilet \| Before food \| \| --- \| --- \| --- \| \| Soap/detergent \|  \|  \| \| Ash/mud \|  \|  \| \| Plane water \|  \|  \| \| Nothing \|  \|  \| \| Others \|  \|  \| |  |
|  | What is your current marital status? | | Never married …………………1  Currently married ……………..2  Widower ………………………3  Divorced ………………………4  Separated ………………………5  Live-In …………………………6  Don’t want to say………………8 |  |
|  | How old were you when you (first) got married? | | _____________completed years  Date of marriage: mm/yyyy |  |
|  | Whether you are biologically related to your husband/wife before marriage? | | Yes ……………1  No …………...2 |  |
|  | If yes, specify the marriage with | | First cousin (paternal and maternal) ……1  Second cousin ………………………….2  Uncle–niece ……………………………3  Others …………………………………4 |  |
| **Section 2: Tobacco & Alcohol Consumption** | | | | |
|  | Do you currently smoke or use tobacco in any other form? | | Yes ………………..1  No …………………2  Don’t want to say ….8 | If ‘yes’ ask 202-205 |
|  | In what other form do you currently smoke or use tobacco? Any other form?   \| Tobacco form \| Frequency \| How often do you use tobacco?  Almost every day……1  Once a week………...2  less than once a week.3  Don’t want to say …..8  (Fill this when the frequency of smoking is more than 0) \| How long have you been using?   \| W \| W \| M \| M \| Y \| Y \| \| --- \| --- \| --- \| --- \| --- \| --- \|   (Fill this when the frequency of smoking is more than 0) \| \| --- \| --- \| --- \| --- \| --- \| --- \| --- \| --- \| --- \| --- \| \| Cigar \|  \|  \|  \| \| A pipe \|  \|  \|  \| \| Hookah \|  \|  \|  \| \| Gutka / paan masala \|  \|  \|  \| \| Tobacco \|  \|  \|  \| \| Khaini \|  \|  \|  \| \| Paan with tobacco \|  \|  \|  \| \| Other chewing tobacco \|  \|  \|  \| \| Snuff \|  \|  \|  \| \| Other  (Specify___) \|  \|  \|  \| | | |  |
|  | During the last 12 months, have you ever tried to stop smoking or using tobacco in any other form? | | Yes ……………1  No …………...2 |  |
|  | In last 12 months, have you visited a doctor/ other health care provider? | | Yes ……………1  No …………...2 |  |
|  | During these visits, were you advised to quit smoking or using tobacco in any other form? | | Yes ……………1  No …………...2 |  |
|  | Do you drink alcohol? | | Yes ………………..1  No …………………2  Don’t want to say ….8 | If ‘yes’ ask 207 - 208 |
|  | \|  \| Frequency \| How often do you use drink alcohol?  Almost every day……1  Once a week………...2  less than once a week.3  Don’t want to say …..8  (Fill this when the frequency of alcohol is more than 0) \| How long have you been drinking?   \| W \| W \| M \| M \| Y \| Y \| \| --- \| --- \| --- \| --- \| --- \| --- \|   (Fill this when the frequency of alcohol is more than 0) \| \| --- \| --- \| --- \| --- \| --- \| --- \| --- \| --- \| --- \| --- \| \| Tadi madi \|  \|  \|  \| \| Country Liquor \|  \|  \|  \| \| Beer \|  \|  \|  \| \| Wine \|  \|  \|  \| \| Hard Liquor \|  \|  \|  \| \| Other  (Specify___) \|  \|  \|  \| | | |  |
|  | During the last 12 months, have you ever tried to stop alcohol? | | Yes ……………1  No …………...2 |  |
|  | Have you ever done any of the following?   \|  \| Yes \| No \| Where did you get?  Home ……1  Shop …….2  Other …… 3  (Specify ________) \| \| --- \| --- \| --- \| --- \| \| Tattooing \|  \|  \|  \| \| Piercing \|  \|  \|  \| | | |  |
|  | Where do you generally go for shaving/hair cut? | | At home ………………….1  Saloon ……………………..2  Village barber …………..3 |  |
|  | Does the razor used for shaving is shared? | | Yes …………….1  No………………2 |  |
| **Section 3: Chronic Disease Conditions** | | | | |
| 301. | \| Have you ever diagnosed with any of the following disease condition by any health care provider? \| \| \| \| If YES, Have you sought treatment for this \| \| Since how long \| \| --- \| --- \| --- \| --- \| --- \| --- \| --- \| \|  \| Yes \| No \| Don’t Know \| Yes \| No \|  \| \| 1.Diabetes \| 1 \| 2 \| 8 \| 1 \| 2 \|  \| \| 2. Hypertension \| 1 \| 2 \| 8 \| 1 \| 2 \|  \| \| 3. Arthritis \| 1 \| 2 \| 8 \| 1 \| 2 \|  \| \| 4. Acid Peptic Disease \| 1 \| 2 \| 8 \| 1 \| 2 \|  \| \| 5. Asthma \| 1 \| 2 \| 8 \| 1 \| 2 \|  \| \| 6. heart diseases \| 1 \| 2 \| 8 \| 1 \| 2 \|  \| \| 7. Stroke \| 1 \| 2 \| 8 \| 1 \| 2 \|  \| \| 8. chronic kidney diseases \| 1 \| 2 \| 8 \| 1 \| 2 \|  \| \| 9. Chronic Liver Disease(alcoholic) \| 1 \| 2 \| 8 \| 1 \| 2 \|  \| \| 10.Chronic Back Ache \| 1 \| 2 \| 8 \| 1 \| 2 \|  \| \| 11. Tuberculosis \| 1 \| 2 \| 8 \| 1 \| 2 \|  \| \| 12. Filariasis \| 1 \| 2 \| 8 \| 1 \| 2 \|  \| \| 13. Visual difficulty \| 1 \| 2 \| 8 \| 1 \| 2 \|  \| \| 14. Deafness \| 1 \| 2 \| 8 \| 1 \| 2 \|  \| \| 15. Cancer \| 1 \| 2 \| 8 \| 1 \| 2 \|  \| \| 16. Dementia \| 1 \| 2 \| 8 \| 1 \| 2 \|  \| \| 17. Epilepsy \| 1 \| 2 \| 8 \| 1 \| 2 \|  \| \| 18. Thyroid disease \| 1 \| 2 \| 8 \| 1 \| 2 \|  \| \| 19. Cancer \| 1 \| 2 \| 8 \| 1 \| 2 \|  \| \| 20. Sickle cell disease \| 1 \| 2 \| 8 \| 1 \| 2 \|  \| \| 21.Confusion \| 1 \| 2 \| 8 \| 1 \| 2 \|  \| \| 22. Anxiety \| 1 \| 2 \| 8 \| 1 \| 2 \|  \| \| 23. Disturbed sleep \| 1 \| 2 \| 8 \| 1 \| 2 \|  \| \| 24. Frequent infection \| 1 \| 2 \| 8 \| 1 \| 2 \|  \| \| 25. Weakness \| 1 \| 2 \| 8 \| 1 \| 2 \|  \| \| 26. Joint pain \| 1 \| 2 \| 8 \| 1 \| 2 \|  \| \| 27. Hands pain \| 1 \| 2 \| 8 \| 1 \| 2 \|  \| \| 28. Feet pain \| 1 \| 2 \| 8 \| 1 \| 2 \|  \| \| 29. Muscle pain \| 1 \| 2 \| 8 \| 1 \| 2 \|  \| \| Any other chronic disease  (Specify________________) \| 1 \| 2 \| 8 \| 1 \| 2 \|  \| | | |  |
| 302. | If 301 is ‘Cancer’, Specify name of the cancer | Oral Cancer ………………..1  Pre cancers lesion ………………2  prostate cancer ……………3  Others ………………………4  (Specify _________________) | |  |
| 303. | If 301 is ‘Sickle cell disease’, then ask the following   \| Specify the condition  Sickle cell disease ………1  Sickle cell trait ………….2  Sickle cell disorder (but don’t know which one) …3 \| when the test was done?  Month:  Year: \| If found “positive” what advice was given by doctor/nurse/ANM  Medical advice …………1  Dietary advice …………2  Both ……………………3 \| \| --- \| --- \| --- \| \|  \|  \|  \| | | |  |
| 304. | Have you ever taken HBV vaccination? | | Yes ………………1  No ………………2 |  |
| 305. | Have you ever under gone through blood transfusion process? | | Yes ……………1  No …………….2 |  |
| 306. | Have you ever under gone through IV process? | | Yes ……………1  No …………….2 |  |
| **Section 4: Functional Limitations** | | | | |
|  | Do you have any physical or mental impairment or health problem that limits the kind or amount of paid work you can do? | | Yes ……………1  No …………...2 | If ‘yes’ ask 402 |
|  | Which form of impairment do you have? | | \|  \| Yes \| No \| \| --- \| --- \| --- \| \| Physical impairment such as lower body or upper body \| 1 \| 2 \| \| Mental impairment such as intellectual, cognition, or learning impairment \| 1 \| 2 \| \| Hearing impairment \| 1 \| 2 \| \| Visual impairment \| 1 \| 2 \| \| Speech impairment such as speech production, language comprehension \| 1 \| 2 \| |  |
|  | Does anyone help you with these difficulties you mentioned above | | Yes ……………1  No …………...2 |  |
|  | Please tell me if you have any difficulty with these because of a physical, mental, emotional, or memory problem. Please exclude any difficulties you expect to last less than three months. Because of a health or memory problem, do you have any difficulty with…? (ADL/IADL) | | \|  \| Yes \| No \| \| --- \| --- \| --- \| \| Dressing, including putting on chappals, shoes, etc \| 1 \| 2 \| \| Walking across a room \| 1 \| 2 \| \| Bathing \| 1 \| 2 \| \| Eating, difficulties \| 1 \| 2 \| \| Getting in or out of bed \| 1 \| 2 \| \| Using the toilet, including getting up and down \| 1 \| 2 \| \| Preparing a hot meal (cooking and serving) \| 1 \| 2 \| \| Shopping for groceries \| 1 \| 2 \| \| Making telephone calls \| 1 \| 2 \| \| Taking medications \| 1 \| 2 \| \| Doing work around the house or garden \| 1 \| 2 \| \| Managing money, such as paying bills and keeping track of expenses \| 1 \| 2 \| \| Getting around or finding address in unfamiliar place \| 1 \| 2 \| |  |
|  | Are you using any aid or supportive device(s) to assist you in the activities of daily living? Examples of supportive devices include spectacles and dentures, and devices to help you in moving or sitting. | | Yes ……………1  No …………...2 |  |
|  | Here are a few aids/instrument listed. Please tell me which of the following device(s), you have been using to assist you in the activities of daily living? | | \|  \| Yes \| No \| \| --- \| --- \| --- \| \| Hearing Aid \| 1 \| 2 \| \| Spectacles/contact lenses \| 1 \| 2 \| \| Denture \| 1 \| 2 \| \| Walker/ walking Sticks \| 1 \| 2 \| \| Wheel chairs \| 1 \| 2 \| \| Adjustable shower stools /Commodes \| 1 \| 2 \| \| Back/ neck collar \| 1 \| 2 \| \| Orthesis and prosthesis \| 1 \| 2 \| \| Others  (Specify_______________) \| 1 \| 2 \| |  |
| **Section 5: Health Care Access & Utilization** | | | | |
|  | Are you covered by health insurance? | | Yes ……………1  No …………...2 | If ‘no’ then ask 502 & if yes ask 503 |
|  | What is the main reason for not having health insurance? | | \|  \| Yes \| No \| \| --- \| --- \| --- \| \| I am not aware about health insurance \| 1 \| 2 \| \| I cannot afford it \| 1 \| 2 \| \| I do not need it \| 1 \| 2 \| \| I do not know where to purchase it \| 1 \| 2 \| \| I tried to get health insurance but was denied it \| 1 \| 2 \| \| My family decided not to purchase it \| 1 \| 2 \| \| Other,  (specify _______________) \| 1 \| 2 \| |  |
|  | What types of health insurance are you covered by? [Multiple answers are allowed] | | \|  \| Yes \| No \| If YES Specify \| \| --- \| --- \| --- \| --- \| \| Central Government Health Scheme (CGHS) \| 1 \| 2 \|  \| \| Employees State Insurance Scheme (ESIS) \| 1 \| 2 \| \| Rashtriya Swasthya Bima Yojana (RSBY) \| 1 \| 2 \| \| Biju Swasthya Kalyan Yojana (BSKY) \| 1 \| 2 \| \| Biju Krushak Kalyan Yojana (BKKY) \| 1 \| 2 \| \| Odisha State Treatment Fund (OSTF) \| 1 \| 2 \| \| Rashtriya Swasthya Bima Yojana (RSBY)/ PMSBY \| 1 \| 2 \| \| Medical reimbursement from an employer \| 1 \| 2 \| \| Other Central government health insurance schemes \| 1 \| 2 \|  \| \| State health government health insurance schemes \| 1 \| 2 \|  \| \| Community/cooperative health insurance schemes \| 1 \| 2 \|  \| \| Health insurance through an employer \| 1 \| 2 \|  \| \| Privately purchased commercial health insurance \| 1 \| 2 \|  \| \| Others  (Specify ______________) \| 1 \| 2 \|  \| |  |
|  | What does this health insurance cover? [Multiple answers are allowed] | | \|  \| Yes \| No \| \| --- \| --- \| --- \| \| Surgery \| 1 \| 2 \| \| Tests ( e.g. X-Rays, MRI, CT scan, lab tests) \| 1 \| 2 \| \| Doctor visits \| 1 \| 2 \| \| Medicines \| 1 \| 2 \| \| Dental care \| 1 \| 2 \| \| In-home care \| 1 \| 2 \| \| Hospitalization charges \| 1 \| 2 \| \| Others,  (Specify ____________________) \| 1 \| 2 \| |  |
|  | In the past 12 months, have you visited any health care facility or any health professional has visited you? | | **Public facility:**  Health post/sub centers……………………….11  Primary health center/Urban Health Center…..12  Community health center……………………..13  District / Sub-district hospital…………………14  Government/tertiary hospital………………….15  Govt. AYUSH hospital………………………..16  **Private facility:**  Private hospital/nursing home………………….21  Private clinic (OPD based services)…………….22  NGO/Charity/Trust/Church-run hospital ……….23  Private AYUSH hospital ………………………..24  **Others:**  Health camp ………………………………..31  Mobile healthcare unit ……………………..32  Pharmacy/drugstore ………………………..33  Home visit ………………………………….34  None ………………………………………..35  Others……………………………………….36  (specify____________________) |  |
|  | In the past 12 months, have you consulted any health care provider? | | Doctor (with MBBS, including surgeon, physician, gynecologist, psychiatrist, ophthalmologist and orthopedician)…………………………………1  AYUSH practitioner (Ayurveda /unani/ siddha /homeopathy) …………………………………2  Dentist ………………………………………...3  Nurse/midwife ………………………………..4  Physiotherapist ……………………………….5  Pharmacist ……………………………………6  Traditional /Folk healers (tribal medicine/bhopa/jhaad-fook/black magic) ……7  None ………………………………………...8  Others………………………………………..9  (specify _______________________) |  |
|  | What were the reasons of your last visit to the healthcare facility? [Multiple answers are allowed] | | \|  \| Yes \| No \| \| --- \| --- \| --- \| \| Preventive checkup \| 1 \| 2 \| \| Regular treatment/checkup/routine follow-up visit Sickness \| 1 \| 2 \| \| Injury/Violence \| 1 \| 2 \| \| Other,  (specify _______________) \| 1 \| 2 \| |  |
|  | [Ask this question if Q.505 and Q. 506 is ‘none  What was your main reason for not seeking a visit? | | \|  \| Yes \| No \| \| --- \| --- \| --- \| \| Did not get sick \| 1 \| 2 \| \| Needed to work \| 1 \| 2 \| \| Didn’t want to give up a day’s work \| 1 \| 2 \| \| Not enough money or cost was too high \| 1 \| 2 \| \| Treatment was unlikely to be effective \| 1 \| 2 \| \| Illness was not serious \| 1 \| 2 \| \| Nobody to accompany \| 1 \| 2 \| \| No quality facilities available nearby \| 1 \| 2 \| \| Had medicine at home \| 1 \| 2 \| \| Family member(s) decided it wasn’t required \| 1 \| 2 \| \| No healthcare facility nearby \| 1 \| 2 \| \| Other,  (Specify ______________________) \| 1 \| 2 \| |  |
|  | Over the last 12 months, how many times you were admitted as patient to a hospital/long-term care facility for at least one night? | | Number of times: ______ | If ‘0’ times then skip to section 6 |
|  | How many nights have you spent in the hospital during the past 12 months? | | Number of nights: ____ |  |
|  | For the last hospitalization, how many months ago were you admitted to the hospital? | | Months ago ____ |  |
|  | Which type of facility did you visit during your last hospitalization? | | Government hospital …………………………….1  Private hospital /Nursing home ………………….2  NGO/Charity/Trust/Church-run hospital ………..3  Private (partial) and /Government (partial)/NGO (partial) …………………………………………..4  Others ……………………………………………5  (specify __________________) |  |
|  | How many nights did you spend in the hospital during your last hospitalization? | | Number of nights ____ |  |
|  | Why were you hospitalized? | | Sickness ……………………………..1  Injury/accident ………………………2  Violence ……………………………..3  Others…………………………………4  (specify ___________________) |  |
|  | What is the main reason of your last hospitalization? | | \|  \| Yes \| No \| \| --- \| --- \| --- \| \| Cancer \| 1 \| 2 \| \| Chronic pain in your joints/arthritis/rheumatism/osteoporosis (joints, back, neck) \| 1 \| 2 \| \| Dengue or other vector-born disease (Chikungunya, Filariasis ) \| 1 \| 2 \| \| Depression or anxiety/tension/sleep problem \| 1 \| 2 \| \| Diabetes or related complications \| 1 \| 2 \| \| Fever/Pyrexia of unknown reason \| 1 \| 2 \| \| Fracture/Muscle rupture \| 1 \| 2 \| \| Gastroenteritis or other diarrheal illness \| 1 \| 2 \| \| High blood pressure (hypertension) \| 1 \| 2 \| \| HIV/AIDS \| 1 \| 2 \| \| Injury/accident (non-occupational) \| 1 \| 2 \| \| Liver diseases (hepatitis, alcoholic liver disease, cirrhosis) \| 1 \| 2 \| \| Liver diseases (hepatitis, alcoholic liver disease, cirrhosis) \| 1 \| 2 \| \| Malaria \| 1 \| 2 \| \| Maternal or Prenatal Conditions (pregnancy-related problem or gynecological problems) \| 1 \| 2 \| \| Occupation/work-related accident/injury \| 1 \| 2 \| \| Other acute/chronic communicable diseases \| 1 \| 2 \| \| Problems with your breathing (asthma/chronic obstructive pulmonary disease [COPD]) \| 1 \| 2 \| \| Problems with your heart, including unexplained pain in chest (angina, myocardial infarction [M.I.], heart-related surgery) \| 1 \| 2 \| \| Stroke/sudden paralysis of one side of body \| 1 \| 2 \| \| Surgery for abdominal causes (appendix, hernia, gall bladder, kidney) \| 1 \| 2 \| \| Surgery for genitourinary (prostate, piles, incontinence) \| 1 \| 2 \| \| Surgery for ophthalmic cause (cataract, glaucoma, retina, cornea) \| 1 \| 2 \| \| Surgery for other causes \| 1 \| 2 \| \| Tuberculosis \| 1 \| 2 \| \| Upper Respiratory Tract Infection (URTI/URI) or Lower Respiratory Tract Infection (LRTI) \| 1 \| 2 \| \| Urinary Tract Infection (UTI) / Reproductive Tract Infection (RTI) \| 1 \| 2 \| \| Other  (specify ____________) \| 1 \| 2 \| |  |
|  | During your last hospitalization, what kind of treatment/services did you receive? | | \|  \| Yes \| No \| \| --- \| --- \| --- \| \| Medical check-up (under observation/routine checkup) \| 1 \| 2 \| \| Injection & IV (drip infusion) \| 1 \| 2 \| \| Laboratory test (Blood/Urine/Stool/Sputum/Saliva) \| 1 \| 2 \| \| Endoscopy or colonoscopy \| 1 \| 2 \| \| Surgery \| 1 \| 2 \| \| X-ray, CT scan, B ultrasonic, MRI \| 1 \| 2 \| \| Medications (allopathic) \| 1 \| 2 \| \| Medications (AYUSH) \| 1 \| 2 \| \| Traditional treatment (massage, acupuncture) \| 1 \| 2 \| \| Other,  please specify ____________ \| 1 \| 2 \| |  |
|  | What was the total amount that you or your household spent on this visit? | | Total expenditure |  |
|  | What was your health status when you left the hospital? | | Recovered from illness, received doctor’s approval…………………………………………….1  Did not recover from illness, but received doctor’s approval……………………………………………..2  Did not recover from illness, requested to leave without doctor’s approval ………………………….3  Patient’s condition can be managed on OPD……….4  Other………………………………………………...5  (Specify____________________) | Ask q519 if q518 is 3 |
|  | Why did you want to leave the hospital before you were recovered? [Multiple answers are allowed] | | \|  \| Yes \| No \| \| --- \| --- \| --- \| \| Couldn’t recover from illness \| 1 \| 2 \| \| Ran out of money; couldn’t afford anymore \| 1 \| 2 \| \| Pushed out; no space in the hospital \| 1 \| 2 \| \| Poor quality and service from health care providers \| 1 \| 2 \| \| Other,  (Specify ______________________) \| 1 \| 2 \| |  |
|  | Do you still suffer from the ailment you originally sought treatment for? | | Yes ……………1  No …………...2 |  |
|  | Did your treatment continue after discharge? | | Yes ……………1  No …………...2 |  |
|  | How long was the duration of treatment after discharge? | | Days completed _____  Days to be continued ____  Continuing for lifelong………………….88888 |  |
|  | Are you able to receive care from the same place, same doctor or same healthcare facility when you need healthcare services? | | Yes ……………..1  No………………2 |  |
|  | If yes, When you are sick or in need of healthcare services, where do you usually go? | | Public-primary ……………..1  Public- specialized …………2  Private ……………………...3  AYUSH ……………………4  Others ………………………5  (Specify ___________________) |  |
|  | **Section 6: Quality of life** | | |  |
|  | Mobility | | I have no problems in walking about …………1  I have slight problems in walking about ………2  I have moderate problems in walking about …..3  I have severe problems in walking about ………4  I am unable to walk about ……………………5 |  |
|  | Pain/discomfort | | I have no pain or discomfort …………………1  I have slight pain or discomfort ………………2  I have moderate pain or discomfort ……………3  I have severe pain or discomfort ………………4  I have extreme pain or discomfort ……………5 |  |
|  | Anxiety/ Depression | | I am not anxious or depressed …………………1  I am slightly anxious or depressed ……………2  I am moderately anxious or depressed …………3  I am severely anxious or depressed ……………4  I am extremely anxious or depressed …………5 |  |
|  | Self-Care | | I have no problems washing or dressing myself .1  I have slight problems washing or dressing myself……..2  I have moderate problems washing or dressing myself…….3  I have severe problems washing or dressing myself ……4  I am unable to wash or dress myself ………..5 |  |
|  | Unusual Activities (work, study, housework, family or leisure activities) | | I have no problems doing my usual activities …1  I have slight problems doing my usual activities.2  I have moderate problems doing my usual activities..3  I have severe problems doing my usual activities …….4  I am unable to do my usual activities …………5 |  |
| **Section 7: Symptomatic Profile** | | | | |
|  | Has your blood pressure ever been checked prior to this survey? | | Yes ……………………………….1  No ………………………………..2 | If ‘yes’ ask 702-704 |
|  | When did you check your blood pressure last? | | Month:  Year: |  |
|  | Have you been informed by any Doctor/Nurse/ANM that you have high/low BP? | | Yes ……………………………….1  No ………………………………..2 |  |
|  | Are you taking any medicine for BP? | | Yes ……………………………….1  No ………………………………..2 |  |
|  | Have you ever been tested for anaemia? | | Yes ……………………………….1  No ………………………2 | If ‘yes’ ask 706 |
|  | If found “positive” what advice was given by doctor/nurse/ANM? | | Medical advice …………1  Dietary advice …………2  Both ……………………3 |  |
|  | Have you ever been tested for blood sugar | | Yes ……………………………….1  No ………………………………..2 | If ‘yes’ ask 708-710 |
|  | If “yes” when blood sugar was checked last time? | | Month:  Year: |  |
|  | What was the result of blood sugar test? | | Low ……………………………..1  Normal …………………………2  High ……………………………...3 |  |
|  | If the answer is “high” what advice was given by doctor/nurse/ANM? | | Medical advice …………1  Dietary advice …………2  Both ……………………3 |  |
|  | Do you have any of the following symptoms? (In last one month)   \| Fever \| Yes \| No \| \| --- \| --- \| --- \| \| Vomiting \| 1 \| 2 \| \| Nausea \| 1 \| 2 \| \| Pain \|  \|  \| \| 1. Joint \| 1 \| 2 \| \| 1. Hands \| 1 \| 2 \| \| 1. Feet \| 1 \| 2 \| \| 1. Muscle \| 1 \| 2 \| \| Body ache \| 1 \| 2 \| \| Headache \| 1 \| 2 \| \| Stomachache \| 1 \| 2 \| \| Anemia \| 1 \| 2 \| \| Swelling Hands \| 1 \| 2 \| \| Swelling Feet \| 1 \| 2 \| \| Dark Urine \| 1 \| 2 \| \| Yellowing of the eyes and skin \| 1 \| 2 \| \| Jaundice \| 1 \| 2 \| \| Tiredness \| 1 \| 2 \| \| Loss of appetite \| 1 \| 2 \| \| Loss of sleep \| 1 \| 2 \| \| Excessive thirst \| 1 \| 2 \| \| Blood in stool \| 1 \| 2 \| \| Swelling in abdomen \| 1 \| 2 \| \| Abdominal pain \| 1 \| 2 \| \| Diarrhea \| 1 \| 2 \| | | |  |
| **Section 8: Biomarker** | | | | |
|  | Weight in Kilogram | | \| Kg: \|  \|  \|  \| . \|  \|  \| \| --- \| --- \| --- \| --- \| --- \| --- \| --- \|   Not present………….994  Refused………………..995  Other……………………996 |  |
|  | Height in Centimeters | | \| Cm: \|  \|  \|  \| . \|  \|  \| \| --- \| --- \| --- \| --- \| --- \| --- \| --- \|   Not present………….994  Refused………………..995  Other……………………996 |  |
|  | Waist circumference in centimeters | | \| Cm: \|  \|  \|  \| . \|  \|  \| \| --- \| --- \| --- \| --- \| --- \| --- \| --- \|   Not present………….994  Refused………………..995  Other……………………996 |  |
|  | Hip circumference in centimeters | | \| Cm: \|  \|  \|  \| . \|  \|  \| \| --- \| --- \| --- \| --- \| --- \| --- \| --- \|   Not present………….994  Refused………………..995  Other……………………996 |  |
|  | Isometric hand grip strength in Kilogram | | \| Kg: \|  \|  \|  \| . \|  \|  \| \| --- \| --- \| --- \| --- \| --- \| --- \| --- \|   Not present………….994  Refused………………..995  Other……………………996 |  |
|  | The systolic and diastolic pressure for the BP reading   \|  \| Measurement1 \| Measurement2 \| Average \| \| --- \| --- \| --- \| --- \| \| Systolic \|  \|  \|  \| \| Diastolic \|  \|  \|  \|   Refused ………………………………….994  Technical problems ………………..995  Other………………………………………996 | | |  |
|  | When you have taken food last? | | 1 hour …………………1  1 to 2 hours ……………2  More than 2 hours …….3 |  |
|  | Record blood glucose in MG/DL | | \| mg/dl: \|  \|  \|  \| \| --- \| --- \| --- \| --- \|   Refused …………………………….995  Other…………………………………996  Not tested…………………………997 |  |
|  | Record haemoglobin level | | \| g/dl: \|  \|  \|  \| . \|  \| \| --- \| --- \| --- \| --- \| --- \| --- \|   Refused …………………………….995  Other…………………………………996  Not tested…………………………997 |  |
|  | Record the SCD/SCT percentage (%) here | | \| Haemoglobin A2/C/E \| % \| \| --- \| --- \| \| Haemoglobin S \| % \| \| Haemoglobin F \| % \| \| Haemoglobin A \| % \|   Refused …………………………….995  Other…………………………………996  Not tested…………………………997 |  |
| **Section 9: Rating of health** | | | | |
| 901 | We would like to know how good or bad your health is TODAY.  This scale is numbered from 0 to 100  100 means the best health you can imagine. 0 means the worst health you can imagine.  Mark an X on the scale to indicate how your health is TODAY  Now, please write the number you marked on the scale in the box below. | | Your Health Today = |  |

**************************************END************************************************
