# Supplementary material for: Odisha tribal family health survey: methods, tools, and protocols for a comprehensive health assessment survey
Source: Front Public Health. 2023 Jul 10;11:1157241. doi: 10.3389/fpubh.2023.1157241 (PMC10364047; doi:10.3389/fpubh.2023.1157241)
Supplement: Supplementary file 2 [file Table_2.DOCX]

**OTFHS**

**ICMR- Regional Medical Research centre, Bhubaneswar**

**OTFHS**

**Manual of Procedures**


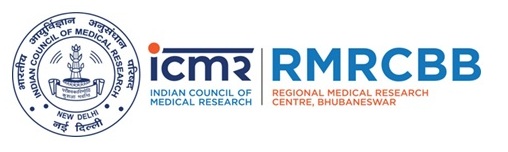


**Contents**

[**Introduction** 3](#_Toc107476510)

[**Survey Design** 4](#_Toc107476511)

[**Stratification** 5](#_Toc107476512)

[**Sample size** 5](#_Toc107476513)

[**Household selection** 10](#_Toc107476517)

[**Household listing** 11](#_Toc107476518)

[**Monitoring** 14](#_Toc107476522)

[**Field Monitoring** 16](#_Toc107476528)

[**Internal monitoring team** 19](#_Toc107476529)

[**External monitoring team** 23](#_Toc107476532)

[**Data Quality Monitoring** 25](#_Toc107476533)

[**Monitoring – Laboratory Practices in the field** 33](#_Toc107476535)

**[Anthropometric Measurement & Biomarker tests](#_Toc107476536)** [36](#_Toc107476536)

**[Blood Sample Collection in Field](#_Toc107476569)** [55](#_Toc107476569)

[**Biomedical Waste Management** 87](#_Toc107476620)

[**MOP TRAVEL** 96](#_Toc107476632)

[**MOP ADMINISTRATION** 97](#_Toc107476633)

[**MOP ROLES & RESPONSIBILITIES OF STAFFS** 100](#_Toc107476634)

[**MOP ROLE AND FUNCTION OF SCSTRTI** 105](#_Toc107476635)

[**MOP LOCAL LIAISON** 106](#_Toc107476636)

[**ORGANOGRAM & HR** 107](#_Toc107476637)

[**Sexual Harassment** 108](#_Toc107476638)

[**Annexures** 108](#_Toc107476640)

# **Introduction**

Odisha Tribal Family Health survey is the first state-level health survey which is focused on the comprehensive evaluation of the health, demographic, and socio-economic parameters of the tribal population in Odisha, India.

The survey will consider indicators related to demography, socio-economic status, maternal health, child health, adolescent health, adult health, and elderly health. The survey will focus on comparing the health status between all the 62 Scheduled Tribes and 13 PVTGs in Odisha, which is the first of its kind. The OTFHS is a state project and is implemented across various tribal sub-plan areas of Odisha. In order to achieve comparability, consistency and the best quality in survey results, sampling activities in the OTFHS is guided by number of sampling principles.

This manual present general guidelines on sampling, monitoring, laboratory practices and administrative processes for OTFHS survey.

**Survey objectives**

1. To describe and compare the health status of tribal groups in Odisha
2. To estimate the prevalence of crucial maternal-child health indicators and chronic diseases among the tribal groups in Odisha
3. To assess the demographic and socio-economic status of the tribal communities in Odisha

The fundamental principles of OTFHS sampling include:

1. Use of an existing sampling frame
2. Complete coverage of the target population
3. Multistage sampling
4. Probability sampling
5. Using a suitable sample size
6. Using the simplest design possible
7. Conducting a household listing and pre-selection of households
8. Providing good sample documentation
9. Maintaining confidentiality of individual's information
10. Implementing the sampling strategy precisely as designed

**Sampling frame**

The sampling frame used in OTFHS is the list of Enumeration Areas (EAs) from a recently completed population census (2011). An EA is usually a geographic area which groups a number of households together for convenient counting purposes for the census. The list in most cases, a list of EAs from a recent census is available. The sampling frame used for OTFHS should be as up to date as possible. The details of Scheduled tribes and PVTGs is evaluated from the census data (Annexure-1). The district wise tribal data with the details of the Tribal population in each village will be collected from the State and district offices.


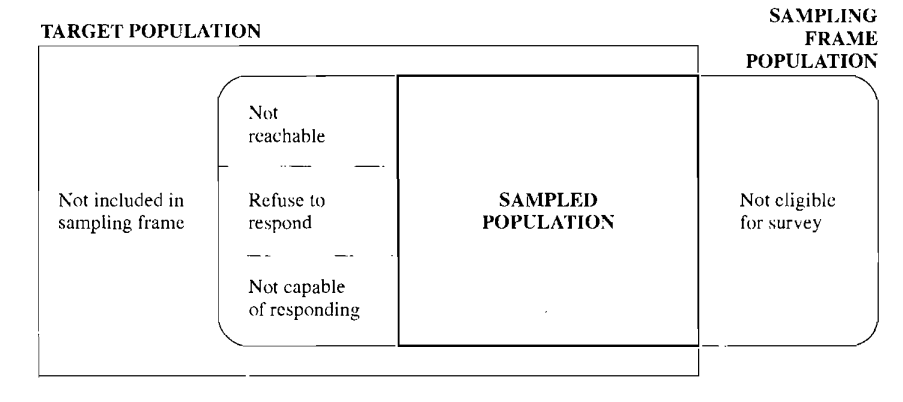


# **Survey Design**

OTFHS is a population based cross sectional survey which will be carried out in Odisha. Odisha has 30 administrative districts with population of 4.37 crores in which schedule tribe population is around 8145081 (22.5%). From these 30 administrative districts 13 districts were selected which have Tribal Sub Plan areas in them. The Tribal Sub-plan (TSP) areas include Tribal Sub-Plan blocks or tehsils with 50 percent or more tribal population. This planning concept was introduced in 5^th^ Five Year Plan (1974-1979). The study setting was the Tribal Sub-Plan (TSP) blocks of Odisha state which were under the 13 districts. Total 314 numbers of blocks were present in the 13 districts in which we have included 119 blocks. We have included all the tribal community (62 STs and 13 PVTGs). The primary sampling units were clusters and the secondary sampling units were the Households.

# **Stratification**

Stratification is the process by which the survey population is divided into subgroups or strata that are as homogeneous as possible using certain criteria. The survey population is stratified into different Tribes and PVTGs. The stratification is introduced at the first stage of sampling.

# **Sample size**

The calculations for OTFHS are based on the expected proportion of high/very high blood sugar (>140 mg/dl) = 13.3% [NFHS-5]. The sample size was calculated based on the following formula

***n* = [DEFF*Np(1-p)]/ [(d^2^/Z^2^_1-α/2_*(N-1)+p*(1-p)] * Finite Population Adjustment**

where:

- N = Total tribe population
- p = expected proportion of high/ very high blood sugar
- d = Relative precision of 30%
- CI=95%;
- α=0.05;
- β=0.2;
- ICC=0.0191 (Bischops, 2020);
- For smaller tribes where the sample size needed was greater than 5% of the entire tribal population, a finite population correction (FPC) was used. Formula used for FPC was $\sqrt{(N-n)/(N-1)}$
- The tribes were classified into 3 categories based on the population of the tribe as below:
  - Category-1 Less than 10000
  - Category-2 10000 to 100000
  - Category-3 More than 100000

Assuming a uniform Cluster size of 30, design effect was estimated for each of the above-mentioned tribe categories as below:

- - Category-1 🡪1.2
  - Category-2 🡪 1.4
  - Category-3 🡪 2.8
- Response rate of 90%;
- Avg. HH size=4

The necessary sample size for individuals were calculated and then the number was extrapolated to the number of households. Back calculation of power for aggregate analyses were used.


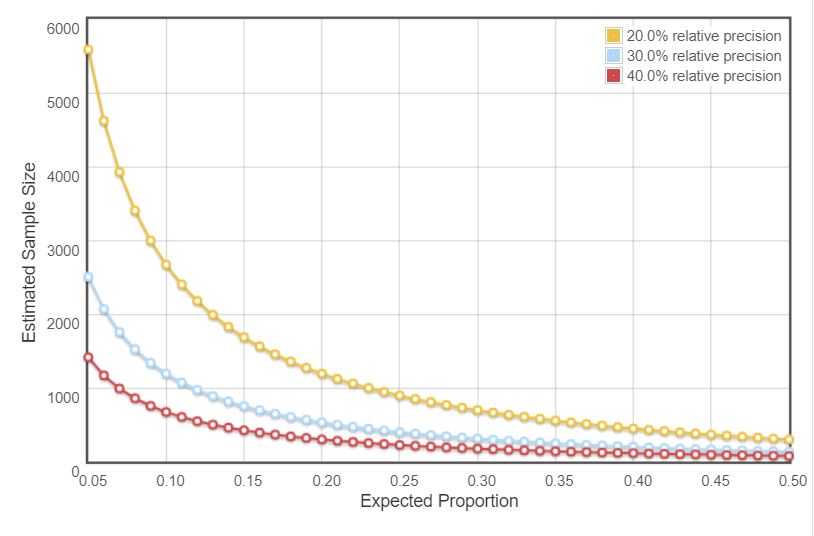


The calculated individual specific sample size is given in Annexure- 1, by assumed non-response rate as 90% the total Sample size calculated for the survey was 42048 from 10489 households. The number of Households will be sampled from 350 clusters across the Tribal sub plan areas in the state covering 62 tribes and 13 PVTGs.

## **Sampling design**

The survey uses multistage sampling design in which 13 districts of Tribal Sub Plan areas were taken. The sampling strategy used is represented in the flow chart

## **Selection of districts and blocks**

In the survey we have selected all the districts listed in the Tribal- Sub Plan (TSP) areas of Odisha. We have included all the Tribal sub plan blocks (119) under each district

## **Cluster selection**

In the first stage of sampling, a stratified sample of Tribal population is selected from different districts through proportionate allocation. In each stratum number of clusters is predetermined and then the number of clusters in each district is calculated based on the proportion allocation of each tribe in the district. Purposive sampling method uses of smaller tribes living in small geographic areas. Total number of clusters is then finally calculated for each district.

| **Tribes** | **Districts** | | | | | | | **Total** |
| --- | --- | --- | --- | --- | --- | --- | --- | --- |
|  | **District-1** | **District-2** | **District-3** | **District-4** | **District-5** | **District-6** | **District-n** |  |
| **Tribe-1** |  |  |  |  |  |  |  |  |
| **Tribe-2** |  |  |  |  |  |  |  |  |
| **Tribe-3** |  |  |  |  |  |  |  |  |
| **Tribe-4** |  |  |  |  |  |  |  |  |
| **Tribe-n** |  |  |  |  |  |  |  |  |
| **Total** |  |  |  |  |  |  |  |  |

The clusters from each districts are selected by probability proportional to size. The cluster selection was carried out in the state office. Scheduled tribes and PVTGs having less sample size and concentrated in limited geographical area were purposively selected.

Required number of clusters from each district

| **Sl.no** | **Name of district** | **Required no. of clusters** |
| --- | --- | --- |
| **1** | Sambalpur | 25 |
| **2** | Debagarh | 11 |
| **3** | Sundargarh | 57 |
| **4** | Kendujhar | 39 |
| **5** | Mayurbhanj | 56 |
| **6** | Balasore | 21 |
| **7** | Gajapati | 10 |
| **8** | Kandhamal | 7 |
| **9** | Kalahandi | 21 |
| **10** | Rayagada | 15 |
| **11** | Nabarangpur | 29 |
| **12** | Koraput | 41 |
| **13** | Malkangiri | 40 |
|  | Total | 372 |

# **Household selection**

In the second stage, a complete household listing is conducted in each of the selected clusters, a fixed number of households is selected by equal probability systematic sampling in the selected clusters. In each selected household, a household questionnaire is completed to identify all eligible study participants in the household. Every eligible study participant will be interviewed with individual questionnaire in those households, and one samples will be collected from each household based on Age-order matrix. The Household selection will be carried out in the field.

# **Household listing**

A household consists of a person or a group of related or unrelated persons, who live together in the same dwelling unit, who acknowledge one adult male or female 15 years old or older as the head of the household, who share the same housekeeping arrangements, and are considered as one unit. In some cases one may find a group of people living together in the same house, but each person has separate eating arrangements; they should be counted as separate one-person households. Collective living arrangements such as army camps, boarding schools, or prisons will not be considered as households. Examples of households are:

- a man with his wife or his wives with or without children
- a man with his wife or his wives, his children and his parents
- a man with his wife or his wives, his married children living together for some social or economic reasons (the group recognize one person as household head)
- a widowed or divorced man or woman with or without children

The head of household is the person who is acknowledged as such by members of the household and who is usually responsible for the upkeep and maintenance of the household.

## **Listing procedure**

When a cluster is selected for the survey, a complete list of households in the selected cluster is conducted prior to the selection of households. The listing operation consists of visiting the selected cluster, collecting geographic coordinates of the cluster and collecting number of households in the cluster. This information is necessary for an equal probability random selection of households in the second stage. With the household listing prior to the main survey, it is possible to pre-select the sample households in advance and the interviewers are asked to interview only the pre-selected households without replacement of non-responding households. The precise location information (GIS) for selected clusters will be collected by the geographic positioning systems (GPS) .

## **Selection of household**

Systematic random sampling is the selection of sampling units at a fixed interval from a list, starting from a randomly determined point. Selection is systematic because selection of the first sampling unit determines the selection of the remaining sampling units.

Systematic sampling is normally carried out as follows: assuming a whole number interval k=N/n, where N is the number of units in the list and n is the number of units to be selected. The procedure begins with an integer random number S that is less than or equal to k. The units to be selected are S, S+k, S+2*k, ..., S+(n-1)*k. When k is not a whole number there may be appreciable errors in rounding it to the nearest whole number, it is suggested that the decimal interval method be used. Selection with a decimal interval may be carried out as follows:

1) Calculate the interval I rounded to two decimal places.

2) Generate a random number R between 0 and 1 with two decimal points.

3) Compute the sequence of sampling numbers: R*k, R*k + k, R*k + 2*k, ..., R*k + (n - 1)*k

4) Round up the above calculated sampling numbers to the next highest whole numbers; these are the selected units' numbers.

The sampling interval will be calculated as k = Total households/ Number of required households. The first household will be selected through random number generator/random number table by selecting a random number between 1 and k.

If the sampled household is not selected because of the following reason:

- - - Household is locked
    - Household has migrated
    - Household is damaged and no one is living
    - ST community not occupying the household

Then the adjacent household will be taken, and the procedure will be carried out until next household is selected.

## **Responsibilities of staff**

Each team consist of four members-

Project Assistant-1

Project Technician (Field Investigator)-1

Project Technician (Laboratory technician)-2

The Project Assistant (RA) will monitor the entire operation. The responsibilities of the PA are to:

- obtain number of households for all the clusters included in the survey;
- keep all the materials (manuals, letters)
- obtain one GIS location for each cluster
- list all the households in the cluster in a systematic manner and select the households through SRS
- communicate to the local health staff/authorities regarding the field visit
- Communicate to the village head/key persons in the village before the field visit.
- Communicate to the main office regarding any issues in the field
- The sampling in each cluster will be carried out by the PA of the team. If PA is not available, then the sampling will be carried out by the Project technician (Field Investigator)
- The following forms will be completed by the PA of the team while in the field
  - - 1. Household attempt form
      2. Household listing form

Household attempt form- In the form the details of attempt made in the cluster will be documented. In general, the team will visit a cluster for 3 days (approx.), so for data and sample collection number of attempts made will be documented in this form.

Household listing form- In this form details regarding the household with individual ID and sample ID will be collected. The details of the individual will also be collected in the form which includes the name, age and gender of the study participants.

The form will be filled by the PA during the data collection. In case PA of the team is not available, the FI will fill the respective form.

The Field Investigator will collect the data and will complete the consent form prior to data collection. He will make sure the consent forms are properly filled and the signature/thumb print are present in the consent form. Following forms will be completed by the FI of the team

1. Consent form
2. Travel logbook

Travel logbook form- The travel logbook is the details regarding daily travel in the field. In case the FI is not present the LT will fill the form.

The LT of the team will collect the sample of the study participants who will provide their consent. He will be responsible for maintaining the following forms

- - - 1. Stock logbook
      2. Sample logbook

Stock logbook- The stock logbook contains the details of consumables that will be used in the field. The list will be maintained by the field team LT.

Sample logbook- The sample logbook is details regarding the Samples collected. The sample logbook will be completed and will be sent to the main office with the samples.

# **Monitoring**

A well-functioning monitoring, evaluation and learning system is an important part of the survey for management and accountability. A structured, timely and reliable monitoring and evaluation will provide information to:

1. Support the survey with accurate, evidence-based reporting that informs management and decision-making to guide and improve performance
2. Contribute to organizational learning and knowledge sharing by reflecting upon and sharing experiences and lessons so that benefit can be derived from what is being done and how it is being done.
3. Ensure accountability and compliance by demonstrating whether the work is being carried out as agreed, and in compliance with established standards of ICMR and in line with donor requirements.
4. Promote and recognize accomplishments and achievements, building morale and contributing to resource mobilization.

## **Goal and Objectives**

The monitoring and evaluation (M&E) strategy is key drivers for Odisha Tribal Family Health Survey (OTFHS) which acknowledges the necessity of developing a functional and robust M&E system for OTFHS to provide useful and timely information to program managers, management committee and funding agency. The program managers will track progress of the Survey to ensure achievements of results through necessary course corrections during implementation of the survey.

## **Goal**

The goal is to establish a sustainable M&E system for tracking progress and demonstrating results of the Survey and to ensure evidence-based practice.

## **Specific objectives**

The specific objectives of the M&E Strategy are to:

1) Improve the quality of the routine data collection systems, e.g., development of registries, routine data collection forms, type, and frequency of reports, etc.

2) Outline specific activities required for strengthening the organizational capacity to conduct effective M&E

## **Key outputs**

The expected key outputs of the M&E Strategy are:

1) Prepare an M&E Action Plan for the survey

2) A functional robust, comprehensive, and well-coordinated M&E system for OTFHS in place; 3) Regular updates on performance indicators available.

4) Project/Survey progress reports are produced on time.

## **Outcomes**

The M&E Strategy is expected to result in:

1) Promoting the practice of evidence-based research

2) Reporting in time to ICMR-RMRC management committee and funding partners

3) Objective decision making for performance improvement; planning and resource allocation; and Promoting accountability of ICMR-RMRCBB.

# **Field Monitoring**

The field Monitoring in built in mechanism in the survey which will be continuous throughout the project. Each survey team in the field is consisted of Project Assistant (Field Supervisor), Field Investigator (FI) and Laboratory technicians (LT). All the staff has their individual role in their team and the field day to day activities will be monitored by the Project Assistant (PA) of the team. The following are the important roles of RA

1. RA are the primary links between the main office and field work, so they are responsible for ensuring both the quality and progress of fieldwork.
2. Monitoring interviewer performance by-
   1. observe at least one interview every day
   2. edit all completed questionnaires in the field; editing must be completed prior to leaving the sample area
   3. conduct regular review sessions with interviewers and advise them of any problems found in their questionnaires.
   4. uploading of the data
3. Daily data quality checks
   1. Data collected is reviewed daily
   2. Data uploaded on the server daily
4. Maintaining logistics
   1. Consent related all forms
   2. Biomedical related logistics
   3. Office stationery
5. Monitoring field expenses
6. Maintaining vehicle logbook

Field check list which will be maintained by the PA of the team.

Supervisor

1. Attendance sheet
2. Cluster list
3. Household listing
4. Travel Log book
5. Sample Log book
6. Manuals
7. Tribe list
8. Stock register
9. Attempt register
10. Completed schedules

Attendance sheet

| Si No. | Name | 1 | 2 | 3 | 4 | 5 | 6 | 7 | 8 | 9 | 10 | 11 | 12 | 13 | 14 | 15 | 16 | 17 | 18 | 19 | 20 | 21 | 22 | 23 | 24 | 25 | 26 | 27 | 28 | 29 | 30 | 31 |
| --- | --- | --- | --- | --- | --- | --- | --- | --- | --- | --- | --- | --- | --- | --- | --- | --- | --- | --- | --- | --- | --- | --- | --- | --- | --- | --- | --- | --- | --- | --- | --- | --- |
| 1 |  |  |  |  |  |  |  |  |  |  |  |  |  |  |  |  |  |  |  |  |  |  |  |  |  |  |  |  |  |  |  |  |
| 2 |  |  |  |  |  |  |  |  |  |  |  |  |  |  |  |  |  |  |  |  |  |  |  |  |  |  |  |  |  |  |  |  |
| 3 |  |  |  |  |  |  |  |  |  |  |  |  |  |  |  |  |  |  |  |  |  |  |  |  |  |  |  |  |  |  |  |  |
| 4 |  |  |  |  |  |  |  |  |  |  |  |  |  |  |  |  |  |  |  |  |  |  |  |  |  |  |  |  |  |  |  |  |
|  |  |  |  |  |  |  |  |  |  |  |  |  |  |  |  |  |  |  |  |  |  |  |  |  |  |  |  |  |  |  |  |  |
| Filled by | | | | | | | | | | | | | | | | | | | | | | | | | | | | | | | | |
| Verified by- | | | | | | | | | | | | | | | | | | | | | | | | | | | | | | | | |

Household listing

| Si No. | Household Id | Individual Id | Data Collected | | | Blood sample collected | | |
| --- | --- | --- | --- | --- | --- | --- | --- | --- |
|  |  |  | 1^st^ attempt | 2^nd^ attempt | 3^rd^ attempt | 1^st^ attempt | 2^nd^ attempt | 3^rd^ attempt |
|  |  |  |  |  |  |  |  |  |
|  |  |  |  |  |  |  |  |  |
| Filled by | | | | | | | | |
| Verified by- | | | | | | | | |

Sample log book

| Si No. | Sample ID  (Barcode) | Household ID | Individual Id | Sample collected  Yes/No | Serum/plasma Collected  Yes/No |
| --- | --- | --- | --- | --- | --- |
|  |  |  |  |  |  |
|  |  |  |  |  |  |
|  |  |  |  |  |  |
| Filled by | | | | | |
| Verified by- | | | | | |

Travel Log Book

| Si No. | Date | Place | Starting time | Starting Km | Ending time | Ending km | Signature | Remark |
| --- | --- | --- | --- | --- | --- | --- | --- | --- |
|  |  |  |  |  |  |  |  |  |
|  |  |  |  |  |  |  |  |  |
|  |  |  |  |  |  |  |  |  |
| Filled by | | | | | | | | |
| Verified by- | | | | | | | | |

Tribe list (Individual Team Form)

| Si No. | Tribe Name | Team Name | Number of Households covered | Number of Samples Collected |
| --- | --- | --- | --- | --- |
|  |  |  |  |  |
|  |  |  |  |  |
|  |  |  |  |  |
|  |  |  |  |  |
|  |  |  |  |  |

The Field Investigator (FI) of the team will look after the collection of data from the field and completeness of the data before uploading the data. The following check list will be filled by the FI and the same will be verified by the PA of the team.

The Laboratory Technician will ensure the collection of samples, storage of samples and transportation of samples from field to the Sample storage unit in the district. He will also make sure all the samples were stored at optimum temperature at district office.

# **Internal monitoring team**

The internal monitoring team will be formed in the center (ICMR-RMRCBB). The internal monitoring team will be-

- Managing committee (OTFHS)
- Project Scientist (OTFHS)

## **OTFHS Management Committee (OMC)**

The Managing Committee (MC) will consist of Senior Scientists of ICMR, Administrative Officers, Account Officers, and External experts in the field. We have constituted on management committee in the Centre (RMRCBB) in which following members are part of the team-

The responsibility of the internal monitoring team (MC) will be-

- Checking the data validity and verify the completeness and adequacy of data
- Sampling protocol followed in the field
- Field interview observation
- Conducting reinterview in a subset of households (2 HH) and cross-checking with collected field data (10% of questionnaire)
- Assessment of time stamps and GIS co-ordinates
- Matching of collected data with Lab tally sheet in a subset of households (2 HH)
- Sample storage and maintenance of cold chain in the field
- Frequency- Quarterly (Every 3 months)

The team will use various checklist as provided in the annexures for monitoring the monthly update of the survey. They also monitor the following things in the field while visiting the field team

- Attendance register
- Stock register
- Household listing
- Lab tally sheet
- Sampling protocol followed
- Reinterviewing of 2 HH (10% of questionnaire)
- Target achieved (Indicator based)

Remarks and Suggestion for improvement

## **OTFHS Project Scientist Team (OPS)**

The internal team of Project Scientist will be the Co PIs for the project and Project scientist hired for the project of different domain specialization.

The responsibility of the internal monitoring team will be-

- Checking the data validity and verify the completeness and adequacy of data
- Sampling protocol followed in the field
- Field interview observation
- Conducting reinterview in a subset of households (2 HH) and cross-checking with collected field data (10% of questionnaire)
- Assessment of time stamps and GIS co-ordinates
- Matching of collected data with Lab tally sheet in a subset of households (2 HH)
- Sample storage and maintenance of cold chain in the field
- Frequency- Monthly

The team will use various checklist as provided in the annexures for monitoring the monthly update of the survey. They also monitor the following things in the field while visiting the field team

- Attendance register
- Stock register
- Household listing
- Lab tally sheet
- Sampling protocol followed
- Reinterviewing of 2 HH (10% of questionnaire)
- Target achieved (Indicator based)
- Remarks and Suggestion for improvement

'

**Tribe list cumulative**

| **Name of the tribe** | **Team A** | **Team B** | **Team C** | **Team D** | **Team E** | **Team F** | **Total** |
| --- | --- | --- | --- | --- | --- | --- | --- |
|  |  |  |  |  |  |  |  |
|  |  |  |  |  |  |  |  |

**Monitoring Checklist**

| **Si No.** | **Name** |  |  |
| --- | --- | --- | --- |
| 1 | Attendance register | Updated   1. Yes 2. No |  |
| 2 | Vehicle logbook | Updated   1. Yes 2. No |  |
| 3 | Sample logbook | Updated   1. Yes 2. No |  |
| 4 | Household tally sheet | Updated   1. Yes 2. No |  |
|  |  |  |  |

**Internal Monitoring Plan**

**Project Scientist (PS)**

|  | **Month-1** | **Month-2** | **Month-3** | **Month-4** | **Month-5** | **Month-6** |
| --- | --- | --- | --- | --- | --- | --- |
| **District Name** |  |  |  |  |  |  |
| **District-1** | **√** | **√** | **√** | **√** | **√** | **√** |
| **District-2** | **√** | **√** | **√** | **√** | **√** | **√** |
| **District-3** | **√** | **√** | **√** | **√** | **√** | **√** |
| **District-4** | **√** | **√** | **√** | **√** | **√** | **√** |
| **District-N** | **√** | **√** | **√** | **√** | **√** | **√** |

**The same will be repeated for next 6 month**

**Management Committee (MC)**

|  | **Month-1** | **Month-2** | **Month-3** | **Month-4** | **Month-5** | **Month-6** |
| --- | --- | --- | --- | --- | --- | --- |
| **District Name** |  |  |  |  |  |  |
| **District-1** | **√** |  |  | **√** |  |  |
| **District-2** | **√** |  |  | **√** |  |  |
| **District-3** | **√** |  |  | **√** |  |  |
| **District-4** | **√** |  |  | **√** |  |  |
| **District-N** | **√** |  |  | **√** |  |  |

**The same will be repeated for next 6 month**

# **External monitoring team**

The external team members will consist of-

- External experts from Medical College and Hospitals
- External experts from NHM/District Hospital

The responsibility of the internal monitoring team will be-

- Checking the data validity and verify the completeness and adequacy of data
- Sampling protocol followed in the field
- Field interview observation
- Conducting reinterview in a subset of households (2 HH) and cross-checking with collected field data
- Matching of collected data with Lab tally sheet in a subset of households (2 HH)
- Sample storage and maintenance of cold chain in the field
- Frequency- Every 3 months

The team will evaluate the project progress and following details will be documented

- Project progress based on coverage of

-Tribes

-Households

-Samples

-Logistics usage

- Samples transportation in district and state
- Field observation
- Suggestion for improvement

**External monitoring team (EM)**

|  | **Month-1** | **Month-2** | **Month-3** | **Month-4** | **Month-5** | **Month-6** |
| --- | --- | --- | --- | --- | --- | --- |
| **District Name** |  |  |  |  |  |  |
| **District-1** |  |  | **√** |  |  | **√** |
| **District-2** |  |  | **√** |  |  | **√** |
| **District-3** |  |  | **√** |  |  | **√** |
| **District-4** |  |  | **√** |  |  | **√** |
| **District-N** |  |  | **√** |  |  | **√** |

**The same will be repeated for next 6 month**

**Overall Monitoring Plan**

|  | **Month-1** | **Month-2** | **Month-3** | **Month-4** | **Month-5** | **Month-6** |
| --- | --- | --- | --- | --- | --- | --- |
| **District Name** |  |  |  |  |  |  |
| **District-1** | **(PS) (MC)** | **(PS)** | **(PS) (EM)** | **(PS) (MC)** | **(PS)** | **(PS) (EM)** |
| **District-2** | **(PS) (MC)** | **(PS)** | **(PS) (EM)** | **(PS) (MC)** | **(PS)** | **(PS) (EM)** |
| **District-3** | **(PS) (MC)** | **(PS)** | **(PS) (EM)** | **(PS) (MC)** | **(PS)** | **(PS) (EM)** |
| **District-4** | **(PS) (MC)** | **(PS)** | **(PS) (EM)** | **(PS) (MC)** | **(PS)** | **(PS) (EM)** |
| **District-N** | **(PS) (MC)** | **(PS)** | **(PS) (EM)** | **(PS) (MC)** | **(PS)** | **(PS) (EM)** |

***PS- Project Scientist, MC- Management Committee, EM- External Monitoring Team**

**The same will be repeated for next 6 month**

# **Data Quality Monitoring**

Ensuring data quality in different stages of the data collection phase and post-data collection phase. Details are given below

1. **Data collection phase - Monitoring Through DBMS**

The Data Base Management System will be used for real-time data monitoring. The Dashboard indicators will show the real-time progress regarding coverage of Districts, Clusters, Households, Samples collected, etc.

Daily Monitoring: DBMS

- Day-wise interview status including response rate
- Interviewer productivity
- Average duration of the interviews
- Negative screening rate
- Key indicators from the field check tables
- Indicators which have a high risk of error during data collection
- Outliers and extreme values in key survey indicators
- Number of missing values in key indicators
- Observations which are duplicates
- List of questions taking most of the interview time
- List of questions with most 'don’t know’ and/or ‘no answer’ responses

Different stages of data flow in the data collection phase mentioned in below diagram.

## **Steps to be followed for data entry & Monitoring**

1. To start data entry, first required to login to connect the server. Enter the given host name, username & password for login the DBMS platform.
2. Once successfully logged in, it will take to MySQL monitor and it is ready for data entry.
3. There are different databases were created according to the different questionnaires of OTFHS, such as; cluster, household, under 5 years old children, 5-9 years old children, adolescents age 10-19 years, women and men age 20-59, geriatric population of age 60 years and above and field investigation.
4. Start the data entry from the cluster module
5. Once the cluster data entry completed, the remaining databases under the cluster database will be automatically generated.
6. Second stage is the start the household data entry, as soon as household data entry completes, there will be a provision to see the individual modules and the field investigation module.
7. Choose the appropriate individual module for starting data entry.
8. Start entering the module by clicking tab of the created module menu button.
9. The DBMS will ask you to enter the sample ID of the individual just before you finish entering the individual data. This ID will be utilized in the section on biomarker measurement, and it will serve as a link between the other modules and the biomarker module.
10. Here database is a combination of columns and rows; each column represents the variable or questions in the questionnaire and row represents the complete information about the study participants.
11. An inbuilt algorithm in the MySQL program automatically handles skip patterns, filters, and eligibility for questionnaires and sections.
12. Once the data entry completed it will show the information about the new data entry were created. After finishing the one subject data entry move to next subject entry.
13. After completion of all the data entry, re-check it again that all the data were entered correctly or not, if any wrong entry was detected re-enter the correct one. Once everything cross checked properly report to RA and upload the data in the server.
14. The provision of synchronizing data from FI’s & LT’s database to RA’s database provides an opportunity for back check of information to improve data quality.
15. Once the data uploaded in the server there will not be any provision for re-reentering data by the FIs, LTs or RAs. If any errors noticed in the later stage report to the Central Office.
16. Details of detected errors should be mentioned in the log-book by the FIs and RAs.
17. Once the all data uploaded, the RA will confirm the data entry about the day and inform to the central office.
18. Uploaded data will verify and confirm from the Central Office. If any wrong data entry detected, immediately revert back to the RA and if possible, will correct it in the data base.
19. The DBMS dashboard help in generating field check tables on key indicators on a daily basis which the Quality Assurance Team reviews in the central office to allow individual level feedback to be communicated back to the teams working across different districts
20. OTFHS assigns a unique code to each field investigator, which helps track the investigator's progress and performance after individual-level feedback is provided.
21. **Post data collection phase**

Data flow in the post data collection phase is mentioned in the below diagram.

Details of data flow in post data collection phase is given below

**Checking errors in raw data**

- 1. ***Preparatory Analysis***: Preparatory analysis is the process of cleaning and transforming raw data prior to processing and analysis. It involves reformatting, correcting and combining the data sets to enrich data.
     1. Identifying duplicate observations:
        1. Data should be unique, no two or more rows should be same in the dataset
        2. Investigate whether the duplicate observation is an incorrect entry or a genuine duplicate
        3. If the duplicates are genuine, one of the cases should be dismissed.
     2. Variable type
        1. Define the all type of variables clearly
        2. Re-coding needs to be performed as per the requirement: Any variable that does not match the anticipated data type should be transformed before the data is finalized. For instance, data intended to be numeric but is available as string (text) or vice versa.
     3. Missing values
        1. Will Check whether the missing values are expected one or not (due to skip pattern or not)
        2. Not expected one – One needs to check with the field team and document the reason.
        3. Missing values will re-enter if the reasons for missing values resolved
        4. If required, missing values can be imported by using any multivariate imputation method
     4. Post coding
        1. Dealing with text entered in ‘other’ field in the variable: Here, most of the survey have structured responses, there is always a possibility of responses mentioned under “other” categories. If any alongside response is entered in “other” category, then the response will be coded in the pre-coded response category. If a response in the “other” category is repeated frequently (heaping), the response can be coded as a new response category.
  2. ***Descriptive statistics***

The data quality problems that surfaced during the data collection phase can be found via descriptive analysis. It can be clearly understood by using data visualization and descriptive statistics. The details are given below

1. Data visualization: Different charts/ graphs, like a pie chart, bar plot, histogram, etc., will be used to do the data visualization.
2. Descriptive/ Summary Statistics:
   1. Measures of central tendency - mean, median and mode
   2. Measures of dispersion - variance, standard deviation, range, interquartile range, and coefficient of variation.
   3. Measures of distribution - percentiles, quartiles, skewness, and kurtosis
   4. Outlier detection
   5. In the data entry platform itself will give the range of values (maximum, minimum) to avoid outliers
   6. Frequency distribution and/ boxplot to be used (continuous variable)
   7. If outlier exists, will re-confirm with the data collection team
   8. Will flag the outlier values if not possible to confirm with the collection team
   9. Definition of outlier for continuous variable (Using Boxplot)


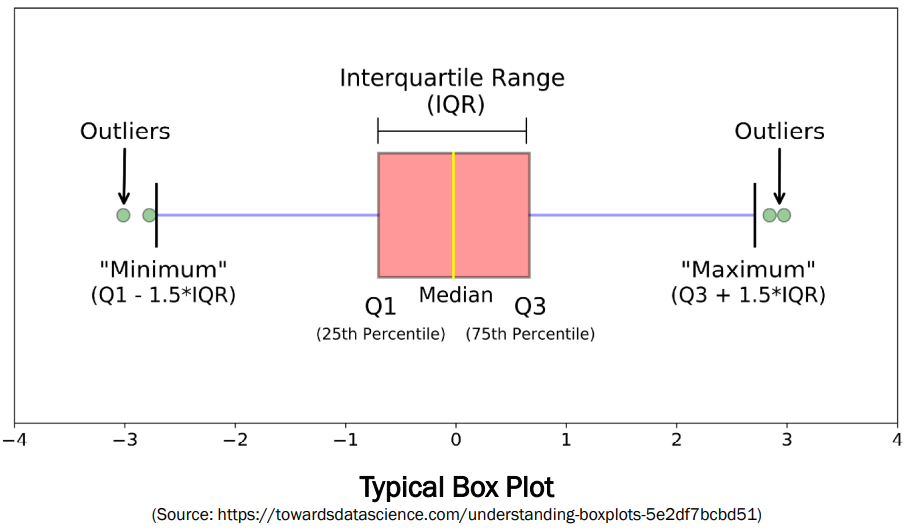


1. Sanity checks
   1. Verify if the data in a variable is consistent with other related variables in the same data
   2. **Preparing metadata;** The meta data will prepare and the following details will be included in the meta data.
      1. definition of all variables
      2. description of all coding values
      3. date of data creation
      4. information about data custodian
      5. documentation of specific data issues

Once the raw data & meta da is ready then will go for final statistical analysis. R (V 4.1.2) for windows software will be used for cleaning and analyzing the data.

# **Monitoring – Laboratory Practices in the field**

1. Remember to chill the cold packs previous night of field survey
2. Check the battery/charge of the tablets, Hb meter and the Glucometer, Gazelle, Omron blood pressure, weighing machine, glucometer
3. Availability of required instruments for anthropometric measurements
4. Availability of consumables necessary for sample testing and collection
5. Availability of MOP with the Field technician
6. Identification of suitable place for measurement and testing
7. Checking of name, age, gender, individual Id & sample Id of the participants prior to anthropometric measurement and testing
8. Height of the participants (all age group) should be measured in bare foot
9. Height of children (<2 years) is to be measured in infantometer
10. Measurement of WHR should be done by same gender (male for male & female for female)
11. Grip strength measurement should be measured as per the convenient choice of hand of the participants
12. Blood pressure measurement should be recorded twice and the average to be recorded (Systolic 1+Systolic 2/Diastolic 1+Diastolic 2= Systolic/Diastolic)
13. Before collection of blood samples explain the procedure and get the verbal consent
14. Measure the BP and collect the samples after making the participants sit comfortably
15. Behave politely with the participants and don’t pressurize if they deny giving samples.
16. Perform the hand sanitization for the participants and self prior to each capillary blood collection
17. After finger pricking, 1^st^ drop of blood to be wiped off with cotton and then test the Hb & Glucose
18. Single prick to be done for both the test.
19. If there is insufficient blood from 1^st^ prick, next finger can be pricked with same lancet.
20. After completion of test, put dry cotton/gauge at the pricking site
21. The participants should be requested to discard the cotton in the biohazard bag
22. For the participants to be tested for Sickle cell, the blood from the syringe should be 1^st^ collected in EDTA vacutainer then in gel vacutainer.
23. Avoid needle prick injury while transferring the blood in 2 different vacutainer.
24. Barcode of the vacutainer should be cross checked before collection of sample.
25. After each collection store the tubes in upright position in vaccine carriers
26. While performing the SCD test mark the 3 MCTs first (buffer, dye and sample)
27. While performing the SCD test the proper mixing of blood sample and dye should be done (20 sec using pipette).
28. Once the result of SCD is displayed, record the screen shot using mobile as well as write the value.
29. The required parameters to be recorded in app as well as in the report format to be provided to the participants.
30. Lab tally sheet should be maintained properly for sample collection/no sample.
31. Parameter should be immediately recorded after measurement
32. In laboratory, serum should be separated by centrifuging the vacutainer at 2800 rpm for 10mins
33. Serum should be separated using the pasteur pipettes and stored in cryovial.
34. Each cryovial should be leveled with the barcode and a cellotape over the barcode to avoid erase due to alcohol and water.
35. Barcode of the cryovials should be cross checked before transferring the serum from vacutainer.
36. Cryovials should be stored in cryoboxes as per the lab tally sheet and kept in cold storage
37. Before transporting the samples to Bhubaneswar, all transport boxes should be packed with sufficient gel pack.
38. All cryoboxes should be labeled properly with Sl no (from-to)
39. Before transporting the samples to RMRC Bhubaneswar the cold chain packaging should be maintained properly and the transport box should be properly labeled and sealed.
40. The lab tally sheet must be provided with the each time sample transport to the RMRC laboratory.

# **Anthropometric Measurement & Biomarker tests**

Final sample packaging and transport to RMRCBB

Serum separation, storage & entry in lab tally sheet, discard of BMW

Transport of samples to DHH

Final data entry & issue of test report

Filling of Lab tally sheet

## **Rationale of the Study**

The rationale of this standard operating procedure includes:

- To demonstrate the procedure of anthropometric measurements of body which provides valuable assessment of health and nutritional status in children and adults.
- To demonstrate the step wise procedure of height and weight measurement, waist to hip ratio to assess obesity and grip strength measurement.
- To instruct the operating process of the equipments i.e hand dynamometer, OMRON blood pressure measuring instrument to record the grip strength value and systolic and diastolic blood pressure level.

## **Equipments Required for Anthropometric Measurement**

- **Electronic weighing scale:** for weighing children and adults. The scale has amaximum capacity of 180 kg.
- **Stadiometer:** for measuring the height of adults and children above 2 years.
- **Infantometer:** for measuring the length of children under 2 years or less than 85 cm.
- **Measuring Tape:** for measuring waist-to- hip ratio (WHR).
- **Digital Hand Dynamometer:** for measuring isometric grip force.
- **Blood Pressure Recording Unit Digital Automated**: for measuring the systolic and diastolic blood pressure.

## **Setting up of Measurement and Sample Collection site**

An ideal place will be identified in the survey village where the anthropometric instruments will be set up and clinical sample collections will be performed. The sites like

- Anganwadi centre
- School
- Front area of any participant’s house (with the consent from the house owner)
- Any shaded place with sufficient light

The portable table and chair will be installed in the identified place where all the field laboratory set up will be done.

## **Roles and responsibilities**

Laboratory technician 1/Laboratory technician 2/Field investigator: To coordinate and record the anthropometric measurement.

## **Checklist before proceeding anthropometric measurement**

- **Layout of the Procedures**: Each step of the measurement procedures is directed at specific participants. The measurer (LT or FI) have to cross check the individual Id and sample Id in Field Investigation Table module and in the report format prior to the measurement.
- **Two person required**: Two people (parents/guardian) are required to measure a child's height or length. The measurer holds the child and takes the measurements. The child’s parent/guardian will helpin measurement. Measurer alone can take the weight of a child and record the results.
- **Age Assessment:** Before you measure, determine the child's age. If the child is less than two years, measure length (that is, with the child lying). If the child is two years of age or older,measure height (that is, with the child standing). If accurate age is not possible to obtain,measure length if the child is less than 85cm. Measure height if the child is equal to or greater than 85cm.
- **Weigh and Measure One Child at a Time:** If there is more than one eligible child in a household, complete the weighing and measuring of one child at a time. Then proceed with the next eligible child.
- **Control the Child:** When you weigh and measure, you must control the child. The strength and mobility of even very young children should not be underestimated. Be firm yet gentle with children. Your own sense of calm and self-confidence will be felt by the parent and the child.

When a child has contact with any measuring equipment, i.e., on an infantometer, you must hold and control the child so the child will not trip or fall. Never leave a child alone with a piece of equipment.

- **Coping with stress:** Since weighing and measuring requires touching and handling children, normal stress levels for this type of survey work are higher than for surveys where only verbal information is collected.

Explain the weighing and measuring procedures to the mother, father, or other responsible adult and to a limited extent, the child, to help minimize possible resistance, fears or discomfort they may feel.

**The criteria for non measurement**

- The parent/responsible adult refuse.
- The child is too sick or distressed.
- The child is physically deformed which will interfere with or give an incorrect measurement.

## **Steps For Measuring Weight**

- Perform the adult weight measurements according to the instructions below.

## **Weight Measurement of Adult and children**

- *Preparing the adult and children to take their weight*

Show the scale to the adult and explain that you will weigh her/him and the children on thescale.

- *Preparing the Scale*

Before the measurement starts place the weighing scale on a flat surface. Uneven surfaces or vibration may cause the scale to malfunction.

**The machine operation and steps involved in weight measurement is mentioned below as per Manufacturer’s instruction:**


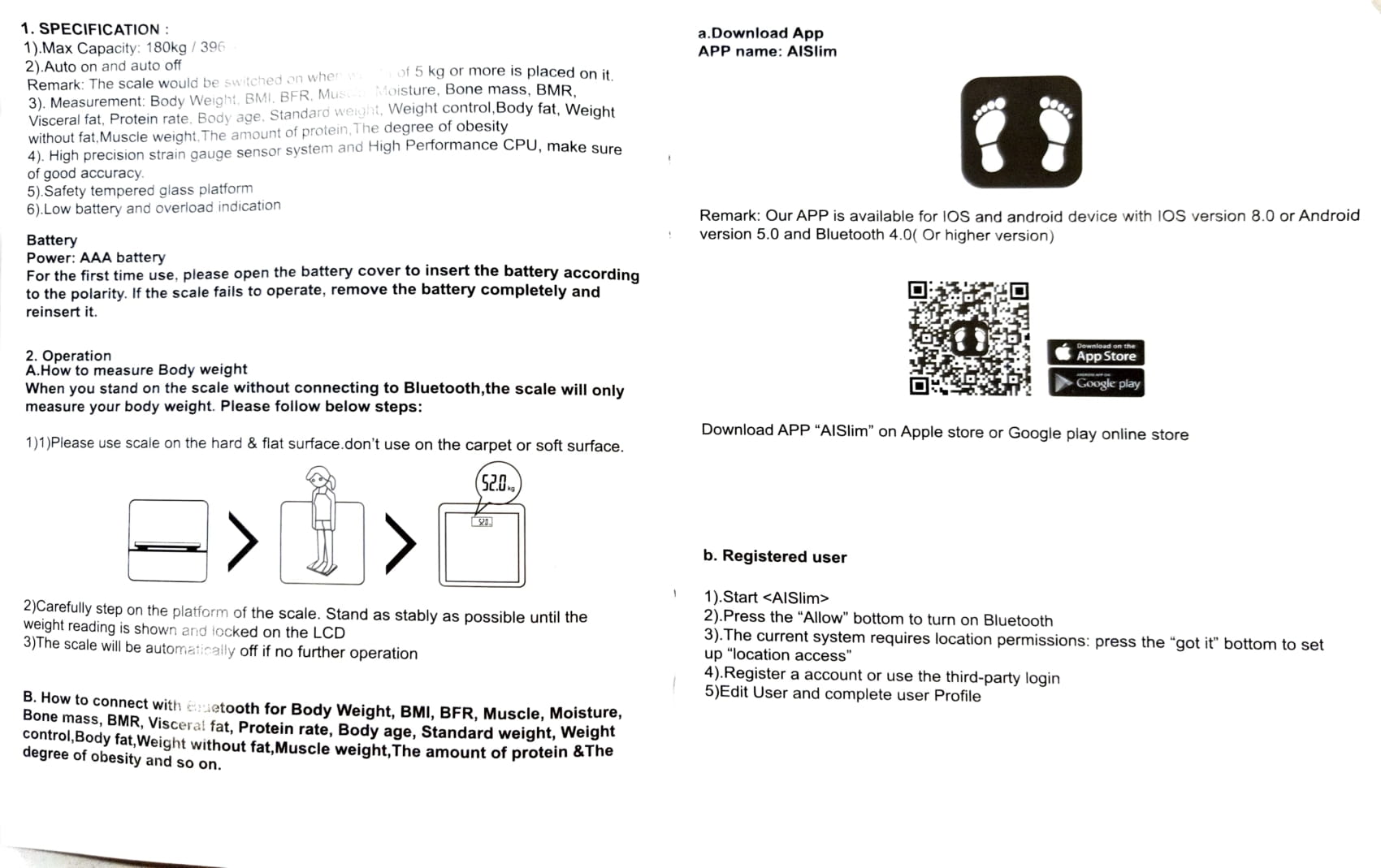

### Weighing Infants or Children Who Must be Held by an Adult While on the Scale

- For weighing the infant or the child on the scale, first the weight of the adult’s weight will be measured who is going to carry the infant or the child.
- Next the adult will be instructed to get down from the weighing machine so that the machine reading becomes 0.00 again.
- The adult will be instructed to carry the infant or child (to be measured) and the weight will be measured again in the weighing machine.
- For calculating the infant or child’s weight the adult measured weight will be deducted from the second measured weight (infant or child plus adult) and the infant weight will be recorded.

## **Steps For Measuring Height**

### Measuring an adult’s height

- Place the stadiometer on a hard, flat surface against a wall, table, tree or staircase. Make sure the stadiometer is stable. Many walls and floors are not at perfect right angles; if necessary, place small rocks underneath the height board to stabilize it during the measurement.
- Ask the person to take off his/her shoes and ask him/her to unbraid or push aside any hair that would interfere with the height measurement. Ask the person to stand on the base of the height stadiometer and to face forward.
- Determine if the person’s feet should be against or away from the back of the height board by observing the imaginary line drawn from the tip of the shoulder to the heel, which is called the “mid-maxillary line” (Arrow3). This line should be perpendicular (i.e. 90°) to the base of the stadiometer where the person is standing. Note that with almost all adults you will have to move the person’s feet away from the back of the height board to put the min the proper position; (Arrow 4).
- Place the knees and feet in the correct position, with knees and feet either together or apart. There are three possible positions for the knees and feet:
- Knees together and feet together
- Knees together and feet apart Whichever touches first!
- Knees apart and feet together


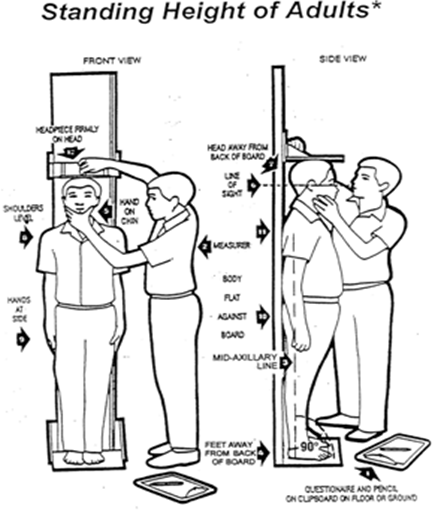


**Adult Height Measurement**

(Figure courtesy: NFHS -4 manual)

- Ask the person to look straight ahead. Cup the respondent’s chin between the thumb and index finger of your left hand and gradually close your hand (Arrow 5). Position the person’s head so that the line of sight is parallel to the ground (Arrow 6). Note that with most adults, the back of the head will not touch the back of the stadiometer—there will be a space between the back of the person’s head and the back of the stadiometer (Arrow 7). After you have placed the person’s head in the proper position, release your hand from the person’s chin and ask him/her to hold his/her head in the position you have just placed it in.
- Make sure the person’s shoulders are level (Arrow 8), the hands are at the person’s side (Arrow 9), and at least the buttocks touches the back of the stadiometer). Note that with most adults, only the buttocks and perhaps the shoulder blades, will touch the back of the stadiometer (Arrows 10 & 11).
- Check the position of the person (Arrows 1-11). Repeat any steps as necessary.
- When the person’s position is correct, lower the head piece on top of the head (Arrow 12) Making sure to push through the person’s hair. **Read and call out the measurement to the nearest 0.1 cm.** Remove the headpiece from the person’s head, and escort the person off the height board.
- Immediately record the measurement on the questionnaire. Record an adult’s **height** measurements in **Questionnaire**. If the adult’s height was not measured, record the appropriate code in **Questionnaire**.
- Check the recorded measurement on the questionnaire for accuracy and legibility. Correct any errors.

### **Measuring a Child’s Height: Standing Up**

- Ask the parent to take off the child’s shoes and to unbraid or push aside any hair that would interfere with the height measurement. Ask the parent to bring the child to the stadiometer and to kneel in front of the child so that the child will look forward at the parent.
- Kneel on the left of the child (Arrow3).


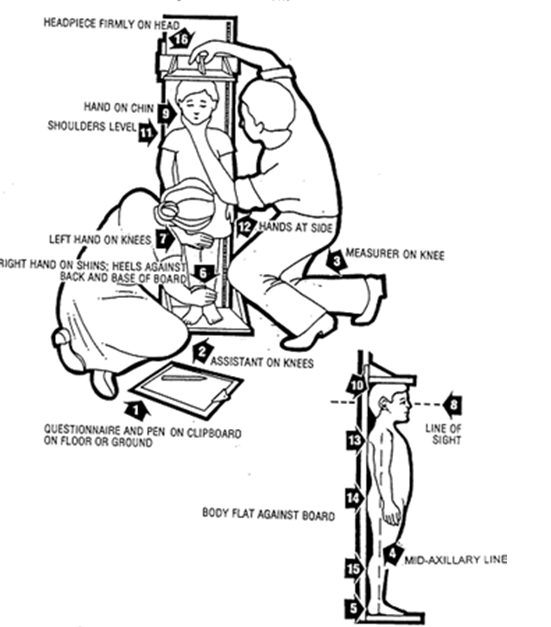


**Child Height Measurement**

(Figure courtesy: NFHS -4 manual)

- Place the child’s knees and feet in the correct position, with knees and feet either together or apart. There are three possible positions for the knees and feet:
- Knees together and feet together
- Knees together rand feet apart whichever touches first!
- Knees apart and feet together
- Determine if the child’s feet should be against or away from the back of the stadiometer by observing the imaginary line drawn from the tip of the shoulder to the heel, which is called the “mid-axillary line” (Arrow 4). This line should be perpendicular (i.e.90°) to the base of the stadiometer where the child is standing.

(You may have to move the child’s feet away from the back of the stadiometer to put them in the proper position). Note that with most preschool-age children who are not heavy or obese, the heels will probably touch the back of the height board (Arrow 5).

- With your thumbs against the index finger of each hand, place your right hand on the child’s shins (Arrow 6) and your left hand on the child’s knees (Arrow 7). Do not wrap your hands around the knees or feet (ankles) or squeeze them together. Make sure the child’s legs are straight.
- Ask the child to look straight ahead at the parent if she is kneeling in front of the child. Make sure the child’s line of sight is parallel to the ground (Arrow 8). Place the thumb and index finger of your left hand, one finger on each side of the child’s chin, and gradually close your hand (Arrow 9). Note that with most preschool-age children who are not heavy or obese, the back of the head will touch the back of the stadiometer (Arrow 10); however, if the child is heavy or obese, there will be a space between the back of the child’s head andthe back of the stadiometer. Make sure the child’s shoulders are level (Arrow 11), the hands are at the child’s side (Arrow 12), and at least the child’s buttocks touch the back of the stadiometer. Note that with most preschool-age children who are not heavy or obese, the back of the head, the shoulder blades, the buttocks, the calves and heels will touch the back of the stadiometer (Arrows10,13,14,15&5).
- Check the position of the child (Arrows 1-15). Repeat any steps as necessary.
- When the child’s position is correct, lower the headpiece on top of the child’s head (Arrow 16) making sure to push through the child’s hair. Read and call out the measurement.
- Immediately record the **height** measurementson the questionnaire.
- Check the recorded measurement on the questionnaire for accuracy and legibility.

### **Measuring a Child’s Length: Lying Down**

This method is used for the children who are less than two years old. The length measured for children in this way is less than 85 centimeters.

- Place the infantometer on a hard, flat surface, such as the ground, floor or a solid table to ensure the infantometer is stable.Place the biomarker questionnaire (tablet) on the ground, floor or table (Arrow 1) and kneel behind the fixed head end of the infantometer if it is on the ground or floor (Arrow 2).
- Kneel at the right side of the child (at the child’s feet) so that you can move the sliding foot piece with your right hand (Arrow3).
- With the help of the parent, gently lower the child on to the infantometer, making sure the measurer supports the child at the trunk of the body while the assistant supports the child’s head.


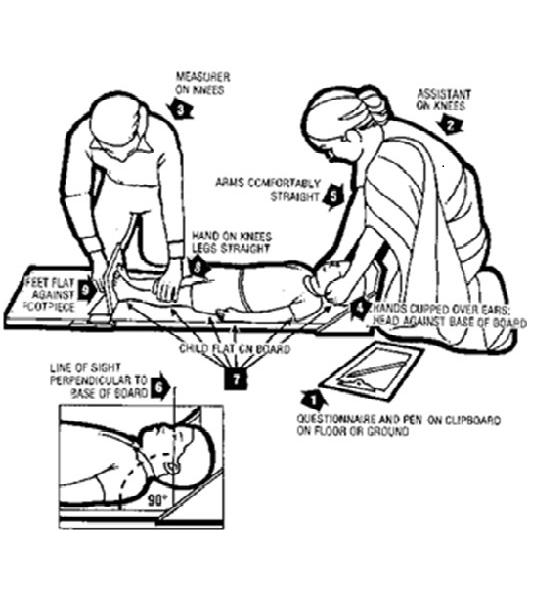


**Child Length Measurement**

(Figure courtesy: NFHS -4 manual)

## **Measurement of Waist –to-Hip Ratio (WHR)**

An important issue in using and interpreting waist circumference or waist–hip ratio is the protocol used to obtain the measurements. Also important is the extent to which the measurement protocol varies across studies, and the potential for standardizing these measurements within a study or survey, when taken by different people (2).

- **Placement, tightness and type of measuring tape**
- **Placement of tape**

### Waist circumference

The WHO STEPS protocol for measuring waist circumference instructs that the measurement be made at the approximate midpoint between the lower margin of the last palpable rib and the top of the iliac crest.

**Abdominal obesity is further defined as waist–hip ratio above 0.90 for males and above 0.85 for females, or a BMI above 30.0**(3).

### Hip circumference

The protocols for hip circumference measurement should be taken around the widest portion of the buttocks. This include following steps:

### Tightness and type of tape

The accuracy of waist and hip circumference measurements depends on the tightness of the measuring tape, and on its correct positioning (i.e. parallel to the floor at the level at which the measurement is made). For both waist and hip, the tape should be snug around the body, but not pulled so tight that it is constricting.

### Abdominal tension at the point of measurement

The tension of the abdominal wall influences the accuracy of the waist circumference measurement. Lowering the tension of the abdominal wall increases waist circumference, whereas increasing the tension (by sucking in) reduces waist circumference. Many individuals unconsciously react to waist measurements by sucking in the abdominal wall; hence, a relaxed posture is best for taking waist measurements. It is recommended to advise the subject to relax and take a few deep, natural breaths before the actual measurement is made, to minimize the inward pull of the abdominal contents during the waist measurement.

### Measurement protocol

### Measure the *waist circumference* at the end of several consecutive natural breaths, at a level parallel to the floor, midpoint between the top of the iliac crest and the lower margin of the last palpable rib in the mid axillary line.

### Measure the *hip circumference* at a level parallel to the floor, at the largest circumference of thebuttocks.

### Make both measurements with a stretch‐resistant tape that is wrapped snugly around the subject, but not to the point that the tape is constricting. Keep the tape level and parallel to the floor at the point of measurement.

### Ensure that the subject is standing upright during the measurement, with arms relaxed at the side, feet evenly spread apart and body weight evenly distributed.

## **GRIP STRENGTH MEASUREMENT**

##### Hand-held dynamometers may also be used to test the strength of individual muscles or muscle groups. A dynamometer is a device that can measure force.

### **Digital Hand Dynamometer Operating Protocol and Procedure**

- Ask the participant to sit in the chair and to hold the dynamometer in one hand (right or left hand whichever the participant uses most frequent to carry out work.
- The hand should be stretched parallel along with the floor.
- Now instruct the participant to pull the handle as strong as he/she can for 10 seconds and then to release it.
- Record the reading from the dynamometer display.

Follow the manufacturer’s instruction:


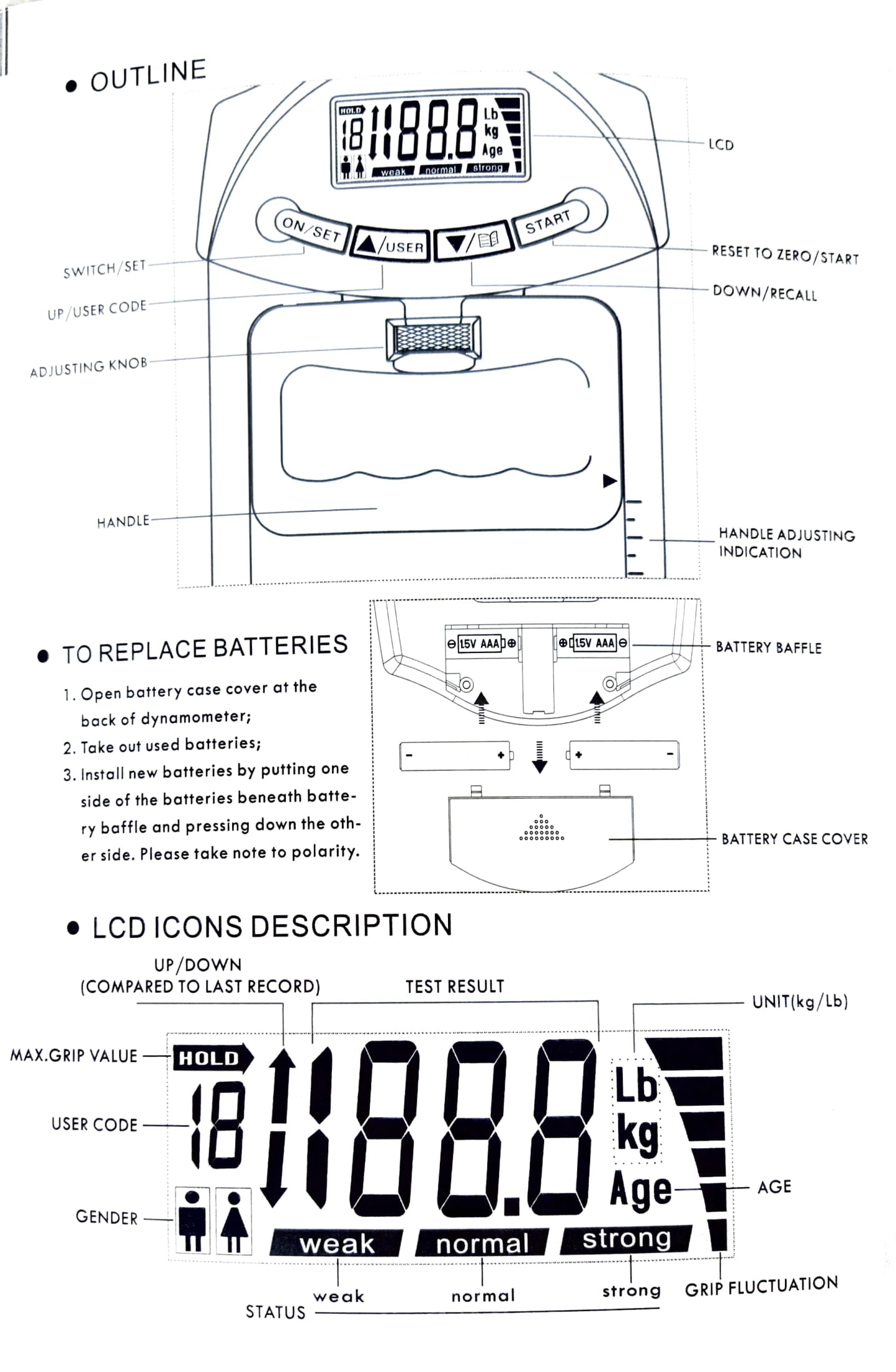


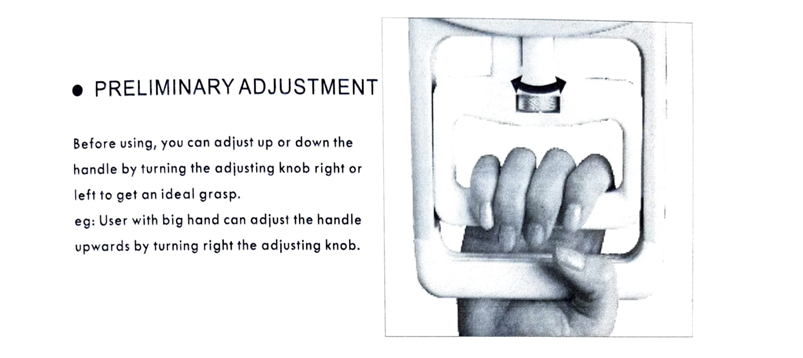

## **BLOOD PRESSURE MEASUREMENT**

##### High blood pressure can damage arteries by making them less elastic, which decreases the flow of blood and oxygen to heart and leads to [heart disease](https://www.cdc.gov/heartdisease/index.htm). In addition, decreased blood flow to the heart can cause chest pain, also called angina. Heart attack occurs when the blood supply to the heart is blocked and heart muscle begins to die without enough oxygen.

 The normal blood pressure range is between 115/75 and 120/80 mmHg. There are three levels of high blood pressure.

- ***Prehypertension*:** The systolic blood pressure ranges between 120 and 139 mmHg or the diastolic blood pressure is between 80 and 89 mmHg.
- ***First stage:*** The systolic blood pressure ranges between 140 and 159 mmHg or the diastolic blood pressure is between 90 and 99 mmHg.
- ***Second stage:*** The systolic blood pressure is 160 mmHg or higher, or the diastolic blood pressure is 100 mmHg or higher.

### **Preliminary Steps In Taking Blood Pressure Measurements From Adults**

- Before starting the blood pressure measurements, the respondent (The participant whose blood pressure will be measured) should have been sitting quietly for at least 5 minutes.
- Be sure that the respondent does not smoke or drink coffee or tea during the measurements since smoking or drinking coffee or tea can affect the blood pressure. If the respondent consumed any alcohol, coffee or tea, or smoked cigarettes before the examination, record this in Questionnaire.
- If the respondent indicates any reason why the blood pressure procedure should not be done on the left arm, use the right. If there is a problem with both arms, do not take the blood pressure and note this in Questionnaire. Observe the respondent’s arm while talking to him/her. If you observe any rashes, small gauze / adhesive dressings, casts, with ere arms, puffiness, tubes, open sores, haematomas or wounds on both arms, do not take the blood pressure.

### Positioning the Respondent for Blood Pressure Measurement

- The respondent should be seated at a table in a relaxed, but not slouched, position with feet flaton the floor. The outer jacket or sweater should be removed and the sleeve should be rolled loosely up to the shoulder, ensuring that two fingers can be placed under the sleeve without difficulty. The respondent’s left arm should be placed on the table, slightly flexed with palmupward.
- The respondent’s arm should be positioned so that it is resting on the table at heart level. Heart level is halfway between the shoulders and the waist. The respondent’s elbow must be no lower than the lowest rib and must not be raised as high as the shoulder.
- If the respondent is tall, it may be necessary to support the arm higher than a standard desk or table-top. Place the tall respondent’s forearm on a pillow or a large book to raise the arm to heart level.
- For smaller or shorter adults, place a cushion or large book on the chair so that the arm is at heart level when the arm is resting on the desk or table-top. Place a box or large book under the respondent’s feet if the feet do not rest flat on the floor.
- The health investigator should be seated facing and slightly to the left of the respondent, permitting easy access to the respondent’s arm. The measuring equipment will be positioned so that the tube to the manometer is away from the respondent’s body while the tube to the inflation bulb is closer to the body.

### How To Seat The Respondent Correctly When Taking a Measurement

- Make sure that all the components of the OMRON BP monitor are present and it is kept at the left side of the respondent if the blood pressure is going to measure from left hand.
- Correct posture during measurement is necessary to get accurate results. Examples of incorrect posture:
- Arched back (leaning forwards)
- Sitting cross-legged
- If the arm cuff is at a lower position than the respondent’s heart, use cushions or pillow to adjust the height of the respondent’s arm.
- Insert the airplug into the airjack (on the left side of the device). The cuff must be fully deflated when it is inserted into the air jack.
- Have the respondent sit in a chair with her/his feet flat on the floor and place her/his arm on a table so that the cuff will be at the same level as her/his heart.
- Have the respondent sit in a chair with her/his feet flat on the floor and place her/his arm on a table so that the cuff will be at the same level as her/his heart.
- Hold the grip on the cuff securely with your hand.
- Turn the palm of the respondent’s hand upward.
- Apply the cuff to the respondent’s upper arm so that the air tube is centred on the middle of the respondent’s inner arm and points down the inside of the arm. The air tube should run down the inside of the respondent’s forearm and be in line with her/his middle finger.
- The bottom of the cuff should be approximately 1 to2 cm above the elbow.
- Whenthecuffispositionedcorrectly,closethe fabricfastenfirmly.
- Makecertainthecufffitssnugly aroundthearm.
- The cuff should make good contact with the respondent’s skin. You should be able to fityour index finger between the cuff and the respondent’s arm easily, so you can pull thecuff off andon.

### Measurements on the right arm

- **Note the following points when applying the cuff to your right arm. Apply the cuff so that the airtubeisatthe side ofthe respondent’selbow.**
- Be careful not to rest your arm on the air tube, or otherwise restrict the flow of air tothe cuff.
- Applythearmcuffsothatnopartofthecuffispositionedovertheelbowjoint.Thecuffshould be 1 to 2 cmabove the elbow.

### Taking a Blood Pressure Reading

For OTFHS, the **OMRON BP Monitor** will be used for respondents with small, medium and large arm circumference. Measure the respondent’s arm circumference to enable you select the correct cuffsize for use. Press the **START** button and ask the respondent to remain still; the cuff will start to inflate automatically. As the cuff begins to inflate, the monitor automatically determines the ideal inflation level. The respondent should remain still and not talk until the measurement is completed.

**Note:** Do not inflate the cuff if it is not wrapped around the respondent’s arm.

Inflation stops automatically and measurement is started. As the cuff slowly deflates, decreasing numbers appear on the display and the Heartbeat display
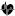
flashes at every heartbeat. In rare circumstances, the monitor might re-inflate the cuff to continue with the measurement.

- **START INFLATE DEFLATE END COMPLETE**
- When the measurement is complete, the arm cuff completely deflates and the blood pressure [and pulse rate] readings are displayed. Record the readings in the appropriate boxes.
- Remove the arm cuff from the respondent’s arm.
- After the measurement is completed, you can either press the O/I start button to turn the monitor off or it will shut off automatically after 5 minutes.
- **Blood Pressure Monitoring Repeatation**
- **For each participant, blood pressure will be measured for two times with a gap of 5 minutes. The average of the both systolic and diastolic readings will be recorded.**

###### Safety and precaution:

- Do not use the instrument close to strong electromagnetic fields such as **mobile telephones or radio installations.**
- If the instrument is not going to be used for a prolonged period, the batteries should be removed.
- Do not open the instrument.

#### WHAT DISPLAY SYMBOLS AND ERROR MESSAGES MEAN


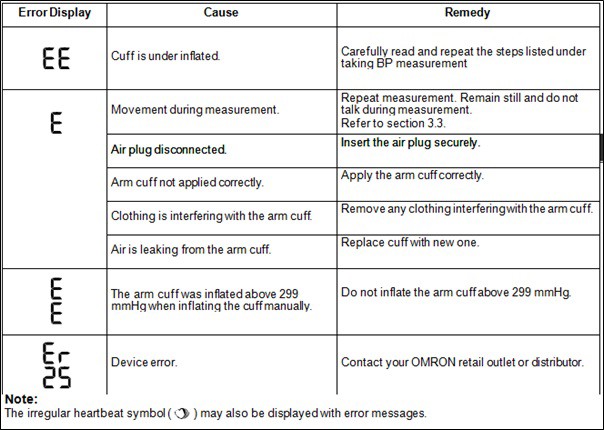


### Troubleshooting


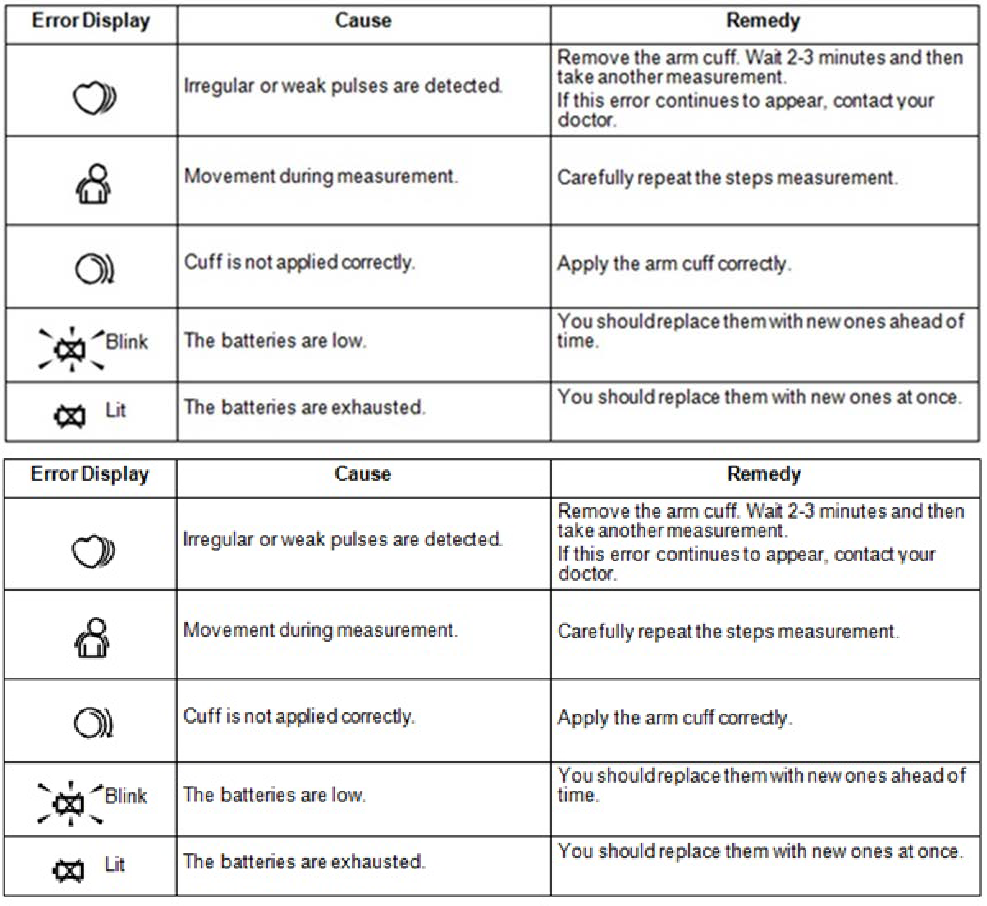


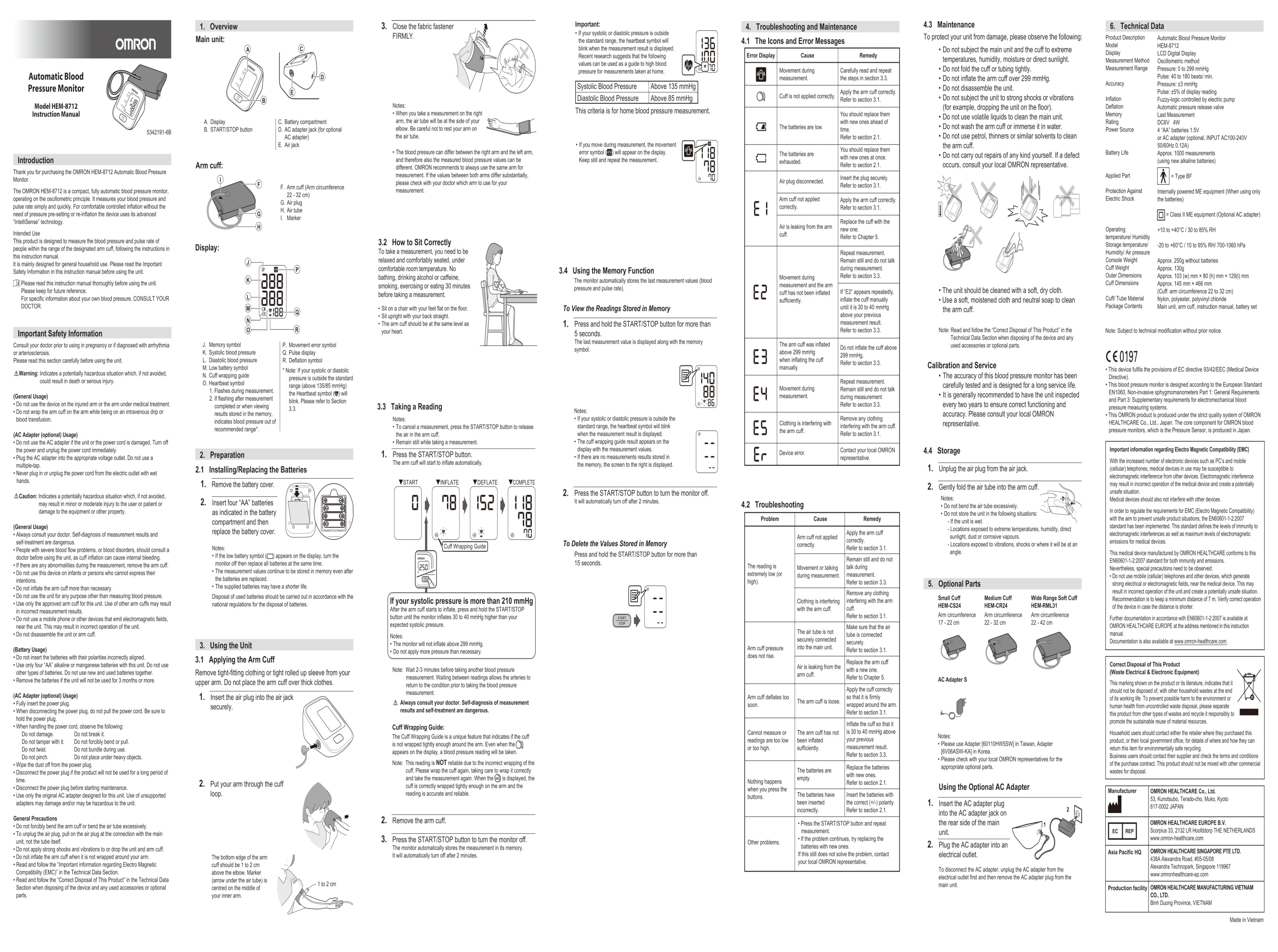


# **Blood Sample Collection in Field**

## **Rationale for Testing**

The rationale of this standard operating procedure is as follows:

- To safely collect the venipuncture blood from the study participants to diagnose and monitor any blood disorder. Blood sample analysis indicates the overall health condition of participants and reveals more specific diseases and conditions.
- To safely collect capillary blood from the study participants for performing theblood sugar test and haemoglobin test in.
- To safe and timely delivery of the blood sample to the laboratory for processing.
- To store the primary samples for appropriate testing and analysis.

### **Materials Required**

- Purple top Ethylenediaminetetraacetic acid (EDTA) blood tubes
- Gel vacutainer
- Syringe with needle(2ml and 5ml)
- Tourniquet band
- Alcohol swab
- Sanitizer
- Absorbent cotton
- Band aid
- Vacutainer rack
- Vaccine carrier
- Gel cool pack
- Biohazard bag
- Bag sealer
- Gloves
- Mask
- Needle destroyer
- Lancet
- Glucometer with strip
- Haemoglobinometer with strip
- Gazelle Hb variant
- Pipette
- Micro centrifuge tube
- Glass slide
- Tips

## **Preparation of the Study Participants**

Before the procedure it is important to follow the steps mentioned below:

- Introduceyourselftotheparticipant,andask him/her tostatetheirfullname.
- Discuss the test to be performed and obtain verbal consent. The participant has aright to refuse a test at any time before the blood sampling, so it is important to ensure thattheparticipanthasunderstoodtheprocedure.

## **Roles and responsibilities**

Laboratory technician 1/Laboratory technician 2: To coordinate and perform capillary blood test and venous blood collection and to record the testing data in the field investigating table manual and in report format.

## **General Procedures For Collection of Capillary Blood Drop Samples From Adults And Children**

Capillary sampling from a finger or heel (in case of infants) an ear lobe may be performed on patients ofanyage,forspecificteststhatrequiresmallquantitiesofblood.However,becausetheprocedureis commonly used in paediatric patients.Capillary blood will be collected to test for the following biomarkers: haemoglobin (anaemia) and random blood glucose (risk for diabetes). Capillary blood can be obtained from the palm side of the end of a finger. Following steps involved in obtaining a capillary blood sample from adults and children.

## **Choice of site**

- For adults and children: Thefingerisusuallythepreferredsiteforcapillarytestinginanadultpatient as well as children.Thesidesoftheheel are only used in paediatric and neonatal patients.
- For paediatric and neonatal patient (6-12 months): Selection of a site for capillary sampling in a paediatric patient is usually based on the age and weight of the patient. If the child is walking, the child’s feet may have calluses that hinder adequate blood flow. The table shows the conditions influencing the choice of heel or finger-prick.

## Preparation of lancet for use:

- **For adults:** A lancet slightly shorter than the estimated depth needed should be used because the pressurecompresses the skin; thus, the puncture depth will be slightly deeper than the lancet length.
- **For paediatric and neonates:** Inheel-pricks,thedepthshouldnotgobeyond2.4mm.Forprematureneonates,a0.85mmlancetisavailable.

**Complications**

Complicationsthatcanariseincapillarysamplinginclude:

- Collapse of veins if the tibial artery is lacerated from puncturing the medial aspect of theheel;
- Osteomyelitisoftheheelbone(calcaneus)
- Hervedamageifthefingersofneonatesarepunctured
- Haematomaandlossofaccesstothevenousbranchused;
- scarring;
- Localizedorgeneralizednecrosis(along-termeffect);
- Skin breakdown from repeated use of adhesive strips (particularly in very young or veryelderlypatients)–thiscanbeavoidedifsufficientpressureisappliedandthepuncturesiteisobservedafter theprocedure.

## Steps In Obtaining Capillary Blood From The Finger Of Adults and Children

Remember the following before collecting the capillary blood drop sample from the finger.

- Chose a place where maximum light availability is there.
- Always put on gloves before beginning the collection of the blood sample from the first participant.

### Select and prepare the prick site

- Blood collection is usually easier if you sit on the side of the participant opposite to the hand that you will collect blood from. For example, if you want to collect blood from the left hand, place yourself to the right side of the participant.
- Only use the third or fourth finger for collecting the blood. Do not use a finger with a scar, a wound or cut, an infection, swelling, a deformity, or a rash.
- Also, do not use a finger on which the respondent is wearing a ring, because the ring may disrupt the free flow of blood to the tip of the finger. You can ask the participant to remove the ring.
- Ask the participant to warm his/her fingers by rubbing the palms together briskly until the skin becomes warm. This will increase blood flow to the fingertip and improve the ease with which a sample can be obtained.
- With an alcohol swab, clean the skin of the finger thoroughly. If the swab is stained (with dirt), clean the finger a second time or until the swab is no longer stained. Allow the alcohol to air dry. Do not blow on the area to dry the alcohol. Blowing may deposit bacteria on the skin and contaminate the prick site.

## Prick the Finger

- Make sure that the finger is below the level of the participantt’s heart to increase the flow of blood to the finger. With your thumb, gently push the blood from the top knuckle toward the fingertip.
- When your thumb reaches the fingertip, maintain a gentle pressure to trap the blood in the finger tip.
- Place the lancet firmly against the skin with the trigger facing upwards, so that the arrow preceding the trigger is visible.
- Avoid placing the lancet on the very tip of the finger or the sides beyond the palm area or you will risk piercing the underlying bone. Proper puncture sites are shown in Figure.
- Use the lancet to prick the skin by placing the blade-slot surface against the area and pressing the trigger. The tip of the blade ejects through the blade slot, producing a micro-incision in the skin, and immediately retracts into the device. After pricking the skin, discard the lancet in the biohazard bag.

## Collect the blood drops

- When the blood appears, use a sterile gauze pad to wipe away the first one or two drops of blood depending on the tests being performed.
- If the blood stops flowing before you have collected, the pricking procedure may be repeated with the respondent’s consent.
- For minors, you must get consent from the parent or adult responsible for the child. Do not reuse any of the supplies used for the first finger prick.
- After the blood collection, discard all used materials in the biohazardous waste bag.

**
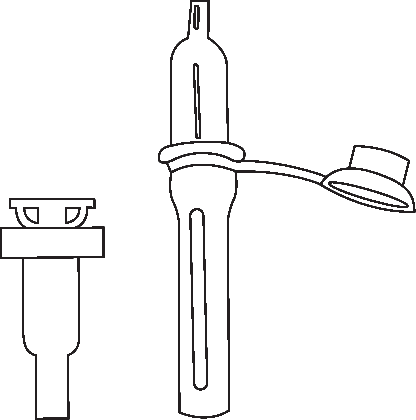
Figure : Illustration of capillary sampling**

**
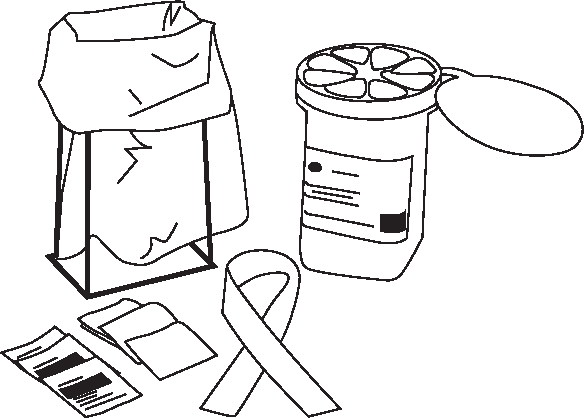
** 1. Lancet and collection tube

2. Assemble equipment and supplies

3.Perform hand hygiene using soap and water/sanitizer

**
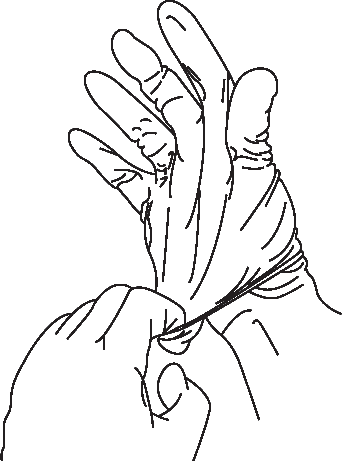
**

4.Put on well fitting non sterile gloves

**
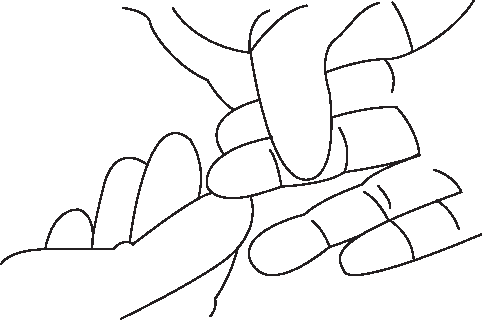
**


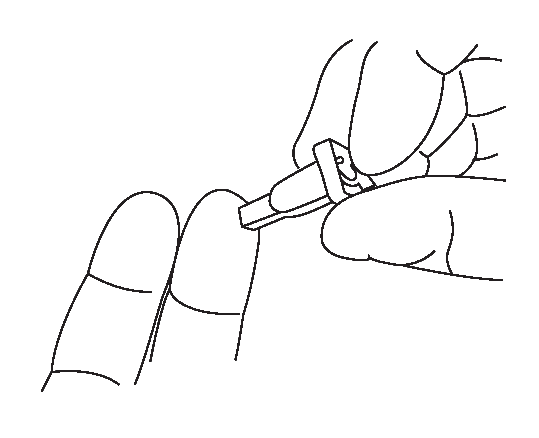
 5.Select the site. Apply 70% alcohol and allow to dry

6.Puncture the skin

**
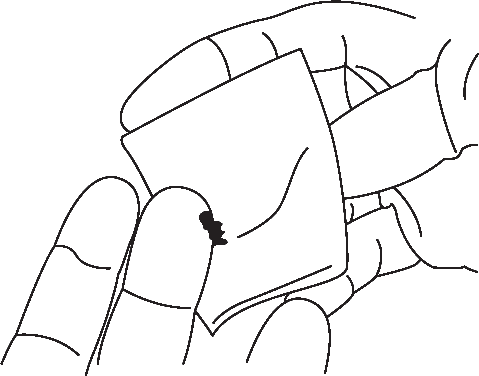
**

7.Wipe away the first drop of blood

**
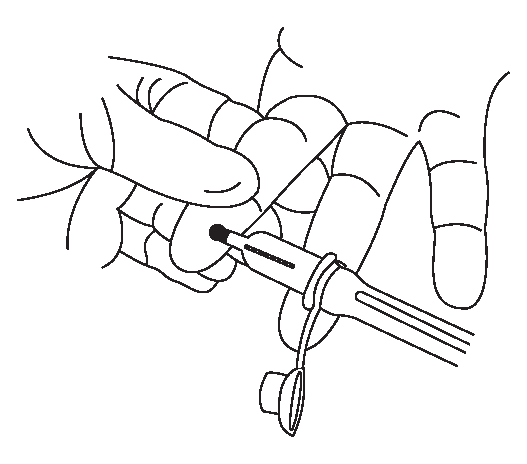
**

8.Avoid sqeezing the finger too tightly


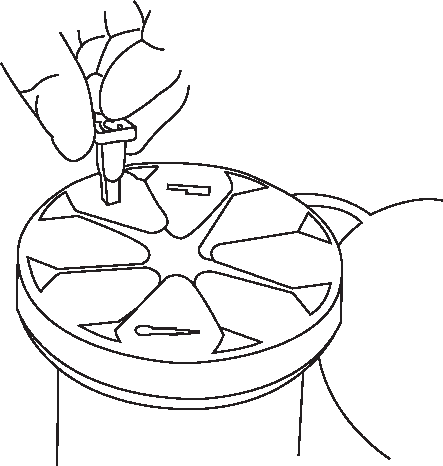
9.Dispose all the sharps appropriately

**
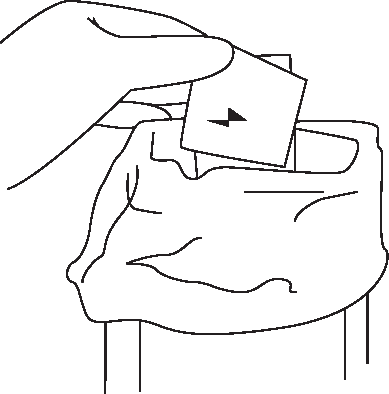
**

10.Dispose the waste materials appropriately

11.Perform hand hygiene after removing the gloves

## General Procedures For Collection of Capillary Blood Drop Samples From Paediatric And Neonates

For children 6 – 12 months, a heel prick should be performed. The following describes the steps that are involved in obtaining a capillary blood drop sample from the heel:

- The prick should be made outside a line drawn from the middle of the big toe to the heel or outside a line drawn from the area between the fourth and fifth toes to the heel.
- Take care to avoid the central area of the foot (to avoid injury to the nerves and tendons) or the centre of the heel (to avoid piercing the heel bone).
- Inheel-pricks,thedepthshouldnotgobeyond2.4mm.Forprematureneonates,a0.85mm lancetisavailable.
- Thedistancefora7pound(3kg)babyfromouterskinsurfacetoboneis:
- medialandlateralheel–3.32mm.
- posteriorheel–2.33mm(thissiteshouldbeavoided,toreducetheriskofhittingbone).
- toe–2.19mm.
- Therecommendeddepthforafinger-prickis:
- forachildover6monthsandbelow8years–1.5mm.
- forachildover8years–2.4mm.
- Toomuchcompressionshouldbeavoided,becausethismaycauseadeeperpuncturethanisneededtogetgoodflow.


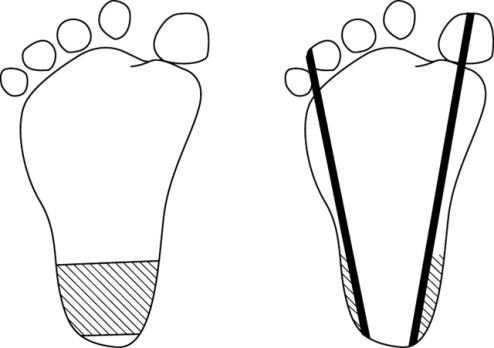

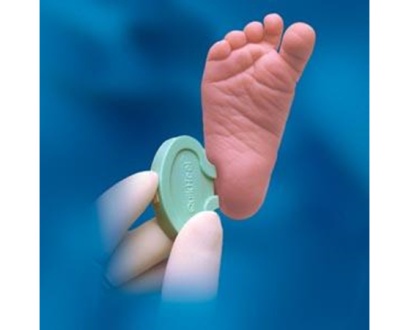


**Figure :** The capillary blood has to be drawn **Figure :** The area ideal for sample collection

from the shaded portion of the hill as by hill prick.

described in the figure.

- Immobilizethechild

Immolization of the child is important before collection of sample. This can be achieved with the help of the parents:

- Sitonthephlebotomychairwiththechildontheparent’slap;
- Immobilizethechild’slowerextremitiesbypositioningtheirlegsaroundthechild’sina cross-legpattern;
- Extendanarmacrossthechild’schest,andsecurethechild’sfreearmbyfirmlytucking itundertheirown;
- Graspthechild’selbow(i.e.theskinpuncturearm),andholditsecurely;
- Usehisorherotherarmtofirmlygraspthechild’swrist,holdingitpalmdown.
- Preparetheskin : Preparetheskinasdescribedaboveforadultpatients.

###### Preparethelancetand prick the skin

- Hold the heel firmly as shown in figure.Apply moderate pressure near the puncture site. This canbe donebywrappingthe heel usingyourthumbandsecondfinger.
- Clean the site with an alcohol swab. Make sure the site is dry before puncturing the skinwith the lancet.In selecting a puncture site, avoid any areas of the skin that are broken orappeartobeinfected.
- Use the lancet for the skin puncture by placing theblade-slotsurfaceagainsttheareaandpressingthe trigger.Ensure thefreeflowofblood.
- Wipeawaythefirst two drops of blood using asterilegauzepadandcollectthethirddropforhaemoglobintesting.
- Afterbloodcollectioniscomplete,discardallmaterialsusedinthecollectionprocedureinalabelledbiohazardouswaste container(bag).
- Ifnecessary,takethefollowingstepstoimprovetheeaseofobtainingbloodbyfinger-prick inpaediatricandneonatalpatients:
- ask the parent to rhythmically tighten and release the child’s wrist, to ensure that thereissufficientflow ofblood;
- keep the child warm by removing as few clothes as possible, swaddling an infant in ablanket, and having a mother or caregiver hold an infant, leaving only the extremity ofthesiteofcapillarysamplingexposed.
- Avoidexcessivemassagingorsqueezingoffingersbecausethiswillcausehaemolysisandimpedebloodflow.
- Unsuccessfulattemptsinpaediatricpatients

Adhere strictly to a limit on the number of times a paediatric patient may be stuck. If nosatisfactory sample has been collected after two attempts, seek a second opinion to decidewhethertomakea furtherattempt,orcancelthetests.

## **Random Blood Sugar (RBS) Test**

Health investigators should keep in mind thatrandom blood glucose testing is done at any time of the day in combination with other biomarkersthat therespondenthas givenher/his consent.

## The Device: ACCU CHEK Glucometer

## **Disposal of The Waste Material**

After performing the test the lancet should be burnt in needle destroyer and all the biohazard waste should be discarded in the appropriate colour coded biohazard bags following the biomedical waste disposal guidelines

## **Haemoglobin Test**

For haemoglobin test the infants, children, adults (men and women) and old aged people are included in the study**.** Following are the stepwise procedure for conducting haemoglobin test in different age group of people**.**

The Device: Haemoglobinometer and the procedure of measuring haemoglobin as per manufacturer’s guidelines**:**

## **Troubleshooting of TrueHb Haemoglobinometer in Field**

It is advisable that after every 3 testing in the haemoglobinometer the sensor has to be cleaned with a piece of dry tissue paper. For this purpose the black coloured portion of the haemoglobinometer should be pulled and opened to clean the sensor. The process is described as follows:

##
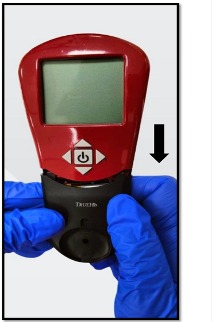

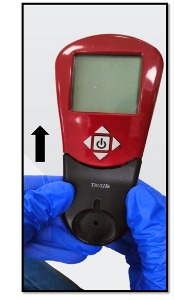


Process for cleaning: -

- Use a damp cloth and clean the black bottom body of the TrueHb Hemometer.
- Remove the bottom body (Black part) by pressing the sides of the TrueHb Hemometer.
- Wipe the lens with a dry cloth. Do not scratch the sensor area.
- Do not use bleach or alcohol to clean the lens and black bottom body of the TrueHb Hemometer.
- Slide the black bottom body back into the TrueHb Hemomete**r**

## **Disposal of The Waste Material**

After performing the test the lancet should be burnt in needle destroyer and all the biohazard waste should be discarded in the appropriate colour coded biohazard bags following the biomedical waste disposal guidelines

## **Sickle Cell Disease/Trait Test**

- For Sickle cell disease/trait test the infants, children, adults (men and women) and old aged people are included in the study**.** Following are the stepwise procedure for conducting haemoglobin test in different age group of people**.**
- The test will be performed after collecting venous blood sample in EDTA tube.
- The details of the blood collection procedure is mentioned in the next section.
- The details of the test is mentioned below:

## The Device: Gazelle Hb variant and the procedure of detecting sickle cell disease/trait as per manufacturer’s guidelines:

## **Disposal of The Waste Materials**

After performing the test the materials and cartridges should be discarded to the appropriate colour coded biohazard bags following the biomedical waste disposal guidelines.

## **General Procedures For Collection of Venous Blood Samples From Adults And Children**

The venous blood samples will be collected from adults and children to test different biochemical markers which reflect abnormal levels of certain substances in the blood. This indicates the disease in the organ or tissue that produces them

## **Preparation and arrangement of equipments**

- Collect all the equipment needed for the procedure and place it in the table.
- A supply of laboratory sample tubes (EDTA tube, gel vacutainer), which should be stored dry and upright in a rack. Prior collecting the blood sample the blood collection tubes.
- Check the barcode labeling in the blood collection tubes, report format and verify the barcode ID before recording the test result.
- Ensure that the rack containing the sample tubes is close to the technician, but away from the patient, to avoid it being accidentally tipped over.

## **Preparation of the Study Participants**

- Before the venepuncture procedure it is important to follow the steps mentioned below:
- Introduceyourselftotheparticipant,andask him/her tostatetheirfullname.
- Askwhethertheparticipanthasallergies,phobiasorhaseverfaintedduringpreviousinjectionsorblooddraws.
- Discuss the test to be performed and obtain verbal consent. The participant has aright to refuse a test at any time before the blood sampling, so it is important to ensure thattheparticipanthasunderstoodtheprocedure.

## Site Selection For Sample Collection

- Extend the participant’s arm and inspect the antecubital fossa or forearm.
- Locate a vein of a good size that is visible, straight and clear.
- The median cubital vein lies between muscles and is usually the most easy to puncture. Under the basilic vein run an artery and a nerve, so puncturing here runs the risk of damaging the nerve or artery and is usually more painful.
- DO NOT insert the needle where veins are diverting, because this increases the chance of a haematoma.
- The vein should be visible without applying the tourniquet. Locating the vein will help in determining the correct size of needle.
- Apply the tourniquet about 4–5 finger widths above the venepuncture site and re-examine the vein.

# Performhandhygieneandputongloves

##### Performhandhygiene by using hand sanitizer.

##### If hands are visibly contaminated, use soap and water for cleaning and dry using a clean towel.

##### Afterperforminghandhygiene,putonwell-fitting gloves.

## Disinfecttheentrysite

- Cleanthesitewitha70% alcoholswabfor30secondsandallowdryingcompletely.
- Applyfirmbutgentlepressure.Startfromthecentreofthevenepuncturesiteandwork downwardandoutwardstocoveranareaof2cmormore.
- Allowtheareatodry.Failuretoallowenoughcontacttimeincreasestheriskof contamination.

DONOTtouchthecleanedsite;inparticular,DONOTplaceafingerovertheveintoguide theshaftoftheexposedneedle.Itthesiteistouched,repeatthedisinfection

- Blood Draw

## Venepuncture

Performvenepunctureasfollows:

- Anchortheveinbyholdingtheparticipant’sarmandplacingathumbBELOWthe venepuncturesite.
- Asktheparticipanttoformafistsotheveinsaremoreprominent.
- Enter the vein swiftly at a 30 degree angle or less, and continue to introduce the needlealongthevein at theeasiestangle ofentry.
- Oncesufficientbloodhasbeencollected,releasethetourniquetBEFOREwithdrawingthe needle. Some guidelines suggest removing the tourniquet as soon as blood flow isestablished,andalwaysbeforeithasbeeninplacefortwominutesormore.
- Withdraw the needle gently and apply gentle pressure to the site with a clean gauze ordrycotton-woolball.Askthepatienttoholdthegauzeorcottonwoolinplace,withthe arm extended and rose. Ask the patient NOT to bend the arm, because doing so causes hematoma.

**Figure : Venepuncture Process**

**
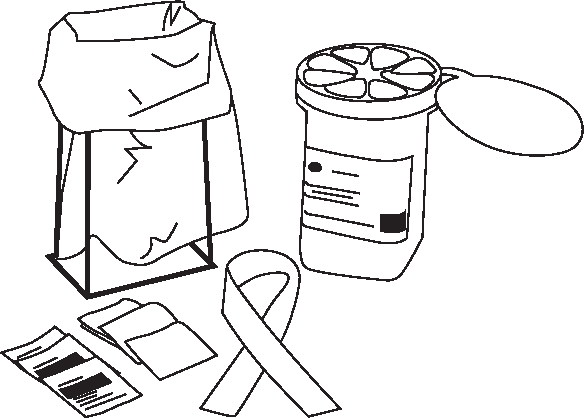

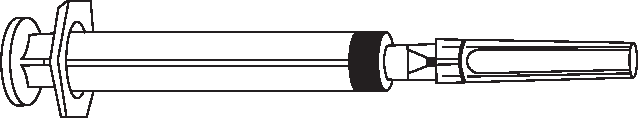
**

1. Assembleequipmentandincludeneedleandsyringeorvacuumtube,dependingonwhichistobeused.
2.
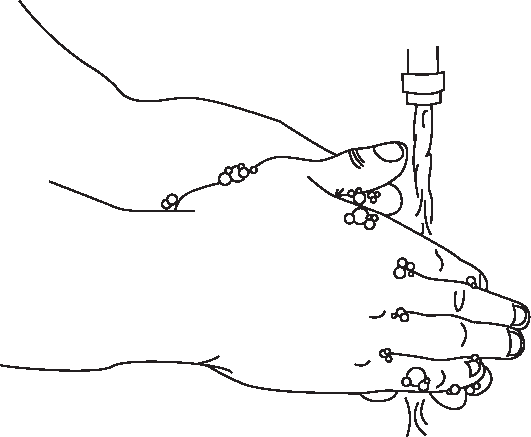
Perform hand heigyne (Use soap and water/Sanitizer) 3. Identify and Prepare patient


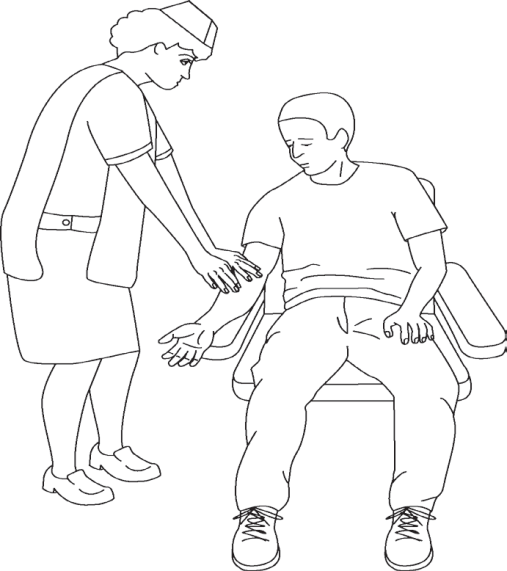


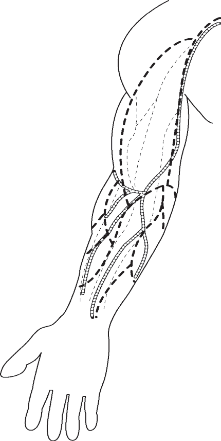


Median cubical vein

Ulner nerve

Ulner artery

Basilic vein

4. Select the site, preferably at the antecubitalarea (i.e. the bend of the elbow). Warming thearmwithahotpack,orhangingthehanddownmay make it easier to see the veins. Palpatetheareatolocatetheanatomiclandmarks.DONOTtouchthesiteoncealcoholorotherantiseptichasbeenapplied.


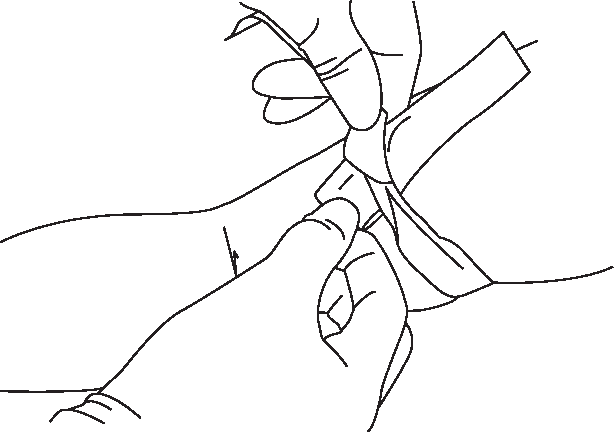


1. Apply a tourniquet, about 4–5 finger widthsabovetheselectedvenepuncturesite.

**
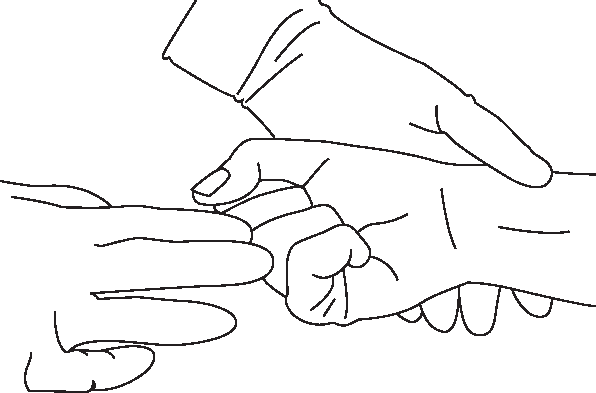

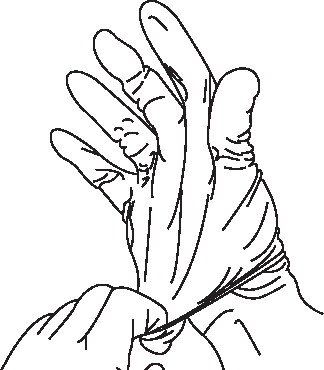
**

1. Ask the patient to form a fistso that the 7. Put on well-fitting gloves

veins are moreprominent


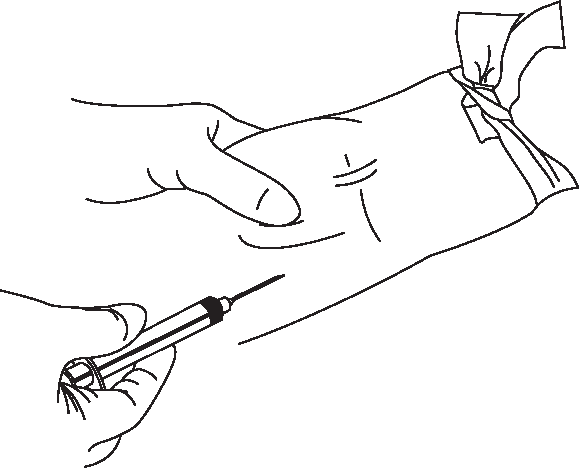

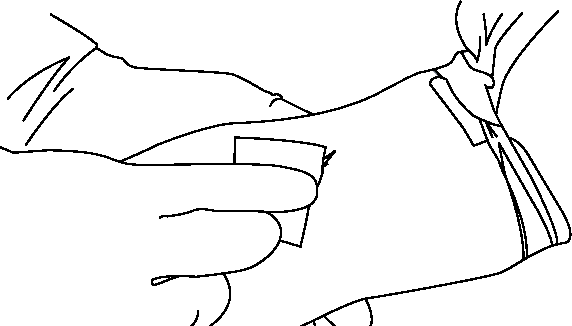


9. Anchor the vein by holdingthepatient’sarm and

8. Disinfect the site using alcohol swab placing a thumb below the venepuncture site for 30 seconds andallowtodrycompletely

10.Enter the vein swiftly at a 30° angle


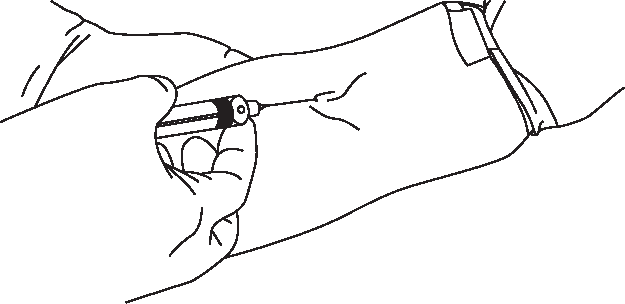


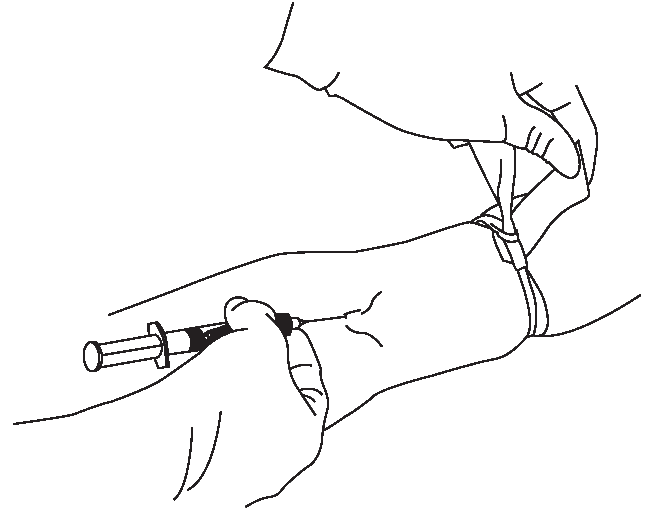


11. Once sufficient blood has been collected release

the tourniquet before withdrawing the needle

12.Withdraw the needle gently and give the patient a clean
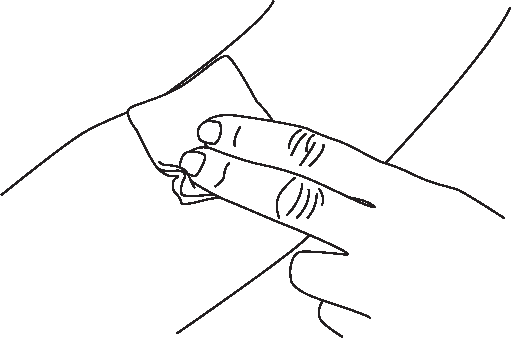
or dry cotton ball to apply to the site with gentle pressure

13. Discard the needle and syringe into a sharp container/burn the needle in needle destroyer


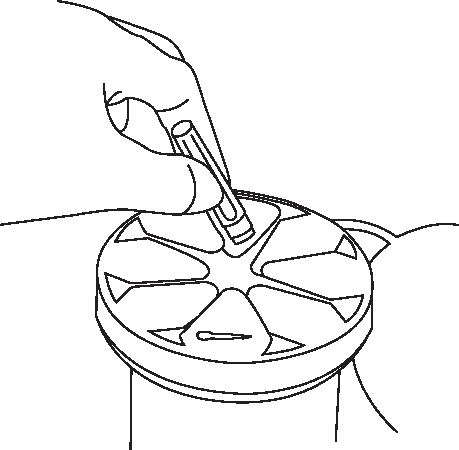


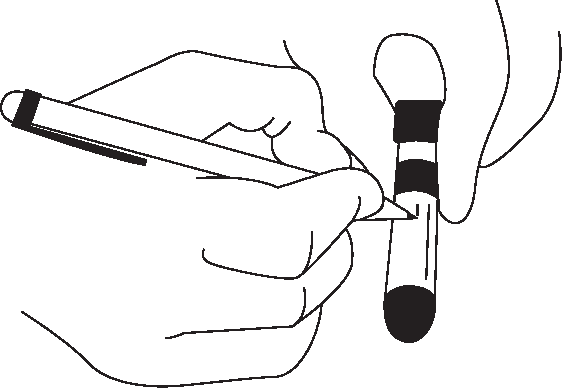
14.Check the label and form for accuracy


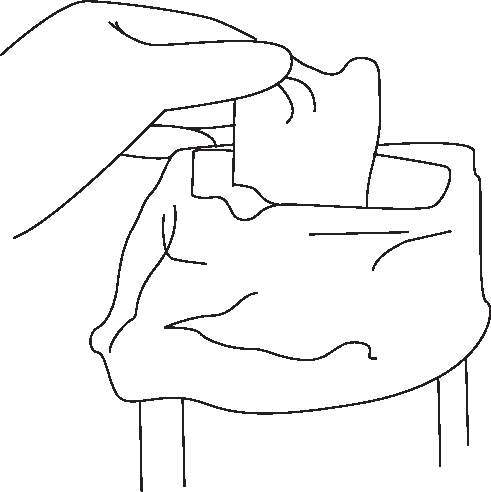
15. Discard sharp and broken items in to the sharp container. Place items

that can drop blood/body fluids in biohazard bags .

16.Perform hand hygiene after removing the gloves

(**Picture Source: WHO Phlabotomy Guidance 1**)

## Transfer of blood from syringe to vial

- Pierce the stopper on the tube with the needle directly above the tube using slow, steadypressure. Do not press the syringe plunger because additional pressure increases the risk ofhaemolysis.
- Where possible, keep the tubes in a rack and move the rack towards you. Inject downwardsinto the appropriate coloured stopper. DO NOT remove the stopper because it will releasethevacuum.

## Order of draw for multiple tube collection

- First approximately 1ml blood should be tranferred to the EDTA (purple cap) tube for testing sickle cell (less amount because it requires only 20µl blood for the testing). Then the rest of the blood should be transferred to the gel vacutainer tube(yellow cap) for serum separation.

## Cleaning ofcontaminatedsurfacesand completion of theprocedure

- Burn the used needle using needle destroyer.
- Check the label in the blood collection tube for accuracy.
- Discard used items into the appropriate category of waste. Items used for phlebotomy that would not release a drop of blood if squeezed (e.g. gloves) may be discarded in the general waste.
- Performhandhygieneagain,asdescribedabove.
- Informtheparticipant whentheprocedureisover.
- Ask the participant how they are feeling. Check the insertion site to verify that it is notbleeding, then thank the patient and say something reassuring and encouraging before thepersonleaves.

## **Sample storage In Field**

All the collected blood samples will be stored in vaccine carrier immediately after collection maintaining cold chain.

## **Coss Checking with Laboratory Tally Sheet**

Before transporting all the samples from field to district headquarter hospital the laboratory tally sheet should be cross checked by the laboratory technician (Refer Annexure 4).

## **Sample transport from field to field laboratory**

The vaccine carrier should be properly closed and transported to the district head quarter hospital laboratory maintain cold chain for further processing.

## **Roles and Responsibilities of Research Assistant (RA) / Field Investigator (FI) before leaving the field**

## The RA should confirm that the laboratory tally sheet is filled up appropriately by the laboratory technician.

- The report format of all the participants are filled with appropriate measurement and testing data and then it should be handed over to the authorized person of ASHA to the respective village.
- All the instruments are switched off and properly cleaned and packed in their respective bags/boxes before leaving the field.
- In absence of RA the above task responsibility will be performed by the FI of the respective team.

## **Sample processing set Up in District Head Quarter Hospital for primary sample processing**

- The sample processing set up will be performed in the assigned laboratory space by the District Health Authority.
- After reaching to the laboratory the RA should contact District Microbiologist for the coordination of sample processing.

### **Materials and Equipments Required:**

- REMI clinical centrifuge
- Cryo vials
- Barcode Stickers
- Pasteur pipette
- Table top waste disposal container/Biohazard Bag
- Tissue paper
- Sanitizer
- Cryo vial box
- Laboratory tally sheet

## **Blood Sample Processing for Serum Separation**

It is important to separate the cellular and liquid portions of a blood specimen as soon as possible when the test requires a sample of serum or plasma. Centrifugal force is used to separate the components of blood like, red blood cells, platelets and plasma from each other.

## **General Procedure for Serum separation**

- Before processing all the collected blood samples must be matched with laboratory tally sheet.
- Assemble the required materials needed to perform the serum separation.
- Wear personal protective wear like mask, gloves, lab coat before processing the sample.
- For serum separation (requires clot time): Whole blood should be allowed to clot and then centrifuged at 2800 rpm for 10 minutes to separate the serum.
- Arrange a rack with sterile cryo vial and label the vials with patient code, date of collection and sample type.
- The serum should be carefully removed with a Pasteur pipette to avoid extracting red cells, and transferred aseptically to the pre labeled sterile cryo vial.
- All the serum containing cryovials will be kept in pre labeled (serial no of cryovials containing sample id, sample type, date of storage, box no) cryo box and stored.
- **Note: Universal Precautions must be used when working with blood. Use of personnel protective equipment is mandatory**.
- Discard the vacutainer in biohazard bag after separating serum/plasma.

## **Storage of samples in field laboratory**

After centrifugation the separated serum will be stored barcode labeled cryo vials in -20°C in the cryo cube boxes serially as per the lab tally sheet until they are transported to RMRC laboratory.

## **Waste Disposal in the Laboratory**

Waste disposal will be done at the sample processing laboratory as per the laboratory SOP instructed by District Microbiologist.

## **Check Points Before Leaving the Laboratory**

Before leaving the laboratory make sure the three steps are done:

- The centrifuge is switched off and the plug is removed.
- The laboratory tally sheet is filled up after serum separation.
- Any spillage on the working table should be cleaned with 70% alcohol.
- Gloves, Tissue paper, used Pasteur pipettes are disposed on biohazard bags.
- Cryo vials are properly labelled with barcode and the barcode is covered in transparent cellotape.
- The samples are stored in proper temperature.
- Gel cool packs are kept in freezer for cooling so that they are frozen while carrying next day to field.
- Inform the District Microbiologist before you leave the laboratory.

## **Roles and Responsibilities**

The primary sample processing and storage of secondary sample (serum) in the laboratory will be performed by LT1 or LT 2 under the supervision of RA/FI.

## **Sample transport to RMRC laboratory at Bhubaneswar**

The serum/plasma samples will be transported from field laboratory to RMRC laboratory by following a triple layer packaging system.

- 1^st^ packaging: after centrifugation the serum/plasma sample will be stored in a labeled cryo vial.
- 2^nd^ packaging: the cryovials will be stored in -20°C in the cryo cube boxes serially as per the lab tally sheet.
- 3^rd^ packaging: the cryo boxes will be further packed in thermocol boxes with gel cool packs and sealed properly.
- The samples should be transported to RMRC Bhubaneswar from each district at least once in a month.

## **Quality Assurance During Sample collection, Processing, Storage and Transport**

- Hand hygiene should be performed before and after collecting the blood samples.
- After collecting the sample the labeling on the collection tube should be matched with the laboratory tally sheet.
- Any adverse event while collecting the sample should be recorded immediately. A log book or a registrar should be maintained for documenting the incident, its possible causes and management.
- The needle stick injury should be reported by the field staff to the higher authority immediately and recorded in a log book mentioning the incident, date and time.
- All infectious waste should be disposed maintain the Biomedical Waste Management guidelines **(Refer BMWM SOP)**
- The storage of the samples should be done maintaining adequate temperature (For blood sample 0-4°C and for serum -20°C ).
- Before transport the samples and the cryo boxes should be checked thoroughly for proper labeling, sealing and must be crosschecked with laboratory tally sheet.
- The transport box must be filled with adequate gel cool packs to maintain the cold chain. After packaging the samples the transport box should be marked with waterproof labeling and sealed properly to maintain the cold chain inside.

## **Roles and Responsibilities**

- The packaging of the serum samples to be transported to RMRC Bhubaneswar will be done by RA and FI.
- LT 1 and LT 2 will assist them for the entire task.
- The field staff (RA and FI) will be responsible for packaging and transporting of the samples to RMRC laboratory. They should thoroughly check that the cryo cube boxes are properly labeled, cyovials are arranged in the cryo cube box in a serial manner maintaining the laboratory tally sheet and should not leak, the transportation box is properly labeled and sealed with adequate ice pack or gel cool packs to maintain the cold chain till it reaches to RMRC laboratory.
- The RA/FI should maintain document (photos, videos ) while packing the samples and record the time when the samples are dispatched.

# **Biomedical Waste Management**

1. **Introduction**

Biomedical Waste, (BMW) or bio wastes are those potential hazardous waste materials, consisting of solids, liquids, sharps, and laboratory waste. Biomedical waste differs from industrial waste. It is mostly from the biological sources or is used in the diagnosis, prevention, or treatment of diseases. The Bio Medical Waste (Handling and Management) Rules, were notified in July 1998.

**Health Hazardous of Biomedical Waste**

The types of waste generated from different health care sectors can impact a higher risk to health. These include infectious waste (15%–25% of total health-care waste), among which are sharps waste (1%), body part waste (1%), chemical or pharmaceutical waste (3%), and radioactive and cytotoxic waste or broken thermometers (less than 1%).

1. **Types Of Waste**

Waste and by-products cover a diverse range of materials. It can be categorized into hazardous and non hazardous waste.

- **Hazardous Waste**
- Infectious waste: waste contaminated with blood and other bodily fluids (e.g., from discarded diagnostic samples), cultures and stocks of infectious agents from laboratory work (e.g., waste from autopsies and infected animals from laboratories), or waste from patients in isolation wards and equipment (e.g., swabs, bandages, and disposable medical devices).
- Pathological waste: human tissues, organs or fluids, body parts, and contaminated animal carcasses.
- Sharps: syringes, needles, disposable scalpels and blades, etc.
- Chemicals: For example, solvents used for laboratory preparations, disinfectants, and heavy metals contained in medical devices (e.g., mercury in broken thermometers) and batteries.
- Pharmaceuticals: expired, unused, and contaminated drugs and vaccines; Genotoxicwaste: highly hazardous, mutagenic, teratogenic, or carcinogenic, such as cytotoxic drugs used in cancer treatment and their metabolites.
- Radioactive waste: such as products contaminated by radionuclides including radioactive diagnostic material or radiotherapeutic materials.
- **Nonhazardous or general waste:**

Waste that does not pose any particular biological, chemical, radioactive, or physical hazard.

- General and office waste: wrapping paper, office paper, cartons, packaging materials including plastic sheets, newspapers and bouquets etc.
- Kitchen waste: leftover food, peels of fruits and vegetables skin and dirty water.

1. **Method Of Waste Management**

- **Waste Minimization:** the production of waste more than requirement is always non acceptable.
- **Segregation:** waste should be segregated in different streams at the point of generation. Segregation at source helps in reducing total cost of disposal, preventing general waste from infectious and reducing chances of infection among health care workers. The segregation of wastes is done as per the guidelines laid by Authorized agency of pollution control board and is defined below:


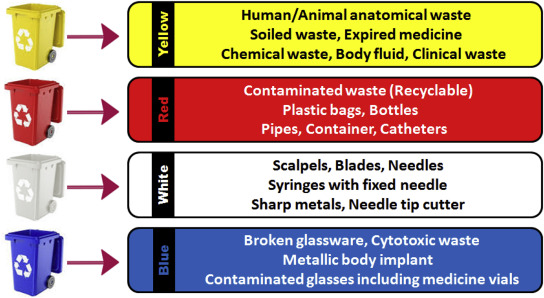


**Figure I: Different colour codes for disposal of infectious waste**

- **Handling and Storage of Bags**
- **Handling:**
- Bags must be replaced when atleast three fourth is full.
- It should be sealed properly.
- The sealed bag should be picked up by its neck.
- Manual handling should be minimized.
- Bags should not be clasped against body.
- Sharp containers should be handled carefully.
- Support from the bottom of the bag should be avoided.
- **Storage:**
- A safe and secured location for storage of segregated biomedical waste in colored bags or containers should be available.
- Secondary handling, recycle, scattering or spillage by animals should not occur.
- Biomedical waste shall not be stored beyond a period of forty eight hours from its generation.
- A system of bar-code and global positioning system for bags or containers.
- Treatment of Biomedical waste inside the laboratory premises
- All the infectious waste of the Laboratory are autoclaved before disposal.

1. **Important Personnel Precautions To Be Taken During Waste Handling**

Proper training has to be provided to all the staff involved in segregation and storage of waste. They should also receive training regarding universal safety precaution. The use of protective equipment should be mandatory for all the personnel handling waste. It can be categorized into two categories: biosafety practices and immunization for the health care workers.

- **Bio safety practices**
- **Gloves**

Heavy duty rubber gloves should be used for waste handling by the waste handlers. The gloves should be washed with soap and disinfectant twice during the handling of the waste (first while wearing and second after removing it). It should be replaced whenever it is teared otherwise can be replaced monthly. The gloves should be kept in hung position whenever not in use.

- **Mask**

Waste handlers should wear mask while sweeping and handling waste.

- **Immunization of the health care worker and waste handler to prevent disease transmission**

All the health care workers and waste handlers whose activities involve contact with patients or with blood/ body fluids (Blood,CSF, Amniotic fluid, Pleural fluid, peritoneal fluid and other body fluids) required for immunization against Hepatitis B vaccine, Tetanus toxoid vaccination and Covid-19 vaccination. Health check up should be conducted during induction and at least once in a year for all these people.

**Management of SPILLS**

- Small Spills (< 10 ml) Cordon the area.
- Bring the spill kit.
- Wear the PPE.
- Cover the spill with tissue paper.
- Pour 1% hypochlorite on the tissue paper.
- Leave it for 15 minutes.
- Pick up the soaked paper with artery forceps and dispose it in yellow bag.
- Mop the are again with 1% sodium hypochlorite.
- Remove the PPE and discard in appropriate bags.
- Replace the spill kit box and keep in designated place.
- Large Spills (>10 ml) Cordon the area.
- Bring the spill kit.
- Wear the PPE.
- Cover the spill with tissue paper.
- Pour 1% hypochlorite on the tissue paper Leave it for 30 minutes.
- Pick up the soaked paper with artery forceps and dispose it in yellow bag.
- Mop the are again with 1% sodium hypochlorite.
- Remove the PPE and discard in appropriate bags.
- Replace the spill kit box and keep in designated place.
- Report the spill incident to the infection control department.

## **Infection Prevention & Control**

**INTRODUCTION**

Infection is the invasion of a host organism’s bodily tissues by disease causing organism, their multiplication and the reaction of host tissues to these organisms and the toxin they produce. Infections which arise in healthcare are termed Healthcare associated infection (HAI).In healthcare settings, germs are found in many places. People are one source of germs including:Patients**,** Healthcare workers**,** Visitors and household members.Infection control prevents or stops the spread of infections in healthcare settings. These practices include standard precautions, decontamination, waste management, surveillance and audit.

1. **PURPOSE OF INFECTION PREVENTION AND CONTROL**

- To ensure establishing a safe procedure of collection of samples from participants.
- To establish standards in prevention, control measures and minimize infection among the field staff and laboratory staff.
- To define procedures for infection prevention and control and to implement them in field as well as in laboratory.

1. **INFECTION CONTROL PROCEDURE AND PRACTICE**

According Centre of Disease Control (CDC) the procedures and practices for infection prevention include:

- Hand hygiene
- Use of personal protective equipment (e.g., gloves, gowns, facemasks), depending on the anticipated exposure
- Respiratory hygiene and cough etiquette
- Management of spillage
- **Hand Disinfection-Aseptic Hygeine Hand Wash**

Hand disinfection with alcohol based hand rub (e.g., 70%alcohol, sterilium) preferably with chlorhexidine and alcohol are practice at least in following condition:

- Whenever touching any patient esp. in inpatient units and critical care areas.
- After handling any potentially infectious object
- Before putting on gloves and after removing them.
- Prior to invasive procedures
- Visibly clean hands
- In high dependency areas and after attending patients in isolation or with known transmissible condition.

Broken skin, cuts and abrasions in any area of exposed skin, particularly the hands and forearms, are covered with a waterproof dressing. Wear gloves if hands are extensively affected. Wrist watches/bracelets are removed.

Alcohol is an effective decontamination agent but should only be used on visibly clean hands. It is also a valuable agent for use, but should only be used 2-3 times consecutively before a hand wash as build up can occur.

- Dispense the required amount of solution onto the hands.
- Ensure solution covers all hand surfaces.
- Rub vigorously, using hand washing technique, until dry.

It is recommended that everyone involved in providing healthcare in the community must be trained in hand decontamination, the use of protective clothing and safe disposal of sharps, and this includes patients and healthcare personnel.

### Hand Care

- Keep nails clean and short.
- Remove rings with stones or ridges.
- Do not wear artificial or gel nails or nail polish.
- When washing hands, wrist watches are removed.
- Sleeves are rolled up to the elbow.
- Nailbrushes should not be used for routine hand washing as they damage the skin and encourage shedding of cells.
- Nailbrushes, where used, must be single use disposable or single use autoclaveable.

***The physical action of washing and rinsing hands under such circumstances is recommended because alcohols, chlorhexidine, iodophors, and other antiseptic agents have poor activity against spores.***

### Hand-hygiene Technique

### When decontaminating hands with an alcohol-based hand rub

- Apply product to palm of one hand and rub hands together,
- Cover all surfaces of hands and fingers by six step technique, **until hands are dry**.
- Follow the manufacturer's recommendations regarding the volume of product to use.

When washing hands with soap and water

- Wet hands first with water
- Apply an amount of product recommended by the manufacturer to hands
- **Rub hands together vigorously for at least 40-60 seconds**
- Cover all surfaces of the hands and fingers by following six step technique
- Rinse hands with water and,
- Dry thoroughly with a **disposable towel/Paper**
- **Use sterile paper towel to turn off the faucet or elbow taps if available**.


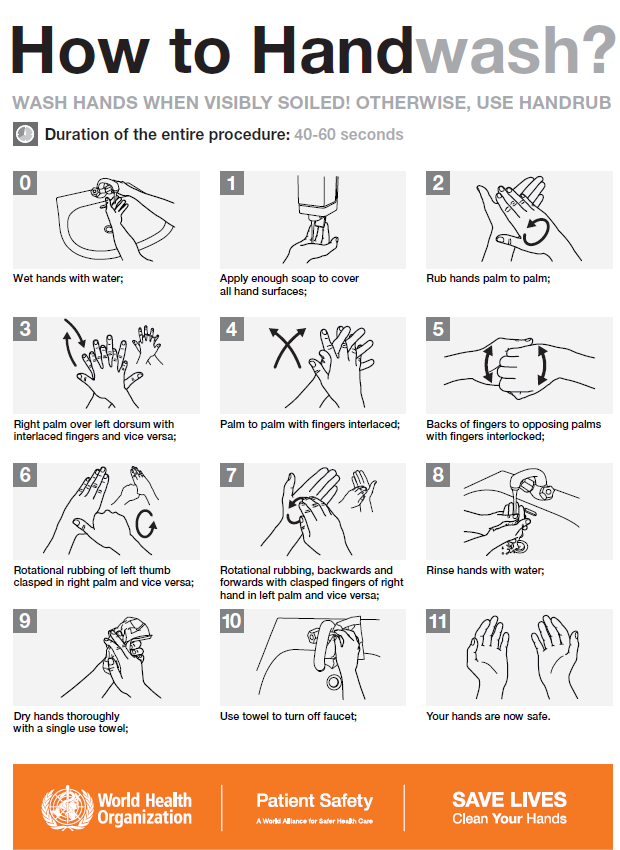


### Re-use/reprocessing of gloves

- As medical gloves are single-use items, glove decontamination and reprocessing are not recommended and should be avoided, even if it is common practice in many health-care settings with low resources and where glove supply is limited.
- At present no standardized, validated and affordable procedure for safe glove reprocessing exists.
- Every possible effort should be made to prevent glove reuse.

## 3.2 Personal Protective Equipment (PPE)

- In determining the type of personal protective equipment to use for a given procedure, the field and laboratory staff should consider the following factors:
- Probability of exposure to blood and body substances;
- Amount likely to be encountered;
- Type of body substance involved; and
- Probable route of transmission of infectious agents
- Full protective wear, including double gloves, protective eye/face-shields, protective footwear and impermeable gowns or aprons, is recommended for operating room or mortuary procedures.

## Gloves

The use of disposable gloves is part of the Standard Precautions concept, which offers consistent guidelines for infection control programmes. As part of personal protective equipment, gloves prevent contact with blood, body fluids, and mucous membranes. They also protect the patient from contamination by the micro-organisms from the wearer’s hands; **gloves are single use items** and are changed after each procedure to further minimize the risk of infection

### Masks

- The type of mask best suited to a particular situation depends on the body substances likely to be encountered and the nature of the activity.

There are two main types of masks used in health care:

- **Surgical masks** *—* fluid-repellent paper filter masks worn during surgical and dental procedures
- **Particulate filter personal respiratory protection devices (P2 respiratory protection devices)** —close fitting masks capable of filtering 0.3-μmparticles and worn when attending patients requiring airborne precautions.
- use.

### Protective foot wear

Protective foot wear should be used when handling biomedical waste as unnoticed cuts and wounds are quite common in the legs. Footwear is also essential to protect legs from ‘sharps’ injury.

**STERILIZATION**

## STERILIZATION

Sterilization is defined as a process where all microbes are removed from a defined object, inclusive of bacterial endospores**.**

**Methods:**

- **Heat Sterilization:**
- **Moist Heat:** Exposure to saturated steam at 121ºC for 15-20 min OR 134ºC for 4 min in any autoclave.

**Recommended Practice:**

### Packing & Loading

For effective sterilization, selection of packaging material plays important role apart from sterilization parameters. The following are keys in selecting a suitable packaging material:

- The packaging material must be permeable to sterilizing agent.
- The packaging material must be impermeable to bacteria and other contaminants.
- The packaging material must resist tears and punctures.
- It should facilitate aseptic presentation of packaged content.

# **MOP TRAVEL**

Vendors for providing vehicles have to be fixed at all the district headquarters where the field teams will be stationed.

Points to be taken into consideration while fixing the vendor:

- Identification of local vendors already engaged in Govt. Projects. (For ease in tendering process)
- 7 seater vehicle needs to be hired for a period of 6-12 months at each field station.
- The vehicle has to report for duty whenever asked to; on all working days.
- Floating up of tendering process in the website of ICMR-RMRC-BB and local newspapers.
- Tendering procedure for the same has to be completed before the field team is being stationed.
- The rates once fixed will be deemed valid for 1 (one) year WEF. date of issue of letter.
- Toll gate & parking charges (extra) will be bear by the party on submission of the bills at the end of the month.
- GST charges (Extra) will be paid by the party as per norms.
- ICMR-RMRC, Bhubaneswar is not responsible for any consequences or damage or liability during the period of hiring of the vehicle.
- The payment will be made within thirty days after submission of the bills.
- The bills should be duly certified by the Principal Investigator, field worker along with duty slip.
- Rate chart strictly followed by the approval rate of competent authority of the ICMR-RMRC, Bhubaneswar.
- Log book of the tour should be maintained properly and duly signed by the traveller and will be submitted to the office for record.
- Up to date vehicle documents like RC Book, Insurance, Fitness, Valid License of the driver, Pollution certificate etc. Should be available in the vehicle.
- Vehicle should always be kept neat and clean.
- The tour program for day/ monthly basis will be communicated by this centre to you for providing vehicle from time to time.
- No fooding and lodging charges will be claimed during the tour.
- All expenses including fuel bills will be paid to the agency by ICMR-RMRC, Bhubaneswar on submission of the bill by the agency at the end of each month.
- All bills will be cleared with due verification by competent authorities ICMR-RMRC, Bhubaneswar.
- Details of Bank Account No., Name of the Bank, Branch Code, IFSC Code and a cancelled cheque along with bills.
- Valid PAN/GST No. should be provided for office record.

| **STATION OF LOCAL VEHICLE VENDORS** | |
| --- | --- |
| **1^ST^ PHASE** | **2^ND^ PHASE** |
| BARIPADA | SAMBALPUR |
| SUNDARGARH | PARLAKHEMUNDI |
| RAYAGADA | MALKANGIRI |
| KORAPUT | NOWRANGPUR |
| BHAWANIPATNA | PHULBANI |
| KEONJHAR |  |

# **MOP ADMINISTRATION**

On Joining

- Required to furnish an undertaking to the effect that no criminal proceedings are either pending or contemplated against the staff in any court of law.
- The engagement is purely on contract basis and the Competent Authority reserves the right to dispense with the contract at any time without assigning any reasons.
- The present assignment is initially for (6 months/ 1 year) from the date of assumption of duty unless subsequently extended.
- The engagement to the position will automatically cease to be on present/extended assignment or completion of the project, whichever is earlier.
- The engagement can be terminated at any point in time by giving one month notice on either side.
- Contract can be terminated forthwith or before expiry of notice period by making payment of a sum equivalent to one month contractual amount. However, the staff will not be permitted to surrender one month contractual amount in lieu of the period of notice of unexpired portion thereof and the staff is required to serve the full period of notice.
- The staff will not be treated as an regular employee of ICMR-RMRC Bhubaneswar.
- The staff will not have any claim on a regular post in ICMR or its institute/ centre or in any Dept of GoI. The staff will give an undertaking to this effect before joining the Project Human Resource Position.
- The staff will be under the administrative control of ICMR-RMRC Bhubaneswar and will be subject to all the rules of ICMR-RMRC Bhubaneswar for Project Human Resource Position.
- The staff will be normally posted at the study site; however maybe temporarily be asked to work at other study sites in the interest of the project work. The staff shall be liable to serve any part of the country.
- The staff is not entitled for any terminal benefit after completion of contract period or otherwise.
- The staff shall not apply for any other outside employment before completion of first six months period under the project.
- After completion of the first six months period under the project, not more than four applications for outside employment shall be forwarded in a year. All applications shall be sent through proper channel not directly.
- The staff has to submit a certificate of physical fitness from civil surgeon/ medical superintendent of a Government Hospital; if fails to submit the same or found unfit in medical examination, the offer to engagement to the Project Human Resource position shall stands cancelled automatically.
- The staff will not divulge any information gathered or outcome of research work during the period of assignment to anyone who is not authorized to have the same.

On working, Attendance & Payments

- - 6 Days a Week Working (Monday to Saturday).
  - Sundays and Public Holidays are Off.
  - Attendance Register Need to be Signed Everyday; which will be with the RA/ PI.
  - Work certificate has to be sent to the central team by 18^th^ of each month from the field; as per norms for salary.
  - Work certificate of central team has to be compiled by 20^th^ of each month along with the field team and be sent to the accounts section for the needful.
  - Payment of stipend or emoluments to the Project Human Resource positions will be subject to availability of funds from the funding agency of the project.

Leave rules as per norms.

- - CCC (Leave) Rules shall not be applicable.
  - Paid leave of absence may be allowed at the rate of 2.5 days for each completed month of engagement or as prescribed by the competent authority from time to time. No other kind of leave shall be admissible.
  - Leave shall not be carried forward beyond one-year contract. Accumulation of leave beyond a calendar year shall not be allowed.
  - On termination of the contract, you shall not be entitled to benefit of encashment of unveiled portion of leave.
  - In event of leave without prior permission of PI/ Guide/ Head of Institute and/ or any willful unauthorized absence, the contract shall be ceased automatically and the concerned person holding Project Human Resource Position will not be permitted to resume duty without prior permission of Head of the Institute.

Allowances

- - Project Human Resource staffs are not entitled to any other allowance such as dearness allowance, transport allowance, LTC, Bonus, etc.
  - They will not be provided any medical facility under CGHS or CS (MA) Rules.
  - No travelling and/or daily allowance will be admissible either for joining the assignment or on expiry of the contract.
  - Travelling in connection with assigned work during the period of engagement, staff will be entitled to draw TA/DA in accordance with emoluments, but it will not be at par with regular/ permanent employees of ICMR.
  - Eligibility for availing TA/DA as per norms.
  - Tour Approval as per norms.
- Logistics consumption register has to be maintained on day to day basis.
- Indent for the regular supply of consumables has to be made well in advance for uninterrupted supply of materials.
- Any malfunctioning of instruments has to be reported immediately; so that it can be addressed at the earliest possible.
- Vehicle movement log book has to be maintained on day to day basis.

# **MOP ROLES & RESPONSIBILITIES OF STAFFS**

**RESPONSIBILITY OF ICMR-RMRC, BHUBANESWAR**

- Implementation of the project as per the approved timeline
- Constitute and convene meetings of a steering committee with representation from health, tribal affairs and other relevant departments/institutions for monitoring the progress of the project.
- The Principal Investigator or a person duly authorized by her will be the contact person for all the compliances.
- The institution will submit phase wise progress report as per the following deliverables:
- Formative phase including project unit set up, staff hiring, data collection tool preparation, data base management system (DBMS) establishment, manual of operating procedures development, procurement of equipments.
- Midpoint of data collection report
- Final report.
- ICMR-RMRC, Bhubaneswar will obtain necessary Ethical Committee approvals before implementation of the project.
- The institution will submit the SOE and UC at the end of the project period.
- The Principal Investigator will submit the soft and hard copy of the final report to SCSTRTI, Bhubaneswar at the end of the project.
- Study findings may be published jointly after due approval from SCSTRTI, Bhubaneswar.
- SO, Micro Project and CDMPHO of the respective districts should be informed about the field visits ahead of the schedule to ensure support by ITDA department.
- The data and the report will be the property of SCSTRTI, Bhubaneswar and ICMR-RMRC, Bhubaneswar will take permission of SCSTRTI for further use as and when needed.

**ROLE OF CENTRAL TEAM IN FIELD**

- Will be visiting each of the six fields every month for monitoring and cross verification of the data collected.
- Will be helping out field team to solve local issues if any faced.
- Will be helping out field team in training as and when required.
- Will be taking care of the regular supply of consumables to the field team.
- Will be taking care of transport of samples collected from the field to ICMR-RMRC central laboratory.

**Programme Manager**

- The program manager is responsible for developing the plan which specifies how the projects goals will be met, and distributing it to stakeholders.
- The day to day activities of the project require strong leadership from the project management team.
- The programme manager must initiate changes needed based on field experience and seek approval from the applicable stakeholders.
- Monitoring the project budget and schedule status
- Dividing the project into tasks.
- The programme manager must communicate the expected completion date to the applicable stakeholders, as well as any milestone dates.
- Monitoring schedule progress
- Determining the project team roles and responsibilities: The programme manager must assign job descriptions and determine what skill sets are required to achieve the projects goals with minimal cost.
- Developing the project team
- Motivating the project team
- Co-ordinate the Liaising with district administration and health department.
- Acquiring the project resources: The project resources must be acquired at a reasonable cost, and be available when the project needs them.
- Monitoring and controlling project resources: Many project resources have escalating cost provisions, or get consumed at a planned rate, especially those which are outsourced.
- Preventing unauthorized scope change (scope creep) It is ridiculously easy to let people add things to the project that were never planned, especially small items that appear to be insignificant.
- Identifying stakeholders: The project stakeholders are the judge and jury who determine the success or failure of the project,
- Managing stakeholder expectations
- Communicating with stakeholders: The programme manager must maintain a stakeholder register and perform the required communication to ensure that stakeholders are kept informed and make the necessary decisions.
- Quality control during project execution, the programme manager ensures that the quality level of the end products meets the specifications determined during project planning.
- Identifying risks: All projects have risks which, if they occur, affect the projects success factors. The better these risks are identified, the better they can be anticipated and managed.
- Analyzing risks: Some risks are more important than others. Strong risk analysis ensures the project manager is focused on the things that can trip up a project.
- Developing risk response plans The most important risks should have a risk response plan drawn up

**Project Scientist C: Public Health**

- Plan the implementation framework of the study with quality assurance through monitoring and supervision.
- Liaise with district administration and health department.
- Sampling framework preparation.
- Carry out pilot studies to check for feasibility of the tool and team.
- Developing the project team
- Motivating the project team
- Monitoring of field data collection.
- Monitoring of DBMS.
- Monitoring schedule progress
- Scientific report preparation.
- Any other responsibilities assigned by PI of the project.

**Project Scientist C: Bio-Chemistry**

- To Prepare Standard Operating Procedures for different Laboratory Tests.
- To prepare list of laboratory tests to be performed at central lab and in the field.
- To make indent for procurement of consumables and non-consumable items to be used in the Central Laboratory and Field.
- To Set up Central Laboratory Facility at ICMR-RMRC, Bhubaneswar.
- To standardize all the equipments/ instruments.
- To Train the Laboratory Technicians and Research Assistant (Laboratory).
- To carry out Laboratory Tests.
- To monitor sample collection, processing and storage in the field.
- To monitor transportation of samples from the field to the storage facility and from the storage facility to central laboratory at ICMR-RMRC, Bhubaneswar.
- Preparation of the reports of the tested samples.
- To monitor daily uploading of reports in to the Data Base Manage System (DBMS).
- To ensure uninterrupted supply of consumables to the field team and in the central laboratory.
- To perform internal audit for the utilization of the consumables at the field and central laboratory.
- To ensure regular maintenance of instruments.
- To make arrangements for repairing of any equipments/ instrument in case of its break down at the earliest possible.

**Project Scientist C: Statistics**

- Tool development for data collection.
- Development of tools manual.
- Data base management with cleaning and analysis.
- Error Checks.
- Data Quality Monitoring.
- Real time data quality check.
- Course preparation.
- Raw data preparation.
- Data Analysis.
- Making Standard operating Procedures for Data Quality, Data flow, Data Base Management System (DBMS).
- Use and functionality of DBMS.
- Development of DBMS dashboard indicators and their daily monitoring
- Daily monitoring of DBMS.
- Preparing report and scientific paper.
- Any other responsibilities assigned by PI of the project.

**Field Supervisor**

- Implementation of field data collection, training and handholding of the field workers in data collection process.
- Ready to reside at study and Travel extensively in the field area.
- Monitor data collection and perform quality checks.
- Coordination with the team and stakeholders involved in data collection activities.
- Rapport build-up local Govt. Officials and local representatives.
- To Make a directory of all District level, Block Level and village level officials along with field workers (ASHAs/ ANMs etc.
- Any other task assigned by the core team.

**Field Investigator**

- Field data collection.
- Entering of data into the software/Tablet.
- Ready to reside at study and Travel extensively in the field area.
- Coordination with the team and stakeholders involved in data collection activities.
- Work in coordination with local Govt. Officials and local representatives.
- Any other task assigned by the core team.

**Laboratory Technician**

- Ready to reside at study site and prepared for extensive field visits.
- Collection of venous blood, processing and storage in the field.
- Able to maintain cold chain protocol of sample transport and storage.
- Knowledge about safe disposal of Biomedical wastes in the field.
- Entering of data into the software/Tablet.
- Any other task assigned by the core team.

**Multi Tasking Staff**

- To help study related field/laboratory activities.
- To carry out the instructions given by the senior project staffs.

#

# **MOP ROLE AND FUNCTION OF SCSTRTI**

1. Provide intellectual support to RMRC and contribute in designing the project and guidance to carry out the work.
2. Issue necessary administrative request to the collectors/DM, PA-ITDA, Deans of Medical Colleges of the state and CDMPHO of the concerned districts to provide required support for data collection as per the study design by the research team.
3. Release Funds as per criteria agreed after submission of the phase wise report, SOE and recommendation of the technical committee.
4. Providing statistical data regarding distribution of different tribal communities along different blocks.
5. Provide necessary support system in the field.
6. Liaising with district officials of the working for different tribal affairs in the district. (ITDA, District Welfare Officer, Block Extension Officer etc.)
7. External Monitoring of the field work.
8. Organize phase end review of the project implementation in presence of the Principal Investigator and both institution heads.

# **MOP LOCAL LIAISON**

- Letters to Collectors of all districts regarding support for the team
- Letters to CDMOs for support, liaising and storage space for samples, Bio-medical waste management.
- Letters to SC&ST department for village list, support of District welfare officers, and local staff support.
- Rapports build up with village heads.
- Involving ASHAs and ANMs working in each village to reach the tribal household and acquire village/ cluster level information.
- Identification of NGOs working in the area; for support in rapport build up.
- Provision of reimbursement of ASHAs and ANMs for their co-operation in the field.
- Making a directory of all District level, Block Level and village level officials along with field workers (ASHAs/ ANMs etc.)

# **ORGANOGRAM & HR**

# **Sexual Harassment**

Sexual harassment will not be tolerated during OTFHS. By sexual harassment, we mean unwelcome sexual advances, requests for sexual favours, and other sexual comments or actions that make the receiver feel offended or intimidated. Sexual harassment may hurt work performance, and in some cases, an individual may feel that they must comply with the unwelcome advances or requests in order to keep their job. Sexual harassment can be committed by a man towards a woman, by a woman towards a man, or between two individuals of the same gender. To avoid any appearance of sexual harassment, individuals should be careful to avoid unnecessary physical contact and suggestive language and should maintain a professional work climate at all times. Anyone who feels that he or she has been the target of sexual harassment or who has witnessed an apparent incident of harassment should immediately report the incident to his or her supervisor, or to the survey manager. The implementing agency is required to investigate the claim and keep reports confidential to the extent possible. The implementing agency must take actions to prevent and correct harassing behaviour. These actions can include changing the workspace, reassigning interviewers or supervisors to different teams and other disciplinary actions. Retaliation against individuals filing complaints of sexual harassment will also trigger disciplinary action.

## Social Media Policy

- No photo of field survey to be uploaded by team members on the social media without permission. Institutional Nodal Officer will upload or release any photo through official media.
- As per ICMR policy, the team members will not interact or provide information to media personnel. Any information inquired by the media is to be provided with the contact details of the managing Principal Investigator of the project or ICMR-RMRC nodal person.
- Any deviation from the policy as mentioned above will be strictly handled, and appropriate disciplinary action will be taken against them.

# **Annexures**

1. Annexure-1

| **Sl No** | **Name of the Tribe** | **Total Population** | Sample size | Households |
| --- | --- | --- | --- | --- |
| 1 | **Chenchu** | **13** | **13** | 4 |
| 2 | **Mankidi** | **31** | **31** | 8 |
| 3 | **Ghara** | **195** | **167** | 42 |
| 4 | **Baiga** | **338** | **228** | 57 |
| 5 | **Desua Bhumij** | **404** | **249** | 63 |
| 6 | **Korua** | **499** | **273** | 69 |
| 7 | **Birhor** | **596** | **292** | 73 |
| 8 | **Gandia** | **1854** | **389** | 98 |
| 9 | **Mankirdia** | **2,222** | **399** | 100 |
| 10 | **Madia** | **2,243** | **400** | 100 |
| 11 | **Kharwar** | **2265** | **400** | 100 |
| 12 | Hill Kharia, Mankirdia and Birhor Development Agency, Jashipur, Mayurbhanj | 3079 | **415** | 104 |
| 13 | Chuktia Bhunjia | 3086 | **415** | 104 |
| 14 | **Rajuar** | **3,518** | **420** | 105 |
| 15 | **Kol** | **4058** | **425** | 107 |
| 16 | **Kawar** | **5225** | **432** | 108 |
| 17 | **Koli, Malhar** | **6,423** | **437** | 110 |
| 18 | **Kotia** | **7,232** | **440** | 110 |
| 19 | **Bagata** | **8813** | **444** | 111 |
| 20 | **Didayi** | **8890** | **444** | 111 |
| 21 | **Parenga** | **9,445** | **445** | 112 |
| 22 | **Tharua** | **9,451** | **445** | 112 |
| 23 | **Kolah Loharas,** | **9,558** | **445** | 112 |
| 24 | Dongria Kondh | 9659 | **445** | 112 |
| 25 | **Lodha** | **9,785** | **445** | 112 |
| 26 | **Pentia** | **10,003** | **520** | 130 |
| 27 | **Binjhia, Binjhoa** | **11419** | **522** | 131 |
| 28 | **Bondo Poraja** | **12231** | **523** | 131 |
| 29 | **Bhunjia** | **12350** | **523** | 131 |
| 30 | **Kulis** | **13,689** | **524** | 131 |
| 31 | **Jatapu** | **14890** | **526** | 132 |
| 32 | **Dharua** | **18151** | **528** | 132 |
| 33 | **Banjara,Banjari** | **18257** | **528** | 132 |
| 34 | **Mahali** | **18,625** | **528** | 132 |
| 35 | **Kondadora** | **20,802** | **529** | 133 |
| 36 | **Dal** | **25598** | **531** | 133 |
| 37 | **Mundari** | **25,655** | **531** | 133 |
| 38 | **Kandha Gauda** | **26403** | **531** | 133 |
| 39 | **Holva** | **28149** | **531** | 133 |
| 40 | **Omanatya** | **28,736** | **531** | 133 |
| 41 | **Matya** | **30,169** | **532** | 133 |
| 42 | Kutia Kondh | 39761 | **533** | 134 |
| 43 | Lanjia Soura | 40913 | **533** | 134 |
| 44 | **Juang** | **47095** | **534** | 134 |
| 45 | **Kora** | **54,408** | **534** | 134 |
| 46 | Paudi Bhuyan | 61303 | **535** | 134 |
| 47 | **Mirdhas** | **75,940** | **535** | 134 |
| 48 | **Ho** | **80608** | **535** | 134 |
| 49 | **Gadaba** | **84689** | **536** | 134 |
| 50 | **Sounti** | **1,12,803** | **1072** | 259 |
| 51 | **Bhumia** | **125977** | **1072** | 259 |
| 52 | **Binjhal** | **137040** | **1072** | 259 |
| 53 | **Koya** | **1,47,137** | **1072** | 259 |
| 54 | **Bathudi** | **217395** | **1073** | 268 |
| 55 | **Kharia, Kharian** | **222844** | **1073** | 268 |
| 56 | **Bhumij** | **283909** | **1074** | 269 |
| 57 | **Bhuiya, Bhuyan** | **306129** | **1074** | 269 |
| 58 | **Kisan** | **331589** | **1074** | 269 |
| 59 | **Oraon** | **3,58,112** | **1074** | 269 |
| 60 | **Paroja** | **3,74,628** | **1074** | 269 |
| 61 | **Bhottada,Dhotada** | **450771** | **1074** | 269 |
| 62 | **Shabar, Lodha** | **5,16,402** | **1074** | 269 |
| 63 | **Saora, Savar,** | **5,34,751** | **1074** | 269 |
| 64 | **Munda,** | **5,58,691** | **1074** | 269 |
| 65 | **Kolha** | **6,25,009** | **1074** | 269 |
| 66 | **Gond, Gondo** | **888581** | **1074** | 269 |
| 67 | **Santal** | **8,94,764** | **1074** | 269 |
| 68 | **Khond, Kond,** | **1627486** | **1075** | 269 |
|  |  | 9622744 | 42048 | 10489 |

Annexure-2

Selected Districts and Codes

| Name of the district | District code | Name of block | Block code | Name of selected village | Cluster code |
| --- | --- | --- | --- | --- | --- |
| SAMBALPUR | SA | Bamra | 01 | Sagara | 01 |
|  |  |  |  | Dehuripada | 02 |
|  |  |  |  | Bamphei | 03 |
|  |  |  |  | Paramanpur | 04 |
|  |  |  |  | Charichuan | 05 |
|  |  |  |  | Badapada | 06 |
|  |  |  |  | Ghughar | 07 |
|  |  |  |  | Parimunda | 08 |
|  |  |  |  | Mahapatrabalanda | 09 |
|  |  |  |  | Turei | 10 |
|  |  | Kuchinda | 02 | Bauriguda(Rupadharbad) | 01 |
|  |  |  |  | Brahmanidei | 02 |
|  |  |  |  | Salebhadi | 03 |
|  |  |  |  | Badmal | 04 |
|  |  |  |  | Kirasasan | 05 |
|  |  |  |  | Dansanadihi | 06 |
|  |  | Jamankira | 03 | Ladampali | 01 |
|  |  |  |  | Ghodabandhuni | 02 |
|  |  |  |  | Godrapada | 03 |
|  |  |  |  | Gariabahal | 04 |
|  |  |  |  | Kasada | 05 |
|  |  |  |  | Jamankira | 06 |
|  |  |  |  | Chinimahul | 07 |
|  |  |  |  | Patrapali | 08 |
|  |  |  |  | Kutab | 09 |
| DEOGARH | DE | Teleibani | 01 | Bhaluguha | 01 |
|  |  |  |  | Salohi | 02 |
|  |  |  |  | Durijungle | 03 |
|  |  |  |  | Kadodihi | 04 |
|  |  |  |  | Prabhasuni | 05 |
|  |  |  |  | Jhaliamara | 06 |
|  |  |  |  | Danardanpali | 07 |
|  |  |  |  | Palkudar | 08 |
|  |  |  |  | Mundagohira | 09 |
|  |  |  |  | Palunipada | 10 |
|  |  |  |  | Dholpada | 11 |
| SUNDARGARH | SU | Hemgir | 01 | Sanarampia | 01 |
|  |  |  |  | Luabahal | 02 |
|  |  |  |  | Buruta | 03 |
|  |  | Subedga | 02 | Lahuraniberna | 01 |
|  |  |  |  | Tedikaha | 02 |
|  |  |  |  | Damkuda | 03 |
|  |  | Ballisankara | 03 | Tileikani | 01 |
|  |  |  |  | Sagjori | 02 |
|  |  |  |  | Budabahal | 03 |
|  |  | Lephipara | 04 | Didigajharan | 01 |
|  |  |  |  | Aunlabahal | 02 |
|  |  | Badagaon | 05 | Siamal | 01 |
|  |  |  |  | Charpali | 02 |
|  |  |  |  | Bhoipali | 03 |
|  |  | Tangarpalli | 06 | Sanbeura | 01 |
|  |  |  |  | Khuntagaon | 02 |
|  |  |  |  | Bandhapali | 03 |
|  |  | Kutra | 07 | Kalijapathar | 01 |
|  |  |  |  | Karmabahal | 02 |
|  |  |  |  | Jharbeda | 03 |
|  |  | Rajgangpur | 08 | Kukudamunda | 01 |
|  |  |  |  | Kesramal | 02 |
|  |  |  |  | Jhagarpur | 03 |
|  |  |  |  | Jaurumal | 04 |
|  |  |  |  | Chhatam | 05 |
|  |  | Sundargarh | 09 | Talsara | 01 |
|  |  |  |  | Lankahuda | 02 |
|  |  | Kuanrmunda | 10 | Dholkana | 01 |
|  |  |  |  | Andali | 02 |
|  |  |  |  | Banki | 03 |
|  |  |  |  | Rampur | 04 |
|  |  |  |  | Teliposh | 05 |
|  |  | Bisra | 11 | Theteiposh | 01 |
|  |  |  |  | Kopranda | 02 |
|  |  |  |  | Barahabas | 03 |
|  |  | Nuagaon | 12 | Jamdarah | 01 |
|  |  |  |  | Kokerama | 02 |
|  |  |  |  | Ghoghia | 03 |
|  |  |  |  | Urmei | 04 |
|  |  | Lathikata | 13 | Lungei | 01 |
|  |  |  |  | Buchahanda | 02 |
|  |  |  |  | Tainsar | 03 |
|  |  |  |  | Manko | 04 |
|  |  |  |  | Hatibandha (CT) | 05 |
|  |  | Bonaigarh | 14 | Adadihi | 01 |
|  |  |  |  | Deoposh | 02 |
|  |  | Lahunipara | 15 | Kendudihi | 01 |
|  |  |  |  | Tankajoda | 02 |
|  |  |  |  | Talbahali | 03 |
|  |  |  |  | Dhokamunda | 04 |
|  |  | Gurundia | 16 | Raniberna | 01 |
|  |  |  |  | Pankadihi | 02 |
|  |  |  |  | Jalei | 03 |
|  |  |  |  | Jharbeda | 04 |
|  |  | Koida | 17 | Sanraksi | 01 |
|  |  |  |  | Choredhara | 02 |
|  |  |  |  | Ganua | 03 |
| Balasore | BA | Nilgiri | 01 | Purunagan | 01 |
|  |  |  |  | Dobati | 02 |
|  |  |  |  | Ajodhya | 03 |
|  |  |  |  | Mainsapata | 04 |
|  |  |  |  | Chatirikhunta | 05 |
|  |  |  |  | Kantabania | 06 |
|  |  |  |  | Jamudiha | 07 |
|  |  |  |  | Dhobasila | 08 |
|  |  |  |  | Sajanagarh | 09 |
|  |  |  |  | Siadimal | 10 |
|  |  |  |  | Bauriora | 11 |
|  |  |  |  | Matiali | 12 |
|  |  |  |  | Arbandh | 13 |
|  |  |  |  | Dalimbapal | 14 |
|  |  |  |  | Bhaunriabad | 15 |
|  |  |  |  | Baunsapala | 16 |
|  |  |  |  | Chandigarh | 17 |
|  |  |  |  | Kunchibania | 18 |
|  |  |  |  | Hatimunda | 19 |
|  |  |  |  | Kishorechandrapur | 20 |
|  |  |  |  | Rissia | 21 |
| Mayurbhanj | MA | Baripada | 01 | Hemchandrapur | 01 |
|  |  |  |  | Sarujharan | 02 |
|  |  | Badasahi | 02 | Managobindapur | 01 |
|  |  |  |  | Dhobadhobani | 02 |
|  |  |  |  | Agual | 03 |
|  |  | Samakhunta | 03 | Badsol | 01 |
|  |  |  |  | GaudRumacolonysahi | 02 |
|  |  | Betanati | 04 | Saitpur | 01 |
|  |  |  |  | Nuagan | 02 |
|  |  |  |  | Dariha SamilHatudi | 03 |
|  |  | Rasgovindpur | 05 | Gambharia | 01 |
|  |  |  |  | Badampur | 02 |
|  |  | Muruda | 06 | Ajana | 01 |
|  |  | Bangiriposi | 07 | Bangriposi | 01 |
|  |  |  |  | Mahulisul | 02 |
|  |  |  |  | Joka | 03 |
|  |  | Saraskana | 08 | Dumurdiha | 01 |
|  |  |  |  | Dighi | 02 |
|  |  | Kuliana | 09 | Sukhuapara | 01 |
|  |  |  |  | Darkholi | 02 |
|  |  |  |  | Dhadipada | 03 |
|  |  | Suliapada | 10 | Jamubadi | 01 |
|  |  | Khunta | 11 | Jadabani | 01 |
|  |  |  |  | Kadapalasa | 02 |
|  |  | Gopabandhunagar | 12 | Sarukana | 01 |
|  |  |  |  | Pathurikata | 02 |
|  |  | Kaptipada | 13 | Jayantipatta Samil Jamudiha | 01 |
|  |  |  |  | Badbadi | 02 |
|  |  |  |  | Talapokhari | 03 |
|  |  |  |  | Bhandar | 04 |
|  |  | Udala | 14 | Nuagaon | 01 |
|  |  |  |  | Khuntapal | 02 |
|  |  | Karanjia | 15 | Kerkera | 01 |
|  |  |  |  | Saradha | 02 |
|  |  | Raruan | 16 | Jamunti | 01 |
|  |  | Joshipur | 17 | Kalasbandha | 01 |
|  |  |  |  | Badajhilli | 02 |
|  |  |  |  | Uttaransa | 03 |
|  |  | Thakurmunda | 18 | Khaparkhai | 01 |
|  |  |  |  | Nischintapur | 02 |
|  |  |  |  | Banamunda | 03 |
|  |  | Sukruli | 19 | Beguniabindha | 01 |
|  |  |  |  | Fulguntha | 02 |
|  |  | Bisoi | 20 | Jodia | 01 |
|  |  |  |  | Arjunbilla | 02 |
|  |  | Bijatola | 21 | Kuali | 01 |
|  |  |  |  | Baliadhipa | 02 |
|  |  | Kusumi | 22 | Chandida | 01 |
|  |  |  |  | Chhanua | 02 |
|  |  | Rairangpur | 23 | Hatia | 01 |
|  |  | Tiring | 24 | Jadunathpur | 01 |
|  |  |  |  | Janghia | 02 |
|  |  | Bahalda | 25 | Bhaleidihi | 01 |
|  |  |  |  | Kumbhirda | 02 |
|  |  | Jamda | 26 | Guduta | 01 |
|  |  |  |  | Dhalpur | 02 |
| Keonjhar | KE | Joda | 01 | Kolhabarapada | 01 |
|  |  |  |  | Kolharaida | 02 |
|  |  |  |  | Daduan | 03 |
|  |  |  |  | HandiBhanga | 04 |
|  |  | Champua | 02 | Satahalia | 01 |
|  |  |  |  | Tolaknanda | 02 |
|  |  |  |  | Moudi | 03 |
|  |  |  |  | Champua | 04 |
|  |  | Jhumpura | 03 | Haridajodi | 01 |
|  |  |  |  | Teliarsala | 02 |
|  |  |  |  | Ukhunda | 03 |
|  |  | Patna | 04 | Kodakhamana | 01 |
|  |  |  |  | Tangarpada | 02 |
|  |  |  |  | Tando | 03 |
|  |  | Ghatagaon | 05 | Jharbeda | 01 |
|  |  |  |  | Paipani | 02 |
|  |  |  |  | Parasurampur | 03 |
|  |  |  |  | Chatia | 04 |
|  |  |  |  | Ghatagaon | 05 |
|  |  | Keonjhar | 06 | Dhanurjayapur | 01 |
|  |  |  |  | Kasira | 02 |
|  |  |  |  | Narasinghapur | 03 |
|  |  |  |  | Tikarapada | 04 |
|  |  |  |  | Gopinathapur(nuagaon) | 05 |
|  |  | Saharpada | 07 | Tando | 01 |
|  |  |  |  | Matiaguni alias gopalpur | 02 |
|  |  |  |  | Belasarei | 03 |
|  |  | Harichandanpur | 06 | Hunda | 01 |
|  |  |  |  | Kanheigola | 02 |
|  |  |  |  | Nipania | 03 |
|  |  |  |  | Bautia | 04 |
|  |  | Telkoi | 07 | Jata | 01 |
|  |  |  |  | Lipinda | 02 |
|  |  |  |  | Ajayapur | 03 |
|  |  | Bansapal | 08 | Uparraiguda | 01 |
|  |  |  |  | Rakam | 02 |
|  |  |  |  | Jaladihi | 03 |
|  |  |  |  | Danla | 04 |
|  |  |  |  | Panasanasa | 05 |
| Gajapati | GA | Guma | 01 | Linga | 01 |
|  |  |  |  | Sukei (Baranga Singi) | 02 |
|  |  | Rayagada | 02 | Padasahi | 01 |
|  |  |  |  | Belapadar | 02 |
|  |  | R. Udayagiri | 03 | Jiranga | 01 |
|  |  |  |  | R. Udayagiri | 02 |
|  |  | Nuagada | 04 | Atarasing | 01 |
|  |  | Mohana | 05 | Alliganda | 01 |
|  |  |  |  | Juba | 02 |
|  |  |  |  | Jamudiha | 03 |
| Kalahandi | KA | Th.Rampur | 01 | Kanjiguda | 01 |
|  |  |  |  | Taragaon | 02 |
|  |  |  |  | Ushamaska | 03 |
|  |  |  |  | Majhigaon | 04 |
|  |  |  |  | Bhitarguma | 05 |
|  |  |  |  | Kumudabahal | 06 |
|  |  |  |  | Tukuguda | 07 |
|  |  |  |  | Badachhatrang | 08 |
|  |  |  |  | Chirka | 09 |
|  |  |  |  | Hadshil | 10 |
|  |  |  |  | Bahadaghat | 11 |
|  |  | Lanjigarh | 02 | Bhaluchanchara | 01 |
|  |  |  |  | Kauguda | 02 |
|  |  |  |  | Leptaguda | 03 |
|  |  |  |  | Lanjigarh | 04 |
|  |  |  |  | Jamchuan | 05 |
|  |  |  |  | Talkalima | 06 |
|  |  |  |  | Dangamundi | 07 |
|  |  |  |  | Hatisal | 08 |
|  |  |  |  | Kumudapadar | 09 |
|  |  |  |  | Sanpajipita | 10 |
| Rayagada | RA | Gunupur | 01 | Labba | 01 |
|  |  | Gudari | 02 | Jamba | 02 |
|  |  | Padmapur | 03 | Nuagan | 03 |
|  |  | Bishamkatak | 04 | Sauraguda | 01 |
|  |  |  |  | Lotaguda | 02 |
|  |  | Muniguda | 05 | Sudepadar | 01 |
|  |  | Chandrapur | 06 | Kudumguda | 01 |
|  |  | Ramnaguda | 07 | Srirampur | 02 |
|  |  | Rayagada | 08 | Kutuli | 01 |
|  |  |  |  | Champi | 02 |
|  |  | Kolnara | 09 | Khuntibadi | 01 |
|  |  |  |  | Salibi | 02 |
|  |  | Kashipur | 10 | Nalachuan | 01 |
|  |  |  |  | Mahantapatakhuri | 02 |
|  |  |  |  | Kucher | 03 |
| Koraput | KO | Jeypore | 01 | Haridaput | 01 |
|  |  |  |  | Dharnahandi | 02 |
|  |  |  |  | Bankobija | 03 |
|  |  |  |  | Kanjeipatraput | 04 |
|  |  | Boriguma | 02 | Dengapadar | 01 |
|  |  |  |  | Gaudaguda | 02 |
|  |  |  |  | Jhilimili | 03 |
|  |  |  |  | Kenduguda | 04 |
|  |  |  |  | Maliguda | 05 |
|  |  | Kotpad | 03 | Guali | 01 |
|  |  |  |  | Dhamonahandi(Dhamanahandi) | 02 |
|  |  |  |  | Nuagam(Thengguda kamalpadar) | 03 |
|  |  |  |  | Chhatarla(Chotorla) | 04 |
|  |  | Boipariguda | 04 | Majhiguda | 01 |
|  |  |  |  | Goudaguda | 02 |
|  |  |  |  | Dandabadi | 03 |
|  |  |  |  | Badiniput | 04 |
|  |  | Kundra | 05 | Bedapaunsi | 01 |
|  |  |  |  | Jhodenga | 02 |
|  |  | Koraput | 06 | Tola | 01 |
|  |  |  |  | Khagodora | 02 |
|  |  | Similiguda | 07 | Lunguri | 01 |
|  |  |  |  | Kudi | 02 |
|  |  | Pottangi | 08 | Sankar | 01 |
|  |  |  |  | Sambai | 02 |
|  |  |  |  | Galigapadar(Galigabadar) | 03 |
|  |  | Nandapur | 09 | Akanta | 01 |
|  |  |  |  | Hatibari | 02 |
|  |  |  |  | Nandigan | 03 |
|  |  | Dasmanthpur | 10 | Angarguda | 01 |
|  |  |  |  | Tikiriguda | 02 |
|  |  |  |  | Kankadaput | 03 |
|  |  | Lamtaput | 11 | Bodapoda | 01 |
|  |  | Narayanpatna | 12 | Dhaigura | 01 |
|  |  |  |  | Bichlachua | 02 |
|  |  |  |  | Bhasamjhola | 03 |
|  |  | Laxmipur | 13 | Minapai | 01 |
|  |  |  |  | Uskabhata | 02 |
|  |  |  |  | Bhitaragada | 03 |
|  |  | Bandhugaon | 14 | Kuntesu | 01 |
|  |  |  |  | Kunjari | 02 |
| Malkangiri | MK | Malkangiri | 01 | Tangapali | 01 |
|  |  |  |  | Jhileruguda | 02 |
|  |  |  |  | Pandiripani | 03 |
|  |  |  |  | Dengaguda | 04 |
|  |  |  |  | Simagudi | 05 |
|  |  |  |  | Limaguda | 06 |
|  |  | Korkunda | 02 | Bankiguda | 01 |
|  |  |  |  | Matapaka | 02 |
|  |  |  |  | Chidupali | 03 |
|  |  |  |  | Gurakhunta | 04 |
|  |  |  |  | Silaiput | 05 |
|  |  |  |  | Rajal konda | 06 |
|  |  |  |  | Nilakhamar | 07 |
|  |  |  |  | Badali | 08 |
|  |  | Kalimela | 03 | Padganpalli | 01 |
|  |  |  |  | Bapanpalli | 02 |
|  |  |  |  | Singabaram | 03 |
|  |  |  |  | Tigal | 04 |
|  |  |  |  | Pendikonda | 05 |
|  |  |  |  | Nilakota | 06 |
|  |  | Podia | 04 | Nunurguda | 01 |
|  |  |  |  | Semilibencha | 02 |
|  |  |  |  | Alma | 03 |
|  |  | Khairput | 05 | Govindapali | 01 |
|  |  |  |  | Kuriguda | 02 |
|  |  |  |  | Andrahal | 03 |
|  |  |  |  | Sikhapali | 04 |
|  |  | Kudumuluguma | 06 | Nakamamudi | 01 |
|  |  |  |  | Papermenta | 02 |
|  |  |  |  | Sunagoi | 03 |
|  |  |  |  | Darlabeda | 04 |
|  |  |  |  | Dangakunda | 05 |
|  |  | Mathili | 07 | Bhandaripangam | 01 |
|  |  |  |  | Tangaguda | 02 |
|  |  |  |  | Tulasi | 03 |
|  |  |  |  | Patraput | 04 |
|  |  |  |  | Kansariput | 05 |
|  |  |  |  | Sarangapali | 06 |
|  |  |  |  | Mundaguda | 07 |
|  |  |  |  | Dangarkhali | 08 |
| Nowrangpur | NO | Nowrangpur | 01 | Chatahandi | 01 |
|  |  |  |  | Bikrampur | 02 |
|  |  | Tentulikhunti | 02 |  | 01 |
|  |  | Papadahandi | 03 | Khajuri | 01 |
|  |  |  |  | Tumberla | 02 |
|  |  |  |  | Saraguda | 03 |
|  |  | Nandahandi | 04 | Dongarbheja | 01 |
|  |  |  |  | Usaripadar | 02 |
|  |  | Kosagumuda | 05 | Ekori | 01 |
|  |  |  |  | Karchamal | 02 |
|  |  |  |  | Pikaddhanua | 03 |
|  |  |  |  | Umargan | 04 |
|  |  | Raighar | 06 | Maripara | 01 |
|  |  |  |  | Budrapada | 02 |
|  |  |  |  | Marangapalli | 03 |
|  |  |  |  | Chalanpara | 04 |
|  |  |  |  | Khuduku | 05 |
|  |  |  |  | Sargiguda | 06 |
|  |  | Umerkote | 07 | Tohara | 01 |
|  |  |  |  | Umargan | 02 |
|  |  |  |  | Saraguda | 03 |
|  |  |  |  | Bamodeibhatta | 04 |
|  |  | Chandahandi | 08 | Kodobhata | 01 |
|  |  |  |  | Bandhokona | 02 |
|  |  | Jharigaon | 09 | Tentuligan | 01 |
|  |  |  |  | Ichhapur | 02 |
|  |  |  |  | Badatemara | 03 |
|  |  |  |  | Siunaguda | 04 |
|  |  | Dabugaon | 10 | Patia | 01 |
|  |  |  |  | Bodo-olma | 02 |
| Kandhamal | KD | Balliguda | 01 | Badrukia | 01 |
|  |  | K.Nuagaon | 02 | Nuagan | 02 |
|  |  | Daringibadi | 03 | Simanbadi | 03 |
|  |  | Tumudibandh | 04 |  | 04 |
|  |  | Kotagarh | 05 | Kotagada | 05 |
|  |  | Tikabali | 06 |  | 06 |
|  |  | G.Udayagiri | 07 | Katingia | 07 |
|  |  | Raikia | 08 |  | 08 |
|  |  | Chakapada | 09 |  | 09 |
|  |  | Phulbani | 10 |  | 10 |
|  |  | Phiringia | 11 | Senisuga | 11 |
|  |  | Khajuripada | 12 | Dadapaju | 12 |
| Nuapada | NU | Komna | 01 | Salepada | 01 |
|  |  |  |  | Chhinmundi | 02 |
|  |  |  |  | Cherchuan | 03 |
|  |  |  |  | Micchapali | 04 |

Annexure-3

| Household attempt form | | | | | | | | |
| --- | --- | --- | --- | --- | --- | --- | --- | --- |
| Sl No. | Household Id | Individual Id | Data Collected | | | Blood sample collected | | |
|  |  |  | 1^st^ attempt | 2^nd^ attempt | 3^rd^ attempt | 1^st^ attempt | 2^nd^ attempt | 3^rd^ attempt |
|  |  |  |  |  |  |  |  |  |
|  |  |  |  |  |  |  |  |  |
|  |  |  |  |  |  |  |  |  |
|  |  |  |  |  |  |  |  |  |
|  |  |  |  |  |  |  |  |  |
|  |  |  |  |  |  |  |  |  |
|  |  |  |  |  |  |  |  |  |
|  |  |  |  |  |  |  |  |  |
|  |  |  |  |  |  |  |  |  |
|  |  |  |  |  |  |  |  |  |
|  |  |  |  |  |  |  |  |  |
|  |  |  |  |  |  |  |  |  |
|  |  |  |  |  |  |  |  |  |
|  |  |  |  |  |  |  |  |  |
|  |  |  |  |  |  |  |  |  |
|  |  |  |  |  |  |  |  |  |
| Filled by | | | | | | | | |
| Verified by- | | | | | | | | |

Annexure-4

| **Household Listing** | | | | | | |
| --- | --- | --- | --- | --- | --- | --- |
| Sl No. | Name | Age | Gender | Household ID | Individual ID | Sample ID |
|  |  |  |  |  |  |  |
|  |  |  |  |  |  |  |
|  |  |  |  |  |  |  |
|  |  |  |  |  |  |  |
|  |  |  |  |  |  |  |
|  |  |  |  |  |  |  |
|  |  |  |  |  |  |  |
|  |  |  |  |  |  |  |
|  |  |  |  |  |  |  |
|  |  |  |  |  |  |  |
|  |  |  |  |  |  |  |
| Filled by | | | | | | |
| Verified by- | | | | | | |

Annexure-4

| Sample Log book | | | | | |
| --- | --- | --- | --- | --- | --- |
| Sl No. | Household ID | Individual ID | Sample ID | Sample Collected | Serum Collected |
|  |  |  |  |  |  |
|  |  |  |  |  |  |
|  |  |  |  |  |  |
|  |  |  |  |  |  |
|  |  |  |  |  |  |
|  |  |  |  |  |  |
|  |  |  |  |  |  |
|  |  |  |  |  |  |
|  |  |  |  |  |  |
|  |  |  |  |  |  |
|  |  |  |  |  |  |
|  |  |  |  |  |  |
| Filled by | | | | | |
| Verified by- | | | | | |

Annexure-5

| Travel Log Book | | | | | |
| --- | --- | --- | --- | --- | --- |
| Sl No. | Date | Place | Starting time | Ending time | Total KM |
|  |  |  |  |  |  |
|  |  |  |  |  |  |
|  |  |  |  |  |  |
|  |  |  |  |  |  |
|  |  |  |  |  |  |
|  |  |  |  |  |  |
|  |  |  |  |  |  |
|  |  |  |  |  |  |
|  |  |  |  |  |  |
|  |  |  |  |  |  |
|  |  |  |  |  |  |
|  |  |  |  |  |  |
| Filled by | | | | | |
| Verified by- | | | | | |

Annexure-6

| Cluster Plan | | | | | | |
| --- | --- | --- | --- | --- | --- | --- |
| Sl No. | Name of the cluster | Week-1 | Week-2 | Week-3 | Week-4 | Contact Person |
|  |  |  |  |  |  |  |
|  |  |  |  |  |  |  |
|  |  |  |  |  |  |  |
|  |  |  |  |  |  |  |
|  |  |  |  |  |  |  |
|  |  |  |  |  |  |  |
|  |  |  |  |  |  |  |
|  |  |  |  |  |  |  |
|  |  |  |  |  |  |  |
|  |  |  |  |  |  |  |
|  |  |  |  |  |  |  |
|  |  |  |  |  |  |  |

Annexure- 7

| **DISTRICT NAME-** | | | | | | | | |
| --- | --- | --- | --- | --- | --- | --- | --- | --- |
|  |  |  | **MONTH-1** | | **MONTH- 2** | | **MONTH- 3** | |
| **Sl no.** | **Consumables** | **Total Quantity Supplied** | **USED** | **LEFT** | **USED** | **LEFT** | **USED** | **LEFT** |
| 1 | Surgical Mask |  |  |  |  |  |  |  |
| 2 | N -95 |  |  |  |  |  |  |  |
| 3 | Tourniquet |  |  |  |  |  |  |  |
| 4 | Syringe (5 ml ) |  |  |  |  |  |  |  |
| 5 | Syringe (2 ml) |  |  |  |  |  |  |  |
| 6 | Gel Vacutainer (5 ml) |  |  |  |  |  |  |  |
| 7 | EDTA Tube (2 ml) |  |  |  |  |  |  |  |
| 8 | Alcohol Swab |  |  |  |  |  |  |  |
| 9 | Band Aid |  |  |  |  |  |  |  |
| 10 | Rubber band / Zip Lock |  |  |  |  |  |  |  |
| 11 | Face Shield |  |  |  |  |  |  |  |
| 12 | Marker Pen |  |  |  |  |  |  |  |
| 13 | White Gloves (medium) |  |  |  |  |  |  |  |
| 14 | White Gloves (Large) |  |  |  |  |  |  |  |
| 15 | Purple Gloves (medium) |  |  |  |  |  |  |  |
| 16 | Purple Gloves (Large) |  |  |  |  |  |  |  |
| 17 | Biohazard poly (Red & Yellow) |  |  |  |  |  |  |  |
| 18 | Sanitizers |  |  |  |  |  |  |  |
| 19 | Cotton |  |  |  |  |  |  |  |
| 20 | Vial Stand |  |  |  |  |  |  |  |
| 21 | Thermocol Box |  |  |  |  |  |  |  |
| 22 | Vaccine container |  |  |  |  |  |  |  |
| 23 | Gel pack |  |  |  |  |  |  |  |
| 24 | Consent Form |  |  |  |  |  |  |  |
| 25 | File |  |  |  |  |  |  |  |
| 26 | Vial box |  |  |  |  |  |  |  |
| 27 | Cryovial |  |  |  |  |  |  |  |
| 28 | Tissue Roll |  |  |  |  |  |  |  |
| 29 | Stamp pad |  |  |  |  |  |  |  |
| 30 | Apron |  |  |  |  |  |  |  |
| 31 | Handwash |  |  |  |  |  |  |  |
| 32 | Notepad |  |  |  |  |  |  |  |
| 33 | Pen Packet |  |  |  |  |  |  |  |

**Annexure-7**

| **Monitoring checklist** | | | |
| --- | --- | --- | --- |
| Sl No. | Description | Yes/No | Remark |
| 1 | Measurement of height bare foot |  |  |
| 2 | Measurement of weight bare foot |  |  |
| 3 | Consent form completed prior to collection of sample |  |  |
| 4 | Hand sanitized before the collection of blood sample |  |  |
| 5 | Biomedical waste properly disposed in the biohazard bags |  |  |
| 6 | The gel vacutainers are properly placed on the collection table in the vial stand |  |  |
| 7 | The gel vacutainer is properly stored in the vaccine carrier |  |  |
|  |  |  |  |

**
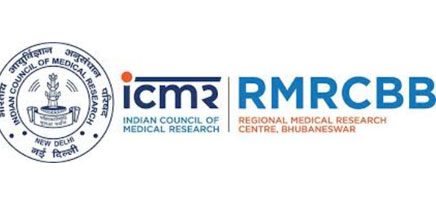
**

**ICMR-Regional Medical Research Centre, Bhubaneswar**

**Odisha Tribal Family Health Survey(OTFHS)**

**Referral Form for Anthropometric measurements and Biomarker Test**

**Sample ID : Name :**

**Age (in years) : Gender:**

1. **Anthropometric measurement and Biomarker Test values**

| **Sl No.** | **Parameter** | **Value** | **Normal range** |
| --- | --- | --- | --- |
| 1 | Height (in cm) |  | NA |
| 2 | Weight (in kg) |  | NA |
| 3 | Waist to Height ratio:  Waist Circumference/Hip Circumference(in cm) |  | Abdominal obesity:  Men: > 0.90; Women: > 00.0.85 |
| 4 | Grip Strength Measurement (in kg) |  | Refer SOP of grip strength measurement for weak, normal and strong response |
| 5 | Blood Pressure:  Systolic/ Diastolic (in mmHg) |  | 120/80 mmHg |
| 6 | Random Blood Sugar (mg/dL) |  | Non-diabetic: ≤200 mg/dL  Diabetes: > 200 mg/dL |
| 7 | Haemoglobin (g/dL) |  | Male: 13.5-18 g/dL  Female: 12-16 g/dL |

1. **SCD/SCT:**

| Haemoglobin A2/C/E | % |
| --- | --- |
| Haemoglobin S | % |
| Haemoglobin F | % |
| Haemoglobin A | % |

**(Signature of Lab Technician)**

**Date:**

**District:**
