# Supplementary material for: Odisha tribal family health survey: methods, tools, and protocols for a comprehensive health assessment survey
Source: Front Public Health. 2023 Jul 10;11:1157241. doi: 10.3389/fpubh.2023.1157241 (PMC10364047; doi:10.3389/fpubh.2023.1157241)
Supplement: Supplementary file 3 [file Table_3.DOCX]

| **Sr No** | **Name of the Tribe** | **Total Population** | **Sample size** | **Households** |
| --- | --- | --- | --- | --- |
| 1 | Chenchu | 13 | 13 | 3 |
| 2 | Mankidi | 31 | 31 | 8 |
| 3 | Ghara | 195 | 195 | 49 |
| 4 | Baiga | 338 | 322 | 80 |
| 5 | Desua Bhumij | 404 | 352 | 88 |
| 6 | Korua | 499 | 386 | 97 |
| 7 | Birhor | 596 | 415 | 104 |
| 8 | Gandia | 1854 | 555 | 139 |
| 9 | Mankirdia | 2,222 | 571 | 143 |
| 10 | Madia | 2,243 | 571 | 143 |
| 11 | Kharwar | 2265 | 572 | 143 |
| 12 | Hill Kharia, Mankirdia and Birhor, Jashipur, Mayurbhanj | 3079 | 593 | 148 |
| 13 | Chuktia Bhunjia | 3086 | 593 | 148 |
| 14 | Rajuar | 3,518 | 601 | 150 |
| 15 | Kol | 4058 | 608 | 152 |
| 16 | Kawar | 5225 | 619 | 155 |
| 17 | Koli, Malhar | 6,423 | 628 | 157 |
| 18 | Kotia | 7,232 | 631 | 158 |
| 19 | Bagata | 8813 | 636 | 159 |
| 20 | Didayi | 8890 | 636 | 159 |
| 21 | Parenga | 9,445 | 638 | 159 |
| 22 | Tharua | 9,451 | 638 | 159 |
| 23 | Kolah Loharas, | 9,558 | 638 | 159 |
| 24 | Dongria Kondh | 9659 | 638 | 159 |
| 25 | Lodha | 9,785 | 639 | 160 |
| 26 | Pentia | 10,003 | 639 | 160 |
| 27 | Binjhia, Binjhoa | 11419 | 642 | 161 |
| 28 | Bondo Poraja | 12231 | 644 | 161 |
| 29 | Bhunjia | 12350 | 644 | 161 |
| 30 | Kulis | 13,689 | 645 | 161 |
| 31 | Jatapu | 14890 | 646 | 162 |
| 32 | Dharua | 18151 | 649 | 162 |
| 33 | Banjara,Banjari | 18257 | 649 | 162 |
| 34 | Mahali | 18,625 | 649 | 162 |
| 35 | Kondadora | 20,802 | 651 | 163 |
| 36 | Dal | 25598 | 654 | 163 |
| 37 | Mundari | 25,655 | 654 | 163 |
| 38 | Kandha Gauda | 26403 | 654 | 163 |
| 39 | Holva | 28149 | 654 | 163 |
| 40 | Omanatya | 28,736 | 654 | 163 |
| 41 | Matya | 30,169 | 654 | 163 |
| 42 | Kutia Kondh | 39761 | 656 | 164 |
| 43 | Lanjia Soura | 40913 | 656 | 164 |
| 44 | Juang | 47095 | 656 | 164 |
| 45 | Kora | 54,408 | 658 | 164 |
| 46 | Paudi Bhuyan | 61303 | 658 | 164 |
| 47 | Mirdhas | 75,940 | 659 | 165 |
| 48 | Ho | 80608 | 659 | 165 |
| 49 | Gadaba | 84689 | 659 | 165 |
| 50 | Sounti | 1,12,803 | 659 | 165 |
| 51 | Bhumia | 125977 | 661 | 165 |
| 52 | Binjhal | 137040 | 661 | 165 |
| 53 | Koya | 1,47,137 | 661 | 165 |
| 54 | Bathudi | 217395 | 661 | 165 |
| 55 | Kharia, Kharian | 222844 | 661 | 165 |
| 56 | Bhumij | 283909 | 661 | 165 |
| 57 | Bhuiya, Bhuyan | 306129 | 661 | 165 |
| 58 | Kisan | 331589 | 661 | 165 |
| 59 | Oraon | 3,58,112 | 661 | 165 |
| 60 | Paroja | 3,74,628 | 661 | 165 |
| 61 | Bhottada,Dhotada | 450771 | 662 | 166 |
| 62 | Shabar, Lodha | 5,16,402 | 662 | 166 |
| 63 | Saora, Savar, | 5,34,751 | 662 | 166 |
| 64 | Munda, | 5,58,691 | 662 | 166 |
| 65 | Kolha | 6,25,009 | 662 | 166 |
| 66 | Gond, Gondo | 888581 | 662 | 166 |
| 67 | Santal | 8,94,764 | 662 | 166 |
| 68 | Khond, Kond, | 1627486 | 662 | 166 |
|  | **TOTAL** | **9622744** | **40921** | **10230** |
