# Supplementary material for: Odisha tribal family health survey: methods, tools, and protocols for a comprehensive health assessment survey
Source: Front Public Health. 2023 Jul 10;11:1157241. doi: 10.3389/fpubh.2023.1157241 (PMC10364047; doi:10.3389/fpubh.2023.1157241)
Supplement: Supplementary file 4 [file Table_4.DOCX]

**ODISHA TRIBAL FAMILY HEALTH SURVEY (OTFHS)**

**INTERVIEWER’S MANUAL**

**ICMR – REGIONAL MEDICAL RESEARCH CENTRE, BHUBANESWAR**

Contents

[Introduction 5](#_Toc107299647)

[Rationale 5](#_Toc107299648)

[Objectives 5](#_Toc107299649)

[Survey Sample 5](#_Toc107299650)

[Survey Organization 6](#_Toc107299651)

[Survey Questionnaires 7](#_Toc107299652)

[Interviewer’s Role 7](#_Toc107299653)

[Training of Interviewers 8](#_Toc107299654)

[Supervision of Interviewers 9](#_Toc107299655)

[Conducting an Interview 9](#_Toc107299656)

[Building Rapport with the Respondent 9](#_Toc107299657)

[1. Make a good first impression. 10](#_Toc107299658)

[2. Obtain respondent(s) consent to be interviewed. 10](#_Toc107299659)

[3. Always have a positive approach. 10](#_Toc107299660)

[4. Assure confidentiality of responses. 10](#_Toc107299661)

[5. Answer any questions from the respondent frankly. 10](#_Toc107299662)

[6. Interview the respondent alone. 11](#_Toc107299663)

[Tips for Conducting the Interview 11](#_Toc107299664)

[1. Be neutral throughout the interview 11](#_Toc107299665)

[2. Never suggest answers to the respondent. 12](#_Toc107299666)

[3. Do not change the wording or sequence of questions. 12](#_Toc107299667)

[4. Handle hesitant respondents tactfully. 12](#_Toc107299668)

[5. Do not form expectations. 13](#_Toc107299669)

[6. Do not hurry the interview. 13](#_Toc107299670)

[7. Use diversionary tactics if necessary 13](#_Toc107299671)

[Language of the Interview 13](#_Toc107299672)

[Field Work Procedure 14](#_Toc107299673)

[A Preparatory Activities 14](#_Toc107299674)

[1. Making callbacks 14](#_Toc107299675)

[2. Keeping answers confidential 14](#_Toc107299676)

[3. Supplies and documents needed for fieldwork 15](#_Toc107299677)

[Contacting Households and Eligible Respondents 15](#_Toc107299678)

[1. Locating sample households 15](#_Toc107299679)

[2. Problems in contacting a household 16](#_Toc107299680)

[3. Identifying respondents 17](#_Toc107299681)

[Checking Completed Questionnaires 18](#_Toc107299682)

[Returning Work Assignments 18](#_Toc107299683)

[Data Quality 18](#_Toc107299684)

[General procedures for completing the questionnaire 18](#_Toc107299685)

[Asking Questions 19](#_Toc107299686)

[1. Parentheses that indicate a choice must be made: 19](#_Toc107299687)

[Recording Responses 19](#_Toc107299688)

[1. Questions with pre-coded responses 19](#_Toc107299689)

[2. Recording responses that are not pre-coded 20](#_Toc107299690)

[3. Recording numbers or dates in boxes 20](#_Toc107299691)

[4. Marking filters 20](#_Toc107299692)

[Correcting Mistakes 21](#_Toc107299693)

[Following Instructions 21](#_Toc107299694)

[1. Skip instructions 21](#_Toc107299695)

[2. Filters 21](#_Toc107299696)

[Flow of OTFHS field data collection 21](#_Toc107299697)

[Cluster questionnaire 22](#_Toc107299698)

[Household Questionnaire 40](#_Toc107299699)

[Completing the Household Questionnaire 40](#_Toc107299700)

[Interview Result codes 41](#_Toc107299701)

[Under 5 Years Old Questionnaire 60](#_Toc107299702)

[5 To 9 Years Old Questionnaire 70](#_Toc107299703)

[10 To 19 Years Old Female Questionnaire 72](#_Toc107299704)

[10 To 19 Years Old Male Questionnaire 94](#_Toc107299705)

[20-59 Years Old Men Questionnaire 95](#_Toc107299706)

[20-59 Years Old Women Questionnaire 96](#_Toc107299707)

[60+ Years Old Questionnaire 98](#_Toc107299708)

[Field Investigation Table 101](#_Toc107299709)

# Introduction

Odisha Tribal family Health Survey (OTFHS) is first state level family health survey focused only on tribal population. The survey will focus on comparing health status between all the 62 Scheduled Tribes and 13 PVTGs which is first of its kind. OTFHS will focus on comprehensive evaluation of the health, demographic as well as socio-economic parameters of the tribal population in Odisha. The OTFHS has been designed as OTFHS, implying all 114 outcomes assessed by OTFHS will be included in OTFHS, with additionally indicators on multimorbidity, care seeking behaviors, social aspects, elderly health etc.

# Rationale

It is well recognized that high quality and reliable data on demographics, socio-economic markers and health are necessary for planning and targeting interventions in specific vulnerable groups. There is limited data available for tribal groups in Odisha, or India for that matter. While there are regular surveys conducted across India to estimate population indicators related to health, such as the National Family Health Survey (OTFHS), these national surveys are not targeted at any group and therefore collect less sample and data from tribal populations in Odisha. In statistical terms, these also tend to be underpowered for such inferences to be made in these specific groups. The proposed Odisha Tribal Family Health Survey (OTFHS) is planned to fill these gaps in evidence. This survey and its findings will provide the policy makers and the general public an accurate and robust estimate of the socio-demographic and health indicators across tribal groups in the state and can help accelerate the tribal health research planning and policy formulation in future

# Objectives

A. To describe and compare the health status of tribal communities in Odisha

B. To estimate the prevalence of key maternal-child health indicators and chronic diseases among the tribal communities in Odisha.

C. To assess the demographic and socio-economic status of the tribal communities in Odisha

# Survey Sample

There are several ways to gather information about people. One way is to contact every person or nearly every person and ask them questions about what you need to know. Talking to everyone is called a complete enumeration, and a national census is a good example of this type of information gathering. This is very costly because it takes a lot of people to talk to everyone. However, in cases such as a national census, it is necessary to have a complete enumeration despite the cost. Another way to collect information is through a sample survey. When it is not necessary to know exact total numbers, a sample survey can collect information about people much more quickly and at a lowcost. The sampling procedure allows us to collect data on a small number of people and draw conclusions that are valid for the whole country. The accuracy of a sample survey depends, among other things, on the size of the sample. The exact number to be interviewed for any survey is determined by statistical methods which we will not try to discuss in this training session. What you should know, however, is that the sample size for this survey reflects the number of interviews that are needed to provide an accurate picture of the population, health and nutrition situation in India. Consequently, it is critical to a survey that fieldworkers try their hardest to complete all assigned interviews to ensure that the correct number of people are included in the survey. The accuracy of a sample survey also depends on another major factor, the absence of bias that would affect the proportions found through the sample. To control or prevent bias from creeping into the results, the selection of people included in the sample must be absolutely random. This means that every person in the total population to be studied has the same opportunity to be selected in the sample. This is why it is so important to make callbacks to reach those people who are not at home, since they may be different from people who are at home. For example, it may be that women who have no children are more likely to be working away from the house, and if we don’t call back to interview them, we may bias the fertility estimates.

For OTFHS, the sample consists of approximately 372 clusters (small geographically defined areas) throughout the 13 districts of Odisha State. A sample of households will then scientifically selected to be included in OTFHS survey from the list in each of the clusters. Each of these households will be visited and information obtained about the household using the Household Questionnaire. All the individuals within these households will be interviewed using an Individual Questionnaire. We expect to interview about 10500 household and 42050 individuals in this survey.

# Survey Organization

OTFHS is being conducted at the request of the SCSTRI Department Odisha which has a primary role in the planning for the survey and in the analysis and dissemination of the survey results. ICMR – Regional Research Centre (RMRC), Bhubaneswar will serve as the implementing agency for OTFHS. RMRC take responsibility for operational matters including planning and conducting fieldwork, processing of collected data and organizing the writing and distribution of reports. Staff from RMRC will be responsible for overseeing the day-to-day technical operations including recruitment and training of field and data processing staff and the supervision of the office and field operations.

During OTFHS fieldwork, you will work in a team consisting of one field supervisor, one field investigator, and two Laboratory Technicians. Each team will have two males and two females. Each team will be accompanied by a driver. Each supervisor will be responsible for a team of interviewers. LTs will be responsible for drawing blood from eligible persons for testing for anemia status, Sickle cell disease, glucose, etc,. They will measure blood pressure for eligible persons. They will also be responsible for the anthropometric measurements of all individuals. In the central office there will be a team of state coordinators responsible for supervising fieldwork teams. These coordinators will ensure regular progress of data collection in the clusters. They will monitor data quality and provide for the regular transfer of completed questionnaires and blood samples to the central office. Information technology specialists will also be assigned to the project.

# Survey Questionnaires

The cluster/Village that have been scientifically selected to be included in the OTFHS sample will be visited and enumerated using a Cluster questionnaire. The cluster information will be collected from the responsible person in the village, it may be Village Pradhan, ASHA, AWW,

The households that have been scientifically selected to be included in the OTFHS sample will be visited and enumerated using a Household Questionnaire. The Household Questionnaire collects information on housing characteristics such as type of water source, sanitation facilities, quality of flooring, and ownership of durable goods. All the individuals in the household will be weighed and measured (height or length) to assess their nutritional status. Among these same populations those who are willing to give the blood sample will do the sample collection. Once the household interview completed, you will use the individual Questionnaires to interview the individual/s you are assigned.

# Interviewer’s Role

The interviewer occupies the central position in OTFHS because he/she collects information from respondents. Therefore, the success of OTFHS depends on the quality of each interviewer’s work.

In general, the responsibilities of an interviewer include the following:

- Locating the structures and households in the sample, and completing the Household Questionnaire
- Identifying all respondents in those households
- Interviewing all respondents in the households using the individual Questionnaire
- Before completing the interview enter the sample ID of all the individuals for anthropometric & blood sample collection
- Checking completed interviews to be sure that all questions were asked
- Returning to households to interview respondents who could not be interviewed during the initial visit.

# Training of Interviewers

Although some people are more adept at interviewing than others, one can become a good interviewer through experience. Your training will consist of a combination of classroom training and practical experience. Before each training session, you should study this manual carefully along with the questionnaire, writing down any questions you have. Ask questions at any time to avoid mistakes during actual interviews. Interviewers can learn a lot from each other by asking questions and talking about situations encountered in practice and actual interview situations. Each of you will receive a package with the following materials.

- Cluster Questionnaire
- Household Questionnaire
- Individual Questionnaires
- Biomarker Questionnaire
- Interviewer’s Manual
- Different Manual of Operating Procedures

Please ensure that you bring these materials each day during the training and to the field during fieldwork. During the training, the questionnaire sections, questions, and instructions will be discussed in detail. You will see and hear demonstration interviews conducted in front of the class as examples of the interviewing process. You will practice reading the questionnaire aloud to another person several times. so that you may become comfortable with reading the questions aloud. You will also be asked to take part in role playing in which you practice by interviewing another trainee. The training will also include field practice interviewing in which you will actually interview household respondents and individuals. The training you receive as an interviewer does not end when the formal training period is completed. Each time a supervisor meets with you to discuss your work, your training is being continued. This is particularly important during the first few days of fieldwork. As you run into situations you did not cover in training, it will be helpful to discuss them with your team. Other interviewers may be running into similar problems, so you can all benefit from each other’s experiences.

# Supervision of Interviewers

Training is a continuous process. Observation and supervision throughout the fieldwork are a part of the training and data collection process. Your team supervisor will play very important roles in continuing your training and in ensuring the quality of OTFHS data. They will:

- Spot-check some of the addresses selected for interviewing to be sure that you interviewed the correct households and the correct respondents
- Review each questionnaire to be sure it is complete and consistent
- Observe some of your interviews to ensure that you are asking the questions in the right manner and recording the answers correctly
- Meet with you on a daily basis to discuss performance and give out future work assignments
- Help you resolve any problems that you might have with finding the assigned households, understanding the questionnaire, or dealing with difficult respondents.

# Conducting an Interview

Successful interviewing is an art and should not be treated as a mechanical process. Each interview is a new source of information, so make it interesting and pleasant. The art of interviewing develops with practice but there are certain basic principles that are followed by every successful interviewer. In this section you will find a number of general guidelines on how to build rapport with a respondent and conduct a successful interview.

## Building Rapport with the Respondent

The supervisor will assign an interviewer to make the first contact with each of the households selected for OTFH. Any capable adult member of the household is a suitable respondent for the household interview. If at least one eligible person is identified in the Household Questionnaire, the interviewer will go on to complete an Individual Questionnaire or pass the interview along to a colleague if they are not the same gender as the respondent. As an interviewer, your first responsibility is to establish a good rapport with a respondent. At the beginning of an interview, you and the respondent are strangers to each other. The respondent’s first impression of you will influence their willingness to cooperate with the survey. Be sure that your manner is friendly as you introduce yourself. Before you start to work in an area, your supervisor will have informed the local leaders, who will in turn inform selected households in the area that you will be coming to interview them. You will also be given a letter and an identification badge that states that you are working with.

### Make a good first impression.

When you arrive at the household, do your best to make the respondent feel at ease. With a few well chosen words, you can put the respondent in the right frame of mind for the interview. Open the interview with a smile and greeting such as “good afternoon” and then proceed with your introduction.

### Obtain respondent(s) consent to be interviewed.

You must obtain a respondent’s informed consent for participation in the survey before you begin an interview. Special statements are included at the beginning of the Household Questionnaire and the Individual Questionnaires. The statements explain the purpose of the survey. They assure a respondent that participation in the survey is completely voluntary and that it is their right to refuse to answer any questions or stop the interview at any point. Be sure to read the informed consent statement exactly as it is written before asking a respondent to participate in a household or individual interview.

### Always have a positive approach.

Never adopt an apologetic manner, and do not use words such as “Are you too busy?” Such questions invite refusal before you start. Rather, tell the respondent, “I would like to ask you a few questions” or “I would like to talk with you for a few moments.”

### Assure confidentiality of responses.

If the respondent is hesitant about responding to the interview or asks what the data will be used for, explain that the information you collect will remain confidential, no individual names will be used for any purpose, and all information will be grouped together to write a report. Also, you should never mention other interviews to the supervisor or field editor in front of a respondent or any other person.

### Answer any questions from the respondent frankly.

Before agreeing to be interviewed, the respondent may ask you some questions about the survey or how he or she was selected to be interviewed. Be direct and pleasant when you answer. The respondent may also be concerned about the length of the interview. If they ask, tell female respondents that the interview usually takes about 30-60 minutes and tell male respondents that the interview takes about 30-40 minutes. If the respondent for the Household Questionnaire is a woman age 50 or older (or a man age 55 or older), you can tell the respondent that the interview usually takes about 25 minutes, since that person will answer only the Household Questionnaire. Indicate your willingness to return at another time if it is inconvenient for the respondent to answer questions then. Respondents may ask questions or want to talk further about the topics you bring up during the interview, e.g., about specific family planning methods. It is important not to interrupt the flow of the interview so tell them that you will be happy to answer their questions or to talk further after the interview.

### Interview the respondent alone.

The presence of a third person during an interview can prevent you from getting frank, honest answers from a respondent. It is, therefore, very important that the individual interview be conducted privately and that all questions be answered by the respondent.

If other people are present, explain to the respondent that some of the questions are private and ask to interview the person in the best place for talking alone. Sometimes asking for privacy will make others more curious, so they will want to listen; you will have to be creative. Establishing privacy from the beginning will allow the respondent to be more attentive to your questions.

If it is impossible to get privacy, you may have to carry out the interview with the other people present. However, in such circumstances, it is important that you remember that:

- If there is more than one eligible respondent in the household, you must not interview one in the presence of the other
- Extra effort should be made to gain privacy if the other person is of the opposite sex, particularly the husband or wife. One way to ensure privacy in this case is to have the husband and wife interviewed simultaneously in two different areas of the household

In all cases where other individuals are present, try to separate yourself and the respondent from the others as much as possible.

## Tips for Conducting the Interview

### Be neutral throughout the interview

Most people are polite and will tend to give answers that they think you want to hear. It is therefore very important that you remain absolutely neutral as you ask the questions. Never, either by the expression on your face or by the tone of your voice, allow the respondent to think that he/she has given the “right” or “wrong” answer to the question. Never appear to approve or disapprove of any of the respondent’s replies.

The questions are all carefully worded to be neutral. They do not suggest that one answer is more likely or preferable to another answer. If you fail to read the complete question, you may destroy that neutrality. For example, the following is a question in OTFHS: “Would you like to have another child or would you prefer not to have any more children?” It is a neutral question. However, if you only ask the first part—“would you like to have another child?”—you are more likely to get a “YES” answer. This is what we call a “leading question.” That is why it is important to read the whole question as it is written.

If the respondent gives an ambiguous answer, try to probe in a neutral way, asking questions such as the following:

“Can you explain a little more?”

“I did not quite hear you; could you please tell me again?”

“There is no hurry. Take a moment to think about it.”

### Never suggest answers to the respondent.

If a respondent’s answer is not relevant to a question, do not prompt him/her by saying something like “I suppose you mean that. . . Is that right?” In many cases, he/she will agree with your interpretation of his/her answer, even when that is not what he/she meant. Rather, you should probe in such a manner that the respondent himself/herself comes up with the relevant answer. You should never read out the list of coded answers to the respondent, even if he/she has trouble answering.

### Do not change the wording or sequence of questions.

The wording of the questions and their sequence in the questionnaire must be maintained. If the respondent has not understood the question, you should repeat the question slowly and clearly. If there is still a problem, you may reword the question, being careful not to alter the meaning of the original question. Provide only the minimum information required to get an appropriate response.

### Handle hesitant respondents tactfully.

There will be situations where the respondent simply says, “I don’t know,” gives an irrelevant answer, acts very bored or detached, or contradicts something they have already said. In these cases, you must try to re-interest them in the conversation. For example, if you sense that they are shy or afraid, try to remove their shyness or fear before asking the next question. Spend a few moments talking about things unrelated to the interview (for example, their town or village, the weather, their daily activities, etc.).

If the respondent is giving irrelevant or elaborate answers, do not stop them abruptly or rudely, but listen to what they have to say. Then try to steer them gently back to the original question. A good atmosphere must be maintained throughout the interview. The best atmosphere for an interview is one in which the respondent sees the interviewer as a friendly, sympathetic, and responsive person who does not intimidate them and to whom they can say anything without feeling shy or embarrassed. As indicated earlier, a major problem in gaining the respondent’s confidence may be one of privacy. This problem can be prevented if you are able to obtain a private area in which to conduct the interview.

If the respondent is reluctant or unwilling to answer a question, explain once again that the same question is being asked of women or men all over India and that the answers will all be merged together. If the respondent is still reluctant, simply write REFUSED next to the question and proceed as if nothing had happened. Remember, the respondent cannot be forced to give an answer.

### Do not form expectations.

You must not form expectations of the ability and knowledge of the respondent. For example, do not assume women and men from rural areas or those who are less educated or illiterate do not know about family planning or various family planning methods.

### Do not hurry the interview.

Ask the questions slowly to ensure the respondent understands what is being asked. After you have asked a question, pause and give the respondent time to think. If the respondent feels hurried or is not allowed to formulate their own opinion, they may respond with “I don’t know” or give an inaccurate answer. If you feel the respondent is answering without thinking just to speed up the interview, say to the respondent, “There is no hurry. Your opinion is very important, so consider your answers carefully.”

### Use diversionary tactics if necessary

If someone walks in when you are asking some sensitive question that requires that complete privacy be maintained, gently change the topic till the person is no longer within hearing distance.

## Language of the Interview

The questionnaires for OTFHS have been translated into Odia language. However, there may be times when you will have to use an interpreter or modify the wording of the questions to fit local dialects and culture. It is very important not to change the meaning of the question when you rephrase it or interpret it into another language. We will be practicing interviews in the local languages during training. Of course, one of the first things you will do when you approach a household to do an interview is to establish the language or languages that are spoken there. We will be arranging the field teams in such a way that you will be working in an area in which your language is spoken, so there should not be many cases in which respondents do not speak your language. In such cases you might be able to find another language that both of you speak and you will be able to conduct the interview in that language. However, in some cases, it will not be possible for you to find a language which both you and the respondent speak. In this case, try to find out if the respondent speaks a language which another member of your team or the team supervisor speaks. If so, tell your supervisor so that he or she can arrange for that person to conduct the interview. If the respondent does not speak a language which any of your team members speak, you will need to rely on a third person to translate for you. Since the interview involves some sensitive topics, it is best if you can find another woman to act as an interpreter if you are conducting the Woman’s interview and a man if you are conducting the man’s interview. You should not use the respondent's spouse as an interpreter under any circumstances. Children are also unsuitable interpreters. Remember; try to avoid using interpreters if at all possible since this can jeopardize the quality of the interview.

# Field Work Procedure

Fieldwork for OTFHS will proceed according to a timetable, and the survey will be successful only if each member of the interviewing team understands and follows correct field procedures. The following sections review these procedures and describe the proper procedures for receiving work assignments and keeping records of selected household

## A Preparatory Activities

### Making callbacks

Because each household has been carefully selected, you must make every effort to conduct interviews with the individuals who are identified as eligible in that household. Sometimes a household member will not be available at the time you first visit. You need to make at least 3 visits on three separate times of the day or days when trying to obtain an individual interview to maximize the possibility of successfully completing the individual interview. At the beginning of each day, you should check to see if you made any appointments for revisiting a household or eligible respondent. If no appointments were made, make your callbacks to a respondent at a different time of day than the earlier visits; for example, if the initial visits were made in the early afternoon, you should try to arrange your schedule so you make a call back in the morning or late afternoon. Scheduling callbacks at different times is important in reducing the rate of non-response (i.e., the number of cases in which you fail to contact a household or complete an individual interview). When using DBMS, at the end of each day transfer the completed household questionnaires and eligible respondents from your DBMS machine to your team supervisor’s DBMS machine.

### Keeping answers confidential

You are responsible for seeing that respondent’s answers are kept confidential. Do not share the results with other interviewers. You should never interview a household in which you know one or more of the members, even if they are only casual acquaintances. If you are assigned to a household in which you know a person even if that person is not eligible for interview, you should notify your supervisor so he can assign that household to another interviewer. You should not attempt to see the completed questionnaires for that household nor discuss the interview results with your colleagues.

### Supplies and documents needed for fieldwork

Before starting fieldwork each morning, verify that you have everything you need for the day’s work. Some necessary supplies include:

- Interviewer’s Manual
- Your personnel identification
- Pens/Pencils
- A bag to carry hard copies of questionnaires, manuals and other survey materials like literacy card, brochures, referral letters, samples of IFA tablets and syrup, etc.

## Contacting Households and Eligible Respondents

### Locating sample households

In recent months, household listing teams visited each of the selected sample PSUs to:

1. prepare up-to-date maps to indicate the location of structures;
2. record address information for each structure or describe their location (for areas lacking street names or numbers on structures);
3. write numbers on structures; and
4. make a list of the names of the heads of households in all of the structures.

A structure is a freestanding building, for a residential or commercial purpose. It may have one or more rooms in which people live; it may be an apartment building, a house, or a thatched hut, for instance. Within a structure, there may be one or more dwelling (or housing) units. A dwelling unit is a room or group of rooms occupied by one or more households. It may be distinguished from the next dwelling unit by a separate entrance. For instance, there would be one dwelling unit in a thatched hut, but there may be 50 dwelling units in an apartment building or five dwelling units in a compound.

Within a dwelling unit, there may be one or more households. By definition, a household consists of a person or group of persons, related or unrelated, who live together in the same dwelling unit, who acknowledge one adult male or female as the head of household, who share the same living arrangements, and are considered as one unit. In some cases one may find a group of people living together in the same house, but each person has separate eating arrangements; they should be counted as separate one-person households. Collective living arrangements such as hostels, army camps, boarding schools, or prisons are not considered as households in OTFHS.

Specific households have been selected to be interviewed, and you should not have any trouble in locating the households assigned to you if you use the structure number and the name of the head of the household to guide you. The structure number is usually written above the door of the house, but sometimes it may be on the wall. Although the supervisor of your team will be with you in the field, it is important that you also know how to locate the structures in the sample by using the sketch map.

### Problems in contacting a household

In some cases you will have problems locating the households that were selected because the people may have moved or the listing teams may have made an error. Here are examples of some problems you may find and how to solve them:

1. The household has moved away and a new one is now living in the same dwelling. In this case, interview the new household.
2. The structure number and the name of the household head do not match with what you find in the field. If you have located the correct dwelling, you should consider the household that is living in the dwelling as the selected household.
3. The household selected does not live in the structure that was listed. If there is a discrepancy between the structure number and the name of the household head, interview whoever is living in the structure assigned to you.
4. The listing shows only one household in the dwelling, but two or more households are living there now. When the listing shows only one household and you find two households, interview both households. The DBMS system will allow you to generate a new household file in this case. Once generated, follow the same procedures. Your supervisor will assign this household a number, which you should enter on the questionnaire. If the listing shows two households, only one of which was selected, and you find three households there now, only interview the one that had been selected and ignore the other two.
5. The head of the household has changed. In some cases, the person listed as the household head may have moved away or died since the listing. Interview the household that is living there.
6. The house is all closed up and the neighbours say the people are on the farm (or away visiting, etc.) and will be back in several days or weeks. The house should be revisited at least two more times to make sure that the household members have not returned.
7. The house is all closed up and the neighbours say that no one lives there; the household has moved away permanently..
8. A household is supposed to live in a structure that when visited is found to be a shop and no one lives there.
9. A selected structure is not found in the cluster, and residents tell you it was destroyed in a recent fire.
10. No one is home and neighbours tell you the family has gone to the market. Return to the household at a time when the family will be back. Discuss with your supervisor any problems you have in locating the households that you are assigned to interview. Remember that the usefulness of the OTFHS sample in representing the tribals of entire state depends on the interviewers locating and visiting all the households they are assigned.

### Identifying respondents

You must identify in the household before starting the interview. If you cannot finish the interview for whatever reason, partially save the case and write the reason in DBMS. Take care to note any information that may be useful when you contact the person later.

You may experience the following types of problems in obtaining an interview with a respondent:

1. Eligible respondent not available. If the eligible respondent is not at home when you visit, enter ‘NOT AT HOME as the result for the visit and ask a neighbour or family member when the respondent will return. You should contact the household at least three times, trying to make each visit at a different time of day. Under no circumstances is it acceptable to conduct all three visits on the same day and then stop attempting to contact the respondent.
2. Respondent refuses to be interviewed. The respondent’s availability and willingness to be interviewed will depend in large part on the initial impression you make when you meet them. Introduce yourself and explain the purpose of the visit. Read the introduction for the Individual Questionnaire. You may emphasize the confidentiality of the information the respondent provides, and/or the short duration of the interview. If the respondent is unwilling to be interviewed, it may be that the present time is inconvenient. Ask whether another time would be more convenient and make an appointment. If the individual still refuses to be interviewed, enter ‘REFUSED as the result for the visit and report it to your supervisor.
3. Interview not completed. A respondent may be called away during the interview or they may not want to answer all the questions at the time you visit them. If an interview is incomplete for any reason, you should arrange an appointment to see the respondent again as soon as possible to obtain the missing information. Be sure that you record that the interview is incomplete by entering ‘PARTLY COMPLETED’ and indicate the time you agreed on for a revisit; you should also report the problem to your supervisor.
4. Respondent incapacitated. There may be cases in which you cannot interview a person because they are too sick, because they are mentally unable to understand your questions, or because they are deaf, etc. In these cases, record Code ‘INCAPACITATED’.

## Checking Completed Questionnaires

It is the responsibility of the interviewer to review each questionnaire when the interview is finished. This review should be done before you leave the household so that you can be sure every appropriate question was asked, that all answers are clear and reasonable, and that your handwriting is legible. Also check that you have followed the skip instructions correctly. You may be able to make minor corrections yourself, but in many cases you will need to talk further with the respondent. Simply explain to the respondent that you made an error and ask the question(s) again. Do not recopy questionnaires. As long as the answers are clear and readable, it is not necessary that the questionnaire itself be neat. Every time you transcribe the answers to a new questionnaire, you increase the chance of an error. For this reason you are not allowed to use work sheets to collect information. Record ALL information on the questionnaires provided. Any calculations you make should be written in the margins or on the back of the questionnaires. Anything out of the ordinary should be explained either in the margins near the relevant question or in the comments section at the end. These comments are very helpful to the supervisor and field editor in checking questionnaires. Comments are also read in the office and used to resolve problems encountered during data entry.

## Returning Work Assignments

At the end of fieldwork each day, check that you have filled out the cover sheet of a Household Questionnaire for each household assigned to you, whether or not you managed to complete an interview. Check also that you have completed the cover sheet of the Individual Questionnaire for each respondent identified, whether you were able to interview them or not.

## Data Quality

It is the responsibility of the field supervisor to review both the Household Questionnaires and the Individual Questionnaires from a sample PSU while the interviewing team is still in the cluster. It is especially important for the field supervisor to conduct thorough edits of questionnaires at the initial stages of fieldwork. The field supervisor will discuss with each interviewer the errors found in the collection of data. It may sometimes be necessary to send an interviewer back to a respondent in order to correct some errors.

# General procedures for completing the questionnaire

To collect the information needed by OTFHS, you must understand how to ask each question, what information the question is attempting to collect, and how to handle problems that might arise during the interview. You must also know how to correctly record the answers the respondent gives and how to follow special instructions in the questionnaire. This part of the training manual is designed to familiarize you with OTFHS questionnaire.

## Asking Questions

It is very important that you ask each question exactly as it is in the questionnaire. When you are asking a question, speak slowly and clearly so that the respondent will have no difficulty hearing or understanding the question. At times you may need to repeat the question to be sure the respondent understands it. In those cases, do not change the wording of the question but repeat it exactly as it is written.

If, after you have repeated a question, the respondent still does not understand it, you may have to restate the question. Be very careful when you change the wording, however, that you do not alter the meaning of the original question. In some cases, you may have to ask additional questions to obtain a complete answer from a respondent (we call this ‘probing’). If you do this, you must be careful that your probes are “neutral” and that they do not suggest an answer to the respondent. Probing requires both tact and skill, and it will be one of the most challenging aspects of your work as a OTFHS interviewer.

You will notice that some questions contain one or more words in parentheses. As shown below, the presence of parentheses indicates that a sentence needs to be adapted to fit the respondent’s specific situation.

### Parentheses that indicate a choice must be made:

The question above is asked to female respondents. How you phrase the question – that is, which word you use in parentheses – will depend on whether the respondent has had children or not. If the woman has children, you would ask “Would you like to have another child, or would you prefer not to have any more children?” Would you like to have a child, or would you prefer not to have any children?” Most of these questions will appear in DBMS with the choice selected for you but some questions will require a choice.

## Recording Responses

There are three types of questions in the OTFHS questionnaire:

1. Questions that have pre-coded responses;
2. Questions that do not have pre-coded responses, i.e., that are “open-ended”; and
3. filters.

### Questions with pre-coded responses

For some questions, we can predict the types of answers a respondent will give. The responses to these questions are listed in the questionnaire. To record a respondent’s ans in DBMS, select the option on the screen.

In some cases, precoded responses will include ‘OTHER’. The OTHER code should be circled only when the respondent’s answer is different from any of the precoded responses listed for the question. Before using the OTHER code, you should make sure the answer does not fit in any of the specified categories. When you circle the OTHER code for a particular question you must always write the respondent’s answer in the space provided. If you need more room, use the margins or the comments section at the end. If you use the comments section, write, “see note in comments section” next to that question. When using DBMS, you will be prompted to type other answer when this code is selected.

### Recording responses that are not pre-coded

The answers to some questions are not precoded but require that you write the respondent’s answer in the space provided. In DBMS, you will type the name.

### Recording numbers or dates in boxes

In some questions, you will record a number or date in the space provided. There are two ways this is done:

Boxes preceded by codes. Whenever the boxes are preceded by codes, you will fill in the boxes in one row only. When using DBMS, you will first enter the numbers and then choose the units.

Boxes without preceding codes. Whenever boxes are present without codes in front of them, you must enter information in all of the boxes. When using DBMS, you will be prompted to enter numbers for each category, i.e. months then years. Example: For a child born in February 2015, you must record both the month and year.

When a response has fewer digits than the number of boxes provided, you should fill in leading zeroes. For example, a response of ‘9’ is recorded ‘09’ in two boxes, or if three boxes had been provided, you would record ‘009’. Recording the answer exactly as given. There are questions where you must write down the response in the respondent’s own words. Try to record those answers exactly as they are given; if you need to shorten a lengthy description, be careful to keep the meaning accurate, and if necessary, write a note on the bottom or side of the page to explain. In DBMS, you will select from a list of pre-defined occupation categories.

### Marking filters

Filters require you to look back to the answer to a previous question and then mark an ‘X’ in the appropriate box. When using DBMS, these filters will be automatically applied for you.

## Correcting Mistakes

It is very important that you record all answers neatly. For precoded responses, be sure that you choosen the correct response carefully. For open ended responses, the reply should be written legibly so that it can be easily read. If you made a mistake in entering a respondent’s answer or she changes her reply, be sure that you change the incorrect response and enter the right answer.

## Following Instructions

Throughout the OTFHS questionnaire, instructions for the interviewer are printed in all.

### Skip instructions

It is very important not to ask a respondent any questions that are not relevant to his or her situation. For example, a woman who is not pregnant should not be asked for how many months she has been pregnant. In cases where a particular response makes subsequent questions irrelevant, an disable (skip) pattern will be automatically applied in DBMS.

### Filters

To ensure the proper flow of the questionnaire, you will sometimes be directed to check a respondent’s answer to an earlier question, indicate what the response was by marking a box, and then follow various skip instructions. Questions of this type are called “filters”; they are used to prevent a respondent from being asked irrelevant, and perhaps embarrassing or upsetting, questions. For filter questions, it is important that you follow the instructions that ask you to check back to an earlier question. Do not rely on your memory. Remember that you do not need to ask the respondent the same question a second time. Check back and mark an ‘X’ in the appropriate box in the filter then follow the skip instructions. When using DBMS, these skips will be automatically applied.

# Flow of OTFHS field data collection

The OTFHS field data collection will follow different stages. The steps detailed below.

1. The field data collection will starts with interview of cluster level questionnaire by the field supervisor (RA)
2. Once the cluster interview completed, it will move to the selected household and starts with the household interview
3. When the first household interview is going on it is the responsibilities of Laboratory Technicians (LT) to set up the place for biomarker measurements collection
4. Once the household interview completed, the next stage is to start with the individual interviews. The number of interviews will be conducted according to the members in the household. Field Investigator and/ Laboratory Technician under the supervision of fiels supervisor will conduct the interview
5. Enter the Sample ID just before concluding the one-on-one interview, then finish it. Next, invite the person to proceed to the location where the biomarker collection will be taking place while writing down their name, age, gender, and Sample ID that they provided during the individual interview on a paper slip.
6. Then the individual will move for biomarker measurement
7. The responsible LT/s will conduct the biomarker tests and will write the results in the prescribed paper format given to the LT
8. Once all the biomarker tests completed LT will enter the reported results in the ‘Filed Investigation Table’ module in the DBMS.

# Cluster questionnaire

Cluster questionnaire collects the information regarding the availability and accessibility of various facilities in the village, especially on educational and health facilities. The enumeration will be done from the responsible person of the village, it may be Village Pradhan, ASHA, AWW, etc., Once the cluster questionnaire completed, the interviewers will move to the respective households for the interviews.

**Cluster belongs to**

Q.1: The cluster question starts asking about the cluster belongs to; the responses may be, village, hamlet Notified Area Council (NAC) or Municipal Corporation.

| Response Categories | Definition |
| --- | --- |
| Village | According to Census Commission, a village is identified by its name having definite boundaries. The Census of India has defined a village as, “The basic unit for rural areas is the revenue village which has definite surveyed boundaries. The revenue village may comprise several hamlets but the entire village has been treated as one unit for presentation of census data. In un-surveyed areas, like village within forest areas, each habitation area with locally recognized boundaries within each forest range officer’s beat, was treated as one unit.” |
| Hamlet | A hamlet is a small [settlement](https://simple.wikipedia.org/wiki/Human_settlement), smaller than a [village](https://simple.wikipedia.org/wiki/Village). Usually, all settlers in a hamlet are centered around a single economic activity. A group of houses in a revenue village. |
| Notified Area Council (NAC) | Notified Area Council (NAC) in [India](https://en.wikipedia.org/wiki/India) is a settlement in transition from rural to urban and therefore a form of an urban political unit comparable to a [municipality](https://en.wikipedia.org/wiki/Nagar_Palika). An urban centre with more than 12,000 and less than 40,000 inhabitants is classified as a Nagar Panchayat. |
| Municipal Corporation | Municipal incorporation occurs when such [municipalities](https://en.wikipedia.org/wiki/Municipality) become self-governing entities under the laws of the state or province in which they are located. Often, this event is marked by the award or declaration of a [municipal charter](https://en.wikipedia.org/wiki/Municipal_charter). A city charter or town charter or municipal charter is a [legal document](https://en.wikipedia.org/wiki/Legal_document) establishing a municipality, such as a [city](https://en.wikipedia.org/wiki/City) or [town](https://en.wikipedia.org/wiki/Town) |

**Number of households in the cluster**

Q.2: Here the question asks about total number of households in the cluster. A household is person or group of persons who commonly live together and would take meals from a common kitchen unless the exigencies of work prevented any of them from doing having a mix of both. In some cases, one may find a group of people living together in same structure, but each person has separate eating arrangement; they should be counted as separated one-person households. Collective living arrangement such as boarding houses, mess hotels, residential hotels, rescue homes, jails, army camps, boarding schools or ashram will not be considered as households, and are not included in the survey.

**Number of ST households in the cluster**

Q.3: On continuation of Q.2, get the number if ST households in the village.

**Source of Drinking Water**

Q.4: The purpose of this question is to assess the cleanliness of the drinking water by asking about the cluster’s main source of water. If drinking water is obtained from several sources, probe to determine the source from which the cluster obtains the majority of its drinking water. If the source varies by season, record the main source used at the time of interview.

| **Definitions of Water Source** | |
| --- | --- |
| ***Response Categories*** | ***Description*** |
| Surface water | Water located above ground and includes rivers, dams, lakes, ponds, streams, canals, and irrigation channels.  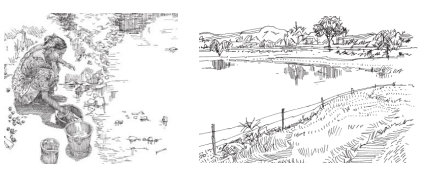 |
| Piped into dwelling | Pipe connected with in-house plumbing to one or more taps, e.g. in the kitchen and bathroom. Sometimes called a house connection.  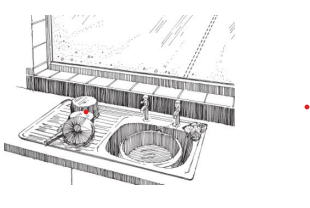 |
| Piped to yard/plot | Pipe connected to a tap outside the house in the yard or plot. Sometimes called a yard connection.  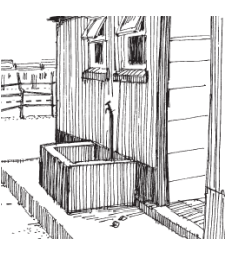 |
| Public tap or standpipe | Public water point from which community members may collect water. A standpipe may also be known as a public fountain or public tap. Public standpipes can have one or more taps and are typically made of brickwork, masonry or concrete.  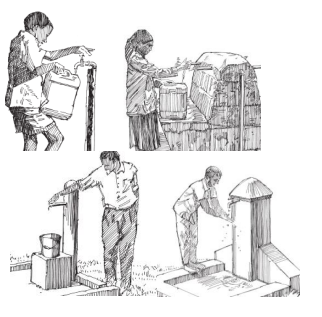 |
| Tube well or borehole | A deep hole that has been driven, bored or drilled with the purpose of reaching ground water supplies. Water is delivered from a tubewell or borehole through a pump which may be human, animal, wind, electric, diesel or solar-powered.  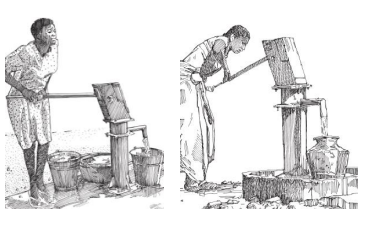 |
| Protected dug well | A dug well that is (1) protected from runoff water through a well lining or casing that is raised above ground level and a platform that diverts spilled water away from the well and (2) covered so that bird droppings and animals cannot fall down the hole. Both conditions must be observed for a dug well to be considered as protected.  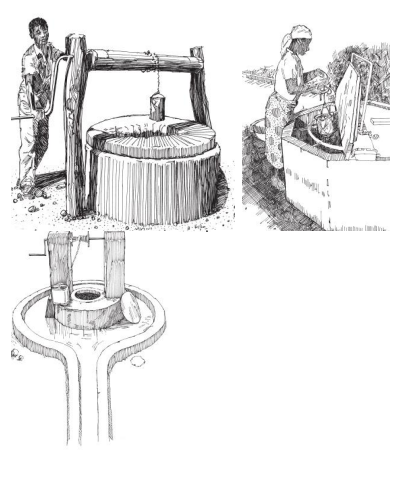 |
| Unprotected dug well | A dug well which is (1) unprotected from runoff water; (2) unprotected from bird droppings and animals; or (3) both.  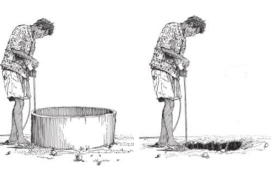 |
| Protected spring | A spring protected from runoff, bird droppings, and animals by a “spring box” which is typically constructed of brick, masonry, or concrete and is built around the spring so that water flows directly out of the box into a pipe without being exposed to outside pollution.  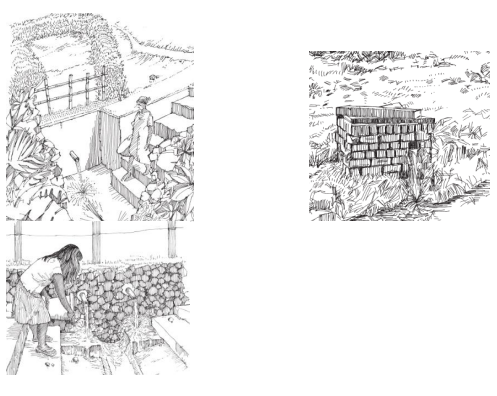 |
| Unprotected spring | A spring that is subject to runoff and/or bird droppings or animals. Unprotected springs typically do not have a “spring box”. |
| Rainwater | Rain that is collected or harvested from surfaces by roof or ground catchment and stored in a container, tank or cistern.  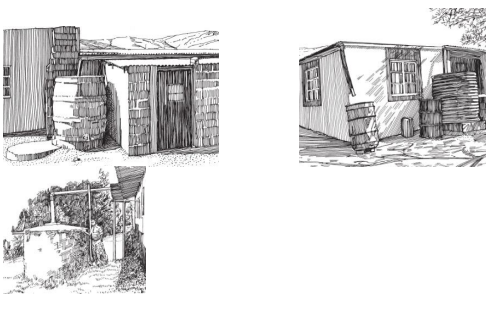 |
| Tanker truck | Water is obtained from a provider who uses a truck to transport water into the community. Typically, the provider sells the water to households.  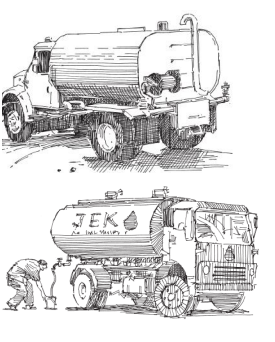 |
| Cart with small tank | Water is obtained from a provider who transports water into a community using a cart and then sells the water. The means for pulling the cart may be motorized or non-motorized (e.g., a bullock).  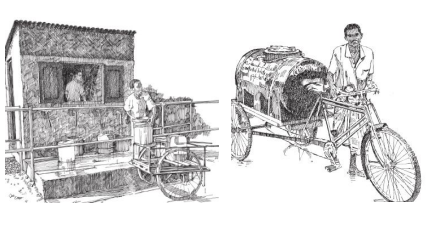 |
| Bottled water | Water that is bottled and sold to the household in bottles. |
| Community RO Plant | Reverse Osmosis (RO) is a process for creating safe drinking water by forcing water under high pressure through a filter. |

**Drainage Facility**

Q.5 – Q. 6: Here Q.6, asks to get the information about whether the drainage facility is available in the village or not. If the answer is ‘yes’, ask Q.7 to get the idea about type of drainage facility used by the village.

**Electricity**

Q.7, asks to get the information about whether the electricity is available in the village or not.

**Source if Irrigation**

Q.8: Village which is irrigated by one or more sources of irrigation such as a, pond/tank, stream/river, canal, well, or tube well is to be recorded here. Land which depends only on rain for irrigation is not to be included as irrigated land.

**Distance from the major places**

Q.9 – Q.12: These questions asking for the distance from the village to nearest town, district head quarter, railway station and bus station. This will help us to find out the average distance from village to the important places.

**Village connected by an all-weather road to the health facility**

Q. 13, is very important to gather the information on the village is connected by an all-weather road to the different health facilities, such as, SC, PHC, CHC, districts hospitals etc.

**Educational facilities available in the village**

Q. 14, asks to enumerate the details about different educational facilities available in the village. Here we are asking all type of formal & non-formal educational facilities available in the village. If the educational facilities not available in the village ask, what is the distance to nearest available educational facilities.

| **Educational Facilities** | **Description** |
| --- | --- |
| Primary School | Schools which are having Classes from 1 – V |
| Secondary School | Schools which are providing 10^th^ class education |
| Higher Secondary School | Schools which are providing 12^th^ class education |
| Collages | These are institutions which can run degree programmes but are not empowered to provide degree on their own and are necessarily have to be attached with some University/University level institution for the purpose of awarding degree. For the purpose of the survey these institutions have been classified as under: |
| Non-formal Education | Non-formal learning includes various structured [learning](https://en.wikipedia.org/wiki/Learning) situations which do not either have the level of [curriculum](https://en.wikipedia.org/wiki/Curriculum), [syllabus](https://en.wikipedia.org/wiki/Syllabus), [accreditation](https://en.wikipedia.org/wiki/Educational_accreditation) and [certification](https://en.wikipedia.org/wiki/Certification) associated with '[formal learning](https://en.wikipedia.org/wiki/Formal_learning)', but have more structure than that associated with '[informal learning](https://en.wikipedia.org/wiki/Informal_learning)', which typically take place naturally and spontaneously as part of other activities.  Examples of non-formal learning include swimming sessions for toddlers, community-based sports programs, and programs developed by organisations such as the [Boy Scouts](https://en.wikipedia.org/wiki/World_Organization_of_the_Scout_Movement), the [Girl Guides](https://en.wikipedia.org/wiki/Girl_Guides), community or non-credit [adult education](https://en.wikipedia.org/wiki/Adult_education) courses, sports or fitness programs, professional conference style [seminars](https://en.wikipedia.org/wiki/Seminar), and continuing professional development. |
| Religious School/Madrasa/Mission School | A religious school is a [school](https://en.wikipedia.org/wiki/School) that either has a [religious](https://en.wikipedia.org/wiki/Religion) component in its operations or its [curriculum](https://en.wikipedia.org/wiki/Curriculum), or exists primarily for the purpose of teaching aspects of a particular religion.  A school can either be of two types, though the same word is used for both in some areas:   1. Religious teaching - Institutions solely or largely for teaching a particular religion, often outside regular school (Examples: Madrasa, Sunday school) 2. General education - Institutions providing general education but run by a religious group, or in some way giving extra weight to a particular religion (Catholic School, Christian School, etc.,)   A mission school or missionary school is a religious school originally developed and run by [Christian missionaries](https://en.wikipedia.org/wiki/Mission_(Christian)). |
| Tribal Residence School / Eklavya Model Residential School (EMRS) | Eklavya Model Residential School (EMRS) is a [Government of India](https://en.wikipedia.org/wiki/Government_of_India) scheme for model [residential school](https://en.wikipedia.org/wiki/Boarding_school) for [Indian tribals](https://en.wikipedia.org/wiki/Indian_tribals) (ST, [Scheduled Tribes](https://en.wikipedia.org/wiki/Scheduled_Tribes)). |

**Health facility available in the village**

Q. 15, ask to get the knowledge about availability of different health facilities in the village. Details about health facilities listed below.

| **Educational Facilities** | **Description** |
| --- | --- |
| Integrated Child Development Services (ICDS)- Anganwadi Centres | Anganwadi is a type of rural child care centre in [India](https://en.wikipedia.org/wiki/India). They were started by the Indian government in 1975 as part of the [Integrated Child Development Services](https://en.wikipedia.org/wiki/Integrated_Child_Development_Services)  (ICDS) program to combat child hunger and [malnutrition](https://en.wikipedia.org/wiki/Malnutrition). A typical Anganwadi center provides basic health care in a village. It is a part of the Indian public health care system. Basic health care activities include [contraceptive](https://en.wikipedia.org/wiki/Contraceptive) counselling and supply, [nutrition](https://en.wikipedia.org/wiki/Nutrition) education and supplementation, as well as pre-school activities. |
| Sub-Centers (SCs) | Sub-Centers (SCs) are the most peripheral health units catering to the health care needs of the rural population. It is the most peripheral contact point between the Primary Health Care system and the community. SC caters the population of 5000 in plain area and 3000 in hilly area. It is manned by one multipurpose worker (male) and one multi-purpose worker (female) /ANM. |
| Primary Health Centre (PHC) | The concept of Primary Health Centre (PHC) is not new to India. Bhore Committee in 1946 gave the concept of a PHC as a basic health unit to provide to the people as close as possible, an integrated curative and preventive health care to the rural population with emphasis on preventive and promotive aspects of health care. It acts as a referral unit for 6 sub-centres and refers out cases to Community Health Centres (CHC- 30 bedded hospital) and higher order public hospitals and sub-district hospitals. It has 4-6 indoor beds for patients covering 20,000 to 30,000 populations. |
| Block PHC | located at block HQ and covering about 100,000 population and with varying number of indoor beds) and additional PHCs/New PHCs covering a population of 20,000-30,000 etc. Regarding the block level PHCs it is expected that they are ultimately going to be upgraded as Community Health Centres with 30 beds for providing specialized services. |
| Community Health Centre (CHC)/ Rural Health Centre | The Community Health Centre (CHC) functions as the secondary level of health care designed to provide first referral curative as well as specialized health care to the rural population. it is catering to approximately 80,000 population in tribal / hilly areas and 1,20,000 population in plain areas so 4 or 5 PHCs are attached to each CHC . It is a 30-bedded hospital providing specialized care in Medicine, Obstetrics and Gynecology, Surgery and Pediatrics. |
| District/ Govt. Hospital | Hospital serving at secondary referral level responsible for a district of a defined geographical area containing defined population i.e. district termed as District Hospital. District Hospital can be graded as 100 bedded, 200 bedded, 300 bedded and 500 bedded. There may be District Hospitals of capacity 700 beds which may be attached to the teaching facility such as Medical Collage. |
| Govt. Dispensary | Dispensary is an outpatient department of a hospital. Dispensary is an office in a hospital or other institutions from which medical supplies and medicines are dispensed or a place for dispensation of free or low cost medical treatment. Dispensaries run by any Ministry/Government Department such as the Railways/Defence/Atomic Energy/etc. |
| Private Clinic | A medical facility smaller than a hospital called a clinic, and is often run by a government agency for health services or a private partnership of physicians (in nations where private practice is allowed). Clinics generally provide only outpatient services. |
| Private Hospital | Any other hospital/ nursing home/ day care centre with facilities for in-patient treatment called a private hospital. |
| AYUSH Health Facility | Each letter of the word AYUSH represents a specific system of medicine: A for Ayurveda, Y for Yoga and Naturoathy, U for Unani, S for Siddha, and H for Homeopathy. Thus AYUSH encompasses the Indian System of Medicines, Yoga and Naturopathy, and Homeopathy. Treatment by any of these systems were therefore qualify as AYUSH treatment, and medicines used by any of these systems were called AYUSH medicines. |
| Nutrition Rehabilitation Center | Nutrition Rehabilitation Center (NRC) is a health facility where children with Severe Acute Malnutrition (SAM) are admitted and managed. A steady linkage with ICDS identifies and refers severely malnourished children in the community using MUAC tape. Children are admitted in NRC as per the defined admission criteria adopted in line with IAP 2006 and new WHO 2009 recommendations and provided with medical and nutritional therapeutic care. |
| Health & Wellness Center | MoHFW envisages upgradation of all 1.5 lakh SCs to Health and Wellness Centres (HWCs) for provision of comprehensive primary healthcare by December 2022. |

**Health providers available in the Village**

Q. 16: Ask about the availability of medical professionals in the village in response to question 16. Health professionals may reside in the village or go there from elsewhere. The details about different health care providers are listed below.

| Health care providers | Description |
| --- | --- |
| Anganwadi Worker (AWW) | These are the staff of the Anganwadi centre in the village. There is one Anganwadi centre for every 1000 population. These centres provide supplementary nutrition, non-formal pre-school education, nutrition and health education, immunization, health check-up and referral services. They are provided with a drug kit and may give tablets for about 1 to 3 children in a day. |
| Accredited Social Health Activist (ASHA) | ASHA is a female health activist in the community who creates awareness on health and its social determinants, mobilize the community towards local health planning and increased utilization of existing health services. ASHA has flexible work schedule for 2-3 hrs/day, 4days/week except during mobilization events, training programme. She works at home and in the community. The norm is “one ASHA for 1000 population”. |
| Trained Birth Attendant (TBA) | A Trained Birth Attendant (TBA) is defined as "an accredited health professional - such as midwife, doctor or nurse - who has been educated and trained to achieve proficiency in the skills needed to manage normal (uncomplicated) pregnancies, childbirth and immediate postnatal period and in the identification, management and referral of complications in women and newborns." |
| Auxiliary Nurse Midwife (ANM) | A female health worker and may have a male health worker. Their main task (as perceived) is to provide immunization to children and antenatal care. Some sub-centres also conduct normal delivery but they have no beds and the sub- centre is not considered as an institution with in-patients. They perform some outpatient care largely in the form of treatment for basic illnesses. Any treatment taken from ANM during her visit to the village can be considered as treatment taken at sub-centre. |
| Trained Lady Doctor & Trained Private Doctor | In this survey the here ‘allopathy’ is used to refer to the broad category of medical practice that is sometimes called Western medicine, biomedicine, evidence-based medicine, or modern medicine. According to MedTerms Dictionary, allopathic medicine is defined as ‘the system of medical practice which treats disease by the use of remedies which produce effects different from those produced by the disease under treatment’. |
| Unani, Ayurveda & Sidha Doctor (Indian System of Medicines) | The doctor practices Ayurveda, Siddha, Unani and Sowa- Rig-Pa medicines are called Indian System of Medicine (ISM). These medicines are also called Desi Dawaiyan in India. Herbal medicines are also included in this category of medicines. The practitioners of these systems may be called Vaidji, Vaidya, Siddha Vaidya, Hakim, etc. (Sometimes people also say Jadi-Booti wale Vaidji, Hakimji, etc.) This category also includes Home-made medicines and Gharelu Nuskhe, Herbal Medicines (Jadi-Bootiyan or Desi Dawa), and the medicines given by local Vaidya/Hakim. e.g. Neem leaves for skin diseases, Tulsi leaves for common cold, Haldi (turmeric) for injuries and fracture, Adarak (ginger) for cough, cold, throat problem etc., Lahasun (Garlic) for gathiya/ joint pain, Kali Mirch (pepper) and honey for dry and productive cough, Ashwagandha, Chyawanprash as tonic /Rasayana for energy, Gulab Jal for eye diseases and face wash, Saunf for indigestion, Ajowain and Hing for stomach pain, Methi seeds, Ajawain, Pudina (mint), Jeera, Sunthi (dry ginger), Laung (clove), Triphala powder for problems like indigestion, loss of appetite, constipation, Laung (clove) oil for toothache, Bilva (Bel) powder for diarrhoea, etc. |
| Homeopathic Doctor | Homeopathy is a system of medicine that uses highly diluted doses from the plant, mineral and animal kingdoms to stimulate natural defenses in the body. Oral Homoeopathy medicine is available in many forms, including the traditional homoeopathic pellets (balls), liquid dilution, tablets (lactose-based) and mother tincture. |
| Registered Medical Practitioner (RMP) | An unqualified healthcare practitioner without any formal registration practicing allopathic medicine can be called an RMP. Widely identified as a rural medical practitioner, the RMP in India enjoys much standing among rural residents and people living in urban slums. |
| Traditional Healer | The traditional medical practitioner or traditional healer can be defined as “someone who is recognized by the community in which he lives as competent to provide health care by using vegetable, animal and mineral substances and certain other methods based on the social, cultural and religious backgrounds as well as the prevailing knowledge, attitudes and beliefs regarding physical, mental and social well-being and the causation of disease and disability in the community”. Traditional healers used different medicinal formulas from various natural substances (animal, mineral and vegetable). They have extensive knowledge on the use of plants and herbs for medicinal and nutritional purposes |
| Faith healer | A method of healing by faith rather than through conventional medicine. Prayers to gods and deities are typically used in the practise of faith healing. Every religion has its own faith-based medical procedures. |
| Untrained Dai | Majority of deliveries in the rural areas are conducted by Dais. |
| Lady Health Visitor (LHV) | Lady health visitors (LHVs) are female health care providers trained to provide nursing care, maternal and child health facilities to individuals in urban and rural communities |
| Bengali Doctor |  |

**Mobile health clinic**

Q. 17 – Q. 18: The Mobile Health Unit (MHU) is envisaged to deliver health care services to people living in the inaccessible and remote areas. In view of the large geographically difficult areas in Orissa, the MHU forms an integral part of the overall health care delivery strategy adopted by the Government of Orissa. Unlike static health facility, this is a unique model aiming to improve the access to health care of population groups residing in the difficult to reach areas.

In order to learn more about mobile health clinics, question 17 must be asked. If the response is "yes," ask Q. 18 to determine how many mobile health visits took place in the village during the previous three months.

Q. 19 ask to get the information on number of health or family welfare camps in the last six months in and around the village.

**Other Facilities available in the village**

Q. 20: Ask question 20 to get the information on other necessary facilities are available in the village or not. Other facilities include, Post / Telegraph office, Pharmacy/medical shop, Bank, Self Help Groups, Haat/Market, etc,.

**Cleaning & Fogging**

Q. 21, ask the number of times cleaning, fogging drive was undertaken in the village during last six months.

**Rogi Kalyan Samiti (RKS)**

Q. 22, This question gathers the information on whether the Rogi Kalyan Samiti been constituted in the PHC of the cluster area. Rogi Kalyan Samiti (Patient Welfare Committee) / Hospital Management Society (HMS)is a simple yet effective management structure. This committee, which would be a registered society, acts as a group of trustees for the hospitals to manage the affairs of the hospital. It consists of members from local Panchayati Raj Institutions (PRIs), NGOs, local elected representatives and officials from Government sector who are responsible for proper functioning and management of the hospital / Community Health Centre / FRUs. RKS / HMS is free to prescribe, generate and use the funds with it as per its best judgement for smooth functioning and maintaining the quality of services.

**Programmes implemented in the village**

Question 23 requests information about the many programmes that have been put into place in the village. The programmes' specifics are provided below.

| Janani Shishu Suraksha Karyakaram (JSSK) | JSSK scheme is to benefit pregnant women who access Government health facilities for their delivery so as to improve Maternal and Child healthcare and to alleviate out of Pocket expenditure on health care. Under JSSK programme, there is an entitlement of free drugs, free referral transport, diagnostics including diet during the duration of stay for every pregnant women and sick neonate up to one year of age. |
| --- | --- |
| Mid-day Meal Programme (MMP) | The Indian Mid-day Meal (MDM) programme provides nutritional support in the form of school-served lunch to kids in grades 1 through 8 (ages 6 to 14 years) who attend primarily government and government-aided schools. |
| Integrated Child Development Scheme (ICDS) | The Integrated Child Development Services (ICDS) Scheme is one of the flagship programmes of the Government of India and represents one of the world’s largest and unique programmes for early childhood care and development. It is the foremost symbol of country’s commitment to its children and nursing mothers, as a response to the challenge of providing pre-school non-formal education on one hand and breaking the vicious cycle of malnutrition, morbidity, reduced learning capacity and mortality on the other. The beneficiaries under the Scheme are children in the age group of 0-6 years, pregnant women and lactating mothers. he ICDS Scheme offers a package of six services, viz., Supplementary Nutrition, Pre-school non-formal education, Nutrition & health education, Immunization, Health check-up and  Referral services |
| National Rural Employment Guarantee Act (NREGA) | National Rural Employment Guarantee Act 2005 or NREGA ([No 42](https://www.indiacode.nic.in/bitstream/123456789/2014/1/A2005-42.pdf)), later renamed as the Mahatma Gandhi National Rural Employment Guarantee Act or MGNREGA in 2009, is an [Indian labour law](https://en.wikipedia.org/wiki/Indian_labour_law) and [social security](https://en.wikipedia.org/wiki/Social_security) measure that aims to guarantee the '[right to work](https://en.wikipedia.org/wiki/Right_to_work)'. It aims to enhance livelihood security in rural areas by providing at least 100 days of wage employment in a financial year to at least one member of every household whose adult members volunteer to do unskilled manual work. Women are guaranteed one third of the jobs made available under the MGNREGA. Another aim of MGNREGA is to create durable assets (such as roads, canals, ponds and wells). Employment is to be provided within 5 km of an applicant's residence, and minimum wages are to be paid. If work is not provided within 15 days of applying, applicants are entitled to an unemployment allowance. That is, if the government fails to provide employment, it has to provide certain unemployment allowances to those people. |
| Village Health, Sanitation and Nutrition Programme (VHN) | The committee has been formed to take collective actions on issues related to health and its social determinants at the village level. hey are particularly envisaged as being central to ‘local level community action’ under NRHM, which would develop to support the process of Decentralised Health Planning. Thus the committee is envisaged to take leadership in providing a platform for improving health awareness and access of community for health services, address specific local needs and serve as a mechanism for community based planning and monitoring. |
| Basudha Scheme | **Basudha Scheme** is the Odisha’s potable Water scheme. It provide safe water for drinking and domestic needs (wherever there is a shortage), on a sustainable basis |
| Pradhan Mantri Ujjwala Yojana (PMUY) | In May 2016, Ministry of Petroleum and Natural Gas (MOPNG), introduced the ‘Pradhan Mantri Ujjwala Yojana’ (PMUY) as a flagship scheme with an objective to make clean cooking fuel such as LPG available to the rural and deprived households which were otherwise using traditional cooking fuels such as firewood, coal, cow-dung cakes etc. Usage of traditional cooking fuels had detrimental impacts on the health of rural women as well as on the environment. |
| Mission Shakti | “Mission Shakti” is the self-help mission for empowering women through promotion of Women Self Help Groups (WSHGs) to take up various socio-economic activities which was launched in the state on 8th March 2001 on the eve of International Women’s Day. Mission Shakti has the clear objective of empowering women through gainful activities by providing credit and market linkage. Empowerment of women through WSHGs under Mission Shakti is a flagship programme of the Government. It envisages that over a period of time more & more women would be part of a WSHG. |
| Pradhan Mantri Kaushal Vikas Yojana (PMKVY) | Pradhan Mantri Kaushal Vikas Yojana (PMKVY) is the flagship scheme of the Ministry of Skill Development & Entrepreneurship (MSDE) implemented by National Skill Development Corporation. The objective of this Skill Certification Scheme is to enable a large number of Indian youth to take up industry-relevant skill training that will help them in securing a better livelihood. Individuals with prior learning experience or skills will also be assessed and certified under Recognition of Prior Learning (RPL). |
| Odisha Rural Development and Marketing Society (ORMAS) | To create different marketing channels for the micro enterprises, rural producers to develop sustainable livelihood through adopting appropriate rural technology, product development & diversification with accepted designs, packaging, certification, branding activities etc, Government in Panchayati Raj & Drinking Water Department has created **“Odisha Rural Development and Marketing Society** **(ORMAS)**” on 14^th^ January 1991and registered under Societies Registration Act-1860, it has been rendering yeomen services in livelihood promotion and rural product marketing in Odisha. |
| Odisha Live hood Mission (OLM) | Government of Odisha formed a society named "Odisha Livelihoods Mission (OLM)" to implement various poverty reduction programmes in the state. It is an autonomous society under the aegis of Department of Mission Shakti, Government of Odisha, presently implementing both National Rural Livelihoods Mission and National Rural Livelihoods Project. |

**Outbreak/Communicable disease reported**

Outbreak means a “sudden occurrence,” while in the epidemiological sense an outbreak is defined as a sudden increase in the disease frequency, related to time, place, and observed population.

Q.24: requesting any outbreaks or contagious diseases that have been reported in the cluster throughout the past year. It could be a disease like malaria, diarrhoea, anthrax, dengue, etc.

**Natural Calamity reported**

A natural disaster is "the negative impact following an actual occurrence of natural hazard in the event that it significantly harms a community".

Q.25: asking to get the knowledge of any natural calamity reported in the village during the last one years from the survey.

**Mobile network**

Q.26: An important question, which asks whether or not the cluster has a mobile network.

# Household Questionnaire

The purpose of the Household Questionnaire is to provide information on general characteristics of the population and their households.

## Completing the Household Questionnaire

To complete the Household Questionnaire, you will need to find a suitable respondent. Any adult member of the household who is capable of providing information needed to fill in the Household Questionnaire can serve as the respondent. If an adult is not available, do not interview a young child; instead, go on to the next household, and call back at the first household later.

Generally you will ask a single individual in the household for the information you will need to complete the household questionnaire. However, as appropriate, you may need to consult other members of the household for specific information.

## Interview Result codes

Before you go to a selected household, the household ID will be generated first in a given format. After that the result of your visit to a household is recorded. You will make every attempt to contact and interview the household, but sometimes it may happen that you make three visits to the household (at different times) and are unable to conduct the interview. In this case, you record the result of the third visit.

The following are descriptions of the various result codes:

- ***Completed***: Enter this code when you have completed the household interview.
- ***No household member at home or no competent respondent at home at time of visit:*** This code is to be used in cases in which the dwelling is occupied, but no one is at home. If no one is at home when you visit, or if there is only a child at home or an adult member who is ill, deaf, or mentally incompetent of the visit. Try to find out from a neighbour or from the children when a competent adult will be present and include this information in the visit record.
- ***Entire household absent for extended period of time:*** This code is to be used only in cases in which no one is at home and the neighbours say that no one will return for several days or weeks. In such cases. Since the neighbours may be mistaken, you should make callbacks to the household to check that no one has returned. In cases in which no one is at home and you cannot find out whether they are gone for a few hours or a few weeks, enter this.
- ***Postponed***: If you contact a household, but for some reason, it is not convenient for them to be interviewed, then schedule a callback interview and enter ‘postponed’ on the cover sheet as a result code for that visit.
- ***Refused***: The impression you make during your initial contacts with members of a household is very important. Be careful to introduce yourself and explain the purpose of the survey. Stress that the interview takes only a short amount of time and that the information will be confidential. If the individual with whom you first talk is unwilling to cooperate, ask to speak with another member of the household, such as the household head. Suggest that you can return at another time if it would be more convenient. If the individual still refuses to cooperate, enter ‘Refused’ and report the problem to your supervisor.
- ***Dwelling vacant or address not a dwelling:*** In some cases, you may find that a structure number assigned to you is unoccupied, that is, it is empty with no furniture and is not being lived in. This is what we call “vacant”. Other times, you may find that a structure is not a residential unit. It is a shop, church, school, workshop, or some other type of facility that is not used as a living area. After making sure there are no residential units in back of or above the premises, enter the result for the visit. Be sure to report the situation to your supervisor.
- ***Dwelling destroyed***: If the dwelling was burned down or was demolished in some other manner.
- ***Dwelling not found:*** You should make a thorough search, asking people in the area whether they are familiar with the address or the name of the household head. If you are still unable to locate the structure, you should enter result for the visit to that household and inform your supervisor.
- **Other**: There may be times that you cannot interview a household and the above categories do not describe the reason. Examples of cases that would fit in the ‘Other’ category would be if the entire cluster is flooded and inaccessible or if the household is quarantined because of a disease.

**Name**

Q. 1: Begin the questions with name of the household head. The person who is identified as the head of the household has to be someone who usually lives in the household. This person may be acknowledged as the head on the basis of age (older), sex (generally, but not necessarily, male), economic status (main provider), or some other reason. It is up to the respondent to define who heads the household. There generally should not be a problem with this. Enter the name with first letter of first name&/second name & last name as capital letter and others small letters.

**Age**

Q. 2: First you are to obtain each person’s age in completed years, that is, the age at the time of the last birthday. Second try to get the date of birth. You can verify the age with any Valid card. And match the DOB with the age told by the participants

**Sex**

Q. 3: Mention the sex of the participants

**Religion**

Q. 4: The religion of the head of the household is asked in order to study the differentials in health and population across different religious groups. Do not try to guess the religion of the head of the household from his/her name. Many names are common among different religions.

**Ethnic Group**

Q. 5: This question is asked to all the respondents irrespective of religion. A sizeable proportion of Christians and Muslims and persons of other religions may still have some affiliation, for example, if they were Hindus before converting to another religion. This information will be used as yet another measure of the socioeconomic background of the households. Mention here either ‘ST’ or PVTGs

**Name of the tribe/ PVTG**

Q. 6: Specify this based on the ethnic group; if the ethnic group is 'ST,' there are 62 distinct groups listed, and if the ethnic group is 'PVTG,' there are 13 different groups listed. After confirming with the participant, enter the group. Do not try to conclude that in a household all the member’s tribe/PVTGs will be same, this may be different in some of the members.

**Members in a Household**

Q. 7: A household may be one person or a group of persons who usually live and eat together. This is not the same as a family. A family includes only people who are related, but a household includes any people who live together, whether or not they are related. For example, three unrelated men who live and cook meals together would not be considered one family, but they would be considered to be members of the same household.

**Members engaged in economic activities**

Q.8: Members who are earning money their daily living.

**Monthly Expenditure**

Q. 9: Household’s usual consumer expenditure is the sum total of monetary values of all goods and services usually consumed (out of purchase or procured otherwise) by the household on domestic account during a month. This has the following components which are given below:

1. Usual expenditure for household purposes in a month.
2. Purchase value of any household durables (mobile phones, TV sets, fridge, fans, cooler, AC, vehicles, computers, furniture, kitchen equipment, etc.) purchased during the last one year and the expenditure per month obtained by dividing by 12.
3. If any household consumption (usually) from (a) wages in kind (b) home-grown stock (c) free collection was there, then the approximate monthly value of the amount usually consumed in a month was imputed.

Then the sum of A+B+C is taken as household’s usual consumer expenditure in a month in whole number of rupees.

**Gross Annual Income**

Q.11: Information on the household’s income from different sources during the last 365 days. Gross income includes all of the household income without any deductions are taken.

**Family possess the following card**

Q. 12: Get the information about the household have any of the following card or not.

- Annapurna Card

The Annapurna Scheme aims at providing food security to meet the requirement of those senior citizens who though eligible have remained uncovered under the National Old Age Pension Scheme. The target group receives 10 kgs of food grains per month free of cost. Senior citizens of 60 years and above, who are eligible for all old age pension schemes, but not covered under the same

- Anna Antyodaya Yojana Card

Antyodaya Anna Yojana (AAY) is one of the initiatives undertaken by the Government of India. It is a public distribution system scheme that was implemented in India in 2000. Depending on the annual income of the family, they will get the Subsidized food and their locality itself.

- Giving food to economically insecure families at subsidized rate
- Providing food about 35KG of rice/wheat to poor families per month
- Food grains of Rs. 3 and Rs. 2 per KG.


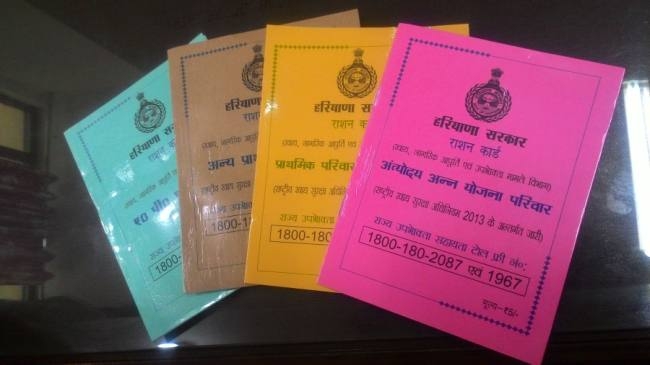


- Ration Card – National Food Security Act/Card (NFSA)

The NFSA full form is the National Food Security Act, and it has a single main purpose. This is to make food available to those who cannot afford it. Here are its objectives in more detail:

- The sole objective and that which is related to all other aims of the food security act is to enable every Indian citizen to obtain adequate food.
- Included in the same clause is the feature of provision of nutritional security by means of making food accessible quantitatively.
- Food and nutrition must be made available qualitatively at prices that individuals can afford.
- The food security act relates to the principles of the Indian Constitution that give the right to every citizen of India to live with dignity and with appropriate means of food consumption to have a healthy life.


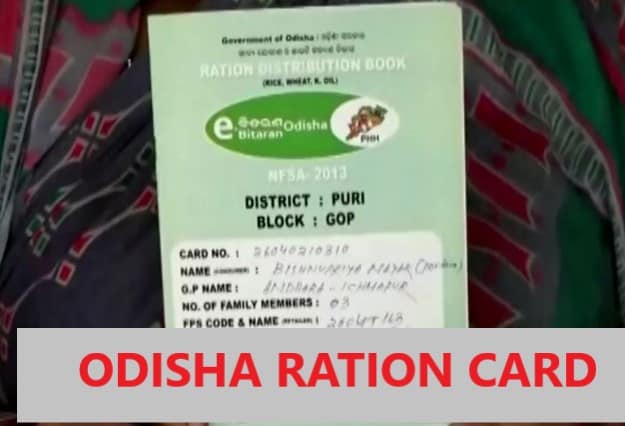


**Health Scheme or Health Insurance**

In the survey, information was collected on the awareness, accessibility and coverage of various health insurance schemes provided by the central and state governments and private insurers. The awareness of and accessibility to health insurance schemes is important for all households, irrespective of their socio-economic status, as it will help people to improve their health-seeking behaviour and prevent catastrophic health expenditure.

Interviewer should explain about health insurance as a type of insurance coverage that pays for medical and surgical expenses that are incurred by the insured. Health insurance can either reimburse the insured for expenses incurred from illness or injury or pay the care provider directly.

- **Employees State Insurance Scheme (ESIS)**

The [Employees' State Insurance Scheme](http://esic.nic.in/index.php) is an integrated measure of Social Insurance embodied in the Employees' State Insurance Act and it is designed to accomplish the task of protecting '**employees**' as defined in the **Employees' State Insurance Act, 1948** against the impact of incidences of sickness, maternity, disablement and death due to employment injury and to provide medical care to insured persons and their families. The ESI Scheme applies to factories and other establishment's viz. Road Transport, Hotels, Restaurants, Cinemas, Newspaper, Shops, and Educational/Medical Institutions wherein 10 or more persons are employed. Employees of the aforesaid categories of factories and establishments, drawing wages upto Rs.15,000/- a month, are entitled to social security cover under the ESI Act. ESI Corporation has also decided to enhance wage ceiling for coverage of employees under the ESI Act from Rs.15,000/- to Rs.21,000/-


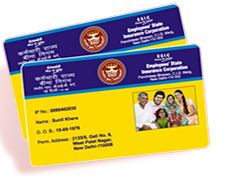


- Central Govt. Health Schemes (CGHS)

Central Government Health Scheme is providing comprehensive medical care to the Central Government employees and pensioners enrolled under the scheme. In fact CGHS caters to the healthcare needs of eligible beneficiaries covering all four pillars of democratic set up in India namely Legislature, Judiciary, Executive and Press. CGHS is the model Health care facility provider for Central Government employees & Pensioners and is unique of its kind due to the large volume of beneficiary base, and open ended generous approach of providing health care.

CGHS provides health care through following systems of Medicine Allopathic, Homoeopathic, Ayurveda, Unani, Siddha and Yoga

- Biju Swasthya Kalyan Yojana (BSKY)

To provide assurance of quality health care to all the citizens of the State, especially the vulnerable sections. With this objective, Biju Swasthya Kalyan Yojana has been launched is as a pathbreaking program to provide universal health coverage, with special emphasis on the health protection of economically vulnerable families. To achieve its objectives, the BSKY has two components:

State Government will bear full cost of all health services delivered to all patients (irrespective of income, status or residence) in all State Government health care facilities starting from Sub centre level to District Head Quarter and Government Medical College Hospital and Blood Bank level.

State Government will bear the cost of healthcare provided in empanelled private hospitals for over 96.5 lakh economically vulnerable families in the State, amounting to Annual Health coverage of Rs. 5 lakh per family and additional Rs. 5lakh for the women members of the family after exhaust of initial limit.


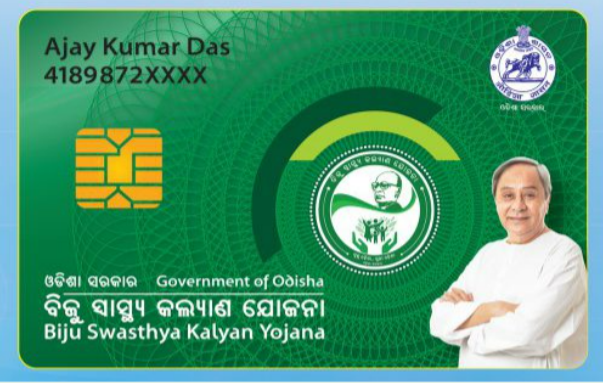


- Rashtriya Swasthya Bima Yojana (RSBY)

RSBY has been launched by Ministry of Labour and Employment, Government of India to provide health insurance coverage for Below Poverty Line (BPL) families. The objective of RSBY is to provide protection to BPL households from financial liabilities arising out of health shocks that involve hospitalization.


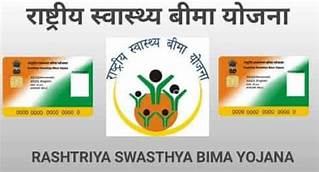


- Odisha State Treatment Fund (OSTF)

The Odisha State Treatment Fund will provide financial assistance to the poor patients who are suffering from life threatening disorders & diseases, for treatment of their major ailments. The financial assistance will be released to the Medical Institutions in which treatment has been/is being given to the patients. The fund is managed by an autonomous society known as “ODISHA STATE TREATMENT FUND SOCIETY”. The Society is registered under the Societies Registration Act, 1860.

- Community Health Insurance Programme
- Other Health Insurance Through Employer
- Other Privately Purchased Commercial Health Insurance

**Injury**

Q16. Ask about that any of the member in the household had any injury that warranted the need for medical treatment or has altered your daily activities for one day or more over the last year. And if any member had any injury, get the details about that from Q17 to Q22.

**Source of water**

The purpose of this question is to assess the cleanliness of the household drinking water by asking about the household’s main source of water. If drinking water is obtained from several sources, probe to determine the source from which the household obtains the majority of its drinking water. If the source varies by season, record the main source used at the time of interview.

| Definitions of Water Source Codes for Q. 29 | |
| --- | --- |
| ***Response Categories*** | ***Definition*** |
| Surface water | Water located above ground and includes rivers, dams, lakes, ponds, streams, canals, and irrigation channels.  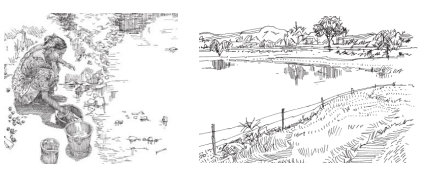 |
| Piped into dwelling | Pipe connected with in-house plumbing to one or more taps, e.g. in the kitchen and bathroom. Sometimes called a house connection.  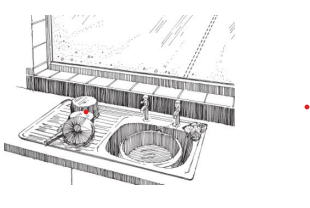 |
| Piped to yard/plot | Pipe connected to a tap outside the house in the yard or plot. Sometimes called a yard connection.  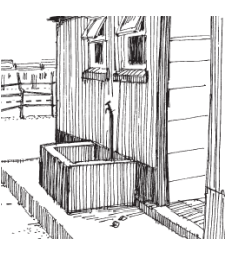 |
| Public tap or standpipe | Public water point from which community members may collect water. A standpipe may also be known as a public fountain or public tap. Public standpipes can have one or more taps and are typically made of brickwork, masonry or concrete.  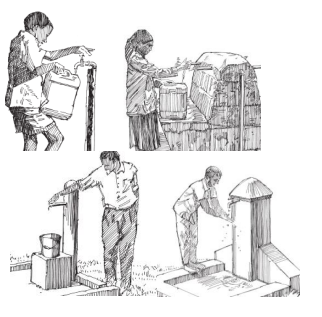 |
| Tube well or borehole | A deep hole that has been driven, bored or drilled with the purpose of reaching ground water supplies. Water is delivered from a tubewell or borehole through a pump which may be human, animal, wind, electric, diesel or solar-powered.  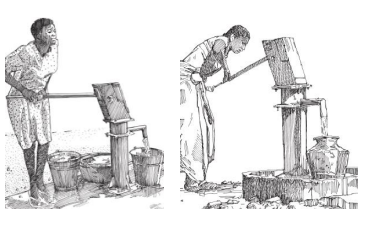 |
| Protected dug well | A dug well that is (1) protected from runoff water through a well lining or casing that is raised above ground level and a platform that diverts spilled water away from the well and (2) covered so that bird droppings and animals cannot fall down the hole. Both conditions must be observed for a dug well to be considered as protected.  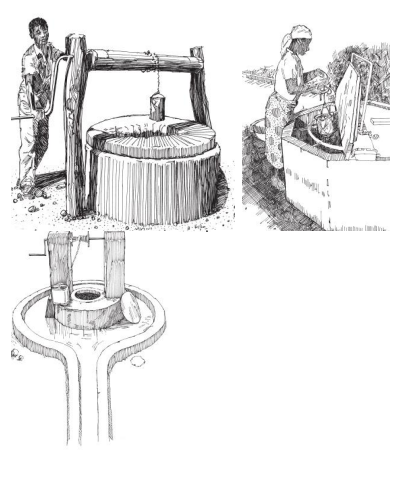 |
| Unprotected dug well | A dug well which is (1) unprotected from runoff water; (2) unprotected from bird droppings and animals; or (3) both.  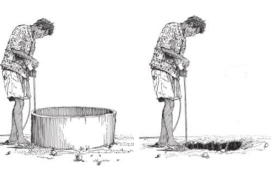 |
| Protected spring | A spring protected from runoff, bird droppings, and animals by a “spring box” which is typically constructed of brick, masonry, or concrete and is built around the spring so that water flows directly out of the box into a pipe without being exposed to outside pollution.  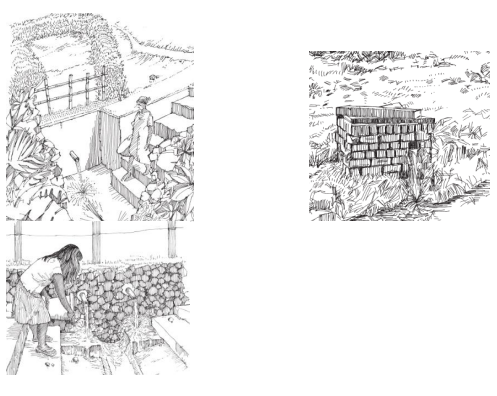 |
| Unprotected spring | A spring that is subject to runoff and/or bird droppings or animals. Unprotected springs typically do not have a “spring box”. |
| Rainwater | Rain that is collected or harvested from surfaces by roof or ground catchment and stored in a container, tank or cistern.  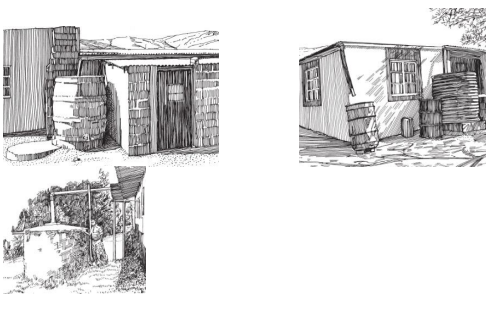 |
| Tanker truck | Water is obtained from a provider who uses a truck to transport water into the community. Typically, the provider sells the water to households.  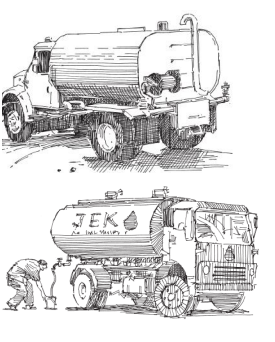 |
| Cart with small tank | Water is obtained from a provider who transports water into a community using a cart and then sells the water. The means for pulling the cart may be motorized or non-motorized (e.g., a bullock).  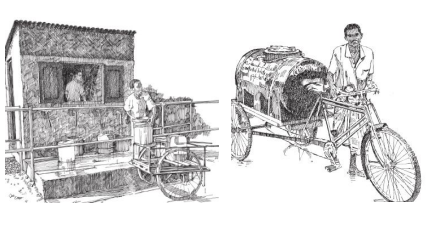 |
| Bottled water | Water that is bottled and sold to the household in bottles. |
| Community RO Plant | Reverse Osmosis (RO) is a process for creating safe drinking water by forcing water under high pressure through a filter. |

**Treatment of Drinking Water**

The purpose of Q.30 is to know whether the household drinking water is treated within the household and if so, what type of treatment is used. The type of treatment used at the household level provides an indication of the quality of the drinking water used in the household.

| Definitions of Water Treatment Codes for Q. 30 | |
| --- | --- |
| Directly drink source water | Drinking water from direct source of getting water |
| Boil | Boiling or heating of water. |
| Using filter | The water flows through media to remove particles and at least some microbes from water. Media used in filtering systems usually include ceramic, sand and composite. |
| Electronic water purifier | A machine which runs on electricity and is used for purifying water |
| Strain it through a cloth | Pouring water through a cloth which acts as a filter for collecting particulates from the water. |
| Let it stand and settle | Holding or storing water undisturbed and without mixing long enough for larger particles to settle to the bottom by gravity |

**Toilet Facility**

The purpose of this question is to obtain a measure of the sanitation level of the household, since toilet facilities are important for disease control and health improvement. If the respondent answers in general terms such as “flush toilet,” probe to determine where the toilet flushes to; likewise, if the respondent answers “latrine”, probe to determine the type of latrine. Below are some definitions for the terms used in the codes for Q. 32.

| Definitions of Toilet Facility Codes in Q. 32 | |
| --- | --- |
| Response Categories | Definition |
| Flush/pour flush toilet | A flush toilet uses a cistern or holding tank for flushing water and has a water seal, which is a U-shaped pipe, below the seat or squatting pan that prevents the passage of flies and odours. A pour flush toilet uses a water seal, but unlike a flush toilet, a pour flush toilet uses water poured by hand for flushing (no cistern is used). |
| - to piped sewer system | A system of sewer pipes (also called sewerage) that is designed to collect human excreta (faeces and urine) and wastewater and remove them from the household environment. Sewerage systems consist of facilities for collection, pumping, treating and disposing of human excreta and wastewater.  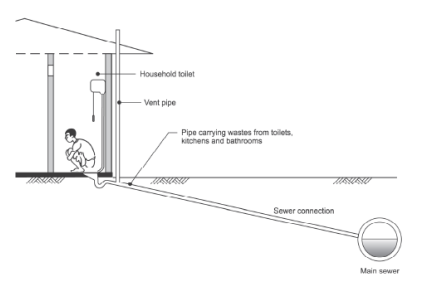 |
| - to septic tank | An excreta collection device consisting of a water-tight settling tank normally located underground, away from the house or toilet.  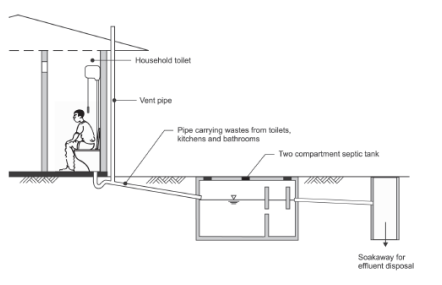 |
| - to pit latrine | A system that flushes excreta to a hole in the ground. |
| - to somewhere else | A system in which the excreta is deposited in or nearby the household environment in a location other than a sewer, septic tank, or pit, e.g., excreta may be flushed to the street, yard/plot, drainage ditch or other location. |
| Pit latrine | Excreta are deposited without flushing directly into a hole in the ground. |
| - ventilated improved pit latrine (VIP)/biogas latrine | A latrine ventilated by a pipe extending above the latrine roof. The open end of the vent pipe is covered with gauze mesh or fly-proof netting and the inside of the superstructure is kept dark.  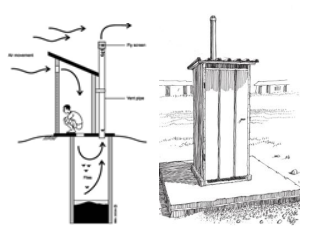 |
| - pit latrine with slab | A latrine with a squatting slab, platform or seat firmly supported on all sides which is raised above the surrounding ground level to prevent surface water from entering the pit and for ease of cleaning.  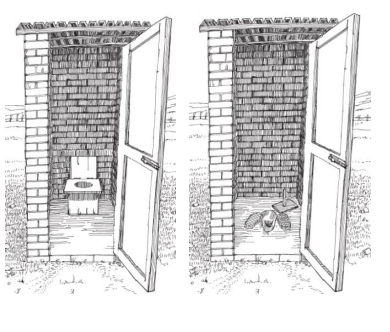 |
| - pit latrine without slab/ open pit | A latrine without a squatting slab, platform or seat. An open pit is a rudimentary hole in the ground where excreta is collected  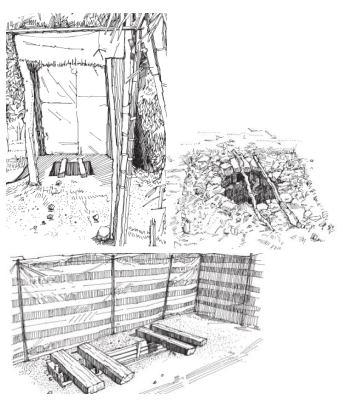 |
| Twin pit/Composting toilet | A toilet into which excreta and carbon-rich material are combined (vegetable wastes, straw, grass, sawdust, ash) and special conditions maintained to produce inoffensive compost.  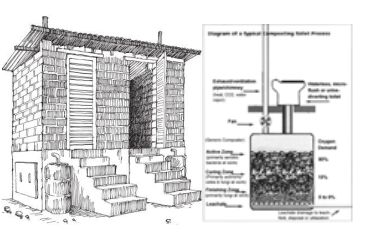 |
| Dry toilet | A place that is used for defecation and from which the faeces are regularly picked-up by a scavenger (usually found in old cities). Known as kamau paikhana in Hindi. |

**Shared toilet facilities**

Q. 34 asks about whether the toilet facilities are shared with other households. In Q. 35, we want to find out how many households, including the respondent’s household, use the same facility. For example, if the respondent’s household shares the toilet with one other household, record “02” in Q. 35. If they share it with two other households, record “03” in Q. 35. The number of households that share toilet facilities is an important measure of the level of hygiene in the household.

**Number of rooms for Sleeping**

Q. 38 Simply ask about the number of rooms that the household uses for sleeping. Include all the rooms where persons in the household sleep, even if those rooms are used for other purposes in the daytime (that is, they are not exclusively used for sleeping). If the household members usually sleep outside the house, you would record ‘00’ for the number of rooms used for sleeping.

**Fuel for Cooking**

Q.41 Information on the type of fuel used for cooking is collected as another measure of the socioeconomic status of the household. The use of some cooking fuels can also have adverse health consequences. Remember that this question asks about fuel for cooking, not fuel for heating or lighting. The category ‘biogas’ includes gases produced by fermenting manure in an enclosed pit. If the household uses more than one fuel for cooking, find out the fuel used most often. If any fuel other than the precoded ones is reported as being the main fuel used for cooking, record ‘96’ and specify the type of fuel in the space provided.

**Use of Iodized salt**

Q. 42 The purpose of this question is to get whether the household uses salt that has been fortified with iodine in cooking. Fortified salt prevents iodine deficiency. Iodine is an important micronutrient and a 38 lack of it may lead to an enlarged thyroid gland in the neck known as goitre or other thyroid-related health problems.

**Place where food is cooked**

The purpose of Q. 43 is to collect information on the location where food is prepared in the household: in the household, in a separate building, or outdoors. This information is important in providing an indicator of the air quality inside and around the dwelling. In Q. 44, information on whether the household has a separate room used as a kitchen provides additional information on the hygiene status of the household.

**Ownership of livestock, herds, poultry or other farm animals**

Q45. Information on whether households own any livestock, herds, poultry or other animals and how many they own is used as an additional indicator of the socioeconomic status of the household. Read out each item and be sure to record yes or no for each item. The number of animals owned does not matter, only that one or more is owned by the household or a usual member of the household.

Q. 46 – Q. 65 are related to the information about the keeping of livestock, handling the waist of livestock, vaccination of livestock, dealing with the dead livestock, details about consumption of meat and related to anthrax.

**Possession of mosquito nets**

It is recognized that the consistent use of insecticide-treated mosquito nets (ITN) decreases the incidence of malaria and malaria-related deaths, especially in very young children. Consequently, many countries are now instituting programs that promote the use of ITNs. Qs. 66-67 inquiries about whether the household has any mosquito nets, and if yes, how many. It does not matter if the nets are actually used or even if they are set up. If they are in the household and could be used while sleeping, they should be counted. Note that ‘cake covers’ or baby nets that are used to keep flies off infants, usually during the daytime, are not considered mosquito nets. These nets cannot be treated with insecticide. Window screens are also not considered mosquito nets. Q. 61 asks where these mosquito nets were obtained. Record all answers given.

**Household items**

The answers to these questions on ownership of certain items will be used as a rough measure of the socioeconomic status of the household. Read out each item and record the answer given after each item. Do not leave any item(s) blank. If the respondent reports that a household item such as a radio is broken, try to find out how long it has been broken and whether it will be fixed. If the item appears to be out of use only temporarily, record YES. Otherwise, for NO.

**Floor material**

This is an observation not a question since you will usually be able to see for yourself what kind of floor the house has. However, ask if you are not sure. If there is more than one kind of flooring material, record the main type of material (the material that covers the largest amount of floor space).

**Roof material**

As with the floor material, you will usually be able to see for yourself what kind of roof material the house has. However, observing the roof material may not always be easy or you may be able to observe part but not the whole roof. Ask the respondent if you are not sure or if you cannot observe the roof properly. If the household lives in an apartment building, look at the roof from a reasonable distance and ask the respondent if necessary. If there is more than one kind of roofing material, record the main type of material (the material that covers the largest amount of roof space).

**Wall material**

As with the floor and roof materials, you will usually be able to see for yourself what kind of material the exterior walls are made of. However, ask the respondent if you are not sure. Again, if there is more than one kind of wall material, record the main type of material (the material that covers the largest amount of wall space).

**Disability Status**

Q. 74 & Q. 75 asking about the any of the member in the household have any type of disabilities. This may, hearing, speech, visual, mental, locomotor etc., if Q.75 yes then get the details about the disability in Q.75

**Dietary intake**

Q. 76 details about the food intake habit of a household. This will assess the whether the household members getting adequate nutritional food or not.

**Details of recent death(s) in household**

These questions are asked to determine if any usual member of the household died during the past 1 years, i.e., since July 2021. If NO usual member died since July 2021 then END THE INTERVIEW. If YES, ask Q. 78 to determine how many usual members died. In continuation also asked to gather details like name, sex, age at death, and cause of death of each usual member who died. If the person who died is a female and died when she was 12 years or older then ask whether the death was a maternal death, i.e., she died during pregnancy, during childbirth or within two months after the end of pregnancy or childbirth.

# Under 5 Years Old Questionnaire

Determine how many children under the age of 5 live in the household before beginning the intervies. The number of interviews will be conducted in proportion to the number of children, with one interview per child.

***Section 1:  Information about the child of age less than 5 years***

**Q. 101: Number of under 5 years children**

Begin the interview with asking with the number of under 5 years old children are there in the household. According to this number the number of interviews will be conducted.

**Q. 102: Name of the Child**

Record the name of each child, beginning with the first born and continuing until the last born. If the woman reports that she had a multiple birth (twins, triplets, etc.), record each of the children on a separate line. Write the name that distinguishes that child from the others. For example, if there are two children, Ravi Kumar and Manoj Kumar, write “Ravi” and “Raj,” not “R. Kumar” and “M.” If the baby never had a name, either because he/she is still very young or because he/she died very young, write “Baby” for the name.

**Q. 103. Month and Year of birth**

When collecting information on a child’s birth date, always look at any documents you collected for the child at the beginning of the interview (e.g., birth certificate or immunization record) to see whether a date of birth was recorded. Before entering a date from these documents, however, check with the respondent to determine whether she believes the date is accurate. In some cases, the information on the document may be the date when the birth was recorded and not the date when the child was born. If the respondent gives you a year of birth but does not know the month of birth, probe to try to estimate the month. Example: If she says her daughter was born in 1997, but she does not know which month, ask her whether she gave birth in the dry season or the monsoon season, whether she remembers if she was pregnant at Diwali time or at Holi, for example, or during some other significant event/season of the year to try to determine the month of birth. Convert months to numbers, as before. If you cannot even estimate a month, record ‘98’ for MONTH. If the month of birth is reported in terms of Hindu or Islamic months, use the conversion tables to convert Hindu or Islamic months into Gregorian months. If the respondent cannot recall the year when the birth occurred, you need to probe carefully. See if the respondent knows a firm birth date for any other child in the household and relate it to that. Example: If she knows the second child was born in 1999 and the first child was just a year old at that time, record ‘1998’.

**Q. 104: Age of child**

The age of all living children should be recorded in completed years. Example: A child who will become three years old next month should be recorded as ‘02’ years today. A child less than one year old will be recorded as age ‘00’ years. Sometimes, a mother will not know the current age of her child. In this case, you may rephrase the question to, “How many years ago was Ravi born?” You can also use other available information such as relating John’s age to the age of a child she does know. Example: The mother may know that her youngest child was born one year ago and that Ravi was around two years old at that time, in which case Ravi would be three years old now. You MUST record an age for all children who are still alive.

**Q. 105: Birth Order of child**

Birth order refers to the order a child is born in their family; first-born and second-born are examples. If the child is second-born then record as 2.

**Q. 106: Child’s Sex**

Record the code for the sex of the child. Although you can often tell the sex from the name, check with the respondent by saying, for example, “and ‘Jaya’ is a girl?” Do not assume the sex of the child from the name.

**Q. 107: Religion**

The religion is asked in order to study the differentials in health and population across different religious groups. Do not try to guess the religion of the individual of the household from his/her name. Many names are common among different religions.

**Q. 108: Ethnic Group**

In Q.108, Record here Scheduled Tribe (ST) or Particularly Vulnerable Tribal Groups (PVTGs). PVTGs groups who are relatively more isolated, archaic, vulnerable, deprived and backward. These tribal groups are the most disadvantaged among the tribals. They live in small, scattered habitats in remote, inaccessible areas. Their livelihoods are especially vulnerable because over the years, the more dominant tribal and non-tribal groups have encroached upon the resources which they originally controlled and accessed for their survival. Despite numerous government schemes to mainstream these groups the results have fallen short of expectations. Since the 5th Five Year Plan when the Tribal Sub Plan (TSP) approach was adopted in the Country, they have been initially identified on the basis of certain criteria prescribed and designated as Primitive Tribal Groups (PTGs) and recently re-designated as Particularly Vulnerable Tribal Groups (PTGs) by Government of India for the purpose of receiving special attention for their all-round development.

In Q.109 list out the name of the ST or PVTG. There are listed 62 ST groups and 13 PVTG groups are there in Odisha.

***Section 2:  Child’s Vaccinations and Vitamin A Supplementation***

**Vaccination Card**

You should have obtained documentation (birth certificates and vaccination cards) for eligible children at the beginning of the interview. If you have not already collected the vaccination card(s), ask the respondent to look for the card(s). In some cases, the respondent may hesitate to take time to look for the card(s), thinking that you are in a hurry. Since it is critical to obtain written documentation of the immunization history for all eligible children, be patient if the respondent needs to search for the card(s).

If, in Q. 203 the woman tells you she does not have a vaccination card for her child, ask her in this question whether she ever had a card for that child. It is possible that she at one time did have a card, but no longer has it.

In Q.204 If the respondent shows you the card for a child, record YES, SEEN. If the respondent says the child has a vaccination card, but she is unable to show it to you because she has lost it, someone else has it, or it is not accessible to her during the interview, record YES, NOT SEEN for that child. If the respondent says she does not have a card for her child, record NO CARD. Each response has a different skip instruction, so be careful to follow the correct skip pattern.

**Recording the Vaccinations**

If you have a vaccination (health) card for the child, fill in the responses to Q. 509, taking the information directly from the card. When there is more than one eligible child, be certain to match the correct card with the child you are asking about. The card may list the vaccinations in a different order than the questionnaire. Check the card carefully and record the vaccination received by the child and number of doses received

**Vaccinations for children with no card**

If you did not see a child’s vaccination card and the respondent tells you that the child did receive at least one vaccination, you will ask about whether the child received each of the following vaccinations: BCG, polio, DPT, Hepatitis B, and measles/MMR. Ask Qs. 206 - 225, following the appropriate skip patterns. Because there are many types of vaccines, we specify how each one is given so the mother will know which vaccine we are asking about. Read the whole sentence before accepting the woman’s response. Notice that there are follow-up questions for the polio, DPT, and Hepatitis B vaccinations. For the polio vaccine, we ask whether the child received it, when the child first received it, and how many times the child received it. For the DPT and Hepatitis B vaccinations, we ask whether the child received the vaccination and how many times.

**Source of Vaccinations**

For children who have received any vaccinations (either listed on the vaccination card or from the mother’s recall), ask the respondent where the child received most of his/her vaccinations (Q. 226). If the child has received only one vaccination, ask where he/she received that vaccination. In either case, ask the respondent whether the place is in the public (run by the government) or private sector.

***Section 3:  Child Feeding Practices***

**Child ever breastfed**

Breastfeeding is important for fertility and child health. For this question (Q. 301), it does not matter how long the respondent breastfed the child, only whether or not she ever gave the child the breast, even if the baby died very young.

**When breastfeeding began**

Q. 302; If the mother reports that the baby was put to the breast immediately after birth, record ‘000.’ Otherwise, record the time in completed hours or days. Examples: The woman said she began breastfeeding within an hour of the birth. Record ‘1’ and record ‘00’ hours. The woman said she began breastfeeding 30 hours after the birth. Record ‘2’ (DAYS) and record ‘01’.

**Still breastfeeding**

Q. 303 is only asked if the child is still alive. Note that it does not matter whether she is giving the child other liquids or foods as well; we are interested in knowing whether the child is being breastfed at all.

Q.304 is the continuation of Q. 303, ask about the reason/s for not breastfeeding the child.

**Bottle with nipple**

The use of bottles with nipples can be unsanitary and can indicate early or inappropriate weaning. You should record ‘YES’ if the child was given anything in a bottle during the day or night before the interview.

**Frequency of breastfeeding**

Q. 307 is asking about the frequency of breastfed yesterday day & night

**Liquids and foods given yesterday**

The purpose of this series of questions is to obtain a better picture of the diversity of the child’s diet.

• Begin by reading the introductory portion of the question slowly, emphasizing that the question concerns what the child drank or ate yesterday during both the day and night. Then ask about each of the items in the order they appear in the question. Be careful to record the response (“YES,” “NO” OR “DON’T KNOW”) for an item before asking about the next item.

• For categories that have more than one item of food or drink, record ‘1’ for “YES” if any item in that category was given. For questions regarding consumption of milk, infant formula, and yogurt, follow up by asking how many times the child consumed the item.

• As you are asking about the initial items, the mother may interrupt and list the foods and liquids that her child consumed. Begin with the foods or liquids she mentions that the child received and record a ‘1’ for each item. You may need to ask the woman to repeat the items to make sure that you have recorded all the food types correctly.

• Sometimes the mother may tell you that the child was given “vegetable soup” or “meat stew”. Since these typically include a variety of food types, it is important that you probe to find out the ingredients included in the soup, porridge or stew. For example, if the mother tells you her child had soup, ask what was in the soup and record all the ingredients. If the soup contained carrots, white potatoes, and beef, record YES in the category of “Pumpkin, carrots, squash or sweet potatoes that are yellow or orange inside” to record the carrots, YES in the category of “White potatoes, white yams, manioc, cassava, or any other foods made from roots” to record the white potatoes, and YES in the category of “Any meat, such as beef, pork, lamb, goat, chicken, or duck” to record the beef.

• The category “clear broth” refers only to clear water-based soups. Soups that include pieces should not be included here, but should be handled as described above.

• Once you have entered all of the foods or liquids that the child consumed, you must go back and ask about any categories which the woman did not mention. If the woman now mentions a food item the child ate yesterday (or had mentioned it before) which is not listed in any of the existing food groups, record ‘1’ in if it is a liquid and ‘1’if it is a solid or semi-solid food. If the respondent tells you that her child was given only the items she has already mentioned (e.g., infant formula and juice), confirm that the child was not given anything else (e.g., by asking “Did (NAME) drink any other liquid at all?” and “Was (NAME) given any other solid or semi-solid food?”).

If the mother confirms that the child was not given any other liquid or food, mark NO for all the other items in the list. Note that if the woman mentions breast milk, it is not necessary to record this information in Q.308 because if the child had breast milk yesterday, the mother would have already told us back in that she is still breastfeeding. Note also that (“Plain water?”) refers to water by itself with nothing added to it. If the mother was not with the child on the day before you conduct the interview, she may not be able to answer these questions. If this situation occurs, ask if you can talk to the person who was responsible for the child’s care while the mother was away about what the child ate. It is also possible that the mother will consult other household members about what the child ate even if she was at home since the mother may not be the only one who fed the child yesterday. If at any time when you are asking Q. 308 you obtain information on liquids or foods the child was given from other household members, you should write down the names of these individuals and a description of the circumstances on the same page as the relevant question. Also put a note in the OBSERVATION section at the end of the interview. Before going on to the next question, check that there is a response recorded for each item in Q. 308. Note: in some parts of the world, use of infant formula and commercially fortified baby cereal is widespread. In other areas, these products may be uncommon, and respondents may not recognize the terms. Definitions of each are provided below: • Infant formula. Infant formula is a commercial product that can be used to provide all or part of the nutrients that infants need for growth and development. Formula may be a powder or a liquid concentrate, either of which must be mixed with water before it is given to an infant. Alternatively, formula can be packaged in a ready-to-use container that can be fed to an infant without adding water. • Commercially fortified baby cereal. Commercially produced cereals specifically produced for feeding to infants or young children. Common infant cereal products available in India include Cerelac and Farex.

***Section 4:  Treatment of Childhood Diseases***

**Syndrome/Disability**

Q401 question asking know whether the child is having any of the syndrome / disability.

- **Cleft lip**

A cleft lip happens if the tissue that makes up the lip does not join completely before birth. This results in an opening in the upper lip. The opening in the lip can be a small slit or it can be a large opening that goes through the lip into the nose. A cleft lip can be on one or both sides of the lip or in the middle of the lip, which occurs very rarely.


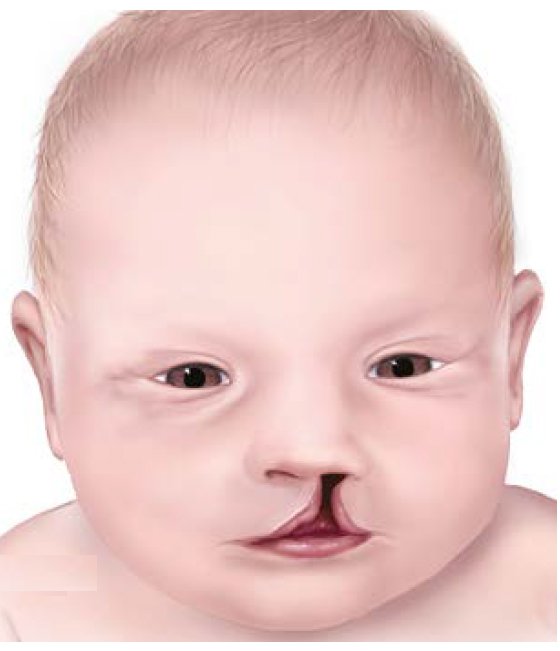


- **Cleft palate**

The roof of the mouth (palate) is formed between the sixth and ninth weeks of pregnancy. A cleft palate happens if the tissue that makes up the roof of the mouth does not join together completely during pregnancy. For some babies, both the front and back parts of the palate are open. For other babies, only part of the palate is open.


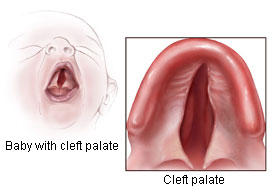


- **Down Syndrome**

Down syndrome is a condition in which a person has an extra chromosome. Chromosomes are small “packages” of genes in the body. They determine how a baby’s body forms and functions as it grows during pregnancy and after birth. Typically, a baby is born with 46 chromosomes. Babies with Down syndrome have an extra copy of one of these chromosomes, chromosome 21. A medical term for having an extra copy of a chromosome is ‘trisomy.’ Down syndrome is also referred to as Trisomy 21. This extra copy changes how the baby’s body and brain develop, which can cause both mental and physical challenges for the baby.


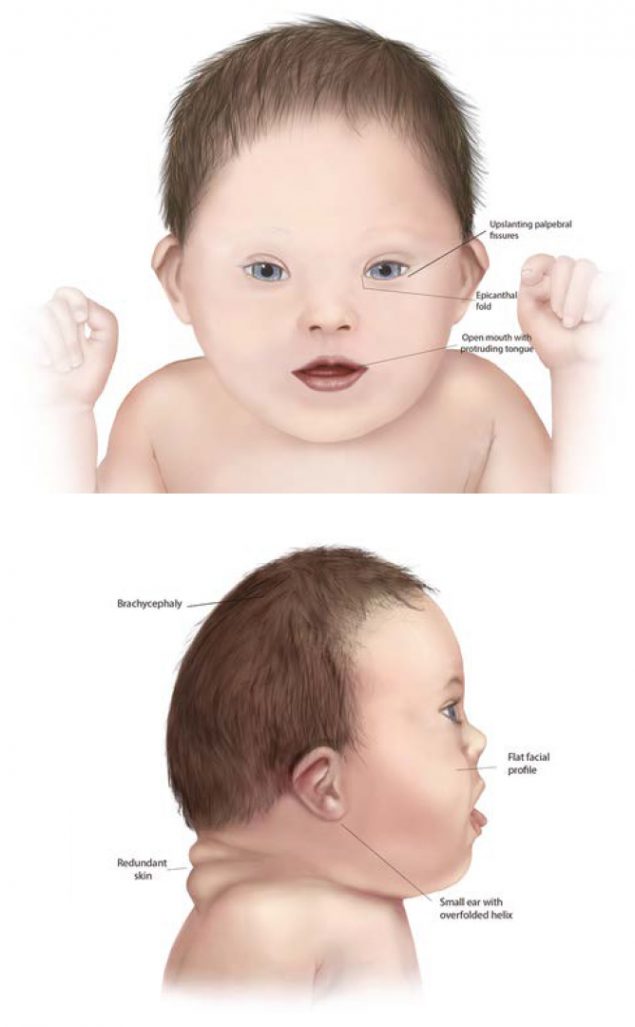


- **Congenital heart defects**

Congenital heart defects (CHDs) are conditions that are present at birth and can affect the structure of a baby’s heart and the way it works. They are the most common type of birth defect. As medical care and treatment have advanced, infants with congenital heart defects are living longer and healthier lives. Many now are living into adulthood.

- **Oral health problems (Dental)**

Cavities (also known as caries or tooth decay) are one of the most common chronic diseases of childhood. Untreated cavities can cause pain and infections that may lead to problems with eating, speaking, playing, and learning.


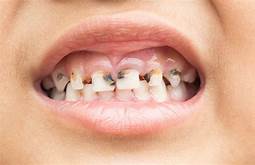


- **Dwarfism**

Dwarfism is short stature that results from a genetic or medical condition. Dwarfism is generally defined as an adult height of 4 feet 10 inches (147 centimeters) or less. The average adult height among people with dwarfism is 4 feet (122 cm).

- **Autism**

Autism spectrum disorder (ASD) is a developmental disability caused by differences in the brain. Some people with ASD have a known difference, such as a genetic condition. Other causes are not yet known. Scientists believe there are multiple causes of ASD that act together to change the most common ways people develop. People with ASD may behave, communicate, interact, and learn in ways that are different from most other people. There is often nothing about how they look that sets them apart from other people.  The abilities of people with ASD can vary significantly. For example, some people with ASD may have advanced conversation skills whereas others may be nonverbal. Some people with ASD need a lot of help in their daily lives; others can work and live with little to no support.

- **Epilepsy**

Epilepsy is a central nervous system (neurological) disorder in which brain activity becomes abnormal, causing seizures or periods of unusual behavior, sensations and sometimes loss of awareness.

- **Abnormality is Vision/ speech/ hearing**

Vision, hearing and speech are an important part of a child's life. When an infant is born, his or her eyesight is immature. The infant later develops the ability to focus. Hearing appears early in fetal development and is necessary for proper progression of speech and language.

- **Mentally Challenged**

This classification is given to children with poor IQ, typically in the range of 70-75 or less. They have low adaptive behaviour or daily living skills (eating, dressing, communication and social skills they are slower than their peers in acquiring life skills such as speech development or logic.

- **Sickle cell disease**

Sickle cell disease (SCD) is a group of inherited red blood cell disorders. Red blood cells contain hemoglobin, a protein that carries oxygen. Healthy red blood cells are round, and they move through small blood vessels to carry oxygen to all parts of the body. In someone who has SCD, the hemoglobin is abnormal, which causes the red blood cells to become hard and sticky and look like a C-shaped farm tool called a “sickle.” The sickle cells die early, which causes a constant shortage of red blood cells. Also, when they travel through small blood vessels, they get stuck and clog the blood flow. This can cause pain and other serious complications (health problems) such as infection, acute chest syndrome and stroke.

**Symptoms in the last one month**

Q402 is about gathering information regarding various symptoms experienced in the month preceding to the survey. Inquire with the mother if the kid has experienced any of the symptoms described.

**Drinking and eating during diarrhea**

**Q. 403 & Q. 404** The amount of fluids or food given while a child has diarrhea may be different than normal. Read the entire question before accepting a response. We are interested in knowing the amount of fluids and/or food the child ate or drank. If a respondent says “less” probe to determine more specifically if she meant “much less” than usual or “somewhat less”.

**Places where advice/treatment for diarrhea/fever sought**

Check the responses from the Q.402, if the child suffered from diarrhea/fever in the last month ask Q.405 to get know the place where advice/treatment for diarrhea was sought. Asking this question to know about mainly how may are preferring public facilities.

**Anemia**

Check the responses from the Q.402, if the child had anemia in the last month ask Q.406 to get the information about what was treatment taken by the child.

**Blood Sugar**

Q. 407 – Q. 410 questions are related to get the information about child’s blood sugar details.

***Section 5:  Rating of health***

Self-rating of health is taken as EQ visual analogue scale (EQ VAS). It records the respondent’s self-rated health on a vertical, visual analogue scale where the endpoints are labelled ‘Best imaginable health state’ and ‘Worst imaginable health state’. This information can be used as a quantitative measure of health outcome as judged by the individual respondents.

The EQ VAS should be scored, for example, as follows:


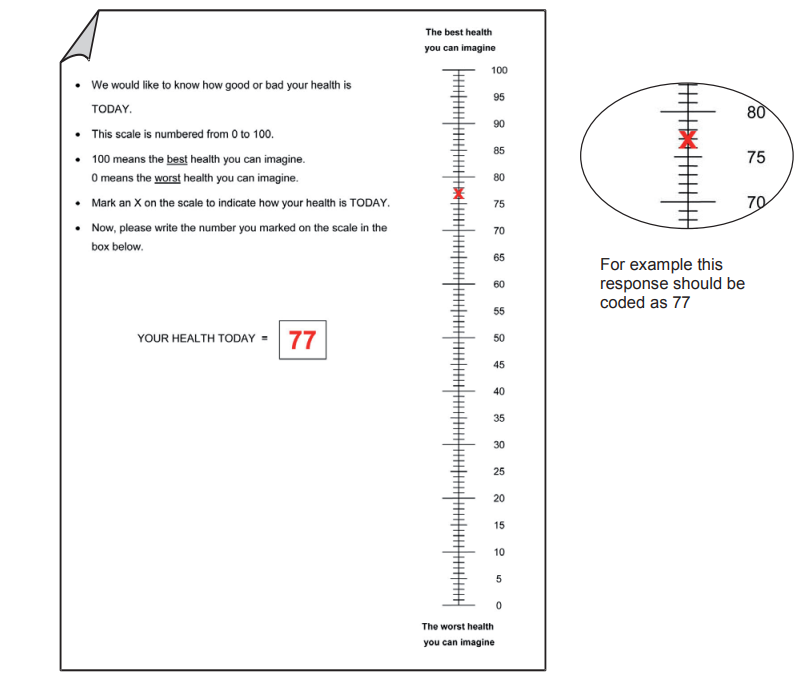


# 5 To 9 Years Old Questionnaire

Determine how many children under the age of 10 live in the household before beginning the interview. The number of interviews will be conducted in proportion to the number of children, with one interview per child.

***Section 1:  Information about the child***

See the Section 1 above in under 5 years old questionnaire section from Q.101 & Q. 109.

**Schooling of the Child**

Q. 110 – Q. 111 are asking to know about that at present the child is going to school or not and if going to school in which class the child is studying.

***Section 2:  Treatment of Childhood Diseases***

See Section 2 above in Under 5 years old questionnaire section.

***Section 3:  IFA Supplementation and deworming medications***

Here in section 3 The child will be telling to narrate their experience of Iron Folic Acid (IFA) and deworming medication consumption.

Iron deficiency Anaemia adversely affects transport of oxygen to tissues and results in diminished work capacity and physical performance. During adolescence, iron deficiency anaemia can result in impaired physical growth, poor cognitive development, reduced physical fitness and work performance and lower concentration on daily tasks. According to the recommendation by Ministry of Health and Family Welfare (MoHFW), India, children with 6 – 10 years old take 30 mg elemental iron and 250 mcg folic acid per child per day for 100 days in a year (Ref. <https://www.nhm.gov.in/images/pdf/programmes/child-health/guidelines/Control-of-Iron-Deficiency-Anaemia.pdf>).

| 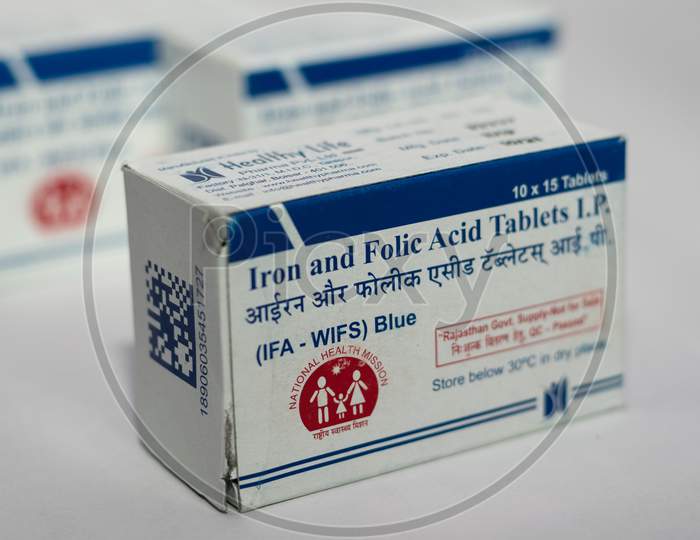 | 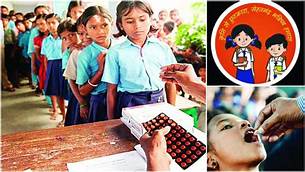 |
| --- | --- |

Ask Q.302 & Q. 303 to get the detailed information about that the child is receiving IFA tablets/deworming medication or not, if received what is the source and frequency of getting the IFA tablets/deworming medications and then ask about whether the child is consuming those as per the recommendation or not.

***Section 4:  Mid-day Meal programme (MDM) & Absenteeism***

This section is asking to the child who is currently going to school. Confirm this from the Q.110.

The Indian Mid-day Meal (MDM) programme provides nutritional support in the form of school-served lunch to kids in grades 1 through 8 (ages 6 to 14 years) who attend primarily government and government-aided schools. Q.401 & Q. 402 will indicate if the child receives MDM on a daily basis on school days or not, and Q. 403 & Q. 404 will indicate whether the child consumes MDM.

Absenteeism from the school may be in many reasons, here in Q.405 – Q.407 try to get the details about the child discontinues from the school from last 15 days.

***Section 5:  Rating of health***

Refer ‘Section 5: Rating of health’ in 0 – 4 years old children questionnaire section mentioned above.

# 10 To 19 Years Old Female Questionnaire

Determine how many females of age between 10 – 19 years live in the household before beginning the interview. The number of interviews will be conducted in proportion to the number of children, with one interview per child.

***Section 1:  Respondent's Background Characteristics***

Ask the respondent’s name, age, religion & ethnic group as per detailed in the previous sections.

**Education**

The term “school” means formal schooling, which includes primary, secondary, and post-secondary school, and any other intermediate levels of schooling in the formal school system. It includes technical or vocational training beyond the primary-school level, such as long-term courses in mechanics or secretarial work. However, this definition of school does not include Bible school or Koranic school or short courses like typing or sewing.

Q.103 – Q. 107: To get the information about the educational status two important things to be considered. First one is whether the respondent is currently going to school/ collage (Q. 103), if yes enter the ‘years of education’. Record only the number of the highest standard that the respondent successfully completed. Examples: If the respondent was attending standard 9 and left school before completing that year, record ‘08.’ Although standard 9 was the highest year she attended, she completed only standard 8. If the attended only two weeks of standard 1 in primary school, record ‘00’ for completed years. If a respondent says simply that she completed primary (or secondary), you must probe to find out the exact number of standards completed. For persons with education beyond the secondary school level, you will need to find out how many years they completed beyond secondary. You will then add the number of years completed beyond the secondary level to the number 12 to calculate the response to Q. 104 & Q. 107. Note that in such cases we are using 12 years as the total number of years required to finish higher secondary. There is no need to ask respondents with post-secondary education how many standards they completed in school. Example: If a woman says she stopped after completing two years of B.A., add 2 years to 12 years of school and enter ‘14’ in the boxes.

**Occupation**

Q. 111: Examples of occupations are clerk, secretary, barber, taxi driver, nurse, teacher, lawyer, salesman, rubber tapper, fisherman, rice farmer, carpenter, etc. We are not interested in the industry that he works in (e.g., agriculture, mining, or services) or where he works (in a bank, a retail clothing store, or a government office), but we need to know what his job is. Example: If the respondent says he works in the Ministry of Planning, ask what he does there. Write the answer in the respondent’s own words. If you are not sure how to write the occupation, it is better to give more detail than less. “Not currently working” is not an acceptable response. If he is unemployed, get a description of his most recent job. If he does more than one job, write down what he does most of the time. If he is not working because he is in school, enter “Going to school/studying.”

| **Occupation Lists** | |
| --- | --- |
| **Professional (technical/ administrative/Managerial, etc.)** | |
|  | Physical Scientists |
|  | Physical Science |
|  | Architects, Engineers, Technologists And Surveyors |
|  | Engineering Technicians |
|  | Aircraft And Ships Officers |
|  | Life Scientists |
|  | Life Science Technicians |
|  | Physicians And Surgeons |
|  | Nursing And Other Medical And Health Technicians |
|  | Scientific, Medical And Technical Persons, Other |
|  | Mathematicians, Statisticians And Related Workers |
|  | Economists, And Related Workers |
|  | Accountants, Auditors And Related Workers |
|  | Social Scientists And Related Workers |
|  | Jurists |
|  | Teachers |
|  | Poets, Authors, Journalists And Related Workers |
|  | Sculptors, Painters, Photographers, And Related Creative Artists |
|  | Composers And Performing Artists |
|  | Professional Workers, N.E.C. |
|  | Elected And Legislative Officials |
|  | Administrative And Executive Officials Government And Local Bodies |
|  | Working Proprietors, Directors And Managers, Wholesale And Retail Trade |
|  | Directors And Managers, Financial Institutions |
|  | Working Proprietors, Directors And Managers Mining |
|  | Working Proprietors, Directors Managers And Related |
|  | Working Proprietors, Directors And Managers, Other Services |
|  | Administrative, Executive And Managerial Workers, N.E.C. |
| **Clerical** | |
|  | Clerical And Other Supervisors |
|  | Village Officials |
|  | Stenographers, Typist And Card And Tape Punching Operators |
|  | Book Keepers, Cashiers And Related Workers |
|  | Computing Machine Operators |
|  | Clerical And Related Workers |
|  | Transport And Communication Supervisors |
|  | Transport Conductors And Guards |
|  | Mail Distributors And Related Workers |
|  | Telephone And Telegraph Operators |
| **Sales** | |
|  | Merchants And Shopkeepers, Wholesale And Retail Trade |
|  | Manufacturers, Agents |
|  | Technical Salesmen And Commercial Travellers |
|  | Salesmen, Shop Assistants And Related Workers |
|  | Insurance, Real Estate, Securities And Business Service, |
|  | Money Lenders And Pawn Brokers |
|  | Sales Workers, N.E.C. |
| **Service worker** | |
|  | Hotel And Restaurant Keepers |
|  | House Keepers, Matron And Stewards (Domestic & Institutional) |
|  | Maids And Related House Keeping Service Workers, N.E.C. |
|  | Building Caretakers, Sweepers, Cleaners And Related Workers |
|  | Launderers, Dry-Cleaners And Pressers, N.E.C. |
|  | Hair Dresser, Barbers, Beauticians And Related Workers |
|  | Protective Service Workers |
|  | Service Workers |
| **Skilled and unskilled manual** | |
|  | Miners, Quarrymen, Well Drillers & Related Workers |
|  | Metal Processors |
|  | Wood Preparation Workers And Paper Makers |
|  | Chemical Processors And Related Workers |
|  | Spinners, Weavers, Knitters, Dyers And Related Workers |
|  | Tanners, Fellmongers And Pelt Dressers |
|  | Food And Beverage Processors |
|  | Tobacco Preparers & Tobacco Product Makers |
|  | Tailors, Dress Makers, Sewers, Upholsterers & Related Workers |
|  | Shoemakers & Leather Goods Makers |
|  | Carpenters, Cabinet & Related Wood Workers |
|  | Stone Cutters & Carvers |
|  | Blacksmiths, Tool Makers And Machine Tools Operators |
|  | Machinery Fitters, Machine Assemblers And Precession Instrument Makers (Except Electrical) |
|  | Electrical Fitters & Related Electrical & Electronic Workers |
|  | Broadcasting Station And Sound Equipment Operators And Cinema Projectionists |
|  | Plumbers, Welders, Sheet Metal & Structural Metal Preparers And Erectors |
|  | Jewellery & Precious Metal Workers And Metal Engravers |
|  | Glass Formers, Potters & Related Workers |
|  | Rubber And Plastic Product Makers Workers |
|  | Paper & Paper Board Products Makers |
|  | Painters |
|  | Stationery Engines And Related Equipment Operators, Oilers |
|  | Transport Equipment Operators (Driver) |
|  | Labourers (Labourers, N.E.C.) |
| **Agricultural** | |
|  | Farm Plantation, Dairy And Other Managers And Supervisors |
|  | Cultivators |
|  | Farmers, Other Than Cultivators |
|  | Agricultural Labourer |
|  | Plantation Labourers & Related Workers |
|  | Other Farm Workers |
|  |  |
|  | Wage Earning (Workers do work according to the wage ther are earning) |
|  | Horticulture (Horticulturists are [agriculturists](https://en.wikipedia.org/wiki/Agriculturists) who grow flowers, fruits and nuts, vegetables and herbs, as well as ornamental trees and lawns) |
|  | Shifting cultivation ( |
|  | Forest collection (Forestry Workers, Hunters And Related Workers) |
|  | Food gathering |
|  | Fishing (Fishermen And Related Workers) |
|  | Going to school/studying |
|  | Looking for work |
|  | Retired |
|  | Unable to work/ ill/ handicapped |
|  | Housework/ Childcare |

**Work all or part of the year**

Q.112: Of interest here is the regularity with which a respondent is working. If she is working all year long, record ‘THROUGHOUT THE YEAR’ even if she works only part-time. For example, if she works only during the sowing season, record ‘SEASONALLY/PART OF THE YEAR’. If she works occasionally, record ‘ONCE IN A WHILE’. If a respondent says that she works throughout the year but she takes one month leave every year, record ‘THROUGHOUT THE YEAR’. Taking vacation or leave does not make a response of THROUGHOUT THE YEAR invalid.

**Type of payment**

Q.113: This question asks for the type of payment that the respondent receives for her work. Payment can come in two forms: in “cash” and in “kind.” For example, a woman who sells fruit in the market to people who pay money for it earns cash for her work. If the woman receives a portion of the fruit she takes to the market as payment, she receives “kind” for her work. You will need to determine if the woman receives payment in cash only, in kind only, both in cash and in kind, or whether she is not paid. If a respondent is a clerk and gets paid a regular salary, she earns CASH ONLY. If she is a domestic servant and she gets food and some cash, she is paid in CASH AND KIND. If she works as a domestic servant and she is not paid a salary but instead gets lodging and food only, she is paid IN KIND ONLY. If she is working on a farm owned by the family and receives no payment of money or other goods, then she is NOT PAID.

**Kind of Toilet Use**

Q.114: Here we want to capture that what kind of toilet facility the respondent is using. Whether the person is using Own toilet or community toilet or shared toilet with other household or using open field.

**Hygiene**

Q.115: This question measures a key aspect of personal hygiene that has implications for the health of person. Here, asks the respondent to know the materials uses to wash the hands. Note whether or not water is available and whether soap/detergent or other cleansing agent like ash/mud/sand is present.

**Health Insurance**

See the Health insurance & health schemes sections in Household questionnaire mentioned above.

***Section 2:  IFA Supplementation and deworming medications***

See the IFA Supplementation and deworming medications sections in 5 – 9 years old children questionnaire section mentioned above.

***Section 3:  Mid-day Meal programme (MDM) & Absenteeism***

Refer the Mid-day Meal programme (MDM) & Absenteeism sections in 5 – 9 years old children questionnaire section mentioned above.

***Section 4:  Smoking & Alcohol Consumption***

**Smoking and tobacco use**

Q.401 – Q. 405: In Q. 401 ask the respondent whether she currently smokes cigarettes. In Q. 402 first find out the use of different form of tobacco and second record the number of cigarettes she smokes each day (frequency). In third question ask about ‘how often do you use tobacco?. In the last part of Q.402 ask ask how long the respondent has been smoking. If less than 1 month record in weeks, if less than two years record in months, and if more than two years record in years.

**Smoking and tobacco use cessation**: These questions are asked of current smokers and/or users of tobacco products as filtered by Q. 401. Ask if the respondent has tried to quit smoking and/or using tobacco in the past 12 months (Q. 403). Then ask if the respondent has visited a doctor OR other health care provider in the past 12 months (Q. 404) and if so, were they advised her to stop smoking and/or using tobacco (Q. 405).

| Cigar  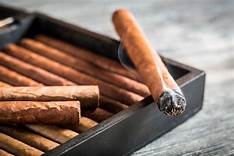 | A pipe  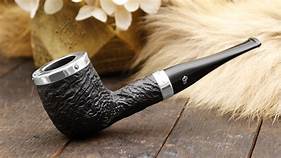 |
| --- | --- |
| Hookah  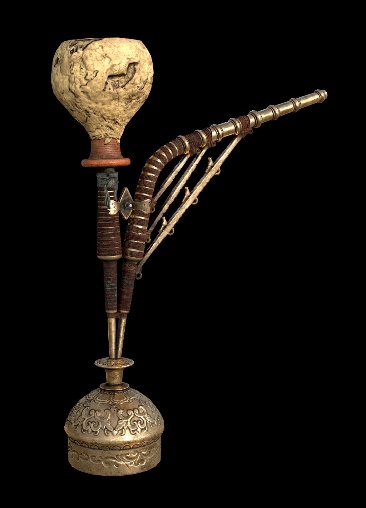 | Gutka / paan masala  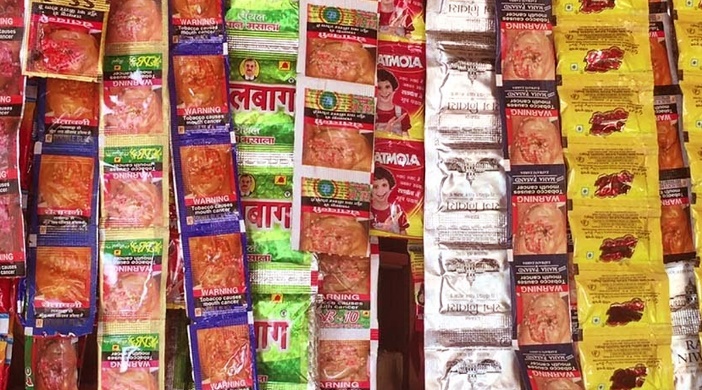 |
| Tobacco  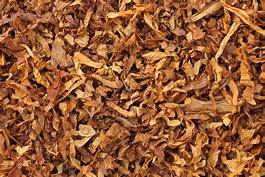 | Khaini  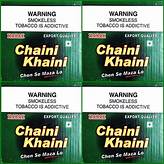 |
| Paan with tobacco  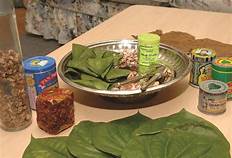 | Snuff  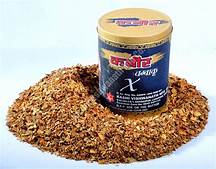 |

**Drinking Alcohol**

Q406 – Q408: In Q. 406, ask the respondent whether she currently drinks alcohol. Then in Q. 407 record the type of alcohol, then record the frequency and how often use alcohol and for how long the respondent has been consuming alcohol regularly. In last, Ask if the respondent has tried to quit drinking alcohol in the past 12 months (Q. 408).

**Tattooing & Piercing**

Q.409: in Q.409, ask the respondent that ever-done tattooing/piercing in her body. And also ask about from where did it get.

***Section 5: Marriage, Reproduction & Family Planning***

This section is only for those respondent’s age is more than 12 years.

**Current marital status**

Q. 501 is concerned with the current marital status of respondents who are 13 years or older. Thus, you should not record an answer in Q. 501 if the respondent is 12 years or younger.

‘Never married’ if the person has never been married

‘Currently married’ if the person is married irrespective of whether the spouse lives in the same household

‘Widower’ if the person is married but her spouse is dead

‘Divorced’ if the person was married, but has legally obtained a divorce from her spouse

‘Separated’ if the person is married, and his/her spouse is alive, but they are separated and do not live together as husband and wife

‘Live – In’ if the person was not married, but lives with her spouse.

In Q.502, ask whether the respondent married once or more than once.

Q. 503, we are asking if the woman was related in any way to husband before they were married. If they were related. If ‘yes’ ask Q. 504 what is the relation.

**Age at first marriage**

This question is being asked of women for whom no year of marriage was entered in Q. 505 despite your best efforts at probing. It is hoped that even if she is unable to tell you her marriage date, she will be able to give her age at the time of marriage. For a woman who has been married more than once, we want to find out her age at the time of her first marriage, not her age when she got married the second or any other time. As with other age questions, if she doesn’t know, probe.

**Month and year of first marriage**

Q. 506: With this question we want to find out the date of the respondent’s first marriage & verify this with the previous question.

**Number of times conceived**

Q.507, asks about number of times got pregnant till the date of interview. If the answer is one or more get how many months after marriage, she got conceived in Q. 508.

**Live births**

Q. 509: We want to know whether the respondent had any pregnancies that did result in a live birth.

**Still births**

Q. 510: A stillbirth is the death or loss of a baby before or during delivery. A stillbirth is loss of a baby at or after 20 weeks of pregnancy.

**Abortions**

Q. 511:

**Living Children**

Q. 512: Asks to see whether the woman has any children who are alive. record it in the boxes by number separately in sons and daughters. Once the number of sons and daughters are entered, calculate the total number of living children and enter it in the total box.

**Children Died**

Q. 513: Asks to see whether the woman has any children who are alive. record it in the boxes by number separately in sons and daughters. Once the number of sons and daughters are entered, calculate the total number of children died and enter it in the total box. This questions on children who have died are extremely important and are among the most difficult on which to obtain accurate data. Some respondents may fail to mention children who died very young, so if a woman answers NO, it is important to probe by asking, “Any baby who cried, who made any movement, sound, or effort to breathe, or who showed any other signs of life even if for a very short time?” Some respondents may be reluctant to talk about this subject and may become sad or upset that you are asking such questions. Be sympathetic and tactful in such situations. Say that you know the subject is painful but that the information is important.

**Number of births in last 5 years**

Q.514: Asks, how many births occurred in 2017 or later. You must include all births in 2017 or later, even if they later died. If the respondent did not have any births in 2017 or later, record ‘0’ and skip to Q. 515.

**Current pregnancy status**

Q.516: If the respondent does not know for certain whether or not she is pregnant, record ‘8’ (UNSURE). If she is not pregnant or if she is unsure, skip to Q. 518.

**Months of pregnancy**

Q. 517: Record the answer in completed months. You may need to check that the woman is responding in completed months. Example: If the woman answers that she is ‘five months pregnant’, ask “Are you in your 5th month of pregnancy, or have you completed your 5th month of pregnancy?” Record ‘04’ if she responds she is in the fifth month of pregnancy and ‘05’ if she has completed the fifth month.

**Contraception**

This section collects information relating to the knowledge and use of various contraceptive methods which a couple can use to avoid or delay pregnancy. Questions about the use of methods of contraception apply to all partners of respondents, whether or not the couple is currently living together or married. For example, if the respondent has been married more than once, it does not matter with which particular husband she may have used a method. The topic of contraception and family planning may be considered a personal matter by a respondent, and she may feel embarrassed to talk about it. To overcome her embarrassment, you must show that you do not feel embarrassed or uncomfortable in any way. Ask these questions as if they were no different from any other questions in the questionnaire. If she is hesitant to answer any of these questions, reassure her that everything she says will be treated confidentially and that the same questions are being asked of women all over the country.

In Q. 518 & Q.519 (knowledge), ask “Have you ever heard of ________?” Record Code ‘Yes’ if she knows the method and ‘No’ if she does not know the method. The respondent may not always understand what you are talking about when you describe a particular method. In such cases, repeat the description. If she still does not understand, you may need to explain the method in different words or in slightly greater detail.

In Q. 519 practice questions are some of the most important in the questionnaire. Depending on the method a respondent mentions, you may need to probe to determine that the method is being used currently. For example, coitus-related methods such as condoms, vaginal methods and withdrawal are used with each act of intercourse, so current users of these methods should have used them during the most recent acts of intercourse. Current users of the pill should be taking pills daily. Other methods provide ongoing protection without daily or regular action by the woman. Contraceptive injections may have been administered two to six months earlier and still provide protection. An IUD, once inserted, protects against pregnancy until it is removed or expelled. If the woman reported in Q. 321 that she had been sterilized in order to avoid having another child, you will record FEMALE STERILIZATION as the current method without asking her which method she is currently using. If the woman’s current husband has been sterilized, you will record MALE STERILIZATION as the current method. If, however, she is no longer married to the man who had a vasectomy, this should not be noted as the current method. If the woman mentions more than one method, record the code for all methods that are currently being used. If more than one method is recorded, follow the skip instruction for the highest method on the list and ask the subsequent questions about that method.

***Description of Contraceptive Methods***

In order to complete the contraceptive table accurately and completely, it is important that you have some knowledge of contraceptive methods yourself and that you are familiar with the names that people use to refer to each method. The following provides additional information on selected methods that are included in the contraceptive table that may be useful in completing the table:

| Female sterilization | There are several types of operations a woman can have that will make her sterile, including a “tube tie” (tubal ligation). Only when the operation was performed specifically to enable the woman to stop having children should you record it as a sterilization. The removal of the uterus (i.e., a hysterectomy) or ovaries will also make the woman sterile, but that is not a contraceptive method. Operations to remove the womb or uterus may be performed for reasons other than to provide contraceptive protection, e.g., because the woman experienced a problem during delivery, the woman had recurrent spells of heavy bleeding, or cancer was found. |
| --- | --- |
| Male sterilization | This is a comparatively minor operation done on men for contraceptive purposes. It is also called vasectomy. In recent years, the “no scalpel” vasectomy has become more common. |
| IUD or PPIUD | A hormonal or Copper T (such as Multilode) or a Lippes Loop are common types of IUDs available in India. An IUD (intrauterine device) is a coil or a T-shaped device that is inserted in the uterus by a doctor or a nurse to prevent pregnancy. The IUD prevents a fertilized egg from being implanted in the uterus wall. A PPIUD (postpartum intrauterine device), is an IUD that is inserted postpartum (that is, after giving birth). An IUD is a reversible form of contraception that can be used for up to 3-10 years (depending on the type) before needing to be replaced. In some states, the IUD is known as 'Tambi'.  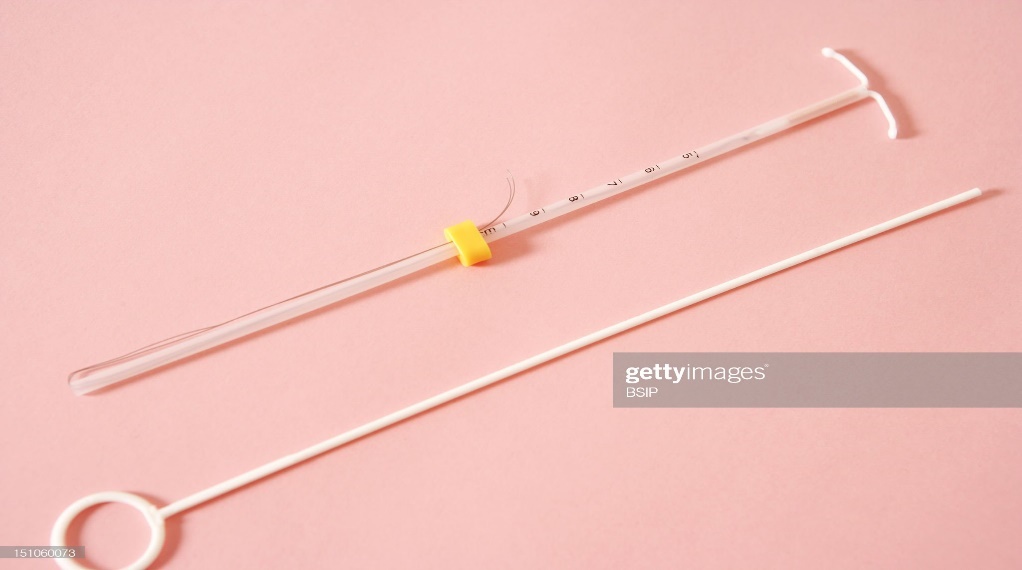 |
| Injectables | An injection of hormone that is released slowly into the bloodstream can be given regularly to women by a health provider to prevent pregnancy. The most common type of injectable contraceptive is given every three months. This is known as depomedroxyprogesterone acetate (DMPA), Depo Provera, Depo, or Megestron ® . Another injectable contraceptive, NET EN (also called Noristerat ® ) is given every two months |
| Pill | This is a pill the woman takes every day or once a week for one type of pill. The pill taken every day is a combination of oestrogen and progesterone (hormones) which prevents the ovary from releasing an egg. These pills are also known as 'Mala D' and 'Mala N' in India. The pill taken once a week is a non-hormonal pill known as “Saheli.”  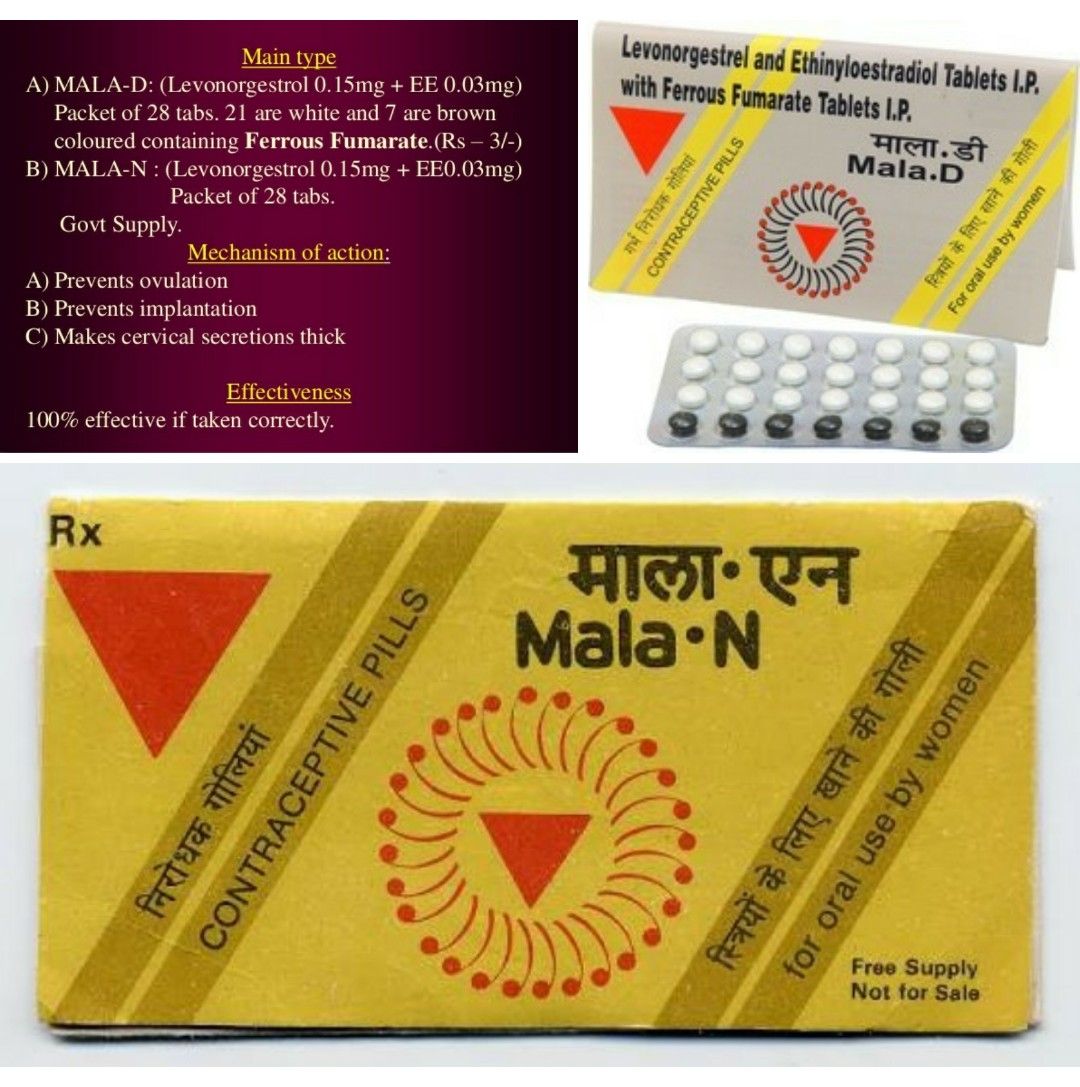 |
| Condom or Nirodh | Men can put a thin, rubber sheath on their penis before sexual intercourse. |
| Female condom | A thin, transparent rubber can be placed in the vagina before sex to avoid pregnancy. |
| Emergency contraception | Women can take pills up to three days after having sex to avoid getting pregnant. These pills are also called “morning-after pills.” |
| Diaphragm | A diaphragm is a shallow cup shaped like a little saucer that's made of soft silicone. The woman bends it in half and inserts it inside her vagina to cover her cervix. A diaphragm must be used with spermicide (a cream or gel that kills sperm) for full effectiveness. |
| Foam/Jelly | A woman can put a spermicidal foam or jelly in her vagina before sex to prevent pregnancy. The foam or jelly has chemicals that stop sperm from reaching an egg. |
| Standard days method | Women can use color-coded beads to track the days of their menstrual cycle when they are most likely to get pregnant, and the couple avoids unprotected sex on those days. These are sometimes referred to as CycleBeads. |
| Lactational Amenorrhea Method (LAM). | Women can postpone the return of menstruation after a birth (and therefore remain unlikely to become pregnant) by breastfeeding frequently. LAM is a specially taught method that makes use of this principle. This method requires a woman to breastfeed frequently (without feeding the child anything else except very limited amounts of plain water) and to know that the method can be used for up to six months after a birth as long as menstruation has not returned. The method also teaches women that if menstruation returns, the child becomes six months old, or the mother starts feeding her child anything other than breastmilk or plain water, they should begin using another method of contraception if they want to avoid becoming pregnant. |
| Rhythm method | This is also called the safe period, periodic abstinence, or the calendar method. This method is based on the principle that by not having sexual relations on certain days of her monthly cycle, a woman can avoid becoming pregnant. Note that this is not the same as prolonged abstinence where the couple stops having sexual relations for months at a time to avoid pregnancy without regard to the woman’s monthly cycle. To ensure that the respondent understands, stress the phrase “on the days of the month she is most likely to get pregnant.” Also, if a woman does not feel like having sex on particular days of her cycle, that does not mean that she is using the rhythm method. |
| Withdrawal | Men can be careful and pull out before climax. |
| Any other method(s) | Women may mention methods that are not described in the table. These may include modern methods such as a sponge, a contraceptive implant, or a cervical cap or traditional or folk methods such as prolonged abstinence or herbs. |

Q. 520 are asking to get the details about whether she ever told by a health worker about any method of family planning that you can use to avoid pregnancy. In this question, a current user is asked whether she was told by a health or family planning provider about other methods of family planning either at the time she obtained her current method or at some other time.

Below image is representing the different available family planning method to avoid pregnancy in the health - care facility.


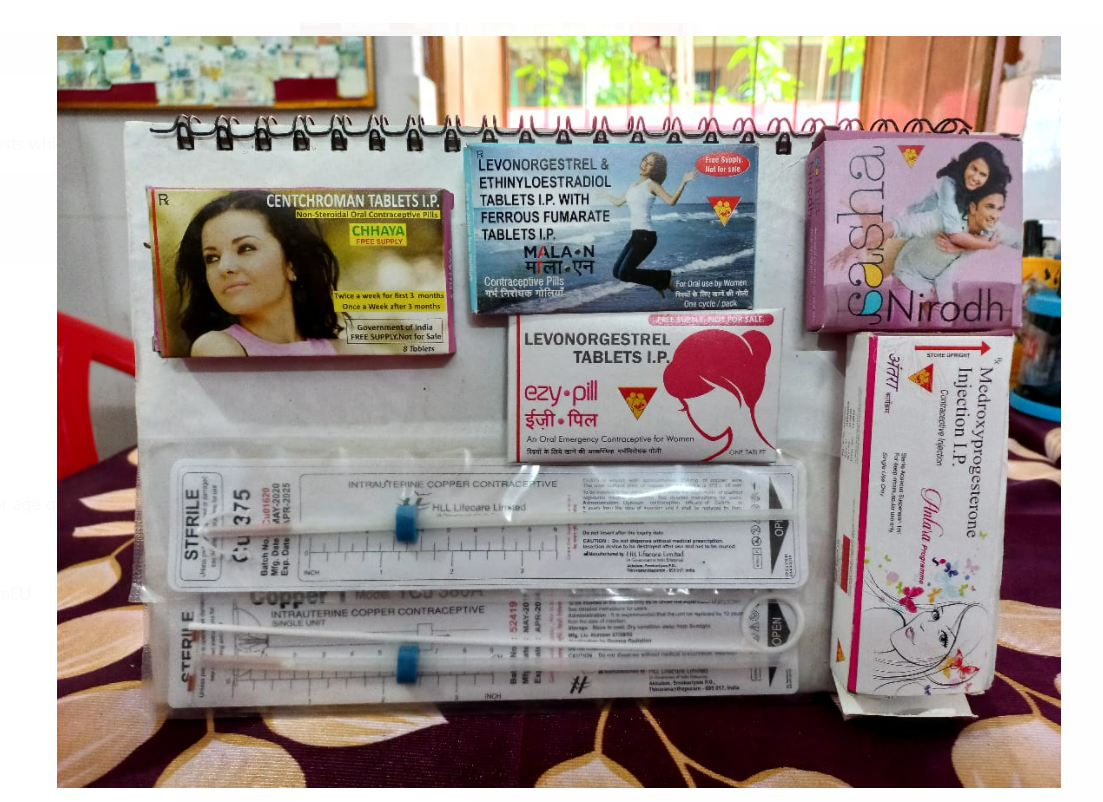


In Q. 521 t information a current user has received about the side effects or problems associated with her current method. Here asks whether the user was told about potential side effects or problems at the time she obtained her current method. If there has been more than one episode of use of the method, make sure that the respondent knows that you are asking about the time that she started using the method during the current episode of use.

**Desired timing of pregnancy**

If respondent answers YES for Q. 522, go to Q. 523. Otherwise, proceed to Q. 601.

***Section 6: Knowledge of HIV/ AIDS***

AIDS is an illness caused by HIV, a virus that weakens the immune system and leads to death through secondary infections, such as pneumonia. It is transmitted through sex or through contact with contaminated blood. This section asks questions concerning knowledge and behaviour related to HIV/AIDS and other diseases that are transmitted through sexual contact.

**Heard of AIDS and HIV**

Q. 601: This question allows us to verify whether a respondent has heard of HIV or AIDS. If there is a local term for HIV or AIDS, use the local term in addition to the word ‘HIV’ or ’AIDS’.

In Q. 601, checks about the transmission, prevention & treatment knowledge of the respondent.

***Transmission, prevention & Treatment knowledge questions***

| Transmission knowledge questions | | | |
| --- | --- | --- | --- |
|  | HIV is the virus that can lead to AIDS.  Can people reduce their chance of getting HIV by having just one uninfected sex partner who has no other sex partners? | | Yes……………………1  No…………………….2  Don’t know…………...8 |
|  | Can people get HIV from mosquito bites? | | Yes……………………1  No…………………….2  Don’t know…………...8 |
|  | Can people get HIV from blood products or blood transfusions? | | Yes…………………..1  No……………………2  Don’t know…………..8 |
|  | Can people get HIV by injecting drugs? | | Yes…………………..1  No……………………2  Don’t know…………..8 |
|  | Can people get HIV by sharing food with a person who has HIV? | | Yes…………………..1  No……………………2  Don’t know…………..8 |
|  | Can HIV be transmitted from a mother to her baby? | |  |
|  | During pregnancy? | | Yes…………………..1  No……………………2  Don’t know…………..8 |
|  | During delivery? | | Yes…………………..1  No……………………2  Don’t know…………..8 |
|  | By breastfeeding? | | Yes…………………..1  No……………………2  Don’t know…………..8 |
| Prevention Knowledge Questions | | | |
|  | Can people reduce their chances of getting HIV by using a condom every time they have sex? | | Yes……………………1  No…………………….2  Don’t know…………...8 |
|  | Is there anything else a person can do to avoid or reduce the chances of getting HIV/AIDS? | | Yes……………………1  No…………………….2  Don’t know…………...8 |
|  | What can a person do? Anything else?   \|  \| YES \| NO \| \| --- \| --- \| --- \| \| Abstain from sex \| 1 \| 2 \| \| Use condoms \| 1 \| 2 \| \| Limit sex to one partner \| 1 \| 2 \| \| Limit number of sexual partners \| 1 \| 2 \| \| Avoid sex with sex workers \| 1 \| 2 \| \| Avoid sex with persons who have many \| 1 \| 2 \| \| partners \| 1 \| 2 \| \| Avoid sex with homosexuals \| 1 \| 2 \| \| Avoid sex with persons who inject \| 1 \| 2 \| \| drugs \| 1 \| 2 \| \| Avoid blood transfusions \| 1 \| 2 \| \| Use blood only from relatives \| 1 \| 2 \| \| Avoid injections \| 1 \| 2 \| \| Use only new/ sterilized needles \| 1 \| 2 \| \| Avoid IV Drip \| 1 \| 2 \| \| Avoid sharp razors/blades \| 1 \| 2 \| \| Avoid kissing \| 1 \| 2 \| \| Avoid mosquito bites \| 1 \| 2 \| \| Others  (Spacify____________) \| 1 \| 2 \| \| Don’t know \|  \|  \| | | |
| Treatment Knowledge | | | |
|  | Are there any special drugs that a doctor or a nurse can give to a woman infected with HIV to reduce the risk of transmission to the baby? | Yes…………………..1  No……………………2  Don’t know…………..8 | |
|  | Have you heard about special antiretroviral drugs (USE LOCAL NAME(S)) that people infected with HIV/AIDS can get from a doctor  or a nurse to help them live longer? | Yes…………………..1  No……………………2  Don’t know…………..8 | |

***Section 7: Women Empowerment, Menstrual Hygiene & Gender based Violence***

**Woman’s participation in household decisions**

Q.701: asks about the three major decisions taken by herself or not. These address the roles of the woman and her husband in making various household decisions. Decision-making can be a complex process so the emphasis is again on who usually makes a specific decision. Choose the response most appropriate after you hear the respondent’s answer to each type of decision.

**Ownership of home(s) or land**

Q. 702: There is increasing evidence that ownership of property by women has positive consequences for women’s empowerment, nutritional and health outcomes, and children’sschooling. Forthese questions, ‘ownership’ implies that the house or land is legally registered in the woman’s name or, since official property records do not always exist or are not maintained, the house or land is recognized as hers and cannot be sold without her signature or equivalent.

Q. 702 is concerned with whether the respondent owns a house either by herself or jointly with someone else. For this question, ‘house’ includes all dwelling types including apartments, duplexes, and houses that are semi-detached or detached, etc., as well as other types of dwellings that are specific to India. If she owns a house (either the one you are in at the time of the interview or any other house), and she is the only owner of the house (she does not share ownership with anyone), record ALONE ONLY. If she doesn’t own a house on her own, but instead jointly owns one with someone else, record JOINTLY ONLY. If she owns a house alone and another house jointly with someone else, record BOTH ALONE AND JOINTLY. If she does not own a house either alone or jointly, record DOES NOT OWN. Note: It does not matter if the house was bought with a loan and the loan is still being paid for; what matters is whether the respondent’s name is on the ownership document or, in the case where there is no paperwork, the respondent believes she has exclusive or part ownership of the house, record as outlined above. For land, the size, quality, or purpose of the land is not relevant to this question; we are only asking about her ownership of any type or size of land.

Note: communally owned land is not owned by her, even if she is part of the community.

**Bank account**

Q. 703: The account can be joint or sole and it can be in any bank or other type of savings institution. We are interested in whether the respondent has a bank account that she herself uses.

**Mobile phone**

Q.704: Availability and use of mobile phones are very important. In these questions, we would like to know whether respondent has a mobile phone that she herself uses. If she does, then Q. 705 asks whether she herself uses her mobile phone for financial transactions. It does not matter with whom or through whom the transaction is made, whether through a bank or through a mobile money transfer system. Financial transactions include paying bills, paying back loans, depositing money, transferring money to another person, and receiving salary or payments.

**Internet use**

Q. 706: asks the respondent if she has ever used the internet. Even if she has used the internet only one time, record ‘1’ for YES. Internet use includes accessing web pages, e-mail, instant messaging, applications (such as WhatsApp), and social media (such as Facebook, Instagram, and Twitter). Internet access can be via a fixed or mobile network, and can occur via desktop, laptop, and tablet computers, mobile phones, and other devices such as e-readers, smart televisions, and game machines. It does not matter if the internet use takes place in the household the respondent is living or elsewhere.

**Health Insurance**

Q.707 & Q. 708: See the Health insurance & health schemes sections in Household questionnaire mentioned above.

**Menstrual Hygiene**

Q. 709 – Q. 716 were asking to get the details about the menstrual hygiene.

Q. 709 asks to determine if the woman ever menstruated. If she has never menstruated, skip to section 8.

Q.710: Age of first menstruation - Ask the woman how old she was when she had her first monthly period. Record the age in completed years.

Q.711: Ask the girl what she uses for menstrual protection, if anything. Record all responses.

Q. 712 & Q.713: Ask the girl how she washes the sanity cloths if she uses them & how she disposes of the sanitary pad

Q. 714 & Q. 715 & Q. 716: Ask to determine if there are any restrictions for any places or any activities for her at the time of menstruation as compared to the normal days.

**Gender based violence**

From Q. 718 – 720 We are interested in knowing whether the respondent has been physically hurt as a result of some deliberate act by anyone. If she experienced any type of violence from anyone, ask Q.719 from whom and then ask in Q.720 what type of violence she experienced is it ‘Sexual or Physical or both.

Q. 721, asks only for those conceived at least one time to know that she experienced any type of violence from her husband during the pregnancy time.

***Section 8: Maternal and Child Health (Mothers - for last birth in the 5 years before the survey)***

**Pregnancy Registration**

Q. 801 – 804: If the respondent’s pregnancy was registered, record ‘yes’. If the pregnancy was not registered, skip to Q. 807. For these questions, we are interested in registration with any other health professional or authority. In Q. 802 record the exact month in which the pregnancy was registered. For example: If the woman reported that she was registered in the second month of her pregnancy then record 2. If the respondent mentioned that her pregnancy was registered in the fifth month of her pregnancy then record 5 For those women whose pregnancy was registered, ask with whom they registered their pregnancy (Q. 803) and if she received a Mother and Child Protection (MCP) Card after registration (Q. 804).

**Months pregnant at first antenatal visit**

Q. 805 – Q. 806: Ask the respondent how many months into her pregnancy she was when she received her first antenatal care. If she does not remember, ask her how many periods she had missed at the time. Assume each missed period corresponds to a month and enter the number in the space provided. For example, if the respondent doesn’t recall how many months pregnant she was when she first received antenatal care, but knows that she had missed three periods, record ‘03’. If the respondent says she first received antenatal care for the pregnancy in the second month of her pregnancy, then record 2.

**Tetanus Toxoid Injections**

Q. 807 – Q. 811: Neonatal tetanus is a disease that kills many babies. Another name for tetanus is lockjaw. If a local term exists for tetanus, it may be used in explaining the disease to the respondent. If a woman receives at least two tetanus vaccinations during any pregnancy, she is considered to be adequately immunized to protect her baby against neonatal tetanus. A child also is considered to be adequately protected if the mother received at least five injections with tetanus vaccine during her lifetime and the last booster was received within a ten-year period prior to the pregnancy.

Qs. 807 – Q. 808 ask about whether the respondent received any tetanus injections during that pregnancy and, if so, how many times she was given the tetanus injection. The tetanus vaccine is usually given to the pregnant woman as an injection in the arm or the shoulder but can also be sometimes in her buttock.

Q. 806 A respondent who does not report receiving at least two injections with tetanus vaccine during the pregnancy must be asked several additional questions to assess whether she was adequately immunized at the time of her pregnancy. For those who received 2 or more tetanus injections during the pregnancy, skip to Q. 112.

Qs. 809-810 inquire about whether she received any tetanus injections prior to the pregnancy (e.g., during an earlier pregnancy or during childhood) and, if so, the total number of tetanus injections she was given before the pregnancy. Q. 811 is asked to know how many years ago the most recent tetanus injection was received before the pregnancy. For a woman who received a single tetanus injection during the pregnancy of her last birth, we are asking about the total number of injections she received before this pregnancy and the most recent tetanus injection that she received prior to the pregnancy of her last birth.

*Example: Gauri was interviewed in July, 2022, . She has two children, Sevati and Manoj. Manoj is her last birth. She says that she had one tetanus injection when she was pregnant with Manoj and two injections when she was pregnant with Sevati who was born in September 2017. She also is sure that she had all of the required childhood immunizations before entering school although she is not sure how many tetanus injections she had. For Gauri, you should record YES in Q. 807, record ‘1’ in Q. 808.. You should record YES in Q. 809 since she had tetanus injections prior to the pregnancy. Since Gauri is sure she had all required immunizations before entering school, you may assume that she had three immunizations during early childhood. If we include the two injections when she was pregnant with Sevati, this means she had a total of five tetanus injections before she became pregnant with Manoj. Thus, you should record ‘5’ in Q. 810. Priorto her pregnancy with Manoj, her most recent tetanus injection wasin 2017, the year of Sevati’s birth. Thus, in Q. 811, record ‘03’ since the tetanus injection was given three years ago.*

**Iron tablets/syrup**

Q. 812 -Q. 813: Anaemia is a common problem during pregnancy that can be overcome by additional intake of iron. Q. 812 asks whether the woman was given or bought any iron folic acid tablets or syrup during her pregnancy. Since some women may not know that they were given iron tablets, show the woman the sample tablets as you ask this question. Note that we are not asking whether or not she consumed the tablets/syrup she was given or bought; rather, we want to know whether she had the tablets/syrup in her possession during the pregnancy. We also are asking if she was given or bought the tablets, not if she already had them at home, so record NO in that instance and skip to Q. 814. If the respondent was given or bought iron tablets/syrup (YES in Q. 812), ask her for how many days during her pregnancy she took the tablets/syrup. Record the response in the boxes in Q. 813. Remember to put a leading zero in front; 30 days would be ‘030’. If she was given or bought iron tablets but never took even one, record ‘000’. If she does not remember, probe for the approximate number of days, e.g., by asking how many months pregnant she was when she began taking the tablets and whether she took the tablets every day after that.

**Place of birth**

Q. 814: The intent of this question is to identify births delivered in a health facility. If the woman gave birth in a health facility, ask whether the place is in the public (run by the government) or private health sector. Write the name of the facility in the space provided if the respondent does not know whether the place is run by the government or is private. Inform your supervisor about the problem. The supervisor will try to identify the type of facility.

**Caesarean section**

A caesarean section or C-section is a delivery of a baby through an incision in the woman’s abdomen and womb, rather than through the birth canal. Such a delivery is necessary for some women due to pregnancy complications. Caesarean sections are also sometimes elective, either for the convenience of the mother or the health care workers performing the surgery. Also, because there can be complications associated with having a vaginal birth following a caesarean, women who have delivered one child via a caesarean are more likely to deliver subsequent children by caesarean. Find out whether the baby was delivered by an operation (Q. 815) and if so, whether the decision to have a caesarean section was made before or after her labour pains started (Q. 816).

**Postpartum check for mothers in the health facility**

Getting a postpartum check soon after the baby is born is crucial for the health of the mother. We are interested in knowing whether the respondent saw anyone for a postpartum check and, if so, who performed the check and how many hours, days, or weeks after the birth the first check took place. This information is gathered separately for the mother and child based on the place of delivery – institutional delivery or home delivery.

Q. 817 – Q. 819: In this set of questions, we are asking only about a health check for mothers who had an institutional delivery. If someone checked on the health of the mother.

Q. 820 is directed to women who delivered in a health facility and inquires whether anyone checked on the woman’s health before she was discharged.

**Cost of delivery**

In Q. 821, ask how much money was spent for delivery, and ask for the total amount of money paid for this delivery.

**Postpartum checkup for mothers who delivered in a place other than a health facility**

Q. 822 is asked of women who delivered outside a health facility. This question is how soon that first check occurred. Q. 823 asks who conducted the first postpartum check after birth.

***Section 9- Chronic Disease Conditions***

**Chronic Disease Conditions**

Chronic diseases are defined broadly as conditions that last 1 year or more and require ongoing medical attention or limit activities of daily living or both. Chronic diseases such as [heart disease](https://www.cdc.gov/chronicdisease/resources/publications/factsheets/heart-disease-stroke.htm), [cancer](https://www.cdc.gov/chronicdisease/resources/publications/factsheets/cancer.htm), and [diabetes](https://www.cdc.gov/chronicdisease/resources/publications/factsheets/diabetes-prediabetes.htm) are the leading causes of death and disability.

Q.901 is asking about whether the respondent ever diagnosed with any of the chronic disease conditions by any of the health provider or not. If ‘yes’ then ask about ever sought the treatment for the diagnosed disease condition/s. Here ask each disease condition one by one.

If the responder indicated in Q. 901 that she have "Cancer," ask Q. 902 for further information about the specific type of cancer she have, and if "Sickle Cell Disorder," ask Q. 903 for more information on that condition.

***Section- 10: Health care seeking behavior***

**Visits to Health Facility**

This section is mainly focusing on the health care-seeking behaviour of the respondent.

Q.1001, asks about visit of any health facility for treatment of any disease or injury during past 12 months by the respondent. If ‘yes’, ask Q. 1002, from where the respondent taken the treatment? In Q. 1003 & Q. 1004 enter the information on for which chronic condition the treatment was taken. And whether the condition is cured or not asking in Q. 1005. If Q. 1001 is ‘no’ skip to section 11.

***Section 11: Symptomatic Profile***

**Tested Blood Pressure, Anemia & Blood Sugar**

Q. 1101 – Q. 1104, asking about to get the detail about the blood pressure if the respondent is suffering from high or low blood pressure. Ask Q. 1101, whether the person ever been checked blood pressure prior to this survey. If ‘yes’ then as, time when last measurement was taken, what was the result, and taking any medicine for blood pressure in Q. 1102 – 1104.

Q. 1105 – Q. 1106: Information regarding the anemia. Q. 1105 asks any time before the survey tested for anemia, , If found “positive” what advice was given by the health provider in Q. 1106?

Q. 1107 – 1110: Information regarding the blood sugar. Asking about, respondent checked the blood sugar prior to the survey (Q. 1107), if ‘yes’, when was the test done? (Q. 1108) what was the test result? (Q. 1109), if the result is ‘high’ what was the advice given by the health provider? (Q.1110)

**Symptomatic profile**

In Q. 1112, it is questioned whether the respondent experienced any of the listed acute symptoms in the month just before the survey. To acquire an accurate response from the respondent, ask about each of the acute symptoms listed one at a time.

***Section 12: Rating of Health***

Refer ‘Section 5: Rating of health’ in 0 – 4 years old children questionnaire mentioned above.

# 10 To 19 Years Old Male Questionnaire

Determine how many male of age between 10 – 19 years live in the household before beginning the interview. The number of interviews will be conducted in proportion to the number of males, with one interview per male.

***Section 1:  Respondent's Background Characteristics***

Refer the section 1: Respondent’s background characteristics in 10 – 19 years old female questionnaire section mentioned above.

***Section 2:  IFA Supplementation and deworming medications***

See the Section 2: IFA Supplementation and deworming medications in 5 – 9 years old children questionnaire section mentioned above.

***Section 3:  Mid-day Meal programme (MDM) & Absenteeism***

Refer the Section 3: Mid-day Meal programme (MDM) & Absenteeism in 5 – 9 years old children questionnaire section mentioned above.

***Section 4: Tobacco & Alcohol Consumption***

Refer the Section: Tobacco & Alcohol Consumption in 10 – 19 years old female questionnaire mentioned above. Additionally ask Q. 410 & Q.411 regarding the shaving/haircut.

***Section 5: Marriage & Family Planning***

Refer ‘Section 5: Marriage, Reproduction & Family Planning’ in 10 – 19 years old female questionnaire mentioned above.

For Q.501 – Q. 503: Refer ‘Current marital status’ subsection

For Q. 504: Refer ‘Age at first marriage’ subsection

For Q. 505 – Q. 507: Refer ‘Contraception’ subsection

***Section 6: Knowledge of HIV/AIDS among Adults***

Refer ‘Section 6: Knowledge of HIV/ AIDS’ in 10 – 19 years old female questionnaire mentioned above.

***Section 7: Chronic Disease Conditions***

Refer ‘Section 9: Chronic Disease Conditions’ in 10 – 19 years old female questionnaire mentioned above.

***Section 8: Health care seeking behavior***

Refer ‘Section 10: Health care seeking behavior’ in 10 – 19 years old female questionnaire mentioned above.

***Section 9: Symptomatic Profile***

Refer ‘Section 11: Health care seeking behavior’ in 10 – 19 years old female questionnaire mentioned above.

***Section 10: Rating of health***

Refer ‘Section 5: Rating of health’ in 0 – 4 years old children questionnaire mentioned above.

# 20-59 Years Old Men Questionnaire

Determine how many male of age between 20 - 59 years live in the household before beginning the interview. The number of interviews will be conducted in proportion to the number of males, with one interview per male.

***Section 1:  Respondent's Background Characteristics***

Refer the section 1: Respondent’s background characteristics in 10 – 19 years old female questionnaire section mentioned above.

***Section 2: Marriage, Family Planning & Reproduction***

Refer ‘Section 5: Marriage, Reproduction & Family Planning’ in 10 – 19 years old female questionnaire mentioned above.

For Q.501 – Q. 503: Refer ‘Current marital status’ subsection

For Q. 504: Refer ‘Age at first marriage’ subsection

For Q. 505 – Q. 507: Refer ‘Contraception’ subsection

***Section 3: Tobacco & Alcohol Consumption***

Refer the Section 4: Tobacco & Alcohol Consumption in 10 – 19 years old female questionnaire mentioned above. Additionally ask Q. 410 & Q.411 regarding the shaving/haircut.

***Section 4: Knowledge of HIV/AIDS among Adults***

Refer ‘Section 6: Knowledge of HIV/ AIDS’ in 10 – 19 years old female questionnaire mentioned above.

***Section 5: Chronic Disease Conditions***

Refer ‘Section 9: Chronic Disease Conditions’ in 10 – 19 years old female questionnaire mentioned above.

***Section 6: Health care seeking behavior***

Refer ‘Section 10: Health care seeking behavior’ in 10 – 19 years old female questionnaire mentioned above.

***Section 7: Symptomatic Profile***

Refer ‘Section 11: Health care seeking behavior’ in 10 – 19 years old female questionnaire mentioned above.

***Section 8: Rating of health***

Refer ‘Section 5: Rating of health’ in 0 – 4 years old children questionnaire mentioned above.

# 20-59 Years Old Women Questionnaire

Determine how many women of age between 20 - 59 years live in the household before beginning the interview. The number of interviews will be conducted in proportion to the number of women, with one interview per women.

***Section 1:  Respondent's Background Characteristics***

Refer the section 1: Respondent’s background characteristics in 10 – 19 years old female questionnaire section mentioned above.

***Section 2: Marriage & Reproduction***

Refer ‘Section 5: Marriage, Reproduction & Family Planning’ in 10 – 19 years old female questionnaire mentioned above.

***Section 3: Family Planning (Currently married women 15-49 years) & Unmet Need for Family Planning***

Refer the subsection titled “Contraception” in ‘Section 5: Marriage, Reproduction & Family Planning’ in 10 – 19 years old female questionnaire mentioned above.

***Section 4: Knowledge of HIV/AIDS among Adults***

Refer ‘Section 6: Knowledge of HIV/ AIDS’ in 10 – 19 years old female questionnaire mentioned above.

***Section 5: Women's Empowerment, Menstrual Hygiene & Gender based Violence***

Refer ‘Section 7: Women's Empowerment, Menstrual Hygiene & Gender based Violence’ in 10 – 19 years old female questionnaire mentioned above.

***Section 6: Tobacco Use and Alcohol Consumption among Adults (age 15 years and above)***

Refer the Section 4: Tobacco & Alcohol Consumption in 10 – 19 years old female questionnaire mentioned above.

***Section 7: Maternal and Child Health (Mothers - for last birth in the 5 years before the survey)***

***Maternity Care***

Refer ‘Section 8: Maternal and Child Health (Mothers - for last birth in the 5 years before the survey)

Maternity Care’ in 10 – 19 years old female questionnaire mentioned above.

***Section 8: Chronic Disease Conditions***

Refer ‘Section 9: Chronic Disease Conditions’ in 10 – 19 years old female questionnaire mentioned above.

***Section 9: Health care seeking behavior***

Refer ‘Section 10: Health care seeking behavior’ in 10 – 19 years old female questionnaire mentioned above.

***Section 10: Symptomatic Profile***

Refer ‘Section 11: Health care seeking behavior’ in 10 – 19 years old female questionnaire mentioned above.

***Section 11: Rating of health***

# 60+ Years Old Questionnaire

Determine how many members of age between 60 & above years are live in the household before beginning the interview. The number of interviews will be conducted in proportion to the number of members, with one interview per member.

***Section 1:  Respondent's Background Characteristics***

Refer the section 1: Respondent’s background characteristics in 10 – 19 years old female questionnaire section mentioned above.

***Section 2: Tobacco Use and Alcohol Consumption***

Refer the Section 4: Tobacco & Alcohol Consumption in 10 – 19 years old female questionnaire mentioned above.

***Section 3: Chronic Disease Conditions***

Refer ‘Section 9: Chronic Disease Conditions’ in 10 – 19 years old female questionnaire mentioned above.

***Section 4: Functional Limitations***

**Physical and mental impairments**

The act of acquiring and preserving biological abilities that enable wellbeing as we age is known as "healthy ageing" (WHO, 2015). Functional capacity is the capacity of an individual to carry out activities necessary for survival in an independent and autonomous way (Freitas et al., 2012). With age, the functional capacity of humans decreases due to the dynamic and progressive ageing process. Data on level of functioning and disability are credible, dependable, and comparable using functional health measurement. Functioning and disability are multifaceted notions that relate to human body structures and functions, as well as their impairments; people's activities, and the constraints they face in those activities; people's participation in all aspects of life and the limitations on that participation.

Impairment is a loss or abnormality of psychological, physiological, or anatomical structure or function. The respondents were asked if they had any form of impairments, including physical (such as locomotor); hearing; visual; speech (such as speech production, language comprehension); and mental (such as intellectual, cognition, or learning) impairments.

Q. 401 – Q. 403: Question 401 seeks information regarding the respondent's functional limitations, if any. List the kind(s) of impairment(s) in Q. 402. Continuing with Q. 403, the respondent is asked if anyone can assist with the issues raised in question 402.

**Activities of daily living (ADL)**

Activities of Daily Living (ADL) is a term used to refer to the normal daily self-care activities, such as movement in bed, changing position from sitting to standing, feeding, bathing, dressing, grooming, and personal hygiene. These activities are fundamental for an independent life. The ability or inability to perform ADLs is used to measure a person’s functional status, especially among individuals with disabilities and the elderly. Elderly who retain their ability to perform ADL have a higher level of independence and overall well-being.

Q. 404: To assess ADL limitations, respondents were asked if they were having any of the following limitations and expected the limitation to last for more than three months: difficulty with dressing, walking across the room, bathing, eating, getting in or out of bed, or using the toilet (including getting up and down). In this section, the percentage of older adults with anyone and any two ADL limitations are presented.

**Use of AIDS or Supportive Devices**

Use of assistive technology (AT) or supportive devices is defined as “the application of organized knowledge and skills, procedures and systems related to provision of assistive products, whose primary purpose is to maintain or improve an individual’s functioning and independence, facilitate participation, and enhance overall well-being and quality of life” (WHO, 2014b). Use of AT or supportive devices provides significant support and benefits for older adults in various ways: safety and prevention (i.e., prevention of falls), mobility and independence, social connectivity and ease of living, preservation of cognitive abilities, delays in depression and functional loss and improved well-being and quality of life.

Q. 405 – Q. 406; survey participants were asked about the use of any aid or supportive device to assist them in their ADL. Any aid or supportive devices includes hearing aid, spectacles/contact lenses, dentures, and any aid for physical disabilities, such as walker/walking sticks, wheelchairs, adjustable shower tools/ commodes, back/neck collar, any orthosis or prosthesis, and any other aid.

***Section 5: Health Care Access & Utilization***

**Health Insurance**

For information on questions 501 and 503, see the section on households titled "Health Insurance. Ask Q. 502 for the respondent's explanation for not having insurance if they are not covered by any health insurance plans. In Q. 504, ask about what are the packages included in the insurance scheme?

**Healthcare utilization in the past 12 months**

Q. 505 – Q.508: A respondent may have visited one or more medical facilities or providers for treatment in response to Q. 505 and Q. 506. If the responder claims to have visited a medical institution or healthcare practitioner, ask Q. 507 to get the primary justification for the visit(s), otherwise ask Q. 508 to learn the justification for not visiting.

**Hospitalization in the past 12 months**

Q.509 – Q. 524: These questions pertain to hospitalization (inpatient care) that you have had during the past 12 months.

Q. 509 asks number of times the respondent hospitalized during last 12 months, if the answer is ‘0’ times then skip the entire questions in the section and move to section 6 otherwise ask the details about the hospitalization from the next questions. Q. 510 & Q. 511 ask to get the information about how many nights and how many months the respondent stayed in hospital during last 12 months and in Q.512 onwards collect the information on last hospitalization.

Ask specific questions about the facility the respondent visited (Q. 512), the number of nights spent there (Q. 513), the cause of the hospitalization (Q. 514 & Q. 515), the type of services received (Q. 516), the total cost of the hospitalization (Q. 517), and the respondent's health at the time of discharge (Q. 518). If the respondent states in response to question 517 that they "did not recover from illness, wished to leave without doctor's clearance," then you should ask question 518 to find out why.

To learn the present state of the illness the responder was dealing with, questions 519 through 522 were asked. Additionally, in questions 523 and 524, it was attempted to learn whether the responder would be willing to visit the same medical center in the future.

***Section 6: Quality of life***

**EQ – 5D – 5L Questions**

EQ - 5D - 5L is a standardized measure of health status developed by the EuroQol Group in order to provide a simple, generic measure of health for clinical and economic appraisal.

Q. 601 – Q. 605: comprises the 5 dimensions (mobility, self-care activities, pain/discomfort, anxiety/depression). However, each dimension has 5 levels: no problems, slight problems, moderate problems, severe problems, and extreme problems. The respondent is asked to indicate his/her health state by selecting the most appropriate statement in each of the 5 dimensions.

**
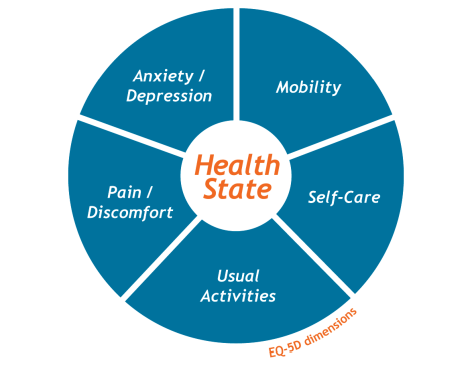
**

***Section 7: Symptomatic Profile***

Refer ‘Section 11: Health care seeking behavior’ in 10 – 19 years old female questionnaire mentioned above.

***Section 8: Rating of health***

Refer ‘Section 5: Rating of health’ in 0 – 4 years old children questionnaire mentioned above.

# Field Investigation Table

The field investigation table questionnaire includes inquiries regarding the collection of biomarkers. weight, height, waist, and hip circumference, random blood glucose hemoglobin, will all be measured at each household member. Adolescents and adults rather than children will be used to test blood pressure and isometric hand grip strength. Blood collection will be taken for testing sickle cell disease after getting the consent from one participant per household. The measurements of the biomarkers should be accurately recorded in the field using the prescribed paper format. Open the "Filed Investigation Table" module in the DBMS to enter the obtained data once all anthropometric measurements and additional blood tests have been performed.

**Sample ID**

Q. 1: The field investigation table will be starts with the sample ID which was given in the last of each of the individual questionnaire. This is the identification mark for connecting individual interview data with the biomarker data. Once the sample ID gives, the name, age & gender of the individual automatically identifies by the DBMS.

**Weight**

Q. 2: this question measuring the weight of the individual in kilogram. If weight was not measured enter the code of respective reason for not taking the weight.

**Height**

Q.3: Measuring the person's height in centimeters. Participants of all ages should have their height measured barefoot, and children under two should have their height measured using an infantometer.

**Waist circumference**

Q.4: Record the waist circumference measurement in Q.4. If the waist circumference was not measured record the appropriate reason.

**Hip circumference**

Q.5: Record the hip circumference measurement in Q.5. If the waist circumference was not measured record the appropriate reason.

**Isometric hand grip strength**

Q.6: Grip strength measurement should be measured as per the convenient choice of hand of the participants.

**The systolic and diastolic pressure**

Q.7: Systolic & Diastolic measurement should be recorded twice and the average to be recorded. The average of the two measurements should be enter as final systolic/diastolic measurement value

**Blood glucose**

Q.8 – Q.9: Before testing blood glucose, ask the participants when he/she has taken food last. Enter the response in question 7 and continue with Q.8 for entering the blood glucose level in MG/DL

**Hemoglobin**

Q.10: The measurement of Hb is the primary method of screening for anaemia. Question 10, measures the the Hb level in g/dl. Hb concentration in a drop of blood obtained from a finger or heel prick. The test is rapid, allowing results to be reported to the respondent immediately following the testing procedure.

**SCD/SCT percentage**

Q.11: This is collecting for testing Sickle cell condition. Sample ID/Barcode of the vacutainer should be cross checked before collection of sample. For the participants to be tested for Sickle cell, the blood from the syringe should be 1st collected in EDTA vacutainer then in gel vacutainer. Once the test done the results of SCD is displayed, record the screen shot using mobile as well as write the value.

*Note: The "Manual of Procedure (MOP)" document's section on "Anthropometric Tests" has further information regarding details about biomarker tests.*
