# Supplementary material for: Odisha tribal family health survey: methods, tools, and protocols for a comprehensive health assessment survey
Source: Front Public Health. 2023 Jul 10;11:1157241. doi: 10.3389/fpubh.2023.1157241 (PMC10364047; doi:10.3389/fpubh.2023.1157241)
Supplement: Supplementary file 5 [file Table_5.DOCX]

| **OTFHS - Key Indicators** | | |  |
| --- | --- | --- | --- |
| **Cluster** | |  |  |
| 1. Number of clusters covered | |  |  |
| 1. Average households in the cluster | |  |  |
| 1. Average ST households in the cluster | |  |  |
| 1. Cluster with an improved drinking–water source^1^ (%) | |  |  |
| 1. Cluster with electricity (%) | |  |  |
| 1. Cluster with mobile network connectivity (%) | |  |  |
| 1. Any outbreak/ communicable disease reported in the cluster during the last one year (%) | |  |  |
| 1. Nearest Health Facilities available in the cluster (%) | |  |  |
| - 1. ICDS - Anganwadi | |  |  |
| - 1. Sub-Centre | |  |  |
| - 1. Health & Wellness Center | |  |  |
| - 1. Primary Health Centre (PHC) | |  |  |
| - 1. Community Health Centre (CHC)/ Rural Health Centre | |  |  |
| - 1. District/ Govt. Hospital | |  |  |
| **Household** | |  |  |
| 1. Population below age 15 years (%) | |  |  |
| 1. Sex ratio at birth for children born in the last five years (females per 1,000 males) | |  |  |
| 1. Population living in households with electricity (%) | |  |  |
| 1. Population living in households with an improved drinking-water source^2^ (%) | |  |  |
| 1. Population living in households that use an improved sanitation facility^3^ (%) | |  |  |
| 1. Households using clean fuel for cooking^4^ (%) | |  |  |
| 1. Households using iodized salt (%) | |  |  |
| 1. Households with any usual member covered under a health insurance/financing scheme (%) | |  |  |
| 1. Any member of the household injured in last 12 months (%) | |  |  |
| 1. Prevalence of hemoglobinopathies % | |  |  |
| **Individual** | |  |  |
| **Age 0 – 4 Years Old Children** | |  |  |
| 1. Population below age 5 years | |  |  |
| 1. Sex ratio at birth for children born in the last five years (females per 1,000 males) | |  |  |
| 1. Children under age 5 years whose birth was registered with the civil authority (%) | |  |  |
| ***Child Vaccinations and Vitamin A Supplementation*** | |  |  |
| 1. Children age 12-23 months fully vaccinated based on information from either vaccination card or mother's recall (%) | |  |  |
| 1. Children age 12-23 months fully vaccinated based on information from vaccination card only^5^ (%) | |  |  |
| 1. Children age 12-23 months who have received BCG (%) | |  |  |
| 1. Children age 12-23 months who have received 3 doses of polio vaccine^6^ (%) | |  |  |
| 1. Children age 12-23 months who have received 3 doses of penta or DPT vaccine (%) | |  |  |
| 1. Children age 12-23 months who have received the first dose of measles-containing vaccine (MCV) (% | |  |  |
| 1. Children age 24-35 months who have received a second dose of measles-containing vaccine (MCV) (%) | |  |  |
| 1. Children age 12-23 months who have received 3 doses of rotavirus vaccine (%) | |  |  |
| 1. Children age 12-23 months who have received 3 doses of penta or hepatitis B vaccine (%) | |  |  |
| 1. Children age 9-35 months who received a vitamin A dose in the last 6 months (%) | |  |  |
| 1. Children age 12-23 months who received most of their vaccinations in a public health facility (%) | |  |  |
| 1. Children age 12-23 months who received most of their vaccinations in a private health facility (%) | |  |  |
| ***Child Feeding Practices*** | |  |  |
| 1. Children under age 3 years breastfed within one hour of birth (%) | |  |  |
| 1. Children under age 6 months exclusively breastfed (%) | |  |  |
| 1. Children age 6-8 months receiving solid or semi-solid food and breastmilk (%) | |  |  |
| 1. Breastfeeding children age 6-23 months receiving an adequate diet7 (%) | |  |  |
| 1. Non-breastfeeding children age 6-23 months receiving an adequate diet^8^ (%) | |  |  |
| 1. Total children age 6-23 months receiving an adequate diet^8^ (% | |  |  |
| ***Treatment of Childhood Diseases (children under age 0 - 4 years)*** | |  |  |
| 1. Prevalence of diarrhoea in the 2 weeks preceding the survey (%) | |  |  |
| 1. Children with diarrhoea in the 2 weeks preceding the survey who received oral rehydration salts (ORS) (%) | |  |  |
| 1. Children with diarrhoea in the 2 weeks preceding the survey taken to a health facility or health provider (%) | |  |  |
| 1. Prevalence of symptoms of acute respiratory infection (ARI) in the 2 weeks preceding the survey (%) | |  |  |
| 1. Children with fever or symptoms of ARI in the 2 weeks preceding the survey taken to a health facility or health provider (%) | |  |  |
| 1. Prevalence of anemia in the 1 month preceding the survey (%) | |  |  |
| **Age 5 – 9 Years Old Children** | |  |  |
| 1. Population below age 5 years | |  |  |
| 1. Children currently going to school | |  |  |
| ***Treatment of Childhood Diseases*** | |  |  |
| 1. Prevalence of diarrhoea in the 2 weeks preceding the survey (%) | |  |  |
| 1. Children with diarrhoea in the 2 weeks preceding the survey who received oral rehydration salts (ORS) (%) | |  |  |
| 1. Children with diarrhoea in the 2 weeks preceding the survey taken to a health facility or health provider (%) | |  |  |
| 1. Prevalence of symptoms of acute respiratory infection (ARI) in the 2 weeks preceding the survey (%) | |  |  |
| 1. Children with fever or symptoms of ARI in the 2 weeks preceding the survey taken to a health facility or health provider (%) | |  |  |
| 1. Prevalence of anemia in the 1 month preceding the survey (%) | |  |  |
| ***Iron Folic Acid (IFA)Supplementation and deworming medications*** | |  |  |
| 1. Children receiving Iron and Folic Acid (IFA) supplement/tablets (%) | |  |  |
| 1. Children consuming Iron and Folic Acid (IFA) supplement/tablets as per recommendation (%) | |  |  |
| 1. Children receiving deworming tablets/syrup? (%) | |  |  |
| ***Mid-day Meal programme & Absenteeism*** | |  |  |
| 1. Children currently going to school receiving mid-day meal (%) | |  |  |
| 1. Children who discontinued school in the last 15 days because of sickness (%) | |  |  |
| **Age 10 – 19 Years & 20 – 59 Years Male** | | | |
| **Characteristics of Male** | **Age 10 – 19 Years** | **20 – 59 Years** |  |
| 1. Male population |  |  |  |
| 1. Male population currently in education |  |  |  |
| 1. Male population who are literate |  |  |  |
| 1. Male with 10 or more years of schooling |  |  |  |
| 1. Male population who ever used the internet |  |  |  |
| ***Iron Folic Acid (IFA)Supplementation and deworming medications*** |  | NA |  |
| 1. Children receiving Iron and Folic Acid (IFA) supplement/tablets (%) |  | NA |  |
| 1. Children consuming Iron and Folic Acid (IFA) supplement/tablets as per recommendation (%) |  | NA |  |
| 1. Children receiving deworming tablets/syrup? (%) |  | NA |  |
| ***Marriage and Fertility*** |  |  |  |
| 1. Men age 25-29 years married before age 21 years (%) | **NA** |  |  |
| ***Tobacco Use and Alcohol Consumption*** |  |  |  |
| 1. Male population who use any kind of tobacco (%) |  |  |  |
| 1. Male population who consume alcohol (%) |  |  |  |
| 1. Male population who ever done tattooing (%) |  |  |  |
| 1. Male population who ever done piercing (%) |  |  |  |
| ***Chronic Disease Conditions*** |  |  |  |
| 1. Male population ever diagnosed with Diabetes |  |  |  |
| 1. Male population ever diagnosed with hypertension |  |  |  |
| 1. Male population ever diagnosed with Sickle cell condition |  |  |  |
| 1. Male population ever diagnosed with Tuberculosis |  |  |  |
| 1. Male population ever diagnosed with Cancer |  |  |  |
| ***Knowledge of HIV/AIDS*** |  |  |  |
| 1. Men who have comprehensive knowledge |  |  |  |
| 1. Men who know that consistent condom use can reduce the chance of getting HIV/AIDS |  |  |  |
| **Age 10 – 19 Years & 20 – 59 Years Female** | | | |
| ***Characteristics of Females*** | **Age 10 – 19 Years** | **20 – 59 Years** |  |
| 1. Female population |  |  |  |
| 1. Female population currently in education |  |  |  |
| 1. Female population who are literate |  |  |  |
| 1. Female with 10 or more years of schooling |  |  |  |
| 1. Female population who ever used the internet |  |  |  |
| ***Marriage and Fertility*** |  |  |  |
| 1. Females married before age 18 years (%) |  |  |  |
| 1. Total fertility rate (children per woman) |  |  |  |
| 1. Females who was already mothers or pregnant at the time of the survey (%) |  |  |  |
| 1. Adolescent fertility rate for women age 15-19 years (Births per 1000 women) |  |  |  |
| ***Current Use of Family Planning Methods (Currently Married Women)*** |  |  |  |
| 1. Any method (%) |  |  |  |
| 1. Any modern method (%) |  |  |  |
| 1. Female sterilization (%) |  |  |  |
| 1. Male sterilization (%) |  |  |  |
| 1. IUD/PPIUD (%) |  |  |  |
| 1. Pill (%) |  |  |  |
| 1. Condom (%) |  |  |  |
| 1. Injectables (%) |  |  |  |
| ***Maternal and Child Health*** |  |  |  |
| 1. Maternity Care (for last birth in the 5 years before the survey) |  |  |  |
| 1. Mothers who had an antenatal check-up in the first trimester (%) |  |  |  |
| 1. Mothers who had at least 4 antenatal care visits (%) |  |  |  |
| 1. Mothers whose last birth was protected against neonatal tetanus9 (%) |  |  |  |
| 1. Mothers who consumed iron folic acid for 100 days or more when they were pregnant (%) |  |  |  |
| 1. Registered pregnancies for which the mother received a Mother and Child Protection (MCP) card (%) |  |  |  |
| 1. Mothers who received postnatal care from a doctor/nurse/LHV/ANM/midwife/other health personnel within 2 days of delivery (%) |  |  |  |
| 1. Average out-of-pocket expenditure per delivery in a public health facility (Rs.) |  |  |  |
| 1. Children born at home who were taken to a health facility for a check-up within 24 hours of birth (%) |  |  |  |
| 1. Children who received postnatal care from a doctor/nurse/LHV/ANM/midwife/other health personnel within 2 days of delivery (%) |  |  |  |
| 1. Delivery Care (for births in the 5 years before the survey) |  |  |  |
| 1. Institutional births (%) |  |  |  |
| 1. Institutional births in public facility (%) |  |  |  |
| 1. Home births that were conducted by skilled health personnel10 (%) |  |  |  |
| 1. Births attended by skilled health personnel10 (%) |  |  |  |
| 1. Births delivered by caesarean section (%) |  |  |  |
| 1. Births in a private health facility that were delivered by caesarean section (%) |  |  |  |
| 1. Births in a public health facility that were delivered by caesarean section (%) |  |  |  |
| ***Tobacco Use and Alcohol Consumption*** |  |  |  |
| 1. Female population who use any kind of tobacco (%) |  |  |  |
| 1. Female population who consume alcohol (%) |  |  |  |
| 1. Female population who ever done tattooing (%) |  |  |  |
| 1. Female population who ever done piercing (%) |  |  |  |
| ***Chronic Disease Conditions*** |  |  |  |
| 1. Female population ever diagnosed with Diabetes |  |  |  |
| 1. Female population ever diagnosed with hypertension |  |  |  |
| 1. Female population ever diagnosed with Sickle cell condition |  |  |  |
| 1. Female population ever diagnosed with Tuberculosis |  |  |  |
| 1. Female population ever diagnosed with Cancer |  |  |  |
| ***Knowledge of HIV/AIDS*** |  |  |  |
| 1. Females who have comprehensive knowledge |  |  |  |
| 1. Females who know that consistent condom use can reduce the chance of getting HIV/AIDS |  |  |  |
| ***Women's Empowerment*** |  |  |  |
| 1. Currently married women who usually participate in three household decisions^8^ |  |  |  |
| 1. Female who worked in the last 12 months and were paid in cash (%) |  |  |  |
| 1. Female owning a house and/or land (alone or jointly with others) (%) |  |  |  |
| 1. Female having a bank or savings account that they themselves use (%) |  |  |  |
| 1. Female having a mobile phone that they themselves use (%) |  |  |  |
| 1. Female age 15-24 years who use hygienic methods of protection during their menstrual period^9^ (%) |  |  |  |
| ***Gender Based Violence*** |  |  |  |
| 1. Ever-married women who have ever experienced spousal violence^10^ (%) |  |  |  |
| 1. Ever-married women who have experienced physical violence during any pregnancy (%) |  |  |  |
| 1. Young women age 18-29 years who experienced sexual violence by age 18 (%) |  |  |  |
| **Age 60 & above Years** | | | |
| **Characteristics of Geriatric group** |  |  |  |
| 1. Geriatric population |  |  |  |
| 1. Geriatric population who are literate (%) |  |  |  |
| 1. Geriatric population with 10 or more years of schooling (%) |  |  |  |
| 1. Geriatric population who ever used the internet (%) |  |  |  |
| 1. Geriatric Population receiving pension (%) |  |  |  |
| 1. Geriatric Population having health insurance (%) |  |  |  |
| ***Tobacco Use and Alcohol Consumption*** |  |  |  |
| 1. Geriatric population who use any kind of tobacco (%) |  |  |  |
| 1. Geriatric population who consume alcohol (%) |  |  |  |
| 1. Geriatric population who ever done tattooing (%) |  |  |  |
| 1. Geriatric population who ever done piercing (%) |  |  |  |
| ***Chronic Disease Conditions*** |  |  |  |
| 1. Geriatric population ever diagnosed with Diabetes (%) |  |  |  |
| 1. Geriatric population ever diagnosed with hypertension (%) |  |  |  |
| 1. Geriatric population ever diagnosed with Arthritis (%) |  |  |  |
| 1. Geriatric population ever diagnosed with Sickle cell condition (%) |  |  |  |
| 1. Geriatric population ever diagnosed with Tuberculosis (%) |  |  |  |
| 1. Geriatric population ever diagnosed with Cancer (%) |  |  |  |
| ***Functional Limitations*** |  |  |  |
| 1. Geriatric population having physical or mental impairment (%) |  |  |  |
| - 1. Hearing impairment (%) |  |  |  |
| - 1. Visual impairment (%) |  |  |  |
| - 1. Speech impairment (%) |  |  |  |
| - 1. Mental impairment (%) |  |  |  |
| 1. Geriatric population using any air or supportive devices (%) |  |  |  |
| ***Quality of life*** |  |  |  |
| - 1. Impaired Mobility (%) |  |  |  |
| - 1. Pain/discomfort (%) |  |  |  |
| - 1. Anxiety/ Depression (%) |  |  |  |
| - 1. Impaired Self-Care (%) |  |  |  |
| - 1. Unusual Activities (work, study, housework, family or leisure activities) (%) |  |  |  |
| *Note:*  *^1& 2^Piped water into dwelling/yard/plot, piped to neighbour, public tap/standpipe, tube well or borehole, protected dug well, protected spring, rainwater, tanker truck, cart with small tank, bottled water, community RO plant.*  *^3^Flush to piped sewer system, flush to septic tank, flush to pit latrine, flush to don't know where, ventilated improved pit (VIP)/biogas latrine, pit latrine with slab, twin pit/composting toilet, which is not shared with any other household.*  *^4^Electricity, LPG/natural gas, biogas.*  *^5^Among children whose vaccination card was shown to the interviewer, percentage vaccinated with BCG, measles-containing vaccine (MCV)/MR/MMR/Measles, and 3 doses each of polio (excluding polio vaccine given at birth) and DPT or penta vaccine.*  *^6^Not including polio vaccination given at birth.*  *^7^Breastfed children receiving 4 or more food groups and a minimum meal frequency, non-breastfed children fed with a minimum of 3 Infant and Young Child Feeding Practices (fed with other milk or milk products at least twice a day, a minimum meal frequency that is, receiving solid or semi-solid food at least twice a day for breastfed infants 6-8 months and at least three times a day for breastfed children 9-23 months, and solid or semi-solid foods from at least four food groups not including the milk or milk products food group).*  *^8^Decisions about health care for herself, making major household purchases, and visits to her family or relatives.*  *^9^locally prepared napkins, sanitary napkins, tampons, and menstrual cups are considered to be hygienic methods of protection.*  *^10^Spousal violence is defined as physical and/or sexual violence.* | | | |
