# Supplementary material for: Odisha tribal family health survey: methods, tools, and protocols for a comprehensive health assessment survey
Source: Front Public Health. 2023 Jul 10;11:1157241. doi: 10.3389/fpubh.2023.1157241 (PMC10364047; doi:10.3389/fpubh.2023.1157241)
Supplement: Supplementary file 6 [file Data_Sheet_1.pdf]

OTFHS

MANUAL OF PROCEDURES

OTFHS

ICMR- REGIONAL MEDICAL RESEARCH CENTRE, BHUBANESWAR

Contents

Introduction ... 3

Survey Design ... 5

Stratification ... 5

Sample size ... 5

Household selection ... 11

Household listing ... 11

Monitoring ... 15

Field Monitoring ... 17

Internal monitoring team ... 21

External monitoring team ... 25

Data Quality Monitoring ... 27

Monitoring – Laboratory Practices in the field ... 36

Anthropometric Measurement & Biomarker tests ... 40

Blood Sample Collection in Field ... 64

Biomedical Waste Management ... 98

Mop travel ... 109

Mop administration ... 111

Mop roles & responsibilities of staffs ... 115

Mop role and function of SCSTRTI ... 121

Mop local liaison ... 122

Organogram & Hr ... 123

Sexual Harassment ... 124

Annexures ... 124

Introduction

Odisha Tribal Family Health survey is the first state-level health survey which is focused on the comprehensive evaluation of the health, demographic, and socio-economic parameters of the tribal population in Odisha, India.

The survey will consider indicators related to demography, socio-economic status, maternal health, child health, adolescent health, adult health, and elderly health. The survey will focus on comparing the health status between all the 62 Scheduled Tribes and 13 PVTGs in Odisha, which is the first of its kind. The OTFHS is a state project and is implemented across various tribal sub-plan areas of Odisha. In order to achieve comparability, consistency and the best quality in survey results, sampling activities in the OTFHS is guided by number of sampling principles.

This manual present general guidelines on sampling, monitoring, laboratory practices and administrative processes for OTFHS survey.

Survey objectives

1. To describe and compare the health status of tribal groups in Odisha
2. To estimate the prevalence of crucial maternal-child health indicators and chronic diseases among the tribal groups in Odisha
3. To assess the demographic and socio-economic status of the tribal communities in Odisha

The fundamental principles of OTFHS sampling include:

1. Use of an existing sampling frame
2. Complete coverage of the target population
3. Multistage sampling
4. Probability sampling
5. Using a suitable sample size

©ICMR-RMRC, Bhubaneswar

6. Using the simplest design possible
7. Conducting a household listing and pre-selection of households
8. Providing good sample documentation
9. Maintaining confidentiality of individual's information
10. Implementing the sampling strategy precisely as designed

Sampling frame

The sampling frame used in OTFHS is the list of Enumeration Areas (EAs) from a recently completed population census (2011). An EA is usually a geographic area which groups a number of households together for convenient counting purposes for the census. The list in most cases, a list of EAs from a recent census is available. The sampling frame used for OTFHS should be as up to date as possible. The details of Scheduled tribes and PVTGs is evaluated from the census data (Annexure-1). The district wise tribal data with the details of the Tribal population in each village will be collected from the State and district offices.

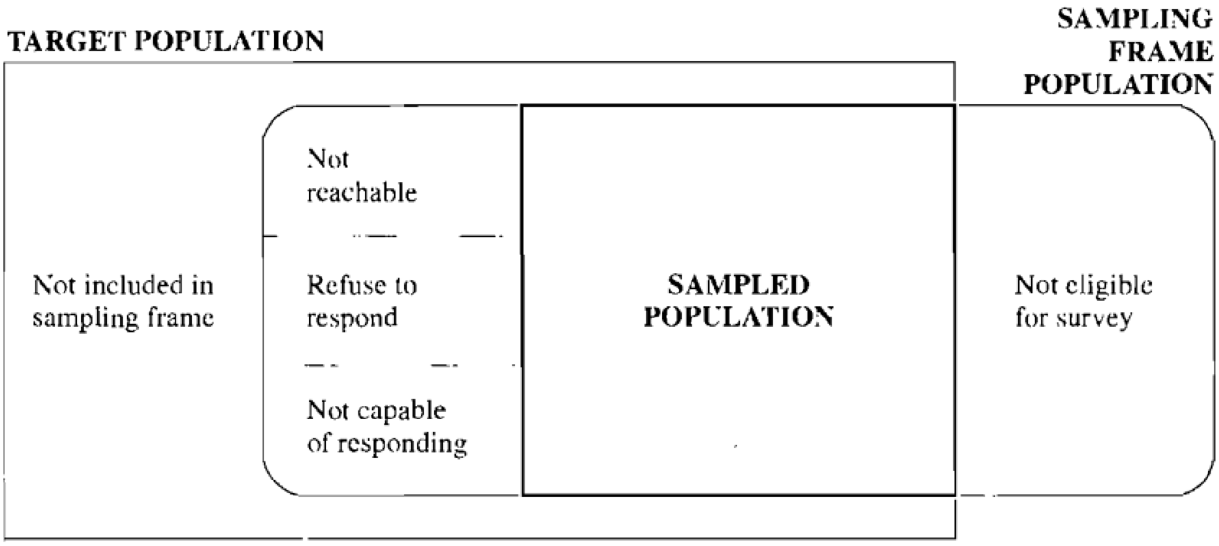

Survey Design

OTFHS is a population based cross sectional survey which will be carried out in Odisha. Odisha has 30 administrative districts with population of 4.37 crores in which schedule tribe population is around 8145081 (22.5%). From these 30 administrative districts 13 districts were selected which have Tribal Sub Plan areas in them. The Tribal Sub-plan (TSP) areas include Tribal Sub-Plan blocks or tehsils with 50 percent or more tribal population. This planning concept was introduced in 5th Five Year Plan (1974-1979). The study setting was the Tribal Sub-Plan (TSP) blocks of Odisha state which were under the 13 districts. Total 314 numbers of blocks were present in the 13 districts in which we have included 119 blocks. We have included all the tribal community (62 STs and 13 PVTGs). The primary sampling units were clusters and the secondary sampling units were the Households.

Stratification

Stratification is the process by which the survey population is divided into subgroups or strata that are as homogeneous as possible using certain criteria. The survey population is stratified into different Tribes and PVTGs. The stratification is introduced at the first stage of sampling.

Sample size

The calculations for OTFHS are based on the expected proportion of high/very high blood sugar (>140 mg/dl) = 13.3% [NFHS-5]. The sample size was calculated based on the following formula

$$n = [DEFF*Np(1-p)] / [(d^2/Z^2_{1-\alpha/2}*(N-1)+p*(1-p)] * \text{Finite Population Adjustment}$$

where:

- N = Total tribe population
- p = expected proportion of high/ very high blood sugar
- d = Relative precision of 30%

- CI=95%;
- α=0.05;
- β=0.2;
- ICC=0.0191 (Bischops, 2020);
- For smaller tribes where the sample size needed was greater than 5% of the entire tribal population, a finite population correction (FPC) was used. Formula used for FPC was $\sqrt{(N-n)/(N-1)}$
- The tribes were classified into 3 categories based on the population of the tribe as below:
    - Category-1 Less than 10000
    - Category-2 10000 to 100000
    - Category-3 More than 100000

  Assuming a uniform Cluster size of 30, design effect was estimated for each of the above-mentioned tribe categories as below:
    - Category-1 →1.2
    - Category-2 → 1.4
    - Category-3 → 2.8
- Response rate of 90%;
- Avg. HH size=4

The necessary sample size for individuals were calculated and then the number was extrapolated to the number of households. Back calculation of power for aggregate analyses were used.

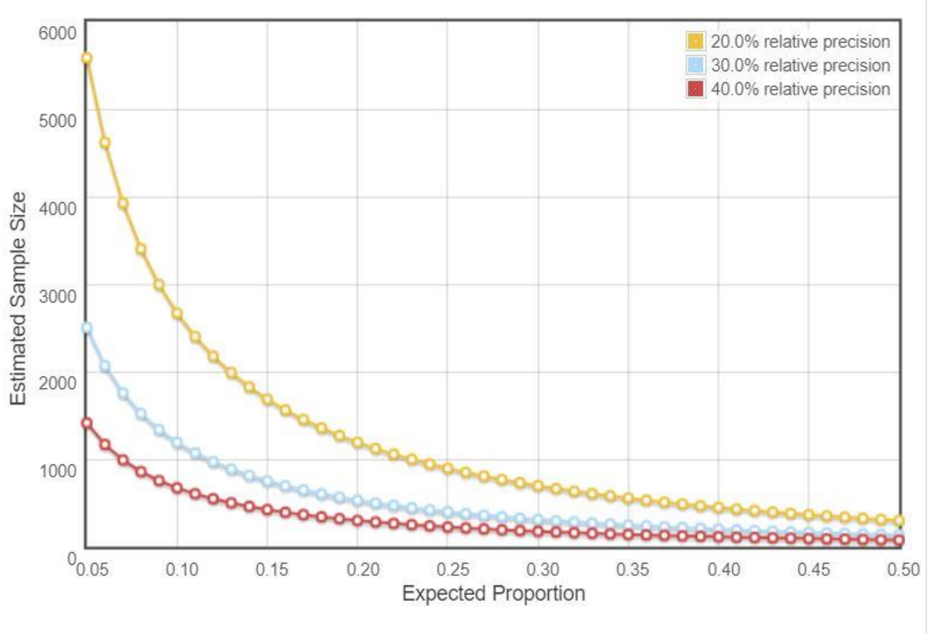

The calculated individual specific sample size is given in Annexure- 1, by assumed non-response rate as 90% the total Sample size calculated for the survey was 42048 from 10489 households. The number of Households will be sampled from 350 clusters across the Tribal sub plan areas in the state covering 62 tribes and 13 PVTGs.

Sampling design

The survey uses multistage sampling design in which 13 districts of Tribal Sub Plan areas were taken. The sampling strategy used is represented in the flow chart

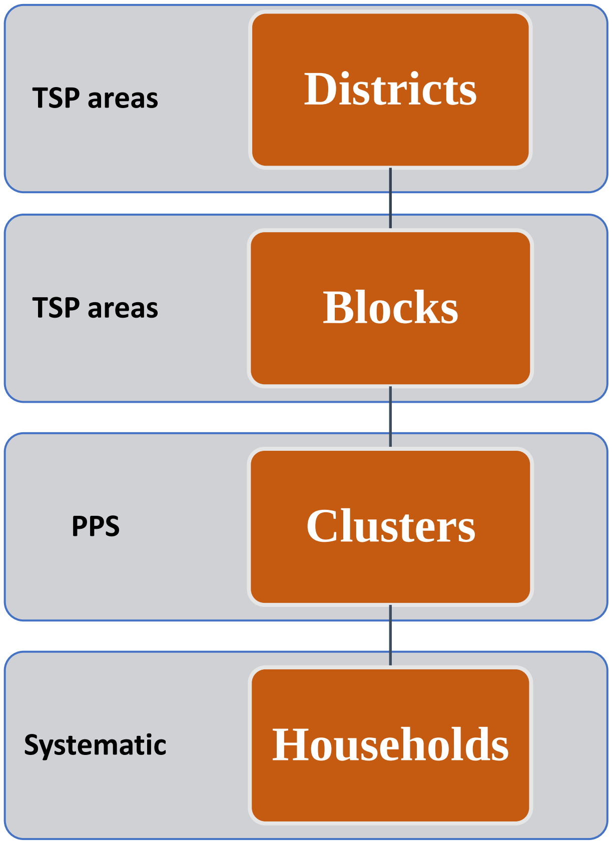

Selection of districts and blocks

In the survey we have selected all the districts listed in the Tribal- Sub Plan (TSP) areas of Odisha. We have included all the Tribal sub plan blocks (119) under each district

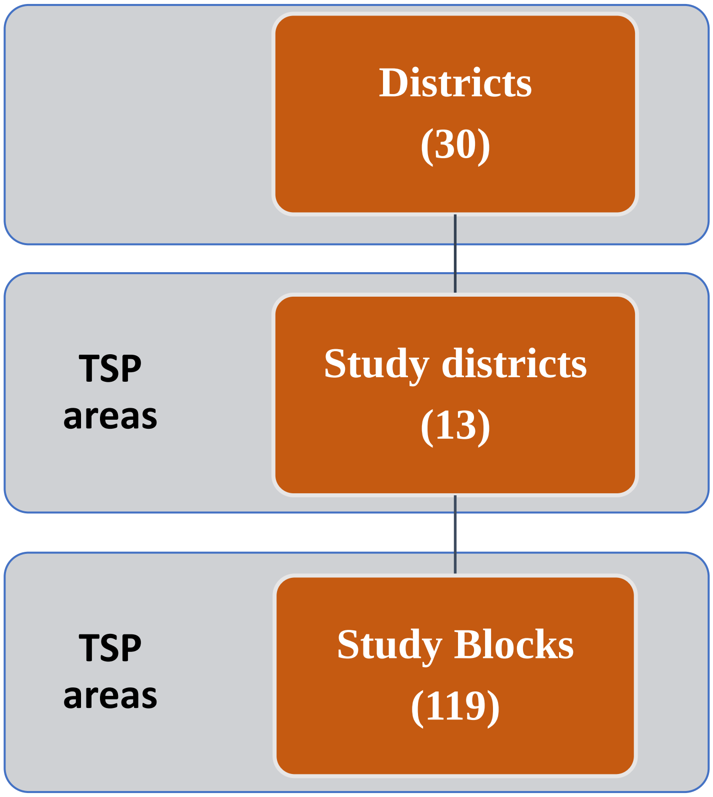

Cluster selection

In the first stage of sampling, a stratified sample of Tribal population is selected from different districts through proportionate allocation. In each stratum number of clusters is predetermined and then the number of clusters in each district is calculated based on the proportion allocation of each tribe in the district. Purposive sampling method uses of smaller tribes living in small geographic areas. Total number of clusters is then finally calculated for each district.

Tribes	Districts							Total
	District -1	District -2	District -3	District -4	District -5	District -6	District -n	
Tribe -1								

**Tribe -2**								
**Tribe -3**								
**Tribe -4**								
**Tribe -n**								
**Total**								

The clusters from each districts are selected by probability proportional to size. The cluster selection was carried out in the state office. Scheduled tribes and PVTGs having less sample size and concentrated in limited geographical area were purposively selected.

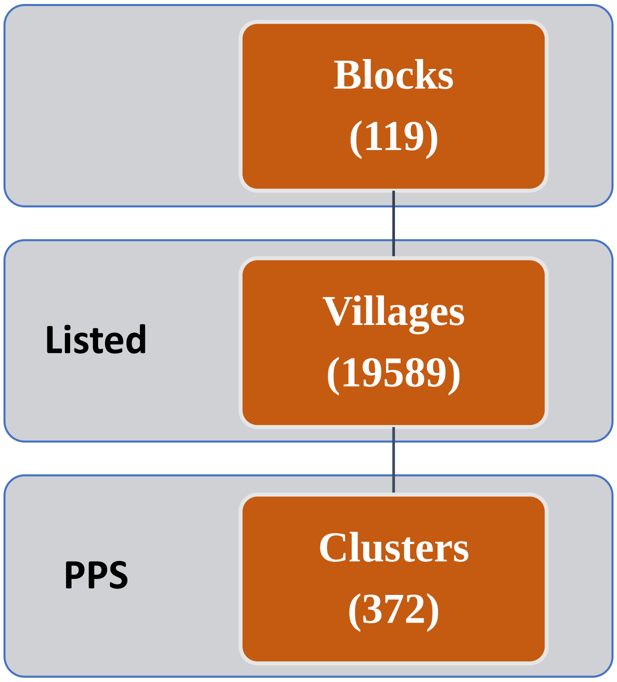

Required number of clusters from each district

Sl.no	Name of district	Required no. of clusters
1	Sambalpur	25
2	Debagarh	11
3	Sundargarh	57
4	Kendujhar	39
5	Mayurbhanj	56
6	Balasore	21
7	Gajapati	10
8	Kandhamal	7
9	Kalahandi	21
10	Rayagada	15
11	Nabarangpur	29
12	Koraput	41
13	Malkangiri	40
	Total	372

Household selection

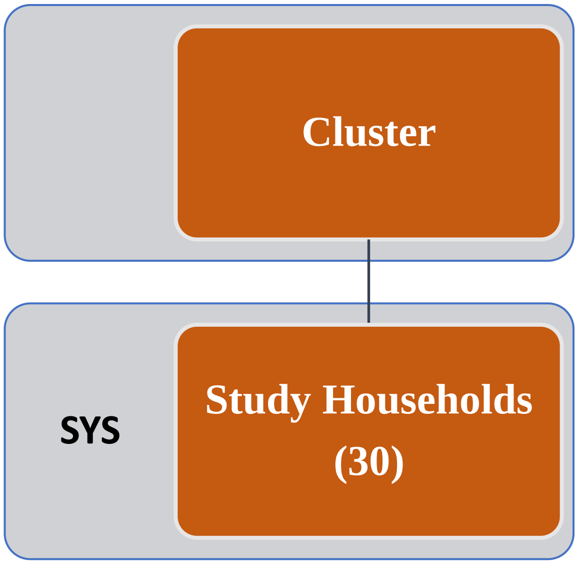

In the second stage, a complete household listing is conducted in each of the selected clusters, a fixed number of households is selected by equal probability systematic sampling in the selected clusters. In each selected household, a household questionnaire is completed to identify all eligible study participants in the household. Every eligible study participant will be interviewed with individual questionnaire in those households, and one samples will be collected from each household based on Age-order matrix. The Household selection will be carried out in the field.

Household listing

A household consists of a person or a group of related or unrelated persons, who live together in the same dwelling unit, who acknowledge one adult male or female 15 years old or older as the head of the household, who share the same housekeeping arrangements, and are considered as one unit. In some cases one may find a group of people living together in the same house, but each person has separate eating arrangements; they should be counted as separate one-person households. Collective living arrangements such as army camps, boarding schools, or prisons will not be considered as households. Examples of households are:

- a man with his wife or his wives with or without children
- a man with his wife or his wives, his children and his parents

- a man with his wife or his wives, his married children living together for some social or economic reasons (the group recognize one person as household head)
- a widowed or divorced man or woman with or without children

The head of household is the person who is acknowledged as such by members of the household and who is usually responsible for the upkeep and maintenance of the household.

Listing procedure

When a cluster is selected for the survey, a complete list of households in the selected cluster is conducted prior to the selection of households. The listing operation consists of visiting the selected cluster, collecting geographic coordinates of the cluster and collecting number of households in the cluster. This information is necessary for an equal probability random selection of households in the second stage. With the household listing prior to the main survey, it is possible to pre-select the sample households in advance and the interviewers are asked to interview only the pre-selected households without replacement of non-responding households. The precise location information (GIS) for selected clusters will be collected by the geographic positioning systems (GPS) .

Selection of household

Systematic random sampling is the selection of sampling units at a fixed interval from a list, starting from a randomly determined point. Selection is systematic because selection of the first sampling unit determines the selection of the remaining sampling units.

Systematic sampling is normally carried out as follows: assuming a whole number interval k=N/n, where N is the number of units in the list and n is the number of units to be selected. The procedure begins with an integer random number S that is less than or equal to k. The units to be selected are S, S+k, S+2*k, ..., S+(n-1)*k. When k is not a whole number there may be appreciable errors in rounding it to the nearest whole number, it is suggested that the decimal interval method be used. Selection with a decimal interval may be carried out as follows:

1) Calculate the interval I rounded to two decimal places.

2) Generate a random number R between 0 and 1 with two decimal points.

3) Compute the sequence of sampling numbers: R*k, R*k + k, R*k + 2*k, ..., R*k + (n - 1)*k

4) Round up the above calculated sampling numbers to the next highest whole numbers; these are the selected units' numbers.

The sampling interval will be calculated as k = Total households/ Number of required households. The first household will be selected through random number generator/random number table by selecting a random number between 1 and k.

If the sampled household is not selected because of the following reason:

- Household is locked
- Household has migrated
- Household is damaged and no one is living
- ST community not occupying the household

Then the adjacent household will be taken, and the procedure will be carried out until next household is selected.

Responsibilities of staff

Each team consist of four members-

Project Assistant-1

Project Technician (Field Investigator)-1

Project Technician (Laboratory technician)-2

The Project Assistant (RA) will monitor the entire operation. The responsibilities of the PA are to:

- obtain number of households for all the clusters included in the survey;
- keep all the materials (manuals, letters)
- obtain one GIS location for each cluster

- list all the households in the cluster in a systematic manner and select the households through SRS
- communicate to the local health staff/authorities regarding the field visit
- Communicate to the village head/key persons in the village before the field visit.
- Communicate to the main office regarding any issues in the field

➢ The sampling in each cluster will be carried out by the PA of the team. If PA is not available, then the sampling will be carried out by the Project technician (Field Investigator)

➢ The following forms will be completed by the PA of the team while in the field

(1) Household attempt form
(2) Household listing form

Household attempt form- In the form the details of the attempt made in the cluster will be documented. In general, the team will visit a cluster for 3 days (approx.), so for data and sample collection the number of attempts made will be documented in this form.

Household listing form- In this form details regarding the household with individual ID and sample ID will be collected. The details of the individual will also be collected in the form which includes the name, age and gender of the study participants.

The form will be filled by the PA during the data collection. In case PA of the team is not available, the FI will fill the respective form.

Ancillary care referral plan - Following the assessment of health conditions undertaken as part of the survey, any individual identified with abnormal findings or suggestive of potential healthcare need, will be provided a detailed report (Additional File-4) in a standard format along with advice for referral to the nearest public health facility for further management. A copy of the same will be forwarded to the health facility for their information, through the village

health worker. All other bedridden and/or seriously ill patients identified during the survey will also be referred to the nearest health facility.

The Field Investigator will collect the data and will complete the consent form prior to data collection. He will make sure the consent forms are properly filled and the signature/thumb print is present in the consent form. The following forms will be completed by the FI of the team

1. Consent form
2. Travel logbook

Travel logbook form- The travel logbook is the details regarding daily travel in the field. In case the FI is not present the LT will fill the form.

The LT of the team will collect the sample of the study participants who will provide their consent. He will be responsible for maintaining the following forms

(1) Stock logbook
(2) Sample logbook

Stock logbook- The stock logbook contains the details of consumables that will be used in the field. The list will be maintained by the field team LT.

Sample logbook- The sample logbook is details regarding the Samples collected. The sample logbook will be completed and will be sent to the main office with the samples.

Monitoring

A well-functioning monitoring, evaluation and learning system is an important part of the survey for management and accountability. Structured, timely and reliable monitoring and evaluation will provide information to:

1. Support the survey with accurate, evidence-based reporting that informs management and decision-making to guide and improve performance

2. Contribute to organizational learning and knowledge sharing by reflecting upon and sharing experiences and lessons so that benefit can be derived from what is being done and how it is being done.
3. Ensure accountability and compliance by demonstrating whether the work is being carried out as agreed, and in compliance with established standards of ICMR and in line with donor requirements.
4. Promote and recognize accomplishments and achievements, building morale and contributing to resource mobilization.

Goal and Objectives

The monitoring and evaluation (M&E) strategy is the key driver for the Odisha Tribal Family Health Survey (OTFHS) which acknowledges the necessity of developing a functional and robust M&E system for OTFHS to provide useful and timely information to program managers, management committee and funding agency. The program managers will track the progress of the Survey to ensure the achievement of results through necessary course corrections during the implementation of the survey.

Goal

The goal is to establish a sustainable M&E system for tracking progress and demonstrating the results of the Survey and to ensure evidence-based practice.

Specific objectives

The specific objectives of the M&E Strategy are to:

1) Improve the quality of the routine data collection systems, e.g., development of registries, routine data collection forms, type, and frequency of reports, etc.

2) Outline specific activities required for strengthening the organizational capacity to conduct effective M&E

Key outputs

The expected key outputs of the M&E Strategy are:

1) Prepare an M&E Action Plan for the survey

2) A functional robust, comprehensive, and well-coordinated M&E system for OTFHS in place; 3) Regular updates on performance indicators available.

4) Project/Survey progress reports are produced on time.

Outcomes

The M&E Strategy is expected to result in:

1) Promoting the practice of evidence-based research

2) Reporting in time to the ICMR-RMRC management committee and funding partners

3) Objective decision making for performance improvement; planning and resource allocation; and Promoting accountability of ICMR-RMRCBB.

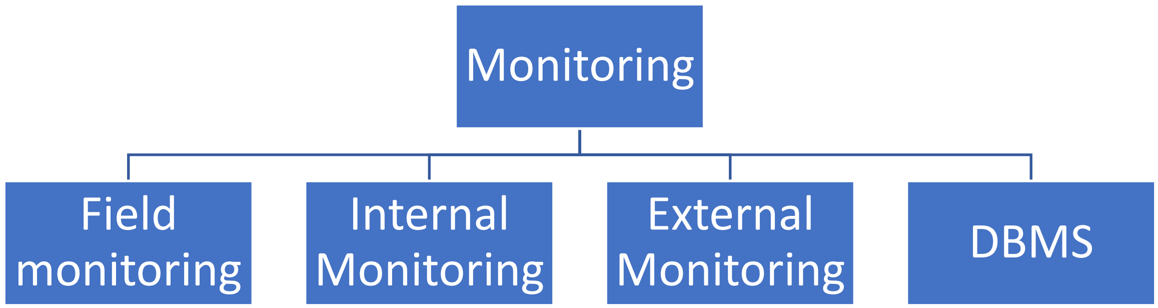

Field Monitoring

Field Monitoring is built in mechanism in the survey which will be continuous throughout the project. Each survey team in the field consists of Project Assistant (Field Supervisor), Field Investigator (FI) and Laboratory technicians (LT). All the staff has their individual role in their team and the field day-to-day activities will be

monitored by the Project Assistant (PA) of the team. The following are the important roles of RA

1. RA are the primary links between the main office and fieldwork, so they are responsible for ensuring both the quality and progress of fieldwork.
2. Monitoring interviewer performance by-
    a. observe at least one interview every day
    b. edit all completed questionnaires in the field; editing must be completed prior to leaving the sample area
    c. conduct regular review sessions with interviewers and advise them of any problems found in their questionnaires.
    d. uploading of the data
3. Daily data quality checks
    a. Data collected is reviewed daily
    b. Data is uploaded on the server daily
4. Maintaining logistics
    a. Consent related to all forms
    b. Biomedical related logistics
    c. Office Stationery
5. Monitoring field expenses
6. Maintaining vehicle logbook

Field checklist which will be maintained by the PA of the team.

Supervisor

1. Attendance sheet
2. Cluster list

3. Household listing
4. Travel Logbook
5. Sample Logbook
6. Manuals
7. Tribe list
8. Stock register
9. Attempt register
10. Completed schedules

<u>Attendance sheet</u>

Si No.	Name	1	2	3	4	5	6	7	8	9	10	11	12	13	14	15	16	17	18	19	20	21	22	23	24	25	26	27	28	29	30	31
1																																
2																																
3																																
4																																
Filled by																																
Verified by-																																

Household listing

Si No.	Household Id	Individual Id	Data Collected			Blood sample collected		
			1st attempt	2nd attempt	3rd attempt	1st attempt	2nd attempt	3rd attempt
Filled by								
Verified by-								

Sample log book

Si No.	Sample ID (Barcode)	Household ID	Individual Id	Sample collected Yes/No	Serum/plasma Collected Yes/No
Filled by					
Verified by-					

Travel Log Book

Si No.	Date	Place	Starting time	Starting Km	Ending time	Ending km	Signature	Remark
Filled by								
Verified by-								

Tribe list (Individual Team Form)

Si No.	Tribe Name	Team Name	Number of Households covered	Number of Samples Collected

The Field Investigator (FI) of the team will look after the collection of data from the field and the completeness of the data before uploading the data. The following checklist will be filled by the FI and the same will be verified by the PA of the team.

The Laboratory Technician will ensure the collection of samples, storage of samples and transportation of samples from the field to the Sample storage unit in the district. He will also make sure all the samples were stored at optimum temperature at the district office.

Internal monitoring team

The internal monitoring team will be formed in the centre (ICMR-RMRCBB). The internal monitoring team will be-

- Managing committee (OTFHS)
- Project Scientist (OTFHS)

OTFHS Management Committee (OMC)

The Managing Committee (MC) will consist of Senior Scientists of ICMR, Administrative Officers, Account Officers, and External experts in the field. We have constituted the management committee in the Centre (RMRCBB) in which the following members are part of the team-

The responsibility of the internal monitoring team (MC) will be-

- Checking the data validity and verify the completeness and adequacy of the data
- Sampling protocol followed in the field
- Field interview observation

- Conducting reinterview in a subset of households (2 HH) and cross-checking with collected field data (10% of the questionnaire)
- Assessment of time stamps and GIS co-ordinates
- Matching of collected data with Lab tally sheet in a subset of households (2 HH)
- Sample storage and maintenance of cold chain in the field
- Frequency- Quarterly (Every 3 months)

The team will use various checklists as provided in the annexures for monitoring the monthly update of the survey. They also monitor the following things in the field while visiting the field team

- Attendance register
- Stock register
- Household listing
- Lab tally sheet
- Sampling protocol followed
- Reinterviewing of 2 HH (10% of the questionnaire)
- Target achieved (Indicator based)

OTFHS Project Scientist Team (OPS)

The internal team of Project Scientists will be the Co-PIs for the project and Project scientists hired for the project of different domain specializations.

The responsibility of the internal monitoring team will be-

- Checking the data validity and verify the completeness and adequacy of the data
- Sampling protocol followed in the field
- Field interview observation

- Conducting reinterview in a subset of households (2 HH) and cross-checking with collected field data (10% of the questionnaire)
- Assessment of time stamps and GIS co-ordinates
- Matching of collected data with Lab tally sheet in a subset of households (2 HH)
- Sample storage and maintenance of cold chain in the field
- Frequency- Monthly

The team will use various checklists as provided in the annexures for monitoring the monthly update of the survey. They also monitor the following things in the field while visiting the field team

- Attendance register
- Stock register
- Household listing
- Lab tally sheet
- Sampling protocol followed
- Reinterviewing of 2 HH (10% of the questionnaire)
- Target achieved (Indicator based)
- Remarks and Suggestions for Improvement

'

Tribe list cumulative

Name of the tribe	Team A	Team B	Team C	Team D	Team E	Team F	Total

Monitoring Checklist

Si No.	Name		
1	Attendance register	Updated<br>1. Yes<br>2. No	
2	Vehicle logbook	Updated<br>1. Yes<br>2. No	
3	Sample logbook	Updated<br>1. Yes<br>2. No	
4	Household tally sheet	Updated<br>1. Yes<br>2. No	

Internal Monitoring Plan

Project Scientist (PS)

	Month-1	Month-2	Month-3	Month-4	Month-5	Month-6
**District Name**						
**District-1**	√	√	√	√	√	√
**District-2**	√	√	√	√	√	√
**District-3**	√	√	√	√	√	√
**District-4**	√	√	√	√	√	√
**District-N**	√	√	√	√	√	√

The same will be repeated for next 6 month

Management Committee (MC)

	Month-1	Month-2	Month-3	Month-4	Month-5	Month-6

District Name						
District-1	√			√		
District-2	√			√		
District-3	√			√		
District-4	√			√		
District-N	√			√		

The same will be repeated for the next 6 month

External monitoring team

The external team members will consist of-

- External experts from Medical Colleges and Hospitals
- External experts from NHM/District Hospital

The responsibility of the internal monitoring team will be-

- Checking the data validity and verify the completeness and adequacy of the data
- Sampling protocol followed in the field
- Field interview observation
- Conducting reinterview in a subset of households (2 HH) and cross-checking with collected field data
- Matching of collected data with Lab tally sheet in a subset of households (2 HH)
- Sample storage and maintenance of cold chain in the field
- Frequency- Every 3 months

The team will evaluate the project's progress and the following details will be documented

- Project progress based on coverage of

  -Tribes

  -Households

  -Samples

  -Logistics usage

- Samples of transportation in district and state
- Field observation
- Suggestion for improvement

External monitoring team (EM)

	**Month-1**	**Month-2**	**Month-3**	**Month-4**	**Month-5**	**Month-6**
**District Name**						
**District-1**			**√**			**√**
**District-2**			**√**			**√**
**District-3**			**√**			**√**
**District-4**			**√**			**√**
**District-N**			**√**			**√**

The same will be repeated for next 6 month

Overall Monitoring Plan

	**Month-1**	**Month-2**	**Month-3**	**Month-4**	**Month-5**	**Month-6**

District Name						
**District-1**	(PS) (MC)	(PS)	(PS) (EM)	(PS) (MC)	(PS)	(PS) (EM)
**District-2**	(PS) (MC)	(PS)	(PS) (EM)	(PS) (MC)	(PS)	(PS) (EM)
**District-3**	(PS) (MC)	(PS)	(PS) (EM)	(PS) (MC)	(PS)	(PS) (EM)
**District-4**	(PS) (MC)	(PS)	(PS) (EM)	(PS) (MC)	(PS)	(PS) (EM)
**District-N**	(PS) (MC)	(PS)	(PS) (EM)	(PS) (MC)	(PS)	(PS) (EM)

***PS- Project Scientist, MC- Management Committee, EM- External Monitoring Team**

The same will be repeated for the next 6 month

Data Quality Monitoring

Ensuring data quality in different stages of the data collection phase and post-data collection phase. Details are given below

1. **Data collection phase - Monitoring Through DBMS**

The Data Base Management System will be used for real-time data monitoring. The Dashboard indicators will show the real-time progress regarding coverage of Districts, Clusters, Households, Samples collected, etc.

Daily Monitoring: DBMS

- Day-wise interview status including response rate
- Interviewer productivity
- The average duration of the interviews
- Negative screening rate
- Key indicators from the field check tables
- Indicators which have a high risk of error during data collection
- Outliers and extreme values in key survey indicators

- Number of missing values in key indicators
- Observations which are duplicates
- List of questions taking most of the interview time
- List of questions with most 'don't know and/or 'no answer' responses

Different stages of data flow in the data collection phase are mentioned in the below diagram.

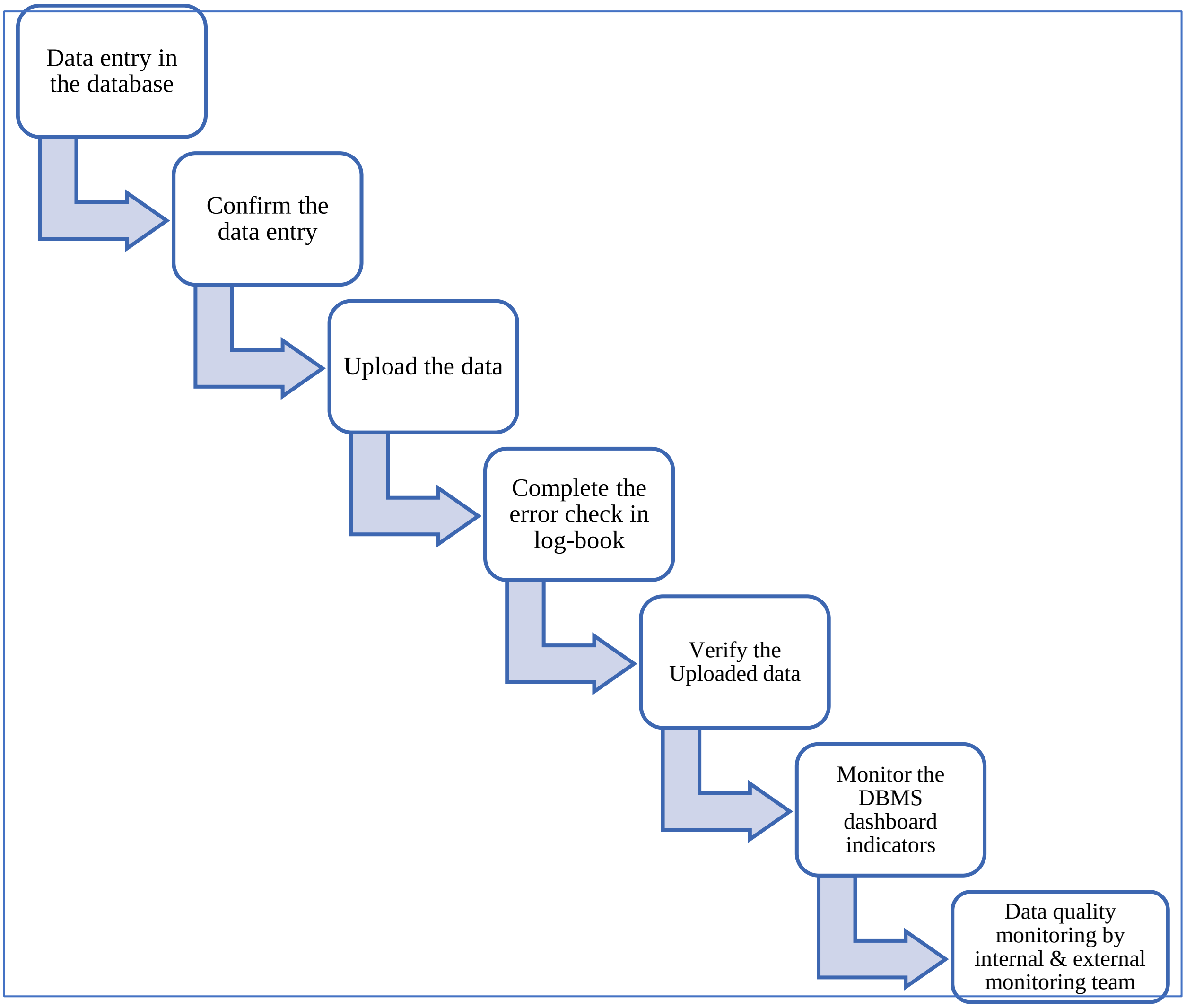

Steps to be followed for data entry & Monitoring

1. To start data entry, first required to log in to connect to the server. Enter the given hostname, username and password to log in to the DBMS platform.
2. Once successfully logged in, it will take to MySQL monitor and it is ready for data entry.
3. There are different databases were created according to the different questionnaires of OTFHS, such as; cluster, household, under 5 years old children, 5-9 years old children, adolescents aged 10-19 years, women and men aged 20-59, the geriatric population of age 60 years and above and field investigation.
4. Start the data entry from the cluster module
5. Once the cluster data entry is completed, the remaining databases under the cluster database will be automatically generated.
6. The second stage is the start of the household data entry, as soon as household data entry completes, there will be a provision to see the individual modules and the field investigation module.
7. Choose the appropriate individual module for starting data entry.
8. Start entering the module by clicking the tab of the created module menu button.
9. The DBMS will ask you to enter the sample ID of the individual just before you finish entering the individual data. This ID will be utilized in the section on biomarker measurement, and it will serve as a link between the other modules and the biomarker module.

10. Here database is a combination of columns and rows; each column represents the variable or questions in the questionnaire and the row represents the complete information about the study participants.
11. An inbuilt algorithm in the MySQL program automatically handles skip patterns, filters, and eligibility for questionnaires and sections.
12. Once the data entry is completed it will show the information about the new data entry that were created. After finishing the one subject data entry move to the next subject entry.
13. After completion of all the data entry, re-check again that all the data were entered correctly or not, if any wrong entry was detected re-enter the correct one. Once everything is cross-checked properly report to RA and upload the data to the server.
14. The provision of synchronizing data from FI's & LT's database to RA's database provides an opportunity for back check of information to improve data quality.
15. Once the data is uploaded to the server there will not be any provision for re-reentering data by the FIs, LTs or RAs. If any errors are noticed in the later stage report to the Central Office.
16. Details of detected errors should be mentioned in the logbook by the FIs and RAs.
17. Once the all data is uploaded, the RA will confirm the data entry about the day and inform the central office.

18. Uploaded data will verify and confirm by the Central Office. If any wrong data entry is detected, immediately revert back to the RA and if possible, will correct it in the database.
19. The DBMS dashboard help in generating field check tables on key indicators on a daily basis which the Quality Assurance Team reviews in the central office to allow individual-level feedback to be communicated back to the teams working across different districts
20. OTFHS assigns a unique code to each field investigator, which helps track the investigator's progress and performance after individual-level feedback is provided.

1. **Post-data collection phase**

Data flow in the post-data collection phase is mentioned in the below diagram.

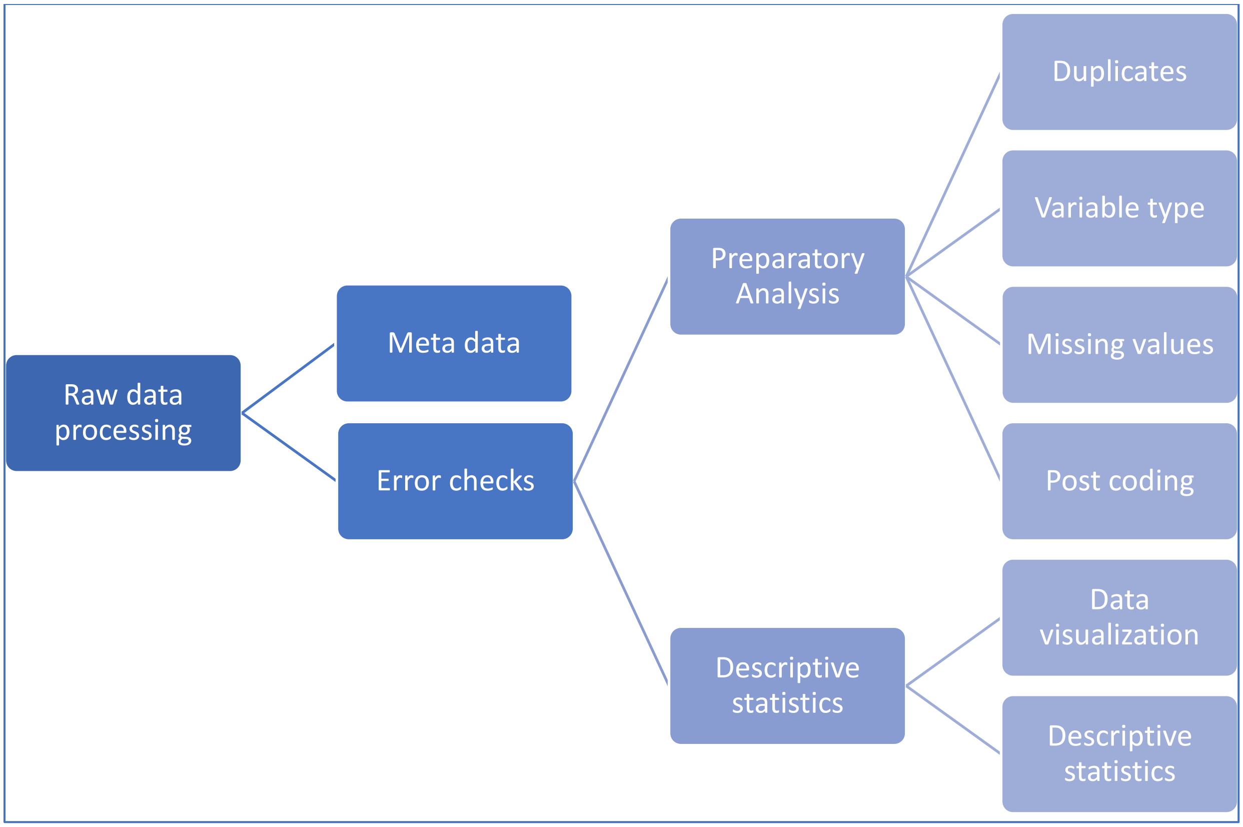

Details of data flow in post data collection phase is given below

Checking errors in raw data

a. ***Preparatory Analysis***: Preparatory analysis is the process of cleaning and transforming raw data prior to processing and analysis. It involves reformatting, correcting and combining the data sets to enrich data.

   i. Identifying duplicate observations:

      1. Data should be unique, no two or more rows should be the same in the dataset
      2. Investigate whether the duplicate observation is an incorrect entry or a genuine duplicate

3. If the duplicates are genuine, one of the cases should be dismissed.

ii. Variable type
1. Define all types of variables clearly
2. Re-coding needs to be performed as per the requirement: Any variable that does not match the anticipated data type should be transformed before the data is finalized. For instance, data intended to be numeric but is available as string (text) or vice versa.

iii. Missing values
1. Will Check whether the missing values are expected or not (due to skipping pattern or not)
2. Not expected one – One needs to check with the field team and document the reason.
3. Missing values will re-enter if the reasons for missing values resolved
4. If required, missing values can be imported by using any multivariate imputation method

iv. Post coding
1. Dealing with text entered in the 'other' field in the variable: Here, most of the survey have structured responses, there is always a possibility of responses mentioned under "other" categories. If any alongside response is entered in the "other" category, then the

response will be coded in the pre-coded response category. If a response in the “other” category is repeated frequently (heaping), the response can be coded as a new response category.

b. *Descriptive statistics*

The data quality problems that surfaced during the data collection phase can be found via descriptive analysis. It can be clearly understood by using data visualization and descriptive statistics. The details are given below

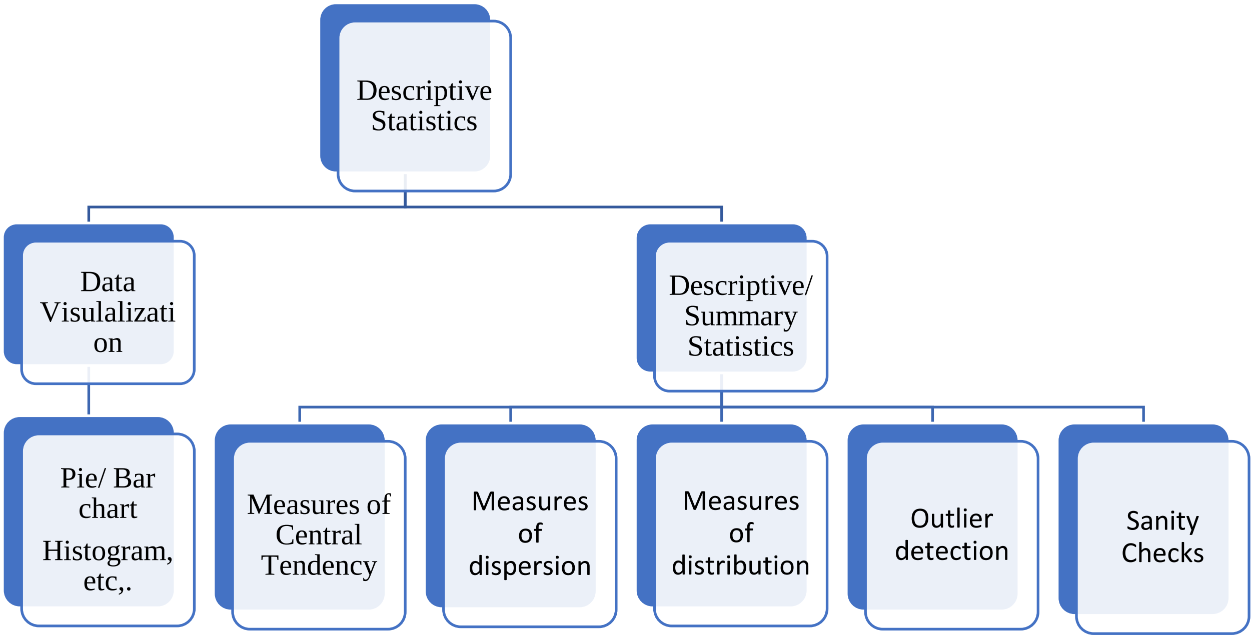

1. Data visualization: Different charts/ graphs, like a pie chart, bar plot, histogram, etc., will be used to do the data visualization.
2. Descriptive/ Summary Statistics:
    a. Measures of central tendency - mean, median and mode

b. Measures of dispersion - variance, standard deviation, range, interquartile range, and coefficient of variation.

c. Measures of distribution - percentiles, quartiles, skewness, and kurtosis

d. Outlier detection

e. In the data entry platform itself will give the range of values (maximum, minimum) to avoid outliers

f. Frequency distribution and/ boxplot to be used (continuous variable)

g. If outlier exists, will re-confirm with the data collection team

h. Will flag the outlier values if not possible to confirm with the collection team

i. Definition of outlier for continuous variable (Using Boxplot)

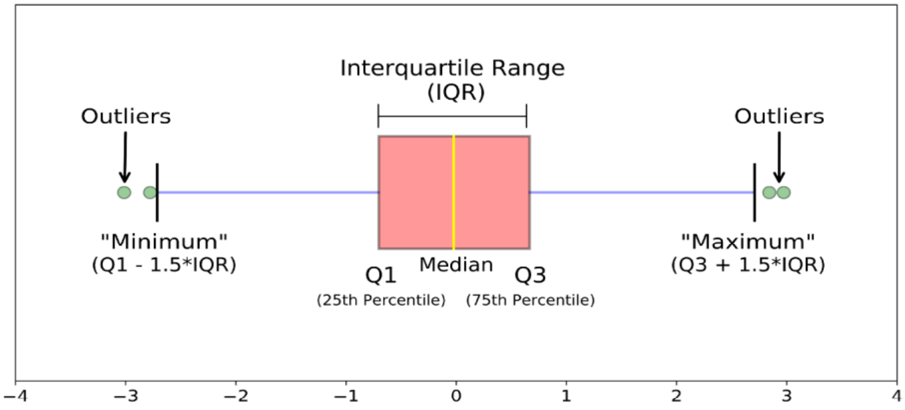

Typical Box Plot

(Source: https://towardsdatascience.com/understanding-boxplots-5e2df7bcbd51)

3. Sanity checks

a. Verify if the data in a variable is consistent with other related variables in the same data

b. **Preparing metadata;** The metadata will prepare and the following details will be included in the metadata.

   i. definition of all variables
   ii. description of all coding values
   iii. date of data creation
   iv. information about data custodian
   v. documentation of specific data issues

Once the raw data & meta da is ready then will go for final statistical analysis. R (V 4.1.2) for Windows software will be used for cleaning and analyzing the data.

Monitoring – Laboratory Practices in the field

1) Remember to chill the cold packs the previous night of the field survey
2) Check the battery/charge of the tablets, Hb meter and the Glucometer, Gazelle, Omron blood pressure, weighing machine, glucometer
3) Availability of required instruments for anthropometric measurements
4) Availability of consumables necessary for sample testing and collection
5) Availability of MOP with the Field technician
6) Identification of suitable place for measurement and testing
7) Checking of name, age, gender, individual Id & sample Id of the participants prior to anthropometric measurement and testing

8) The height of the participants (all age groups) should be measured in barefoot
9) The height of children (<2 years) is to be measured in an infantometer
10) Measurement of WHR should be done by same gender (male for male & female for female)
11) Grip strength measurement should be measured as per the convenient choice of the hand of the participants
12) Blood pressure measurement should be recorded twice and the average to be recorded (Systolic 1+Systolic 2/Diastolic 1+Diastolic 2= Systolic/Diastolic)
13) Before collection of blood samples explain the procedure and get the verbal consent
14) Measure the BP and collect the samples after making the participants sit comfortably
15) Behave politely with the participants and don't pressurize them if they deny giving samples.
16) Perform the hand sanitization for the participants and themselves prior to each capillary blood collection
17) After finger pricking, 1st drop of blood is to be wiped off with cotton and then test the Hb & Glucose
18) Single prick to be done for both the test.
19) If there is insufficient blood from 1st prick, the next finger can be pricked with the same lancet.
20) After completion of the test, put dry cotton/gauge at the pricking site

21) The participants should be requested to discard the cotton in the biohazard bag
22) For the participants to be tested for Sickle cell, the blood from the syringe should be 1st collected in EDTA vacutainer and then in a gel vacutainer.
23) Avoid needle prick injury while transferring the blood in 2 different vacutainers.
24) The Barcode of the vacutainer should be cross-checked before the collection of samples.
25) After each collection store the tubes in an upright position in vaccine carriers
26) While performing the SCD test mark the 3 MCTs first (buffer, dye and sample)
27) While performing the SCD test the proper mixing of blood sample and dye should be done (20 sec using a pipette).
28) Once the result of SCD is displayed, record the screenshot using mobile as well as write the value.
29) The required parameters are to be recorded in the app as well as in the report format to be provided to the participants.
30) Lab tally sheet should be maintained properly for sample collection/no sample.
31) The parameter should be immediately recorded after measurement
32) In the laboratory, serum should be separated by centrifuging the vacutainer at 2800 rpm for 10mins
33) Serum should be separated using the Pasteur pipettes and stored in a cryovial.

34) Each cryovial should be levelled with the barcode and a cello tape over the barcode to avoid erasing due to alcohol and water.
35) The Barcode of the cryovials should be cross-checked before transferring the serum from the vacutainer.
36) Cryovials should be stored in cryo boxes as per the lab tally sheet and kept in cold storage
37) Before transporting the samples to Bhubaneswar, all transport boxes should be packed with sufficient gel packs.
38) All cryo boxes should be labelled properly with Sl no (from-to)
39) Before transporting the samples to RMRC Bhubaneswar the cold chain packaging should be maintained properly and the transport box should be properly labeled and sealed.
40) The lab tally sheet must be provided each time sample is transported to the RMRC laboratory.

Anthropometric Measurement & Biomarker tests

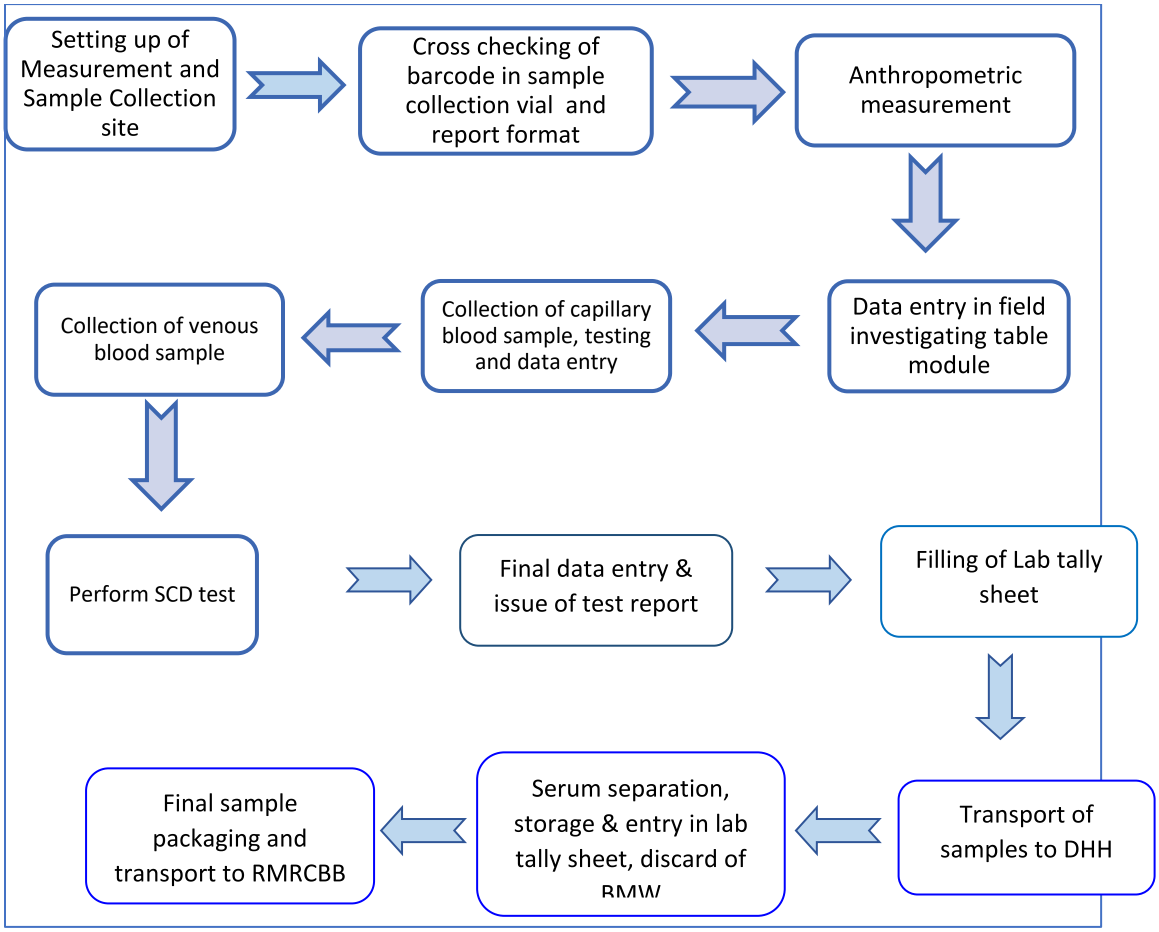

The rationale of the Study

The rationale of this standard operating procedure includes:

1. To demonstrate the procedure of anthropometric measurements of the body which provides valuable assessment of health and nutritional status in children and adults.

2. To demonstrate the step-wise procedure of height and weight measurement, waist-to-hip ratio to assess obesity and grip strength measurement.

3. To instruct the operating process of the equipment i.e. hand dynamometer, OMRON blood pressure measuring instrument to record the grip strength value and systolic and diastolic blood pressure level.

Equipment Required for Anthropometric Measurement

- **Electronic weighing scale:** for weighing children and adults. The scale has a maximum capacity of 180 kg.
- **Stadiometer:** for measuring the height of adults and children above 2 years.
- **Infantometer:** for measuring the length of children under 2 years or less than 85 cm.
- **Measuring Tape:** for measuring waist-to-hip ratio (WHR).
- **Digital Hand Dynamometer:** for measuring isometric grip force.
- **Blood Pressure Recording Unit Digital Automated**: for measuring the systolic and diastolic blood pressure.

Setting up of Measurement and Sample Collection site

An ideal place will be identified in the surveyed village where the anthropometric instruments will be set up and clinical sample collections will be performed. The sites like

- Anganwadi centre
- School
- The front area of any participant's house (with the consent from the house owner)
- Any shaded place with sufficient light

The portable table and chair will be installed in the identified place where all the field laboratory set-up will be done.

Roles and responsibilities

Laboratory technician 1/Laboratory technician 2/Field investigator: To coordinate and record the anthropometric measurement.

Checklist before proceeding with anthropometric measurement

- **The Layout of the Procedures**: Each step of the measurement procedures is

directed at specific participants. The measurer (LT or FI) have to cross-check the individual Id and sample Id in the Field Investigation Table module and in the report format prior to the measurement.

- **Two persons required**: Two people (parents/guardians) are required to measure a child's height or length. The measurer holds the child and takes the measurements. The child's parent/guardian will help in measurement. Measurer alone can take the weight of a child and record the results.
- **Age Assessment:** Before you measure, determine the child's age. If the child is less than two years, measure length (that is, with the child lying). If the child is two years of age or older, measure height (that is, with the child standing). If accurate age is not possible to obtain, measure length if the child is less than 85cm. Measure height if the child is equal to or greater than 85cm.
- **Weigh and Measure One Child at a Time:** If there is more than one eligible child in a household, complete the weighing and measuring of one child at a time. Then proceed with the next eligible child.
- **Control the Child:** When you weigh and measure, you must control the child. The strength and mobility of even very young children should not be underestimated. Be firm yet gentle with children. Your own sense of calm and self-confidence will be felt by the parent and the child.
- When a child has contact with any measuring equipment, i.e., on an infantometer, you must hold and control the child so the child will not trip or fall. Never leave a child alone with a piece of equipment.
- **Coping with stress:** Since weighing and measuring requires touching and handling children, normal stress levels for this type of survey work are higher than for surveys where only verbal information is collected. Explain the weighing and measuring procedures to the mother, father, or other responsible adult and to a limited extent, the child, to help minimize possible resistance, fears or discomfort they may feel.

The criteria for non measurement

- The parent/responsible adult refuse.
- The child is too sick or distressed.
- The child is physically deformed which will interfere with or give an incorrect measurement.

Steps For Measuring Weight

- Perform the adult weight measurements according to the instructions below.

Weight Measurement of Adult and children

- *Preparing the adult and children to take their weight*
  Show the scale to the adult and explain that you will weigh her/him and the children on thescale.
- *Preparing the Scale*
  Before the measurement starts place the weighing scale on a flat surface. Uneven surfaces or vibration may cause the scale to malfunction.

The machine operation and steps involved in weight measurement is mentioned below as per Manufacturer's instruction:

1. SPECIFICATION :

1).Max Capacity: 180kg / 396[illegible]

2).Auto on and auto off

Remark: The scale would be switched on when w[illegible] of 5 kg or more is placed on it.

3). Measurement: Body Weight, BMI, BFR, Muscle, Moisture, Bone mass, BMR, Visceral fat, Protein rate, Body age, Standard weight, Weight control,Body fat, Weight without fat,Muscle weight,The amount of protein,The degree of obesity

4). High precision strain gauge sensor system and High Performance CPU, make sure of good accuracy.

5).Safety tempered glass platform

6).Low battery and overload indication

Battery

Power: AAA battery

For the first time use, please open the battery cover to insert the battery according to the polarity. If the scale fails to operate, remove the battery completely and reinsert it.

2. Operation

A.How to measure Body weight

When you stand on the scale without connecting to Bluetooth,the scale will only measure your body weight. Please follow below steps:

1)1)Please use scale on the hard & flat surface.don't use on the carpet or soft surface.

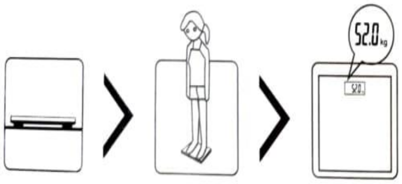

2)Carefully step on the platform of the scale. Stand as stably as possible until the weight reading is shown and locked on the LCD

3)The scale will be automatically off if no further operation

B. How to connect with Bluetooth for Body Weight, BMI, BFR, Muscle, Moisture, Bone mass, BMR, Visceral fat, Protein rate, Body age, Standard weight, Weight control,Body fat,Weight without fat,Muscle weight,The amount of protein &The degree of obesity and so on.

a.Download App

APP name: AISlim

Remark: Our APP is available for IOS and android device with IOS version 8.0 or Android version 5.0 and Bluetooth 4.0(Or higher version)

Download on the App Store

Google play

Download APP "AISlim" on Apple store or Google play online store

b. Registered user

1).Start <AISlim>

2).Press the "Allow" bottom to turn on Bluetooth

3).The current system requires location permissions: press the "got it" bottom to set up "location access"

4).Register a account or use the third-party login

5)Edit User and complete user Profile

3. Error icon of the scale

Overload or Measure in error operations: the LCD will indicates "Err", when scale is over max capacity .Please remove the weight to avoid any damage.

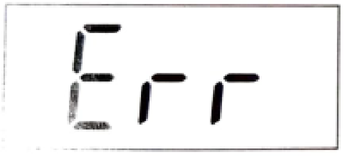

Low battery:the LCD w[illegible]"Lo", Please open the battery cover and replace the new battery with corre[illegible]egative.

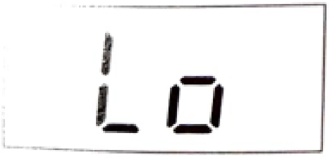

Weighing Infants or Children Who Must be Held by an Adult While on the Scale

- For weighing the infant or the child on the scale, first, the weight of the adult's weight will be measured who is going to carry the infant or the child.
- Next the adult will be instructed to get down from the weighing machine so that the machine reading becomes 0.00 again.
- The adult will be instructed to carry the infant or child (to be measured) and the weight will be measured again in the weighing machine.
- For calculating the infant or child's weight the adult measured weight will be deducted from the second measured weight (infant or child plus adult) and the infant weight will be recorded.

Steps For Measuring Height

Measuring an adult's height

- Place the stadiometer on a hard, flat surface against a wall, table, tree or staircase. Make sure the stadiometer is stable. Many walls and floors are not at perfect right angles; if necessary, place small rocks underneath the height

board to stabilize it during the measurement.

- Ask the person to take off his/her shoes and ask him/her to unbraid or push aside any hair that would interfere with the height measurement. Ask the person to stand on the base of the height stadiometer and to face forward.

- Determine if the person's feet should be against or away from the back of the height board by observing the imaginary line drawn from the tip of the shoulder to the heel, which is called the "mid-maxillary line" (Arrow3). This line should be perpendicular (i.e. 90°) to the base of the stadiometer where the person is standing. Note that with almost all adults you will have to move the person's feet away from the back of the height board to put the min the proper position; (Arrow 4).

- Place the knees and feet in the correct position, with knees and feet either together or apart. There are three possible positions for the knees and feet:
    - Knees together and feet together
    - Knees together and feet apart
    - Knees apart and feet together

Whichever touches first!

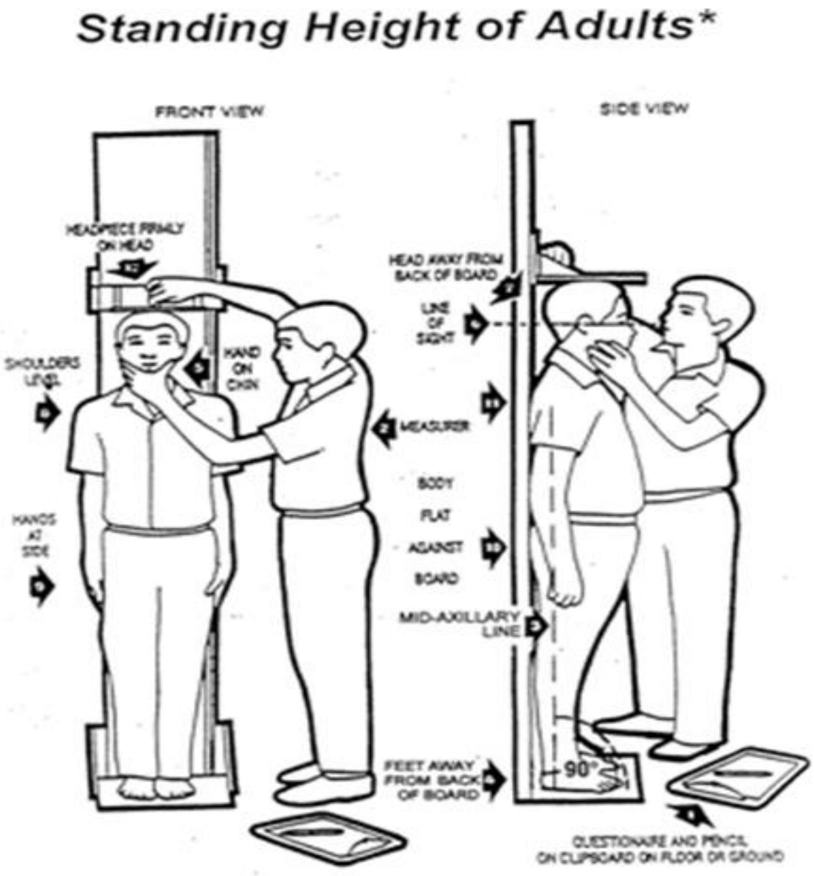

Adult Height Measurement

(Figure courtesy: NFHS -4 manual)

- Ask the person to look straight ahead. Cup the respondent's chin between the thumb and index finger of your left hand and gradually close your hand (Arrow 5). Position the person's head so that the line of sight is parallel to the ground (Arrow 6). Note that with most adults, the back of the head will not touch the back of the stadiometer—there will be a space between the back of the person's head and the back of the stadiometer (Arrow 7). After you have placed the person's head in the proper position, release your hand from the person's chin and ask him/her to hold his/her head in the position you have just placed it in.
- Make sure the person's shoulders are level (Arrow 8), the hands are at the person's side (Arrow 9), and at least the buttocks touches the

back of the stadiometer). Note that with most adults, only the buttocks and perhaps the shoulder blades, will touch the back of the stadiometer (Arrows 10 & 11).

- Check the position of the person (Arrows 1-11). Repeat any steps as necessary.
- When the person’s position is correct, lower the head piece on top of the head (Arrow 12) Making sure to push through the person’s hair. **Read and call out the measurement to the nearest 0.1 cm.** Remove the headpiece from the person’s head, and escort the person off the height board.
- Immediately record the measurement on the questionnaire. Record an adult’s **height** measurements in **Questionnaire**. If the adult’s height was not measured, record the appropriate code in **Questionnaire**.
- Check the recorded measurement on the questionnaire for accuracy and legibility. Correct any errors.

Measuring a Child’s Height: Standing Up

- Ask the parent to take off the child’s shoes and to unbraid or push aside any hair that would interfere with the height measurement. Ask the parent to bring the child to the stadiometer and to kneel in front of the child so that the child will look forward at the parent.

- Kneel on the left of the child (Arrow3).

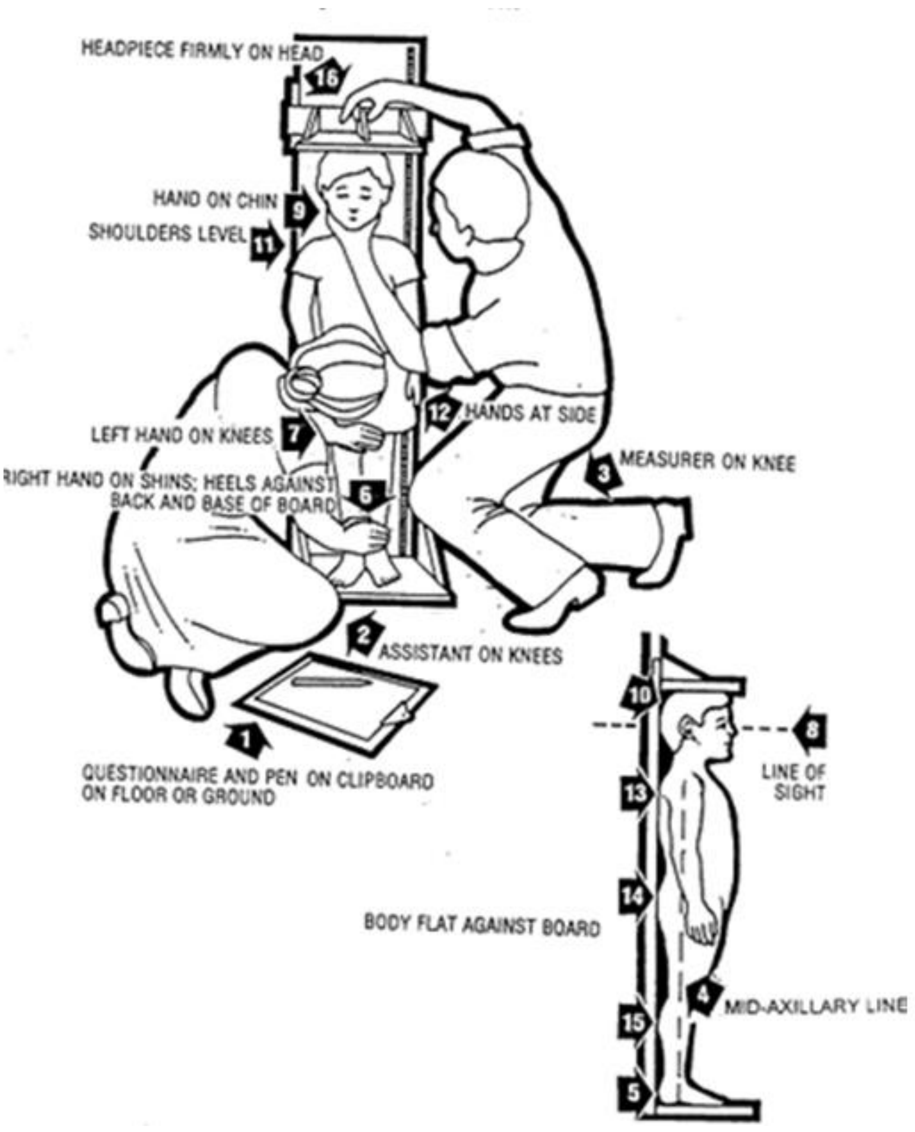

Child Height Measurement

(Figure courtesy: NFHS -4 manual)

- Place the child’s knees and feet in the correct position, with knees and feet either together or apart. There are three possible positions for the knees and feet:
  - Knees together and feet together
  - Knees together rand feet apart whichever touches first!
  - Knees apart and feet together

- Determine if the child’s feet should be against or away from the back of the stadiometer by observing the imaginary line drawn from the tip of the shoulder to the heel, which is called the “mid-axillary line” (Arrow 4). This line should be perpendicular (i.e.90°) to the base of the stadiometer where the child is standing.
  (You may have to move the child’s feet away from the back of the stadiometer to put them in the proper position). Note that with most preschool-age children who are not heavy or obese, the heels will probably touch the back of the height board (Arrow 5).

- With your thumbs against the index finger of each hand, place your right hand on the child’s shins (Arrow 6) and your left hand on the child’s knees (Arrow 7). Do not wrap your hands around the knees or feet (ankles) or squeeze them together. Make sure the child’s legs are straight.
- Ask the child to look straight ahead at the parent if she is kneeling in front of the child. Make sure the child’s line of sight is parallel to the ground (Arrow 8). Place the thumb and index finger of your left hand, one finger on each side of the child’s chin, and gradually close your hand (Arrow 9). Note that with most preschool-age children who are not heavy or obese, the back of the head will touch the back of the stadiometer (Arrow 10); however, if the child is heavy or obese, there will be a space between the back of the child’s head andthe back of the stadiometer. Make sure the child’s shoulders are level (Arrow 11), the hands are at the child’s side (Arrow 12), and at least the child’s buttocks touch the back of the stadiometer. Note that with most preschool-age children who are not heavy or obese, the back of the head, the shoulder blades, the buttocks, the calves and heels will touch the back of the stadiometer (Arrows10,13,14,15&5).
- Check the position of the child (Arrows 1-15). Repeat any steps as necessary.
- When the child’s position is correct, lower the headpiece on top of the child’s head (Arrow 16) making sure to push through the child’s hair. Read and call out the measurement.
- Immediately record the **height** measurementson the questionnaire.
- Check the recorded measurement on the questionnaire for accuracy and

legibility.

Measuring a Child's Length: Lying Down

This method is used for the children who are less than two years old. The length measured for children in this way is less than 85 centimeters.

- Place the infantometer on a hard, flat surface, such as the ground, floor or a solid table to ensure the infantometer is stable.Place the biomarker questionnaire (tablet) on the ground, floor or table (Arrow 1) and kneel behind the fixed head end of the infantometer if it is on the ground or floor (Arrow 2).
- Kneel at the right side of the child (at the child's feet) so that you can move the sliding foot piece with your right hand (Arrow3).
- With the help of the parent, gently lower the child on to the infantometer, making sure the measurer supports the child at the trunk of the body while the assistant supports the child's head.

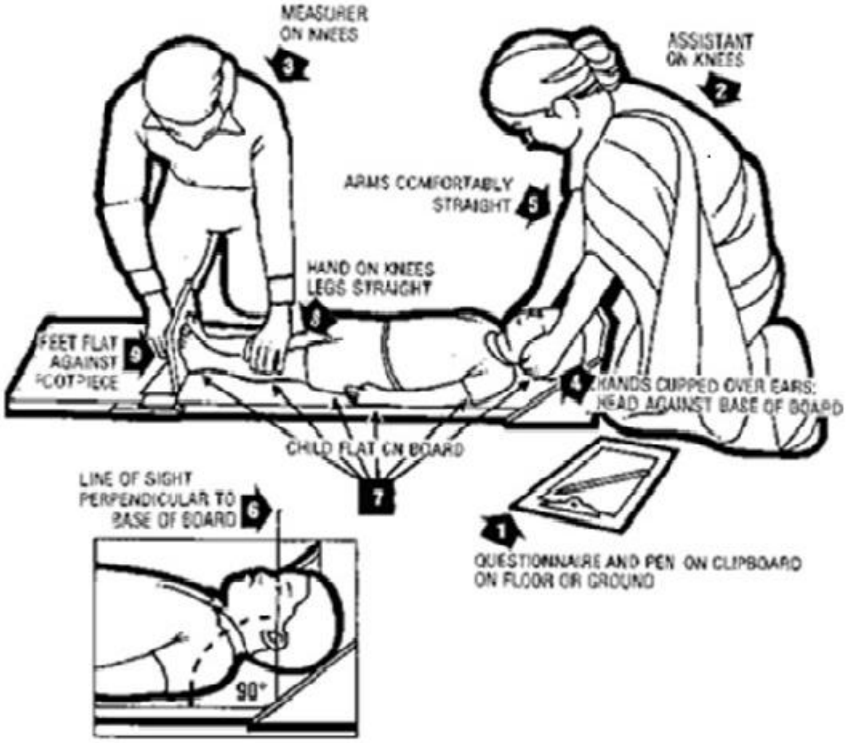

Child Length Measurement

(Figure courtesy: NFHS -4 manual)

Measurement of Waist –to-Hip Ratio (WHR)

An important issue in using and interpreting waist circumference or waist–hip ratio is the protocol used to obtain the measurements. Also important is the extent to which the measurement protocol varies across studies, and the potential for standardizing these measurements within a study or survey, when taken by different people (2).

- ➢ **Placement, tightness and type of measuring tape**
- **Placement of tape**

Waist circumference

The WHO STEPS protocol for measuring waist circumference instructs that the measurement be made at the approximate midpoint between the lower margin of the last palpable rib and the top of the iliac crest.
Abdominal obesity is further defined as waist–hip ratio above 0.90 for males and above 0.85 for females, or a BMI above 30.0(3).

Hip circumference
The protocols for hip circumference measurement should be taken around the widest portion of the buttocks. This include following steps:

Tightness and type of tape
The accuracy of waist and hip circumference measurements depends on the tightness of the measuring tape, and on its correct positioning (i.e. parallel to the floor at the level at which the measurement is made). For both waist and hip, the tape should be snug around the body, but not pulled so tight that it is constricting.

Abdominal tension at the point of measurement
The tension of the abdominal wall influences the accuracy of the waist circumference measurement. Lowering the tension of the abdominal wall increases waist circumference, whereas increasing the tension (by sucking in) reduces waist circumference. Many individuals unconsciously react to waist measurements by sucking in the abdominal wall; hence, a relaxed posture is best for taking waist measurements. It is recommended to advise the subject to relax and take a few deep, natural breaths before the actual measurement is made, to minimize the inward pull of the abdominal contents during the waist measurement.

Measurement protocol

- Measure the *waist circumference* at the end of several consecutive natural breaths, at a level parallel to the floor, midpoint between the top of the iliac crest and the lower margin of the last palpable rib in the mid axillary line.
- Measure the *hip circumference* at a level parallel to the floor, at the largest

circumference of the buttocks.

- Make both measurements with a stretch-resistant tape that is wrapped snugly around the subject, but not to the point that the tape is constricting. Keep the tape level and parallel to the floor at the point of measurement.
- Ensure that the subject is standing upright during the measurement, with arms relaxed at the side, feet evenly spread apart and body weight evenly distributed.

GRIP STRENGTH MEASUREMENT

Hand-held dynamometers may also be used to test the strength of individual muscles or muscle groups. A dynamometer is a device that can measure force.

Digital Hand Dynamometer Operating Protocol and Procedure

- Ask the participant to sit in the chair and to hold the dynamometer in one hand (right or left hand whichever the participant uses most frequent to carry out work.
- The hand should be stretched parallel along with the floor.
- Now instruct the participant to pull the handle as strong as he/she can for 10 seconds and then to release it.
- Record the reading from the dynamometer display.

Follow the manufacturer's instruction:

- **OUTLINE**

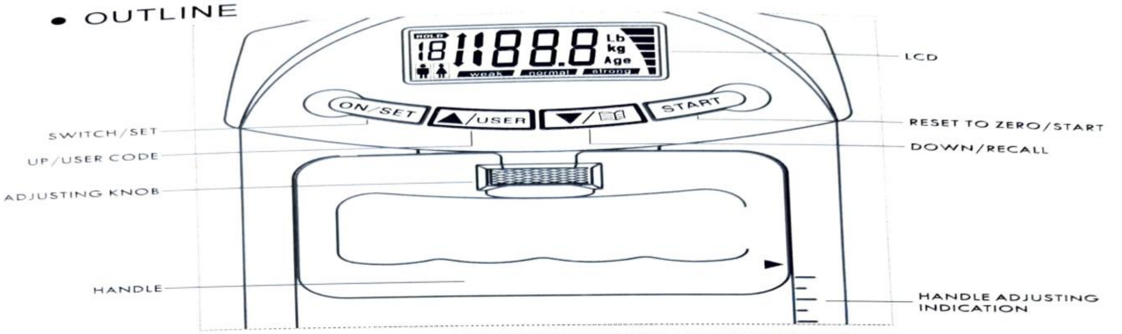

- **TO REPLACE BATTERIES**

1. Open battery case cover at the back of dynamometer;
2. Take out used batteries;
3. Install new batteries by putting one side of the batteries beneath battery baffle and pressing down the other side. Please take note to polarity.

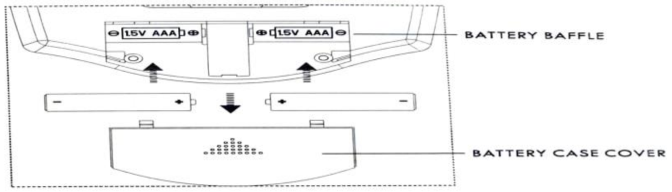

- **LCD ICONS DESCRIPTION**

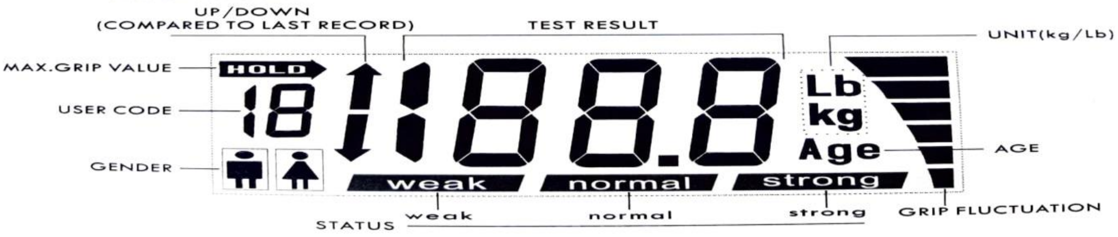

- **PRELIMINARY ADJUSTMENT**

Before using, you can adjust up or down the handle by turning the adjusting knob right or left to get an ideal grasp.

eg: User with big hand can adjust the handle upwards by turning right the adjusting knob.

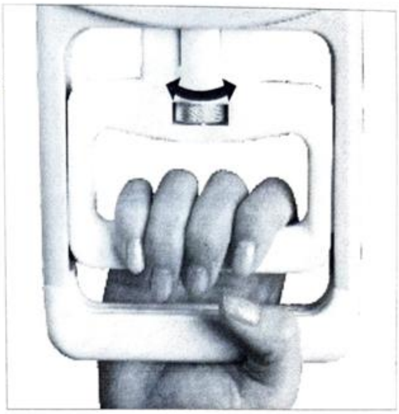

- APPENDIX: PHYSICAL STATUS ACCORDING TO THE TEST RESULT GIVEN BY THE DYNAMOMETE[illegible]

AGE	MALE			FEMALE		
	Weak	Normal	Strong	Weak	Normal	Strong
10—11	<12.6	12.6-22.4	>22.4	<11.8	11.8-21.6	>21.6
12—13	<19.4	19.4-31.2	>31.2	<14.6	14.6-24.4	>24.4
14—15	<28.5	28.5-44.3	>44.3	<15.5	15.5-27.3	>27.3
16—17	<32.6	32.6-52.4	>52.4	<17.2	17.2-29.0	>29.0
18—19	<35.7	35.7-55.5	>55.5	<19.2	19.2-31.0	>31.0
20—24	<36.8	36.8-56.6	>56.6	<21.5	21.5-35.3	>35.3
25—29	<37.7	37.7-57.5	>57.5	<25.6	25.6-41.4	>41.4
30—34	<36.0	36.0-55.8	>55.8	<21.5	21.5-35.3	>35.3
35—39	<35.8	35.8-55.6	>55.6	<20.3	20.3-34.1	>34.1
40—44	<35.5	35.5-55.3	>55.3	<18.9	18.9-32.7	>32.7
45—49	<34.7	34.7-54.5	>54.5	<18.6	18.6-32.4	>32.4
50—54	<32.9	32.9-50.7	>50.7	<18.1	18.1-31.9	>31.9
55—59	<30.7	30.7-48.5	>48.5	<17.7	17.7-31.5	>31.5
60—64	<30.2	30.2-48.0	>48.0	<17.2	17.2-31.0	>31.0
65—69	<28.2	28.2-44.0	>44.0	<15.4	15.4-27.2	>27.2
70—99	<21.3	21.3-35.1	>35.1	<14.7	14.7-24.5	>24.5

BLOOD PRESSURE MEASUREMENT

High blood pressure can damage arteries by making them less elastic, which decreases the flow of blood and oxygen to heart and leads to heart disease. In addition, decreased blood flow to the heart can cause chest pain, also called angina. Heart attack occurs when the blood supply to the heart is blocked and heart muscle begins to die without enough oxygen. The normal blood pressure range is between 115/75 and 120/80 mmHg. There are three levels of high blood pressure.

- ***Prehypertension***: The systolic blood pressure ranges between 120 and 139 mmHg or the diastolic blood pressure is between 80 and 89 mmHg.
- ***First stage:*** The systolic blood pressure ranges between 140 and 159 mmHg or the diastolic blood pressure is between 90 and 99 mmHg.

- ***Second stage:*** The systolic blood pressure is 160 mmHg or higher, or the diastolic blood pressure is 100 mmHg or higher.

Preliminary Steps In Taking Blood Pressure Measurements From Adults

- Before starting the blood pressure measurements, the respondent (The participant whose blood pressure will be measured) should have been sitting quietly for at least 5 minutes.

- Be sure that the respondent does not smoke or drink coffee or tea during the measurements since smoking or drinking coffee or tea can affect blood pressure. If the respondent consumed any alcohol, coffee or tea, or smoked cigarettes before the examination, record this in Questionnaire.

- If the respondent indicates any reason why the blood pressure procedure should not be done on the left arm, use the right. If there is a problem with both arms, do not take the blood pressure and note this in Questionnaire. Observe the respondent's arm while talking to him/her. If you observe any rashes, small gauze / adhesive dressings, casts, with ere arms, puffiness, tubes, open sores, haematomas or wounds on both arms, do not take the blood pressure.

Positioning the Respondent for Blood Pressure Measurement

- The respondent should be seated at a table in a relaxed, but not slouched, position with feet flat on the floor. The outer jacket or sweater should be removed and the sleeve should be rolled loosely up to the shoulder, ensuring that two fingers can be placed under the sleeve without difficulty. The respondent's left arm should be placed on the table, slightly flexed with the palm upward.

- The respondent's arm should be positioned so that it is resting on the table at heart level. The heart level is halfway between the shoulders and the waist. The respondent's elbow must be no lower

than the lowest rib and must not be raised as high as the shoulder.

- If the respondent is tall, it may be necessary to support the arm higher than a standard desk or tabletop. Place the tall respondent's forearm on a pillow or a large book to raise the arm to heart level.

- For smaller or shorter adults, place a cushion or large book on the chair so that the arm is at heart level when the arm is resting on the desk or tabletop. Place a box or large book under the respondent's feet if the feet do not rest flat on the floor.

- The health investigator should be seated facing and slightly to the left of the respondent, permitting easy access to the respondent's arm. The measuring equipment will be positioned so that the tube to the manometer is away from the respondent's body while the tube to the inflation bulb is closer to the body.

How To Seat The Respondent Correctly When Taking a Measurement

- Make sure that all the components of the OMRON BP monitor are present and that it is kept on the left side of the respondent if the blood pressure is going to measure from the left hand.
- Correct posture during measurement is necessary to get accurate results. Examples of incorrect posture:
    - Arched back (leaning forwards)
    - Sitting cross-legged
- If the arm cuff is at a lower position than the respondent's heart, use cushions or a pillow to adjust the height of the respondent's arm.
- Insert the air plug into the air jack (on the left side of the device). The cuff must be fully deflated when it is inserted into the air jack.
- Have the respondent sit in a chair with her/his feet flat on the floor and place her/his arm on a table so that the cuff will be at the same level as her/his heart.
- Have the respondent sit in a chair with her/his feet flat on the floor and place her/his arm on a table so that the cuff will be at the same level as her/his heart.
- Hold the grip on the cuff securely with your hand.

- Turn the palm of the respondent's hand upward.
- Apply the cuff to the respondent's upper arm so that the air tube is centred on the middle of the respondent's inner arm and points down the inside of the arm. The air tube should run down the inside of the respondent's forearm and be in line with her/his middle finger.

- The bottom of the cuff should be approximately 1 to 2 cm above the elbow.

- Whenthecuffispositionedcorrectly, close the fabric and fasten firmly.

- Makecertainthecufffitssnugly around the arm.
- The cuff should make good contact with the respondent's skin. You should be able to fit your index finger between the cuff and the respondent's arm easily, so you can pull the cuff off and on.

Measurements on the right arm

Note the following points when applying the cuff to your right arm. Apply the cuff so that the air tube is at the side of the respondent's elbow.

- Be careful not to rest your arm on the air tube, or otherwise restrict the flow of air to the cuff.
- Applythearmcuffsothatnopartofthecuffispositionedovertheelbowjoint.The cuffs should be 1 to 2 cm above the elbow.

Taking a Blood Pressure Reading

For OTFHS, the **OMRON BP Monitor** will be used for respondents with small, medium and large arm circumference. Measure the respondent's arm circumference to enable you to select the correct cuffsize for use. Press the **START** button and ask the respondent to remain still; the cuff will start to inflate automatically. As the cuff begins to inflate, the monitor automatically determines the ideal inflation level. The respondent should remain still and not talk until the measurement is completed.

Note: Do not inflate the cuff if it is not wrapped around the respondent's arm.

Inflation stops automatically and measurement is started. As the cuff slowly deflates, decreasing numbers appear on the display and the Heartbeat display flashes at every heartbeat. In rare circumstances, the monitor might re-inflate the cuff to continue with the measurement.

- **START TO INFLATE DEFLATE THE END COMPLETE**

- When the measurement is complete, the arm cuff completely deflates and the blood pressure [and pulse rate] readings are displayed. Record the readings in the appropriate boxes.

- Remove the arm cuff from the respondent’s arm.

- After the measurement is completed, you can either press the O/I start button to turn the monitor off or it will shut off automatically after 5 minutes.

  - **Blood Pressure Monitoring Repetition**
  - **For each participant, blood pressure will be measured two times with a gap of 5 minutes. The average of both systolic and diastolic readings will be recorded.**

  - **Safety and precaution**:

  - Do not use the instrument close to strong electromagnetic fields such as **mobile telephones or radio installations.**
  - If the instrument is not going to be used for a prolonged period, the batteries should be removed.
  - Do not open the instrument.

WHAT DISPLAY SYMBOLS AND ERROR MESSAGES MEAN

Error Display	Cause	Remedy
EE	Cuff is under inflated.	Carefully read and repeat the steps listed under taking BP measurement
E	Movement during measurement.	Repeat measurement. Remain still and do not talk during measurement. Refer to section 3.3.
	Air plug disconnected.	Insert the air plug securely.
	Arm cuff not applied correctly.	Apply the arm cuff correctly.
	Clothing is interfering with the arm cuff.	Remove any clothing interfering with the arm cuff.
	Air is leaking from the arm cuff.	Replace cuff with new one.
E E	The arm cuff was inflated above 299 mmHg when inflating the cuff manually.	Do not inflate the arm cuff above 299 mmHg.
Er 25	Device error.	Contact your OMRON retail outlet or distributor.

Note:
The irregular heartbeat symbol ([illegible]) may also be displayed with error messages.

Troubleshooting

Error Display	Cause	Remedy
	Irregular or weak pulses are detected.	Remove the arm cuff. Wait 2-3 minutes and then take another measurement. If this error continues to appear, contact your doctor.
	Movement during measurement.	Carefully repeat the steps measurement.
	Cuff is not applied correctly.	Apply the arm cuff correctly.
Blink	The batteries are low.	You should replace them with new ones ahead of time.
Lit	The batteries are exhausted.	You should replace them with new ones at once.

Error Display	Cause	Remedy
	Irregular or weak pulses are detected.	Remove the arm cuff. Wait 2-3 minutes and then take another measurement. If this error continues to appear, contact your doctor.
	Movement during measurement.	Carefully repeat the steps measurement.
	Cuff is not applied correctly.	Apply the arm cuff correctly.
Blink	The batteries are low.	You should replace them with new ones ahead of time.
Lit	The batteries are exhausted.	You should replace them with new ones at once.

Automatic Blood Pressure Monitor

Model HEM-8712
Instruction Manual

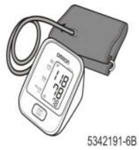

5342191-6B

Introduction

Thank you for purchasing the OMRON HEM-8712 Automatic Blood Pressure Monitor.

The OMRON HEM-8712 is a compact, fully automatic blood pressure monitor, operating on the oscillometric principle. It measures your blood pressure and pulse rate simply and quickly. For comfortable controlled inflation without the need of pressure pre-setting or re-inflation the device uses its advanced "IntelliSense" technology.

Intended Use
This product is designed to measure the blood pressure and pulse rate of people within the range of the designated arm cuff, following the instructions in this instruction manual.
It is mainly designed for general household use. Please read the Important Safety Information in this instruction manual before using the unit.

Please read this instruction manual thoroughly before using the unit.
Please keep for future reference.
For specific information about your own blood pressure, CONSULT YOUR DOCTOR.

Important Safety Information

Consult your doctor prior to using in pregnancy or if diagnosed with arrhythmia or arteriosclerosis.
Please read this section carefully before using the unit.

⚠**Warning:** Indicates a potentially hazardous situation which, if not avoided, could result in death or serious injury.

(General Usage)
- Do not use the device on the injured arm or the arm under medical treatment.
- Do not wrap the arm cuff on the arm while being on an intravenous drip or blood transfusion.

(AC Adapter (optional) Usage)
- Do not use the AC adapter if the unit or the power cord is damaged. Turn off the power and unplug the power cord immediately.
- Plug the AC adapter into the appropriate voltage outlet. Do not use a multiple-tap.
- Never plug in or unplug the power cord from the electric outlet with wet hands.

⚠**Caution:** Indicates a potentially hazardous situation which, if not avoided, may result in minor or moderate injury to the user or patient or damage to the equipment or other property.

(General Usage)
- Always consult your doctor. Self-diagnosis of measurement results and self-treatment are dangerous.
- People with severe blood flow problems, or blood disorders, should consult a doctor before using the unit, as cuff inflation can cause internal bleeding.
- If there are any abnormalities during the measurement, remove the arm cuff.
- Do not use this device on infants or persons who cannot express their intentions.
- Do not inflate the arm cuff more than necessary.
- Do not use the unit for any purpose other than measuring blood pressure.
- Use only the approved arm cuff for this unit. Use of other arm cuffs may result in incorrect measurement results.
- Do not use a mobile phone or other devices that emit electromagnetic fields, near the unit. This may result in incorrect operation of the unit.
- Do not disassemble the unit or arm cuff.

(Battery Usage)
- Do not insert the batteries with their polarities incorrectly aligned.
- Use only four "AA" alkaline or manganese batteries with this unit. Do not use other types of batteries. Do not use new and used batteries together.
- Remove the batteries if the unit will not be used for 3 months or more.

(AC Adapter (optional) Usage)
- Fully insert the power plug.
- When disconnecting the power plug, do not pull the power cord. Be sure to hold the power plug.
- When handling the power cord, observe the following:
  - Do not damage. Do not break it.
  - Do not tamper with it. Do not forcibly bend or pull.
  - Do not twist. Do not bundle during use.
  - Do not pinch. Do not place under heavy objects.
- Wipe the dust off from the power plug.
- Disconnect the power plug if the product will not be used for a long period of time.
- Disconnect the power plug before starting maintenance.
- Use only the original AC adapter designed for this unit. Use of unsupported adapters may damage and/or may be hazardous to the unit.

General Precautions
- Do not forcibly bend the arm cuff or bend the air tube excessively.
- To unplug the air plug, pull on the air plug at the connection with the main unit, not the tube itself.
- Do not apply strong shocks and vibrations to or drop the unit and arm cuff.
- Do not inflate the arm cuff when it is not wrapped around your arm.
- Read and follow the "Important information regarding Electro Magnetic Compatibility (EMC)" in the Technical Data Section.
- Read and follow the "Correct Disposal of This Product" in the Technical Data Section when disposing of the device and any used accessories or optional parts.

1. Overview

Main unit:

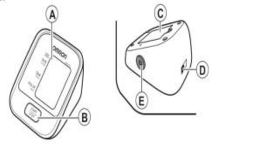

A. Display
B. START/STOP button
C. Battery compartment
D. AC adapter jack (for optional AC adapter)
E. Air jack

Arm cuff:

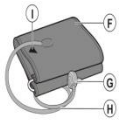

F. Arm cuff (Arm circumference 22 - 32 cm)
G. Air plug
H. Air tube
I. Marker

Display:

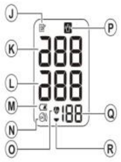

J. Memory symbol
K. Systolic blood pressure
L. Diastolic blood pressure
M. Low battery symbol
N. Cuff wrapping guide
O. Heartbeat symbol
  1. Flashes during measurement.
  2. If flashing after measurement completed or when viewing results stored in the memory, indicates blood pressure out of recommended range*.

P. Movement error symbol
Q. Pulse display
R. Deflation symbol

* Note: If your systolic or diastolic pressure is outside the standard range (above 135/85 mmHg) the Heartbeat symbol (♥) will blink. Please refer to Section 3.3.

2. Preparation

2.1 Installing/Replacing the Batteries

1. Remove the battery cover.
2. Insert four "AA" batteries as indicated in the battery compartment and then replace the battery cover.

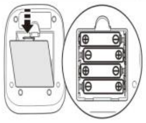

Notes:
- If the low battery symbol (▭) appears on the display, turn the monitor off then replace all batteries at the same time.
- The measurement values continue to be stored in memory even after the batteries are replaced.
- The supplied batteries may have a shorter life.

Disposal of used batteries should be carried out in accordance with the national regulations for the disposal of batteries.

3. Using the Unit

3.1 Applying the Arm Cuff

Remove tight-fitting clothing or tight rolled up sleeve from your upper arm. Do not place the arm cuff over thick clothes.

1. Insert the air plug into the air jack securely.

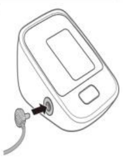

2. Put your arm through the cuff loop.

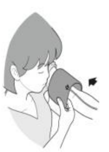

The bottom edge of the arm cuff should be 1 to 2 cm above the elbow. Marker (arrow under the air tube) is centred on the middle of your inner arm.

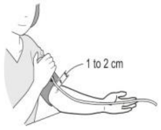

3. Close the fabric fastener FIRMLY.

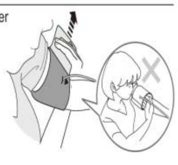

Notes:
- When you take a measurement on the right arm, the air tube will be at the side of your elbow. Be careful not to rest your arm on the air tube.

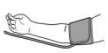

- The blood pressure can differ between the right arm and the left arm, and therefore also the measured blood pressure values can be different. OMRON recommends to always use the same arm for measurement. If the values between both arms differ substantially, please check with your doctor which arm to use for your measurement.

3.2 How to Sit Correctly

To take a measurement, you need to be relaxed and comfortably seated, under comfortable room temperature. No bathing, drinking alcohol or caffeine, smoking, exercising or eating 30 minutes before taking a measurement.

- Sit on a chair with your feet flat on the floor.
- Sit upright with your back straight.
- The arm cuff should be at the same level as your heart.

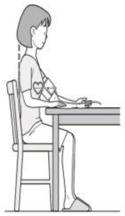

3.3 Taking a Reading

Notes:
- To cancel a measurement, press the START/STOP button to release the air in the arm cuff.
- Remain still while taking a measurement.

1. Press the START/STOP button.
The arm cuff will start to inflate automatically.

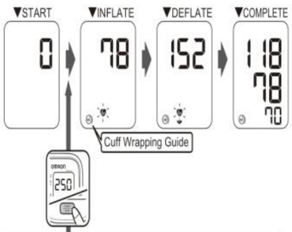

If your systolic pressure is more than 210 mmHg
After the arm cuff starts to inflate, press and hold the START/STOP button until the monitor inflates 30 to 40 mmHg higher than your expected systolic pressure.

Notes:
- The monitor will not inflate above 299 mmHg.
- Do not apply more pressure than necessary.

Note: Wait 2-3 minutes before taking another blood pressure measurement. Waiting between readings allows the arteries to return to the condition prior to taking the blood pressure measurement.

⚠ **Always consult your doctor. Self-diagnosis of measurement results and self-treatment are dangerous.**

Cuff Wrapping Guide:
The Cuff Wrapping Guide is a unique feature that indicates if the cuff is not wrapped tightly enough around the arm. Even when the ◯ appears on the display, a blood pressure reading will be taken.

Note: This reading is **NOT** reliable due to the incorrect wrapping of the cuff. Please wrap the cuff again, taking care to wrap it correctly and take the measurement again. When the ◯ is displayed, the cuff is correctly wrapped tightly enough on the arm and the reading is accurate and reliable.

2. Remove the arm cuff.
3. Press the START/STOP button to turn the monitor off.
The monitor automatically stores the measurement in its memory.
It will automatically turn off after 2 minutes.

Important:
- If your systolic or diastolic pressure is outside the standard range, the heartbeat symbol will blink when the measurement result is displayed. Recent research suggests that the following values can be used as a guide to high blood pressure for measurements taken at home.

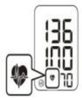

Systolic Blood Pressure	Above 135 mmHg
Diastolic Blood Pressure	Above 85 mmHg

This criteria is for home blood pressure measurement.

- If you move during measurement, the movement error symbol (▣) will appear on the display. Keep still and repeat the measurement.

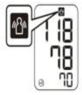

3.4 Using the Memory Function

The monitor automatically stores the last measurement values (blood pressure and pulse rate).

To View the Readings Stored in Memory

1. Press and hold the START/STOP button for more than 5 seconds.
The last measurement value is displayed along with the memory symbol.

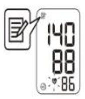

Notes:
- If your systolic or diastolic pressure is outside the standard range, the heartbeat symbol will blink when the measurement result is displayed.
- The cuff wrapping guide result appears on the display with the measurement values.
- If there are no measurements results stored in the memory, the screen to the right is displayed.

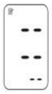

2. Press the START/STOP button to turn the monitor off.
It will automatically turn off after 2 minutes.

To Delete the Values Stored in Memory

Press and hold the START/STOP button for more than 15 seconds.

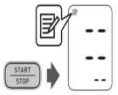

Blood Sample Collection in Field

Rationale for Testing

The rationale of this standard operating procedure is as follows:

- To safely collect the venipuncture blood from the study participants to diagnose and monitor any blood disorder. Blood sample analysis indicates the overall health condition of participants and reveals more specific diseases and conditions.
- To safely collect capillary blood from the study participants for performing theblood sugar test and haemoglobin test in.
- To safe and timely delivery of the blood sample to the laboratory for processing.
- To store the primary samples for appropriate testing and analysis.

Materials Required

- Purple top Ethylenediaminetetraacetic acid (EDTA) blood tubes
- Gel vacutainer
- Syringe with needle(2ml and 5ml)
- Tourniquet band
- Alcohol swab
- Sanitizer
- Absorbent cotton
- Band aid
- Vacutainer rack
- Vaccine carrier
- Gel cool pack
- Biohazard bag
- Bag sealer
- Gloves
- Mask
- Needle destroyer
- Lancet
- Glucometer with strip

- Haemoglobinometer with strip
- Gazelle Hb variant
- Pipette
- Microcentrifuge tube
- Glass slide
- Tips

Preparation of the Study Participants

Before the procedure it is important to follow the steps mentioned below:

- Introduce yourself to the participant, and ask him/her to state their full name.
- Discuss the test to be performed and obtain verbal consent. The participant has the right to refuse a test at any time before the blood sampling, so it is important to ensure that the participant has understood the procedure.

Roles and responsibilities

Laboratory technician 1/Laboratory technician 2: To coordinate and perform a capillary blood test and venous blood collection and to record the testing data in the field investigating table manually and in report format.

General Procedures For Collection of Capillary Blood Drop Samples From Adults And Children

Capillary sampling from a finger or heel (in the case of infants) or an ear lobe may be performed on patients of any age, for specific tests that require small quantities of blood. However, because the procedure is commonly used in paediatric patients. Capillary blood will be collected to test for the following biomarkers: haemoglobin (anaemia) and random blood glucose (risk for diabetes). Capillary blood can be obtained from the palm side of the end of a finger. Following steps are involved in obtaining a capillary blood sample from adults and children.

Choice of site

- For adults and children: The finger is usually the preferred site for capillary testing in adult patients as well as children. The sides of the heel are only used in paediatric and neonatal patients.
- For paediatric and neonatal patients (6-12 months): The selection of a site for capillary sampling in a paediatric patient is usually based on the age and weight of the patient. If the child is walking, the child's feet may have calluses that hinder adequate blood flow. The table shows the conditions influencing the choice of heel or finger-prick.

Preparation of lancet for use:

- **For adults:** A lancet slightly shorter than the estimated depth needed should be used because the pressure compresses the skin; thus, the puncture depth will be slightly deeper than the lancet length.
- **For paediatric and neonates:** Inheel-pricks, the depth should not go beyond 2.4mm. For premature neonates, a 0.85 mm lancet is available.

Complications

Complications that can arise in capillary sampling include:

- The collapse of veins if the tibial artery is lacerated from puncturing the medial aspect of the heel;
- Osteomyelitis of the heel bone (calcaneus)
- Herve damage if the fingers of neonates are punctured
- Haematoma and loss of access to the venous branch used;
- scarring;
- Localizedorgeneralizednecrosis(along-term effect);
- Skin breakdown from repeated use of adhesive strips (particularly in very young or very elderly patients)–this can be avoided if sufficient pressure is applied and the puncture site is observed after the procedure.

Steps In Obtaining Capillary Blood From The Finger Of Adults and Children

Remember the following before collecting the capillary blood drop sample from the finger.

- Chose a place where maximum light availability is there.
- Always put on gloves before beginning the collection of the blood sample from the first participant.

Select and prepare the prick site

- Blood collection is usually easier if you sit on the side of the participant opposite to the hand that you will collect blood from. For example, if you want to collect blood from the left hand, place yourself on the right side of the participant.
- Only use the third or fourth finger for collecting the blood. Do not use a finger with a scar, a wound or cut, an infection, swelling, a deformity, or a rash.
- Also, do not use a finger on which the respondent is wearing a ring, because the ring may disrupt the free flow of blood to the tip of the finger. You can ask the participant to remove the ring.
- Ask the participant to warm his/her fingers by rubbing the palms together briskly until the skin becomes warm. This will increase blood flow to the fingertip and improve the ease with which a sample can be obtained.
- With an alcohol swab, clean the skin of the finger thoroughly. If the swab is stained (with dirt), clean the finger a second time or until the swab is no longer stained. Allow the alcohol to air dry. Do not blow on the area to dry the alcohol. Blowing may deposit bacteria on the skin and contaminate the prick site.

Prick the Finger

- Make sure that the finger is below the level of the participant's heart to increase the flow of blood to the finger. With your thumb, gently push the blood from the top knuckle toward the fingertip.
- When your thumb reaches the fingertip, maintain a gentle pressure to trap the blood in the fingertip.
- Place the lancet firmly against the skin with the trigger facing upwards, so that the arrow preceding the trigger is visible.

- Avoid placing the lancet on the very tip of the finger or the sides beyond the palm area or you will risk piercing the underlying bone. Proper puncture sites are shown in Figure.
- Use the lancet to prick the skin by placing the blade-slot surface against the area and pressing the trigger. The tip of the blade ejects through the blade slot, producing a micro-incision in the skin, and immediately retracts into the device. After pricking the skin, discard the lancet in the biohazard bag.

Collect the blood drops

- When the blood appears, use a sterile gauze pad to wipe away the first one or two drops of blood depending on the tests being performed.
- If the blood stops flowing before you have collected it, the pricking procedure may be repeated with the respondent's consent.
- For minors, you must get consent from the parent or adult responsible for the child. Do not reuse any of the supplies used for the first finger prick.
- After the blood collection, discard all used materials in the biohazardous waste bag.

Figure: Illustration of capillary sampling

1. Lancet and collection tube

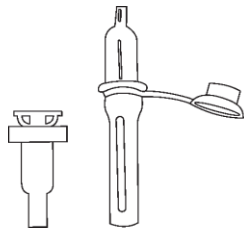

2. Assemble equipment and supplies

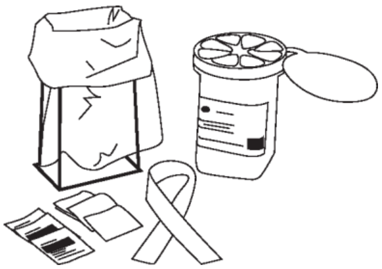

3. Perform hand hygiene using soap and water/sanitizer

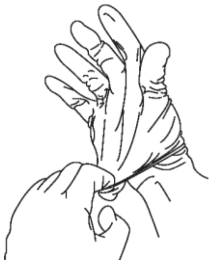

4. Put on well-fitting non-sterile gloves

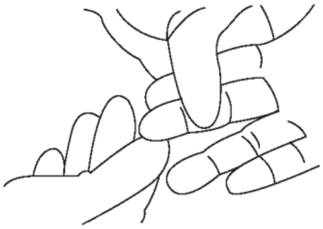

5. Select the site. Apply 70% alcohol and allow to dry

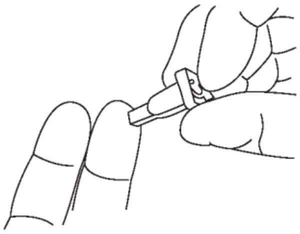

6. Puncture the skin

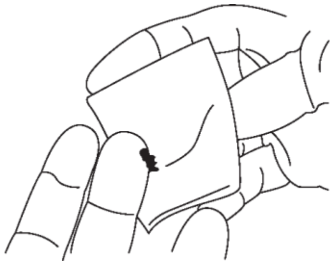

7. Wipe away the first drop of blood

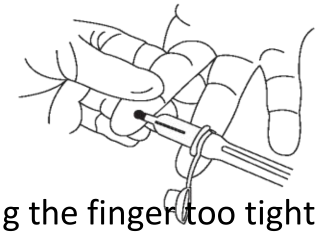

8. Avoid squeezing the finger too tightly

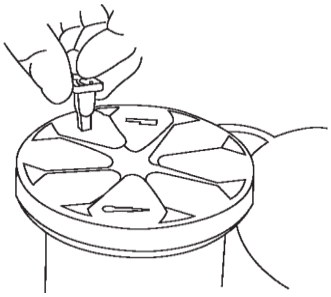

9. Dispose of all the sharps appropriately

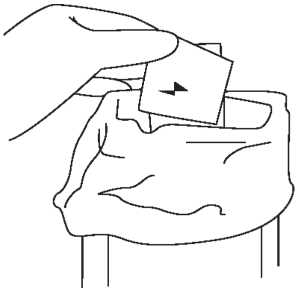

10. Dispose of the waste materials appropriately

11. Perform hand hygiene after removing the gloves

General Procedures For Collection of Capillary Blood Drop Samples From Paediatric And Neonates

For children 6 – 12 months, a heel prick should be performed. The following describes the steps that are involved in obtaining a capillary blood drop sample from the heel:

- The prick should be made outside a line drawn from the middle of the big toe to the heel or outside a line drawn from the area between the fourth and fifth toes to the heel.
- Take care to avoid the central area of the foot (to avoid injury to the nerves and tendons) or the centre of the heel (to avoid piercing the heel bone).
- Inheel-pricks, the depth should not go beyond 2.4mm. For premature neonates, a 0.85mm lancet is available.
- Thedistancefora7pound(3kg) baby from the outer skin surface to the bone is:

- medial and lateral heel–3.32mm.
- posterior heel–2.33 mm (this site should be avoided, to reduce the risk of hitting bone).
- toe–2.19mm.

- Therecommendeddepthforafinger-pricks:

- For a child over 6 months and below 8 years–1.5mm.
- For a child over 8 years–2.4mm.
- Too much compression should be avoided, because this may cause a deeper puncture than is needed to get good flow.

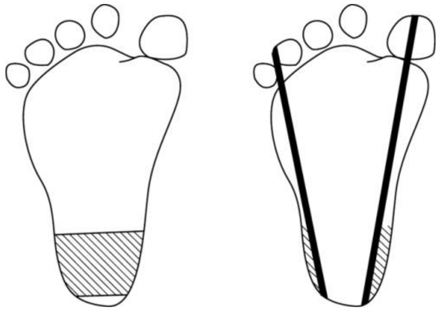

Figure : The capillary blood has to be drawn from the shaded portion of the hill as described in the figure.

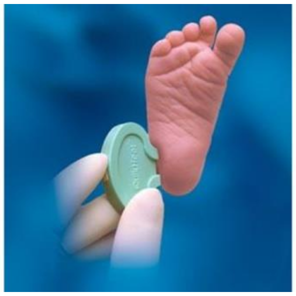

Figure : The area ideal for sample collection by hill prick.

- Immobilizethechild

Immolization of the child is important before the collection of the sample. This can be achieved with the help of the parents:

- Sit on the phlebotomy chair with the child on the parent’s lap;
- Immobilize the child’s lower extremities by positioning their legs around the child’s in a cross-leg pattern;
- Extend an arm across the child’s chest, and secure the child’s free arm by firmly tucking it under their own;
- Grasp the child’s elbow (i.e. the skin puncture arm), and hold it securely;
- Use his or her other arm to firmly grasp the child’s wrist, holding it palm down.

- Prepare the skin: Prepare the skin as described above for adult patients.
- **Prepare the lancet and prick the skin**

- Hold the heel firmly as shown in the figure. Apply moderate pressure near the puncture site. This can be done by wrapping the heel using your thumb and second finger.

- Clean the site with an alcohol swab. Make sure the site is dry before puncturing the skin with the lancet. In selecting a puncture site, avoid any areas of the skin that are broken or appear to be infected.

- Use the lancet for the skin puncture by placing the blade-slot surface against the area and pressing the trigger. Ensure the free flow of blood.

- Wipe away the first two drops of blood using a sterile gauze pad and collect the third drop for haemoglobin testing.

- After blood collection is complete, discard all materials used in the collection procedure in a labelled biohazardous waste container(bag).

- If necessary, take the following steps to improve the ease of obtaining blood by finger-prick in paediatric and neonatal patients:

- ask the parent to rhythmically tighten and release the child's wrist, to ensure that there is sufficient flow of blood;
- keep the child warm by removing as few clothes as possible, swaddling an infant in a blanket, and having a mother or caregiver hold the infant, leaving only the extremity of the site of capillary sampling exposed.
- Avoid excessive massaging or squeezing of fingers because this will cause haemolysis and impede blood flow.

- Unsuccessful attempts in paediatric patients

  Adhere strictly to a limit on the number of times a paediatric patient may be stuck. If no satisfactory sample has been collected after two attempts, seek a second opinion to decide whether to make a further attempt or cancel the tests.

Random Blood Sugar (RBS) Test

Health investigators should keep in mind that random blood glucose testing is done at any time of the day in combination with other biomarkers once the respondent has given her/his consent.

The Device: ACCU CHEK Glucometer

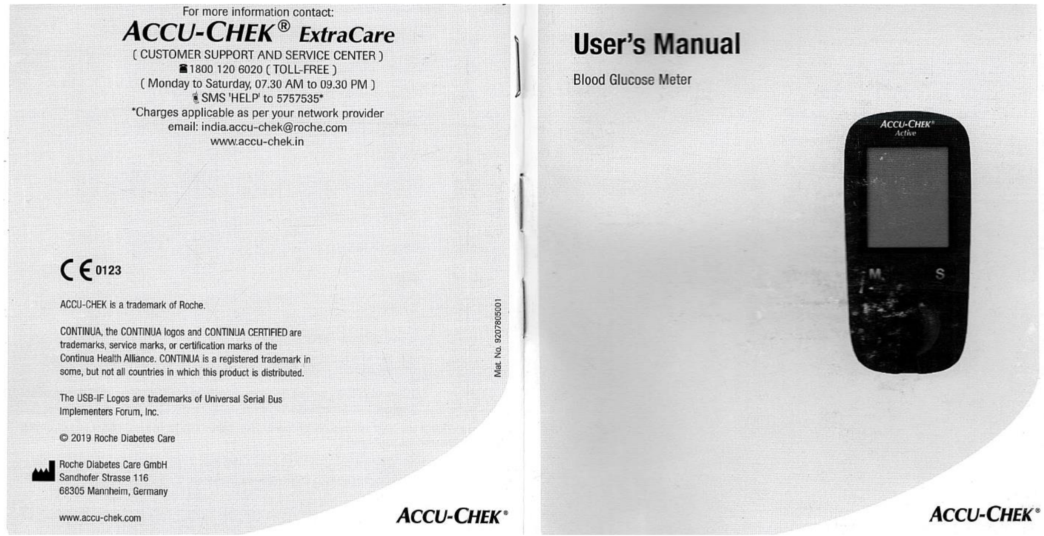

Intended use

The Accu-Chek Active blood glucose meter

The Accu-Chek Active blood glucose meter is intended to be used for quantitative blood glucose tests in fresh capillary blood. The meter may only be used together with Accu-Chek Active test strips. Consult the package insert of the test strips if you intend to use different sample material.

The blood glucose monitoring system comprises the meter and test strips and is suitable for self-testing and for professional use. People with diabetes can use this system to self-test their blood glucose. Healthcare professionals can use this system to check patients' blood glucose values and they can use it in suspected cases of diabetes and in emergency diagnostics.

The system is suitable for testing blood glucose with blood obtained from alternative sites.

The system must not be used to diagnose or rule out diabetes.

The system is only suitable for use outside the body.

Visually impaired persons must not use the meter.

The system does not require a code chip.

The meter must only be used for its intended purpose; if used otherwise preventive measures could be ineffective.

⚠

- Healthcare professionals must also read the instructions and notes in Chapter 10 "Testing blood glucose in more than one patient".
- All objects that could come into contact with human blood are potential sources of infection.

  There is a risk of infections (e.g. hepatitis B, hepatitis C, HIV) being transmitted if the meter is used by other people, even family members, or if healthcare professionals use the same meter to test blood glucose in more than one person.
- Only use the Accu-Chek Active meter with Accu-Chek Active test strips that are approved by Roche. Other test strips deliver incorrect results.
- Keep the blood glucose monitoring system with all its components away from children under the age of 3. There is a risk of suffocation if small parts (e.g. covers, caps or similar objects) are swallowed.

About this User's Manual

Read this User's Manual carefully and completely before testing blood glucose for the first time. If you have any questions, please contact your customer support and service centre (see page 61).

This User's Manual will provide you with all the information you need to operate, troubleshoot and care for your blood glucose meter. Follow the correct operating procedures for the meter and comply with all instructions for use. You can turn the beep tone of the meter on or off. This User's Manual assumes that the beep is on.

Actions to be taken appear as shown in the example below:

1

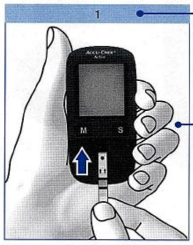

Title bar of the figure with a number or figure caption

Figure displaying actions to be taken

Slide the test strip into the test strip guide in the direction of the arrows until you feel it lock into place.

Action to be taken (blue text)

The meter turns on and first performs a default display check (approx. 2 seconds).

Information on action to be taken (black text)

Flashing elements

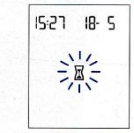

In this User's Manual you will see examples of display screens. Elements that are surrounded by a halo in these examples actually flash on the meter's display.

Please note: All dates, times or results shown on the display screens in this User's Manual are intended only as examples.

Getting to know the blood glucose monitoring system

The Accu-Chek Active blood glucose meter and its components

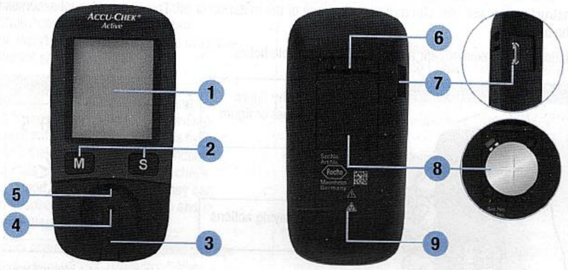

1. **Display**
   displays the current and saved test results or device messages
2. **Buttons**
   see "Buttons of the blood glucose meter" overview
3. **Cover**
   covers the measurement window
4. **Measurement window**
   underneath the cover
5. **Test strip guide**
   slide the test strip in here
6. **Locking catch**
   opens the battery compartment cover
7. **USB port**
   the USB cable is inserted here in order to transfer data to a computer
8. **Battery compartment**
   right: open battery compartment, battery type CR2032
9. **Type plate**

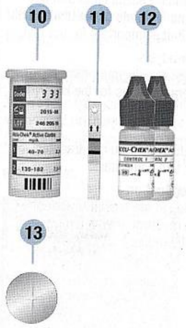

10. **Test strip container**
11. **Test strip**
12. **Control solutions**
13. **Battery**

Buttons of the blood glucose meter

Overview of the M and S button functions:

M button	S button
M	S
Press the M button to	Press the S button to
turn the meter on and call up saved values	turn the meter on and call up settings, e.g. for the time
mark the test result after a test	mark the test result after a test
change settings	go to the next setting
call up the previous saved test result	call up the next saved test result
change from the earliest saved test result to the averages	change from the averages to the saved test results
call up the previous average	call up the next average
Press the M **and** S buttons at the same time to	
perform a display check	
save settings after the final display is shown on the display and turn the meter off	
turn the meter off	
Press the M **or** S button to	
turn the test reminder beep off	

2 Steps before testing

Checking the display

You can check if all of the display elements are properly displayed, by carrying out a full display check.

1

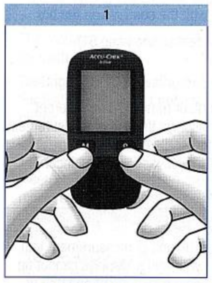

With the meter turned off, press the M and S buttons at the same time for approximately 2 seconds until the meter is switched on.

2

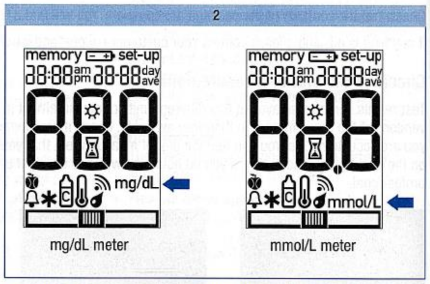

Compare the elements displayed on your meter with the display shown here.

If any of the display elements are missing or the unit of measurement shown for the blood glucose results is not the correct one, please ask your healthcare professional to exchange the meter.

Press the M and S buttons at the same time to end the display check and turn the meter off.

Testing blood glucose

Using the Accu-Chek Active meter, you can test blood glucose in two different ways. You can eithe- apply the blood drop to the test field while the test strip is in the meter (see page 17), or you can remove the test strip from the meter and then apply the blood drop to the test field (see page 18).

Read the package insert which comes with the test strips. Please also consult "Notes on blood glucose testing" (see page 20).

Preparing to test blood glucose

You need the following items to carry out the blood glucose test:

- your meter
- the Accu-Chek Active test strips
- a lancing device to obtain blood
- a lancet for the lancing device

1

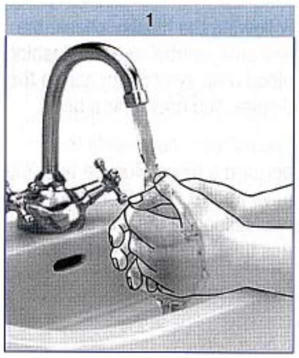

Wash your hands thoroughly with soap and warm water and dry them well. This reduces contamination of the puncture site and stimulates blood flow.

2

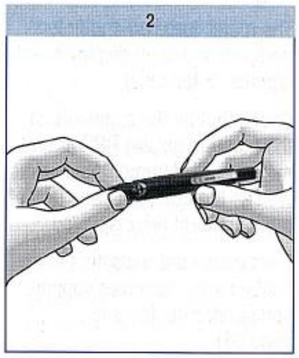

Prepare your lancing device to obtain blood.

3

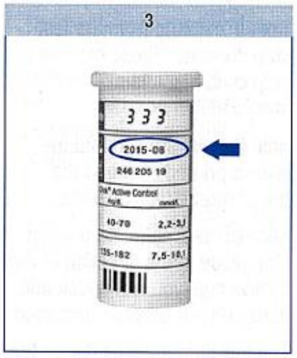

Check the use by date on the test strip container.

Use only test strips which are within the use by date.

Testing blood glucose

Turning the meter on

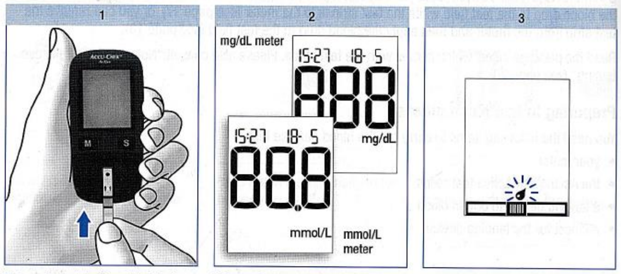

1 Take a test strip from the test strip container. Close the test strip container again immediately.

Hold the test strip so that the arrows printed on it and the green square face upwards.

Slide the test strip into the test strip guide in the direction of the arrows until you feel it lock into place. Do not bend the test strip.

The test strip must lie flat on the measurement window cover.

2 The meter turns on and first performs a default display check (approx. 2 seconds).

Check that all the segments of the numeric display 888 (mg/dL meter) or 88.8 (mmol/L meter) as well as the time, date and unit of measurement are displayed.

If segments are missing, please contact your customer support and service centre (see page 61).

3 Following the display check, the test strip symbol and the flashing blood drop symbol appear on the display. You now hear a beep.

The meter is now ready to perform a blood glucose test. You have approx. 90 seconds to apply blood to the test strip. After this time, the meter turns itself off.

Applying blood

With test strip in the meter

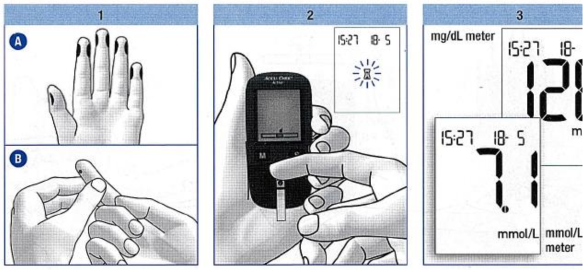

1 Lance the side of a fingertip with your lancing device.

Figure A shows the recommended sites for obtaining blood.

Encourage a blood drop to form by gently massaging the finger towards the fingertip (see B).

2 Apply the blood drop to the centre of the green field and then remove your finger from the test strip.

As soon as the meter detects the blood, you hear a beep.

The test starts. The flashing hourglass symbol ⌛ indicates that the test is in progress.

If the amount of blood you applied is insufficient, after a few seconds an acoustic warning in the form of 3 beeps sounds. You can then apply an additional amount of blood.

3 The test is over in approxim[illegible] 5 seconds. The test result appears on the display and hear a beep. The meter automatically saves the tes[illegible] result.

You can now mark the test [illegible] set a test reminder or turn t[illegible] meter off.

Applying blood

With test strip outside the meter

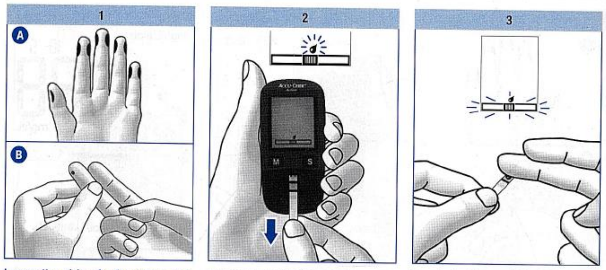

1 Lance the side of a fingertip with your lancing device.

Figure A shows the recommended sites for obtaining blood.

Encourage a blood drop to form by gently massaging the finger towards the fingertip (see B).

2 When the test strip and the flashing blood drop symbols are displayed:

Remove the test strip from the meter.

3 The test strip and blood drop symbols flash afterwards on the display. Now you have approx. 20 seconds to apply blood to the test strip and to slide it into the meter again. During the last 5 seconds, a beep sounds once per second. It is to remind you to slide the test strip with blood back into the meter. If you do not slide the test strip back into the meter within this time, the meter displays the error message E-2.

Apply the blood drop to the centre of the green field.

Turning the meter off

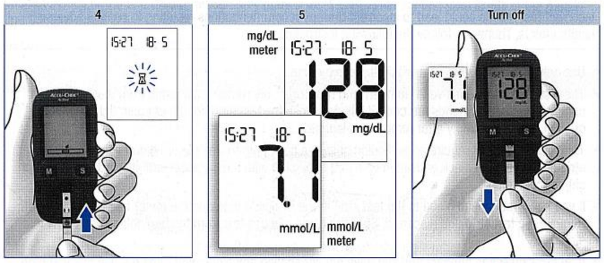

4 Slide the test strip into the test strip guide in the direction of the arrows until you feel it lock into place.

The test strip must lie flat on the measurement window cover.

The test starts. The flashing ⌛ hourglass symbol indicates that the test is in progress.

5 The test is over in approximately 8 seconds. The test result appears on the display and you hear a beep. The meter automatically saves the result.

You can now mark the test result, set a test reminder or turn the meter off.

Turn off Remove the test strip from the meter.

The meter turns itself off.

Discard used test strips according to local regulations. Healthcare professionals must discard used test strips according to the health and safety policy of their laboratory or institution.

If you do not remove the test strip and do not press any buttons, the meter turns off automatically after approx. 30 seconds.

Notes on blood glucose testing

> Incorrect test results could lead to incorrect therapy recommendations resulting in serious adverse health effects. Therefore, follow the following instructions:

- Use only test strips which are within the use by date.
- The test strips are sensitive to humidity and moisture. Only remove test strips from the test strip container when your hands are completely dry. Close the test strip container again tightly with its original cap immediately after removing a test strip.
- This meter does not require an activation chip or a code chip for measuring blood glucose. If your test strip box contains a black activation chip or a code chip with a different colour, discard the activation chip or code chip.
- If you have not applied blood to the test strip in the available time and the meter has turned itself off: Remove the test strip and discard it. Start the blood glucose test from the beginning with a new test strip.
- If an error message appears and you have not yet applied any blood, you must not use this test strip any more. Start the blood glucose test from the beginning with a new test strip.
- Do not rub the blood drop onto the test field when applying blood.
- Do not store used test strips in a test strip container which still contains unused test strips.
- Do not bend the test strip while sliding it into the meter. The test strip must lie flat on the measurement window cover.
- Do not to bend or move the test strip before or while applying blood, or while the test is in progress.
- Only apply blood to the test strip when the drop symbol is flashing on the display.
- If 888 or 888 are not shown completely during the default display check (e.g. 828), test results cannot be displayed correctly. In this case, contact your customer support and service centre.

> If you want to apply blood to a test strip which is not in the meter: Do not remove the test strip until the drop symbol flashes on the display. If you remove the test strip before this time, the meter turns itself off.

Disposal of The Waste Material

After performing the test the lancet should be burnt in needle destroyer and all the biohazard waste should be discarded in the appropriate colour coded biohazard bags following the biomedical waste disposal guidelines

Haemoglobin Test

For haemoglobin test the infants, children, adults (men and women) and old aged people are included in the study. Following are the stepwise procedure for conducting haemoglobin test in different age group of people.

The Device: Haemoglobinometer and the procedure of measuring haemoglobin as per manufacturer's guidelines:

Pricking finger is mentioned in the manual.

Chapter – 2 Intended Use and Test Principle

Intended use

The TrueHb Hemoglobin Monitoring kit is intended to be used for the quantitative measurement of Hemoglobin in fresh capillary whole blood samples taken from the forearm, upper arm, hand, thigh, calf, or fingers. The TrueHb Hemoglobin Monitoring kit is intended for use outside the body (in vitro diagnostic use) by healthcare professionals & individual only. TrueHb Hemoglobin Monitoring kit is calibrated against the complete blood count analyzer that is verified of calibration as per International Council for Standardization in Hematology (International Council for Standardization in Hematology technical report 1-2009: new reference material for haemiglobincyanide or Hemoglobin for use in standardization of blood Hemoglobin measurements). This is a screening device only. Do not take any decision of medical relevance without first consulting your medical practitioner.

Test principle

TrueHb Hemoglobin Monitoring kit is based on the principle of reflectance photometry. A drop of blood is applied on the Strip. It disperses within the hydrophilic mesh. The Hemoglobin is extracted out from the RBC and, with the help of reagents present in the Strip, is converted into a complex. The optical reflectance is measured which is inversely proportional to the concentration of Hemoglobin in blood sample. This corresponds to the total Hemoglobin present in blood.

Important:

For accurate results:

- Read instructions carefully before use
- The TrueHb Hemometer must only be used with TrueHb Strips
- Test only whole blood Specimens. EDTA or heparin anticoagulants can be used for in vitro diagnostic only.
- For traceability of the quality and calibration of the device, please use the serial number of the device and batch code of TrueHb Strips mentioned on the device packaging label.

Introduction

Hemoglobin is a protein present in the red blood cells (RBCs). It is associated with many functions like transport of nutrients, and transport of oxygen to a variety of tissues inside the body.

Thus, total Hemoglobin is one of the most frequently ordered laboratory tests. The most common purpose for conducting this test is for the detection of anemia. In addition, Hemoglobin levels also guide many clinical diagnosis and therapeutic interventions.

Serial assessments are often made to track disease progression, blood loss and efficacy of the therapies involved to restore the Hemoglobin levels to normal, the changes involved before and after operations or blood transfusions and screening of the blood donors to ensure that the Hemoglobin content of the donor meets the minimum required Hemoglobin for donating blood.

TrueHb Hemoglobin Monitoring kit can be used for measuring Hemoglobin (Hb) from samples taken from the forearm, upper arm, hand, thigh, calf, or fingers.

Normal Hemoglobin Levels

The normal Hemoglobin range (anything below or above range is low or high Hemoglobin respectively) is indicated as follows:

Adult Males 13.5-18 g/dL

Adult Females 12-16 g/dL

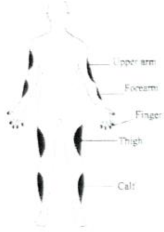

Interpretation of Results

TrueHb Hemoglobin Monitoring kit measures the Hemoglobin within blood. In the event of unexpected results, following steps are recommended:-

- Check the expiry date of TrueHb Strips.
- Repeat the test again using a new Strip if TrueHb Strips are not expired
- If problem still persists, discontinue using the TrueHb Strips and contact the nearest Distributor or contact our customer care.

Chapter – 3

Warnings

- We recommend to take blood sample from the tip of your fingers and removing first two blood drops after pricking and use third drop for the test
- Severe dehydration and excessive water loss may cause falsely elevated results. If you think you are severely dehydrated, contact your healthcare professional immediately.
- Keep the TrueHb Hemometer and TrueHb Strips out of children's reach.
- Do not use TrueHb Hemoglobin Monitoring kit for treating or managing any disease condition.
- As per medical experts, one must avoid donating blood in case his/her Hemoglobin level is under 12.5g/dl.

Chapter –4 About the TrueHb Hemoglobin monitoring kit

About TrueHb Hemometer

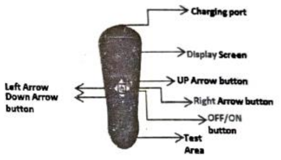

Display Screen: Displays your test results, date and time etc.

Right, Left, Up and Down Buttons: To move among displayed items

On / off / Select button: This button turns the TrueHb Hemometer on and off and also selects the highlighted item

Test Strip slot: For inserting the test Strip

Mini-USB charging port: For charging the battery

Display Indications:

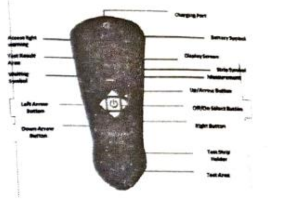

Batch code: Indicates the code number of test Strip.

Battery Symbol: Indicates the battery level

Strip Symbol: Indicates when to inserttest Strip

Blood Drop Symbol: This symbol appears when the TrueHb Hemometer is ready for you to apply blood drop.

Test Result Area: Shows the Hemoglobin level

Measurement Unit s: Indicates the units for the test result (g/dL)

Memory: Indicates a test result is being recalled from memory

Turning ON & OFF the TrueHb Hemometer:

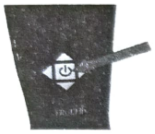

Long press the ⏻ button for more than one second to turn ON the TrueHb Hemometer. If the TrueHb Hemometer does not turn on, try charging the TrueHb Hemometer. Long press the same ⏻ button for more than 1 second to turn OFF the TrueHb Hemometer at any point in time.

Performing the Test:

1. Turn ON and charge the TrueHb Hemometer. The system undergoes an auto check and Auto-calibration after which the battery level, Strip's Batch code are displayed within 2 seconds.

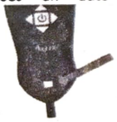

2. If the displayed batch code is found to be different from the code mentioned on the Strip bottle, Use ◄ and ► buttons to move to the correct digit and ▲ and ▼ buttons to set the correct value. Accept the batch code by pressing the ⏻ button.
3. The TrueHb Hemometer would flash 'Strip' symbol on the display. Insert a fresh test-Strip into the TrueHb Hemometer with the arrows on the Strip facing up and pointing towards the display.
4. Ensure the correct positioning of the Strip with the guiding V notch and the positioning hole in the Strip properly placed and locked.
5. Do not allow fingers or other foreign objects to come in contact with the white test area of the test-Strip
6. Immediately after inserting the fresh test Strip the display would flash the ♦ (Blood) symbol. Prick a body surface, and drop the second or third hanging drop of blood from on the white test area on the Strip. Make sure the body part Used for extracting blood sample is clean and disinfected.
7. Ensure that there is sufficient blood (a minimum of 10μl) to completely cover the white colored test area.

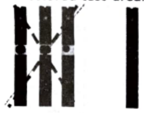

Incorrect Correct

8. The TrueHb Hemometer will automatically start sample evaluation and display ⌛ and a time counter.
9. Within a minute the test result will be displayed on the TrueHb Hemometer. The SI unit of the test result is g/dL. Up to 1000 most recent test result will automatically be stored in the TrueHb Hemometer
10. Remove the used test Strip from the TrueHb Hemometer and dispose it as per local Regulations and blood sample disposal guidelines.
11. TrueHb Hemometer should be broken down into small pieces in case of disposal.

Quality Control

To check performance of the TrueHb Hemometer and TrueHb Strips, known specimen of blood control solution shall be used to check the performance of the kit. In case you find any diversion in the result, please check the expiry of the control solution. If you continue to get control solution test results that falls outside the range printed on the control solution, stop using the TrueHb Strips immediately. Bio-Rad - Meter Trax Control - Low, Mid and High is a recommended control solution for Hemoglobin test.

Chapter –5 Do's and Don'ts for TrueHb Hemometer & Strips

Do's

1. The TrueHb Hemometer should be cleaned after each use to remove any excess blood.
2. Remove any residual blood or other matter on the outside of the TrueHb Hemometer by using non fiber cloth dampened with mild disinfectant or water. Clean the TrueHb Hemometer before and after use with non- fiber dampened cloth.
3. Clean the optical lens regularly by removing the black bottom part of TrueHb Hemometer with moist cotton cloth every day before use and after every 10 blood tests, or in case where dust or dirt has fallen on the optical lens. Make sure you do not scratch the optical lens' surface as damaging the lens may lead to inconsistent results.
4. Store your TrueHb Hemometer and Strips, and other items in your carrying case after each use in a cool, dry place 20°C to 30°C.
5. Clean your hands properly before performing the test.
6. Use the TrueHb Strips provided with the TrueHb Hemometer for accurate results.
7. Please check the expiration date of the TrueHb Strips provided on the container before using them for testing the Hemoglobin.
8. Store TrueHb Strips only in their original vial.
9. Keep the Strip channel clean
10. Keep the TrueHb Hemometer Dry and avoid exposing it to extreme temperatures or humidity as per the specifications.
11. For in vitro diagnostic use
12. Use TrueHb Strips only with the genuine TrueHb Hemometer.

Don't

1. Do not use alcohols to clean the TrueHb Hemometer. Alcohols damage the TrueHb Hemometer and the optical lens.
2. Don't refrigerate the TrueHb Hemometer & Strips.
3. Do not use strong, abrasive or corrosive cleaning solutions as they can damage the TrueHb Hemometer and the optical lens.
4. Never dip or immerse the device in disinfectant solution.
5. Do not let any liquids, dirt, dust, blood, or control solution spill inside the TrueHb Hemometer as it may damage the electronics of the TrueHb Hemometer.
6. Do not store the TrueHb Hemometer close to strong magnetic field, wireless system or mobile telephones, as this may interfere with the proper operation of the TrueHb Hemometer.
7. Do not touch the TrueHb Strips where blood sample is to be applied.
8. Do not get the water and other liquids inside the TrueHb Hemometer.
9. Do not clean the unit with substances such as gasoline, paint thinner or other organic solvents to avoid any damage to the TrueHb Hemometer.
10. Avoid exposing the TrueHb Hemometer to extreme temperature and humidity.
11. Do not freeze or refrigerate the TrueHb Hemometer & Strips.
12. Use TrueHb Strips only within the system operating temperature range as specified in the specifications
13. Store your TrueHb Strips in their original vial only; never transfer them to another vial or any other container
14. Never store individual TrueHb Strips outside the vial

15. After removing a TrueHb Strip from the vial, immediately close the vial cap tightly.
16. With clean, dry hands, you may gently touch the TrueHb Strip on white parts while handling them.
17. Do not use TrueHb Strips beyond the expiration date as this may cause inaccurate results
18. Do not bend, cut, deform or alter TrueHb Strips
19. Do not remove the strip while the test is running.
20. Do not keep the device idle for 60 or more than 60 days, in case the device is not in use, please charge the device fully.

Chapter –6 How to perform the Finger-Prick Test

1. Before calling the patient put on powder-free gloves, and keep alcohol swabs or gauze pads ready for use.
2. For maximum and neutral blood flow the patient's fingers or puncture site should be kept straight, and not bebent.
3. Freely flowing blood sample is essential for accurate results. Take following precautions to get the right sample:

i. For enabling natural blood flow within the patient's body make the patient sit comfortably on the chair.
A young child should be seated in the guardian's lap so that the child is comfortable.

ii. Clean the puncture site with the alcohol swab or gauze pad before pricking and let it dry. Gently rub or massage the puncture site before puncture, to enable free flow of blood in the puncture site makes sure that the puncture site is warmso that blood circulates freely before sampling.

iii. Ring or middle finger of lefthand should be preferred as the puncture site as these partshave been found to produce best results
Note- Remove ring, ornaments, knots or any other substance, which may obstruct the natural blood flow in thepuncture.

iv. Using a rolling movement ofyour thumb, lightly press the finger from the top knuckle towards the tip. This stimulates the flow of blood towards the puncture site. When the thumb has reached the fingertip, maintain gentle pressure and puncture the palm side surface of the finger – halfway between the nail and the finger pad. Using the spot on the finger causes lesspin and produces the best flow of blood. While performing the puncture, the hand or the sample site must be at or below the heart level.

4. The first two drops of blood coming out of the puncture site may contain cell debris or excessive plasma, and therefore wipe away the first two drops of blood using gauze pads and only use the third hanging drop for evaluation to have homogenous blood sample.
5. Turn ON the TrueHb Hemometer and insert a batch code of TrueHb Strip on the TrueHb Hemometer with the help of buttons and press the power button.
6. Insert a fresh Strip in the TrueHb Hemometer. TrueHb Hemometer display will ask for blood drop, puncture the finger and use the third hanging drop of blood on the test Strip.
7. Count down starts on the TrueHb Hemometer and Hemoglobin result will be shown on the display within a minute.
8. Dispose the used TrueHb Strip as per local biological waste disposal guidelines.

Precautions while performing the test

i. In case of insufficient blood sample, DO **not** milk the puncture site. The homogeneity of the blood sample disrupts upon milking. In case of insufficient blood sample repeat the sampling at a fresh puncture site.
Note – For accurate results, use 23 gauge lancets

ii. Avoid handling a TrueHb Strips from its container when your fingers are wet with alcohol or water as these may damage the TrueHb Strips.

iii. If third drop of blood is not enough redo the sampling at a fresh puncture site.

iv. DO not try to top up the testing area in case the entire Strip is not reddened in one attempt as topping up can cause falsely deflected results.

v. Be careful to not obstruct blood flow to the puncture site. Do not hold the patient's hand too tightly as that may obstruct blood flow to the fingers.

Warning: Do not use your TrueHb Strips if your vial is damaged or left open to air. This could lead to error messages or false readings.
The TrueHb Monitoring kit contains small parts that may be dangerous if swallowed

Chapter – 7 TrueHb Hemoglobin Monitoring kit Specifications

Assay method	Reflectance Photometry
Automatic shutoff	Yes
Battery life	300 cycles
Display	LCD
Measurement units	(g/dL)
Memory	1000 blood sample test
Blood Range	0 – 24g/dl
Operating Relative Humidity	5% to 95% (non-condensing)
Operating temperature of TrueHb Hemometer	5 to 52˚C
Power Source	One 3.7 volt Lithium-polymer rechargeable battery
Sample	Venous & Capillary Whole blood
Storage Conditions of TrueHb Strips	0˚C to 30˚C
Testing Time	20 to 59 Sec
Blood Volume	10 µl

Chapter – 8 Troubleshooting

I. TrueHb Hemometer does not turn ON	
**Probable Cause**	**What to Do**
The battery is discharged	Charge the **TrueHb Hemometer**
TrueHb Hemometer doesn't charge	Call customer support
The display is damaged	Call customer support
Defective **TrueHb Hemometer**	Call customer support
**II. Wide/Unexpected variance in results**	
**Probable Cause**	**What to Do**
Optical Detector is not clean	Properly clean **TrueHb Hemometer** with damp cloth
Insufficient amount of blood	Make sure test region of Strip is completely reddened with enough blood sample (min. 10µl)
TrueHb Strips expired, damaged or poorly stored	Use a fresh Strip stock
**TrueHb Hemometer** body damaged or hardware problem	Call customer support

Error Messages

Error 1

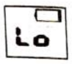

Lo along with the battery symbol
What it means – The battery is exhausted during device startup
What to do – Kindly charge the device via USB cable

Error 2

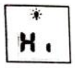

Hi along with the flashing bulb
What it means – The ambient light is very high for testing, and user needs to test under shade
What to do - Kindly perform the test under the shade.

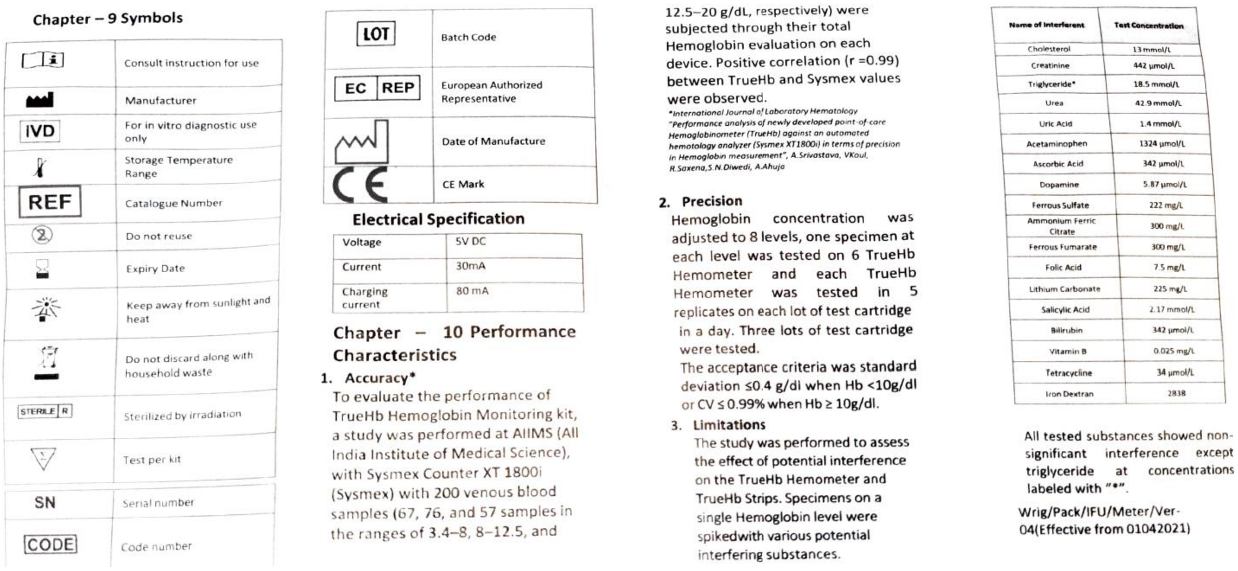

Chapter – 9 Symbols

	Consult instruction for use
	Manufacturer
IVD	For in vitro diagnostic use only
	Storage Temperature Range
REF	Catalogue Number
	Do not reuse
	Expiry Date
	Keep away from sunlight and heat
	Do not discard along with household waste
STERILE R	Sterilized by irradiation
	Test per kit
SN	Serial number
CODE	Code number
LOT	Batch Code
EC REP	European Authorized Representative
	Date of Manufacture
CE	CE Mark

Electrical Specification

Voltage	5V DC
Current	30mA
Charging current	80 mA

Chapter – 10 Performance Characteristics

1. **Accuracy***
To evaluate the performance of TrueHb Hemoglobin Monitoring kit, a study was performed at AIIMS (All India Institute of Medical Science), with Sysmex Counter XT 1800i (Sysmex) with 200 venous blood samples (67, 76, and 57 samples in the ranges of 3.4–8, 8–12.5, and 12.5–20 g/dL, respectively) were subjected through their total Hemoglobin evaluation on each device. Positive correlation (r =0.99) between TrueHb and Sysmex values were observed.

**International Journal of Laboratory Hematology "Performance analysis of newly developed point-of-care Hemoglobinometer (TrueHb) against an automated hemotology analyzer (Sysmex XT1800i) in terms of precision in Hemoglobin measurement", A.Srivastava, VKoul, R.Saxena,S.N.Diwedi, A.Ahuja*

2. **Precision**
Hemoglobin concentration was adjusted to 8 levels, one specimen at each level was tested on 6 TrueHb Hemometer and each TrueHb Hemometer was tested in 5 replicates on each lot of test cartridge in a day. Three lots of test cartridge were tested.
The acceptance criteria was standard deviation ≤0.4 g/dl when Hb <10g/dl or CV ≤ 0.99% when Hb ≥ 10g/dl.

3. **Limitations**
The study was performed to assess the effect of potential interference on the TrueHb Hemometer and TrueHb Strips. Specimens on a single Hemoglobin level were spikedwith various potential interfering substances.

Name of Interferent	Test Concentration
Cholesterol	13 mmol/L
Creatinine	442 µmol/L
Triglyceride*	18.5 mmol/L
Urea	42.9 mmol/L
Uric Acid	1.4 mmol/L
Acetaminophen	1324 µmol/L
Ascorbic Acid	342 µmol/L
Dopamine	5.87 µmol/L
Ferrous Sulfate	222 mg/L
Ammonium Ferric Citrate	300 mg/L
Ferrous Fumarate	300 mg/L
Folic Acid	7.5 mg/L
Lithium Carbonate	225 mg/L
Salicylic Acid	2.17 mmol/L
Bilirubin	342 µmol/L
Vitamin B	0.025 mg/L
Tetracycline	34 µmol/L
Iron Dextran	2838

All tested substances showed non-significant interference except triglyceride at concentrations labeled with "*".

Wrig/Pack/IFU/Meter/Ver-04(Effective from 01042021)

Troubleshooting of TrueHb Haemoglobinometer in Field

It is advisable that after every 3 testing in the haemoglobinometer, the sensor has to be cleaned with a piece of dry tissue paper. For this purpose, the black-coloured portion of the haemoglobinometer should be pulled and opened to clean the sensor. The process is described as follows:

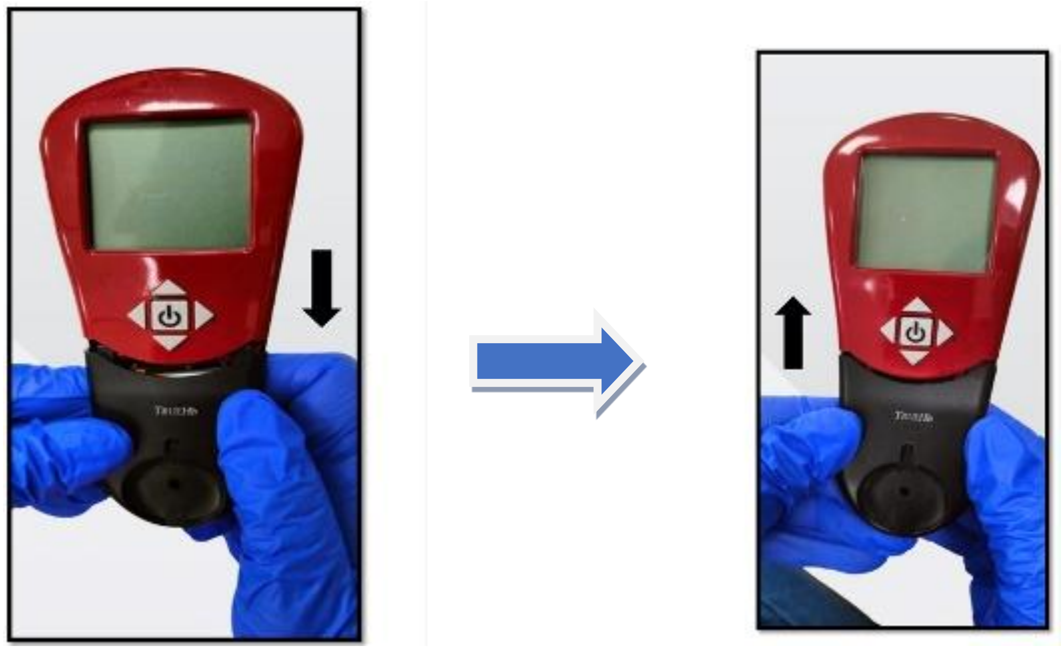

Process for cleaning: -

- Use a damp cloth and clean the black bottom body of the TrueHb Hemometer.
- Remove the bottom body (Black part) by pressing the sides of the TrueHb Hemometer.
- Wipe the lens with a dry cloth. Do not scratch the sensor area.
- Do not use bleach or alcohol to clean the lens and black bottom body of the TrueHb Hemometer.
- Slide the black bottom body back into the TrueHb Hemomete**r**

Disposal of The Waste Material

After performing the test the lancet should be burnt in a needle destroyer and all the biohazard waste should be discarded in the appropriate colour-coded biohazard bags following the biomedical waste disposal guidelines

Sickle Cell Disease/Trait Test

- For Sickle cell disease/trait test infants, children, adults (men and women) and old aged people are included in the study**.** Following are the stepwise procedure for conducting haemoglobin tests in different age groups of people**.**
- The test will be performed after collecting a venous blood sample in an EDTA tube.
- The details of the blood collection procedure are mentioned in the next section.
- The details of the test are mentioned below:

The Device: Gazelle Hb variant and the procedure of detecting sickle cell disease/trait as per manufacturer's guidelines:

Gazelle™ Hb Variant Test Instructions for Use

gazelle™

Intended Use

Gazelle™ Hb Variant is an *in vitro* diagnostic test intended for quantitative measurement of hemoglobin A, F, S, A2/C/E using cellulose acetate electrophoresis. It uses the Hb Variant Test Multipack, including cartridges. The test also provides an interpretation of the results. The test is for professional use in a healthcare point-of-care lab setting and requires human whole blood.

Overview

Gazelle™ Hb Variant is an *in vitro* diagnostic device that can aid clinicians in the diagnosis of certain hemoglobin abnormalities.

The **Gazelle Hb Variant** test uses a miniaturized version of electrophoresis technology for hemoglobin variant detection. It identifies and quantifies Hb A (normal), Hb S (sickle), Hb F (fetal) and Hb A2/C/E.* The Reader uses this information to offer interpretative statements for possible conditions such as sickle cell disease and trait, and certain thalassemias. A small sample consisting of whole blood and marker fluid (which serves as a control) is lysed and applied to the cartridge, which is then placed into the Reader for analysis. In about 8 minutes, the Reader displays the result on the screen, including hemoglobin types and percentages. Interpretative statements are also presented on the screen.

* See *Limitations*

Gazelle consists of a Reader and Gazelle™ Hb Variant Test Multipack. Test results are stored digitally on the Reader and can be printed or transferred to a USB drive as a PDF.

Principle of Procedure

Microchip electrophoresis: Gazelle uses a miniaturized version of the gold standard test known as electrophoresis. When the disposable cartridge containing a lysed blood sample is inserted into the Reader, the hemoglobin types are separated according to their charge. In about 8 minutes, the interpretation (normal, trait, disease) as well as the types and percentages of hemoglobin are displayed on the screen and stored digitally.

Gazelle separates hemoglobin types in a small volume of blood on a piece of cellulose acetate paper that is housed in a cartridge. The basis of Gazelle's technology lies in hemoglobin electrophoresis in which different hemoglobin types, including A (normal), S (sickle), C (hemoglobin C disease), A2, E (hemoglobin E disease), and F (fetal) have different net negative charges in an alkaline solution and will move across the paper at different speeds due to an applied voltage. The different hemoglobin types will separate into visible bands on the paper.

The Gazelle Reader contains custom software which controls the user interface, voltage/current used in the electrophoresis process, video and image capture over the duration of the test, imaging analysis algorithms, data storage, and data transfer. The image analysis algorithm extracts the image characteristics of the hemoglobin bands to determine the type of hemoglobin as well as its relative amount. This information is used to offer an interpretative statement based on hemoglobin proportions.

Warnings and Precautions

WARNING: Operators should familiarize themselves with the Warnings and Precautions provided in the *Gazelle™ Setup Guide* or onscreen *Gazelle™ Diagnostic Device User Manual* before proceeding with the test.

WARNING: Only operate Gazelle Hb Variant for its intended purpose and in accordance with these instructions for use and warnings. If the Reader is used in a manner not specified in the instructions for use, protection provided by the equipment will be impaired. The Reader (including power supply) is designed to operate within the manufacturer's specifications. Do not exceed the manufacturer specifications when in use.

WARNING: The reader contains a lithium ion battery. Keep the device away from heat greater than the operating temperature and fire.

DANGER: Gazelle Hb Variant Buffer contains a 1:10 part dilution of TBE Buffer, which at full strength may damage fertility and the unborn child. A health hazard determination with a diluted buffer has not been conducted.

WARNING: Gazelle Hb Variant Marker Fluid contains Xylene Cyanol which may cause skin, eye or respiratory irritation.

WARNING: Do not move the device while a test is in progress. To avoid damage to the Reader, power down the Reader before moving it. Press and hold the power button until the POWER OFF? Screen appears and select YES from the screen selections.

WARNING: Do not insert fingers or other foreign objects inside the Malaria Cartridge Chamber.

WARNING: Follow proper infection control guidelines for handling all specimens and related items. Properly dispose of all contaminated waste.

WARNING: Dispose of the following as biohazardous waste: used or expired fluid bottles, stampers, Hb Variant cartridges and contents at the end of each test and unused testing fluids at the end of the day. Do not dispose of buffer in a manner that could allow it to enter the ground water system.

WARNING: If it becomes necessary to dispose of the Reader, follow local guidelines for disposing non-reusable devices with lithium ion batteries.

CAUTION: If any Gazelle product is dropped, cracked, found to be damaged, or open when received, DO NOT USE.

CAUTION: Do not reuse cartridges.

CAUTION: Contamination of the work area with previous samples may cause erroneous results. Handle samples according to standard laboratory practices.

CAUTION: Gazelle factors age into the analysis, therefore, it is important to accurately enter the patient's age.

NOTE: Any serious incident that has occurred in relation to this *in vitro* diagnostic medical device should be reported to the manufacturer and the authority having jurisdiction where the *in vitro* diagnostic medical device was purchased or used.

Materials Included with Gazelle Hb Variant Test Multipack REF S-10

- 25 Hb Variant Cartridges
- 1 bottle of Hb Variant Buffer (45 mL)
- 1 bottle of Hb Variant Marker Fluid (5 mL)
- 25 Hb Variant Stampers (Blood Sample Applicators)
- 1 Stamper Stand
- *Gazelle™ Hb Variant Test Instructions for Use*

Associated Materials Required

- Gazelle™ Reader REF GZ-100

Active Ingredients of Materials Provided

- Gazelle Marker Fluid contains 0.00625g/mL Xylene Cyanol and other ingredients which might influence the measurement.
- Gazelle Buffer contains 1% TBE Buffer by volume and other ingredients which might influence the measurement.

Materials Required but not Provided

- Vortexer
- 2 Eppendorf tubes (1.5 mL) per day of testing, plus one additional tube for each test
- Pipettor and pipette tips (20 mL and 200 mL)
- Glass slides
- Blood contamination waste container
- Sharps container
- Personal protective equipment

Storage and Shelf-life

- Multipack transportation temperature range: 5°C to 45°C
- Multipack storage and temperature range: 5°C to 45°C
- Multipack storage and operating relative humidity range:
- 5% to 95%
- The Multipack expires after 1 year and 3 months

NOTE: All stability testing was conducted on one lot.

CAUTION: All materials should only be used before their stated expiration dates.

Specimen Handling

- Blood samples are stable after collection up to 14 days in refrigerated conditions (2°C-4°C) with EDTA, Sodium Citrate, ACD, Sodium Fluoride / Potassium Oxalate, and Sodium Heparin anticoagulants.
- Blood samples are stable after collection up to 8 hours at room temperature (up to 35°C) in EDTA anticoagulant containers.
- Blood samples are stable after collection up to 24 hours at room temperature (up to 35°C) with Sodium Citrate, ACD, Sodium Fluoride / Potassium Oxalate, and Sodium Heparin anticoagulants.
- Frozen blood samples are not supported.

Calibration and Quality Control Procedures

You can verify that the device (including Reader, cartridge, and fluids) is operating correctly after initial setup by running the Hb Variant Test with a sample of known composition, e.g. a fresh or appropriately stored human blood sample of Hb AA. The Hb Variant Cartridges do not require calibration. Hb Variant Marker Fluid serves as a control for each Hb Variant Test.

If you encounter a problem during this verification process, please see "Where to Go for Help".

Performance Characteristics

	DISEASE* VS. NORMAL***	DISEASE* VS. TRAIT**	TRAIT** VS. NORMAL*
True Positive	76	76	54
True Negative	458	54	458
False Positive	0	0	7
False Negative	0	0	0
Sensitivity	100%	100%	100%
Specificity	100%	100%	98.5%

* Disease: HbSS, HbSC, β-thalassemia major/intermedia
**Trait: HbAS, HbAC, HbA/β-thalassemia
***Normal: HbAA

Reference Method: HPLC

Gazelle quantification with HPLC = 99.5%

Repeatability was demonstrated using four different anticoagulants on one cartridge lot, one buffer lot, and one marker fluid lot, by one User across four days of testing.

Limit of Detection

Hb TYPE	LIMIT OF DETECTION (LOD)	ACCURACY
A	4%	100%
S	4%	100%
F, A2/C/E	Not Measured	-

NOTE: 100% accuracy

Limitations

- Some hemoglobin types with similar electrophoretic mobilities, and those occurring in small proportions, must be detected or differentiated by methodologies other than the Gazelle Hb Variant Test.
- The Gazelle Hb Variant Test may not be accurate for babies born before a 37-week gestation period. This test should be delayed until the baby's age plus the gestion period reaches 37 weeks or more.
- Gazelle should not be used to test children less than 6 months of age for beta-thalassemia. If the entered age is 6 months or less Gazelle does not check for beta thalassemia.
- Hb A2/C/E comigrate. Hb A2 is measured in combination with Hb C and Hb E, and therefore cannot be measured if Hb C or Hb E is present. In determining the presence of beta thalassemia trait, Hb A2 can be measured down to 2%
- Test results should be used in conjunction with other clinical laboratory findings and clinical judgement.

Gazelle™ Hb Variant Test Procedure

1 Materials and Patient Information

Before you start, we recommend familiarizing yourself with the testing process. The following steps are recommended:

- Read through these instructions for use.
- Watch a demonstration of the test procedure at https://www.Hemexhealth.com/crc (password: GZLC)
- Try the test with a sample of a known hemoglobin composition.
- If there are problems with the test, please read the troubleshooting section of the onscreen *Gazelle™ User Manual* for possible causes and remedies.

1.1 Ensure that the registered Admin has set up the reader and downloaded any software updates as explained in the *Gazelle™ Setup Guide* that shipped with the reader.

1.2 Gather the materials needed for the Hb Variant Test.

NOTE: The supplied buffer and marker dropper bottles are designed to prevent pipetting directly from the container in order prevent possible contamination. It is recommended that you fill one Eppendorf tube with buffer and the other with marker fluid to use during a day of testing. You will need 450 μL of buffer and 40 μL of marker fluid per test.

⚠ **CAUTION:** Be sure to discard unused fluid from the Eppendorf tubes at the end of the day.

1.4 From the HOME screen, select Hb VARIANT.

1.5 Fill out the patient information screens using the touch keyboard.

NOTE: You will be asked to indicate if the patient is an infant (a child under six months). If you select "YES", you will also be required to enter child's age in months (0-1 month, 1-2 months, etc.)

⚠ **CAUTION:** If the word "DEMO" appears in the PATIENT ID field, the Reader has been set to demo mode. An Admin must go to ADMIN SETTINGS>DEVICE CONFIG to turn off this mode before testing can occur.

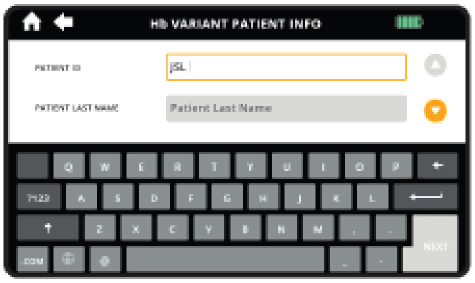

1.6 When prompted, confirm that patient information is correct, and press CONTINUE.

Continued on reverse side

2 Sample Preparation

The following written steps for preparing a sample are designed to accompany the Reader's onscreen video instructions. Each video instruction will repeat until the User is ready to take the next action.

CAUTION: Be sure to invert the blood sample a couple of times before using to ensure the blood is mixed.

Mix Blood with Marker Fluid

2.1 Pipette 40 µL of Gazelle Marker Fluid and 20 µL of blood into an Eppendorf tube (See an illustration of this step in Figure 2.1) and close the lid on the tube.

2.2 Press the Reader's onscreen timer, and vortex the solution in the tube for 20 seconds. (Figure 2.2)

20 After 20 seconds, the Reader's next video instruction will automatically begin.

CAUTION: The Hb Variant Test will automatically cancel if the User does not press the next timer in Step 2.6 within 10 minutes.

Wet Cartridge Paper with Buffer

2.3 Open an Hb Variant Cartridge (figure below) from the Hb Variant Test Multipack.

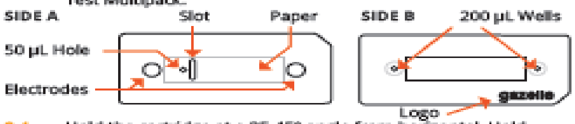

2.4 Hold the cartridge at a 35-45° angle from horizontal. Hold the pipettor at 90° to the cartridge and in one smooth motion, slowly pipette 50 µL of Gazelle Buffer into the round hole next to the cartridge slot. (Figure 2.4)

CAUTION: For best results, ensure that the buffer solution has soaked into the entire width and length of the cartridge paper strip by rocking the cartridge from side to side as the buffer runs down the paper. Proceed immediately to the next step just before the buffer reaches the blotter at the end of the paper.

You can watch a video on the paper wetting process at: https://www.HemexHealth.com/crc (password: GZLC)

2.5 Insert the cartridge into the stamper stand with the cartridge's notched corner facing up. (Figure 2.5)

2.6 Press the onscreen START TIMER button, and do not touch the cartridge for 60 seconds.

60 After 60 seconds, the Reader's next video instruction will automatically begin.

CAUTION: The Hb Variant Test will automatically cancel if the User does not press START TEST in Step 3.1 within 5 minutes.

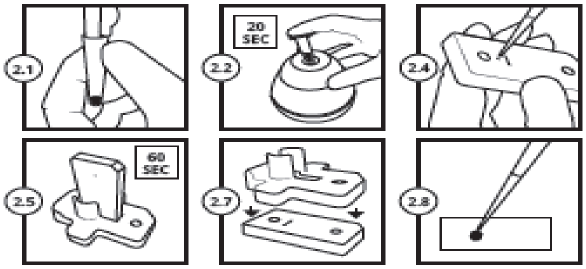

Apply the Blood Sample to Cartridge

2.7 Lay the cartridge flat with the slot side up. (Side A in the diagram). Place the stamper stand directly over the cartridge. (Figure 2.7)

2.8 Pipette 20 µL of the blood and marker fluid mixture onto a glass slide. (Figure 2.8)

WARNING: Although the glass slide may be used throughout a day of tests, it should be disposed of in a sharps container at the end of the day.

2.9 Gently touch the corner of a new stamper to the mixture until the stamper tip is filled from end to end. (Figure 2.9)

CAUTION: Do not immerse the stamper into the mixture!

2.10 Gently place the stamper with blood and marker fluid mixture into the slot on the stamper stand. (Figure 2.10)

2.11 Place a finger on each side of the stamper stand and hold it down for five seconds. DO NOT press on the stamper directly. (Figure 2.11)

To watch a video about the stamping process, go to: https://www.HemexHealth.com/crc (password: GZLC)

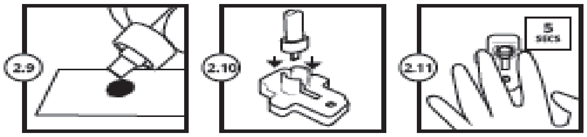

2.12 Press CONTINUE on the Reader to proceed to the next video instruction.

Fill Wells and Load Cartridge

2.13 Remove the stamper and stamper stand from the cartridge.

2.14 Flip the cartridge over to SIDE B with the notched corner facing you, and pipette 200 µL Gazelle Buffer into the left well first. (Figure 2.14) Next, pipette 200 µL of buffer into the right well. For best results, angle the pipette toward the center of the cartridge as shown in Figure 2.14.

To watch a video about the well filling process, go to: https://www.HemexHealth.com/crc (password: GZLC)

2.15 Taking care not to spill the fluid in the cartridge, use one hand to lift and hold the lid of the Reader open, and the other to insert the cartridge into the chamber on the LEFT. (Figure 2.15)

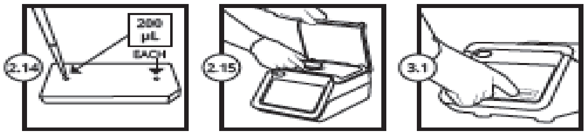

3 Test Operation and Results

3.1 Close the lid and press START TEST on the screen. (Figure 3.1). The test takes about 8 minutes to complete. A video showing the progress of the blue marker and the movement of hemoglobin type[s] will appear on the screen along with an progress bar to display the remaining test time.

3.2 The Hb VARIANT RESULTS screen will show a picture of the hemoglobin bands representing specific hemoglobin types and the relative percentage of each.

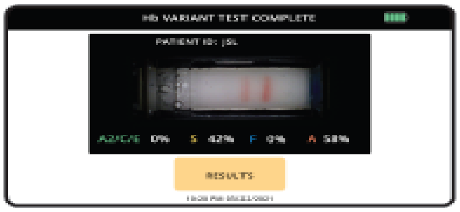

3.3 Press NEXT to go the Hb VARIANT SUMMARY screen. The identified hemoglobin types and their relative percentages will be shown on the chart. The summary screen will also display an interpretation of the results.

CAUTION: Test results should be used in conjunction with other clinical laboratory findings and clinical judgement.

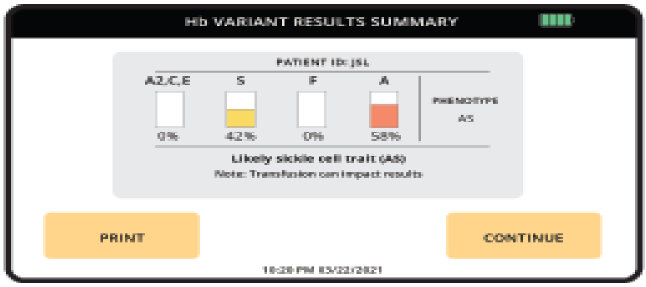

3.4 After the test completes, remove the cartridge from the Reader and properly dispose of the cartridge.

WARNING: Discard the cartridge and stamper as blood contaminated waste after each use.

3.5 If a printer has been configured for the Reader by an Admin, pressing PRINT on the Reader will send a report to the printer.

3.6 Select CONTINUE and NEW PATIENT to be taken back to a new Hb Variant PATIENT INFO screen.

4 RETEST Message or Problems with Test

In the case that the Reader displays a RETEST SAMPLE message or the final result does not show clear readable bands, please try the following:

4.1 Follow the suggestion on the retest message.

4.2 Review the instructional videos at : https://HemexHealth.com/crc (password: GZLC)

4.3 Go to the Troubleshooting Section of the on-screen *Gazelle™ User Manual* to troubleshoot your result .

4.4 Select CONTINUE and RETEST LAST PATIENT to be taken back to the start of the onscreen instructions. You can prepare a new sample without having to reenter information for the patient

Where to Go for Help

Onscreen User Manual

Refer to the onscreen *Gazelle™ Diagnostic Device User Manual*, which can be accessed by going to the HOME>SETTINGS>HELP>USER MANUAL for information about Gazelle menus and error messages.

Disposal of The Waste Materials

After performing the test the materials and cartridges should be discarded to the appropriate colour-coded biohazard bags following the biomedical waste disposal guidelines.

General Procedures For Collection of Venous Blood Samples From Adults And Children

The venous blood samples will be collected from adults and children to test different biochemical markers which reflect abnormal levels of certain substances in the blood. This indicates the disease in the organ or tissue that produces them

Preparation and arrangement of equipment

- Collect all the equipment needed for the procedure and place it on the table.
- A supply of laboratory sample tubes (EDTA tube, gel vacutainer), should be stored dry and upright in a rack. Prior to collecting the blood sample the blood collection tubes.
- Check the barcode labelling in the blood collection tubes, report format and verify the barcode ID before recording the test result.

- Ensure that the rack containing the sample tubes is close to the technician, but away from the patient, to avoid it being accidentally tipped over.

Preparation of the Study Participants

- Before the venepuncture procedure it is important to follow the steps mentioned below:
- Introduce yourself to the participant, and ask him/her to state their full name.
- Ask whether the participant has allergies, phobias or has ever fainted during previous injections or blood draws.
- Discuss the test to be performed and obtain verbal consent. The participant has the aright to refuse a test at any time before the blood sampling, so it is important to ensure that the participant has understood the procedure.

Site Selection For Sample Collection

- Extend the participant’s arm and inspect the antecubital fossa or forearm.
- Locate a vein of a good size that is visible, straight and clear.
- The median cubital vein lies between muscles and is usually the easiest to puncture. Under the basilic vein run an artery and a nerve, so puncturing here runs the risk of damaging the nerve or artery and is usually more painful.
- DO NOT insert the needle where veins are diverting, because this increases the chance of a haematoma.

- The vein should be visible without applying the tourniquet. Locating the vein will help in determining the correct size of the needle.
- Apply the tourniquet about 4–5 finger widths above the venepuncture site and re-examine the vein.

Perform hand hygiene and put on gloves

- Perform hand hygiene by using hand sanitizer.
- If hands are visibly contaminated, use soap and water for cleaning and dry using a clean towel.
- After performing hand hygiene, put on well-fitting gloves.

Disinfect the entry site

- Clean the site with a 70% alcohol swab for 30 seconds and allow drying completely.
- Apply firm but gentle pressure. Start from the centre of the venepuncture site and work downward and outwards to cover an area of 2 cm or more.
- Allow the area to dry. Failure to allow enough contact time increases the risk of contamination.

DO NOT touch the cleaned site; in particular, DO NOT place a finger over the vein to guide the shaft of the exposed needle. It the site is touched, repeat the disinfection

- Blood Draw

Venepuncture

Perform venepuncture as follows:

- Anchor the vein by holding the participant's arm and placing a thumb BELOW the venepuncture site.
- Ask the participant to form a fist so the veins are more prominent.
- Enter the vein swiftly at a 30-degree angle or less, and continue to introduce the needle along the vein at the easiest angle of entry.
- Once sufficient blood has been collected, release the tourniquet BEFORE withdrawing the needle. Some guidelines suggest removing the tourniquet as soon as blood flow is established, and always before it has been in place for two minutes or more.
- Withdraw the needle gently and apply gentle pressure to the site with

a clean gauze or dry cotton wool ball. Ask the patient to hold the gauze or cotton wool in place, with the arm extended and rose. Ask the patient NOT to bend the arm, because doing so causes hematoma.

Figure : Venepuncture Process

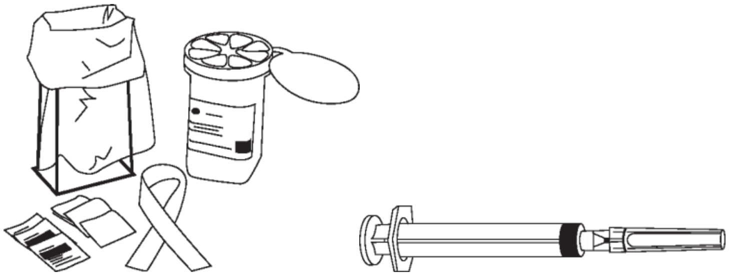

1. Assemble equipment and include needle and syringe or vacuum tube, depending on which is to be used.

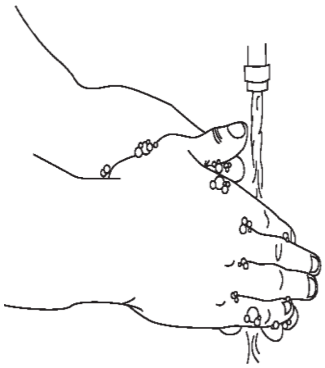

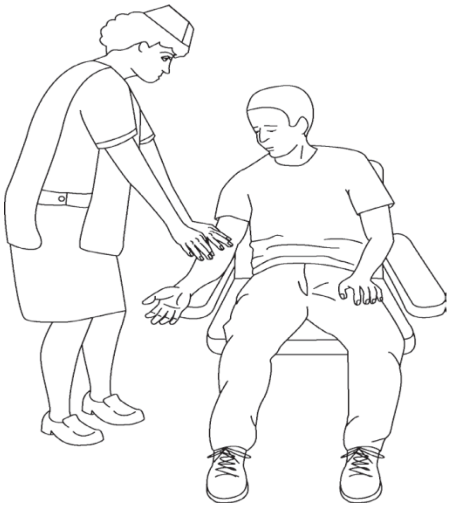

2. Perform hand heigyne (Use soap and water/Sanitizer)

3. Identify and Prepare patient

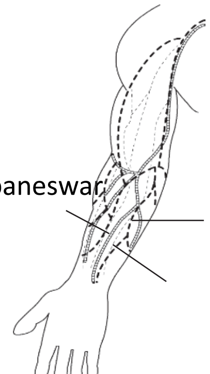

Median cubical vein

Ulner nerve

Ulner artery

Basilic vein

4. Select the site, preferably at the antecubital area (i.e. the bend of the elbow). Warming the arm with a hot pack, or hanging the hand down may make it easier to see the veins. Palpate the area to locate the anatomic landmarks. DO NOT touch the site once alcohol or other antiseptic has been applied.

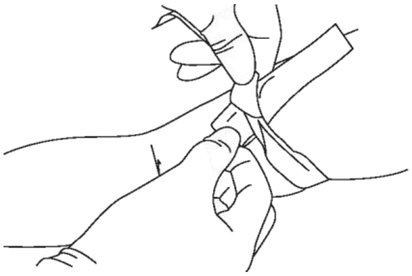

1. Apply a tourniquet, about 4–5 finger widths above the selected venepuncture site.

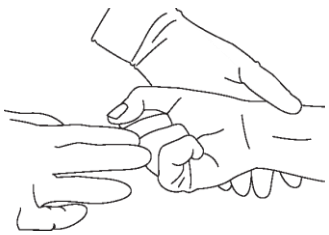

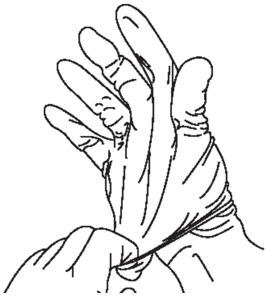

2. Ask the patient to form a fistso that the

7. Put on well-fitting gloves

veins are moreprominent

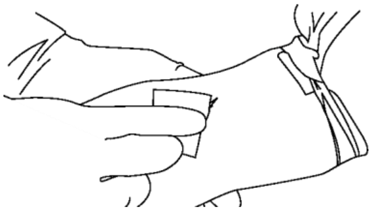

8. Disinfect the site using alcohol swab for 30 seconds andallowtodrycompletely

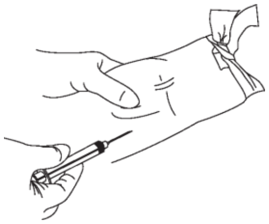

9. Anchor the vein by holdingthepatient'sarm and placing a thumb below the venepuncture site

10.Enter the vein swiftly at a 30° angle

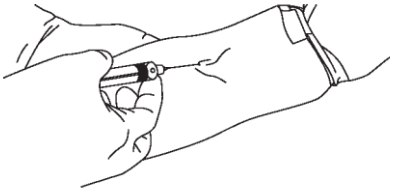

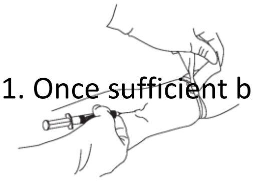

11. Once sufficient blood has been collected release the tourniquet before withdrawing the needle

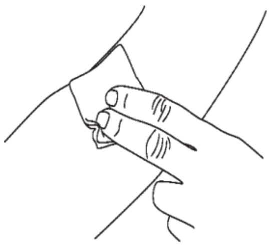

12.Withdraw the needle gently and give the patient a cleanor dry cotton ball to apply to the site with gentle pressure

13. Discard the needle and syringe into a sharp container/burn the needle in needle destroyer

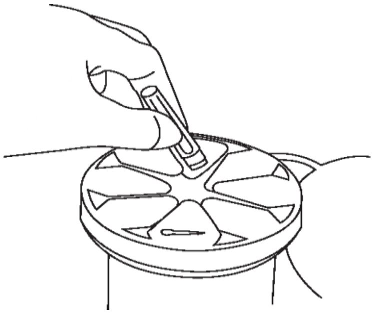

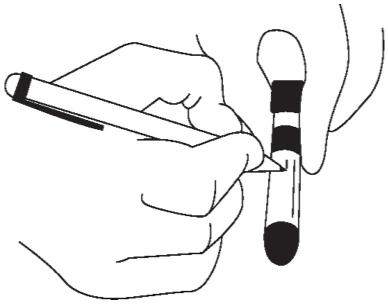

14.Check the label and form for accuracy

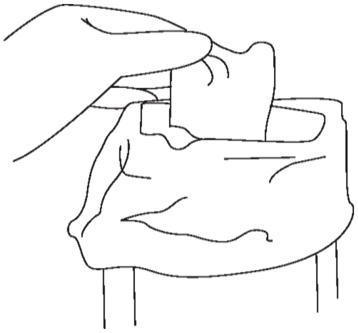

15. Discard sharp and broken items in to the sharp container. Place items that can drop blood/body fluids in biohazard bags .

16.Perform hand hygiene after removing the gloves

(**Picture Source: WHO Phlabotomy Guidance 1**)

Transfer of blood from the syringe to vial

- Pierce the stopper on the tube with the needle directly above the tube using slow, steady pressure. Do not press the syringe plunger because additional pressure increases the risk of haemolysis.
- Where possible, keep the tubes in a rack and move the rack towards you. Inject downwards into the appropriate coloured stopper. DO NOT remove the stopper because it will release the vacuum.

Order of draw for multiple tube collection

- First approximately 1ml blood should be transferred to the EDTA (purple cap) tube for testing sickle cell (less amount because it requires only 20µl blood for the

testing).Then the rest of the blood should be transferred to the gel vacutainer tube(yellow cap) for serum separation.

Cleaning of contaminated surfaces and completion of the procedure

- Burn the used needle using a needle destroyer.
- Check the label on the blood collection tube for accuracy.
- Discard used items into the appropriate category of waste. Items used for phlebotomy thatwould not release a drop of blood if squeezed (e.g. gloves) may be discarded in the generalwaste.
- Performhandhygieneagain, as described above.
- Inform the participant when the procedure is over.
- Ask the participant how they are feeling. Check the insertion site to verify that it is not bleeding, then thank the patient and say something reassuring and encouraging before the person leaves.

Sample storage In Field

All the collected blood samples will be stored in the vaccine carrier immediately after collection maintaining the cold chain.

Coss Checking with Laboratory Tally Sheet

Before transporting all the samples from the field to the district headquarter hospital the laboratory tally sheet should be cross-checked by the laboratory technician (Annexure 4).

Sample transport from the field to the field laboratory

The vaccine carrier should be properly closed and transported to the district headquarter hospital laboratory to maintain the cold chain for further processing.

Roles and Responsibilities of Research Assistant (RA) / Field Investigator (FI) before leaving the Field

- The RA should confirm that the laboratory tally sheet is filled up appropriately by the laboratory technician.
- The report format of all the participants is filled with appropriate measurement and testing data and then it should be handed over to the authorized person of ASHA in the respective village.

- All the instruments are switched off and properly cleaned and packed in their respective bags/boxes before leaving the field.
- In the absence of RA the above task responsibility will be performed by the FI of the respective team.

Sample processing set Up in District Head Quarter Hospital for primary sample processing

- The sample processing set-up will be performed in the assigned laboratory space by the District Health Authority.
- After reaching the laboratory the RA should contact District Microbiologist for the coordination of sample processing.

Materials and Equipment Required:

- REMI clinical centrifuge
- Cryo vials
- Barcode Stickers
- Pasteur pipette
- Table top waste disposal container/Biohazard Bag
- Tissue paper
- Sanitizer
- Cryo vial box
- Laboratory tally sheet

Blood Sample Processing for Serum Separation

It is important to separate the cellular and liquid portions of a blood specimen as soon as possible when the test requires a sample of serum or plasma. Centrifugal force is used to separate the components of blood like red blood cells, platelets and plasma from each other.

General Procedure for Serum Separation

- Before processing all the collected blood samples must be matched with a laboratory tally sheet.

- Assemble the required materials needed to perform the serum separation.
- Wear personal protective wear like a mask, gloves, lab coat before processing the sample.
- For serum separation (requires clot time): Whole blood should be allowed to clot and then centrifuged at 2800 rpm for 10 minutes to separate the serum.
- Arrange a rack with sterile cryo vial and label the vials with patient code, date of collection and sample type.
- The serum should be carefully removed with a Pasteur pipette to avoid extracting red cells, and transferred aseptically to the pre labeled sterile cryo vial.
- All the serum containing cryovials will be kept in pre labeled (serial no of cryovials containing sample id, sample type, date of storage, box no) cryo box and stored.
- **Note: Universal Precautions must be used when working with blood. Use of personnel protective equipment is mandatory**.
- Discard the vacutainer in biohazard bag after separating serum/plasma.

Storage of samples in field laboratory

After centrifugation the separated serum will be stored barcode labeled cryo vials in -20°C in the cryo cube boxes serially as per the lab tally sheet until they are transported to RMRC laboratory.

Waste Disposal in the Laboratory

Waste disposal will be done at the sample processing laboratory as per the laboratory SOP instructed by District Microbiologist.

Check Points Before Leaving the Laboratory

Before leaving the laboratory make sure the three steps are done:

- The centrifuge is switched off and the plug is removed.
- The laboratory tally sheet is filled up after serum separation.
- Any spillage on the working table should be cleaned with 70% alcohol.
- Gloves, Tissue paper, used Pasteur pipettes are disposed on biohazard bags.
- Cryo vials are properly labelled with barcode and the barcode is covered in transparent cellotape.

- The samples are stored in proper temperature.
- Gel cool packs are kept in freezer for cooling so that they are frozen while carrying next day to field.
- Inform the District Microbiologist before you leave the laboratory.

Roles and Responsibilities

The primary sample processing and storage of secondary sample (serum) in the laboratory will be performed by LT1 or LT 2 under the supervision of RA/FI.

Sample transport to RMRC laboratory at Bhubaneswar

The serum/plasma samples will be transported from field laboratory to RMRC laboratory by following a triple layer packaging system.

- 1st packaging: after centrifugation the serum/plasma sample will be stored in a labeled cryo vial.
- 2nd packaging: the cryovials will be stored in -20°C in the cryo cube boxes serially as per the lab tally sheet.
- 3rd packaging: the cryo boxes will be further packed in thermocol boxes with gel cool packs and sealed properly.
- The samples should be transported to RMRC Bhubaneswar from each district at least once in a month.

Quality Assurance During Sample collection, Processing, Storage and Transport

- Hand hygiene should be performed before and after collecting the blood samples.
- After collecting the sample the labeling on the collection tube should be matched with the laboratory tally sheet.
- Any adverse event while collecting the sample should be recorded immediately. A log book or a registrar should be maintained for documenting the incident, its possible causes and management.
- The needle stick injury should be reported by the field staff to the higher authority immediately and recorded in a log book mentioning the incident, date and time.

- All infectious waste should be disposed maintain the Biomedical Waste Management guidelines **(Refer BMWM SOP)**
- The storage of the samples should be done maintaining adequate temperature (For blood sample 0-4°C and for serum -20°C).
- Before transport the samples and the cryo boxes should be checked thoroughly for proper labeling, sealing and must be crosschecked with laboratory tally sheet.
- The transport box must be filled with adequate gel cool packs to maintain the cold chain. After packaging the samples the transport box should be marked with waterproof labeling and sealed properly to maintain the cold chain inside.

Roles and Responsibilities

- The packaging of the serum samples to be transported to RMRC Bhubaneswar will be done by RA and FI.
- LT 1 and LT 2 will assist them for the entire task.
- The field staff (RA and FI) will be responsible for packaging and transporting of the samples to RMRC laboratory. They should thoroughly check that the cryo cube boxes are properly labeled, cyovials are arranged in the cryo cube box in a serial manner maintaining the laboratory tally sheet and should not leak, the transportation box is properly labeled and sealed with adequate ice pack or gel cool packs to maintain the cold chain till it reaches to RMRC laboratory.
- The RA/FI should maintain documents (photos, videos) while packing the samples and record the time when the samples are dispatched.

Biomedical Waste Management

1. Introduction

Biomedical Waste, (BMW) or bio wastes are those potential hazardous waste materials, consisting of solids, liquids, sharps, and laboratory waste. Biomedical waste differs from industrial waste. It is mostly from the biological sources or is

used in the diagnosis, prevention, or treatment of diseases. The Bio Medical Waste (Handling and Management) Rules, were notified in July 1998.

Health Hazardous of Biomedical Waste

The types of waste generated from different health care sectors can impact a higher risk to health. These include infectious waste (15%–25% of total health-care waste), among which are sharps waste (1%), body part waste (1%), chemical or pharmaceutical waste (3%), and radioactive and cytotoxic waste or broken thermometers (less than 1%).

2. **Types Of Waste**

Waste and by-products cover a diverse range of materials. It can be categorized into hazardous and non hazardous waste.

- **Hazardous Waste**
- Infectious waste: waste contaminated with blood and other bodily fluids (e.g., from discarded diagnostic samples), cultures and stocks of infectious agents from laboratory work (e.g., waste from autopsies and infected animals from laboratories), or waste from patients in isolation wards and equipment (e.g., swabs, bandages, and disposable medical devices).
- Pathological waste: human tissues, organs or fluids, body parts, and contaminated animal carcasses.
- Sharps: syringes, needles, disposable scalpels and blades, etc.
- Chemicals: For example, solvents used for laboratory preparations, disinfectants, and heavy metals contained in medical devices (e.g., mercury in broken thermometers) and batteries.
- Pharmaceuticals: expired, unused, and contaminated drugs and vaccines; Genotoxicwaste: highly hazardous, mutagenic, teratogenic, or carcinogenic, such as cytotoxic drugs used in cancer treatment and their metabolites.
- Radioactive waste: such as products contaminated by radionuclides including radioactive diagnostic material or radiotherapeutic materials.

- **Nonhazardous or general waste:**
  Waste that does not pose any particular biological, chemical, radioactive, or physical hazard.
- General and office waste: wrapping paper, office paper, cartons, packaging materials including plastic sheets, newspapers and bouquets etc.

- Kitchen waste: leftover food, peels of fruits and vegetables skin and dirty water.

3. Method Of Waste Management

- **Waste Minimization:** the production of waste more than requirement is always non acceptable.
- **Segregation:** waste should be segregated in different streams at the point of generation. Segregation at source helps in reducing total cost of disposal, preventing general waste from infectious and reducing chances of infection among health care workers. The segregation of wastes is done as per the guidelines laid by Authorized agency of pollution control board and is defined below:

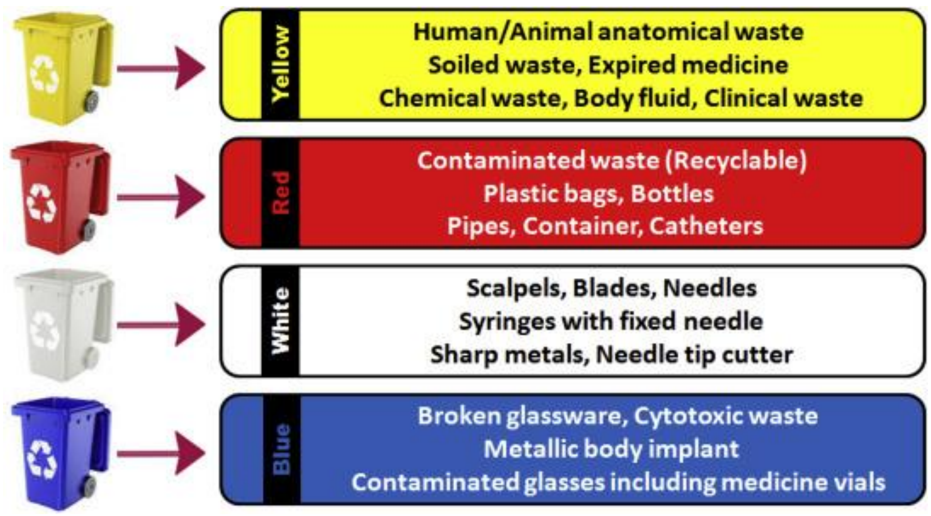

Figure I: Different colour codes for disposal of infectious waste

- **Handling and Storage of Bags**
- **Handling:**
  - Bags must be replaced when atleast three fourth is full.
  - It should be sealed properly.
  - The sealed bag should be picked up by its neck.
  - Manual handling should be minimized.
  - Bags should not be clasped against body.
  - Sharp containers should be handled carefully.

- Support from the bottom of the bag should be avoided.

- **Storage:**
  - A safe and secured location for storage of segregated biomedical waste in colored bags or containers should be available.
  - Secondary handling, recycle, scattering or spillage by animals should not occur.
  - Biomedical waste shall not be stored beyond a period of forty eight hours from its generation.
  - A system of bar-code and global positioning system for bags or containers.

➢ Treatment of Biomedical waste inside the laboratory premises

- All the infectious waste of the Laboratory are autoclaved before disposal.

4. Important Personnel Precautions To Be Taken During Waste Handling

Proper training has to be provided to all the staff involved in segregation and storage of waste. They should also receive training regarding universal safety precaution. The use of protective equipment should be mandatory for all the personnel handling waste. It can be categorized into two categories: biosafety practices and immunization for the health care workers.

➢ **Bio safety practices**

- **Gloves**

  Heavy duty rubber gloves should be used for waste handling by the waste handlers. The gloves should be washed with soap and disinfectant twice during the handling of the waste (first while wearing and second after removing it). It should be replaced whenever it is teared otherwise can be replaced monthly. The gloves should be kept in hung position whenever not in use.

- **Mask**

  Waste handlers should wear mask while sweeping and handling waste.

➢ **Immunization of the health care worker and waste handler to prevent disease transmission**

All the health care workers and waste handlers whose activities involve contact with patients or with blood/ body fluids (Blood,CSF, Amniotic fluid, Pleural fluid, peritoneal fluid and other body fluids) required for immunization against Hepatitis B vaccine, Tetanus toxoid vaccination and Covid-19 vaccination. Health check up should be conducted during induction and at least once in a year for all these people.

Management of SPILLS

- Small Spills (< 10 ml) Cordon the area.
- Bring the spill kit.
- Wear the PPE.
- Cover the spill with tissue paper.
- Pour 1% hypochlorite on the tissue paper.
- Leave it for 15 minutes.
- Pick up the soaked paper with artery forceps and dispose it in yellow bag.
- Mop the are again with 1% sodium hypochlorite.
- Remove the PPE and discard in appropriate bags.
- Replace the spill kit box and keep in designated place.
- Large Spills (>10 ml) Cordon the area.
- Bring the spill kit.
- Wear the PPE.
- Cover the spill with tissue paper.
- Pour 1% hypochlorite on the tissue paper Leave it for 30 minutes.
- Pick up the soaked paper with artery forceps and dispose it in yellow bag.
- Mop the are again with 1% sodium hypochlorite.
- Remove the PPE and discard in appropriate bags.
- Replace the spill kit box and keep in designated place.
- Report the spill incident to the infection control department.
-

Infection Prevention & Control

INTRODUCTION

Infection is the invasion of a host organism's bodily tissues by disease causing organism, their multiplication and the reaction of host tissues to these organisms and the toxin they produce. Infections which arise in healthcare are termed Healthcare associated infection (HAI).In healthcare settings, germs are found in many places. People are one source of germs including:Patients**,** Healthcare workers**,** Visitors and household members.Infection control prevents or stops the spread of infections in healthcare settings. These practices include standard precautions, decontamination, waste management, surveillance and audit.

1. PURPOSE OF INFECTION PREVENTION AND CONTROL

- To ensure establishing a safe procedure of collection of samples from participants.
- To establish standards in prevention, control measures and minimize infection among the field staff and laboratory staff.
- To define procedures for infection prevention and control and to implement them in field as well as in laboratory.

2. INFECTION CONTROL PROCEDURE AND PRACTICE

According Centre of Disease Control (CDC) the procedures and practices for infection prevention include:

- Hand hygiene
- Use of personal protective equipment (e.g., gloves, gowns, facemasks), depending on the anticipated exposure
- Respiratory hygiene and cough etiquette
- Management of spillage

➢ **Hand Disinfection-Aseptic Hygeine Hand Wash**

Hand disinfection with alcohol based hand rub (e.g., 70%alcohol, sterilium) preferably with chlorhexidine and alcohol are practice at least in following condition:

- Whenever touching any patient esp. in inpatient units and critical care areas.
- After handling any potentially infectious object
- Before putting on gloves and after removing them.
- Prior to invasive procedures
- Visibly clean hands

- In high dependency areas and after attending patients in isolation or with known transmissible condition.

Broken skin, cuts and abrasions in any area of exposed skin, particularly the hands and forearms, are covered with a waterproof dressing. Wear gloves if hands are extensively affected. Wrist watches/bracelets are removed.

Alcohol is an effective decontamination agent but should only be used on visibly clean hands. It is also a valuable agent for use, but should only be used 2-3 times consecutively before a hand wash as build up can occur.

- Dispense the required amount of solution onto the hands.
- Ensure solution covers all hand surfaces.
- Rub vigorously, using hand washing technique, until dry.

It is recommended that everyone involved in providing healthcare in the community must be trained in hand decontamination, the use of protective clothing and safe disposal of sharps, and this includes patients and healthcare personnel.

➢ Hand Care

- Keep nails clean and short.
- Remove rings with stones or ridges.
- Do not wear artificial or gel nails or nail polish.
- When washing hands, wrist watches are removed.
- Sleeves are rolled up to the elbow.

- Nailbrushes should not be used for routine hand washing as they damage the skin and encourage shedding of cells.
- Nailbrushes, where used, must be single use disposable or single use autoclaveable.

The physical action of washing and rinsing hands under such circumstances is recommended because alcohols, chlorhexidine, iodophors, and other antiseptic agents have poor activity against spores.

➢ Hand-hygiene Technique

When decontaminating hands with an alcohol-based hand rub

- Apply product to palm of one hand and rub hands together,
- Cover all surfaces of hands and fingers by six step technique, **until hands are dry**.

- Follow the manufacturer's recommendations regarding the volume of product to use.

When washing hands with soap and water

- Wet hands first with water
- Apply an amount of product recommended by the manufacturer to hands
- **Rub hands together vigorously for at least 40-60 seconds**
- Cover all surfaces of the hands and fingers by following six step technique
- Rinse hands with water and,
- Dry thoroughly with a **disposable towel/Paper**
- **Use sterile paper towel to turn off the faucet or elbow taps if available**.

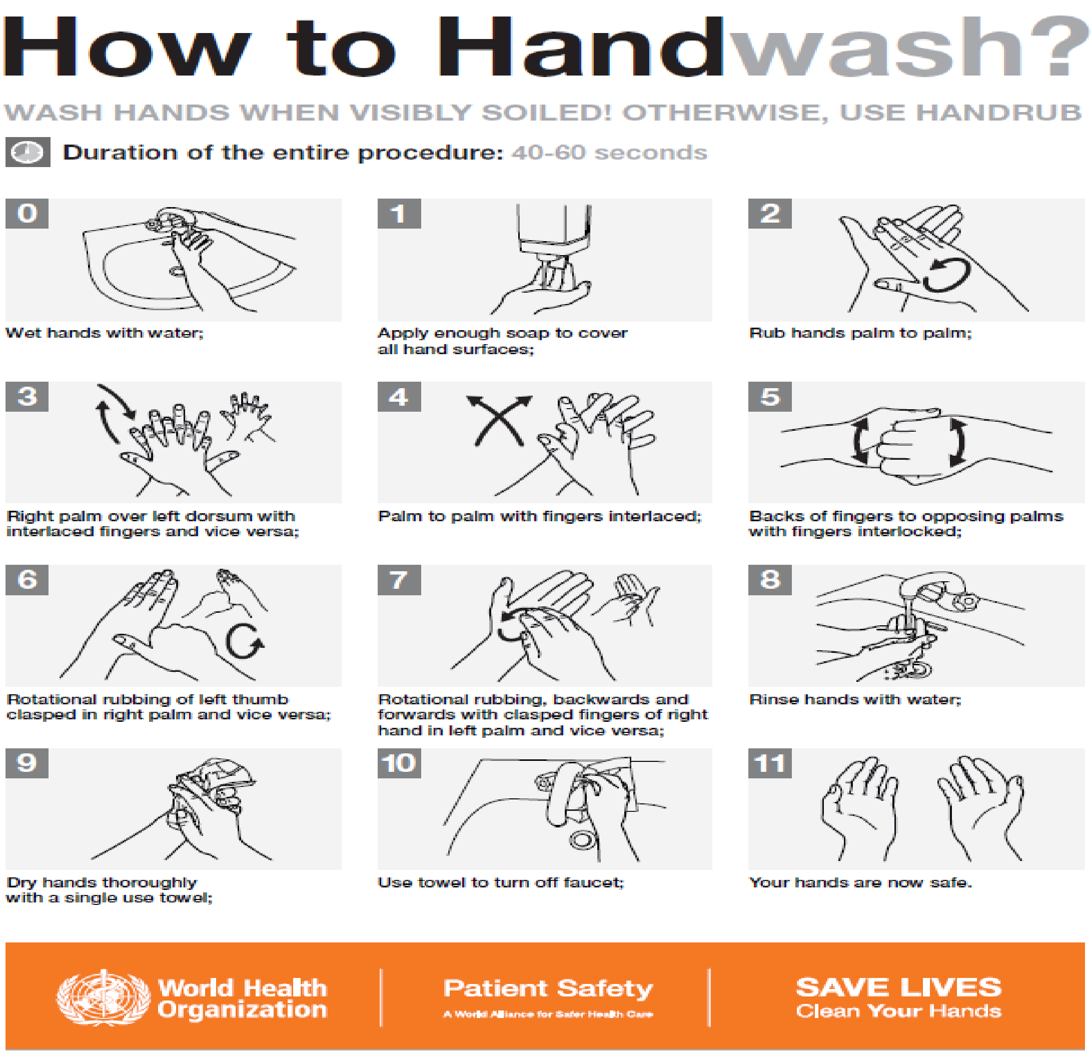

- Re-use/reprocessing of gloves
- As medical gloves are single-use items, glove decontamination and reprocessing are not recommended and should be avoided, even if it is common practice in many health-care settings with low resources and where glove supply is limited.
- At present no standardized, validated and affordable procedure for safe glove reprocessing exists.
- Every possible effort should be made to prevent glove reuse.

3.2 Personal Protective Equipment (PPE)

- In determining the type of personal protective equipment to use for a given procedure, the field and laboratory staff should consider the following factors:
- Probability of exposure to blood and body substances;
- Amount likely to be encountered;
- Type of body substance involved; and
- Probable route of transmission of infectious agents
- Full protective wear, including double gloves, protective eye/face-shields, protective footwear and impermeable gowns or aprons, is recommended for operating room or mortuary procedures.
- Gloves

The use of disposable gloves is part of the Standard Precautions concept, which offers consistent guidelines for infection control programmes. As part of personal protective equipment, gloves prevent contact with blood, body fluids, and mucous membranes. They also protect the patient from contamination by the micro-organisms from the wearer's hands; **gloves are single use items** and are changed after each procedure to further minimize the risk of infection

- Masks
- The type of mask best suited to a particular situation depends on the body substances likely to be encountered and the nature of the activity.

There are two main types of masks used in health care:

- **Surgical masks** — fluid-repellent paper filter masks worn during surgical and dental procedures
- **Particulate filter personal respiratory protection devices (P2 respiratory protection devices)** —close fitting masks capable of filtering 0.3-µmparticles and worn when attending patients requiring airborne precautions.
- use.
- Protective foot wear

Protective foot wear should be used when handling biomedical waste as unnoticed cuts and wounds are quite common in the legs. Footwear is also essential to protect legs from 'sharps' injury.

STERILIZATION

STERILIZATION

Sterilization is defined as a process where all microbes are removed from a defined object, inclusive of bacterial endospores**.**

Methods:

- **Heat Sterilization:**
- **Moist Heat:** Exposure to saturated steam at 121ºC for 15-20 min OR 134ºC for 4 min in any autoclave.

Recommended Practice:

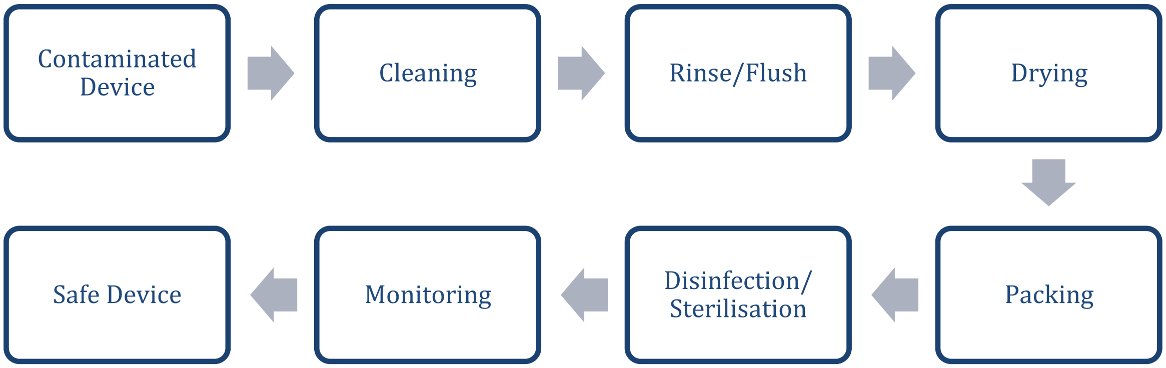

- Packing & Loading

For effective sterilization, selection of packaging material plays important role apart from sterilization parameters. The following are keys in selecting a suitable packaging material:

- The packaging material must be permeable to sterilizing agent.
- The packaging material must be impermeable to bacteria and other contaminants.
- The packaging material must resist tears and punctures.
- It should facilitate aseptic presentation of packaged content.

Mop travel

Vendors for providing vehicles have to be fixed at all the district headquarters where the field teams will be stationed.

Points to be taken into consideration while fixing the vendor:

- Identification of local vendors already engaged in Govt. Projects. (For ease in tendering process)
- 7 seater vehicle needs to be hired for a period of 6-12 months at each field station.
- The vehicle has to report for duty whenever asked to; on all working days.
- Floating up of tendering process in the website of ICMR-RMRC-BB and local newspapers.
- Tendering procedure for the same has to be completed before the field team is being stationed.
- The rates once fixed will be deemed valid for 1 (one) year WEF. date of issue of letter.
- Toll gate & parking charges (extra) will be bear by the party on submission of the bills at the end of the month.
- GST charges (Extra) will be paid by the party as per norms.
- ICMR-RMRC, Bhubaneswar is not responsible for any consequences or damage or liability during the period of hiring of the vehicle.
- The payment will be made within thirty days after submission of the bills.
- The bills should be duly certified by the Principal Investigator, field worker along with duty slip.
- Rate chart strictly followed by the approval rate of competent authority of the ICMR-RMRC, Bhubaneswar.
- Log book of the tour should be maintained properly and duly signed by the traveller and will be submitted to the office for record.
- Up to date vehicle documents like RC Book, Insurance, Fitness, Valid License of the driver, Pollution certificate etc. Should be available in the vehicle.
- Vehicle should always be kept neat and clean.

- The tour program for day/ monthly basis will be communicated by this centre to you for providing vehicle from time to time.
- No fooding and lodging charges will be claimed during the tour.
- All expenses including fuel bills will be paid to the agency by ICMR-RMRC, Bhubaneswar on submission of the bill by the agency at the end of each month.
- All bills will be cleared with due verification by competent authorities ICMR-RMRC, Bhubaneswar.
- Details of Bank Account No., Name of the Bank, Branch Code, IFSC Code and a cancelled cheque along with bills.
- Valid PAN/GST No. should be provided for office record.

STATION OF LOCAL VEHICLE VENDORS	
**1ST PHASE**	**2ND PHASE**
BARIPADA	SAMBALPUR
SUNDARGARH	PARLAKHEMUNDI
RAYAGADA	MALKANGIRI
KORAPUT	NOWRANGPUR
BHAWANIPATNA	PHULBANI
KEONJHAR	

Mop administration

On Joining

- Required to furnish an undertaking to the effect that no criminal proceedings are either pending or contemplated against the staff in any court of law.
- The engagement is purely on contract basis and the Competent Authority reserves the right to dispense with the contract at any time without assigning any reasons.
- The present assignment is initially for (6 months/ 1 year) from the date of assumption of duty unless subsequently extended.
- The engagement to the position will automatically cease to be on present/extended assignment or completion of the project, whichever is earlier.
- The engagement can be terminated at any point in time by giving one month notice on either side.
- Contract can be terminated forthwith or before expiry of notice period by making payment of a sum equivalent to one month contractual amount. However, the staff will not be permitted to surrender one month contractual amount in lieu of the period of notice of unexpired portion thereof and the staff is required to serve the full period of notice.
- The staff will not be treated as an regular employee of ICMR-RMRC Bhubaneswar.
- The staff will not have any claim on a regular post in ICMR or its institute/ centre or in any Dept of GoI. The staff will give an undertaking to this effect before joining the Project Human Resource Position.

- The staff will be under the administrative control of ICMR-RMRC Bhubaneswar and will be subject to all the rules of ICMR-RMRC Bhubaneswar for Project Human Resource Position.
- The staff will be normally posted at the study site; however maybe temporarily be asked to work at other study sites in the interest of the project work. The staff shall be liable to serve any part of the country.
- The staff is not entitled for any terminal benefit after completion of contract period or otherwise.
- The staff shall not apply for any other outside employment before completion of first six months period under the project.
- After completion of the first six months period under the project, not more than four applications for outside employment shall be forwarded in a year. All applications shall be sent through proper channel not directly.
- The staff has to submit a certificate of physical fitness from civil surgeon/ medical superintendent of a Government Hospital; if fails to submit the same or found unfit in medical examination, the offer to engagement to the Project Human Resource position shall stands cancelled automatically.
- The staff will not divulge any information gathered or outcome of research work during the period of assignment to anyone who is not authorized to have the same.

On working, Attendance & Payments

- 6 Days a Week Working (Monday to Saturday).
- Sundays and Public Holidays are Off.
- Attendance Register Need to be Signed Everyday; which will be with the RA/ PI.

- Work certificate has to be sent to the central team by 18th of each month from the field; as per norms for salary.
- Work certificate of central team has to be compiled by 20th of each month along with the field team and be sent to the accounts section for the needful.
- Payment of stipend or emoluments to the Project Human Resource positions will be subject to availability of funds from the funding agency of the project.

Leave rules as per norms.

- CCC (Leave) Rules shall not be applicable.
- Paid leave of absence may be allowed at the rate of 2.5 days for each completed month of engagement or as prescribed by the competent authority from time to time. No other kind of leave shall be admissible.
- Leave shall not be carried forward beyond one-year contract. Accumulation of leave beyond a calendar year shall not be allowed.
- On termination of the contract, you shall not be entitled to benefit of encashment of unveiled portion of leave.
- In event of leave without prior permission of PI/ Guide/ Head of Institute and/ or any willful unauthorized absence, the contract shall be ceased automatically and the concerned person holding Project Human Resource Position will not be permitted to resume duty without prior permission of Head of the Institute.

Allowances

- Project Human Resource staffs are not entitled to any other allowance such as dearness allowance, transport allowance, LTC, Bonus, etc.
- They will not be provided any medical facility under CGHS or CS (MA) Rules.
- No travelling and/or daily allowance will be admissible either for joining the assignment or on expiry of the contract.
- Travelling in connection with assigned work during the period of engagement, staff will be entitled to draw TA/DA in accordance with emoluments, but it will not be at par with regular/ permanent employees of ICMR.
- Eligibility for availing TA/DA as per norms.
- Tour Approval as per norms.

- Logistics consumption register has to be maintained on day to day basis.
- Indent for the regular supply of consumables has to be made well in advance for uninterrupted supply of materials.
- Any malfunctioning of instruments has to be reported immediately; so that it can be addressed at the earliest possible.
- Vehicle movement log book has to be maintained on day to day basis.

Mop roles & responsibilities of staff

RESPONSIBILITY OF ICMR-RMRC, BHUBANESWAR

- Implementation of the project as per the approved timeline
- Constitute and convene meetings of a steering committee with representation from health, tribal affairs and other relevant departments/institutions for monitoring the progress of the project.
- The Principal Investigator or a person duly authorized by her will be the contact person for all the compliances.
- The institution will submit phase wise progress report as per the following deliverables:
- Formative phase including project unit set up, staff hiring, data collection tool preparation, data base management system (DBMS) establishment, manual of operating procedures development, procurement of equipments.
- Midpoint of data collection report
- Final report.
- ICMR-RMRC, Bhubaneswar will obtain necessary Ethical Committee approvals before implementation of the project.
- The institution will submit the SOE and UC at the end of the project period.
- The Principal Investigator will submit the soft and hard copy of the final report to SCSTRTI, Bhubaneswar at the end of the project.
- Study findings may be published jointly after due approval from SCSTRTI, Bhubaneswar.
- SO, Micro Project and CDMPHO of the respective districts should be informed about the field visits ahead of the schedule to ensure support by ITDA department.
- The data and the report will be the property of SCSTRTI, Bhubaneswar and ICMR-RMRC, Bhubaneswar will take permission of SCSTRTI for further use as and when needed.

ROLE OF CENTRAL TEAM IN FIELD

- Will be visiting each of the six fields every month for monitoring and cross verification of the data collected.
- Will be helping out field team to solve local issues if any faced.
- Will be helping out field team in training as and when required.
- Will be taking care of the regular supply of consumables to the field team.
- Will be taking care of transport of samples collected from the field to ICMR-RMRC central laboratory.

Programme Manager

- The program manager is responsible for developing the plan which specifies how the projects goals will be met, and distributing it to stakeholders.
- The day to day activities of the project require strong leadership from the project management team.
- The programme manager must initiate changes needed based on field experience and seek approval from the applicable stakeholders.
- Monitoring the project budget and schedule status
- Dividing the project into tasks.
- The programme manager must communicate the expected completion date to the applicable stakeholders, as well as any milestone dates.
- Monitoring schedule progress
- Determining the project team roles and responsibilities: The programme manager must assign job descriptions and determine what skill sets are required to achieve the projects goals with minimal cost.
- Developing the project team
- Motivating the project team
- Co-ordinate the Liaising with district administration and health department.
- Acquiring the project resources: The project resources must be acquired at a reasonable cost, and be available when the project needs them.
- Monitoring and controlling project resources: Many project resources have escalating cost provisions, or get consumed at a planned rate, especially those which are outsourced.

- Preventing unauthorized scope change (scope creep) It is ridiculously easy to let people add things to the project that were never planned, especially small items that appear to be insignificant.
- Identifying stakeholders: The project stakeholders are the judge and jury who determine the success or failure of the project,
- Managing stakeholder expectations
- Communicating with stakeholders: The programme manager must maintain a stakeholder register and perform the required communication to ensure that stakeholders are kept informed and make the necessary decisions.
- Quality control during project execution, the programme manager ensures that the quality level of the end products meets the specifications determined during project planning.
- Identifying risks: All projects have risks which, if they occur, affect the projects success factors. The better these risks are identified, the better they can be anticipated and managed.
- Analyzing risks: Some risks are more important than others. Strong risk analysis ensures the project manager is focused on the things that can trip up a project.
- Developing risk response plans The most important risks should have a risk response plan drawn up

Project Scientist C: Public Health

- Plan the implementation framework of the study with quality assurance through monitoring and supervision.
- Liaise with district administration and health department.
- Sampling framework preparation.
- Carry out pilot studies to check for feasibility of the tool and team.
- Developing the project team
- Motivating the project team
- Monitoring of field data collection.
- Monitoring of DBMS.
- Monitoring schedule progress

- Scientific report preparation.
- Any other responsibilities assigned by PI of the project.

Project Scientist C: Bio-Chemistry

- To Prepare Standard Operating Procedures for different Laboratory Tests.
- To prepare list of laboratory tests to be performed at central lab and in the field.
- To make indent for procurement of consumables and non-consumable items to be used in the Central Laboratory and Field.
- To Set up Central Laboratory Facility at ICMR-RMRC, Bhubaneswar.
- To standardize all the equipments/ instruments.
- To Train the Laboratory Technicians and Research Assistant (Laboratory).
- To carry out Laboratory Tests.
- To monitor sample collection, processing and storage in the field.
- To monitor transportation of samples from the field to the storage facility and from the storage facility to central laboratory at ICMR-RMRC, Bhubaneswar.
- Preparation of the reports of the tested samples.
- To monitor daily uploading of reports in to the Data Base Manage System (DBMS).
- To ensure uninterrupted supply of consumables to the field team and in the central laboratory.
- To perform internal audit for the utilization of the consumables at the field and central laboratory.
- To ensure regular maintenance of instruments.
- To make arrangements for repairing of any equipments/ instrument in case of its break down at the earliest possible.

Project Scientist C: Statistics

- Tool development for data collection.
- Development of tools manual.

- Data base management with cleaning and analysis.
- Error Checks.
- Data Quality Monitoring.
- Real time data quality check.
- Course preparation.
- Raw data preparation.
- Data Analysis.
- Making Standard operating Procedures for Data Quality, Data flow, Data Base Management System (DBMS).
- Use and functionality of DBMS.
- Development of DBMS dashboard indicators and their daily monitoring
- Daily monitoring of DBMS.
- Preparing reports and scientific papers.
- Any other responsibilities assigned by PI of the project.

Field Supervisor

- Implementation of field data collection, training and handholding of the field workers in data collection process.
- Ready to reside at study and Travel extensively in the field area.
- Monitor data collection and perform quality checks.
- Coordination with the team and stakeholders involved in data collection activities.
- Rapport build-up local Govt. Officials and local representatives.
- To Make a directory of all District level, Block Level and village level officials along with field workers (ASHAs/ ANMs etc.
- Any other task assigned by the core team.

Field Investigator

- Field data collection.
- Entering of data into the software/Tablet.
- Ready to reside at study and Travel extensively in the field area.

- Coordination with the team and stakeholders involved in data collection activities.
- Work in coordination with local Govt. Officials and local representatives.
- Any other task assigned by the core team.

Laboratory Technician

- Ready to reside at study site and prepared for extensive field visits.
- Collection of venous blood, processing and storage in the field.
- Able to maintain cold chain protocol of sample transport and storage.
- Knowledge about safe disposal of Biomedical wastes in the field.
- Entering of data into the software/Tablet.
- Any other task assigned by the core team.

Multi Tasking Staff

- To help study related field/laboratory activities.
- To carry out the instructions given by the senior project staffs.

Mop role and function of scstrti

1. Provide intellectual support to RMRC and contribute in designing the project and guidance to carry out the work.
2. Issue necessary administrative request to the collectors/DM, PA-ITDA, Deans of Medical Colleges of the state and CDMPHO of the concerned districts to provide required support for data collection as per the study design by the research team.
3. Release Funds as per criteria agreed after submission of the phase wise report, SOE and recommendation of the technical committee.
4. Providing statistical data regarding distribution of different tribal communities along different blocks.
5. Provide necessary support systems in the field.
6. Liaising with district officials of the working for different tribal affairs in the district. (ITDA, District Welfare Officer, Block Extension Officer etc.)
7. External Monitoring of the field work.
8. Organize phase end review of the project implementation in presence of the Principal Investigator and both institution heads.

Mop local liaison

- Letters to Collectors of all districts regarding support for the team
- Letters to CDMOs for support, liaising and storage space for samples, Bio-medical waste management.
- Letters to SC&ST department for village list, support of District welfare officers, and local staff support.
- Rapports build up with village heads.
- Involving ASHAs and ANMs working in each village to reach the tribal household and acquire village/ cluster level information.
- Identification of NGOs working in the area; for support in rapport build up.
- Provision of reimbursement of ASHAs and ANMs for their cooperation in the field.
- Making a directory of all District level, Block Level and village level officials along with field workers (ASHAs/ ANMs etc.)

Director, RMRC-BB: Dr. Sanghamitra Pati
Director, SCSTRTI: Prof. (Dr.) A. B. Ota

↓

Management Committee
Dr. Subrat Kumar Palo, Scientist-D, Chairperson
Dr. Debdutta Bhattacharya, Scientist -D
Dr. Jyotirmayee Turuk, Scientist- C
Dr. Srikanta Kanungo, Scientist- C
Dr. Jaya Singh Kshatri, Scientist-C
Dr. Pranab Mohapatra, Assistant Professor (KIIMS)- External Member
Sh. Raj Kumar, Sr. AO
Sh. P Chatterjee, ACO

↓

Project Scientists
Programme Manager
Scientist C (Public Health)
Scientist C (Biochemistry)
Scientist C (Statistics)

↓

Field Supervisors (6)

↓

Field Investigators (06), Laboratory Technicians (12)

↓

Multitasking Task (1)

ORGANOGRAM & HR

Sexual Harassment

Sexual harassment will not be tolerated during OTFHS. By sexual harassment, we mean unwelcome sexual advances, requests for sexual favours, and other sexual comments or actions that make the receiver feel offended or intimidated. Sexual harassment may hurt work performance, and in some cases, an individual may feel that they must comply with the unwelcome advances or requests in order to keep their job. Sexual harassment can be committed by a man towards a woman, by a woman towards a man, or between two individuals of the same gender. To avoid any appearance of sexual harassment, individuals should be careful to avoid unnecessary physical contact and suggestive language and should maintain a professional work climate at all times. Anyone who feels that he or she has been the target of sexual harassment or who has witnessed an apparent incident of harassment should immediately report the incident to his or her supervisor, or to the survey manager. The implementing agency is required to investigate the claim and keep reports confidential to the extent possible. The implementing agency must take actions to prevent and correct harassing behaviour. These actions can include changing the workspace, reassigning interviewers or supervisors to different teams and other disciplinary actions. Retaliation against individuals filing complaints of sexual harassment will also trigger disciplinary action.

Social Media Policy

- No photo of field survey to be uploaded by team members on the social media without permission. Institutional Nodal Officer will upload or release any photo through official media.
- As per ICMR policy, the team members will not interact or provide information to media personnel. Any information inquired by the media is to be provided with the contact details of the managing Principal Investigator of the project or ICMR-RMRC nodal person.
- Any deviation from the policy as mentioned above will be strictly handled, and appropriate disciplinary action will be taken against them.

Annexures

1. Annexure-1

Sl No	Name of the Tribe	Total Population	Sample size	Households
1	Chenchu	13	13	4
2	Mankidi	31	31	8
3	Ghara	195	167	42
4	Baiga	338	228	57
5	Desua Bhumij	404	249	63
6	Korua	499	273	69
7	Birhor	596	292	73
8	Gandia	1854	389	98
9	Mankirdia	2,222	399	100
10	Madia	2,243	400	100
11	Kharwar	2265	400	100
12	Hill Kharia, Mankirdia and Birhor Development Agency, Jashipur, Mayurbhanj	3079	415	104
13	Chuktia Bhunjia	3086	415	104
14	Rajuar	3,518	420	105
15	Kol	4058	425	107
16	Kawar	5225	432	108
17	Koli, Malhar	6,423	437	110
18	Kotia	7,232	440	110
19	Bagata	8813	444	111
20	Didayi	8890	444	111
21	Parenga	9,445	445	112
22	Tharua	9,451	445	112
23	Kolah Loharas,	9,558	445	112
24	Dongria Kondh	9659	445	112
25	Lodha	9,785	445	112
26	Pentia	10,003	520	130
27	Binjhia, Binjhoa	11419	522	131
28	Bondo Poraja	12231	523	131
29	Bhunjia	12350	523	131

30	Kulis	13,689	524	131
31	Jatapu	14890	526	132
32	Dharua	18151	528	132
33	Banjara,Banjari	18257	528	132
34	Mahali	18,625	528	132
35	Kondadora	20,802	529	133
36	Dal	25598	531	133
37	Mundari	25,655	531	133
38	Kandha Gauda	26403	531	133
39	Holva	28149	531	133
40	Omanatya	28,736	531	133
41	Matya	30,169	532	133
42	Kutia Kondh	39761	533	134
43	Lanjia Soura	40913	533	134
44	Juang	47095	534	134
45	Kora	54,408	534	134
46	Paudi Bhuyan	61303	535	134
47	Mirdhas	75,940	535	134
48	Ho	80608	535	134
49	Gadaba	84689	536	134
50	Sounti	1,12,803	1072	259
51	Bhumia	125977	1072	259
52	Binjhal	137040	1072	259
53	Koya	1,47,137	1072	259
54	Bathudi	217395	1073	268
55	Kharia, Kharian	222844	1073	268
56	Bhumij	283909	1074	269
57	Bhuiya, Bhuyan	306129	1074	269
58	Kisan	331589	1074	269
59	Oraon	3,58,112	1074	269
60	Paroja	3,74,628	1074	269
61	Bhottada,Dhotada	450771	1074	269
62	Shabar, Lodha	5,16,402	1074	269

63	**Saora, Savar,**	**5,34,751**	**1074**	269
64	**Munda,**	**5,58,691**	**1074**	269
65	**Kolha**	**6,25,009**	**1074**	269
66	**Gond, Gondo**	**888581**	**1074**	269
67	**Santal**	**8,94,764**	**1074**	269
68	**Khond, Kond,**	**1627486**	**1075**	269
		9622744	42048	10489

Annexure-2

Selected Districts and Codes

Name of the district	District code	Name of block	Block code	Name of selected village	Cluster code
SAMBALPUR	SA	Bamra	01	Sagara	01
				Dehuripada	02
				Bamphei	03
				Paramanpur	04
				Charichuan	05
				Badapada	06
				Ghughar	07
				Parimunda	08
				Mahapatrabalanda	09
				Turei	10
		Kuchinda	02	Bauriguda(Rupadharbad)	01
				Brahmanidei	02
				Salebhadi	03
				Badmal	04
				Kirasasan	05
				Dansanadihi	06
		Jamankira	03	Ladampali	01
				Ghodabandhuni	02
				Godrapada	03
				Gariabahal	04
				Kasada	05
				Jamankira	06
				Chinimahul	07
				Patrapali	08
				Kutab	09
DEOGARH	DE	Teleibani	01	Bhaluguha	01
				Salohi	02
				Durijungle	03
				Kadodihi	04
				Prabhasuni	05

			07	Jhaliamara	06
				Danardanpali	07
				Palkudar	08
				Mundagohira	09
				Palunipada	10
				Dholpada	11
SUNDARGA RH	SU	Hemgir	01	Sanarampia	01
				Luabahal	02
				Buruta	03
		Subedga	02	Lahuraniberna	01
				Tedikaha	02
				Damkuda	03
		Ballisankara	03	Tileikani	01
				Sagjori	02
				Budabahal	03
		Lephipara	04	Didigajharan	01
				Aunlabahal	02
		Badagaon	05	Siamal	01
				Charpali	02
				Bhoipali	03
		Tangarpalli	06	Sanbeura	01
				Khuntagaon	02
				Bandhapali	03
		Kutra	07	Kalijapathar	01
				Karmabahal	02
				Jharbeda	03
		Rajgangpur	08	Kukudamunda	01
				Kesramal	02
				Jhagarpur	03
				Jaurumal	04
				Chhatam	05
		Sundargarh	09	Talsara	01
				Lankahuda	02
		Kuanrmunda	10	Dholkana	01
				Andali	02
				Banki	03

				Rampur	04
				Teliposh	05
		Bisra	11	Theteiposh	01
				Kopranda	02
				Barahabas	03
		Nuagaon	12	Jamdarah	01
				Kokerama	02
				Ghoghia	03
				Urmei	04
		Lathikata	13	Lungei	01
				Buchahanda	02
				Tainsar	03
				Manko	04
				Hatibandha (CT)	05
		Bonaigarh	14	Adadihi	01
				Deoposh	02
		Lahunipara	15	Kendudihi	01
				Tankajoda	02
				Talbahali	03
				Dhokamunda	04
		Gurundia	16	Raniberna	01
				Pankadihi	02
				Jalei	03
				Jharbeda	04
		Koida	17	Sanraksi	01
				Choredhara	02
				Ganua	03
Balasore	BA	Nilgiri	01	Purunagan	01
				Dobati	02
				Ajodhya	03
				Mainsapata	04
				Chatirikhunta	05
				Kantabania	06
				Jamudiha	07
				Dhobasila	08
				Sajanagarh	09

				Siadimal	10
				Bauriora	11
				Matiali	12
				Arbandh	13
				Dalimbapal	14
				Bhaunriabad	15
				Baunsapala	16
				Chandigarh	17
				Kunchibania	18
				Hatimunda	19
				Kishorechandrapur	20
				Rissia	21
Mayurbhanj	MA	Baripada	01	Hemchandrapur	01
				Sarujharan	02
		Badasahi	02	Managobindapur	01
				Dhobadhobani	02
				Agual	03
		Samakhunta	03	Badsol	01
				GaudRumacolonysahi	02
		Betanati	04	Saitpur	01
				Nuagan	02
				Dariha SamilHatudi	03
		Rasgovindpur	05	Gambharia	01
				Badampur	02
		Muruda	06	Ajana	01
		Bangiriposi	07	Bangriposi	01
				Mahulisul	02
				Joka	03
		Saraskana	08	Dumurdiha	01
				Dighi	02
		Kuliana	09	Sukhuapara	01
				Darkholi	02
				Dhadipada	03
		Suliapada	10	Jamubadi	01
		Khunta	11	Jadabani	01
				Kadapalasa	02

		Gopabandhunagar	12	Sarukana	01
				Pathurikata	02
		Kaptipada	13	Jayantipatta Samil Jamudiha	01
				Badbadi	02
				Talapokhari	03
				Bhandar	04
		Udala	14	Nuagaon	01
				Khuntapal	02
		Karanjia	15	Kerkera	01
				Saradha	02
		Raruan	16	Jamunti	01
		Joshipur	17	Kalasbandha	01
				Badajhilli	02
				Uttaransa	03
		Thakurmunda	18	Khaparkhai	01
				Nischintapur	02
				Banamunda	03
		Sukruli	19	Beguniabindha	01
				Fulguntha	02
		Bisoi	20	Jodia	01
				Arjunbilla	02
		Bijatola	21	Kuali	01
				Baliadhipa	02
		Kusumi	22	Chandida	01
				Chhanua	02
		Rairangpur	23	Hatia	01
		Tiring	24	Jadunathpur	01
				Janghia	02
		Bahalda	25	Bhaleidihi	01
				Kumbhirda	02
		Jamda	26	Guduta	01
				Dhalpur	02
Keonjhar	KE	Joda	01	Kolhabarapada	01
				Kolharaida	02
				Daduan	03

				HandiBhanga	04
		Champua	02	Satahalia	01
				Tolaknanda	02
				Moudi	03
				Champua	04
		Jhumpura	03	Haridajodi	01
				Teliarsala	02
				Ukhunda	03
		Patna	04	Kodakhamana	01
				Tangarpada	02
				Tando	03
		Ghatagaon	05	Jharbeda	01
				Paipani	02
				Parasurampur	03
				Chatia	04
				Ghatagaon	05
		Keonjhar	06	Dhanurjayapur	01
				Kasira	02
				Narasinghapur	03
				Tikarapada	04
				Gopinathapur(nuagaon)	05
		Saharpada	07	Tando	01
				Matiaguni alias gopalpur	02
				Belasarei	03
		Harichandanpur	06	Hunda	01
				Kanheigola	02
				Nipania	03
				Bautia	04
		Telkoi	07	Jata	01
				Lipinda	02
				Ajayapur	03
		Bansapal	08	Uparraiguda	01
				Rakam	02
				Jaladihi	03
				Danla	04
				Panasanasa	05

Gajapati	GA	Guma	01	Linga	01
				Sukei (Baranga Singi)	02
		Rayagada	02	Padasahi	01
				Belapadar	02
		R. Udayagiri	03	Jiranga	01
				R. Udayagiri	02
		Nuagada	04	Atarasing	01
		Mohana	05	Alliganda	01
				Juba	02
				Jamudiha	03
Kalahandi	KA	Th.Rampur	01	Kanjiguda	01
				Taragaon	02
				Ushamaska	03
				Majhigaon	04
				Bhitarguma	05
				Kumudabahal	06
				Tukuguda	07
				Badachhatrang	08
				Chirka	09
				Hadshil	10
				Bahadaghat	11
		Lanjigarh	02	Bhaluchanchara	01
				Kauguda	02
				Leptaguda	03
				Lanjigarh	04
				Jamchuan	05
				Talkalima	06
				Dangamundi	07
				Hatisal	08
				Kumudapadar	09
				Sanpajipita	10
Rayagada	RA	Gunupur	01	Labba	01
		Gudari	02	Jamba	02
		Padmapur	03	Nuagan	03
		Bishamkatak	04	Sauraguda	01
				Lotaguda	02

		Muniguda	05	Sudepadar	01
		Chandrapur	06	Kudumguda	01
		Ramnaguda	07	Srirampur	02
		Rayagada	08	Kutuli	01
				Champi	02
		Kolnara	09	Khuntibadi	01
				Salibi	02
		Kashipur	10	Nalachuan	01
				Mahantapatakhuri	02
				Kucher	03
Koraput	KO	Jeypore	01	Haridaput	01
				Dharnahandi	02
				Bankobija	03
				Kanjeipatraput	04
		Boriguma	02	Dengapadar	01
				Gaudaguda	02
				Jhilimili	03
				Kenduguda	04
				Maliguda	05
		Kotpad	03	Guali	01
				Dhamonahandi(Dhamanahandi)	02
				Nuagam(Thengguda kamalpadar)	03
				Chhatarla(Chotorla)	04
		Boipariguda	04	Majhiguda	01
				Goudaguda	02
				Dandabadi	03
				Badiniput	04
		Kundra	05	Bedapaunsi	01
				Jhodenga	02
		Koraput	06	Tola	01
				Khagodora	02
		Similiguda	07	Lunguri	01

				Kudi	02
		Pottangi	08	Sankar	01
				Sambai	02
				Galigapadar(Galigabadar)	03
		Nandapur	09	Akanta	01
				Hatibari	02
				Nandigan	03
		Dasmanthpur	10	Angarguda	01
				Tikiriguda	02
				Kankadaput	03
		Lamtaput	11	Bodapoda	01
		Narayanpatna	12	Dhaigura	01
				Bichlachua	02
				Bhasamjhola	03
		Laxmipur	13	Minapai	01
				Uskabhata	02
				Bhitaragada	03
		Bandhugaon	14	Kuntesu	01
				Kunjari	02
Malkangiri	MK	Malkangiri	01	Tangapali	01
				Jhileruguda	02
				Pandiripani	03
				Dengaguda	04
				Simagudi	05
				Limaguda	06
		Korkunda	02	Bankiguda	01
				Matapaka	02
				Chidupali	03
				Gurakhunta	04
				Silaiput	05
				Rajal konda	06
				Nilakhamar	07
				Badali	08
		Kalimela	03	Padganpalli	01
				Bapanpalli	02
				Singabaram	03

				Tigal	04
				Pendikonda	05
				Nilakota	06
		Podia	04	Nunurguda	01
				Semilibencha	02
				Alma	03
		Khairput	05	Govindapali	01
				Kuriguda	02
				Andrahal	03
				Sikhapali	04
		Kudumulugum a	06	Nakamamudi	01
				Papermenta	02
				Sunagoi	03
				Darlabeda	04
				Dangakunda	05
		Mathili	07	Bhandaripangam	01
				Tangaguda	02
				Tulasi	03
				Patraput	04
				Kansariput	05
				Sarangapali	06
				Mundaguda	07
				Dangarkhali	08
Nowrangpu r	NO	Nowrangpur	01	Chatahandi	01
				Bikrampur	02
		Tentulikhunti	02		01
		Papadahandi	03	Khajuri	01
				Tumberla	02
				Saraguda	03
		Nandahandi	04	Dongarbheja	01
				Usaripadar	02
		Kosagumuda	05	Ekori	01
				Karchamal	02
				Pikaddhanua	03
				Umargan	04
		Raighar	06	Maripara	01

				Budrapada	02
				Marangapalli	03
				Chalanpara	04
				Khuduku	05
				Sargiguda	06
		Umerkote	07	Tohara	01
				Umargan	02
				Saraguda	03
				Bamodeibhatta	04
		Chandahandi	08	Kodobhata	01
				Bandhokona	02
		Jharigaon	09	Tentuligan	01
				Ichhapur	02
				Badatemara	03
				Siunaguda	04
		Dabugaon	10	Patia	01
				Bodo-olma	02
Kandhamal	KD	Balliguda	01	Badrukia	01
		K.Nuagaon	02	Nuagan	02
		Daringibadi	03	Simanbadi	03
		Tumudibandh	04		04
		Kotagarh	05	Kotagada	05
		Tikabali	06		06
		G.Udayagiri	07	Katingia	07
		Raikia	08		08
		Chakapada	09		09
		Phulbani	10		10
		Phiringia	11	Senisuga	11
		Khajuripada	12	Dadapaju	12
Nuapada	NU	Komna	01	Salepada	01
				Chhinmundi	02

				Cherchuan	03
				Micchapali	04

Annexure-3

Household attempt form				
Sl No.	Household Id	Individual Id	Data Collected	Blood sample collected

			1st attempt	2nd attempt	3rd attempt	1st attempt	2nd attempt	3rd attempt
Filled by								
Verified by-								

Annexure-4

Household Listing

Sl No.	Name	Age	Gender	Household ID	Individual ID	Sample ID
Filled by						
Verified by-						

Annexure-4

Sample Log book

Sl No.	Household ID	Individual ID	Sample ID	Sample Collected	Serum Collected
Filled by					
Verified by-					

Annexure-5

Travel Log Book					
Sl No.	Date	Place	Starting time	Ending time	Total KM
Filled by					
Verified by-					

Annexure-6

Cluster Plan						
Sl No.	Name of the cluster	Week-1	Week-2	Week-3	Week-4	Contact Person

Annexure- 7

DISTRICT NAME-								
			MONTH-1		MONTH- 2		MONTH- 3	
**Sl no.**	**Consumables**	**Total Quantity Supplied**	**USED**	**LEFT**	**USED**	**LEFT**	**USED**	**LEFT**
1	Surgical Mask							

2	N -95							
3	Tourniquet							
4	Syringe (5 ml)							
5	Syringe (2 ml)							
6	Gel Vacutainer (5 ml)							
7	EDTA Tube (2 ml)							
8	Alcohol Swab							
9	Band Aid							
10	Rubber band / Zip Lock							
11	Face Shield							
12	Marker Pen							
13	White Gloves (medium)							
14	White Gloves (Large)							
15	Purple Gloves (medium)							
16	Purple Gloves (Large)							
17	Biohazard poly (Red & Yellow)							
18	Sanitizers							
19	Cotton							
20	Vial Stand							
21	Thermocol Box							
22	Vaccine container							
23	Gel pack							
24	Consent Form							
25	File							
26	Vial box							
27	Cryovial							
28	Tissue Roll							
29	Stamp pad							
30	Apron							
31	Handwash							
32	Notepad							
33	Pen Packet							

Annexure-7

**Monitoring checklist**			
Sl No.	Description	Yes/No	Remark

1	Measurement of height bare foot		
2	Measurement of weight bare foot		
3	Consent form completed prior to collection of sample		
4	Hand sanitized before the collection of blood sample		
5	Biomedical waste properly disposed in the biohazard bags		
6	The gel vacutainers are properly placed on the collection table in the vial stand		
7	The gel vacutainer is properly stored in the vaccine carrier		

ICMR-Regional Medical Research Centre, Bhubaneswar
Odisha Tribal Family Health Survey(OTFHS)

Referral Form for Anthropometric measurements and Biomarker Test

Sample ID : **Name :**

Age (in years) : **Gender:**

c) **Anthropometric measurement and Biomarker Test values**

**Sl No.**	**Parameter**	**Value**	**Normal range**
1	Height (in cm)		NA
2	Weight (in kg)		NA
3	Waist to Height ratio:<br>Waist Circumference/Hip Circumference(in cm)		Abdominal obesity:<br>Men: > 0.90; Women: > 00.0.85
4	Grip Strength Measurement (in kg)		Refer SOP of grip strength measurement for weak, normal and strong response
5	Blood Pressure:<br>Systolic/ Diastolic (in mmHg)		120/80 mmHg
6	Random Blood Sugar (mg/dL)		Non-diabetic: ≤200 mg/dL<br>Diabetes: > 200 mg/dL
7	Haemoglobin (g/dL)		Male: 13.5-18 g/dL<br>Female: 12-16 g/dL

d) SCD/SCT:

Haemoglobin A2/C/E	%
Haemoglobin S	%
Haemoglobin F	%
Haemoglobin A	%

(Signature of Lab Technician)

Date:

District:
